# Supplementary material for: Impact of the extension of a performance-based financing scheme to nutrition services in Burundi on malnutrition prevention and management among children below five: A cluster-randomized control trial
Source: PLoS One. 2020 Sep 18;15(9):e0239036. doi: 10.1371/journal.pone.0239036 (PMC7500612; doi:10.1371/journal.pone.0239036)
Supplement: S1 Table — Notes: N refers to the number of observations; n refers to the number of positive (yes to a categorical variable); SD refers to the standard deviation. Source: Authors. (DOCX) [file pone.0239036.s001.docx]

|  | N | Mean | SD |
| --- | --- | --- | --- |
| Household size (number of people) | 6199 | 5.64 | 0.04 |
| Number of children under 5 year per household | 6199 | 1.66 | 0.01 |
|  |  |  |  |
|  | **N** | **n** | **proportion** |
| Head of household has no activity | 5756 | 83 | 1% |
| Head of household activity is agriculture / farming / fishing | 5756 | 4727 | 82% |
| Head of household does not read or write | 6080 | 1779 | 29% |
| Head of household reads and writes | 6080 | 4181 | 69% |
| Head of household has no education | 6015 | 4049 | 67% |
| Head of household has primary level education | 6015 | 1706 | 28% |
| Head of household has secondary or tertiary level education | 6015 | 260 | 4% |
| Owns land for agriculture | 6195 | 5598 | 90% |
| Owns a motorized vehicle | 6199 | 127 | 2% |
| Owns a bicycle | 6199 | 1475 | 24% |
| Has electricity | 6199 | 288 | 5% |
| Owns soap | 6194 | 4013 | 65% |
| Water source: tap | 6199 | 192 | 3% |
| Water source: fountain | 6199 | 2497 | 40% |
| Water source: protected well or spring | 6199 | 2290 | 37% |
| Enough water for all purposes | 6197 | 4953 | 80% |
| Not enough water at all | 6197 | 1043 | 17% |
| Cooks with fire wood | 6199 | 5703 | 92% |
|  |  |  |  |
| Food insecurity |  |  |  |
| Proportion of households who have: |  |  |  |
| Food security | 6189 | 505 | 8% |
| Mild food insecurity | 6189 | 296 | 5% |
| Moderate food insecurity | 6189 | 1176 | 19% |
| Severe food insecurity | 6189 | 4212 | 68% |
|  |  |  |  |
| Feeding practices |  |  |  |
| Reports minimum number of food groups (meal diversity) | 5542 | 2732 | 49.3% |
| Reports minimum number of required meals (frequency) | 6164 | 2697 | 43.8% |
| Reports acceptable feeding practice (diversity and frequency) | 6144 | 1535 | 25.0% |
|  |  |  |  |
| Age and sex of surveyed child |  |  |  |
| 6-11 months | 6196 | 2234 | 36% |
| 12-17 months | 6196 | 2113 | 34% |
| 18-23 months | 6196 | 1849 | 30% |
| male | 6199 | 3028 | 49% |
