# Supplementary figures and images for: Impact of the extension of a performance-based financing scheme to nutrition services in Burundi on malnutrition prevention and management among children below five: A cluster-randomized control trial
Source: PLoS One. 2020 Sep 18;15(9):e0239036. doi: 10.1371/journal.pone.0239036 (PMC7500612; doi:10.1371/journal.pone.0239036)

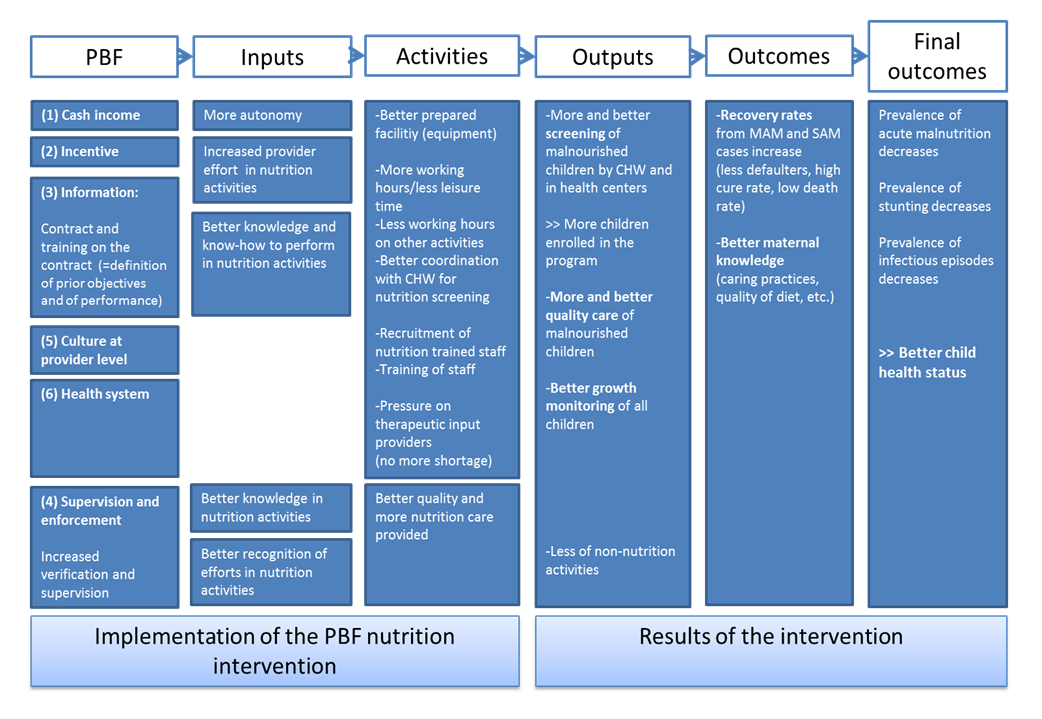

Supplement: S1 Fig — Source: Authors. (TIF) [file pone.0239036.s002.tif]
