# Supplementary material for: Impact of the extension of a performance-based financing scheme to nutrition services in Burundi on malnutrition prevention and management among children below five: A cluster-randomized control trial
Source: PLoS One. 2020 Sep 18;15(9):e0239036. doi: 10.1371/journal.pone.0239036 (PMC7500612; doi:10.1371/journal.pone.0239036)
Supplement: S2 File — Source: MSPLS. Note technique relative à l’intégration de la nutrition dans la stratégie nationale de financement basé sur la performance. 2013. (PDF) [file pone.0239036.s004.pdf]

Quality indicators specific to PBF-N

Growth monitoring (HC level)

1. Equipment for growth monitoring is available and functional (includes: measuring tool, weighing scale, child monitoring register, children clinical files, blank child health record booklets).
2. Proportion of children below two years old whom WHZ is above -2SD (maximum score if this proportion exceeds 97%; 0 if below 95%).
3. Proportion of children below two years old who attend growth monitoring and promotion sessions during the last three months (maximum score if above 90%).
4. For each individual growth monitoring and promotion session, we observed a discussion on the weight and height of the child, the mother or caregiver was asked whether she had concerns about the weight or the diet of their child, and they were given nutrition and development advices age-appropriate.
5. The weight for age and height for age growth curves are plotted in 90% of the child clinical files.

MAM service (HC level)

1. The inventory of the equipment for the MAM service exists and is actualised.
2. Equipment for the MAM service is available and functional (includes: measuring tool, mid-upper arm circumference tape, weighing scale, displayed MAM service admission and exit criteria, MAM service register, acute malnutrition management national guidelines, children weight for height tables).
3. Admission criteria are respected (in at least 9 out of the 10 randomly selected clinical files).
4. MAM service register is correctly filled in, according to requirements.
5. MAM cases are managed correctly, i.e. following the guidelines, regarding the identification and admission criteria and symptoms as well as management (20 clinical files selected randomly).
6. Performance criteria are all reached: average weight gain is above 2-3 grams per kg and day, average stay duration is less than two months, recovery rate is above 75%, defaulter rate is below 15%, death rate is below 3%, non-responding rate is below 10%).
7. MAM treatment and systematic treatment are available (includes: corn-soy blend, vitamin A, folic acid, albendazole/mebendazole, amoxycillin, artemisinin-based combination therapy).

Uncomplicated SAM service (HC level)

1. The inventory of the equipment for the uncomplicated SAM service exists and is actualised.
2. Equipment for the uncomplicated SAM service is available and functional (includes: measuring tool, mid-upper arm circumference tape, weighing scale, displayed uncomplicated SAM service admission and exit criteria, uncomplicated SAM service register, acute malnutrition management national guidelines, children weight for height tables).
3. Admission criteria are respected (in at least 9 out of the 10 randomly selected clinical files).
4. Uncomplicated SAM service register is correctly filled in, according to requirements.
5. Uncomplicated SAM cases are managed correctly, i.e. following the guidelines, regarding the identification and admission criteria, investigation and identification of complications, as well as management (20 clinical files selected randomly).
6. Uncomplicated SAM performance criteria are all reached: average weight gain is above 4-6 grams per kg and day, average stay duration is less than 30 days, recovery rate is above 75%, defaulter rate is below 15%, death rate is below 5%, transfer to hospital rate is below 10%, non-responding rate is below 10%).
7. Uncomplicated SAM treatment and systematic treatment are available (includes: Plumpy-Nut, vitamin A, folic acid, albendazole/mebendazole, amoxycillin, artemisinin-based combination therapy).

Complicated SAM service (hospital level)

1. The inventory of the equipment for the complicated SAM service exists and is actualised.
2. Equipment for the complicated SAM service is available and functional (includes: measuring tool, mid-upper arm circumference tape, weighing scale, displayed complicated SAM service admission and exit criteria, complicated SAM service register, acute malnutrition management national guidelines, children weight for height tables, graduated cups and basins).
3. Admission criteria are respected (in all of the 5 randomly selected clinical files).
4. Complicated SAM service register is correctly filled in, according to requirements.

5. Complicated SAM cases are managed correctly, i.e. following the guidelines, regarding the identification and admission criteria, investigation and identification of complications, diagnosis, management with at least one documented medical consultation per day (5 clinical files selected randomly).
6. Complicated SAM treatment and systematic treatment are available (includes: F-100 and F-75 therapeutic milks, ReSoMal oral rehydration solution, Plumpy-Nut, vitamin A, folic acid, albendazole/mebendazole, amoxycillin, artemisinin-based combination therapy).
