# Supplementary material for: Impact of the extension of a performance-based financing scheme to nutrition services in Burundi on malnutrition prevention and management among children below five: A cluster-randomized control trial
Source: PLoS One. 2020 Sep 18;15(9):e0239036. doi: 10.1371/journal.pone.0239036 (PMC7500612; doi:10.1371/journal.pone.0239036)
Supplement: S3 File — Source: Authors. (PDF) [file pone.0239036.s005.pdf]

Protocole de recherche

Etude d'impact du FBP Nutrition au niveau de la communauté au Burundi

Principaux investigateurs

ISTEEBU

IMT Blue Square

Banque Mondiale

Ministère de la Santé Publique et de la Lutte contre le SIDA

Version 4, du 29 Août 2014

Contenu

Résumé ... 2
Collaborateurs au Burundi et leurs rôles respectifs ... 3
Contexte et justification de l'étude et de l'enquête ... 3
Contexte ... 3
Health Results Innovation Trust Fund (HRITF) ... 3
Justification de l'étude ... 4
Le FBP et la gratuité des soins au Burundi ... 4
La nutrition au Burundi ... 4
Objectifs de l'évaluation d'impact du FBP Nutrition au niveau des ménages ... 5
Méthodologie ... 5
1. Echantillonnage ... 5
2. Mesures ... 7
3. Personnel et organisation pratique ... 8
4. Contrôle de qualité ... 9
5. Analyses ... 9
6. Ethique ... 10
Mise en œuvre de l'enquête ... 10
1. Pré-enquête ... 10
Pré-test et adaptation des questionnaires ... 10
Plan de travail de terrain ... 11
Achat de matériel ... 12
Programme de saisie des données (DEP) ... 12
Recrutement de personnel qualifié sur le terrain ... 13
Formation des agents enquêteurs ... 13
2. Travail de collecte sur le terrain ... 14
Organisation : personnel et durée de l'enquête ... 14
Gestion des données ... 14
3. Analyse et publication des résultats de l'enquête ... 15
4. Propriété des données ... 15
Equipes ... 16
L'équipe de préparation et collecte des données : l'ISTEEBU ... 16
L'équipe d'évaluation IMT Blue Square ... 16
Le MSPLS ... 17
La Banque Mondiale ... 17
Annexe ... 18
Calendrier / Diagramme de Gantt ... 18
Budget ... 18
Questionnaires ... 19
Questionnaires ménage ... 19
Questionnaires enfant ... 28
Demandes de consentement éclairé ... 38

Résumé

L'enquête EI-FBPNut-Mén I ici présentée s'inscrit dans le cadre de l'étude d'impact du volet Nutrition du Financement Basé sur la Performance (FBP) au Burundi. Cette étude d'impact s'inscrit dans le contexte d'une meilleure connaissance des résultats du FBP au Burundi, politique nationale de financement de la santé en vigueur depuis 2010, et profite de la mise en place d'un nouveau volet du FBP, à savoir le volet Nutrition, ajouté pour lutter contre la malnutrition, véritable fléau au Burundi. Cette étude permettra d'évaluer l'efficacité du FBP, mode de financement de la santé le plus utilisé au Burundi, et de comprendre dans quelle mesure ce mode de financement est plus ou moins efficace, particulièrement en matière de lutte contre la malnutrition.

Dans le cadre de cette étude, il est prévu d'effectuer des collectes de données primaires auprès des ménages ainsi qu'auprès des centres de santé nutritionnels (c-à-d disposant de services de supplémentation nutritionnelle et de thérapeutique ambulatoire). Notamment, une série de deux enquêtes auprès des ménages ayant des enfants âgés de 6-24 mois est prévue. Celle présentée ici est la première de la série et correspond à l'enquête de base (baseline) ; elle permet ainsi d'évaluer la sécurité alimentaire des ménages, l'état de santé et nutritionnel des enfants et les connaissances des parents y relatives, avant le démarrage du volet Nutrition dans le FBP.

Pour obtenir des informations statistiquement fiables, un total de 6,480 enfants âgés de 6-24 mois et leurs ménages seront sélectionnés pour l'enquête. La sélection se fera de la manière suivante. Un échantillon de 90 centres de santé nutritionnels sera tiré de manière aléatoire à partir de la liste des 193 centres de santé nutritionnels disposant des deux types de services nutritionnels : le service de supplémentation nutritionnel (SSN) pour la malnutrition aigüe modérée, et le service de thérapeutique ambulatoire (STA) pour la malnutrition aigüe sévère sans complications. Dans chaque aire de responsabilité de chacun de ces 90 centres de santé, 6 sous-collines seront tirées aléatoirement (échantillonnage auto-pondéré), soit 540 sous-collines au total. Dans chacune des sous-collines, 12 ménages avec enfants de 6-24 mois seront sélectionnés aléatoirement (échantillonnage en grappe), et se verront proposer l'enquête.

La collecte de données sera faite autour d'un module administré au chef de ménage ou à son conjoint, pour collecter notamment des informations sur la structure du ménage (2), le niveau socio-économiques du ménage (3), et la sécurité alimentaire du ménage (4) ; et d'un module administré à chaque enfant de 6-24 mois et un de ses parents, pour collecter des informations sur la nutrition et la santé de l'enfant (5), avec prise de mesures anthropométriques (6), et l'alimentation de l'enfant (7).

L'ensemble des données seront collectées électroniquement, à partir de smartphones Android et l'application Open Data Kit. Ainsi, la cohérence de ces données entrées sera vérifiée automatiquement, les données pourront être transférées régulièrement à Bujumbura, il n'y aura pas de travail de saisie/double-saisie, et la base de données pourra être consolidée et analysable dès la fin de la collecte de données sur le terrain.

Les données seront analysées par l'équipe mettant en œuvre l'évaluation d'impact, à savoir l'équipe de l'IMT Blue Square.

La réussite de l'étude d'impact du FBP Nutrition sera assurée à travers le suivi et le soutien d'un Comité de pilotage et d'un Comité technique mis en place au niveau du Ministère de la Santé Publique et de la Lutte contre le SIDA (MSPLS). La coordination technique de l'enquête auprès des centres de santé est confiée à l'Institut de Statistiques et d'Etudes Economiques du Burundi (ISTEEBU), avec l'appui technique de l'équipe de l'IMT Blue Square. Le processus de l'enquête EI-FBPNut-Mén I est prévu sur une période de 5 mois et coûtera 200,000 dollars US.

Collaborateurs au Burundi et leurs rôles respectifs

L'ISTEEBU rapportera directement au Task Team leader de la Banque Mondiale au Burundi et à l'équipe d'évaluation IMT Blue Square durant les étapes de préparation, de mise en œuvre de la collecte de données et de la livraison des données.

L'ISTEEBU sera responsable de l'exécution technique de l'enquête EI-FBPNut-Mén I, en particulier la préparation et la réalisation du travail sur le terrain, le traitement des données collectées et la rédaction des rapports d'enquête. L'ISTEEBU fournira les locaux nécessaires devant servir de bureau central pour le personnel de l'enquête. L'ISTEEBU sera responsable de la gestion du budget de l'enquête EI-FBPNut-Mén I. Il assurera aussi d'autres tâches administratives. Le personnel de l'ISTEEBU sera responsable de la supervision des opérations techniques quotidiennes, y compris le recrutement et la formation du personnel de terrain et de traitement et la supervision des activités de bureau et de terrain.

L'équipe d'évaluation IMT Blue Square assurera un suivi technique continu de la préparation et de l'exécution de l'enquête. L'équipe sera aussi responsable de l'analyse des données et de la diffusion des résultats au Burundi. A ce titre des ateliers sont prévus à l'automne 2014.

Le Ministère de la Santé Publique collabore à cette étude, car ses résultats lui seront utiles pour affiner sa politique de financement de la santé à travers le FBP et bien définir le volet nutrition du FBP. Des comités de pilotage et technique de l'étude seront instaurés.

Enfin, la Banque Mondiale assure le financement de l'enquête et de l'étude d'impact (y compris l'analyse de ces données).

En outre, le Comité National d'Éthique examinera le protocole proposé et devra l'approuver avant la réalisation de l'enquête.

Contexte et justification de l'étude et de l'enquête

Contexte

Health Results Innovation Trust Fund (HRITF)

Le HRITF vise à concevoir et mettre en œuvre des programmes pilotes de FBP durables qui améliorent efficacement les résultats de santé des mères et des enfants (c'est-à-dire les OMD 4 et 5). L'hypothèse est que le FBP peut être utilisé pour améliorer l'accès aux soins de santé

appropriés ainsi que la qualité des soins de santé et encourager les individus à adopter des bons comportements de santé.

Le HRITF finance les interventions pilotes de FBP – que ce soit du côté de l'offre (régimes de paiement fournisseur, sous-traitance) ou du côté de la demande (transferts conditionnels en espèces). Un élément central du HRITF est de mieux comprendre et documenter l'ampleur avec laquelle les politiques FBP sont efficaces, réalisables, et dans quelles circonstances. Par conséquent, une évaluation rigoureuse de tous les programmes de FBP de la santé est indispensable pour générer de nouvelles connaissances qui peuvent informer les gouvernements et les partenaires pour concevoir et utiliser efficacement les mécanismes de FBP.

C'est dans ce contexte que l'évaluation du FBP Nutrition au Burundi est réalisée.

Justification de l'étude

Le FBP et la gratuité des soins au Burundi
En 2006, face à des besoins en santé de la population croissants, le gouvernement a mis en place l'exemption des paiements directs pour les enfants de moins de 5 ans et les accouchements. Cette décision s'est heurtée à de nombreux problèmes, notamment de financement. Parallèlement, le Burundi expérimentait dans trois provinces une nouvelle stratégie de financement de la santé, le financement basé sur la performance (FBP), dont l'objectif était d'améliorer le système de santé. Après ces quelques expériences pilotes, le FBP s'est étendu à l'échelle du pays en avril 2010. Une décision importante prise par le gouvernement fut celle de fusionner le FBP et la gratuité des soins.

En 2012, avec le soutien de la Banque Mondiale, le gouvernement Burundais a renforcé les indicateurs existants concernant le planning familial en doublant les tarifs FBP. Il compte également introduire des indicateurs liés à la nutrition dans le FBP existant, ce qui est une opportunité à la fois pour améliorer le statut nutritionnel des enfants et pour réaliser une analyse rigoureuse des forces et des faiblesses du FBP.

La nutrition au Burundi
Au Burundi, la malnutrition est une barrière évidente à la réalisation des OMD liés à la santé. En effet, 58% des enfants de moins de 5 ans souffrent de malnutrition aigüe chronique. Suivant la tendance internationale, le Ministère de la santé du Burundi a développé en 2010 un protocole proposant un plan de traitement et de suivi de la malnutrition pour les enfants. Aujourd'hui, seulement un tiers des centres de santé et la moitié des hôpitaux offrent des services de prise en charge de la malnutrition.

L'intégration des services de nutrition dans le FBP est un défi important dans la mesure où actuellement, la mise en œuvre des services de nutrition est fortement dépendante d'organisations externes comme l'UNICEF ou le Programme Alimentaire Mondial. De plus, on constate que les services de nutrition sont négligés de par un manque de connaissances et de savoir-faire du personnel de santé et un manque de supervision des activités de nutrition. Aussi, l'introduction du FBP devrait permettre de faire reculer ces obstacles.

Objectifs de l'évaluation d'impact du FBP Nutrition au niveau des ménages

L'objectif ultime du programme de FBP Nutrition est d'améliorer le statut nutritionnel de la population. Cela sera possible à travers une amélioration des soins de santé, une plus rapide et meilleure identification de la malnutrition, un traitement mieux réalisé, et la mise en œuvre d'activités préventives dont l'objectif est d'améliorer les soins apportés par les parents à leurs enfants. L'évaluation d'impact du FBP Nutrition au niveau des ménages s'articule donc autour des questions de recherche suivantes :

- Le FBP Nutrition résulte-t-il en une réduction des taux de malnutrition aigüe et chronique dans la communauté ? Est-ce que cette réduction touche les populations de manière équitable (i.e. selon les différents niveaux de richesse, la géographie, etc.) ?
- Est-ce que le FBP Nutrition produit des externalités sur d'autres résultats de santé (ex. incidence des maladies, taux de vaccination, etc.) ?

L'évaluation sera basée sur une étude comparative randomisée, c'est-à-dire que le FBP Nutrition sera introduit dans un groupe de 45 CdS (groupe intervention) choisis aléatoirement. Les résultats de l'intervention dans les aires de responsabilité de ces CdS seront comparés à ceux obtenus pour un autre groupe de 45 CdS, similaires aux premiers, sans FBP Nutrition (groupe contrôle où l'organisation actuelle des soins est maintenue). Pour faciliter l'acceptabilité de l'étude par tous les CdS, le groupe d'intervention et le groupe de contrôle recevront les mêmes montants (sous forme de subsides dépendant de la performance pour les premiers, et sous forme de dotation fixée comme correspondant à la moyenne des subsides pour les seconds).

Les données d'enquêtes auprès des ménages seront collectées avant que l'intervention FBP Nutrition ne démarre (baseline, T0), autour de juin-juillet 2014 ; puis deux ans après que l'intervention ait été mise en œuvre (T2), autour de juin-juillet 2016.

Méthodologie

1. Echantillonnage

Un total de 6,480 enfants de 6-24 mois (et leurs ménages) seront sélectionnés pour participer à l'enquête. La sélection se fera de la manière suivante. Un échantillon de 90 centres de santé nutritionnels sera tiré de manière aléatoire à partir de la liste des 193 centres de santé nutritionnels disposant des deux types de services nutritionnels : le service de supplémentation nutritionnel (SSN) pour la malnutrition aigüe modérée, et le service de thérapeutique ambulatoire (STA) pour la malnutrition aigüe sévère sans complications. Les 90 CdS seront divisés en deux groupes, ceux bénéficiant du programme FBP Nutrition (groupe d'intervention), et ceux du groupe de contrôle (recevant une compensation sous forme de dotation financière). Les deux groupes de CdS seront constitués de façon à ce qu'ils soient similaires sur des paramètres essentiels définis à priori (taille de la population de couverture, volume des cas de malnutrition traités, volume du personnel) ; ils seront appariés en 45 paires. Au sein des 45 paires, l'allocation d'un des centres au groupe intervention (FBP Nutrition) se fera par randomisation simple, l'autre CdS de la paire étant automatiquement attribué au groupe contrôle (matching ou randomisation bloquée) : cette

opération sera réalisée lors d'un atelier fin mai 2014, où les 90 centres de santé seront représentés et tireront au sort leur statut : allocation du traitement ou contrôle. Etant donné la taille limitée du pays, certains des centres de santé pourront être proches les uns des autres géographiquement. Cependant, le risque de contamination entre les groupes contrôle et intervention est faible compte tenu de la nature de l'intervention. Il est en effet peu probable que des familles visitent préférentiellement les centres du groupe intervention parce que ceux-ci ont modifié leur organisation de soins suite à l'intervention (PBF). Il est aussi peu probable que les responsables de ces centres de santé « recrutent » des patients sur la zone contrôle. Par ailleurs, lors de l'enquête ménage, le nom du centre de santé visité lors d'un épisode de maladie de l'enfant est enregistré, ce qui permettra éventuellement de mesurer l'ampleur d'une quelconque contamination et d'ajuster les résultats pour ce facteur.

Dans chaque aire de responsabilité de chacun de ces 90 centres de santé (unités primaires de sondage), 6 sous-collines seront tirées aléatoirement (comme unités secondaires de sondage, USS), soit 540 sous-collines au total. La population des sous-collines a été recensée en 2008, et une mise à jour effectuée en 2013. La probabilité de sélection sera proportionnelle à la taille de la population de la sous-colline (échantillonnage auto-pondéré). Dans chacune des sous-collines (ou USS), 12 ménages avec enfants de 6-24 mois seront sélectionnés aléatoirement (échantillonnage en grappe), et se verront proposer l'enquête. Plus exactement, dans chaque sous-colline ou USS, un ménage sera aléatoirement tiré au sort sur base de la liste des ménages vivant dans cette USS. Les enquêteurs avanceront ensuite de proche en proche à partir de ce ménage jusqu'à ce que 12 enfants âgés de 6-24 mois soit recrutés. Cette méthode est préférée à un tirage aléatoire simple sur base de la liste des ménages afin d'éviter de nombreux déplacements. Ce processus nous amènera à un total de 6,480 enfants âgés de 6-24 mois. Pour maximiser la présence des ménages lors du passage des enquêteurs et éviter au maximum un éventuel biais de sélection, la date de visite sera communiquée à l'avance aux habitants de la sous-colline. Par ailleurs, la population sera sensibilisée au bien-fondé de l'enquête via des messages radiophoniques préalables.

La taille de l'échantillon est calculée sur base de la plus petite différence de résultat qui peut être considérée comme étant significative pour la santé publique, c-à-d une différence d'environ 25% dans la prévalence de la malnutrition aigüe (2.5 points de % en termes absolus) autour des centres de santé du groupe de traitement. En supposant que le programme FBP Nutrition va résulter en une réduction du taux de prévalence de malnutrition aigüe parmi les enfants âgés de 6 à 24 mois de 10%[1] à 7.5%, 3,240 enfants sont nécessaires dans chaque groupe (traitement et contrôle) pour une erreur de type α de 5% et une erreur de type β de 20%[2]. Le nombre de grappes est de 45 dans chaque bras (traitement et contrôle), avec 65 enfants par grappe. Ce nombre est augmenté à 72 enfants par grappe pour permettre des données manquantes ou incomplètes.

[1] Source: Enquête Démographie et Santé 2010.

[2] Calculé avec la formule de Hayes & Bennett (1999) : c (nb de grappes) = $1+ (z_\alpha/2 + z_\beta)^2[\pi0\ (1-\pi_0)/n + \pi1(1-\pi_1)/n+ k^2(\pi_0{}^2+\pi_1{}^2)]/(\pi0-\pi1)2$, avec n=nombre d'enfants par grappe, et k le coefficient de variation de grappe à 0.25.

Cette enquête sera reconduite en T2 autour des mêmes 90 centres de santé qui font partie de l'étude, mais pas auprès des mêmes enfants[3].

2. Mesures

Le principal résultat sera observé sur le taux de prévalence de malnutrition aigüe parmi les enfants de 6-24 mois. Le taux de malnutrition chronique est l'autre variable de résultat importante qui sera considérée. D'autres variables de résultats, secondaires, seront considérées, comme la prévalence d'infections durant les deux semaines qui ont précédé la visite, ou les habitudes alimentaires de l'enfant. Un indice de sécurité alimentaire sera construit pour chaque ménage. Enfin, un indice socio-économique sera également construit pour chaque ménage, comme variable de contrôle mais aussi pour étudier le niveau d'équité du programme de FBP Nutrition.

Les principales dimensions à mesurer sont résumées dans le tableau 1 ci-dessous ; les questionnaires complets sont disponibles en annexe.

A l'arrivée dans le ménage, l'équipe d'enquête s'assurera qu'il y a bien au moins un enfant de 6-24 mois dans le ménage et qu'il est présent au moment de l'enquête et que la personne qui s'occupe principalement de lui est également présente ; l'équipe s'assurera également que le chef de ménage ou son conjoint est bien présent et disponible à répondre[4]. Au total, deux questionnaires seront administrés au chef de ménage ou à son conjoint (questionnaires 2 et 3). Ensuite, on s'adressera à l'enfant (aux enfants) de 6-24 mois et à sa mère (leurs mères) ou à la personne qui s'occupe principalement de lui (d'eux) : les questionnaires 4 et 5 seront administrés à la mère ou à la personne qui s'occupe principalement de l'enfant, tandis que les mesures anthropométriques (questionnaire 5) seront prises uniquement sur l'enfant/les enfants ; autant que possible, les mesures du périmètre brachial des mères des enfants éligibles seront également prises (et utilisées comme variable de contrôle). Tous les enfants de 6-24 mois du ménage seront inclus. Dans le dernier ménage visité sur une sous-colline spécifique, tous les enfants de 6-24 mois seront inclus même si le nombre de 6 est déjà atteint. La durée totale du questionnaire, pour un ménage avec un enfant de 6-24 mois, est de 30 à 45 minutes ; il faut compter 20 minutes de plus par enfant supplémentaire.

[3] On s'intéressera à nouveau aux enfants de 6-24 mois, et ceux de la première vague en T0 auront grandi et ne seront donc plus éligibles lors de la seconde vague en T2

[4] Les enquêteurs expliqueront les objectifs de l'étude et proposeront au chef ou à son conjoint de signer la demande de consentement éclairé. Les ménages enquêtés auront bien entendu le droit de refuser de participer à l'enquête.

Tableau 1 : Données collectées

	**Dimensions**	**Questionnaire**	**Administration à**
Principal résultat	Prévalence de malnutritions aigüe et chronique (z-scores poids-pour-taille, taille-pour-âge, etc.)	#6	Enfants de 6-24 mois
Résultats secondaires	Etat de santé	#5	Mères des enfants de 6-24 m (ou personnes s'occupant principalement d'eux)
	Pratiques alimentaires des enfants	#4	Mères des enfants de 6-24 m (ou personnes s'occupant principalement d'eux)
Variables de contrôle	Indice de sécurité alimentaire	#3	Chef de ménage ou conjoint
	Caractéristiques socio-économiques	#2	Chef de ménage ou conjoint

Ces questionnaires ont été préparés au préalable par l'équipe d'évaluation d'impact de l'IMT Blue Square, en français. Ils seront testés et adaptés dans un premier temps par l'équipe IMT Blue Square. Puis, en consultation avec cette équipe, l'ISTEEBU adaptera les modules d'enquête au contexte du pays, y compris la formulation des questions et les codes de réponse afin d'être adaptés au contexte de l'étude. Une fois que l'ISTEEBU aura adapté le questionnaire français afin de tenir compte des besoins spécifiques du projet, l'équipe d'évaluation de l'IMT Blue Square traduira l'ensemble du questionnaire vers le kirundi ; l'ISTEEBU l'adaptera et le pré-testera, puis adaptera à nouveau le questionnaire en fonction des résultats du pré-test, afin de saisir avec précision l'information prévue sur les populations étudiées. La dernière version des questionnaires sera à nouveau traduite vers le français par un traducteur indépendant et formaté dans un format identique à celui utilisé pour la mise en œuvre effective.

Un cahier de procédures précisant dans le détail la collecte des données sur le terrain sera rédigé par IMT Blue Square. Ce manuel servira aussi pour la formation des enquêteurs.

3. Personnel et organisation pratique

Les enquêteurs seront organisés en équipes de 12 enquêteurs et 1 coordonnateur superviseur, idéalement un MD. Les enquêteurs travailleront par paires, et chaque paire administrera l'enquête à six ménages par jour.

Les équipes devront au préalable (1) annoncer aux chefs de colline et de sous-collines leur arrivée, (2) décrire l'étude et ses objectifs et expliquer comment les ménages sont sélectionnés pour l'étude. Six coordonnateurs-superviseurs de terrain seront responsables pour le coaching et la supervision de 6 paires d'enquêteurs chacun, et pour les aspects organisationnels avec la communauté et le ministère de la santé. Dix-huit (18) véhicules pouvant contenir quatre à cinq personnes (y compris le chauffeur) seront nécessaires aux équipes de collecte durant toute la période de collecte.

Avec 6 équipes (soit 72 enquêteurs et 6 coordonnateurs-superviseurs), la collecte des données des 6,480 enfants et leurs ménages dans les aires de responsabilités des 90 centres de santé sélectionnés prendra 30 jours ouvrés, soit cinq semaines (avec un jour de repos pas semaine).

A la fin de la journée, les données saisies sous Android seront transférées vers la plateforme ODK, et ainsi, le coordonnateur superviseur responsable de l'équipe, ainsi que les superviseurs basés à Bujumbura pourront les vérifier. Les données saisies sur le papier, s'il y en a, seront envoyées à Bujumbura à la fin de chaque semaine.

Chaque semaine, une réunion sera organisée par le coordonnateur-superviseur d'équipes terrain. Cela devrait donner à l'équipe l'occasion de discuter des problèmes liés à la supervision, à l'organisation du travail de terrain, des problèmes de saisie des données, etc., utiles pour le rapport intérimaire. En effet, des rapports d'étape hebdomadaires du nombre et des identifiants des ménages enquêtés avec succès seront réalisés chaque fin de semaine par le coordonnateur-superviseur.

Il devra y avoir une communication étroite entre le bureau central de l'ISTEEBU et le personnel de terrain pendant le travail de collecte. Les détails concernant la supervision et les communications seront discutés durant la formation et figureront dans les manuels de l'enquêteur.

4. Contrôle de qualité

Le contrôle de qualité sera assuré à travers la supervision et le suivi des équipes pendant les travaux sur le terrain. Les coordonnateurs-superviseurs d'équipes seront responsables de la qualité du travail de leurs équipes : ils tiendront des réunions régulières avec eux de façon à renforcer leur formation et à corriger les erreurs éventuelles commises au cours de la collecte. De plus, les coordonnateurs-superviseurs procéderont à des Lot Quality Assessment Sampling (LQAS) pour vérifier l'exactitude des données collectées, en effectuant des re-visites auprès de ménages enquêtés. Chaque superviseur contrôlera la qualité du travail d'une ou deux équipes chaque jour. Le jour de ce contrôle de qualité est choisi aléatoirement pour chaque équipe et est inconnu de l'équipe. La présence de plus de 3 erreurs sur un échantillon de 10 contre-mesures nécessitera un nouveau passage des enquêteurs et un recyclage des enquêteurs concernés.

Par ailleurs, l'équipe d'évaluation de l'IMT Blue Square engagera également deux superviseurs qui assureront un contrôle de qualité complémentaire. Aussi, deux personnes de l'encadrement technique, ainsi que la Direction du projet s'occuperont de la supervision au niveau de l'ISTEEBU.

5. Analyses

L'analyse des données d'enquête de baseline sera réalisée sous STATA. Elle permettra dans un premier temps de : (1) donner un vue d'ensemble de l'état nutritionnel des enfants de 6-24 mois et des pratiques alimentaires au Burundi, et (2) valider le design de l'étude, c'est-à-dire s'assurer que les ménages du groupe d'intervention sont comparables avec ceux du groupe de contrôle.

Dans un second temps, les données d'enquête de baseline seront couplées et analysées avec celles de l'enquête de suivi T2, i.e. deux ans plus tard, pour estimer l'impact de l'intervention 'FBP Nutrition' sur la prévalence de malnutrition aigüe et chronique, et autres résultats secondaires. Il s'agira d'estimations de différences-en-différences, comparant à la fois le 'avant-après' et le 'avec-sans' (intervention ou contrôle). Des modèles statistiques multiniveaux avec effets aléatoires au niveau du centre de santé seront utilisés. Les variables dépendantes continues seront analysées dans des modèles de régressions à effets mixtes, tandis que les variables catégorielles seront

analysées avec des modèles de régressions logistiques ou régressions de Poisson. D'autres facteurs de la malnutrition, comme la sécurité alimentaire du ménage, le statut socio-économique, et autres, seront utilisés comme variables de contrôle. Les interactions avec la saison, l'âge et le sexe de l'enfant, et les paramètres socio-économiques seront analysées. Une analyse de l'équité sera également réalisée afin de comprendre si le programme FBP Nutrition bénéficie plus aux ménages pauvres ou pas[5].

Se référer à la note conceptuelle de la recherche pour plus de détails sur les méthodes d'analyse.

6. Ethique

Le principal enjeu éthique dans cette étude réside dans le respect des parties qui seront sollicitées pour nous confier une information (le chef de ménage ou son conjoint, le parent de l'enfant et l'enfant lui-même) et le respect de la confidentialité de ces informations. Notre respect pour leurs droits passera par l'obtention d'un consentement éclairé.

En effet, les objectifs de l'enquête seront décrits par les enquêteurs aux membres du ménage. Ces derniers pourront librement accepter ou refuser de répondre aux questions sans devoir donner une motivation à ce refus. Pour ceux qui acceptent, un formulaire de consentement libre et éclairé sera signé (cf. Annexe). Ils auront le droit de ne pas répondre aux questions.

Par ailleurs, si les mesures anthropométriques de l'enfant détectent un cas de malnutrition (aigüe ou chronique), il sera conseillé au parent de l'enfant de se rendre dans les services appropriés.

Un autre enjeu éthique important résidera au niveau de la manipulation des données. Il est escompté de saisir un maximum de données sur des tablettes numériques. En phase de pré-analyse, ces données collectées seront anonymisées et la confidentialité de tout un chacun sera sauvegardée.

Enfin, la recherche s'inscrit dans une volonté de contribuer de façon pertinente et rapide au processus politique. Il est important de noter que cette étude d'impact s'inscrit dans un financement structurel par la Banque Mondiale du système de santé burundais. En cas de résultats confirmant l'efficacité de l'intervention, celle-ci sera mise à l'échelle sur l'ensemble du pays.

Mise en œuvre de l'enquête

1. Pré-enquête

Pré-test et adaptation des questionnaires

L'équipe d'évaluation de l'IMT Blue Square réalisera un pré-test de l'enquête EI-FBPNut-Mén I dès que les questionnaires auront été finalisés et traduits vers le kirundi. L'objectif du pré-test est de détecter d'éventuels problèmes dans les questionnaires, ainsi que d'évaluer le temps nécessaire pour conduire les interviews. La même équipe adaptera ensuite à nouveau le questionnaire en

[5] L'indice de richesse sera utilisé pour calculer des indices de concentration et dessiner des courbes de concentration. L'équité géographique pourrait également être analysée.

fonction des résultats du pré-test. La dernière version des questionnaires sera à nouveau traduite vers le français par un traducteur indépendant et formaté dans un format identique à celui utilisé pour la mise en œuvre effective.

Le test sera réalisé par trois membres burundais de l'équipe de l'IMT Blue Square (un médecin, un spécialiste en santé publique et un statisticien ayant de l'expérience en matière d'enquêtes), autour d'un centre de santé éligible.

Les résultats du pré-test seront utilisés pour modifier les instruments de l'enquête et les procédures de terrain, si nécessaire. On examinera de manière toute particulière la façon dont se sont déroulés les tests et on modifiera les procédures de terrain en conséquence. Toutes les décisions concernant les modifications après la pré-enquête devront être prises avec toutes les parties prenantes et validées par le comité technique.

Plan de travail de terrain

Les listes des 90 CdS de l'étude et des 540 sous-collines sélectionnées seront fournies par l'équipe d'évaluation IMT Blue Square, avec une description de la procédure d'échantillonnage. L'ISTEEBU devra ensuite collecter des listes de ménages habitant dans chaque sous-colline ; parmi eux, un ménage sera tiré au sort par sous-colline. Un plan de travail de terrain sera ensuite écrit pour décrire en détail la procédure de sélection des 12 ménages à enquêter par sous-colline, ainsi que tous les aspects du travail de terrain qui sera mené par la firme d'enquête, y compris :

- Diagramme de Gantt mis à jour et final
- Composition d'une équipe de terrain
    - Nombre d'agents enquêteurs
    - Nombre de superviseurs de terrain
    - Qualifications, formation de chacun
- Pour chaque membre d'équipe : taches prévues, responsabilités et calendrier des réalisations attendues
- La durée prévue que chaque équipe passera dans l'aire de responsabilité d'un CdS
- Transport et logistique d'hébergement
- Protocole afin de confirmer que l'emplacement des sous-collines et des ménages a été correctement identifié
- Procédures pour retrouver les enfants âgés de 6-24 mois absents lors du passage des enquêteurs
- Plans de supervision et de vérification pour garantir le respect des protocoles de collecte de données et confirmer la qualité de la collecte et saisie de données, comprenant au moins 10 re-visites auprès d'un échantillon aléatoire de l'échantillon de l'évaluation pour confirmer la validité des données
- Protocoles et procédures pour traiter les données incohérentes
- Protocoles pour la saisie des données sur les smartphones
- Protocoles pour la collecte de données au cas où le smartphone tombe en panne
- Protocoles de transmission de données vers le niveau central

Ce Plan de travail de terrain sera présenté à l'équipe d'évaluation IMT Blue Square pour commentaire et révisé au besoin avant de commencer le travail sur le terrain. L'ISTEEBU devra alors mettre en œuvre l'enquête, et adhérer étroitement au plan. Si les conditions de terrain imposaient des changements importants à ces plans, les superviseurs de terrain de la firme d'enquête seraient obligés d'informer l'équipe d'évaluation, sous la forme d'un rapport écrit.

Achat de matériel

Pour cette enquête, des smartphones Android seront nécessaires pour la collecte des données. Il est prévu que chaque paire d'enquêteurs en dispose d'un. Ainsi il faudra prévoir au minimum 36 smartphones Android pour l'enquête ; avec une marge de risque de 10%, il faudra s'en procurer 40 au total. Les modèles proposés ont les caractéristiques techniques suivantes :

- Samsung Galaxy Young
    - Numéro du modèle : GT-S6312
    - Version Android : 4.1.2
    - Version de la bande de base : S6312WWAMD1
    - Numéro de version: JZO54K.S6312XXAMI1
- Dual Samsung Galaxy Chat
    - Numéro du modèle : GT-B5330
    - Version Android: 4.1.2
    - Version de la bande de base: B5330XXUBME1
    - Numéro de version: JZO54K.B5330XXUBME1

Il sera également bon de prévoir des stylets pour la saisie sur écran tactile (si les modèle Samsung Galaxy Young est choisi).

Il faudra également un ordinateur portable par chef d'équipe sur le terrain (soit 7 au total si on considère une marge de risque), et deux ordinateurs de bureau au niveau de Bujumbura.

Des outils de mesures anthropométriques seront également nécessaires, à raison d'un set par paire d'enquêteurs. Trente-huit (38) sets seront donc nécessaires. Un set comprend :

- Une toise SECA 417 (ou toise UNICEF),
- Une balance SECA 877,
- Et un mètre de mesure SECA Girth ou SECA 212.

L'ISTEEBU fera le nécessaire pour obtenir ce matériel avant la formation des agents enquêteurs.

Programme de saisie des données (DEP)

L'équipe d'évaluation IMT Blue Square fournira au préalable le programme de saisie des données sous Open Data Kit, qui sera utilisé sur les smartphones Android des enquêteurs. L'interface utilisateur sera traduite en kirundi, et le programme sera adapté pour refléter les modifications apportées par le questionnaire de base suite au pré-test du questionnaire. Le programme adapté devra être robuste :

- Et la plage de saisie de données et les contrôles de cohérence devront être adaptés à des valeurs appropriées au contexte du Burundi, sur la base de données existantes.
- En effet, le programme de saisie des données devra effectuer des contrôles de cohérence (dans la mesure du possible) ; la violation de ces contrôles devra conduire à un message immédiat et transparent, ainsi qu'une méthode pratique pour corriger les erreurs, et à une documentation de toutes les réponses qui violent la cohérence.
- Le programme devra permettre des réponses ouvertes (texte) et des réponses « autres » en dehors des choix de réponses fournis dans le questionnaire.
- Les noms des variables générées par le programme devront correspondre clairement et logiquement aux étiquettes des questions utilisées dans le questionnaire.

Recrutement de personnel qualifié sur le terrain

L'ISTEEBU recrutera le personnel nécessaire pour cette enquête. Cela comprend :

Au niveau de l'équipe centrale :

- 1 gestionnaire de projet et de données, à temps plein
- 1 coordonnateur de bureau à temps partiel

Au niveau de chaque équipe sur le terrain (il y en aura six (6)) :

- 1 Coordonnateur superviseur (idéalement, médecin)
- 12 (6 x 2) enquêteurs

Au final soixante-douze (72) enquêteurs seront recrutés par l'ISTEEBU et organisés en six équipes. Chaque équipe sera composée d'un chef d'équipe coordonnateur superviseur, et de six paires d'enquêteurs.

Tous les candidats aux postes d'agents de terrain seront sélectionnés sur la base de leur maturité, de leur capacité à communiquer, de leur niveau d'instruction, de leur connaissance de la langue locale et de leur disponibilité à travailler loin de chez eux pour une période de près de cinq semaines. Tout sera fait pour engager du personnel de terrain qualifié dont le profil sera prédéfini par l'équipe technique de l'enquête.

Formation des agents enquêteurs

Tous les candidats suivront une formation d'une semaine, portant sur tous les aspects de l'enquête. Un nombre de candidats supérieur aux besoins seront formés de façon à pouvoir choisir les meilleurs éléments et, éventuellement, à faire des changements au cours des premiers jours de terrain. La formation aura lieu dans un cadre de capacité suffisante pour accueillir les candidats. L'ISTEEBU fournira les formateurs et l'équipe d'évaluation de l'IMT Blue Square les assistera.

L'équipe d'évaluation IMT Blue Square fournira le projet de formation (incluant matériel de formation) à l'ISTEEBU. L'ISTEEBU devra adapter le matériel au contexte local en consultation avec l'équipe d'évaluation. Si nécessaire, le matériel didactique et les manuels pratiques seront traduits en langue locale.

Le programme de formation comprendra : une description détaillée du contenu des questionnaires ; une présentation des techniques d'interview ; et une formation sur l'utilisation des smartphones Android et sur la façon de remplir les questionnaires sous Open Data Kit. Chaque enquêteur réalisera au moins six entretiens pendant la durée de formation.

Au moins une journée sera réservée aux chefs d'équipe, aux coordonnateurs et au personnel d'encadrement pour les former sur la façon d'observer les interviews sur le terrain, de vérifier, stocker et transférer vers Bujumbura les questionnaires remplis.

Le programme de formation inclura :

- *Théorique*: théorie du questionnaire et de chaque question afin de bien comprendre l'objectif de chaque question ; les techniques d'entretien et protocoles de terrain doivent également être couverts.
- *Pratique en classe*: exercices individuels et de groupe pour se familiariser avec la pratique de poser des questions et remplir un questionnaire.
- *Essai pilote*: après la théorie et la pratique en classe, les enquêteurs doivent aller sur le terrain pour administrer le questionnaire complet à un petit nombre de ménages (en dehors de l'échantillon de l'étude). Le pré-test ne se concentrera pas sur les ajustements majeurs au questionnaire, mais simulera plutôt l'administration du questionnaire dans des circonstances normales. L'essai-pilote devrait également servir de test pour l'utilisation des smartphones.
- *Évaluation*: A la fin de la formation, les étudiants doivent être évalués selon leur compréhension du questionnaire et leur capacité à enregistrer correctement les données en utilisant les mêmes scénarios de test comme utilisé dans la pratique en classe. La formation ne devrait se terminer qu'une fois que les équipes de terrain auront fait preuve de maîtrise des tâches désignées.

Après avoir terminé le stage de formation, tout agent de terrain sélectionné devra avoir une connaissance approfondie du rôle à jouer dans la collecte des données afin d'atteindre le maximum d'efficacité dans les travaux sur le terrain.

2. Travail de collecte sur le terrain

Organisation : personnel et durée de l'enquête

Cf. Section « 3. Personnel et organisation pratique » (page 8) et « 4. Contrôle de qualité » (page 9) pour ces points. Il est prévu 30 jours de travail de collecte afin d'obtenir les données sur les 6,480 ménages autour des 90 centres de santé sélectionnés.

Gestion des données

Chaque fin de journée, les données collectées sous Android seront transférées vers la plateforme internet ODK (cela sera néanmoins dépendant de la disponibilité du réseau 3G dans la zone où se trouveront les enquêteurs ; en cas d'indisponibilité du réseau 3G dans la zone, les données seront stockées sur disque dur et transmises ultérieurement); ainsi l'équipe de supervision basée à Bujumbura pourra y avoir accès. Chaque fin de semaine, les données éventuellement collectées sur

papier seront envoyées vers l'ISTEEBU Bujumbura. Tout cela servira notamment à la vérification des questionnaires, la codification des variables non pré-codées et in fine la préparation pour la consolidation et l'analyse.

L'ensemble des activités de traitement des données sera supervisé par le gestionnaire de données basé à l'ISTEEBU Bujumbura en collaboration avec un coordonnateur de l'équipe d'évaluation IMT Blue Square.

La saisie des données se fera sur smartphone Android en utilisant un programme (Open Data Kit) qui contrôle l'étendue des données et la logique des sauts du questionnaire, ainsi que la cohérence interne. Dans l'ensemble, l'édition des données comprendra la vérification des étendues, la structure des questionnaires et un ensemble de contrôle de cohérence interne. Toutes les erreurs détectées au cours du processus d'édition seront corrigées. Une fois que la vérification et l'apurement des données auront été terminées, les dates imputées seront ajoutées au fichier de données, les facteurs de pondération seront calculés et ajoutés au fichier de données et tous les tableaux nécessaires pour le rapport préliminaire et le rapport final seront produits et vérifiés.

3. Analyse et publication des résultats de l'enquête

L'ISTEEBU produira un rapport général sur la collecte des données ainsi que le dictionnaire des variables[6]. L'équipe d'évaluation IMT Blue Square analysera ensuite les données.

L'équipe d'évaluation IMT Blue Square préparera un rapport complet sur l'ensemble des résultats de l'enquête au niveau du pays, ainsi que sur la comparabilité des ménages du groupe de contrôle avec ceux du groupe d'intervention. Un premier jet de ce rapport sera produit courant septembre ; il sera finalisé et présenté au Burundi au cours de l'automne 2014.

Dès que le rapport final sera disponible, l'équipe d'évaluation IMT Blue Square conjointement avec l'équipe de l'ISTEEBU organiseront un séminaire national de présentation des résultats. Selon un plan de communication et de diffusion des données (cf. *Memorandum d'entente*), il sera organisé des séminaires régionaux afin de présenter les résultats provinciaux aux autorités locales.

4. Propriété des données

Un *memorandum d'entente* sur la propriété, l'accès et l'utilisation des données est prévu d'être signé par le Gouvernement du Burundi et la Banque Mondiale, tous deux co-propriétaires des données, ainsi que l'ISTEEBU, autorité statistique du Burundi et organisation conductrice de l'enquête, et l'IMT Blue Square, qui a conçu la méthodologie de l'étude et analysera les données dans ce cadre. Ce *memorandum d'entente* spécifie que les bases de données brutes devront en permanence rester confidentielles. En revanche, les données ne comportant aucune liste nominatives le seront provisoirement, tant que les résultats de l'étude d'impact –développés principalement par l'équipe IMT Blue Square– ne seront pas validés et diffusés par la Banque Mondiale et le MSPLS. Par la suite, elles ne seront plus confidentielles et pourront être partagées avec des chercheurs burundais ou

[6] Le dictionnaire des variables sera en fait très largement préalablement préparé par l'équipe d'évaluation IMT Blue Square, lors de la phase de préparation des masques de saisie sous ODK. L'ISTEEBU devra simplement le compléter compte tenu des données obtenues sur le terrain.

internationaux : ceux-ci devront pour cela soumettre une demande au MSPLS et à la Banque Mondiale, avant d'obtenir l'autorisation d'accès et d'analyse des données.

Equipes

L'équipe de préparation et collecte des données : l'ISTEEBU

Comme dit plus haut, l'ISTEEBU sera responsable de l'exécution de l'enquête, en particulier la préparation et la réalisation du travail sur le terrain, le traitement des données collectées et la rédaction des rapports d'enquête, et donc le responsable final de l'enquête. L'ISTEEBU fournira les locaux nécessaires devant servir de bureau central pour le personnel de l'enquête. L'ISTEEBU sera responsable de la gestion du budget de l'enquête EI-FBPNut-Mén I. Il assurera aussi d'autres tâches administratives. Le personnel de l'ISTEEBU sera responsable de la supervision des opérations techniques quotidiennes, y compris le recrutement et la formation du personnel de terrain et de traitement et la supervision des activités de bureau et de terrain.

L'équipe d'évaluation IMT Blue Square

L'équipe d'évaluation IMT Blue Square assurera un suivi technique continu de la préparation et de l'exécution de l'enquête. L'équipe sera aussi responsable de l'analyse des données et de la diffusion des résultats au Burundi. Le principal investigateur de l'étude globale au sein de l'équipe d'évaluation IMT Blue Square est Bruno Meessen, IMT. Le chercheur responsable et leader de l'étude analysant les données ménage ici présentées est Patrick Kolsteren, IMT, assisté de Dominique Roberfroid, IMT, et Catherine Korachais, IMT (cf. note conceptuelle).

L'équipe de conception méthodologique et d'analyse des données est en effet composée de deux experts en nutrition, tous deux basés à l'Institut de Médecine Tropicale d'Anvers (IMT) : Dominique Roberfroid, médecin et expert en nutrition et épidémiologie, ayant 13 ans d'expérience dans les pays à revenus faible et intermédiaire, et Patrick Kolsteren, médecin, pédiatre et expert en nutrition et santé de l'enfant avec 30 années d'expérience. Tous deux ont une expérience conséquente en méthodes d'évaluations qualitatives et quantitatives, notamment en essais contrôlés randomisés. Est également dans l'équipe Epco Hasker, médecin épidémiologiste avec plus de 15 ans d'expérience, notamment dans la conception de procédures d'échantillonnage, la collecte de données sur le terrain, et l'analyse de données ; son rôle dans ce projet est principalement d'assurer la qualité des données d'enquête. Deux économistes de l'IMT sont également fort impliqués dans cette évaluation d'impact : Catherine Korachais, chercheur ayant 10 ans d'expérience en économie du développement et de la santé et de solides compétences quantitatives ; et Bruno Meessen, chercheur avec 20 ans d'expérience en santé publique ; son expertise dans le financement des soins de santé dans les pays à faibles revenus, et notamment en financement basé sur la performance, permet de bien concevoir le design de la recherche et de déceler les implications politiques, et permettra de proposer de bonnes recommandations au Burundi.

Mais tout ceci ne peut se faire sans l'implication de chercheurs Burundais. En effet, à travers son partenariat avec Blue Square, l'équipe d'évaluation inclut deux chercheurs burundais, Léonard Ntakarutimana, expert en santé publique et parasitologue avec plus de 10 ans d'expérience en tant

que chercheur et conseiller politique de santé publique ; et Manassé Nimpagaritse, médecin et spécialiste en santé publique, avec 10 ans d'expérience. Ce dernier poursuit son doctorat sur le sujet, sous la supervision de Bruno Meessen (IMT) et de Jean Macq (Université Catholique de Louvain, Belgique), et en collaboration avec l'INSP. Enfin, l'équipe dispose également d'un expert en qualité de collecte de données, Désiré Munezero, démographe et statisticien avec plus de 5 années d'expérience dans la formation et la supervision de collecte de données, ainsi que la gestion des données. Son expertise sera utile pour la bonne réalisation de l'enquête.

Le MSPLS

Le Ministère de la Santé Publique collabore à cette étude, car ses résultats lui seront utiles pour affiner sa politique de financement de la santé à travers le FBP et bien définir le volet nutrition du FBP. Aussi, le MSPLS est copropriétaire des données (avec la Banque Mondiale). Des comités de pilotage et technique de l'étude sont instaurés dans ce cadre. Enfin, la participation de certains membres du MSPLS dans l'écriture des rapports et articles sera de mise.

La Banque Mondiale

La Banque Mondiale, à travers le *Health Results Innovation Trust Fund* (HRITF), est l'institution commanditaire de l'étude.

Aussi, c'est la Banque Mondiale qui finance l'intervention pilote de FBP Nutrition (dont la mise en œuvre dépend du Ministère chargé de la Santé), ainsi que l'évaluation d'impact de cette intervention. En effet, un élément central du HRITF est de mieux comprendre et documenter l'ampleur avec laquelle les politiques FBP sont efficaces, réalisables, et dans quelles circonstances. L'évaluation rigoureuse du programme de FBP Nutrition ici discutée permettra de générer de nouvelles connaissances qui peuvent informer le gouvernement du Burundi, mais aussi les autres gouvernements et partenaires pour concevoir et utiliser efficacement les mécanismes de FBP. Ainsi, la Banque Mondiale assure également le financement de l'enquête (assurée par l'ISTEEBU) et de l'étude d'impact (assurée par l'IMT Blue Square). Elle est ainsi copropriétaire des données (avec le MSPLS, cf. Memorandum d'Entente). Les membres burundais et internationaux de l'équipe de la Banque Mondiale pourront être impliqués dans la rédaction des rapports.

L'équipe de la Banque Mondiale se compose de la manière suivante :

- Health Sector Development Support Project task team:
    - Driss M. Zine-Eddine E. (Senior Health Economist, Task Team Leader, AFTHW)
    - Alain Desire Karibwami (Health Specialist, Co-Task Team Leader, AFTHE)
    - Tomo Morimoto (Operations Officer, AFTHW)
    - Clarette Rwagatore (Program Assistant)
    - Nicole Hamon (Language Program Assistant, AFTHW)
    - Rigobert Mpendwanzi (Short-Term Consultant, PBF Specialist, AFTHE)
    - Richard Shugugu (Short-Term Consultant, Demographer, AFTHE)
    - Lyse Kanyambo (Team Assistant)
- Health Nutrition and Population team (technical support):
    - Christel Vermeersch (Senior Economist, HDNHE)
    - Elisa Rothenbuhler (Health Economist, HDNHE)
    - Paul Jacob Robyn (Health Economist, AFTHW)

Annexe

Calendrier / Diagramme de Gantt

Planning de la préparation et collecte des données ici présentée

	Fév ' 14	Mars ' 14	Avril ' 14	Mai ' 14	Juin ' 14	Juil ' 14	Août ' 14	Sept ' 14	Oct. ' 14	Nov. ' 14
**Autorisation éthique**										
Protocole d'enquête adapté et soumis au Comité éthique										
Autorisation éthique, visa statistique, autorisation terrain										
**Préparation enquête et collecte**										
Pré-test du questionnaire & adaptation du questionnaire										
Protocole d'enquête final, plan écrit du travail de terrain										
Protocole de saisie des données, programme sous ODK										
Recrutement des enquêteurs et superviseurs										
Achat du matériel nécessaire réalisé										
Formation des enquêteurs										
Test pilote final à la fin de la formation										
Collecte des données							X	X		
Nettoyage et gestion des données										
Livraison des données (y c. dictionnaire et rapports de collecte)										
Travail d'analyse des données										
Rapport général, première version										
Rapport général, version finale, Présentation publique										

Note : En bleu, l'ISTEEBU est leader, en vert, c'est l'IMT Blue Square.

Planning des vagues de collectes de données : baseline et vagues de suivi

		Janvier	Février	Mars	Avril	Mai	Juin	Juillet	Août	Septembte	Octobre	Novembre	Décembre
Baseline (EI-FBPNut-Mén I)	2014								X	X			
	2015												
Suivi (EI-FBPNut-Mén III)	2016									X	X		

Budget

Il est prévu un budget de 200,000 USD pour cette enquête. Cela sera entièrement financé par la Banque Mondiale.

Questionnaires

Questionnaires ménage

1. Identification

**Q101.**	Combien d'enfants âgés de 6 à 24 mois vivent dans le ménage ?	Si >1, remplir le nombre correspondant de questionnaires enfants<br>Si 0, **Fin du questionnaire**	|__|
**Q102.**	Le chef de ménage ou son conjoint sont-ils présents et disposés à répondre?	1.Oui<br>0.Non **(Fin du questionnaire)**	|__|
**Q103.**	Les mères ou personnes s'occupant principalement des enfants de 6 à 24 mois sont-elles présentes et disposées à répondre ?	1.Oui, au moins une est présente et disposée à répondre<br>0.Non **(Fin du questionnaire)**	|__|
**Q104.**	Code FOSA (unité primaire de sondage)	Code	|__|__|
**Q105.**	Code de l'enquêteur principal (1)	Code	|__|__|
**Q106.**	Code de l'enquêteur secondaire (2)	Code	|__|__|
**Q107.**	Date de l'enquête	Jour/mois/année	|__|__|/|__|__|/|__|__|
**Q108.**	Province de résidence	Code	|__|__|
**Q109.**	Commune de résidence	Code	|__|__|
**Q110**	Colline de résidence	Code	|__|__|
**Q111**	Sous-colline de résidence	Code	|__|__|
**Q112**	Code ménage	Code	|__|__|
**Q113**	Nom du répondant principal (chef de ménage ou conjoint)	Ecrire le nom	____________________

2. Caractéristiques socio-économiques du ménage

CE QUESTIONNAIRE EST A ADMINISTRER AU REPONDANT PRINCIPAL (CHEF DE MENAGE OU CONJOINT)

**1.**	Combien de personnes vivent dans ce ménage ?	Nombre	|__|__|
**2.**	Combien d'enfants de moins de 5 ans vivent dans ce ménage ?	Nombre	|__|__|
**3.**	Combien de membres du ménage sont des personnes âgées de plus de 65 ans ?	Nombre	|__|__|
**4.**	Quel est votre sexe ?	1. Masculin<br>2. Féminin	|__|
**5.**	Quel est votre âge ?	En années	|__|__|
**6.**	Quel est votre état matrimonial ?	1. Marié ou vivant avec qqun<br>2. Veuf/veuve<br>3. Divorcé(e) ou séparé(e)<br>4. Jamais marié	|__|
**7.**	Au cours des 12 derniers mois, quelle a été le principal secteur d'activité économique dans lequel vous avez été actif (active) ?	01. Agriculture/ Elevage/ Pisciculture<br>02. Mines/ Carrières<br>03. Fabrication / industrie<br>04. Construction<br>05. Transport<br>06. Commerce/ Vente, Services<br>07. Education/ Santé<br>08. Administration<br>97. Autre, préciser<br>99. Aucun	|__|__|
**8.**	Savez-vous lire ou écrire ?	1. Lire et écrire<br>2. Lire seulement<br>3. Ni lire ni écrire	|__|
**9.**	Quel est le niveau de scolarisation le plus élevé que vous ayez atteint ?	0. Aucun niveau<br>1. Pré-primaire<br>2. Primaire<br>3. Secondaire 1e cycle<br>4. Secondaire 2e cycle<br>5. Supérieur<br>6. Ecole informelle<br>7. Ne sait pas	|__|
**10.**	Quelle est votre relation avec le chef de ménage ?	1. Chef de ménage **(passer à la question 17)**<br>2. Femme/mari<br>3. Fils/fille<br>4. Gendre/belle-fille<br>5. Petit-fils/petite-fille<br>6. Père/mère<br>7. beau-père/belle-mère<br>8. Frère/sœur<br>9. Sans lien de parenté<br>10. Ne sait pas<br>99. Autre lien, préciser.	|__|__|
**11.**	Quel est le sexe du chef de ménage ?	1. Masculin<br>2. Féminin	|__|
**12.**	Quel est l'âge du chef de ménage ?	En années	|__|__|

**13.**	Quel est l'état matrimonial du chef de ménage ?	1. Marié ou vivant avec qqun<br>2. Veuf/veuve<br>3. Divorcé(e) ou séparé(e)<br>4. Jamais marié		|__|
**14.**	Au cours des 12 derniers mois, quelle a été le principal secteur d'activité économique dans lequel le chef de ménage a été actif (active) ?	01. Agriculture/ Elevage/ Pisciculture<br>02. Mines/ Carrières<br>03. Fabrication / industrie<br>04. Construction<br>05. Transport<br>06. Commerce/ Vente, Services<br>07. Education/ Santé<br>08. Administration<br>97. Autre, préciser<br>99. Aucun		|__|__|
**15.**	Le chef de ménage sait-il lire ou écrire ?	1. Lire et écrire<br>2. Lire seulement<br>3. Ni lire ni écrire		|__|
**16.**	Quel est le niveau de scolarisation le plus élevé que le chef de ménage ait atteint ?	0. Aucun niveau<br>1. Pré-primaire<br>2. Primaire<br>3. Secondaire 1e cycle<br>4. Secondaire 2e cycle<br>5. Supérieur<br>6. Ecole informelle<br>7. Ne sait pas		|__|
**17.**	Dans ce ménage avez-vous :			
	L'électricité ?	1. Oui	0. Non	|__|
	Un poste de radio ?	1. Oui	0. Non	|__|
	Si oui, combien ?			|__|__|
	Un poste de télévision ?	1. Oui	0. Non	|__|
	Si oui, combien ?			|__|__|
	Un téléphone portable ?	1. Oui	0. Non	|__|
	Si oui, combien ?			|__|__|
	Un téléphone fixe ?	1. Oui	0. Non	|__|
	Si oui, combien ?			|__|__|
	Un réfrigérateur ?	1. Oui	0. Non	|__|
	Si oui, combien ?			|__|__|
**18.**	Est-ce qu'un membre de votre ménage possède :			
	Une montre ?	1. Oui	0. Non	|__|
	Si oui, combien en avez-vous dans votre ménage?			|__|__|
	Une bicyclette ?	1. Oui	0. Non	|__|
	Si oui, combien en avez-vous dans votre ménage?			|__|__|
	Une motocyclette/scooter ?	1. Oui	0. Non	|__|
	Si oui, combien en avez-vous dans votre ménage?			|__|__|
	Une charrette tirée par un animal ?	1. Oui	0. Non	|__|
	Si oui, combien en avez-vous dans votre ménage?			|__|__|
	Une voiture/camionnette?	1. Oui	0. Non	|__|
	Si oui, combien en avez-vous dans votre ménage?			|__|__|

**19.**	Est-ce qu'un membre de votre ménage a un compte dans une banque ou une autre institution financière ?	1. Oui 0. Non	|__|
**20.**	D'où provient principalement l'eau que boivent les membres de votre ménage ?<br><br>ENQUETEUR: VERIFIER SI POSSIBLE	1. Eau du robinet dans le logement<br>2. Eau du robinet dans la cour<br>3. Fontaine publique<br>4. Eau du robinet privé/voisin<br>5. Puits à pompe ou forage<br>6. Puits creusé protégé<br>7. Puits creusé non protégé<br>8. Eau de source protégée<br>9. Eau de source non protégée<br>10. Eau de pluie<br>11. Camion-citerne<br>12. Eau de surface (rivière/barrage/lac/mare/fleuve/canal d'irrigation)<br>13. Eau en bouteille<br>99. Autre, préciser	|__|__|
**21.**	Est-ce que vous traitez l'eau avant de la boire ?	0 = Non, l'eau est déjà traitée<br>1 = Non, je ne traite jamais l'eau<br>2 = Oui, parfois<br>3 = Oui, toujours<br>9 = Ne sait pas	|__|
**22.**	Achetez-vous de l'eau ?	0 = Non, ni pour l'eau de boisson ni pour l'eau hors boisson<br>1 = Oui, seulement pour l'eau de boisson<br>2 = Oui, pour l'eau de boisson et l'eau hors boisson<br>9 = Ne sait pas	|__|
**23.**	Votre source d'approvisionnement principale en eau est-elle disponible toute l'année ?	0 = Non<br>1 = Oui<br>9 = Ne sait pas	|__|
**24.**	Quelle est la distance estimée à votre point principal d'approvisionnement en eau de boisson ?	1 = < 500m 4 = < 30min<br>2 = 501-1000m 5 = 31-59min<br>3 = > 1km 6 = 1-2heures<br>9 = Ne sait pas 7 = > 2heures	|__|
**25.**	Est-ce que la quantité d'eau à laquelle vous avez accès par jour pour votre ménage (boisson et hors boisson) est suffisante ?	1 = Oui, pour la boisson et hors boisson<br>2 = Oui, seulement pour l'eau de boisson<br>3 = Oui, seulement pour l'eau hors boisson<br>4 = Non<br>9 = Ne sait pas	|__|
**26.**	Quel type de combustible votre ménage utilise-t-il principalement pour cuisiner ?	1. Electricité<br>2. Lignite/tourbe<br>3. Charbon de bois<br>4. Bois de chauffe<br>5. Paille/branchages/herbes<br>6. Résidus agricoles<br>7. Gaz<br>8. Pas de repas préparé dans le ménage<br>9. Autre, préciser	|__|
**27.**	Dans ce ménage, combien de pièces utilisez-vous pour dormir ?		|__|__|

**28.**	Dormez-vous sous une moustiquaire ?	0 = Non car je n'en ai pas<br>1 = Non mais il y en a dans le ménage<br>2 = Oui moustiquaire imprégnée<br>3 = Oui moustiquaire non imprégnée	|__|
**29.**	Est-ce qu'un membre de votre ménage possède des terres agricoles ?	1. Oui<br>0. Non	|__|
**30.**	Combien d'ares de terres cultivées les membres du ménage ont-ils ?		|__|__|
**31.**	Parmi les animaux suivants, combien votre ménage en possède-t-il ?		
	a) Vaches laitières ou taureaux ?		|__|__|
	b) Chèvres ?		|__|__|
	c) Moutons ?		|__|__|
	d) Porcs ?		|__|__|
	e) Volailles (poulet, canard, pigeon, dinde, etc. ?)		|__|__|__|
	f) Lapins ?		|__|__|
	g) Cobayes ?		|__|__|
**32.**	Quelles sont les principales activités qui ont fait vivre le ménage au cours des six derniers mois ?<br><br>VOIR LISTE DES CODES	a) La principale activité<br>b) La 2ème plus importante activité<br>c) La 3ème plus importante activité	|__|__| |__|__| |__|__|
**33.**	Quel est le principal matériau du sol ?<br><br>ENQUETEUR : VERIFIER	1. Terre /sable<br>2. Bouse / fumier / crottin<br>3. Planches en bois / palmes / bambou<br>4. Carreaux/carrelage<br>5. Ciment<br>6. Moquette<br>7. Parquet/ bois ciré<br>9. Autre, préciser	|__|
**34.**	Quel est le principal matériau du toit ?<br><br>ENQUETEUR : VERIFIER	1. Pas de toit<br>2. Chaume/palmes/feuilles<br>3. Mottes de terre<br>4. Palmes/bambou<br>5. Planches en bois<br>6. Métal/tôles<br>7. Béton/ Ciment<br>8. Tuiles céramiques/Ardoise industrielles<br>9. Bâches<br>10. Tuile locale<br>99. Autre, préciser	|__|__|

**35.**	Quel est le principal matériau des murs ?<br><br>ENQUETEUR : VERIFIER	1.Bambou avec boue<br>2.Bambou/palme/tronc<br>3.Blocs de ciment<br>4.Bois de récupération<br>5.Briques cuites<br>6.Briques en terre non cuite<br>7.Briques en terre non cuite mais recouverte<br>8.Ciment<br>9.Pas de murs<br>10.Pierres avec boue<br>11.Pierres avec chaux/ciment<br>12.Planches en bois/carton<br>13.Terre<br>99. Autre, préciser	|__|__|
**36.**	Avez-vous du savon dans le ménage ?<br><br>ENQUETEUR: VERIFIER (DEMANDER A VOIR)	0 = Non<br>1 = Oui mais pas vu<br>2 = Oui, vu<br>9 = Ne sait pas	|__|
**37.**	Quel type de toilettes les membres de votre ménage utilisent-ils habituellement ?<br><br>ENQUETEUR: VERIFIER	1. Chasse d'eau connectée à un système d'égout<br>2. Chasse d'eau connectée à une fosse sceptique<br>3. Chasse d'eau connectée à des latrines<br>4. Chasse d'eau connectée à quelque chose d'autre mais ne sait pas quoi<br>5. Latrines avec dalle lavable<br>6. Latrines avec dalle non lavable<br>7. Latrines sans dalle / avec trou ouvert<br>8. Toilettes / latrines suspendues<br>9. Pas de toilettes / nature<br>99. Autre	|__|__|
**38.**	Partagez-vous ces toilettes avec d'autres ménages ?	1. Oui<br>0. Non	|__|

Codes des activités / sources de revenus :
01 = Agriculture, produits vivriers
02 = Agriculture de rente
03 = Elevage (inclus vente du bétail et produits d'élevage)
04 = Pêche
05 = Exploitation minière
06 = Fabrication / Industrie
07 = Construction
08 = Transport
09 = Commerce / vente de services
10 = Education / santé
11 = Administration
12 = Pension
13 = Location de terres, parcelles
14 = Prêts
15 = Dons / Transferts d'argent ou nourriture
16 = Vente de l'aide
97 = Autre

3. Sécurité alimentaire du ménage

CE QUESTIONNAIRE EST A ADMINISTRER AU REPONDANT PRINCIPAL (CHEF DE MENAGE OU CONJOINT)

**1.**	Au cours des quatre dernières semaines, avez-vous été préoccupé parce que votre ménage n'avait pas assez de nourriture?	1. Oui<br>0. Non **(passer à Q3)**	|__|
**2.**	Avec quelle fréquence cette situation est-elle survenue au cours de ces quatre dernières semaines?	1. une ou deux fois<br>2. trois à 10 fois<br>3. plus de 10 fois	|__|
**3.**	Est-ce que vous-même ou un membre de votre ménage n'a pas pu manger les types de nourriture que vous préférez à cause d'un manque de ressources?	1. Oui<br>0. Non **(passer à Q5)**	|__|
**4.**	Avec quelle fréquence cette situation est-elle survenue au cours de ces quatre dernières semaines?	1. une ou deux fois<br>2. trois à 10 fois<br>3. plus de 10 fois	|__|
**5.**	Au cours des quatre dernières semaines, est-ce que vous-même ou un membre de votre ménage a mangé une variété limitée d'aliments parce que les ressources n'étaient pas suffisantes?	1. Oui<br>0. Non **(passer à Q7)**	|__|
**6.**	Avec quelle fréquence cette situation est-elle survenue au cours de ces quatre dernières semaines?	1. une ou deux fois<br>2. trois à 10 fois<br>3. plus de 10 fois	|__|
**7.**	Ces quatre dernières semaines, est-ce que vous-même ou un membre de votre ménage a mangé une nourriture que vous ne souhaitiez pas manger à cause du manque de ressources pour obtenir d'autres types de nourriture?	1. Oui<br>0. Non **(passer à Q9)**	|__|
**8.**	Avec quelle fréquence cette situation est-elle survenue au cours de ces quatre dernières semaines?	1. une ou deux fois<br>2. trois à 10 fois<br>3. plus de 10 fois	|__|
**9.**	Ces quatre dernières semaines, est-ce que vous-même ou un membre de votre ménage a mangé un repas plus petit que vous n'auriez souhaité parce qu'il n'y avait pas assez à manger?	1. Oui<br>0. Non **(passer à Q11)**	|__|
**10.**	Avec quelle fréquence cette situation est-elle survenue au cours de ces quatre dernières semaines?	1. une ou deux fois<br>2. trois à 10 fois<br>3. plus de 10 fois	|__|
**11.**	Ces quatre dernières semaines, est-ce que vous-même ou un membre de votre ménage a mangé moins de repas par jour parce qu'il n'y avait pas assez de nourriture?	1. Oui<br>0. Non **(passer à Q13)**	|__|

**12.**	Avec quelle fréquence cette situation est-elle survenue au cours de ces quatre dernières semaines?	1. une ou deux fois<br>2. trois à 10 fois<br>3. plus de 10 fois	|___|
**13.**	Ces quatre dernières semaines, est-il arrivé que le ménage soit sans nourriture du tout parce qu'il n'y avait pas de ressources pour en acheter?	1. Oui<br>0. Non **(passer à Q15)**	|___|
**14.**	Avec quelle fréquence cette situation est-elle survenue ces quatre dernières semaines?	1. une ou deux fois<br>2. trois à 10 fois<br>3. plus de 10 fois	|___|
**15.**	Ces quatre dernières semaines, est-ce que vous-même ou un membre de votre ménage est allé au lit en ayant faim parce qu'il n'y avait pas assez de nourriture?	1. Oui<br>0. Non **(passer à Q17)**	|___|
**16.**	Avec quelle fréquence cette situation est-elle survenue ces quatre dernières semaines?	1. une ou deux fois<br>2. trois à 10 fois<br>3. plus de 10 fois	|___|
**17.**	Ces quatre dernières semaines, est-ce que vous-même ou un membre de votre ménage a passé toute une journée (càd ni le jour ni la nuit) sans manger parce qu'il n'y avait pas assez de nourriture?	1. Oui<br>0. Non **(passer à Q19)**	|___|
**18.**	Avec quelle fréquence cette situation est-elle survenue ces quatre dernières semaines?	1. une ou deux fois<br>2. trois à 10 fois<br>3. plus de 10 fois	|___|
**19.**	A présent, j'aimerais vous poser des questions sur la nourriture dont disposait votre ménage lors de différents mois de l'année. Quand vous répondez à ces questions, pourriez-vous y réfléchir sur les 12 derniers mois ?<br><br>Lors de ces douze derniers mois, y a-t-il eu des mois pendant lesquels vous n'aviez pas assez de nourriture pour répondre aux besoins de votre ménage ?	1.Oui<br>0.Non **(fin quest. ménage)**<br>9. Ne sait pas	|___|
**20.**	Quels étaient les mois (de ces 12 derniers mois) pendant lesquels vous n'aviez pas assez de nourriture pour répondre aux besoins de votre ménage?<br><br>NE PAS LIRE LA LISTE DES MOIS ; COCHER LES MOIS CITES	1. le ménage n'avait pas assez à manger pendant ce mois<br><br>0. le ménage a eu assez à manger pendant ce mois	
**20.a**	Janvier		|___|
**20.b**	Février		|___|
**20.c**	Mars		|___|
**20.d**	Avril		|___|
**20.e**	Mai		|___|
**20.f**	Juin		|___|
**20.g**	Juillet		|___|
**20.h**	Août		|___|
**20.i**	Septembre		|___|
**20.j**	Octobre		|___|
**20.k**	Novembre		|___|
**20.l**	Décembre		|___|

Questionnaires enfant

ENQUETEUR, REMPLIR UN QUESTIONNAIRE POUR CHAQUE ENFANT ELIGIBLE, C'EST-A-DIRE POUR CHAQUE ENFANT AGE ENTRE 6 MOIS ET 24 MOIS. CE QUESTIONNAIRE EST A ADMINISTRER A LA MERE OU LE CAS ECHEANT A PERSONNE QUI S'OCCUPE PRINCIPALEMENT DE L'ENFANT, EN PRESENCE DE L'ENFANT.

DEMANDER LE CARNET/FICHE DE VACCINATION DE L'ENFANT.

**Q104**	Code FOSA (unité primaire de sondage)	Code	|__|__|
**Q105**	Code de l'enquêteur principal (1)	Code	|__|__|
**Q106**	Code de l'enquêteur secondaire (2)	Code	|__|__|
**Q107**	Date de l'enquête	Jour/mois/année	|__|__|/|__|__|/|__|__|
**Q108**	Province de résidence	Code	|__|__|
**Q109**	Commune de résidence	Code	|__|__|
**Q110**	Colline de résidence	Code	|__|__|
**Q111**	Sous-colline de résidence	Code	|__|__|
**Q112**	Code ménage	Code	|__|__|
**Q401**	Nom du répondant (mère ou personne qui s'occupe principalement de l'enfant)	Ecrire le nom	______________________
**Q402**	Nom de l'enfant	Ecrire le nom	______________________
**Q403**	Code enfant	Code	|__|__|

**Q404**	Quelle est votre relation avec l'enfant ?	1. Mère<br>2. Père<br>3. Beau-père/belle-mère<br>4. Grand-père/Grand-mère<br>5. Oncle/Tante<br>6. Frère/Sœur<br>7. Autre, préciser.	|__|
**Q405**	LE REPONDANT EST IL DIFFERENT DU REPONDANT AUX QUESTIONNAIRES 2 ET 3 ?	1. Oui **(→ Lui poser les questions suivantes)**<br>2. Non **(→ Q1)**	|__|
**Q406**	Quelle est votre relation avec le chef de ménage ?	1. Chef de ménage<br>2. Femme/mari<br>3. Fils/fille<br>4. Gendre/belle-fille<br>5. Petit-fils/petite-fille<br>6. Père/mère<br>7. beau-père/belle-mère<br>8. Frère/sœur<br>9. Sans lien de parenté<br>10. Ne sait pas<br>99. Autre lien, préciser.	|__|__|
**Q407**	Quel est votre sexe ?	1. Masculin<br>2. Féminin	|__|
**Q408**	Quel est votre âge ?	En années	|__|__|
**Q409**	Quel est votre état matrimonial ?	1. Marié ou vivant avec qqun<br>2. Veuf/veuve<br>3. Divorcé(e) ou séparé(e)<br>4. Jamais marié	|__|
**Q410**	Au cours des 12 derniers mois, quelle a été le principal secteur d'activité économique dans lequel vous avez été actif (active) ?	01. Agriculture/ Elevage/ Pisciculture<br>02. Mines/ Carrières<br>03. Fabrication / industrie<br>04. Construction<br>05. Transport<br>06. Commerce/ Vente, Services<br>07. Education/ Santé<br>08. Administration<br>97. Autre, préciser<br>99. Aucun	|__|__|
**Q411**	Savez-vous lire ou écrire ?	1. Lire et écrire<br>2. Lire seulement<br>3. Ni lire ni écrire	|__|
**Q412**	Quel est le niveau de scolarisation le plus élevé que vous ayez atteint ?	0. Aucun niveau<br>1. Pré-primaire<br>2. Primaire<br>3. Secondaire 1e cycle<br>4. Secondaire 2e cycle<br>5. Supérieur<br>6. Ecole informelle<br>7. Ne sait pas	|__|

4. Etat nutritionnel et de santé de l'enfant

UN QUESTIONNAIRE PAR ENFANT ELIGIBLE (6-24 MOIS)

**1.**	Quel est le sexe de l'enfant ?	1. Masculin<br>2. Féminin	|__|
**2.**	Quelle est la date de naissance de l'enfant ?	*JJ/MM/AA*<br>99.Ne sait pas	|__|__|/|__|__|/<br>|__|__|
**3.**	Quel est l'âge de l'enfant en mois ?	Age en mois<br>99.Ne sait pas	|__|__|
**4.**	La mère biologique vit-elle dans le ménage ?	1. Oui, c'est moi<br>2. Oui, mais ce n'est pas moi<br>0. Non, elle vit ailleurs<br>9. Non, elle est décédée	|__|
**5.**	L'enfant fréquente-t-il un centre de santé ? (consultations préventives et curatives)	1. Oui<br>0. Non	|__|
**6.**	Quel centre de santé fréquente-t-il le plus régulièrement ?	Nom FOSA :<br><br>Code FOSA :	________<br><br>|__|__|__|
**7.**	A quelle distance se trouve ce centre de santé ?	Km :<br>Mètres, si <1 km :	|__|__| |__|__|__|
**8.**	Combien de temps mettez-vous pour vous rendre au centre de santé ?	Heures :<br>Minutes :	|__| |__|__|
**9.**	Au cours des 2 dernières semaines, l'enfant a-t-il été malade ?	1. Oui<br>0. Non **(→ Q14)**	|__|
**10.**	Si oui, avez-vous consulté le centre de santé?	1. Oui<br>0. Non	|__|
**11.**	Si oui, combien de fois ?		|__|
**12.**	La première fois, l'enfant a-t-il eu :<br>a) Diarrhée (selles liquides 3 fois/jours ou plus) ?<br>b) Fièvre ?<br>c) Infections respiratoires (toux persistante) ?<br>d) Autre, préciser	 1. Oui 0. Non<br>1. Oui 0. Non<br>1. Oui 0. Non	 |__| |__| |__| _________
**13.**	La deuxième fois, l'enfant a-t-il eu :<br>a) Diarrhée (selles liquides 3 fois/jours ou plus) ?<br>b) Fièvre ?<br>c) Infections respiratoires (toux persistante) ?<br>d) Autre, préciser	 1. Oui 0. Non<br>1. Oui 0. Non<br>1. Oui 0. Non	 |__| |__| |__| _________
**14.**	L'enfant est-il inscrit dans un programme de supplémentation nutritionnelle (SSN) ou de thérapeutique ambulatoire (STA) ?	1. Oui<br>0. Non	|__|
**15.**	Si oui, dans quel centre de santé se rend-il ?	Nom FOSA<br><br>Code FOSA	________<br><br>|__|__|__|
**16.**	A quelle distance se trouve ce centre de santé ?	Km :<br>Mètres, si 0 km :	|__|__| |__|__|__|
**17.**	Combien de temps mettez-vous pour arriver ce centre de santé ?	Heures :<br>Minutes :	|__| |__|__|

**18.**	Si ne se rend pas au (NOM de la FOSA unité primaire de sondage) : pourquoi n'allez-vous pas à ce (NOM de la FOSA unité primaire de sondage) » ?<br><br>POSSIBILITE DE COCHER PLUSIEURS REPONSES	1. Trop loin<br>2. Trop cher<br>3. Le personnel n'est pas agréable<br>4. N'ont pas de nourriture<br>5. Ne connaissait pas<br>6. Autre (à préciser)	|__| |__| |__| <br>|__| |__| |__|
**19.**	Pouvez-vous me montrer le carnet/fiche de vaccination de l'enfant ?	1. Oui<br>0. Non **(→Q25)**	|__|
**20.**	L'enfant a-t-il reçu les vaccins suivants ?<br><br>A COMPLETER A PARTIR DU CARNET/FICHE DE VACCINATION PAR L'ENQUETEUR		
	BCG ?	1. Oui 0. Non 9. Non renseigné	|__|
	Rougeole ?	1. Oui 0. Non 9. Non renseigné	|__|
	Polio(1-3) ?	1. Oui 0. Non 9. Non renseigné	|__|
	Pentavalent(1-3)	1. Oui 0. Non 9. Non renseigné	|__|
	Rota (1-2)	1. Oui 0. Non 9. Non renseigné	|__|
	PCV13(I-III)	1. Oui 0. Non 9. Non renseigné	|__|
	VAR1 ?	1. Oui 0. Non 9. Non renseigné	|__|
	VAR2	1. Oui 0. Non 9. Non renseigné	|__|
	Autre ?	1. Oui 0. Non 9. Non renseigné	|__|
**21.**	Combien de fois l'enfant a-t-il été pesé depuis sa naissance ?<br><br>A COMPLETER A PARTIR DU CARNET/FICHE DE VACCINATION PAR L'ENQUETEUR	Nb de fois :	|__|__|
**22.**	Quelle est la forme de la courbe de croissance ?<br><br>A COMPLETER A PARTIR DU CARNET/FICHE DE VACCINATION PAR L'ENQUETEUR	1.Ascendante<br>2.Stationnaire<br>3.Descendante<br>4. Pas de courbe sur le carnet	|__|
**23.**	SI POIDS DE NAISSANCE INSCRIT DANS LE CARNET, LE NOTER	En kilos (avec deux chiffres après la virgule)	|__|__|.|__|__|
**24.**	VERIFIER LA DATE DE NAISSANCE A PARTIR DU CARNET DE VACCINATION.	Jour/Mois/Année<br>99 99 99 = Non renseigné	|__|__|/|__|__|/|__|__|

5. Alimentation de l'enfant

UN QUESTIONNAIRE PAR ENFANT ELIGIBLE (6-24 MOIS)

**25.**	L'enfant a-t-il été allaité au sein ?	1. Oui<br>0. Non **(fin du questionnaire)**<br>9. Ne sait pas	|__|
**26.**	Combien de temps après la naissance avez-vous mis l'enfant au sein pour la première fois ?	1. Immédiatement après la naissance<br>2. Dans les 30 premières minutes<br>3. Après 30 minutes<br>4. Après 1 heure<br>5. Après 2 heures<br>6. Après 6 heures<br>7. Après 12 heures<br>8. Après 24 heures<br>9. Ne sait pas	|__|
**27.**	Allaitez-vous encore l'enfant au sein ?	1. Oui **(→ Q 39)**<br>0. Non	|__|
**28.**	A partir de combien de mois avez-vous cessé de donné le sein à l'enfant ?	Nombre de mois<br>99. Ne sait pas	|__|__|
**29.**	Pour quelles raisons avez-vous cessé d'allaiter l'enfant ?<br><br>QUESTION A CHOIX MULTIPLE	1. Fait tomber vite le sein<br>2. Crevasse au sein<br>3. Maladie de l'enfant<br>4. Maladie de la mère<br>5. Grossesse<br>6. Reprise du travail de la mère<br>7. Refus de téter<br>8. Pas de lait<br>9. Préfère le lait artificiel<br>10. Fait mal aux mamelons<br>11. Age de sevrage<br>12. Reprise de la relation sexuelle<br>13. Utilisation de contraceptif<br>99. Autres (à préciser)	|__|__| |__|__| |__|__| |__|__| |__|__| |__|__| |__|__| |__|__| |__|__| |__|__| |__|__| |__|__| |__|__|
**30.**	A la naissance de l'enfant, lui avez-vous donné le premier lait maternel de couleur jaunâtre appelé Colostrum ?	1. Oui<br>0. Non<br>9. Ne sait pas	|__|
**31.**	Depuis hier à la même heure que maintenant, combien de fois l'enfant a-t-il tété ?	1. Moins de 3 fois<br>2. 3 à 7 fois<br>3. 8 fois ou plus<br>7. n.a. (a arrêté l'allaitement)	|__|

**32.**	A quel moment allaitez-vous l'enfant ?<br><br>PLUSIEURS REPONSES SONT POSSIBLES	1. A la demande<br>2. Selon ma disponibilité<br>3. A des moments précis<br>4. Quand le sein pèse<br>7. n.a. (a arrêté l'allaitement)<br>9. Autres à préciser	|__| |__| |__| |__| |__| |__|
**33.**	Depuis hier à la même heure que maintenant, l'enfant a-t-il reçu:		
	a)Suppléments de vitamines, minéraux ou médicaments ?	1. Oui 0. Non 7. N.a. 9. Ne sait pas	|__|
	b)Eau ?	1. Oui 0. Non 7. N.a. 9. Ne sait pas	|__|
	c)Eau sucrée, jus de fruits, thé?	1. Oui 0. Non 7. N.a. 9. Ne sait pas	|__|
	d)Solution de réhydratation orale (SRO) ?	1. Oui 0. Non 7. N.a. 9. Ne sait pas	|__|
	e)Préparation pour bébé vendue commercialement ?	1. Oui 0. Non 7. N.a. 9. Ne sait pas	|__|
	f)Lait en boite, en poudre ou frais ?	1. Oui 0. Non 7. N.a. 9. Ne sait pas	|__|
	g)Autres liquides ?	1. Oui 0. Non 7. N.a. 9. Ne sait pas	|__|
	h)Aliments solides ou semi-solide (purée) ?	1. Oui 0. Non 7. N.a. 9. Ne sait pas	|__|
**34.**	Depuis hier à la même heure que maintenant, combien de fois l'enfant a-t-il mangé des repas solides ou des aliments semi-solides autres que des liquides ?	1. 0 fois **(→Q39)**<br>2. Moins de 3 fois<br>3. 3 fois<br>4. 4 fois<br>5. 5 fois ou plus	|__|
**35.**	Durant les dernières 24 heures, quel type de nourriture avez-vous donné à votre enfant :		
	a) Pain, riz, pâtes ou autres aliments dérivés de céréales	1. Oui 0. Non 9. Ne sait pas	|__|
	b) Carottes, courge ou patates douces à chair jaune ou orange	1. Oui 0. Non 9. Ne sait pas	|__|
	c) Pommes de terre à chair blanche, ignames à chair blanche, manioc ou autres tubercules	1. Oui 0. Non 9. Ne sait pas	|__|
	d) Tous légumes à feuilles vert foncé	1. Oui 0. Non 9. Ne sait pas	|__|
	e) Fruits riches en vitamine A (Mangues mûres, papayes mûres, Melons, Néré etc.)	1. Oui 0. Non 9. Ne sait pas	|__|
	f)- Autres fruits ou légumes	1. Oui 0. Non 9. Ne sait pas	|__|
	g)- Foie, rognon, cœur ou autres abats	1. Oui 0. Non 9. Ne sait pas	|__|
	h)-Viandes telles que bœuf, porc, agneau, chèvre, poulet ou canard	1. Oui 0. Non 9. Ne sait pas	|__|
	i) Œufs	1. Oui 0. Non 9. Ne sait pas	|__|
	j) Poisson frais ou séché, crustacés ou fruits de mer	1. Oui 0. Non 9. Ne sait pas	|__|
	k) Plats ou aliments contenant des haricots, pois, lentilles, noix ou graines	1. Oui 0. Non 9. Ne sait pas	|__|

	l) Fromage, yaourt ou autre produit laitier	1. Oui 0. Non 9. Ne sait pas	|__|
	m) Huile, graisse ou beurre ou tout aliment en contenant	1. Oui 0. Non 9. Ne sait pas	|__|
	n) Tous aliments sucrés tels que chocolats, bonbons, friandises, pâtisseries, gâteaux ou biscuits	1. Oui 0. Non 9. Ne sait pas	|__|
	o) Condiments aromatiques tels que piments, épices, herbes ou poudres de poisson	1. Oui 0. Non 9. Ne sait pas	|__|
	p) Larves, escargots ou insectes		
	q) Aliments préparés avec de l'huile de palme rouge, de la noix de palme rouge ou de la pulpe de noix de palme rouge	1. Oui 0. Non 9. Ne sait pas	|__|
**36.**	Selon vous est ce que le régime alimentaire que vous avez décrit convient à (NOM) ?	1. Oui **(→Q38)**<br>0. Non **(→Q37)**<br>9. Ne sait pas **(→Q37)**	|__|
**37.**	Si NON qu'aimeriez-vous améliorer dans le régime alimentaire de (NOM) ?<br><br>*Plusieurs réponses sont possibles*	1. Augmenter la quantité<br>2. Augmenter le nombre de repas<br>3. Varier les aliments<br>4. Donner un aliment spécifique, préciser lequel________________________<br>5. Autre, préciser :________________________<br>6. Ne sait pas	|__| |__| |__| |__| <br>|__| |__|
**38.**	A combien de mois avez-vous commencé à donner à l'enfant d'autres aliments en plus du lait maternel ?	Nombre de mois<br>99. Ne sait pas	|__|
**39.**	Êtes-vous satisfait de l'état nutritionnel de (NOM) ?	1. Oui **(→Q41)**<br>0. Non **(→Q40)**<br>9. Ne sait pas **(→Q40)**	|__|
**40.**	Si NON comment aimeriez-vous que (NOM) soit ?	1. Plus grand<br>2. Plus gros/corpulent<br>3. Moins grand<br>4. Moins gros/corpulent<br>5. Autre, préciser:___________________<br>6. Ne sait pas	|__| |__| |__| |__| |__| |__|
**41.**	A partir de quels signes pouvez-vous dire qu'un enfant est malnutri ?<br><br>*Plusieurs réponses sont possibles*	1. Enfant amaigri<br>2. Enfant au visage vieux<br>3. Enfant présentant des œdèmes<br>4. Enfant apathique<br>5. Cheveux roux<br>6. Autre, préciser:___________________<br>7. Ne sait pas	|__| |__| |__| |__| |__| |__| |__|
**42.**	Depuis les 6 derniers mois, l'enfant a-t-il reçu des suppléments de vitamines, minéraux ou médicaments ? (donner un exemple pour illustrer)<br><br>PLUSIEURS REPONSES SONT POSSIBLES	1. Oui, la vit A et/ou fer<br>2. Oui, autre à préciser<br>0. Non **(→Q48)**	|__|

**43.**	Si oui, comment les avez-vous obtenus ?	1. Visite de routine du centre de santé<br>2. Pendant les campagnes spéciales<br>3. Chez un voisin ou membre de famille<br>4. Achat à la pharmacie ou au marché<br>9. Autres à préciser	|__|
**44.**	Où avez-vous accouché de l'enfant ?	1. A domicile<br>2. En cours de route<br>3. Hôpital/ centre de santé public<br>4. Clinique/ centre de santé privé<br>9. Autre (à préciser)	|__|
**45.**	Quand l'enfant est né, était-il ?	1. Plus gros que la moyenne<br>2. Moyen<br>3. Plus petit que la moyenne<br>9. Ne sait pas	|__|
**46.**	Dans les 45 jours qui ont suivi la naissance de l'enfant, avez-vous reçu une dose de vitamine A ?	1. Oui<br>0. Non<br>9. Ne sait pas	|__|
**47.**	Quels conseils donneriez-vous à une femme enceinte sur l'alimentation de son bébé qui va bientôt naître ?<br><br>***NE LISEZ PAS LES REPONSES, LAISSEZ LA FEMME REPONDRE ENCERCLEZ TOUT CE QUI S'APPLIQUE***	1. Initie l'allaitement maternel dans l'heure qui suit la naissance	|__|
		2. Donne le colostrum au bébé	|__|
		3. Positionne et garde le nourrisson correctement au sein	|__|
		4. Allaite à la demande	|__|
		5. Allaite régulièrement pendant la journée	|__|
		6. Allaite pendant la nuit	|__|
		7. Offre le second sein après que le nourrisson ait vidé le premier	|__|
		8. Donne seulement du lait maternel; ne donne pas d'eau ou de thé ou autres liquides ou aliments jusqu'à 6 mois	|__|
		9. Continue à allaiter quand tu seras malade	|__|
		10. Augmente la fréquence d'allaitement pendant et après la maladie du nourrisson, incluant la diarrhée	|__|
		11. Recherche de l'aide auprès d'un travailleur de santé formé ou d'un conseiller si elle a des problèmes avec l'allaitement maternel	|__|
		12. Mange suffisamment d'aliments nutritifs toi-même	|__|
		13. Prends des suppléments de vitamines tel que recommandé par l'gent de santé de santé	|__|
		14. AUTRE :________________________	|__|
		15. AUTRE : ______________________	|__|
		16. NE SAIT PAS :	|__|

**48.**	Quels conseils sur l'alimentation donneriez-vous à une mère pour que son enfant de 6 mois continue à bien grandir dans les mois qui viennent ?<br><br>NE LISEZ PAS LES REPONSES, LAISSEZ LA FEMME REPONDRE ET ENCERCLEZ TOUT CE QUI S'APPLIQUE	1.	Donner uniquement le lait maternel jusqu'a 6 mois	|__|
		2.	Commence à donner des aliments additionnels a l'âge de 6 mois	|__|
		3.	Commencer avec des aliments mous ou la bouillie	|__|
		4.	Continuer avec l'allaitement jusqu'a l'âge de deux ans ou au-delà	|__|
		5.	Augmenter la fréquence des repas avec l'âge de l'enfant *(à partir de 6 mois, 1-2 repas et 1-2 collations en plus de l'allaitement)* *à partir de 9 mois, 3-4 repas et 1-2 collations en plus de l'allaitement)*	|__|
		6.	Augmenter le volume des repas avec l'âge de l'enfant	|__|
		7.	Donner des repas contenant assez d'énergie	|__|
		8.	Offrir chaque jour une variété d'aliments, d'au moins 4 groupes d'aliments (n'entourer la réponse que si la femme a cité au moins 4 aliments appartenant à au moins 4 des 7 groupes alimentaires)	|__|
		9.	Pratiquer une bonne hygiène dans la préparation et le stockage des aliments de complément (lavage des mains ; utilisation de l'eau et des ustensiles propres pour préparer les repas; bien couvrir les repas après la cuisson etc.)	|__|
		10.	Continuer a allaiter et a donner des aliments de complément pendant les périodes de maladie	|__|
		11.	Donner a l'enfant des aliments riches en vitamines (vitamine a, fer, etc.…)	|__|
		12.	Donner des suppléments (vitamines, fer)	|__|
		13.	Encourager l'enfant à manger (le stimuler)	|__|
		14.	Autre ________________________	|__|
		15.	Autre ________________________	|__|
		16.	Ne sait pas	|__|

6. Mesures anthropométriques de l'enfant

UN QUESTIONNAIRE PAR ENFANT ELIGIBLE (6-24 MOIS)

<table>
<tr><td>49.</td><td>Puis-je prendre le poids de l'enfant (00.00 kg) ?</td><td>Mesure Enquêteur 1 :</td><td>|__|__|.|__|__|</td></tr>
<tr><td>50.</td><td></td><td>Mesure Enquêteur 2 :</td><td>|__|__|.|__|__|</td></tr>
<tr><td>51.</td><td>Puis-je prendre la taille de l'enfant (000.0 cm) ?</td><td>Type de mesure Enquêteur 1 :
1 Debout
2 Couché</td><td>|__|</td></tr>
<tr><td>52.</td><td></td><td>Mesure Enquêteur 1 :</td><td>|__|__|.|__|</td></tr>
<tr><td>53.</td><td></td><td>Type de mesure Enquêteur 2 :
1 Debout
2 Couché</td><td>|__|</td></tr>
<tr><td>54.</td><td></td><td>Mesure Enquêteur 2 :</td><td>|__|__|.|__|</td></tr>
<tr><td>55.</td><td>Puis-je mesurer le périmètre brachial de l'enfant (00.0 cm) ?</td><td>Mesure Enquêteur 1 :</td><td>|__|__|.|__|</td></tr>
<tr><td>56.</td><td></td><td>Mesure Enquêteur 2 :</td><td>|__|__|.|__|</td></tr>
<tr><td>57.</td><td>L'enfant a-t'il des œdèmes aux pieds ?</td><td>1. Oui 0. Non</td><td>|__|</td></tr>
<tr><td>58.</td><td>Puis-je mesurer le périmètre brachial de la mère de l'enfant (00.0 cm) ?</td><td>Mesure Enquêteur 1 :</td><td>|__|__|.|__|</td></tr>
<tr><td>59.</td><td></td><td>Mesure Enquêteur 2 :</td><td>|__|__|.|__|</td></tr>
</table>

Etude d'impact du FBP Nutrition au niveau de la communauté au Burundi

Demandes de consentement éclairé

Note : ce texte sera traduit en Kirundi.

Bonjour. Je m'appelle ______________________________. Je travaille pour l'Institut de Statistiques et d'Etudes Economiques du Burundi, ISTEEBU. Avec le Ministère chargé de la Santé, nous effectuons une enquête pour sur la nutrition des enfants de moins de deux ans au Burundi. Les informations que nous collectons aideront le gouvernement à améliorer les services de prévention et de prise en charge de la malnutrition.

Votre ménage a été sélectionné pour participer à cette enquête. Nous souhaiterions vous poser quelques questions sur votre ménage et sur la nutrition de votre enfant de moins de 2 ans, ainsi que prendre les mesures anthropométriques de votre enfant. Cela permettra notamment d'établir si le statut nutritionnel de votre enfant est bon ou s'il nécessite de consulter un centre de santé. Les questions prennent habituellement entre 30 et 50 minutes.

Vous êtes libre d'accepter ou de refuser de participer à l'étude. De plus, vous être libre de modifier ou d'ajuster vos réponses à tout moment. Votre refus ou acceptation de participer à cette interview n'aura aucun effet négatif sur vous ou sur votre ménage. Aussi, si vous acceptez de participer, vous êtes libre de refuser certaines questions ou d'arrêter l'entretien à tout moment. Enfin, si vous acceptez de prendre part à cet exercice, la confidentialité de vos propos et des mesures anthropométriques de votre enfant sontt garantis. Vos données personnelles n'apparaîtront sur aucun document. Elles seront remplacés par des codes dont seul le responsable de l'étude a la clé. Toutes les informations que vous nous donnerez sont strictement confidentielles et elles ne seront transmises à personne d'autres que les membres de l'équipe d'enquête. Si vous voulez, vous pouvez avoir accès à vos données récoltées. Dans ce cas, veuille bien avoir l'amabilité d'informer l'enquêteur.

Si vous souhaitez plus d'informations sur l'enquête, vous pouvez contacter Mr Noé Nduwabike à l'ISTEEBU.

DONNEZ LA CARTE AVEC LES INFORMATIONS POUR CONTACTER CES PERSONNES

Acceptez-vous de répondre à mes questions ?...|__|

1. Je marque mon accord pour participer à l'enquête
2. Je suis désolé de ne pas pouvoir participer à l'enquête → *ARRET DE L'INTERVIEW*

Je déclare être âgé d'au moins 18 ans et avoir reçu les informations nécessaires pour cette étude

SIGNATURE OU EMPREINTES DIGITALES: ....

SIGNATURE DE L'ENQUÊTEUR/ENQUÊTRICE : ....

DATE:.. |__|__|/|__|__|/|__|__|

L'ENQUÊTÉ ACCEPTE DE RÉPONDRE ?.. |__|
(1 OUI – 2 NON → FIN)
