# Supplementary material for: Impact of the extension of a performance-based financing scheme to nutrition services in Burundi on malnutrition prevention and management among children below five: A cluster-randomized control trial
Source: PLoS One. 2020 Sep 18;15(9):e0239036. doi: 10.1371/journal.pone.0239036 (PMC7500612; doi:10.1371/journal.pone.0239036)
Supplement: S4 File — Source: Authors. (PDF) [file pone.0239036.s006.pdf]

ANTWERP UNIVERSITY HOSPITAL
UNIVERSITY OF ANTWERP
ETHICS COMMITTEE

Request for advice addressed to the Ethics Committee concerning a proposal for a clinical trial.

**EC NO**	**EUDRACT NO**	**UNIQUE BELG. NO**
……/………/……	……….-………………-……….	………………………………

TITLE

Etude d'impact du FBP Nutrition au niveau de la communauté au Burundi ....

INFORMATION CONCERNING THE STUDY GROUP

Principal investigator/ Coordinating investigator in our centre: **Bruno Meessen**

Participating members: **Dominique Roberfroid, Catherine Korachais, Patrick Kolsteren**

Correspondence address: **ITM, Nationalestraat 155, 2000 Antwerp**...

Where will the study be performed?
Burundi..

Is this a multicentre experiment? **- NO** -
If so, which are the other collaborating study groups: ....

....

Has the Ethics Committee been selected by you in order to act as the Ethics Committee providing Single Opinion (ECPSO)
- **NO –**
The protocol has been submitted and approved by the EC of Burundi
(If so, please add a list of the Belgian committees)
(If not, please add name and e-mail address of the ECPSO)

Is this a commercial trial?– **NO**

Who is the initiator of the study? *(delete where not applicable).*

- Study group itself: the government of Burundi financed by the World Bank
- Pharmaceutical or other Company
  Name sponsor + address + contact person (+ phone no. + e-mail address if available) ....
- Scientific association
  Name: ....

ATTACHMENTS NECESSARY FOR THE REVIEW OF THE STUDY

	Present
- Information sheet of our Ethics Committee	◉
- Protocol of the trial	◉
- Information sheet for the patient and/or family	◉
- Informed Consent Form for patients	◉
- Pharmacological & Toxicological file (only for research with/ on drugs)	○
- All diaries and questionnaires that are provided to the study participants	◉
- Arrangements for recruitment of subjects	◉
- Insurance document	○
- Billing schedule	○
- Contract between the sponsor of the study and the principal investigator	◉
- CV of the participating physician(s)	◉
- List of the local ethical committees and local investigators (if applicable)	◉

Please submit <u>all documents in duplicate</u>!

BUDGET AND FINANCING OF THE STUDY

How will the study be financed?

- Industry ....
- Government (IWT, FWO, …) ....
- Own resources ....
- Other : **World Bank** ....

Is the fee that you receive as investigator reasonable and in proportion to your performances? (Please mention the amount)

No fees foreseen, the role of ITM is technical support. The field work will be subcontracted to a local study group based on a tendering procedure. ....

How is this amount authorized (salaries, fees, running costs etc)?

....

Are some or all investigations in this study covered by the RIZIV? If so, why?

NA ....

Will the participants receive financial compensation? If so, how much and what exactly does the compensation cover?
NO financial compensation..
....

INFORMATION CONCERNING THE AIM OF THE STUDY

Phase of the study
I – II – III – IV – other : **pre post evaluation with a control group** ....

Study period:
- Start date: **Fall 2014** ....
- End date: **Fall 2016**...

Version of the protocol (mentioning the version number and version date)
Version 3, 4 July 2014...
Version of the investigator's brochure (mentioning the version number and version date)
....
Version of the informed consent (mentioning the version number and version date)
Version 3, 4 July 2014...

Aim of the study
The government of Burundi is implementing a new financing scheme in health centres. The objective is to provide additional financial compensations to health centres on the basis of their performance in nutrition activities: it consists in the introduction of criteria focusing on malnutrition prevention and care activities in the existing performance based financing (PBF) system on (i) acute and chronic malnutrition rates in the community, (ii) better equity in those nutrition outcomes, and (iii) externalities on other health outcomes.

Then the general objective of this study is to assess the effects of this new financing scheme, to document its impact and to study the chains through which it occurred. This study will provide key evidence for countries with an existing PBF scheme and confronted with malnutrition problems on the appropriateness to extend the strategy to nutrition services. If this impact evaluation brings positive results, this may have positive implications for the global fight against malnutrition.

Brief summary of the project
The intervention -introducing PBF in nutrition activities- aims at improving the identification and treatment of malnourished children in the catchment area of health centres.

The study aims at assessing the effects of this intervention in the results of nutrition in the community (catchment area of selected health centres). This is evaluated in terms of acute

and chronic malnutrition prevalence as well as quality of care and nutrition within the households.

This is a pre post evaluation with a control group. In the intervention group, health centres will receive a financial bonus according to the results of the identification of malnourished children and the quality of care they provide. In the control health centres, the basic budget is increased without a performance requirement.

The performance of this financing strategy is judged on the results in terms of malnutrition rates in the community (it is also judged at the health centre level, on the results of the identification and treatment of malnourished children, documented through the reports and clinical files of the health centre, see protocol with similar title sent in June 2014)....................

Scientific rationale of the study

Health care provision in low and middle income countries is lagging behind. Quality of care is often poor and motivation of health workers low. Providing an output based compensation is a way to stimulate health workers to perform better and provide better results. However, so far there is a lack of evidence on performance based financing in low- and middle-income countries. The impact evaluation study presented here will provide with the opportunity to do such an assessment and get scientific insights on the effectiveness of PBF in Burundi, and more generally in low-income countries. ....

If there is a placebo arm, please justify:

In order to provide evidence, a control arm is necessary. In this particular case, control health centres also receive a budget increase but no based on performance. The underlying assumption is that control over output and results will increase input and so also positive results and thus income for the health centre staff. This increase motivation will not take place in the control health centres that will perform as usual. ....

The study is (delete where not applicable):

- epidemiological

Has the study already been performed elsewhere, either in whole or in part?

– NO –

If so, where? ....

If so, what was the outcome? ....

Are there any other competitive studies in your unit during the same period?

– NO –

If so, how will the patients be recruited in these different studies? ....

INFORMATION CONCERNING THE PARTICIPANTS

Number of participants to be recruited:

- in total: **6,480 children aged 6-24 months + their mother or main care-taker + their household head or equivalent**
- in this centre: ....

How will the participants be recruited and what arrangements were made for this recruitment?

The 90 health centres are selected randomly through a list of 190 health centres providing moderate and severe acute malnutrition care in Burundi. We will consider their catchment areas and randomly select six 'sous-collines' (sub-administrative unit) in each catchment area. Within each 'sous-colline', twelve eligible children (i.e. aged 6-24 months) will be selected in the following way: one household will be randomly selected from a list of all households living in the 'sous-colline'. If the household is eligible (i.e. with a child aged 6-24 months), it will be selected for the survey; otherwise, an eligible replacement household will be sought in its neighbourhood and selected for the survey. Afterwards, another eligible household in the neighbourhood will be sought in the neighbourhood, and selected for the survey. This process will be repeated until we get a total of 12 children aged 6-24 months in the 'sous-colline'.

<u>**Inclusion criteria**</u>

- healthy participants? **NA**
- participants suffering from: **NA** ....
- age: **children aged 6-24 months** ....
- gender M/F: **both** ....
- pregnant women or women who might become pregnant during the research? - **NO –**

Is there a control group? **YES**
If so, does it consist of healthy participants?: **NA – same as above** ....
If so, does it consist of participants suffering from: ....
If so, number of participants: ....

Which investigations should the participants undergo?

- Purely clinical evaluation

frequency: ....

- Function tests or dynamic tests

which: ....

frequency: ....

- Radiographic and/or radio-isotopic investigations

which: ....

frequency: ....

- Blood samples

blood volume: ....

frequency: ....

Have all necessary measures been taken to keep the number and volume of the blood samples to a strict minimum? ....

Will the blood sampling also include sample for DNA investigation? ....

Has separate consent been asked for this? ....

For which investigations will this DNA sample be used? ....

How long will the samples be stored? ....

How will the anonymization be done? ....

- Tissue samples

....

- Other

Anthropometric measures (weight, height, mid upper arm circumference...................................

Are there any particular investigations that are not included in the routine clinical practice?

– NO –

<u>Risks</u>

Taking into account the current knowledge of science, do you think that participants in the research:

a) **will incur no risk, complication or adverse event.**

b) may incur potential risks, complications or adverse events.

which? ....

Has this been mentioned in the informed consent? ....

c) are most likely to have a risk, complications or adverse events:

which? ....

Has this been mentioned in the informed consent? ....

Informed Consent

In case a standard treatment is being withheld, has this been mentioned in the informed consent?
N.A. ....

Will the consent be obtained after a clear and objective explanation of the aim and risks of the research?
YES ....

Is the information sheet complete, does it mention possible alternative treatments, have all special investigations been mentioned, as well as all possible risks that may occur, has it been written in lay terms and have all abbreviations been explained?
Information sheet is complete and has been written in lay terms; all investigations have been mentioned. ....

Does the information sheet contain a paragraph in which the participant has been explained that he/she can stop his/her participation in the study at any time, without this having any influence on his/her treatment?
YES ....

Is the patient asked for informed consent to enter his/her data in a database? Does the information sheet mention that the patient has the right to access the database with regard to the information scored about him/her and that he/she can ask to adapt his/her data?
NA. ....

Does the informed consent clearly mention that the doctor/researcher will be remunerated?
NA ....

Is there a Dutch information sheet and informed consent form available?
YES – NO - **NA**

In case no written consent can be obtained because of extreme urgency, will the consent be asked from the participant or its legal representative as soon as possible?
YES – NO - **NA**

In case this is an investigation in children, will the consent been asked from the legal representatives?
YES – NO - NA

SUPERVISION OF PARTICIPANTS

Is there continuous medical supervision during the study?
YES – NO - **NA**

Is there supervision in the hours following the experiment?
YES – NO - **NA**

By whom ? ....

How can this person be contacted ? ....

POINTS OF INTEREST FOR THE INSURANCE

Are all persons involved in the research insured according to the law?

There are no experiments, no insurance is foreseen.

Is the insurance document valid for the entire duration of the study?

. ...

Date: **28 July 2014**

Principal investigator/ Coordinating investigator
(name + signature)

Bruno Meessen

Head of department/service
(name + signature)

Marleen Boelaert
