# Supplementary material for: Impact of the extension of a performance-based financing scheme to nutrition services in Burundi on malnutrition prevention and management among children below five: A cluster-randomized control trial
Source: PLoS One. 2020 Sep 18;15(9):e0239036. doi: 10.1371/journal.pone.0239036 (PMC7500612; doi:10.1371/journal.pone.0239036)
Supplement: S5 File — Source: Authors. (PDF) [file pone.0239036.s007.pdf]

Protocole de recherche

Etude d'impact du FBP Nutrition au niveau des centres de santé au Burundi

Principaux investigateurs

INSP

IMT Blue Square

Banque Mondiale

Ministère de la Santé Publique et de la Lutte contre le SIDA

Version 3, du 30 Juillet 2014

Contenu

Résumé ... 2
Collaborateurs au Burundi et leurs rôles respectifs ... 3
Contexte et justification de l'étude et de l'enquête ... 4
  Contexte ... 4
    Health Results Innovation Trust Fund (HRITF) ... 4
  Justification de l'étude ... 4
    Le FBP et la gratuité des soins au Burundi ... 4
    La nutrition au Burundi ... 4
  Objectifs de l'évaluation d'impact du FBP Nutrition au niveau des CdS ... 5
Méthodologie ... 6
  1. Echantillonnage ... 6
  2. Mesures ... 6
  3. Personnel et organisation pratique ... 9
  4. Contrôle de qualité ... 10
  5. Analyses ... 10
  6. Ethique ... 11
Mise en œuvre de l'enquête ... 12
  1. Pré-enquête ... 12
    Pré-test et adaptation des questionnaires ... 12
    Plan de travail de terrain ... 12
    Achat de matériel ... 13
    Programme de saisie des données (DEP) ... 14
    Recrutement de personnel qualifié sur le terrain ... 14
    Formation des agents enquêteurs ... 15
  2. Travail de collecte sur le terrain ... 16
    Organisation : personnel et durée de l'enquête ... 16
    Gestion des données ... 16
  3. Analyse et publication des résultats de l'enquête ... 17
  4. Propriété des données ... 17
Equipes ... 17
  L'équipe de préparation et collecte des données : l'INSP ... 17
  L'équipe d'évaluation IMT Blue Square ... 18
  Le MSPLS ... 18
  La Banque Mondiale ... 19
Annexe ... 20
  Calendrier / Diagramme de Gantt ... 20
  Budget ... 20
  Questionnaires ... 21
    Module (1) Qualité de la prise en charge thérapeutique des cas de malnutrition aigüe modérée et sévère ... 21
    Module (2) Qualité de la prise en charge nutritionnelle dans les consultations curatives et préventives ... 31
    Module (3) Aspects organisationnels ... 46
    Module (4) Service nutritionnel ... 60
    Module (5) Connaissances du personnel nutrition ... 86
  Demandes de consentement éclairé, par type de questionnaire ... 108

Résumé

L'enquête EI-FBPNut-CdS I ici présentée s'inscrit dans le cadre de l'étude d'impact du volet Nutrition du Financement Basé sur la Performance (FBP) au Burundi. Cette étude d'impact s'inscrit dans le contexte d'une meilleure connaissance des résultats du FBP au Burundi, politique nationale de financement de la santé en vigueur depuis 2010, et profite de la mise en place d'un nouveau volet du FBP, à savoir le volet Nutrition, ajouté pour lutter contre la malnutrition, véritable fléau au Burundi. Cette étude permettra d'évaluer l'efficacité du FBP, mode de financement de la santé le plus utilisé au Burundi, et de comprendre dans quelle mesure ce mode de financement est plus ou moins efficace, particulièrement en matière de lutte contre la malnutrition.

Dans le cadre de cette étude, il est prévu d'effectuer des collectes de données primaires auprès des ménages ainsi qu'auprès des centres de santé nutritionnels (c-à-d disposant de services de supplémentation nutritionnelle et de thérapeutique ambulatoire). Notamment, une série de trois enquêtes auprès des centres de santé nutritionnels est prévue. Celle présentée ici est la première de la série et correspond à l'enquête de base (baseline) ; elle permet ainsi de statuer sur la performance des centres de santé offrant des services nutritionnels en matière de lutte contre la malnutrition avant le démarrage du volet Nutrition dans le FBP.

Pour obtenir des informations statistiquement fiables, un échantillon national de 90 centres de santé nutritionnels sera tiré de manière aléatoire à partir de la liste des 193 centres de santé nutritionnels disposant des deux types de services nutritionnels : le service de supplémentation nutritionnel (SSN) pour la malnutrition aigüe modérée, et le service de thérapeutique ambulatoire (STA) pour la malnutrition aigüe sévère sans complications. Les résultats de ces deux services seront retranscrits à partir des registres et fiches cliniques individuelles. Tous les responsables des centres de santé (CdS), ainsi que le personnel le plus impliqué dans les activités de prévention et de prise en charge de la malnutrition seront interviewés ; ceux-ci seront également observés à travers des séances d'observations. Aussi, pour chaque CdS, dix accompagnants d'enfants seront sélectionnés à la sortie et interviewés sur leur satisfaction des services.

Ainsi, la collecte de données sera faite à partir de plusieurs modules : (1) un module 'fiches cliniques' sur les réalisations des activités de prévention et de prise en charge de la malnutrition (Questionnaires STA et SSN), (2) un module sur la qualité de prise en charge nutritionnelle dans les consultations curatives et préventives (Questionnaires C, P et S), (3) un module sur les aspects organisationnels du CdS adressé au responsable du CdS (Questionnaire G), (4) un module sur l'organisation des services de nutrition (Questionnaire N), et (5) un module sur les connaissances du personnel affecté à la malnutrition (Questionnaire V).

La plupart des données seront collectées électroniquement, à partir de smartphones Android et l'application Open Data Kit. Ainsi, la cohérence de ces données entrées sera vérifiée automatiquement, les données pourront être transférées régulièrement à Bujumbura, il n'y aura pas de travail de saisie/double-saisie, et la base de données pourra être consolidée et analysable dès la fin de la collecte de données sur le terrain. En revanche, pour certains questionnaires (C, P, vignettes 1-2-3 de V, et grille du questionnaire G), le questionnaire papier sera utilisé pour plus de

praticité. Ces données seront envoyées à Bujumbura une fois par semaine, pour que le travail de saisie puisse se faire. Celui-ci se fera sous CS Pro.

Les données seront analysées par l'équipe mettant en œuvre l'évaluation d'impact, à savoir l'équipe de l'IMT Blue Square.

La réussite de l'étude d'impact du FBP Nutrition sera assurée à travers le suivi et le soutien d'un Comité de pilotage et d'un Comité technique mis en place au niveau du Ministère de la Santé Publique et de la Lutte contre le SIDA (MSPLS). La coordination technique de de l'enquête auprès des centres de santé est confiée à l'Institut National de Santé Publique (INSP), avec l'appui technique de l'équipe de l'IMT Blue Square. Le processus de l'enquête EI-FBPNut-CdS I est prévu sur une période de 6 mois et coûtera 70,000 dollars US.

Collaborateurs au Burundi et leurs rôles respectifs

L'INSP rapportera directement au Task Team leader de la Banque Mondiale au Burundi et à l'équipe d'évaluation IMT Blue Square durant les étapes de préparation, de mise en œuvre de la collecte de données et de la livraison des données.

L'INSP sera responsable de l'exécution technique de l'enquête EI-FBPNut-CdS I, en particulier la préparation et la réalisation du travail sur le terrain, le traitement des données collectées et la rédaction des rapports d'enquête. L'INSP fournira les locaux nécessaires devant servir de bureau central pour le personnel de l'enquête. L'INSP sera responsable de la gestion du budget de l'enquête EI-FBPNut-CdS I. Il assurera aussi d'autres tâches administratives. Le personnel de l'INSP sera responsable de la supervision des opérations techniques quotidiennes, y compris le recrutement et la formation du personnel de terrain et de traitement et la supervision des activités de bureau et de terrain.

L'équipe d'évaluation IMT Blue Square assurera un suivi technique continu de la préparation et de l'exécution de l'enquête. L'équipe sera aussi responsable de l'analyse des données et de la diffusion des résultats au Burundi. A ce titre des ateliers sont prévus à l'automne 2014.

Le Ministère de la Santé Publique collabore à cette étude, car ses résultats lui seront utiles pour affiner sa politique de financement de la santé à travers le FBP et bien définir le volet nutrition du FBP. Des comités de pilotage et technique de l'étude seront instaurés.

Enfin, la Banque Mondiale assure le financement de l'enquête et de l'étude d'impact (y compris l'analyse de ces données).

En outre, le Comité National d'Éthique examinera le protocole proposé et devra l'approuver avant la réalisation de l'enquête.

Contexte et justification de l’étude et de l’enquête

Contexte

Health Results Innovation Trust Fund (HRITF)
Le HRITF vise à concevoir et mettre en œuvre des programmes pilotes de FBP durables qui améliorent efficacement les résultats de santé des mères et des enfants (c'est-à-dire les OMD 4 et 5). L'hypothèse est que le FBP peut être utilisé pour améliorer l’accès aux soins de santé appropriés ainsi que la qualité des soins de santé et encourager les individus à adopter des bons comportements de santé.

Le HRITF finance les interventions pilotes de FBP – que ce soit du côté de l’offre (régimes de paiement fournisseur, sous-traitance) ou du côté de la demande (transferts conditionnels en espèces). Un élément central du HRITF est de mieux comprendre et documenter l'ampleur avec laquelle les politiques FBP sont efficaces, réalisables, et dans quelles circonstances. Par conséquent, une évaluation rigoureuse de tous les programmes de FBP de la santé est indispensable pour générer de nouvelles connaissances qui peuvent informer les gouvernements et les partenaires pour concevoir et utiliser efficacement les mécanismes de FBP.

C’est dans ce contexte que l’évaluation du FBP Nutrition au Burundi est réalisée.

Justification de l’étude

Le FBP et la gratuité des soins au Burundi
En 2006, face à des besoins en santé de la population croissants, le gouvernement a mis en place l’exemption des paiements directs pour les enfants de moins de 5 ans et les accouchements. Cette décision s’est heurtée à de nombreux problèmes, notamment de financement. Parallèlement, le Burundi expérimentait dans trois provinces une nouvelle stratégie de financement de la santé, le financement basé sur la performance (FBP), dont l’objectif était d’améliorer le système de santé. Après ces quelques expériences pilotes, le FBP s’est étendu à l’échelle du pays en avril 2010. Une décision importante prise par le gouvernement fut celle de fusionner le FBP et la gratuité des soins.

En 2012, avec le soutien de la Banque Mondiale, le gouvernement Burundais a renforcé les indicateurs existants concernant le planning familial en doublant les tarifs FBP. Il compte également introduire des indicateurs liés à la nutrition dans le FBP existant, ce qui est une opportunité à la fois pour améliorer le statut nutritionnel des enfants et pour réaliser une analyse rigoureuse des forces et des faiblesses du FBP.

La nutrition au Burundi
Au Burundi, la malnutrition est une barrière évidente à la réalisation des OMD liés à la santé. En effet, 58% des enfants de moins de 5 ans souffrent de malnutrition aigüe chronique. Suivant la tendance internationale, le Ministère de la santé du Burundi a développé en 2010 un protocole proposant un plan de traitement et de suivi de la malnutrition pour les enfants. Aujourd’hui, seulement un tiers des centres de santé et la moitié des hôpitaux offrent des services de prise en charge de la malnutrition.

L'intégration des services de nutrition dans le FBP est un défi important dans la mesure où actuellement, la mise en œuvre des services de nutrition est fortement dépendante d'organisations externes comme l'UNICEF ou le World Food Program. De plus, on constate que les services de nutrition sont négligés de par un manque de connaissances et de savoir-faire du personnel de santé et un manque de supervision des activités de nutrition. Aussi, l'introduction du FBP devrait permettre de faire reculer ces obstacles.

Objectifs de l'évaluation d'impact du FBP Nutrition au niveau des CdS

L'évaluation d'impact du FBP Nutrition au niveau des centres de santé (CdS) fournissant à la fois un service de supplémentation nutritionnelle (SSN) et un service thérapeutique ambulatoire (STA) s'articule autour des questions de recherche suivantes :

- Le FBP Nutrition résulte-t-il en une amélioration de la prise en charge préventive et curative des problèmes nutritionnels dans les CdS ?
- Par quels mécanismes le FBP Nutrition produit-il (ou pas) ce résultat ?

Il est prévu de répondre à la première question en évaluant l'évolution d'indicateurs de résultat, tels que les taux de guérison de malnutrition aigüe sévère (MAS) et modérée (MAM), la durée des traitements des cas de MAS et MAM, les taux de rechute, les taux de décès ou le pourcentage des cas suivis régulièrement qui sont référés au CdS. Pour la deuxième question, il est prévu d'évaluer l'évolution de l'organisation et la qualité du centre et des services nutritionnels, les connaissances, attitudes et savoir-faire du personnel de santé vis-à-vis de la malnutrition, et la satisfaction des utilisateurs des services[1].

L'évaluation sera basée sur une étude comparative randomisée, c'est-à-dire que le FBP Nutrition sera introduit dans un certain nombre de CdS (groupe intervention) choisis aléatoirement et les résultats de l'intervention comparés à un groupe de CdS similaires sans FBP Nutrition (groupe contrôle où l'organisation actuelle des soins est maintenue). Pour faciliter l'acceptabilité de l'étude par tous les CdS, le groupe d'intervention et le groupe de contrôle recevront les mêmes montants (sous forme de subsides dépendant de la performance pour les premiers, et sous forme de dotation fixée comme correspondant à la moyenne des subsides pour les seconds).

Les données d'enquêtes auprès des CdS inclus seront collectées avant que l'intervention FBP Nutrition démarre (baseline, T0), courant avril 2014 ; puis un an après que l'intervention ait été mise en œuvre (T1), autour de juillet 2015 ; puis encore un an après (T2), autour de juillet 2016. L'enquête présentée dans ce document porte sur les données T0.

---

[1] Une des lacunes observées dans les services nutritionnels est le manque de connaissances et de savoir-faire du personnel. Pour faire face à cet obstacle, des formations, payantes, seront proposées aux centres de santé. Lors d'un atelier, fin mai-début juin 2014, l'équipe d'évaluation du FBP Nutrition attribuera des scores de performance, en termes d'organisation et de de qualité des services nutritionnels, à chaque centre de santé. Ainsi chaque responsable de centre de santé pourra prendre des décisions de formation en connaissance de cause. C'est un objectif secondaire de ces enquêtes.

Méthodologie

1. Echantillonnage

Au total, 193 CdS sont éligibles au Burundi, c'est-à-dire qu'ils assurent les services SSN et STA. Ils seront listés et numérotés, et 90 d'entre eux seront choisis pour faire partie de l'étude par tirage aléatoire simple. Sur base des résultats de l'enquête de base (T0), les CdS similaires sur des paramètres essentiels définis à priori (taille de la population de couverture, qualité de la prise en charge actuelle des cas de malnutrition, volume des cas de malnutrition traités, volume du personnel) seront appariés en 45 paires. Au sein des 45 paires, l'allocation d'un des centres au groupe intervention (FBP Nutrition) se fera par randomisation simple, l'autre CdS de la paire étant automatiquement attribué au groupe contrôle (matching ou randomisation bloquée). La séquence de randomisation sera générée par ordinateur dans le programme STATA.

Le taux de guérison des enfants malnutris pris en charge par le CdS est considéré comme le paramètre d'impact principal de l'intervention au niveau des CdS. Pour permettre de détecter une augmentation du taux de guérison de 80% à 90% avec un pouvoir statistique de 80%, un risque d'erreur alpha de 5%, et une corrélation intra-paire estimée à 0.15[2], 12 enfants ayant souffert de malnutrition aigüe sévère et 12 de malnutrition aigüe modérée seront inclus par CdS[3].

Dans chaque CdS, un échantillon aléatoire de 10 consultations préventives et curatives sera observée de façon structurée (voir questionnaires C et P). Les accompagnants des enfants dont la consultation aura été observée seront également interviewés à la sortie de la consultation pour mesurer leur degré de satisfaction (cf. questionnaire S). Le nombre de 10 a été fixé a priori.

2. Mesures

Les principales dimensions à mesurer sont résumées dans le tableau 1 ci-dessous ; les questionnaires complets sont disponibles en annexe.

L'information concernant les 12 enfants traités pour malnutrition aigüe sévère sera obtenue rétrospectivement à partir des dossiers individuels du STA. Les fiches individuelles de 12 enfants sortis du STA seront sélectionnées aléatoirement parmi les fiches des enfants traités dans les 3 mois avant l'enquête, et l'information utile sera extraite dans le formulaire STA. Il en sera de même pour l'information concernant les 12 enfants traités pour malnutrition aigüe modérée, à travers des dossiers individuels du SSN. La randomisation se fera à partir d'une liste de nombres générés par ordinateur (STATA) et une liste différente par centre sera utilisée.

A l'arrivée de l'équipe d'enquête, les questionnaires sur l'organisation générale (G) et des services de nutrition (N) seront complétés. Ceci permettra entre autres d'identifier les agents de santé les

[2] Reinhard Kaiser, Bradley A. Woodruff, Oleg Bilukha, Paul B. Spiegel, Peter Salama 2006. Using Design Effects From Previous Cluster Surveys to Guide Sample Size Calculation in Emergency Settings. Disasters 30(2):199–211.

[3] Hayes,R.J.; Bennett,S., Simple sample size calculation for cluster-randomized trials, Int J Epidem, 1999, 28(2): 319-326

plus impliqués dans les services de nutrition (maximum trois). Ce sont les consultations de ces trois agents qui seront évalués à travers des observations directes (C et P).

En ce qui concerne les questionnaires relatifs à la qualité des consultations, 10 couples mère-enfant consultant les trois agents de santé nutrition identifiés seront choisis au hasard (tirage au sort à partir de tickets dans un chapeau) au moment où ils entrent en consultation, et invités à participer à l'étude ; leur consentement éclairé sera recueilli. Au moment de la consultation, ces personnes seront accompagnées par l'enquêteur chargé de l'observation de la consultation (Questionnaires C et P). A la fin de leur consultation, ces personnes seront dirigées vers un deuxième enquêteur qui administrera le questionnaire de satisfaction et reprendra les mesures anthropométriques de l'enfant (Questionnaire S).

Le questionnaire sur les connaissances du personnel ou vignettes seront administrés en fin de journée aux trois mêmes agents de santé identifiés (Questionnaire V) afin de minimiser le dérangement du travail du CdS. Ces vignettes sont des mises en situation clinique. Un motif de consultation est proposé, et l'agent de santé peut poser toutes les questions nécessaires pour arriver à un diagnostic et proposer une prise en charge. Trois vignettes seront administrées à chacun des trois agents de santé sélectionnés.

Tableau 1 : Dimensions, sources de données et outils

**Dimensions**	**Sources de données**	**Outils**	**N par CdS**
1. Qualité de la prise en charge thérapeutique des cas de malnutrition aigüe modérée et sévère	Registre STA<br>Registre SSN	Formulaire STA<br>Formulaire SSN	12<br>12
2. Qualité de la prise en charge nutritionnelle dans les consultations curatives et préventives	Observation directe<br>-des consultations curatives<br>-des consultations préventives<br><br>Satisfaction des clients	 Questionnaire C<br>Questionnaire P<br><br>Questionnaire S	 10 C et P<br>*au total*<br><br>10 S
3. Organisation générale du CdS	Interview du gestionnaire CdS	Questionnaire G	1
4. Organisation des services de nutrition	Interview du responsable nutrition	Questionnaire N	1
5. Connaissances du personnel nutrition	Vignettes (simulation de cas)	Questionnaire V	1 Petit questionnaire d'informations sur l'agent<br>+ 3 vignettes par agent de santé<br>*(max. 3 agents de santé)*

Ces questionnaires ont été préparés au préalable par l'équipe d'évaluation d'impact de l'IMT Blue Square, en français. Ils seront testés et adaptés dans un premier temps par l'équipe IMT Blue Square. Puis, en consultation avec cette équipe, l'INSP adaptera les modules d'enquête au contexte du pays, y compris la formulation des questions et les codes de réponse afin d'être adaptés au contexte de l'étude. Une fois que l'INSP aura adapté le questionnaire français afin de tenir compte des besoins spécifiques du projet, l'équipe d'évaluation de l'IMT Blue Square traduira l'ensemble du questionnaire vers le kirundi ; l'INSP l'adaptera et le pré-testera, puis adaptera à nouveau le

questionnaire en fonction des résultats du pré-test, afin de saisir avec précision l'information prévue sur les populations étudiées. La dernière version des questionnaires sera à nouveau traduite vers le français par un traducteur indépendant et formaté dans un format identique à celui utilisé pour la mise en œuvre effective.

Un cahier de procédures précisant dans le détail la collecte des données sur le terrain sera rédigé par IMT Blue Square. Ce manuel servira aussi pour la formation des enquêteurs.

3. Personnel et organisation pratique

Les enquêteurs travailleront en équipes de trois (8 équipes). Chaque équipe visitera un centre de santé par jour. Les équipes devront au préalable (1) décrire l'étude et ses objectifs et expliquer que le CdS a été sélectionné pour l'étude, (2) identifier si les services de vaccination sont réalisés certains jours de la semaine afin de s'assurer que, le jour de la visite, des consultations préventives pourront être observées, (3) prendre RDV avec les gestionnaires des CdS et les responsables des services nutritionnels, et (4) leur demander de préparer les différents registres nécessaires à la bonne réalisation de l'enquête. Deux coordonnateurs de terrain seront responsables pour le coaching et la supervision de 4 équipes chacun, et pour les aspects organisationnels avec la communauté et le ministère de la santé. Dix (10) véhicules pouvant contenir quatre à cinq personnes (y compris le chauffeur) seront nécessaires aux équipes de collecte durant toute la période de collecte.

Avec 8 équipes, la collecte des données des 90 centres de santé sélectionnés prendra 12 jours ouvrés, soit trois semaines. Dans chaque équipe de trois enquêteurs :

- Un enquêteur sera responsable :
    - des questions sur les aspects organisationnels au responsable du CdS (Questionnaire G) en début de journée,
    - des entretiens à la sortie des accompagnants d'enfants (Questionnaire S) durant la journée,
    - en binôme avec le deuxième enquêteur, des questionnaires et vignettes attribuées à trois agents de santé nutritionnels (Questionnaire V), à la fin de la journée.

- Le deuxième enquêteur (médecin ou infirmier) sera responsable :
    - des questions sur les aspects organisationnels des services nutritionnels au responsable de service (Questionnaire N) en début de journée,
    - de l'observation des activités de suivi de la croissance et de prise en charge de la malnutrition (Questionnaires C et P) durant la journée.
    - en binôme avec le premier enquêteur, des questionnaires et vignettes attribuées à trois agents de santé nutritionnels (Questionnaire V), à la fin de la journée.

- Le troisième enquêteur sera responsable pour :
    - la retranscription des fiches cliniques du STA (Formulaire STA) et des fiches cliniques du SSN (Formulaire SSN) durant la journée ;
    - il jouera également le rôle de contrôleur dans l'équipe

A la fin de la journée, les données saisies sous Android seront transférées vers la plateforme ODK, et ainsi, le coordonnateur superviseur responsable de l'équipe, ainsi que les superviseurs basés à Bujumbura pourront les vérifier. Les données saisies sur le papier seront envoyées à Bujumbura à la fin de chaque semaine.

Chaque semaine, une réunion sera organisée par le coordonnateur-superviseur d'équipes terrain. Cela devrait donner à l'équipe l'occasion de discuter des problèmes liés à la supervision, à l'organisation du travail de terrain, des problèmes de saisie des données, etc., utiles pour le rapport

intérimaire. En effet, des rapports d'étape hebdomadaires du nombre et des identifiants de CdS réalisés avec succès seront réalisés chaque fin de semaine par le coordonnateur-superviseur.

Il devra y avoir une communication étroite entre le bureau central de l'INSP et le personnel de terrain pendant le travail de collecte. Les détails concernant la supervision et les communications seront discutés durant la formation et figureront dans les manuels de l'enquêteur.

Tableau 2. Organisation des enquêteurs sur le terrain

<table>
<tr><th>Enquêteurs</th><th>#1</th><th>#2 (Infirmier)</th><th>#3</th></tr>
<tr><td>Avant l'ouverture du centre de santé au public</td><td>Questionnaire sur l'organisation générale (G)
Responsable du CdS</td><td>Questionnaire sur l'organisation des services de nutrition (N)
Responsable des services nutrition</td><td rowspan="3">Qualité de la prise en charge thérapeutique des cas de malnutrition aigüe sévère (STA)
et des cas de malnutrition aigüe sévère (SSN)

Retranscription fiches STA
Retranscription fiches SSN</td></tr>
<tr><td>Durant les heures d'ouverture</td><td>Entretiens à la sortie (S)
Accompagnants d'enfants</td><td>Observation sur la qualité de prise en charge nutritionnelle dans les consultations curatives (C) et préventives (P)
Agents de santé nutritionnels</td></tr>
<tr><td>A la fin de la journée</td><td colspan="2">Vignettes sur connaissances du personnel de nutrition (V)
Agents de santé nutritionnels</td></tr>
</table>

4. Contrôle de qualité

Le contrôle de qualité sera assuré à travers la supervision et le suivi des équipes pendant les travaux sur le terrain. Les coordonnateurs-superviseurs d'équipes seront responsables de la qualité du travail de leurs équipes : ils tiendront des réunions régulières avec eux de façon à renforcer leur formation et à corriger les erreurs éventuelles commises au cours de la collecte. De plus, les coordonnateurs-superviseurs procéderont à des Lot Quality Assessment Sampling (LQAS) sur les données retranscrites à partir des registres SSN et STA, ainsi que sur les exit interviews (notamment l'anthropométrie) afin de détecter rapidement des problèmes de qualité dans la collecte des données et de les corriger. Ils observeront également à cette fin un échantillon de consultations préventives et curatives, et de séances d'administration des vignettes. Ils seront présents sur le terrain durant toute la période de l'enquête.

Par ailleurs, l'équipe d'évaluation de l'IMT Blue Square engagera également deux superviseurs qui assureront un contrôle de qualité complémentaire. Aussi, deux personnes de l'encadrement technique, ainsi que la Direction du projet s'occuperont de la supervision au niveau de l'INSP.

5. Analyses

L'analyse des données d'enquête de baseline sera réalisée sous STATA. Elle permettra dans un premier temps de : (1) donner un vue d'ensemble de l'organisation et la qualité des services nutritionnels dans les centres de santé au Burundi, et (2) valider le design de l'étude, i.e. s'assurer que les centres de santé du groupe d'intervention sont comparables avec ceux du groupe de contrôle.

Les données permettront aussi de renseigner chacun des centres de santé visités sur leurs performances en termes de gestion ou de services nutritionnels : des scores seront calculés et délivrés lors d'un atelier où tous les centres de santé enquêtés seront représentés, et où des formations pour s'améliorer seront proposées.

Dans un second temps, les données d'enquête de baseline seront couplées et analysées avec celles des enquêtes de suivi, un an puis deux ans plus tard, pour estimer l'impact de l'intervention 'FBP Nutrition' en termes de résultats (i.e. taux de guérison de la malnutrition aigüe sévère ou modérée) mais aussi en termes de processus (i.e. connaissances et savoir-faire des agents de santé, satisfaction des usagers, etc.). Il s'agira d'estimations de différences-en-différences, comparant à la fois le 'avant-après' et le 'avec-sans' (intervention ou contrôle).

Se référer à la note conceptuelle de la recherche pour plus de détails sur les méthodes d'analyse.

6. Ethique

Cette recherche ne soulève pas d'enjeux éthiques majeurs. L'étude d'impact n'implique pas de nouveaux prestataires de soins - ce sont les CdS déjà en place. Les prestations qui seront évaluées sont des fonctions déjà établies : ce sont les activités relatives à la prévention et la prise en charge de la malnutrition infantile. Les équipes nutritionnelles des CdS qui feront partie de l'étude sont déjà censées mettre en œuvre toutes les activités que nous allons observer. L'étude d'impact ne porte pas sur une nouvelle activité (ex : un nouveau traitement, un nouveau protocole de prise en charge), mais sur le mode de financement de prestataires. À cet égard, il est également important de noter que les groupes de contrôle et d'intervention recevront des ressources équivalentes - il n'y a pas de distorsion ou d'iniquité à ce niveau. Notre étude d'impact compare deux stratégies de motivation du personnel des CdS, qui toutes les deux ont été prouvées comme efficaces dans des situations analogues. Il s'agit de comparer un modèle supposant que l'individu est surtout sensible aux incitants économiques (groupe d'intervention) contre un modèle supposant que l'individu est mobilisé par une motivation intrinsèque.

Selon nous, le principal enjeu éthique dans cette étude réside dans le respect des parties qui seront sollicitées pour nous confier une information : le nourrisson, la personne accompagnant l'enfant et le personnel de santé.

En ce qui concerne le nourrisson, nous veillerons à ce que le processus d'enquête n'affecte pas le respect et l'attention qui doivent être accordées par le personnel de la santé. Si nous devons interagir avec l'enfant (par exemple pour vérifier ses données anthropométriques) nous veillerons à confier cette tâche à un personnel burundais qualifié. En ce qui concerne la personne accompagnante et le personnel de santé, notre respect pour leurs droits passera par l'obtention d'un consentement éclairé. Enfin, si les mesures anthropométriques de l'enfant (prises lors de l'entretien à la sortie) détectent un cas de malnutrition, il sera conseillé à l'accompagnant de se rendre dans les services appropriés.

Les objectifs de l'enquête seront décrits par les enquêteurs aux personnes éligibles pour l'enquête. Ces dernières pourront librement accepter ou refuser de répondre aux questions. Pour celles qui

acceptent, un consentement libre et éclairé sera signé (cf. Annexe). Elles auront le droit de ne pas répondre aux questions sans devoir donner une motivation à ce refus.

L'autre principal enjeu éthique de cette recherche résidera au niveau de la manipulation des données. Il est escompté de saisir un maximum de données sur des tablettes numériques. En phase de pré-analyse, ces données collectées seront anonymisées et la confidentialité de tout un chacun sera sauvegardée.

Enfin, la recherche s'inscrit dans une volonté de contribuer de façon pertinente et rapide au processus politique. Il est important de noter que cette étude d'impact s'inscrit dans un financement structurel par la Banque Mondiale du système de santé burundais. En cas de résultats confirmant l'efficacité de l'intervention, celle-ci sera mise à l'échelle sur l'ensemble du pays. Une grande partie des données collectées sous ce protocole seront analysées par un chercheur burundais (Dr Manassé Nimpagaritse) pour identifier les mécanismes pouvant expliquer pourquoi certains centres de santé ont mieux réagi que d'autres à l'intervention. Cette information sera identifiée comme 'bonnes pratiques' et partagée lors du passage à l'échelle.

Mise en œuvre de l’enquête

1. Pré-enquête

Pré-test et adaptation des questionnaires
L’équipe d’évaluation de l’IMT Blue Square réalisera un pré-test de l’enquête EI-FBPNut-CdS I dès que les questionnaires auront été finalisés et traduits vers le kirundi. L’objectif du pré-test est de détecter d’éventuels problèmes dans les questionnaires, ainsi que d’évaluer le temps nécessaire pour conduire les interviews. La même équipe adaptera ensuite à nouveau le questionnaire en fonction des résultats du pré-test. La dernière version des questionnaires sera à nouveau traduite vers le français par un traducteur indépendant et formaté dans un format identique à celui utilisé pour la mise en œuvre effective.

Le test sera réalisé par trois membres burundais de l’équipe de l’IMT Blue Square (un médecin, un spécialiste en santé publique et un statisticien ayant de l’expérience en matière d’enquêtes), sur deux-trois jours auprès de deux ou trois centres de santé éligibles.

Les résultats du pré-test seront utilisés pour modifier les Instruments de l'enquête et les procédures de terrain, si nécessaire. On examinera de manière toute particulière la façon dont se sont déroulés les tests et on modifiera les procédures de terrain en conséquence. Toutes les décisions concernant les modifications après la pré-enquête devront être prises avec toutes les parties prenantes et validées par le comité technique.

Plan de travail de terrain
La liste des 90 CdS à enquêter sera fournie par l’équipe d’évaluation IMT Blue Square, avec une description de la procédure d’échantillonnage. Un plan de travail de terrain sera ensuite écrit pour décrire en détail tous les aspects du travail de terrain qui sera mené par la firme d’enquête, y compris :

- Diagramme de Gantt mis à jour et final
- Composition d'une équipe de terrain
    - Nombre d'agents enquêteurs
    - Nombre de superviseurs de terrain
    - Qualifications, formation de chacun
- Pour chaque membre d'équipe : taches prévues, responsabilités et calendrier des réalisations attendues
- La durée prévue que chaque équipe passera autour d'un CdS
- Transport et logistique d'hébergement
- Protocole afin de confirmer que l'emplacement a été correctement identifié
- Plans de supervision et de vérification pour garantir le respect des protocoles de collecte de données et confirmer la qualité de la collecte et saisie de données, comprenant au moins 10 re-visites auprès d'un échantillon aléatoire de l'échantillon de l'évaluation pour confirmer la validité des données
- Protocoles et procédures pour traiter les données incohérentes
- Protocoles pour la saisie des données sur les smartphones
- Protocoles pour la collecte de données au cas où le smartphone tombe en panne
- Protocoles de transmission de données vers le niveau central

Ce Plan de travail de terrain sera présenté à l'équipe d'évaluation IMT Blue Square pour commentaire et révisé au besoin avant de commencer le travail sur le terrain. L'INSP devra alors mettre en œuvre l'enquête, et adhérer étroitement au plan. Si les conditions de terrain imposaient des changements importants à ces plans, les superviseurs de terrain de la firme d'enquête seraient obligés d'informer l'équipe d'évaluation, sous la forme d'un rapport écrit.

Achat de matériel

Pour cette enquête, des smartphones Android seront nécessaires pour la collecte des données. Il est prévu que chaque enquêteur en dispose d'une. Ainsi il faudra prévoir au minimum 24 tablettes ou smartphones Android pour l'enquête ; avec une marge de risque de 5%, il faudra s'en procurer 26 au total. Les modèles conseillés ont les caractéristiques techniques suivantes :

- Samsung Galaxy Young
    - Numéro du modèle : GT-S6312
    - Version Android : 4.1.2
    - Version de la bande de base : S6312WWAMD1
    - Numéro de version: JZO54K.S6312XXAMI1
- Dual Samsung Galaxy Chat
    - Numéro du modèle : GT-B5330
    - Version Android: 4.1.2
    - Version de la bande de base: B5330XXUBME1
    - Numéro de version: JZO54K.B5330XXUBME1

Il sera également bon de prévoir des stylets pour la saisie sur écran tactile (si les modèle Samsung Galaxy Young est choisi).

Il faudra également un ordinateur portable par chef d'équipe sur le terrain (soit 3 au total si on considère une marge de risque), et deux ordinateurs de bureau au niveau de Bujumbura.

Des outils de mesures anthropométriques seront également nécessaires, à raison d'un set par équipe. Huit (8) sets seront donc nécessaires. Un set comprend :

- Une toise SECA 417 (ou toise UNICEF),
- Une balance SECA 877,
- Et un mètre de mesure SECA Girth ou SECA 212.

L'INSP fera le nécessaire pour obtenir ce matériel avant la formation des agents enquêteurs.

Programme de saisie des données (DEP)

L'équipe d'évaluation IMT Blue Square fournira au préalable le programme de saisie des données sous Open Data Kit, qui sera utilisé sur les smartphones Android des enquêteurs. L'interface utilisateur sera traduite en kirundi, et le programme sera adapté pour refléter les modifications apportées par le questionnaire de base suite au pré-test du questionnaire. Le programme adapté devra être robuste :

- Et la plage de saisie de données et les contrôles de cohérence devront être adaptés à des valeurs appropriées au contexte du Burundi, sur la base de données existantes.
- En effet, le programme de saisie des données devra effectuer des contrôles de cohérence (dans la mesure du possible) ; la violation de ces contrôles devra conduire à un message immédiat et transparent, ainsi qu'une méthode pratique pour corriger les erreurs, et à une documentation de toutes les réponses qui violent la cohérence.
- Le programme devra permettre des réponses ouvertes (texte) et des réponses « autres » en dehors des choix de réponses fournis dans le questionnaire.
- Les noms des variables générées par le programme devront correspondre clairement et logiquement aux étiquettes des questions utilisées dans le questionnaire.

Pour les questionnaires administrés sur papier (C, P, V et grille de G), l'équipe d'évaluation fournira également le programme de saisie des données sous CS Pro. Les mêmes caractéristiques citées ci-dessus sont de mise.

Recrutement de personnel qualifié sur le terrain

L'INSP recrutera le personnel nécessaire pour cette enquête. Cela comprend :

Au niveau de l'équipe centrale :

- 1 gestionnaire de projet et de données, à temps plein
- 1 coordonnateur de bureau à temps partiel

Au niveau de chaque équipe sur le terrain (il y en aura deux) :

- 1 Coordonnateur superviseur (idéalement, médecin)
- 12 (4 x 3) enquêteurs (comprenant idéalement 4 x 1 infirmiers)

Au final vingt-quatre (24) agents de terrain seront recrutés par l'INSP et organisés en deux équipes. Chaque équipe sera composée d'un chef d'équipe coordonnateur superviseur, et de quatre groupes de trois enquêteurs.

Tous les candidats aux postes d'agents de terrain seront sélectionnés sur la base de leur maturité, de leur capacité à communiquer, de leur niveau d'instruction, de leur connaissance de la langue locale et de leur disponibilité à travailler loin de chez eux pour une période de près de quatre semaines. Tout sera fait pour engager du personnel de terrain qualifié dont le profil sera prédéfini par l'équipe technique de l'enquête.

Formation des agents enquêteurs
Tous les candidats suivront une formation d'une semaine, portant sur tous les aspects de l'enquête. Un nombre de candidats supérieur aux besoins seront formés de façon à pouvoir choisir les meilleurs éléments et, éventuellement, à faire des changements au cours des premiers jours de terrain. La formation aura lieu dans un cadre de capacité suffisante pour accueillir les candidats. L'INSP fournira les formateurs et l'équipe d'évaluation de l'IMT Blue Square les assistera.

L'équipe d'évaluation IMT Blue Square fournira le projet de formation (incluant matériel de formation) à l'INSP. L'INSP devra adapter le matériel au contexte local en consultation avec l'équipe d'évaluation. Si nécessaire, le matériel didactique et les manuels pratiques seront traduits en langue locale.

Le programme de formation comprendra : une description détaillée du contenu des questionnaires ; une présentation des techniques d'interview ; et une formation sur l'utilisation des tablettes/smartphones Android et sur la façon de remplir les questionnaires sous Open Data Kit. Chaque enquêteur réalisera au moins six entretiens pendant la durée de formation.

Au moins une journée sera réservée aux chefs d'équipe, aux coordonnateurs et au personnel d'encadrement pour les former sur la façon d'observer les interviews sur le terrain, de vérifier, stocker et transférer vers Bujumbura les questionnaires remplis.

Le programme de formation inclura :

- *Théorique*: théorie du questionnaire et de chaque question afin de bien comprendre l'objectif de chaque question ; les techniques d'entretien et protocoles de terrain doivent également être couverts.
- *Pratique en classe*: exercices individuels et de groupe pour se familiariser avec la pratique de poser des questions et remplir un questionnaire.
- *Essai pilote*: après la théorie et la pratique en classe, les enquêteurs doivent aller sur le terrain pour administrer le questionnaire complet à un petit nombre de CdS (en dehors de l'échantillon de l'étude). Le pré-test ne se concentrera pas sur les ajustements majeurs au questionnaire, mais simulera plutôt l'administration du questionnaire dans des circonstances normales. L'essai-pilote devrait également servir de test pour l'utilisation des smartphones ou tablettes.
- *Évaluation*: A la fin de la formation, les étudiants doivent être évalués selon leur compréhension du questionnaire et leur capacité à enregistrer correctement les données en

utilisant les mêmes scénarios de test comme utilisé dans la pratique en classe. La formation ne devrait se terminer qu'une fois que les équipes de terrain auront fait preuve de maîtrise des tâches désignées.

Après avoir terminé le stage de formation, tout agent de terrain sélectionné devra avoir une connaissance approfondie du rôle à jouer dans la collecte des données afin d'atteindre le maximum d'efficacité dans les travaux sur le terrain.

2. Travail de collecte sur le terrain

Organisation : personnel et durée de l'enquête

Cf. Section « 3. Personnel et organisation pratique » (page 9) et « 4. Contrôle de qualité » (page 10) pour ces points. Il est prévu 12 jours de travail de collecte afin d'obtenir les données sur les 90 centres de santé sélectionnés.

Gestion des données

Chaque fin de journée, les données collectées sous Android seront transférées vers la plateforme internet ODK (cela sera néanmoins sujet au réseau 3G disponible dans la zone où se trouveront les enquêteurs); ainsi l'équipe de supervision basée à Bujumbura pourra y avoir accès. Chaque fin de semaine, les données collectées sur papier seront envoyées vers l'INSP Bujumbura. Ces données seront saisies sous CS Pro. Tout cela servira notamment à la vérification des questionnaires, la codification des variables non pré-codées et in fine la préparation pour la consolidation et l'analyse.

L'ensemble des activités de traitement des données sera supervisé par le gestionnaire de données basé à l'INSP Bujumbura en collaboration avec un coordonnateur de l'équipe d'évaluation IMT Blue Square.

La saisie des données relatives aux questionnaires G[4], N, S, SSN, STA et V[5] se fera sur smartphone Android en utilisant un programme (Open Data Kit) qui contrôle l'étendue des données et la logique des sauts du questionnaire, ainsi que la cohérence interne. Les questionnaires C, P et V, ainsi que la grille des droits décisionnels dans le questionnaire G seront administrés sur papier : leur saisie se fera a posteriori (après chaque semaine, les questionnaires remplis seront envoyés à Bujumbura), en utilisant le programme CS Pro. Dans l'ensemble, l'édition des données comprendra la vérification des étendues, la structure des questionnaires et un ensemble de contrôle de cohérence interne. Toutes les erreurs détectées au cours du processus d'édition seront corrigées. Une fois que la vérification et l'apurement des données auront été terminées, les dates imputées seront ajoutées au fichier de données, les facteurs de pondération seront calculés et ajoutés au fichier de données et tous les tableaux nécessaires pour le rapport préliminaire et le rapport final seront produits et vérifiés.

[4] Sauf pour la grille des droits décisionnels.
[5] Uniquement pour la partie sur les informations générales.

3. Analyse et publication des résultats de l'enquête

L'INSP produira un rapport général sur la collecte des données ainsi que le dictionnaire des variables[6]. L'équipe d'évaluation IMT Blue Square analysera ensuite les données.

Il est prévu que l'équipe d'évaluation produise dans un premier temps un rapport synthétique sur la performance des 90 centres de santé évalués, en termes de gestion ainsi qu'en termes d'activités de prévention et lutte contre la malnutrition. Les résultats de ce rapport, d'une quinzaine de pages, seront présentés publiquement lors de l'atelier d'assignation aléatoire du statut intervention ou contrôle, prévu la dernière semaine de mai 2014. L'équipe de l'IMT Blue Square sera chargé de la publication de 100 copies de ce rapport.

L'équipe d'évaluation IMT Blue Square préparera également un rapport plus complet sur l'ensemble des résultats de l'enquête au niveau du pays, ainsi que sur la comparabilité des centres de santé du groupe de contrôle avec ceux du groupe d'intervention. Un premier jet de ce rapport sera produit courant juillet ; il sera finalisé et présenté au Burundi au cours de l'automne 2014.

Dès que le rapport final sera disponible, l'équipe d'évaluation IMT Blue Square conjointement avec l'équipe de l'INSP organiseront un séminaire national de présentation des résultats. Selon un plan de communication et de diffusion des données (cf. Memorandum d'entente), il sera organisé des séminaires régionaux afin de présenter les résultats provinciaux aux autorités locales.

4. Propriété des données

Un *memorandum d'entente* sur la propriété, l'accès et l'utilisation des données est prévu d'être signé par le MSPLS et la Banque Mondiale, propriétaires des données, et l'IMT Blue Square, qui a conçu la méthodologie de l'étude et analysera les données dans ce cadre. Ce *memorandum d'entente* spécifie que les bases de données brutes comprenant des listes nominatives devront en permanence rester confidentielles. En revanche, les données ne comportant aucune liste nominatives le seront provisoirement, tant que les résultats de l'étude d'impact –développés principalement par l'équipe IMT Blue Square– ne seront pas validés et diffusés par la Banque Mondiale et le MSPLS. Par la suite, elles ne seront plus confidentielles et pourront être partagées avec des chercheurs burundais ou internationaux : ceux-ci devront pour cela soumettre une demande au MSPLS et à la Banque Mondiale, avant d'obtenir l'autorisation d'accès et d'analyse des données.

Equipes

L'équipe de préparation et collecte des données : l'INSP

Comme dit plus haut, l'INSP sera responsable de l'exécution de l'enquête, en particulier la préparation et la réalisation du travail sur le terrain, le traitement des données collectées et la rédaction des rapports d'enquête, et donc le responsable final de l'enquête. L'INSP fournira les

[6] Le dictionnaire des variables sera en fait très largement préalablement préparé par l'équipe d'évaluation IMT Blue Square, lors de la phase de préparation des masques de saisie sous ODK et CS Pro. L'INSP devra simplement le compléter compte tenu des données obtenues sur le terrain.

locaux nécessaires devant servir de bureau central pour le personnel de l'enquête. L'INSP sera responsable de la gestion du budget de l'enquête EI-FBPNut-CdS I. Il assurera aussi d'autres tâches administratives. Le personnel de l'INSP sera responsable de la supervision des opérations techniques quotidiennes, y compris le recrutement et la formation du personnel de terrain et de traitement et la supervision des activités de bureau et de terrain.

L'équipe d'évaluation IMT Blue Square

L'équipe d'évaluation IMT Blue Square assurera un suivi technique continu de la préparation et de l'exécution de l'enquête. L'équipe sera aussi responsable de l'analyse des données et de la diffusion des résultats au Burundi. Le principal investigateur de l'étude au sein de l'équipe d'évaluation IMT est Bruno Meesen, IMT ; les co-principal investigateurs sont Patrick Kolsteren et Dominique Roberfroid, IMT.

L'équipe de conception méthodologique et analyse des données est en effet composée de deux experts en nutrition, tous deux basés à l'Institut de Médecine Tropicale d'Anvers (IMT) : Dominique Roberfroid , médecin et expert en nutrition et épidémiologie, ayant 13 ans d'expérience dans les pays à revenus faible et intermédiaire, et Patrick Kolsteren , médecin, pédiatre et expert en nutrition et santé de l'enfant. Tous deux ont une expérience conséquente en méthodes d'évaluations qualitatives et quantitatives, notamment en essais contrôlés randomisés. Est également dans l'équipe Epco Hasker, médecin épidémiologiste avec plus de 15 ans d'expérience, notamment dans la conception de procédures d'échantillonnage, la collecte de données sur le terrain, et l'analyse de données ; son rôle dans ce projet est principalement d'assurer la qualité des données d'enquête. Deux économistes de l'IMT sont également fort impliqués dans cette évaluation d'impact : Catherine Korachais, chercheur ayant 10 ans d'expérience en économie du développement et de la santé et de solides compétences quantitatives ; et Bruno Meessen, chercheur avec 20 ans d'expérience en santé publique ; son expertise dans le financement des soins de santé dans les pays à faibles revenus, et notamment en financement basé sur la performance, permet de bien concevoir le design de la recherche et de déceler les implications politiques, et permettra de proposer de bonnes recommandations au Burundi.

Mais tout ceci ne peut se faire sans l'implication de chercheurs Burundais. En effet, à travers son partenariat avec Blue Square, l'équipe d'évaluation inclut deux chercheurs burundais, Léonard Ntakarutimana, expert en santé publique et parasitologue avec plus de 10 ans d'expérience en tant que chercheur et conseiller politique de santé publique ; et Manassé Nimpagaritse, médecin et spécialiste en santé publique, avec 10 ans d'expérience. Ce dernier poursuit son doctorat sur le sujet, sous la supervision de Bruno Meessen (IMT) et de Jean Macq (Université Catholique de Louvain, Belgique), et en collaboration avec l'INSP. Enfin, l'équipe dispose également d'un expert en qualité de collecte de données, Désiré Munezero, démographe et statisticien avec plus de 5 années d'expérience dans la formation et la supervision de collecte de données, ainsi que la gestion des données. Son expertise sera utile à l'INSP pour la bonne réalisation de l'enquête.

Le MSPLS

Le Ministère de la Santé Publique collabore à cette étude, car ses résultats lui seront utiles pour affiner sa politique de financement de la santé à travers le FBP et bien définir le volet nutrition du

FBP. Aussi, le MSPLS est copropriétaire des données (avec la Banque Mondiale). Des comités de pilotage et technique de l'étude sont instaurés dans ce cadre. Enfin, la participation de certains membres du MSPLS dans l'écriture des rapports et articles sera de mise.

La Banque Mondiale

La Banque Mondiale, à travers le *Health Results Innovation Trust Fund* (HRITF), est l'institution commanditaire de l'étude.

Aussi, c'est la Banque Mondiale qui finance l'intervention pilote de FBP Nutrition (dont la mise en œuvre dépend du Ministère chargé de la Santé), ainsi que l'évaluation d'impact de cette intervention. En effet, un élément central du HRITF est de mieux comprendre et documenter l'ampleur avec laquelle les politiques FBP sont efficaces, réalisables, et dans quelles circonstances. L'évaluation rigoureuse du programme de FBP Nutrition ici discutée permettra de générer de nouvelles connaissances qui peuvent informer le gouvernement du Burundi, mais aussi les autres gouvernements et partenaires pour concevoir et utiliser efficacement les mécanismes de FBP. Ainsi, la Banque Mondiale assure également le financement de l'enquête (assurée par l'INSP) et de l'étude d'impact (assurée par l'IMT Blue Square). Elle est ainsi copropriétaire des données (avec le MSPLS, cf. Memorandum d'Entente). Les membres burundais et internationaux de l'équipe de la Banque Mondiale pourront être impliqués dans la rédaction des rapports.

L'équipe de la Banque Mondiale se compose de la manière suivante :

- Health Sector Development Support Project task team:
    - Driss M. Zine-Eddine E. (Senior Health Economist, Task Team Leader, AFTHW)
    - Alain Desire Karibwami (Health Specialist, Co-Task Team Leader, AFTHE)
    - Tomo Morimoto (Operations Officer, AFTHW)
    - Clarette Rwagatore (Program Assistant)
    - Nicole Hamon (Language Program Assistant, AFTHW)
    - Rigobert Mpendwanzi (Short-Term Consultant, PBF Specialist, AFTHE)
    - Richard Shugugu (Short-Term Consultant, Demographer, AFTHE)
    - Lyse Kanyambo (Team Assistant)
- Health Nutrition and Population team (technical support):
    - Christel Vermeersch (Senior Economist, HDNHE)
    - Elisa Rothenbuhler (Health Economist, HDNHE)
    - Paul Jacob Robyn (Health Economist, AFTHW)

Annexe

Calendrier / Diagramme de Gantt

Planning de la préparation et collecte des données ici présentée

	Jan ' 14	Fév ' 14	Mars ' 14	Avril ' 14	Mai ' 14	Juin ' 14	Juillet	Août ' 14	Sept. ' 14	Oct. ' 14
**Autorisation éthique**										
Protocole d'enquête adapté et soumis au Comité éthique		[bleu]								
Autorisation éthique, visa statistique, autorisation terrain				[bleu]						
**Préparation enquête et collecte**										
Pré-test du questionnaire & adaptation du questionnaire		[vert]								
Protocole d'enquête final, plan écrit du travail de terrain		[bleu]	[bleu]							
Protocole de saisie des données, programme sous ODK			[vert]							
Recrutement des enquêteurs et superviseurs			[bleu]							
Achat du matériel nécessaire réalisé				[bleu]						
Formation des enquêteurs				[bleu]						
Test pilote final à la fin de la formation				[bleu]						
Collecte des données				[bleu] X						
Nettoyage et gestion des données				[bleu]						
Livraison des données (y c. dictionnaire et rapports de collecte)				[bleu]						
Travail d'analyse des données					[vert]	[vert]	[vert]	[vert]	[vert]	
Rapport sur la performance des CdS, Présentation publique					[vert]					
Rapport général, première version							[vert]			
Rapport général, version finale, Présentation publique									[vert]	[vert]

Note : En bleu, l'INSP est leader, en vert, c'est l'IMT Blue Square.

Planning des vagues de collectes de données : baseline et vagues de suivi

		Janvier	Février	Mars	Avril	Mai	Juin	Juillet	Août	Septembte	Octobre	Novembre	Décembre
Baseline (EI-FBPNut-CdS I)	2014					X							
Suivi 1 (EI-FBPNut-CdS II)	2015							X					
Suivi 2 (EI-FBPNut-CdS III)	2016							X					

Budget

Il est prévu un budget de 70,000 USD pour cette enquête. Cela sera entièrement financé par la Banque Mondiale.

Questionnaires

Module (1) Qualité de la prise en charge thérapeutique des cas de malnutrition aigüe modérée et sévère

EN DEBUT DE JOURNEE, DEMANDER AU **RESPONSABLE DES SERVICES NUTRITIONNELS** (OU A DEFAUT, A SON REMPLAÇANT) L'ACCES AUX FICHES CLINIQUES INDIVIDUELLES DES SERVICES SSN ET STA.

SELECTIONNER LES 12 FICHES SSN ET LES 12 FICHES STA A RETRANSCRIRE SELON LE PROTOCOLE DECRIT DANS LE MANUEL.

Formulaire STA

ATTENTION ! NE PRENDRE QUE LES FICHES DES ENFANTS DE MOINS DE 5 ANS (60 MOIS) ET DONT L'ENTREE DANS LE SERVICE STA REMONTE A 6 MOIS AU PLUS[7] ET QUI SONT DEJA SORTIS DU SERVICE STA. AU CAS OU CERTAINES INFORMATIONS MANQUENT SUR LES FICHES, ESSAYER DE COMPLETER AVEC CELLES QUI SE TROUVENT DANS LE REGISTRE D'ENTREE OU DANS LE REGISTRE DE SORTIE DU SERVICE STA.

UNE FOIS CE TRAVAIL FAIT, AVANT DE DEMARRER LA RETRANSCRIPTION INDIVIDUELLE DE CHAQUE FICHE, REMPLIR LE CADRE SUIVANT :

**id001**	Date d'enquête	Jour/mois/année	|__|__|/|__|__|/|__|__|
**id002**	Code de l'enquêteur		|__|__|
**id003**	**Code de la province**	**Voir codes provinces**	|__|__|
**id004**	**Code FOSA**	**Voir codes FOSA**	|__|__||__|__||__|__|
**sta001**	**Nombre total de fiches cliniques STA sur les 6 derniers mois**		|__|__|__

PUIS POUR CHAQUE FICHE :

**id001**	**Date d'enquête**	**Jour/mois/année**	|__|__|/|__|__|/|__|__|
**id002**	Code de l'enquêteur		|__|__|
**id003**	**Code FOSA**		|__|__|
**ADMISSION**			
**sta002**	N° MAS		|__|__||__|__|__|
**sta003**	L'âge de l'enfant est-il renseigné ?	1. Oui (en mois)<br>8. Non renseigné	|__|__|
**sta004**	Sexe de l'enfant	1. Garçon<br>2. Fille<br>8. Non renseigné	|__|
**sta005**	Province		|__|__|
**sta006**	Commune		|__|__|
**sta007**	Colline		|__|__|
**sta008**	Sous-colline		|__|__|
**sta009**	Date d'entrée STA	Jour/mois/année	|__|__|/|__|__|/|__|__|

[7] Si l'enquête a lieu en mai 2014, cela signifie qu'on ne prend que les fiches dont la date d'entrée est comprise entre novembre 2013 et avril 2014 compris.

**sta010**	Référé par	1. Agent de santé<br>2. Hôpital<br>3. Suivi de la croissance<br>4. Autre FOSA<br>5. Visite spontanée<br>6. SSN<br>7. Autre (à préciser)<br>8. Non renseigné	|__|
**sta011**	L'enfant a-t-il déjà été traité pour malnutrition	1. Oui<br>0. Non<br>8. Non renseigné	|__|
**sta012**	Le poids à l'entrée est-il renseigné ?	1. Oui (en kg)<br>8. Non renseigné	|__|__|,|__|
**sta013**	La taille à l'entrée est-elle renseignée ?	1. Oui (en cm)<br>8. Non renseigné	|__|__|__|,|__|
**sta014**	Le périmètre brachial à l'entrée est-il renseigné ?	1. Oui (en mm)<br>8. Non renseigné	|__|__|__|
**sta015**	Œdèmes à l'entrée	0. Non (ou 0)<br>1. +<br>2. ++<br>3. +++<br>8. Non renseigné	|__|
**sta016**	Test de l'appétit	1. Bon<br>2. Moyen<br>3. Refus<br>8. Non renseigné	|__|
**TRAITEMENT**			
**sta017**	Nombre total de visites		|__|__|
**sta018**	Nombre total de paquets de Plumpy Nut		|__|__|__|
**sta019**	Nombre de visites sans Plumpy Nut		|__|__|
**sta020**	Autres nourritures reçues ?	1. Oui (à préciser))<br>0. Non	-------------------------------------
**sta021**	Traitement reçu ?<br><br>QUESTION A CHOIX MULTIPLE	1.Amoxycilline ?<br>2.Vitamine A ?<br>3.Acide folique ?<br>4.Al-/Me-bendazole ?<br>7. Autres<br>8. Non renseigné	|__| |__| |__| |__| |__| |__| |__|
**sta022**	L'enfant a-t-il reçu le vaccin anti-rougeoleux ?	1. Oui<br>0. Non<br>8. Non renseigné	|__|
**sta023**	L'enfant a-t-il reçu un traitement anti-paludéen ?	1. Oui<br>0. Non<br>8. Non renseigné	|__|

SORTIE

**sta024**	Date de sortie	Jour/mois/année	|__|__|/|__|__|/|__|__|
**sta025**	Poids à la sortie	En kilos<br>9. Non renseigné	|__|__|,|__|
**sta026**	Taille à la sortie	En cm<br>9. Non renseigné	|__|__|__|,|__|
**sta027**	Périmètre brachial à la sortie	En mm<br>9. Non renseigné	|__|__|__|
**sta028**	Œdèmes à la sortie	0. Non (ou 0)<br>1. +<br>2. ++<br>3. +++<br>8. Non renseigné	|__|
**sta029**	Raison de sortie	1. Transfert au SST<br>2. Transfert au SSN<br>3. Guérison<br>4. Abandon<br>5. Décès<br>6. Non répondant<br>7. Autre (à préciser)<br>8. Non renseigné	|__| ----------------------------------

sta030 COMMENTAIRES DE L'ENQUETEUR :

Outils

FICHE INDIVIDUELLE DE SUIVI AU SERVICE THERAPEUTIQUE AMBULATOIRE (STA)

Nom du STA/CDS:__________________________

Nom du patient		Poids cible		N° MAS:

Age : **Sexe :**
Province :..............**Commune**......................**Colline :**...................**Sous colline :**..............................
Nom du père :... **Nom de la mère :**..

Semaine	1	2	3	4	5	6	7	8	9	10	11	12
Date												
**Anthropométrie**												
Poids *(kg)*												
Perte de poids * *(Oui/Non)*												
Taille *(cm)*												
P/T en ET												
PB *(mm)*												
IMC												
Œdème *(0, + ++ +++)*												
*** Changement de poids (dans le cas d'un MARASME): si en-dessous du poids d'admission en semaine 3, transférer au Service de Stabilisation (SSt) pour un suivi**												
**Histoire Médicale**												
Diarrhée *(# jours)*												
Vomissements *(# jours)*												
Fièvre *(# jours)*												
Toux *(# jours)*												
**Examen physique**												
Température *(°C)*												
Fréquence respiratoire *(# / min)*												
Déshydratation *(Oui/Non)*												
Anémie *(Oui/Non)*												
Infections cutanées *(Oui/Non)*												

**Semaine**	**1**	**2**	**3**	**4**	**5**	**6**	**7**	**8**	**9**	**10**	**11**	**12**
**Résultat du Test d'Appetit** ***Bon / Moyen / Refus***												
Traitement Spécifique ** *(Oui/Non) annoter*												
Autres médicaments prescrits *(annoter)*												
PlumpyNut *(nbre unités données)*												
Nom de l'examinateur												
**RESULTAT à la sortie**												

G = guéri A = abandon (3 absences consécutives) T = transfert au SST
D = décès NR = Non répondant AU = Autre (Annoter)

**Poids à la sortie**		**Taille à la sortie**		**PB à la sortie**		**P/T (ET) à la sortie**	
**IMC à la sortie**		**Nom du SSN où le patient est référé**					

	**Semaine**	**1**	**2**	**3**	**4**	**5**	**6**	**7**	**8**	**9**	**10**
**MEDICAMENTS SYSTEMATIQUES**	**Amoxycilline**		*7 jours pour les cas admis directement au STA. Ne pas donner à la soties du SST*								
	**Vitamine A**		*Si n'en n'a pas reçu dans le mois précédent*					**Examen Laboratoire**			
	**Acide Folique**		*Si signes d'anémie seulement*				**TYPE**				
	**Albendazole/ Mébendazole**		*A l'admission pour tous les cas*				**RESULTATS**				
**MEDICAMENTS SPECIFIQUES**	**Vaccin anti-rougeoleux.**										
	**Paludisme**										
	**Autre:**										

Actions prises ***(préciser la date)*** **ou remarques :**

Formulaire SSN

ATTENTION ! NE PRENDRE QUE LES FICHES DES ENFANTS DE MOINS DE 5 ANS (60 MOIS) ET DONT L'ENTREE DANS LE SERVICE SSN REMONTE A 6 MOIS AU PLUS[8] ET QUI SONT DEJA SORTIS DU SERVICE SSN. AU CAS OU CERTAINES INFORMATIONS MANQUENT SUR LES FICHES, ESSAYER DE COMPLETER AVEC CELLES QUI SE TROUVENT DANS LE REGISTRE D'ENTREE OU DANS LE REGISTRE DE SORTIE DU SERVICE SSN.

AVANT DE DEMARRER LA RETRANSCRIPTION INDIVIDUELLE DE CHAQUE FICHE :

**id001**	**Date d'enquête**	**Jour/mois/année**	**|__|__|/|__|__|/|__|__|**
**id002**	Code de l'enquêteur		|__|__|
**id003**	**Code de la province**	**Voir codes provinces**	**|__|__|**
**id004**	**Code FOSA**	**Voir codes FOSA**	**|__|__||__|__||__|__|**
**ssn001**	**Nombre total de fiches cliniques SSN sur les 6 derniers mois**		**|__|__|__|__**

PUIS POUR CHAQUE FICHE :

**Id001**	**Date d'enquête**	**Jour/mois/année**	**|__|__|/|__|__|/|__|__|**
**Id002**	Code de l'enquêteur		|__|__|
**Id003**	Code FOSA		|__|__|
**ADMISSION**			
**ssn002**	N° d'enregistrement		|__|__||__|__|__|
**ssn003**	L'âge de l'enfant est-il renseigné ?	1. Oui (en mois)<br>8. Non renseigné	|__|__|
**ssn004**	Sexe de l'enfant	1. Garçon2. Fille<br>8. Non renseigné	|__|
**ssn005**	Province		|__|__|
**ssn006**	Commune		|__|__|
**ssn007**	Colline		|__|__|
**ssn008**	Sous-colline		|__|__|
**ssn009**	Date d'entrée SSN	Jour/mois/année	|__|__|/|__|__|/|__|__|

[8] Si l'enquête a lieu en mai 2014, cela signifie qu'on ne prend que les fiches dont la date d'entrée est comprise entre novembre 2013 et avril 2014 compris.

**ssn010**	Référé par	1. Agent de santé<br>2. Hôpital<br>3. Suivi de la croissance<br>4. Autre FOSA<br>5. Visite spontanée<br>6. STA<br>7. Autre (à préciser)<br>8. Non renseigné	|__|
**ssn011**	L'enfant a-t-il déjà été traité pour malnutrition	1. Oui<br>0. Non<br>8. Non renseigné	|__|
**ssn012**	Le poids à l'entrée est-il renseigné ?	1. Oui (en kg)<br>8. Non renseigné	|__|__|,|__|
**ssn013**	La taille à l'entrée est-elle renseignée ?	1. Oui (en cm)<br>8. Non renseigné	|__|__|__|,|__|
**ssn014**	Le périmètre brachial à l'entrée est-il renseigné ?	1. Oui (en mm)8. Non renseigné	|__|__|__|
**ssn015**	Œdèmes à l'entrée	0. Non (ou 0)<br>1. +<br>2. ++<br>3. +++<br>8. Non renseigné	|__|
**TRAITEMENT**			
**ssn016**	Nombre total de visites		|__|__|
**ssn017**	Quantité totale de CSB reçue	kilos	|__|__|,|__|
**ssn018**	Quantité totale d'huile reçue	litres	|__|__|,|__|
**ssn019**	Quantité totale de sucre reçue	kilos	|__|__|,|__|
**ssn020**	Autres nourritures reçues ?	1.Oui (à préciser)<br>0.Non_(-> ssn022)	|__|
**ssn021**	Quantité totale d'autres nourritures reçues ?		|__|__|,|__|
**ssn022**	Nombre de visites sans nourriture reçue ?		|__|__|
**ssn023**	Traitement reçu ?	1.Al-/Me-bendazole ?<br>2.Vitamine A ?<br>3.Fer folate ?<br>7Autres (à préciser)	|__| |__| |__| |__|
**SORTIE**			
**ssn024**	Date de sortie	Jour/mois/année	|__|__|/|__|__|/|__|__|
**ssn025**	Le poids à la sortie est-il renseigné ?	1. Oui (en kg)8. Non renseigné	|__|__|,|__|
**ssn026**	La taille à la sortie est-elle renseignée ?	1. Oui (en cm)<br>8. Non renseigné	|__|__|__|,|__|

**ssn027**	Le périmètre brachial à la sortie est-il renseigné ?	1. Oui (en mm)<br>8. Non renseigné	|__|__|__|
**ssn028**	Œdèmes à la sortie	0. Non (ou 0)<br>1. +<br>2. ++<br>3. +++<br>8. Non renseigné	|__|
**ssn029**	Raison de sortie	1. Transfert au SST<br>2. Transfert au STA<br>3. Guérison 4. Abandon<br>5. Décès<br>6. Non répondant<br>7. Autre (à préciser)<br>8. Non renseigné	|__|

ssn030 COMMENTAIRES DE L'ENQUETEUR :

Outils

RÉPUBLIQUE DU BURUNDI
MINISTÈRE DE LA SANTÉ PUBLIQUE

PROGRAMME NATIONAL INTÉGRÉ D'ALIMENTATION ET DE NUTRITION (PRONIANUT)

FICHE DE SUIVI INDIVIDUELLE DANS LE SSN

Nom du SSN/ CDS : ____________________

**Nom du patient**							**N° d'enregistrement:**					
**Adresse/ Provenance**	Province………………… Colline :…………………			Commune: …………… Sous colline…………………			**Age:**			**Sexe:**		
**Semaine**	**1**	**2**	**3**	**4**	**5**	**6**	**7**	**8**	**9**	**10**	**11**	**12**
Date												
**Anthropométrie**												
Poids *(kg)*												
Perte de poids * *(Oui/Non)*												
Taille *(cm)*												
P/T (ET)												
PB *(mm)*												
IMC												
Œdèmes *(0, + ++ +++)*												
*** Changement du poids nécessite un suivi et visite à domicile pour investiguer les causes de la perte**												
**Ration reçue (vivres) – Indiquez type et quantité (CSB, huile, etc)**												
CSB												
Huile												
Sucre												
**Traitement Systématique**												
Albdendazole/ Mebendazole												
Vitamin A												
Fer folate												
**RESULTAT a la sortie (type)**												
**G = guéri A = abandon (3 absences consécutives) T = transfert au SST ou STA D = décès NR = Non répondant AU = Autre (a noter)**												

**Poids à la sortie**		**PB à la sortie**		**Taille à la sortie**		**P/T à la sortie (il faut > -2 ET)**		**IMC à la sortie**	

Questionnaire C

INFO ENQUETEUR : REFEREZ-VOUS AU MANUEL DE L'ENQUETEUR POUR LA SELECTION DES CONSULTATIONS A OBSERVER ET POUR LE CONSENTEMENT ECLAIRE

Q1 : Informations générales

ID001	DATE DE L'ENQUETE (JOUR/MOIS/ANNEE)		|__|__|/|__|__|/|__|__|
ID002	CODE DE L'ENQUETEUR		|__|__|
ID003	CODE DE LA PROVINCE		|__|__|
ID004	CODE DE LA FOSA		|__|__|/|__|__|/|__|__|
C101	NOM ET PRENOM DE L'AGENT DE SANTE		
C102	CODE DE L'AGENT DE SANTE	VOIR CODES DANS LA GRILLE DU PERSONNEL (QUESTIONNAIRE G)	|__|__|__|
C103	SEXE DE L'AGENT DE SANTE	1. HOMME<br>2. FEMME	|__|
C104	AGE DE L'AGENT DE SANTE		|__|__|
C105	A SIGNE LE CONSENTEMENT ECLAIRE ?	1. OUI0. NON : FIN DU QUESTIONNAIRE	|__|
C106	CODE DE L'ACCOMPAGNANT DE L'ENFANT		|__|__|__|
C107	SEXE DE L'ACCOMPAGNANT DE L'ENFANT	1. HOMME2. FEMME	|__|
C108	AGE DE L'ACCOMPAGNANT DE L'ENFANT		|__|__|
C109	A SIGNE LE CONSENTEMENT ECLAIRE ?	1. OUI0. NON : FIN DU QUESTIONNAIRE	|__|

HEURE AU DEBUT DE LA CONSULTATION	__ __ : __ __
**Raison de la consultation par l'accompagnant de l'enfant:**	

LA SUITE EST A REMPLIR SUR PAPIER.

Q2 : Quelles questions sont posées à l'accompagnant de l'enfant?

ICI POUR CHAQUE LIGNE COCHER « OUI » (1) LORSQUE L'AGENT DE SANTE POSE LA QUESTION, PUIS INDIQUER LA REPONSE DU PATIENT OU DE L'ACCOMPAGNANT DANS LA COLONNE « REPONSE ». UNE FOIS LA CONSULTATION TERMINEE, COCHER « NON » (0) SI LA QUESTION N'A PAS ETE POSEE (ET N'INDIQUER RIEN DANS LA COLONNE REPONSE).

Questions	Oui	Non	Réponse
c201 Age de l'enfant (mois) ?	1	0	
c202 Sexe de l'enfant ?	1	0	
c203 Présence de diarrhée ?	1	0	
c203_1 Durée de la diarrhée	1	0	
c203_2 Sang dans les selles ?	1	0	
c203_3 Douleurs/crampes abdominales ?	1	0	
c204 Présence de toux ?	1	0	
c204_1 Toux productive ou sèche ?	1	0	
c204_2 Durée de la toux	1	0	
c204_3 Douleurs de gorge?	1	0	
c204_4 Difficultés à respirer ?	1	0	
c205 Appétit ?	1	0	
c205_1 L'allaitez-vous encore ?	1	0	
c205_2 Combien de fois depuis ce matin/hier ?	1	0	
c205_3 Capable de boire/d'avaler ?	1	0	
c205_4 Vomissements ?	1	0	
c205_5 Quel autre aliment reçoit-il d'habitude ?	1	0	
c205_6 A quel âge (mois) avez-vous commencé les aliments ?	1	0	
c205_7 Qu'a-t-il mangé depuis hier ?	1	0	
c205_8 Alimentation changée récemment ?	1	0	
c206 Présence de fièvre ?	1	0	
c206_1 Périodicité ?	1	0	
c206_2 Sudation/frissons ?	1	0	
c206_3 Présence de convulsions ?	1	0	
c206_4 Altération de la conscience ?	1	0	
c207 Quelles maladies a-t-il déjà eu auparavant ?	1	0	
c208 A reçu des médicaments récemment?	1	0	
c209 Combien de médicaments ?	1	0	

Q3: Quel examen médical a été réalisé ?

ICI POUR CHAQUE LIGNE COCHER « OUI » (1) LORSQUE L'AGENT DE SANTE REALISE L'EXAMEN, PUIS INDIQUER LE RESULTAT DE L'EXAMEN DANS LA COLONNE « RESULTATS ». UNE FOIS LA CONSULTATION TERMINEE, COCHER « NON » (0) SI L'EXAMEN N'A PAS ETE REALISE.

	Examens	Oui	Non	Résultats
c301	Etat général de l'enfant ?	1	0	
	c301_1 Température ?	1	0	
	c301_2 Pouls ?	1	0	
	c301_3 Rythme respiratoire ?	1	0	
	c301_4 Tirage sous-costal	1	0	
c302	Déshydratation ?	1	0	
	c302_1 Yeux enfoncés récemment ?	1	0	
	c302_2 Pli cutané ?	1	0	
c303	Signes d'anémie ?	1	0	
c304	Raideur de la nuque ?	1	0	
c305	Fontanelle bombée	1	0	
c306	Examiner la gorge et les oreilles	1	0	
c307	Examiner rate/foie ?	1	0	
c308	Malnutrition ?	1	0	
	c308_1 Poids ?	1	0	
	c308_2 Forme de la courbe de croissance ?	1	0	
	c308_3 Taille ?	1	0	
	c308_4 Circonférence brachiale ?	1	0	
	c308_5 Œdèmes aux pieds ?	1	0	
	c308_6 Z-score calculé	1	0	
	c308_7 Test d'appétit	1	0	
c309	Signes de rougeole ?	1	0	
c310	Etat vaccinal ?	1	0	
c311	Examens de laboratoire ? Lesquels ? (notez les résultats)			
	c311_1	1	0	
	c311_2	1	0	
	c311_3	1	0	
	c311_4	1	0	

Q4 : Quel(s) diagnostic(s) a(ont) été posé(s) ?

ICI POUR CHAQUE LIGNE COCHER « OUI » (1) LORSQUE L'AGENT DE SANTE POSE LE DIAGNOSTIC, PUIS INDIQUER LES EXPLICATIONS DE L'AGENT DANS LA COLONNE « EXPLICATIONS DE L'AGENT ». UNE FOIS LA CONSULTATION TERMINEE, COCHER « NON » (0) POUR TOUS LES DIAGNOSTICS QUI N'ONT PAS ETE POSES.

	Diagnostics	Oui	Non	Explications de l'agent de santé
c401	Malaria sévère (malaria + anémie)	1	0	
c402	Malaria	1	0	
c403	Anémie	1	0	
c404	Méningite	1	0	
c405	Malnutrition compliquée (pneumonie)	1	0	
c406	Pneumonie	1	0	
c407	Malnutrition modérée	1	0	
c408	Malnutrition sévère	1	0	
c409	Déshydratation	1	0	

**c410**	**Parasitose intestinale**	1	0
**c410**	**Diarrhée**	1	0
**c411**	**Manque d’appétit**	1	0
**c412**	**Retard de croissance**	1	0
**c413**	**Ne sait pas**	1	0

Q5 : Quels traitements ou actions ont-été entrepris ?

ICI POUR CHAQUE LIGNE COCHER « OUI » (1) LORSQUE L'AGENT DE SANTE ENTREPREND LE TRAITEMENT OU L'ACTION, PUIS INDIQUER LES EXPLICATIONS DE L'AGENT DANS LA COLONNE « EXPLICATIONS DE L'AGENT ». UNE FOIS LA CONSULTATION TERMINEE, COCHER « NON » (0) POUR TOUS LES TRAITEMENTS OU ACTIONS QUI N'ONT PAS ETE ENTREPRIS.

	Traitements ou actions	Oui	Non	Explications de l'agent de santé
c501	Traitement anti-malarique ? Lequel ?	1	0	
c502	Traitement antibiotique ? Lequel ? Pour quelle indication ?	1	0	
c503	Traitement antipyrétique ? Lequel ?	1	0	
c504	Prescription fer+acide folique	1	0	
c505	Prescription de SRO ?	1	0	
c506	Prescription d'antiparasitaire ?	1	0	
c507	Envoyer l'enfant au SSN ?	1	0	
c508	Envoyer l'enfant au STA ?	1	0	
c509	Référer l'enfant au SST ?	1	0	
c510	Renvoyer l'enfant à la maison et donner une date de visite de suivi ?	1	0	
c511	Garder l'enfant en observation dans la FOSA ?	1	0	
c512	Compléter les vaccinations ?	1	0	
c513	Donner des conseils diététiques ? Lesquels ?	1	0	-<br>-<br>-<br>-
c514	Donner des conseils sur l'hygiène ? Lesquels ?	1	0	-<br>-<br>-
c515	Expliquer à la maman l'utilisation des médicaments ?	1	0	
c516	Expliquer à la maman les signes de danger ?	1	0	
c517	Expliquer à la maman l'urgence d'aller au SST et ce qui va s'y passer ?	1	0	
c518	Discuter avec la maman les actions possibles et lui demander son avis ?	1	0	
c519	S'assurer que la maman a bien compris les explications ?	1	0	
c520	Demander à la maman si elle a d'autres questions à poser ?	1	0	

Heure à la fin de la consultation	__ __ : __ __

INFO ENQUETEUR : EMMENEZ LE PARENT AUPRES DE L'ENQUETEUR CHARGE DE L'INTERVIEW DE SORTIE ET COMMUNIQUEZ LUI LE CODE D'IDENTIFICATION DU PARENT. PUIS POSEZ LA QUESTION 6 A L'AGENT DE SANTE INTERVIEWE.

Q6: Quelle est la perception de l'agent de santé par rapport à cette consultation ?

**c601**	**Avez-vous trouvé cette consultation difficile ?**	0. Non<br>1. Oui	|__|
**c602**	**Diriez-vous de cette consultation qu'elle était**	1.De très bonne qualité<br>2. De bonne qualité<br>3. De qualité acceptable<br>4. De qualité faible<br>5. De très faible qualité	|__|
**c603**	**Selon vous, y avait-il des aspects particulièrement bien fait par vous ?**	--	
**c604**	**Quels aspects auriez-vous aimé améliorer ?**	--	
**c605**	**Avez-vous d'autres commentaires ?**	--	

Q7. Commentaires de l'enquêteur

**c701**	**Durée de la consultation ?**	minutes	|__|__|
**c702**	**Diriez-vous de cette consultation qu'elle était (en termes thérapeutiques)**	1.De très bonne qualité<br>2. De bonne qualité<br>3. De qualité acceptable<br>4. De qualité faible<br>5. De très faible qualité	|__|
**c703**	**Diriez-vous de cette consultation qu'elle était (en termes de conseils)**	1.De très bonne qualité<br>2. De bonne qualité<br>3. De qualité acceptable<br>4. De qualité faible<br>5. De très faible qualité	|__|
**c704**	**Quelle était l'attitude de l'agent de santé par rapport au parent et à son enfant ? (écoute, respect, communication, etc.)**	1.De très bonne qualité<br>2. De bonne qualité<br>3. De qualité acceptable<br>4. De qualité faible<br>5. De très faible qualité	|__|
**c705**	**Quels aspects de la consultation seraient améliorables?**	--	

INFO ENQUETEUR : REMERCIEZ L'AGENT DE SANTE INTERVIEWE POUR SA PARTICIPATION

Questionnaire P

INFO ENQUETEUR : REFEREZ-VOUS AU MANUEL DE L'ENQUETEUR POUR LA SELECTION DES CONSULTATIONS A OBSERVER ET POUR LE CONSENTEMENT ECLAIRE

Q1 : Informations générales

ID001	DATE DE L'ENQUETE (JOUR/MOIS/ANNEE)		|__|__|/|__|__|/|__|__|
ID002	CODE DE L'ENQUETEUR		|__|__|
ID003	CODE DE LA PROVINCE		|__|__|
ID004	CODE DE LA FOSA		|__|__|/|__|__|/|__|__|
P101	NOM ET PRENOM DE L'AGENT DE SANTE		
P102	CODE DE L'AGENT DE SANTE	VOIR CODE DANS LA GRILLE DU PERSONNEL (QUESTIONNAIRE G)	|__|__|__|
P103	SEXE DE L'AGENT DE SANTE	1. HOMME<br>2. FEMME	|__|
P104	AGE DE L'AGENT DE SANTE		|__|__|
P105	A SIGNE LE CONSENTEMENT ECLAIRE ?	1. OUI<br>0. NON : FIN DU QUESTIONNAIRE	|__|
P106	CODE DE L'ACCOMPAGNANT DE L'ENFANT		|__|__|__|
P107	SEXE DE L'ACCOMPAGNANT DE L'ENFANT	1. HOMME<br>2. FEMME	|__|
P108	AGE DE L'ACCOMPAGNANT DE L'ENFANT		|__|__|
P109	A SIGNE LE CONSENTEMENT ECLAIRE ?	1. OUI0. NON : FIN DU QUESTIONNAIRE	|__|

HEURE AU DEBUT DE LA CONSULTATION	__ __ : __ __
**Raison de la consultation:**	

LA SUITE EST A REMPLIR SUR PAPIER.

Q2 : Quelles questions sont posées au parent/accompagnant de l'enfant ?

ICI POUR CHAQUE LIGNE COCHER « OUI » (1) LORSQUE L'AGENT DE SANTE POSE LA QUESTION, PUIS INDIQUER LA REPONSE DU PATIENT OU DE L'ACCOMPAGNANT DANS LA COLONNE « REPONSE ». UNE FOIS LA CONSULTATION TERMINEE, COCHER « NON » (0) SI LA QUESTION N'A PAS ETE POSEE (ET N'INDIQUER RIEN DANS LA COLONNE REPONSE).

	Questions	Oui	Non	Réponse
p201	Age de l'enfant (mois) ?	1	0	
p202	Sexe de l'enfant ?	1	0	
p203	L'allaitez-vous encore ?	1	0	
p204	Combien de fois depuis ce matin/hier ?	1	0	
p205	Rencontrez-vous des difficultés pour l'allaiter ?	1	0	
p206	Quel autre aliment reçoit-il d'habitude ?	1	0	
p207	A quel âge (mois) avez-vous commencé ?	1	0	
p208	Qu'a-t-il mangé depuis hier ?	1	0	
p209	A-t-il bon appétit ?	1	0	
p210	Rencontrez-vous des difficultés pour l'alimenter	1	0	
p211	Alimentation changée récemment ?	1	0	
p212	A-t-il eu des problèmes de santé	1	0	
p213	Présence de diarrhée ?	1	0	
p214	Présence de toux ?	1	0	
p215	Présence de fièvre ?	1	0	

Q3: Quel examen médical a été réalisé ?

ICI POUR CHAQUE LIGNE COCHER « OUI » (1) LORSQUE L'AGENT DE SANTE REALISE L'EXAMEN, PUIS INDIQUER LE RESULTAT DE L'EXAMEN DANS LA COLONNE « RESULTATS ». UNE FOIS LA CONSULTATION TERMINEE, COCHER « NON » (0) SI L'EXAMEN N'A PAS ETE REALISE.

	Examens	Oui	Non	Résultats
p301	Etat général de l'enfant ?	1	0	
p302	Déshydratation ?	1	0	
p303	Signes d'anémie ?	1	0	
p304	Malnutrition ?	1	0	
	p304_1 Poids ?	1	0	
	p304_2 Forme de la courbe de croissance ?	1	0	
	p304_3 Taille ?	1	0	
	p304_4 Circonférence brachiale ?	1	0	
	p304_5 Œdèmes aux pieds ?	1	0	
	p304_6 Z-score calculé	1	0	
p305	Etat vaccinal ?	1	0	

Q4 : Quel(s) diagnostic(s) a (ont) été posé(s) ?

ICI POUR CHAQUE LIGNE COCHER « OUI » (1) LORSQUE L'AGENT DE SANTE POSE LE DIAGNOSTIC, PUIS INDIQUER LES EXPLICATIONS DE L'AGENT DANS LA COLONNE « EXPLICATIONS DE L'AGENT ». UNE FOIS LA CONSULTATION TERMINEE, COCHER « NON » (0) POUR TOUS LES DIAGNOSTICS QUI N'ONT PAS ETE POSES.

	Diagnostics	Oui	Non	Explications de l'agent de santé
p401	Croissance normale	1	0	
p402	Malnutrition modérée	1	0	
p403	Malnutrition sévère	1	0	
p404	Déshydratation	1	0	
p405	Manque d'appétit	1	0	
p406	Retard de croissance	1	0	
p407	Stagnation de la croissance	1	0	
p408	Ralentissement de la croissance	1	0	
p409	Ne sait pas	1	0	

Q5 : Quels traitements ou actions ont-été entrepris ?

ICI POUR CHAQUE LIGNE COCHER « OUI » (1) LORSQUE L'AGENT DE SANTE ENTREPREND LE TRAITEMENT OU L'ACTION, PUIS INDIQUER LES EXPLICATIONS DE L'AGENT DANS LA COLONNE « EXPLICATIONS DE L'AGENT ». UNE FOIS LA CONSULTATION TERMINEE, COCHER « NON » (0) POUR TOUS LES TRAITEMENTS OU ACTIONS QUI N'ONT PAS ETE ENTREPRIS.

	Traitements ou actions	Oui	Non	Explications de l'agent de santé
p501	Encourager la maman si bonne croissance	1	0	
p502	Montrer et expliquer la courbe à la maman	1	0	
p503	Envoyer l'enfant au SSN ?	1	0	
p504	Envoyer l'enfant au STA ?	1	0	
p505	Référer l'enfant au SST ?	1	0	
p506	Renvoyer l'enfant à la maison et donner une date de visite de suivi ?	1	0	
p507	Garder l'enfant en observation dans la FOSA ou l'envoyer à la consultation curative ?	1	0	
p508	Compléter les vaccinations ?	1	0	
p509	Donner des conseils diététiques ? Lesquels ?	1	0	-<br>-<br>-<br>-<br>-
p510	Donner des conseils sur l'hygiène ? Lesquels ?	1	0	-<br>-<br>-<br>-
p511	S'assurer que l'accompagnant a bien compris les messages ?	1	0	
p512	S'assurer que l'accompagnant pourra appliquer les recommandations ?	1	0	
p513	Demander si l'accompagnant à d'autres questions ?	1	0	

Heure à la fin de la consultation	__ __ : __ __

INFO ENQUETEUR : EMMENEZ LE PARENT AUPRES DE L'ENQUETEUR CHARGE DE L'INTERVIEW DE SORTIE ET COMMUNIQUEZ-LUI LE CODE D'IDENTIFICATION DU PARENT. PUIS POSEZ LA QUESTION 6 A L'AGENT DE SANTE INTERVIEWE.

Q6: Quelle est la perception de l'agent de santé par rapport à cette consultation ?

**p601**	**Avez-vous trouvé cette consultation difficile ?**	0. Non<br>1. Oui	|__|
**p602**	**Diriez-vous de cette consultation qu'elle était**	1. De très bonne qualité<br>2. De bonne qualité<br>3. De qualité acceptable<br>4. De qualité faible<br>5. De très faible qualité	|__|
**p603**	**Selon vous, y avait-il des aspects particulièrement bien fait par vous ?**	---------------<br>---------------<br>---------------<br>---------------<br>---------------	
**p604**	**Quels aspects auriez-vous aimé améliorer ?**	---------------<br>---------------<br>---------------<br>---------------<br>---------------	
**p605**	**Avez-vous d'autres commentaires ?**	---------------<br>---------------<br>---------------<br>---------------<br>---------------	

Q7. Commentaires de l'enquêteur

**p701**	**Durée de la consultation ?**	minutes	|__|__|
**p702**	**Diriez-vous de cette consultation qu'elle était (en termes thérapeutiques)**	1.De très bonne qualité<br>2. De bonne qualité<br>3. De qualité acceptable<br>4. De qualité faible<br>5. De très faible qualité	|__|
**p703**	**Diriez-vous de cette consultation qu'elle était (en termes de conseils)**	1. De très bonne qualité<br>2. De bonne qualité<br>3. De qualité acceptable<br>4. De qualité faible<br>5. De très faible qualité	|__|
**p704**	**Quelle était l'attitude de l'agent de santé par rapport au parent et à son enfant ? (écoute, respect, communication, etc.)**	1. De très bonne qualité<br>2. De bonne qualité<br>3. De qualité acceptable<br>4. De qualité faible<br>5. De très faible qualité	|__|
**p705**	**Quels aspects de la consultation seraient améliorables?**	---------------<br>---------------	

INFO ENQUETEUR : REMERCIEZ L'AGENT DE SANTE INTERVIEWE POUR SA PARTICIPATION

Questionnaire S

INFO ENQUETEUR : REFEREZ-VOUS AU MANUEL DE L'ENQUETEUR POUR LA SELECTION DES PATIENTS A INTERVIEWER ET POUR LE CONSENTEMENT ECLAIRE.

LE CONSENTEMENT ECLAIRE DOIT ABSOLUMENT ETRE OBTENU POUR TOUTES LES PERSONNES QUI RECOIVENT CE QUESTIONNAIRE.

Q1 : Informations générales

ID001	DATE DE L'ENQUETE (JOUR/MOIS/ANNEE)		|__|__|/|__|__|/|__|__|
ID002	CODE DE L'ENQUETEUR		|__|__|
ID003	CODE DE LA PROVINCE		|__|__|
ID004	CODE DE LA FOSA		|__|__|/|__|__|/|__|__|
S101	CODE DE L'ACCOMPAGNANT DE L'ENFANT		|__|__|__|
S102	SEXE DE L'ACCOMPAGNANT DE L'ENFANT	1. HOMME<br>2. FEMME	|__|
S103	AGE DE L'ACCOMPAGNANT DE L'ENFANT		|__|__|
S104	AVEZ-VOUS SIGNE LE CONSENTEMENT ECLAIRE ?	1. OUI<br>0. NON : FIN DU QUESTIONNAIRE	|__|
S105	CODE DE LA COLLINE D'HABITATION	VOIR CODES COLLINE	|__|__|
S106	CODE DE LA SOUS-COLLINE D'HABITATION	VOIR CODES SOUS-COLLINE	|__|__|
S107	QUELLE EDUCATION AVEZ-VOUS REÇU ?	0.AUCUN NIVEAU<br>1.PRE-PRIMAIRE<br>2.PRIMAIRE<br>3.SECONDAIRE 1ER CYCLE<br>4.SECONDAIRE 2E CYCLE<br>5.SUPERIEUR<br>6. ECOLE INFORMELLE<br>8. NE SAIT PAS	|__|__|
S108	QUEL EST VOTRE STATUT MATRIMONIAL ?	1. CELIBATAIRE<br>2. EN COUPLE/MARIE(E)<br>3.VEUF (VEUVE)	|__|
S109	COMBIEN D'ENFANTS AVEZ-VOUS EU AU TOTAL ?		|__|__|
S110	COMBIEN D'ENFANTS VIVANTS AVEZ-VOUS ?		|__|__|
S111	QUELLE EDUCATION A REÇU VOTRE CONJOINT ?	0.AUCUN NIVEAU<br>1.PRE-PRIMAIRE<br>2.PRIMAIRE<br>3.SECONDAIRE 1ER CYCLE<br>4.SECONDAIRE 2E CYCLE<br>5.SUPERIEUR<br>6. ECOLE INFORMELLE<br>8. NE SAIT PAS	|__|__|
S112	VOTRE MENAGE POSSEDE-T-IL DES VACHES LAITIERES OU TAUREAUX ?	1.OUI<br>0.NON	|__|
S113	VOTRE MENAGE POSSEDE-T-IL DES CHEVRES OU DES MOUTONS ?	1. OUI<br>0. NON	|__|
S114	VOTRE MENAGE POSSEDE-T-IL DES PORCS ?	1. OUI<br>0. NON	|__|

s115 Votre menage possede-t-il des volailles (poules, canard, pintades ou pigeons) ?	1. Oui<br>0. Non	|__|
s116 Votre menage possede-t-il des lapins ?	1. Oui<br>0. Non	|__|
s117 Votre menage possede-t-il des cobayes ?	1. Oui<br>0. Non	|__|
s118 Avez-vous l'eau courante a la maison ?	1. Oui<br>0. Non	|__|
s119 Avez-vous l'electricite a la maison?	1. Oui<br>0. Non	|__|
s120 Votre famille possede-t-elle une montre?	1 Oui<br>0 Non	|__|
s121 Votre famille possede-t-elle un telephone (fixe et/ou portable) ?	1.Oui<br>0. Non	|__|
s122 Votre famille possede-t-elle une radio?	1 Oui<br>0 Non	|__|
s123 Votre famille possede-t-elle une television ?	1. Oui<br>0. Non	|__|
s124 Votre famille possede-t-elle un refrigerateur ?	1.Oui<br>0. Non	|__|
s125 Votre famille possede-t-elle des bicyclettes ?	1 Oui<br>0 Non	|__|
s126 Votre famille possede-t-elle une motocyclette ou scooter ?	1 Oui<br>0 Non	|__|
s127 Votre famille possede-t-elle des charrettes ou charrues ?	1 Oui<br>0 Non	|__|
s128 Votre famille possede-t-elle une voiture ou camionnette ?	1 Oui<br>0 Non	|__|

LA SUITE EST A REMPLIR SUR PAPIER.

Q2: Perception du parent par rapport à cette consultation

s201 De quoi votre enfant souffre-t-il (diagnostic)?	--<br>--<br>--<br>--<br>--<br>---
s202 Comment va-t-on améliorer sa santé (traitement)?	--<br>--<br>--<br>--<br>--<br>---
s203 Avez-vous reçu des conseils sur l'alimentation de l'enfant ? Lesquels ?	--<br>--<br>--<br>--<br>--<br>---

**s204 Quels sont les aspects qui vous ont le plus plu dans cette consultation ?**	---<br>---<br>---<br>---<br>---<br>--
**s205 Quels sont les aspects qui vous ont le moins plus dans cette consultation ?**	---<br>---<br>---<br>---<br>---<br>--
**s206 Diriez-vous que vous êtes satisfaits par :**<br><br>**POUR CHAQUE SOUS QUESTION COCHER LA MODALITE CITEE**<br><br>**s206_1 le temps avant de rencontrer le personnel soignant**<br>**s206_2 la propreté de la formation sanitaire**<br>**s206_3 l'accueil dans la formation sanitaire**<br>**s206_4 le caractère privé (intimité) de la consultation**<br>**s206_5 l'écoute du personnel de santé**<br>**s206_6 la façon du personnel de vous parler**<br>**s206_7 la façon d'examiner votre enfant**<br>**s206_8 le coût des services**<br>**s206_9 le coût des médicaments**<br>**s206_10 la disponibilité des médicaments**<br>**s206_11 les conseils reçus pour le traitement**<br>**s206_12 les conseils reçus pour l'alimentation de l'enfant**<br>**s206_13 les conseils reçus si l'état de l'enfant ne s'améliore pas**	1. Très satisfait<br>2. Moyennement satisfait<br>3. Pas satisfait<br>4. Ne sait pas<br>5. Non concerné<br><br>I__I<br>I__I<br>I__I<br>I__I<br>I__I<br>I__I<br>I__I<br>I__I<br>I__I<br>I__I<br>I__I<br>I__I<br>I__I
**s207 Diriez-vous de cette consultation qu'elle était**	1.De très bonne qualité I__I<br>2. De bonne qualité<br>3. De qualité habituelle<br>4. De qualité faible<br>5. De très faible qualité
**s208 Que Suggéreriez-vous pour améliorer la qualité des services ?**	--<br>--<br>--<br>--<br>--<br>--

Q3. L'interviewer reprend les mesures anthropométriques de l'enfant

s301 Acceptez-vous que l'on prenne les mesures anthropométriques de votre enfant ?	1. Oui<br>0. Non (fin du questionnaire)	|__|
**S302 Est-ce que la balance du CdS est calibrée ? (TEST AVEC UN POIDS FIXE DE 10 KG)**	1. Oui<br>0. Non	
s303 Sexe de l'enfant	1. Masculin<br>2. Féminin	|__|
**S304_1 Poids de l'enfant avec la balance du CdS**	En kilogrammes	|__|__|,|__| kilos
**S304_2 Poids de l'enfant avec la balance SECA**	En kilogrammes	|__|__|,|__| kilos
**S305 Forme de la courbe de croissance**	1. ascendante<br>2. Stationnaire<br>3. Descendante<br>9. Pas de courbe	|__|
**S3036 Taille de l'enfant**	En centimètres	|__|__|__|,|__| cm
**S307 Périmètre brachial de l'enfant**	En millimètres	|__|__|__| mm
**S308 Œdèmes aux pieds**	1. Oui0. Non	|__|

APRES LA PRISE DE MESURES ANTHROPOMETRIQUES, L'ENQUETEUR VERIFIE SI L'ENFANT N'EST PAS MALNUTRI AIGU (MODERE OU SEVERE) OU CHRONIQUE. SI IL L'EST, IL LE DIT A SON ACCOMPAGNANT ET LUI SUGGERE D'ALLER DANS LES SERVICES APPROPRIES.

Module (3) Aspects organisationnels

Questionnaire G

QUESTIONNAIRE A ADRESSER AU **TITULAIRE RESPONSABLE DU CENTRE DE SANTE** (OU A DEFAUT, A SON REMPLAÇANT) AVANT L'OUVERTURE DES SERVICES DE SANTE AU PUBLIC.REFEREZ-VOUS AU MANUEL DE L'ENQUETEUR POUR LE CONSENTEMENT ECLAIRE.

Identification

**id001**	Date d'enquête	Jour/mois/année	|__|__|/|__|__|/|__|__|
**id002**	Code de l'enquêteur		|__|__|
**id003**	**Code de la province**	**Voir codes provinces**	**|__|__|**
**id004**	**Code FOSA**	**Voir codes FOSA**	**|__|__||__|__||__|__|**

Vérification du questionnaire par le contrôleur

Nom du contrôleur	
Code du contrôleur	|_|_|
Date	|_|_|/|_|_|/|_|_|

Vérification du questionnaire par le superviseur

Nom du superviseur	
Code du superviseur	|_|_|
Date	|_|_|/|_|_|/|_|_|

Q1 : Informations sur la personne interrogée

g101	Nom et prénom de l'agent de santé		
g102	Code. de l'agent de santé		|__|__|
g103	Quel poste occupez-vous dans ce centre de santé ?	1 Titulaire du centre de santé<br>2 Titulaire adjoint du centre de santé<br>3 Gestionnaire du centre de santé<br>7 Autre, préciser :	|__|

Q2 : Informations générales sur le centre de santé

g201	Qui est propriétaire de ce centre de santé ?	1 Gouvernement<br>2 Mission / Organisation religieuse<br>3 Organisation privée à but lucratif<br>4 Organisation Non Gouvernementale (ONG)<br>7 Autre, préciser :	|__|
g202	Depuis quelle année ce centre de santé est-il en service ?	Année	|__|__|__|__|
g203	Quelle est la principale source d'approvisionnement en eau ?	1 Canalisation jusque dans l'enceinte du CdS<br>2 Canalisation dans la cour / parcelle occupée<br>3 Robinet public / borne fontaine<br>4 Puits protégé<br>5 Puits non protégé<br>6 Source protégée<br>7 Source non protégée<br>8 Eau de pluie<br>9 Eau superficielle (lac, rivière ou ruisseau)<br>10 Eau embouteillée<br>97 Autre, préciser :	|__|
g204	Le CdS dispose-t-il d'un téléphone fixe et/ou portable ?	1 Oui<br>0 Non	|__|
g205	Le CdS dispose-t-il d'un ordinateur fonctionnel ?	1 Oui<br>0 Non	|__|
g206	Le CdS dispose-t-il d'une imprimante fonctionnelle ?	1 Oui<br>0 Non	|__|
g207	Le CdS dispose-t-il d'un accès, gratuit ou payant, à une photocopieuse fonctionnelle ?	1 Oui<br>0 Non	|__|
g208	Le CdS dispose-t-il d'une connexion internet ?	1 Oui<br>0 Non	|__|

Q3 : Grille du personnel dans ce centre

g301	g302	g303	g304	g305	g306	g307	g308
Code	Nom et prénom de l'agent de santé	Sexe de l'agent de santé	Employeur	Quel type de contrat avec employeur ?	Diplôme le plus élevé ?	Poste en tant que prestataire tel que défini par le MSPLS ?	Année de la dernière formation
		1. Homme<br>2. Femme	1 Etat<br>2 Centre de santé<br>3 ONG<br>7 Autre, préciser :	1 Permanent (public) et donnant droit à une pension (personnel sous statut)<br>2 Personnel sous contrat (Etat)<br>3 Personnel sous contrat (Centre de santé)<br>4 Permanent (Privé) et donnant droit à une pension<br>5 Contrat de courte durée (< 6 mois)<br>6 Contrat à durée déterminée (> 6 mois)<br>7 Informel (pas de contrat)<br>8 Bénévole<br>97 autre, préciser :	1 Niveau A3<br>2 Niveau A2<br>3 Niveau A1<br>4 Niv. Licence<br>5 Médecin généraliste<br>7 Autre, préciser.	01 Titulaire du centre de santé<br>02 Titulaire Adjoint du CDS<br>03 Chef d'équipe des services préventifs<br>04 Infirmier dans le service<br>05 Chef du service laboratoire<br>06 Technicien de laboratoire<br>07 Chef du service de promotion de la santé<br>08 Technicien de Promotion de la Santé (TPS)<br>09 Gestionnaire financier<br>10 Secrétaire<br>11 Médecin<br>12 Gestionnaire de la pharmacie<br>97 Autres	
1							
2							
3							
4							
5							
6							
7							
8							
9							
10							
11							

Q4 : Système de santé

g401	Le CdS fournit il le paquet minimum d'activités au complet?	1 Oui<br>0 Non	|__|
g402	Connaissez-vous la population couverte par ce CdS ?	1. Oui (nombre)<br>8 Ne sait pas	|__|__|__|__|__|__|
g403	Connaissez-vous le nombre d'enfants de moins de 5 ans ?	1. Oui (nombre)<br>8 Ne sait pas	|__|__|
g404	Savez-vous combien de sous-collines sont dans l'aire de responsabilité du CdS ?	1. Oui (nombre)<br>8 N sait pas	|__|__|
g405	Ce centre de santé réfère-t-il des patients vers d'autres formations sanitaires ?	1 Oui<br>0 Non	|__|
g406	Votre centre de santé travaille-t-il avec des agents de santé communautaire ?	1 Oui<br>0 Non	|__|
g407	Savez-vous combien ils sont ?	1. Oui (nombre)<br>8 Ne sait pas	|__|__|
g408	Est-ce que les ASC de l'aire de responsabilité sont organisés en GASC ?	1 Oui<br>0 Non→(Saut à section suivante)	|__|
g409	Est-ce que quelqu'un s'occupe de la relation avec le GASC au sein du personnel ?	1 Oui0 Non	|__|

Q5 : Partenaires techniques et financiers

	g501	g502	g503	g504	g505
	Code PTF	Connaissez-vous la date du début d'appui ?	Connaissez-vous la date prévisionnelle de fin ?	Domaine d'intervention	Couvre toutes les collines/sous-collines de l'aire de responsabilité
		1. Oui (année)<br><br>8. Ne sait pas	1. Oui (année)<br><br>8 Ne sait pas	COCHER LES BONNES REPONSES (CHOIX MULTIPLE)<br>1.Réhabilitation<br>2.FBP<br>3.Intervention à base communautaire<br>4.Système d'Information Sanitaire<br>5.Sécurité alimentaire<br>6.Hygiène et Assainissement<br>7.Alimentation en eau potable<br>8.Lutte contre la maladie(VIH, TBC, Palu, Epidémies, maladies mentales)<br>9.Santé maternelle et infantile(PF, Système de référence et contre référence)<br>10.Nutrition(Appui aux FARN,Regroupement et formation des ASC sur la nutrition,Formation du Personnel du CdS sur la nutrition,<br>Fournit le PlumpyNut,Fournit le CSB/Huile/Sucre,Fournit la nourriture aux accompagnants(ration de protection),anime les séances de CCC,Jardinspotagers,Cantines scolaires, etc.)<br>97.Autres(préciser)	1.Oui<br>2.Non<br>8. Ne sait pas
1					|__|
2					|__|
3					|__|
4					|__|
5					|__|
6					|__|
7					|__|
8					|__|
9					|__|
10					|__|
11					|__|
12					|__|

Q6 : Administration &Gestion – questions générales

g601	Est-ce que le centre de santé dispose d'un comité de gestion?	1 Oui<br>0 Non	|__|
g602	Est-ce que le centre de santé dispose d'un comité de santé ?	1 Oui<br>0 Non	|__|
g603	Procédez-vous à une évaluation régulière des membres du personnel ?	1. Oui<br>0. Non (saut g605)	|__|
g604	Quelle est la périodicité ?	1 Mensuelle<br>2 Trimestrielle<br>3 Semestrielle<br>4 Annuelle<br>7 Autre, préciser.	|__|
g605	Procédez-vous à un inventaire des stocks de médicaments du CdS ?	1 Oui<br>0 Non (saut g607)	|__|
g606	Si oui, quelle en est la périodicité ?	1 Mensuelle<br>2 Trimestrielle<br>3 Semestrielle<br>4 Annuelle<br>7 Autre, préciser.	|__|
g607	Le budget du CdS pour l'exercice 2014 a-t-il été élaboré ?	1 Oui, vu.<br>0 Non (saut g609)	|__|
g608	Si oui, à combien s'élevait-il ?	En millions de FBU	|__|__|__|
g609	Savez-vous combien de réunions techniques ont eu lieu au cours des 3 derniers mois ?	1. Oui (nombre)<br>8. Ne sait pas	|__|
g610	Au cours des 3 derniers mois, savez-vous combien de visites votre centre de santé a reçu en provenance du niveau central dans le cadre de la supervision ou de l'appui technique ?	1. Oui (nombre)8. Ne sait pas	|__||__| <br>|__||__| |__||__|
g611	Au cours des 3 derniers mois, savez-vous combien de visites votre centre de santé a reçu en provenance du niveau provincial dans le cadre de la supervision ou de l'appui technique ?	1. Oui (nombre)<br>8. Ne sait pas	
g612	Au cours des 3 derniers mois, savez-vous combien de visites votre centre de santé a reçu en provenance du niveau du district dans le cadre de la supervision ou de l'appui technique ?	1. Oui (nombre)<br>8. Ne sait pas	
g613	Procédez-vous à des réunions entre managers et employés ?	1. Oui<br>0. Non (saut g615)	|__|
g614	Quelle est la fréquence de ces réunions ?	1. Bi-mensuelle<br>2. Mensuelle<br>3. Trimestrielle<br>4. Semestrielle<br>5. Annuelle<br>7. Autre (à préciser)	|__|
g615	Quelles sont les **sources de financement** des activités du	1 Paiements directs des patients<br>2 La MFP	

	centre de santé ?<br><br>LIRE ET COCHER TOUTES LES REPONSES POSITIVES.	3 La CAM<br>4 La FDN<br>5 La PNB<br>6 Les employeurs privés<br>7 Les mutuelles privées<br>8 Subsides FBP<br>9 Subsides de l'Etat<br>10 Dons<br>11 Ministère de la Solidarité Nationale<br>97 Autre, préciser.	
g616	Votre centre de santé vend-il des **cartes d'assurance maladie** (CAM) ?	1 Oui<br>0 Non	|__||__|
g617	Depuis quel date ?	Mois, Année	|__||__| / |__|__|__|__|
g618	Le personne médical subit-il régulièrement des examens de dépistage ?	1. Oui<br>0. Non<br>8. Ne sait pas	|__|
g619	Pour quelles maladies ?		
g620	Est-ce un automatisme en cas d'accident du travail impliquant un matériel souillé ?	1. Oui<br>0. Non	|__|
g621	Des réunions de partage des connaissances sont-elles organisées suite à la formation des membres du personnel ?	1. Oui, toujours<br>2. Oui, parfois<br>0. Non	|__|
g622	Parmi les documents suivants, lesquels sont présents ET à jour au sein du CdS ?<br><br>LIRE LES CHOIX POSSIBLE ET NOTER LES REPONSES CITEES	1. Organigramme de la structure<br>2. Registre des présences<br>3. Registre de garde<br>4. Contrats de travail du personnel propre à la structure<br>5. Fiches de poste<br>6. Registre / fiches de suivi des formations<br>7. Suivi des accidents du travail<br>8. Documents de statut du personnel	|__| |__| |__| |__| |__| |__| |__| |__|

Q7. Organisation interne générale – droits décisionnels

A.	Si vous deviez prendre une décision de ce type (cf tableau suivant), qui serait impliqué dans la décision?	1 : il fait partie des décideurs<br>0 : il ne décide rien<br>9 : non applicable	|__|

A REMPLIR SUR PAPIER.

POUR CHAQUE LIGNE, POSER LA QUESTION AU GESTIONNAIRE. PAR EXEMPLE, POUR LA QUESTION G701, DIRE : « SI VOUS DEVIEZ DECIDER DE REMPLACER L'INFIRMIER TITULAIRE ACTUEL PAR UN NOUVEL INFIRMIER TITULAIRE, QUI SERAIT IMPLIQUE DANS CETTE DECISION ? ». SI LE GESTIONNAIRE REPOND « LE PROPRIETAIRE ET LE BDS », ALORS INDIQUER 1 DANS LES COLONNES CORRESPONDANTES ; PUIS INDIQUER 0 DANS TOUTES LES AUTRES COLONNES (REPONSES NON CITEES) DE LA MEME LIGNE.

ATTENTION, AUCUNE CASE NE DOIT RESTER VIDE.

Ressources humaines

		Propriétaire* (congrégation, paroisse, actionnaire, ministère de la santé…)	Comité de gestion du CdS	Infirmier titulaire	Infirmier titulaire Adjoint	Gestionnaire du CDS	Réunion du personnel du CdS	Responsable du service nutritionnel	Autre collègue de la nutrition	Autre collègue du CdS	Bureau du District	Bureau Provincial de Santé	Pronianut	Acteur tiers (ONG…)
g701	Remplacer l'infirmier titulaire actuel par un nouvel infirmier titulaire	|__|	|__|	|__|	|__|	|__|	|__|	|__|	|__|	|__|	|__|	|__|	|__|	|__|
g702	Réallouer les responsabilités au sein de l'équipe du centre de santé pour renforcer le service nutritionnel	|__|	|__|	|__|	|__|	|__|	|__|	|__|	|__|	|__|	|__|	|__|	|__|	|__|
g703	Allouer un nouvel agent statutaire (payé sur budget de l'Etat) pour renforcer l'équipe de nutrition	|__|	|__|	|__|	|__|	|__|	|__|	|__|	|__|	|__|	|__|	|__|	|__|	|__|
g704	Choix de l'identité de l'agent statutaire en question	|__|	|__|	|__|	|__|	|__|	|__|	|__|	|__|	|__|	|__|	|__|	|__|	|__|
g705	Allouer un nouvel agent contractuel (payé sur les recettes de la FoSa) pour renforcer l'équipe de nutrition	|__|	|__|	|__|	|__|	|__|	|__|	|__|	|__|	|__|	|__|	|__|	|__|	|__|

		Propriétaire* (congrégation, paroisse, actionnaire, ministère de la santé…)	Comité de gestion du CdS	Infirmier titulaire	Infirmier titulaire Adjoint	Gestionnaire du CDS	Réunion du personnel du CdS	Responsable du service nutritionnel	Autre collègue de la nutrition	Autre collègue du CdS	Bureau du District	Bureau Provincial de Santé	Pronianut	Acteur tiers (ONG…)
g706	Choix de l'identité de l'agent contractuel en question	|__|	|__|	|__|	|__|	|__|	|__|	|__|	|__|	|__|	|__|	|__|	|__|	|__|
g707	Refuser ou renvoyer un agent statutaire	|__|	|__|	|__|	|__|	|__|	|__|	|__|	|__|	|__|	|__|	|__|	|__|	|__|
g708	Licencier un agent contractuel	|__|	|__|	|__|	|__|	|__|	|__|	|__|	|__|	|__|	|__|	|__|	|__|	|__|
g719	Revoir le système de primes pour obtenir un plus gros effort du personnel de la nutrition	|__|	|__|	|__|	|__|	|__|	|__|	|__|	|__|	|__|	|__|	|__|	|__|	|__|
g710	Choisir la personne qui va suivre une formation en nutrition	|__|	|__|	|__|	|__|	|__|	|__|	|__|	|__|	|__|	|__|	|__|	|__|	|__|
g711	Revoir les horaires de travail	|__|	|__|	|__|	|__|	|__|	|__|	|__|	|__|	|__|	|__|	|__|	|__|	|__|
g712	Convoquer une réunion avec les agents de santé communautaire sur le thème de la nutrition	|__|	|__|	|__|	|__|	|__|	|__|	|__|	|__|	|__|	|__|	|__|	|__|	|__|

Infrastructure et équipement

		Propriétaire* (congrégation, paroisse, actionnaire, ministère de la santé…)	Comité de gestion du CdS	Infirmier titulaire	Infirmier titulaire Adjoint	Gestionnaire	Réunion du personnel du CdS	Responsable du service nutritionnel	Autre collègue de la nutrition	Autre collègue du CdS	Bureau du District	Bureau Provincial de Santé	Pronianut	Acteur tiers (ONG…)
g713	Revoir l'allocation des locaux pour favoriser l'activité nutritionnelle	|__|	|__|	|__|	|__|	|__|	|__|	|__|	|__|	|__|	|__|	|__|	|__|	|__|
g714	Contracter un corps de métier pour une petite réhabilitation de bâtiments pour renforcer le service nutritionnel (financée sur recettes de la FoSa) « petite » correspond à un montant inférieur à 1 million FBU	|__|	|__|	|__|	|__|	|__|	|__|	|__|	|__|	|__|	|__|	|__|	|__|	|__|
g715	Contracter un entrepreneur pour une grosse réhabilitation ou une construction de bâtiments pour renforcer le service nutritionnel (sur recettes de la FoSa)	|__|	|__|	|__|	|__|	|__|	|__|	|__|	|__|	|__|	|__|	|__|	|__|	|__|

		Propriétaire* (congrégation, paroisse, actionnaire, ministère de la santé…)	Comité de gestion du CdS	Infirmier titulaire	Infirmier titulaire Adjoint	Gestionnaire	Réunion du personnel du CdS	Responsable du service nutritionnel	Autre collègue de la nutrition	Autre collègue du CdS	Bureau du District	Bureau Provincial de Santé	Pronianut	Acteur tiers (ONG…)
	« grosse » correspond à un montant jusqu'à 5 millions FBU													
g716	Faire réparer un équipement utile pour la nutrition (sur recettes de la FoSa)	|__|	|__|	|__|	|__|	|__|	|__|	|__|	|__|	|__|	|__|	|__|	|__|	|__|
g717	Acquérir un nouvel équipement pour la nutrition (auprès d'un partenaire)	|__|	|__|	|__|	|__|	|__|	|__|	|__|	|__|	|__|	|__|	|__|	|__|	|__|

Intrants, outils de gestion et médicaments

		Propriétaire* (congrégation, paroisse, actionnaire, ministère de la santé…)	Comité de gestion du CdS	Infirmier titulaire	Infirmier titulaire Adjoint	Gestionnaire du CDS	Gestionnaire de la pharmacie de de stock	Réunion du personnel du CdS	Responsable du service nutritionnel	Autre collègue de la nutrition	Autre collègue du CdS	Bureau du District	Bureau Provincial de Santé	Pronianut	Acteur tiers (ONG…)
g718	Quantité des intrants nutritionnels commandés par la FoSa	|__|	|__|	|__|	|__|	|__|	|__|	|__|	|__|	|__|	|__|	|__|	|__|	|__|	|__|
g719	Quantité des intrants nutritionnels reçus par la FoSa	|__|	|__|	|__|	|__|	|__|	|__|	|__|	|__|	|__|	|__|	|__|	|__|	|__|	|__|
g720	Quantité des médicaments ('nutritionnels') commandés par la FoSa	|__|	|__|	|__|	|__|	|__|	|__|	|__|	|__|	|__|	|__|	|__|	|__|	|__|	|__|
g721	Quantité des medicaments ('nutritionnels') reçus par la FoSa	|__|	|__|	|__|	|__|	|__|	|__|	|__|	|__|	|__|	|__|	|__|	|__|	|__|	|__|
g722	Quantité des outils de gestion 'programme nutritionnel' commandés par la FoSa (y compris périmètres brach)	|__|	|__|	|__|	|__|	|__|	|__|	|__|	|__|	|__|	|__|	|__|	|__|	|__|	|__|
g723	Quantité des outils de gestion 'programme nutritionnel' reçus par la FoSa (y compris périmètres brach)	|__|	|__|	|__|	|__|	|__|	|__|	|__|	|__|	|__|	|__|	|__|	|__|	|__|	|__|
g724	Exécution	|__|	|__|	|__|	|__|	|__|	|__|	|__|	|__|	|__|	|__|	|__|	|__|	|__|	|__|

		Propriétaire* (congrégation, paroisse, actionnaire, ministère de la santé…)	Comité de gestion du CdS	Infirmier titulaire	Infirmier titulaire Adjoint	Gestionnaire du CDS	Gestionnaire de la pharmacie de de stock	Réunion du personnel du CdS	Responsable du service nutritionnel	Autre collègue de la nutrition	Autre collègue du CdS	Bureau du District	Bureau Provincial de Santé	Pronianut	Acteur tiers (ONG…)
	d'une supervision des stocks (confrontation stocks sur fiches avec stocks réels)														
g725	Reproduction d'outils de gestion sur une photocopieuse payante (sur recettes de la FoSa)	|__|	|__|	|__|	|__|	|__|	|__|	|__|	|__|	|__|	|__|	|__|	|__|	|__|	|__|

Module (4) Service nutritionnel

Questionnaire N

QUESTIONNAIRE A ADRESSER AU **RESPONSABLE DES SERVICES NUTRITIONNELS** (OU A DEFAUT, A SON REMPLAÇANT) AVANT L'OUVERTURE DU CENTRE DE SANTE AU PUBLIC. REFEREZ-VOUS AU MANUEL DE L'ENQUETEUR POUR LE CONSENTEMENT ECLAIRE.

Identification

**id001**	Date d'enquête	Jour/mois/année	|__|__|/|__|__|/|__|__|
**id002**	Code de l'enquêteur	Voir codes enquêteur	|__|__|
**id003**	**Code de la province**	**Voir codes provinces**	**|__|__|**
**id004**	**Code FOSA**	**Voir codes FOSA**	**|__|__||__|__||__|__|**

Vérification du questionnaire par le contrôleur

Nom du contrôleur	
Code du contrôleur	|_||_|
Date	|_||_|/|_||_|/|_||_|

Vérification du questionnaire par le superviseur

Nom du superviseur	
Code du superviseur	|_||_|
Date	|_||_|/|_||_|/|_||_|

Q1 : Informations sur la personne interrogée

n101	Nom et prénom de l'agent de santé		
n102	Code de l'agent de santé		|__||__|
n103	Quel poste occupez-vous dans ce centre de santé ?	01 Titulaire du centre de santé<br>02 Titulaire Adjoint du CDS<br>03 Chef d'équipe des services préventifs<br>04 Infirmier dans le service<br>05 Chef du service laboratoire<br>06 Technicien de laboratoire<br>07 Chef du service de promotion de la santé<br>08 Technicien de Promotion de la Santé (TPS)<br>09 Gestionnaire financier<br>10 Secrétaire<br>11 Médecin<br>12 Gestionnaire de la pharmacie<br>97 Autres	|__|

Q2 : Informations générales sur les services de prévention et prise en charge de la malnutrition

	**Détection**		
n201	Votre CDS offre-t-il un **programme de suivi de la croissance** ?	1 Oui<br>0 Non (-> n214)	|__|
n202	Si oui, quels sont les indicateurs anthropométriques utilisés ?<br><br>COCHER LES REPONSES POSITIVES(VERIFIER SYSTEMATIQUEMENT LES REGISTRES)	1 Poids<br>2 Taille<br>3 PB<br>7 Autres(à préciser)	|__|
n203	Est-ce que l'activité de suivi de la croissance est réalisée dans une salle de consultation ?	1 Oui, systématiquement<br>2 Oui, parfois<br>0 Non	|__|
n204	Dans le cadre de l'activité de suivi de la croissance, prodiguez-vous des conseils individualisés ou en groupe ?	1) conseils individualisés<br>2) conseils en groupe<br>0) pas de conseils	|__|
n205	La courbe de croissance est-elle remplie dans le carnet de l'enfant ?<br>VERIFIER 10 CARNETS	1 Oui, systématiquement<br>2 Oui, parfois<br>0 Non	|__|
n206	Utilisez-vous un protocole particulier dans le suivi de la croissance ?<br>VERIFIER	1 Oui, vu<br>0 Non	|__|
n207	Le protocole de suivi de la croissance est-il affiché ?<br>VERIFIER	1 Oui, vu<br>0 Non	|__|
n208	Est-ce que votre CdS fournit des **services de détection de la malnutrition** ?<br>VERIFIER LES REGISTRES DE CONSULTATION	1 Oui<br>0 Non	|__|

n209	A quelles occasions le personnel de votre CdS détecte-t–il les problèmes de malnutrition ?<br><br>COCHER LA/LES REPONSE(S) CITEE(S) (PLUSIEURS REPONSES POSSIBLES)	1 Chez la femme enceinte en Consultation Prénatale<br>2 Chez la femme allaitante en consultation post-natale<br>3 Chez l'enfant lors des séances de vaccination<br>4 Chez l'enfant en consultation de nourrisson sain<br>5 Chez l'enfant en consultation curative<br>6 Chez l'adulte en consultation curative<br>7 Chez l'enfant en consultation nutrition (référence ASC ou directe)<br>97 Autre, préciser.	|__|
n210	Est-ce que la détection des problèmes de malnutrition est réalisée dans une salle de consultation ?	1 Oui, systématiquement<br>2 Oui, parfois<br>0 Non	|__|
n211	Dans le cadre de l'activité de détection de malnutrition, prodiguez-vous des conseils individualisés ?	1) conseils individualisés<br>2) conseils en groupe<br>0) pas de conseils	|__|
n212	Les grilles (tables) poids/taille et la formule de calcul de l'IMC sont-elles affichées dans le service ?<br>VERIFIER SI C'EST AFFICHE DANS CE SERVICE ET VERIFIER SI C'EST AFFICHE EGALEMENT EN CC ET VACCINATION	1 Oui, les deux<br>2 Oui, uniquement la grille poids/taille<br>3. Oui, uniquement la formule de calcul<br>0 Non	|__|
n213	Quels sont les outils et **matériel** disponibles dans votre CdS pour la détection de la malnutrition et le suivi de la croissance ? Pour chaque matériel, encercler le chiffre uniquement lorsqu'il est présent en quantité au moins égale au chiffre entre parenthèses<br><br>COCHER LES REPONSES CITEES	1 Toise(min 2)<br>2 Balance Salter(min 2)<br>3 Pèse-bébé(min 2)<br>4 Mètre ruban ou Bracelet PB (min 2)<br>5 Grille ou table poids/taille (min 3)<br>6 IMC pour les enfants de plus de 5 ans(min 3)<br>7.Culottes (min. 4) ou bassines en plastique<br>8.Balance adulte électronique<br>9Balance électronique mère enfant	|__|

n214	Quels sont les outils et **matériel** **<u>FONCTIONNELS ?</u>**<br><br>QUESTION A CHOIX MULTIPLE<br>COCHER LES BONNES REPONSES	1 Toise(min 2)<br>2 Balance Salter(min 2)<br>3 Pèse-bébé(min 2)<br>4 Mètre ruban ou Bracelet PB (min 2)<br>5 Grille ou table poids/taille (min 3)<br>6 IMC pour les enfants de plus de 5 ans(min 3)<br>7.Culottes (min. 4) ou bassines en plastique<br>8.Balance adulte électronique<br>9. Balance électronique mère enfant	
	**SSN**		
n215	Est-ce que votre CdS fournit un **service de supplémentation nutritionnelle** ?	1 Oui<br>0 Non (-> n241)	|__|
n216	Savez-vous depuis quelle année ce CDS dispose-t-il d'un SSN ?	1. Oui (année)<br><br>8. Ne sait pas	|__|__|__|__|
n217	Est-ce que le service de supplémentation nutritionnelle (SSN) est donné dans une pièce de consultation à part ?	1 Oui, systématiquement<br>2 Oui, parfois<br>0 Non	|__|
n218	A quelle fréquence votre CdS fournit-il un un service de supplémentation nutritionnelle (SSN) ?	1. Plusieurs jours par semaine<br>2.1 jour par semaine (-> n219)<br>3.1 jour par deux semaines (-> n219)<br>7. Autres(Préciser) (-> n219)<br>8. Ne sait pas (-> n219)	|__|
n218_1	Pendant combien de jours par semaine votre CdS fournit un service de supplémentation nutritionnelle (SSN) ?	Indiquer le nombre de jours (entre 1 et 7)	|__|
n219	Quels sont les noms et prénoms des agents de santé qui fournissent les prestations lors de ce service de SSN ?	Nom Prénom	|__|
n220	Code des agents de santé	Voir codes dans la grille du personnel (questionnaire G)	

n221	Quelsoutils et matériel indispensables au SSN sontdisponibles dans le service ?<br><br>COCHER LES REPONSES CITEES	1.registre de SSN pour les enfants de moins de 5 ans.<br>2. registre de SSN pour les femmes enceintes et allaitantes et les autres cas<br>3.Cartes de suivi individuel au SSN<br>4.Registre de dépistage<br>5.Fiches des statistiques hebdomadaires/mensuelles SSN (rapport mensuel)<br>6.Fiche de rapport de distribution<br>7.Fiche de bilan de stock<br>8.Registre de stock (pour le contrôle de l'approvisionnement)<br>9.Fiche de bordereau de livraison<br>10.Mesurettes<br>11. Toise(min. 2)<br>12.Balance Salter (min. 2)<br>13.Culottes (min. 4) ou bassines en plastique<br>14.Balance adulte électronique<br>15.Balance électronique mère enfant<br>16.Mètre ruban ou Bracelet PB<br>17.Table Référence OMS pour le Poids/Taille<br>18.Table IMC<br>19.Matériel d'éducation (boites à images, dépliants et affiches)<br>20.Matériels de démonstration culinaire<br>21.Grand bassin et spatules	|__||__|
n222	Quels sont les outils à jour ?	1.registre de SSN pour les enfants de moins de 5 ans.<br>2. registre de SSN pour les femmes enceintes et allaitantes et les autres cas<br>3.Cartes de suivi individuel au SSN<br>4.Registre de dépistage<br>5.Fiches des statistiques hebdomadaires/mensuelles SSN (rapport mensuel)<br>6.Fiche de rapport de distribution<br>7.Fiche de bilan de stock<br>8.Registre de stock (pour le contrôle de l'approvisionnement)<br>9.Fiche de bordereau de livraison	

n223	Quel **matériel est <u>FONCTIONNEL ?</u>**<br><br>QUESTION A CHOIX MULTIPLE<br>COCHER LES BONNES REPONSES	1.Mesurettes<br>2. Toise(min. 2)<br>3.Balance Salter (min. 2)<br>4.Culottes (min. 4) ou bassines en plastique<br>5.Balance adulte électronique<br>6.Balance électronique mère enfant<br>7.Mètre ruban ou Bracelet PB<br>8.Matériel d'éducation (boites à images, dépliants et affiches)<br>9.Matériels de démonstration culinaire<br>10.Grand bassin et spatules	
n224_1	Les formulaires vierges du registre de SSN pour les enfants de moins de 5 ans peuvent-ils suffire pour un trimestre ?	1. Oui<br>0. Non<br>9. pas de registre ni de fiches en cours d'utilisation	|__|
n224_2	Les formulaires vierges du registre de SSN pour les femmes enceintes et allaitantes et les autres cas peuvent-ils suffire pour un trimestre ?	1. Oui<br>0. Non<br>9. pas de registre ni de fiches en cours d'utilisation	|__|
n224_3	Les formulaires vierges pour les cartes de suivi individuel au SSN peuvent-ils suffire pour un trimestre ?	1. Oui<br>0. Non<br>9. pas de registre ni de fiches en cours d'utilisation	|__|
n224_4	Les formulaires vierges pour le registre de dépistage peuvent-ils suffire pour un trimestre ?	1. Oui<br>0. Non<br>9. pas de registre en cours d'utilisation	|__|
n224_5	Les formulaires vierges pour la fiche des statistiques hebdomadaires / mensuelles SSN peuvent-ils suffire pour un trimestre ?	1. Oui<br>0. Non	|__|
n224_6	Les formulaires vierges pour la fiche de rapport de distribution peuvent-ils suffire pour un trimestre ?	1. Oui<br>0. Non<br>9. pas de registre ni de fiches en cours d'utilisation	|__|
n224_7	Les formulaires vierges pour la fiche de bilan de stock peuvent-ils suffire pour un trimestre ?	1. Oui<br>0. Non<br>9. pas de registre ni de fiches en cours d'utilisation	|__|
n224_8	Les formulaires vierges pour le registre de stock (pour le contrôle de l'approvisionnement) peuvent-ils suffire pour un trimestre ?	1. Oui<br>0. Non<br>9. pas de registre ni de fiches en cours d'utilisation	|__|
n225	Quels produits de traitement diététique sont disponibles ?(vérifier sur la fiche de stock)<br>COCHER LE PRODUIT DISPONIBLE	0. Aucun<br>1.CSB<br>2.Huile<br>3.Sucre	|__|

n226	Est-ce que la CMM du CSB est documentée ?	1. Oui<br>9. pas de fiche de stock, ou fiche de stock mal remplie<br>0. Non	|__|
n226_1	Si oui, reporter la CMM du CSB	En kilogrammes, avec une décimale	|__||__||__|.|__|KG
n226_2	Quel sont les stocks restants de CSB aujourd'hui ?<br>VERIFIER LA FICHE DE STOCK	En kilogrammes, avec une décimale	|__||__||__|.|__|KG
n227	Est-ce que la CMM de l'huile est documentée ?	1. Oui<br><br>0. Non<br>9. pas de fiche de stock, ou fiche de stock mal remplie	|__|
n227_1	Si oui, reporter la CMM de l'huile	En litres, avec une décimale	|__||__||__|.|__|KG
n227_2	Quel sont les stocks restants d'huile aujourd'hui ?<br>VERIFIER LA FICHE DE STOCK	En litres, avec une décimale	|__||__||__|.|__|KG
n228	Est-ce que la CMM de sucre est documentée ?	1. Oui,<br><br>0. Non<br>9. pas de fiche de stock, ou fiche de stock mal remplie	|__|
n228_1	Si oui, reporter la CMM du sucre	En kilogrammes, avec une décimale	|__||__||__|.|__|KG
n228_2	Quel sont les stocks restants de sucre aujourd'hui ?<br>VERIFIER LA FICHE DE STOCK	En kilogrammes, avec une décimale	|__||__||__|.|__|KG
n229	Le CDS a-t-il connu des ruptures de stock durant les trois derniers mois pour au moins les produits suivants?<br>QUESTION A CHOIX MULTIPLE<br>(Vérifier sur les fiches de stock)	0. Aucune rupture de stock<br>1. CSB<br>2.Pas de fiche de stock de CSB<br>3. Huile<br>4. Pas de fiches de stock d'huile<br>5. Sucre<br>6.Pas de fiche de stock de sucre	|__|
n230	Le CDS a-t-il connu des ruptures de stock durant les trois derniers mois pour les produits suivants ?<br><br>QUESTION A CHOIX MULTIPLE<br>(Vérifiez sur les fiches de stock)	0. Aucune rupture de stock<br>1.Vitamine A 100 000UI<br>2. Pas de fiche de stock de Vitamine A 100 000UI<br>3.Vitamine A 200 000UI<br>4. Pas de fiche de stock de Vitamine A 200 000UI<br>5.Albendazole ou Mebendazole<br>6. Pas de fiche de stock d'Al/Mebendazole<br>7.Fer/Folate<br>8. Pas de fiche de stock de Fer/Folate<br>9.Vaccin Anti-Rougeoleux<br>10. Pas de fiche de stock de vaccin anti-rougeoleux	|__|

n231	Les indicateurs de performance des SSN sont-ils calculés et rapportés mensuellement (considérez six derniers mois) ?	1 Oui, systématiquement<br>2 Oui, parfois<br>0 Non	|__|
n232	Le CDS s'intéresse-t-il à la récupération des abandons (registre des abandons avec sous-collines et ASC concernés, feed back des ASC)	1.Oui vu<br>0.Non Pas vu	|__|
n233	Au cours des trois derniers mois, le personnel du CDS : titulaire ou infirmier du service ; a-t-il effectué des sorties au niveau communautaire ?(Vérifiez les rapports avec personnes rencontrées)<br>SI BESOIN NOTEZ COMMENTAIRES	1.Oui vu<br>0.Non Pas vu	|__|
	**STA**		
n234	Est-ce que votre CDS fournit un **service de thérapeutique ambulatoire** ?	1 Oui<br>0 Non (-> Passer à la section suivante)	|__|
n235	Savez-vous depuis quelle année ce CDS dispose-t-il d'un STA ?	1. Oui (année)<br>9. Ne sait pas	|__|__|__||__|
n236	Est-ce que le service de thérapeutique ambulatoire (STA) est donné dans une pièce de consultation à part ?	1 Oui, systématiquement<br>2 Oui, parfois<br>3 Non	|__|
n237	A quelle fréquence votre CdS fournit un service de thérapeutique ambulatoire (STA) ?	1. Plusieurs jours par semaine<br>2.1 jour par semaine (-> n238)<br>3.1 jour par deux semaines (-> n238)<br>7. Autre(Préciser) (-> n238)<br>8. Ne sait pas (-> n238)	|__|
n237_1	Combien de jours par semaine ?	Indiquer le nombre de jours (entre 1 et 7)	|__|
n238	Quels sont les noms et prénoms des agents de santé qui fournissent les prestations lors de ce service thérapeutique ambulatoire (STA) ?<br>INSCRIRE NOM ET PRENOM DE CHAQUE AGENT DE SANTE	Nom Prénom	|__|
n239	Code des agents de santé qui fournissent les prestations lors de ce service thérapeutique ambulatoire (STA)	Code de la grille du personnel (questionnaire G)	|__|

n240	Quels sont les outils et matériel disponibles dans le service STA ?<br>QUESTION A CHOIX MULTIPLE<br><br>COCHER LES REPONSES CITEES	1.registre STA pour les enfants de moins de cinq ans et autres admissions.<br>2.Cartes de suivi individuel STA<br>3.Registre de dépistage<br>4.Fiches des statistiques hebdomadaires/mensuelles STA (rapport mensuel)<br>5.Fiche de rapport de distribution<br>6.Fiche de bilan de stock<br>7.Registre de stock (pour le contrôle de l'approvisionnement)<br>8.Fiche de bordereau de livraison<br>9 Toise(min. 2)<br>10.Balance Salter (min. 2)<br>11.Culottes (min. 4) ou bassines en plastique<br>12.Balance adulte électronique<br>13.Balance électronique mère enfant<br>14.Mètre ruban ou Bracelet PB<br>15.Table Référence OMS pour le Poids/Taille<br>16.Table IMC<br>17. Matériel d'éducation (boites à images, dépliants et affiches)	|__|
n241	Quels outils sont à jour ?<br><br>QUESTION A CHOIX MULTIPLE	0. Aucun<br>1.registre STA pour les enfants de moins de cinq ans et autres admissions.<br>2.Cartes de suivi individuel STA<br>3.Registre de dépistage<br>4.Fiches des statistiques hebdomadaires/mensuelles STA (rapport mensuel)<br>5.Fiche de rapport de distribution<br>6.Fiche de bilan de stock<br>7.Registre de stock (pour le contrôle de l'approvisionnement)<br>8.Fiche de bordereau de livraison	
n242	Quel est le **matériel** <u>**FONCTIONNEL ?**</u><br><br>QUESTION A CHOIX MULTIPLE<br>COCHER LES BONNES REPONSES	1. Toise(min. 2)<br>2.Balance Salter (min. 2)<br>3.Culottes (min. 4) ou bassines en plastique<br>4.Balance adulte électronique<br>5.Balance électronique mère enfant<br>6.Mètre ruban ou Bracelet PB<br>7.Matériel d'éducation (boites à images, dépliants et affiches)<br>8.Matériels de démonstration culinaire<br>9.Grand bassin et spatules	

n243_1	Les formulaires vierges pour le registre STA pour les enfants de moins de 5 ans et autres admissions peuvent-ils suffire pour un trimestre ?	1. Oui<br>0. Non<br>9. pas de registre ni de fiches en cours d'utilisation	|__|
n243_2	Les formulaires vierges pour les cartes de suivi individuel au STA peuvent-ils suffire pour un trimestre ?	1. Oui<br>0. Non<br>9. pas de registre ni de fiches en cours d'utilisation	|__|
n2431_3	Les formulaires vierges pour le registre de dépistage peuvent-ils suffire pour un trimestre ?	1. Oui<br>0. Non<br>9. pas de registre ni de fiches en cours d'utilisation	|__|
n243_4	Les formulaires vierges pour la fiche des statistiques hebdomadaires / mensuelles SSN peuvent-ils suffire pour un trimestre ?	1. Oui<br>0. Non	|__|
n243_5	Les formulaires vierges pour la fiche de rapport de distribution peuvent-ils suffire pour un trimestre ?	1. Oui<br>0. Non<br>9. pas de registre ni de fiches en cours d'utilisation	|__|
n243_6	Les formulaires vierges pour la fiche de bilan de stock peuvent-ils suffire pour un trimestre ?	1. Oui<br>0. Non<br>9. pas de registre ni de fiches en cours d'utilisation	|__|
n243_7	Les formulaires vierges pour le registre de stock (pour le contrôle de l'approvisionnement) peuvent-ils suffire pour un trimestre ?	1. Oui<br>0. Non<br>9. pas de registre ni de fiches en cours d'utilisation	|__|
n244	Le produit de traitement diététique (Plumpy nut) est-il disponible (vérifier sur la fiche de stock)	1.Oui<br>0.Non	|__|
n245	Est-ce que la CMM de Plumpy Nut est documentée ?	1. Oui,<br>0. Non	|__|
n245_1	Si oui, reporter la CMM du Plumpy Nut	Nombre de sachets	|__||__||__|
n245_2	Quel sont les stocks restants de Plumpy Nut aujourd'hui ?<br>VERIFIER LA FICHE DE STOCK	Nombre de sachets	|__||__||__|
n246	Le CDS a-t-il connu des ruptures de stock durant les trois derniers mois pour ce produit (Vérifiez sur les fiches de stock)	1. Oui<br>0. Non<br>9.Pas de fiche de stock	|__|
n247	Quels produits de traitement Médical systématique sontdisponibles au CDS ?<br><br>QUESTION A CHOIX MULTIPLE<br>COCHER LES PRODUITS DISPONIBLES	0. Aucun<br>1.Vitamine A 100 000UI(6-12 mois)<br>2.Vitamine A 200 000UI(›12 mois)<br>3.Acide folique<br>4. Amoxicilline<br>5. ASAQ<br>6.Albendazole ou Mebendazole (›12mois)<br>7. Vaccin Anti-Rougeoleux	|__|
n248	Est-ce que la CMM de Vitamine A 100000 UI est documentée ?	1. Oui,<br>0. Non	|__|

n248_1	Si oui, reporter la CMM de Vitamine A 100 000 UI	En boites	|__||__||__|
n248_2	Quel sont les stocks restants de Vitamine A 100 000 UI aujourd'hui ?<br>VERIFIER LA FICHE DE STOCK	En boites	|__||__||__|
n249	Est-ce que la CMM de Vitamine A 200000 UI est documentée ?	1. Oui,<br>0. Non	|__|
n249_1	Si oui, reporter la CMM de Vitamine A 200000 UI	En boites	|__||__||__|
n249_2	Quel sont les stocks restants de Vitamine A 200000 UI aujourd'hui ?<br>VERIFIER LA FICHE DE STOCK	En boites	|__||__||__|
n250	Est-ce que la CMM d'acide folique est documentée ?	1. Oui,<br>0. Non	|__|
n250_1	Si oui, reporter la CMM d'acide folique	En boites	|__||__||__|
n250_2	Quel sont les stocks restants d'acide folique aujourd'hui ?<br>VERIFIER LA FICHE DE STOCK	En boites	|__||__||__|
n251	Est-ce que la CMM d'amoxicilline en gélules de 250mg est documentée ?	1. Oui,<br>0. Non	|__|
n251_1	Si oui, reporter la CMM d'amoxicilline en gélule de 250 mg	En boites	|__||__||__|
n251_2	Quel sont les stocks restants d'amoxicilline en gélule de 250 mg aujourd'hui ?<br>VERIFIER LA FICHE DE STOCK	En boites	|__||__||__|
n252	Est-ce que la CMM d'amoxicilline en gélule de 500 mg est documentée ?	1.Oui<br>0.Non	|__|
n252_1	Si oui, reporter la CMM d'amoxicilline en gélule de 500 mg	En boites	|__||__||__|
n252_2	Quel sont les stocks restants d'amoxicilline en gélule de 500 mg aujourd'hui ?<br>VERIFIER LA FICHE DE STOCK	En boites	|__||__||__|
n253	Est-ce que la CMM d'amoxicilline en suspension de 125 mg est documentée ?	1.Oui<br>0.Non	|__|
n253_1	Si oui, reporter la CMM d'amoxicilline en suspension de 125 mg	En boites	|__||__||__|
n253_2	Quel sont les stocks restants d'amoxicilline en suspension de 125 mg aujourd'hui ?<br>VERIFIER LA FICHE DE STOCK	En boites	|__||__||__|
n254	Est-ce que la CMM d'amoxicilline en suspension de 250 mg est documentée ?	1.Oui<br>0.Non	|__|
n254_1	Si oui, reporter la CMM d'amoxicilline en suspension de 250 mg	En boites	|__||__||__|
n254_2	Quel sont les stocks restants d'amoxicilline en suspension de 250 mg aujourd'hui ?<br>VERIFIER LA FICHE DE STOCK	En boites	|__||__||__|
n255	Est-ce que la CMM d'ASAQ est documentée ?	1. Oui,<br>0. Non	|__|

n255_1	Si oui, reporter la CMM d'ASAQ	En boites	|__||__||__|
n255_2	Est-ce que la CMM d'ASAQ est documentée ?	En boites	|__||__||__|
n256	Est-ce que la CMM d'Albendazole est documentée ?	1. Oui,<br>0. Non	|__|
n256_1	Si oui, reporter la CMM d'Albendazole	En boites	|__||__||__|
n256_2	Quel sont les stocks restants d'Albendazole aujourd'hui ?<br>VERIFIER LA FICHE DE STOCK	En boites	|__||__||__|
n257	Est-ce que la CMM de Mebendazole est documentée ?	1.Oui<br>0.Non	|__|
n257_1	Si oui, reporter la CMM de Mebendazole	En boites	|__||__||__|
n257_2	Quel sont les stocks restants de Mebendazole aujourd'hui ?<br>VERIFIER LA FICHE DE STOCK	En boites	|__||__||__|
n258	Est-ce que la CMM de vaccin anti-rougeoleux est documentée ?	1. Oui,<br>0. Non	|__|
n258_1	Si oui, reporter la CMM de vaccins anti-rougeoleux	En ampoules	|__||__||__|
n258_2	Quel sont les stocks restants de vaccins anti-rougeoleux aujourd'hui ?<br>VERIFIER LA FICHE DE STOCK	En ampoules	|__||__||__|
n259	Le CDS a-t-il connu des ruptures de stock durant les trois derniers mois pour les produits suivants ?<br><br>QUESTION A CHOIX MULTIPLE<br>(Vérifiez sur les fiches de stock)	0. Aucune rupture de stock<br>1.Amoxicilline en gélules de 250 mg<br>2. Pas de fiche de stock d'amoxicilline en gélules de 250 mg<br>3. Amoxicilline en gélules de 5000 mg<br>4. Pas de fiche de stock d'amoxicilline en gélules de 500 mg<br>5. Amoxicilline en suspension de 125 mg<br>6. Pas de fiche de stock d'amoxicilline en suspension de 125 mg<br>7. Amoxicilline en suspension de 250 mg<br>8. Pas de fiche de stock d'amoxicilline en suspension de 250 mg<br>9. ASAQ<br>10. Pas de fiche de stock d'ASAQ	|__|
n260	Les indicateurs de performance des STA sont-ils calculés et rapportés mensuellement (considérez les six derniers mois) ?	1 Oui, systématiquement<br>2 Oui, parfois<br>0 Non	|__|
n261	Le CDS s'intéresse-t-il à la récupération des abandons (registre des abandons avec sous-collines et ASC concernés, feed back des ASC)	1.Oui vu<br>0.Non, pas vu	|__|

n262	Au cours des trois derniers mois, le personnel du CDS : titulaire ou infirmier du service ; a-t-il effectué des sorties au niveau communautaire ?<br>(Vérifiez les rapports avec personnes rencontrées)<br>SI BESOIN NOTEZ COMMENTAIRES	1.Oui vu<br>0.Non, pas vu	|__|

Q3 : Grille du personnel dans les services liés à la malnutrition

n301	n302	n303	n304	n305	n306	n307	n308	n309	n310	n311	n312
Id #	Nom et prénom de l'agent de santé	Statut de l'agent de santé<br><br>1 Infirmier de la FOSA<br>2 TPS de la FOSA/Communal<br>3 ASC<br>7 Autre, préciser.	Quelle sont les activités spécifiques de l'agent de santé ?<br><br>1.Mesures anthropométriques en CC<br>2.Mesures anthropométriques en consultations enfants sains<br>3.Mesures anthropométriques en vaccination<br>4. Mesures anthropométriques en service nutritionnel<br>5.CCC<br>6.Distribution des intrants thérapeutiques nutritionnels<br>7.Rapportage/Statistique<br>8.Encadrement des ASC/GASC<br>97.Autres(Préciser)	Rôle dans le suivi de la croissance<br><br>1 responsable<br>2 agent de santé subordonné<br>3 personnel d'appui<br>4 Aucun rôle dans ce service<br>7 Autre, préciser :	Combien d'heures par semaine travaille-t-il dans ce service ?	Rôle dans le service de détection de malnutrition<br><br>1 responsable<br>2 agent de santé subordonné<br>3 personnel d'appui<br>4 Aucun rôle dans ce service<br>7 Autre, préciser :	Combien d'heures par semaine travaille-t-il dans ce service ?	Rôle dans le SSN<br><br>1 responsable<br>2 agent de santé subordonné<br>3 personnel d'appui<br>4 Aucun rôle dans ce service<br>7 Autre, préciser :	Combien d'heures par semaine travaille-t-il dans ce service ?	Rôle dans le STA<br><br>indiquer son rôle dans service :<br>1 responsable<br>2 agent de santé subordonné<br>3 personnel d'appui<br>4 Aucun rôle dans ce service<br>7 Autre, préciser :	Combien d'heures par semaine travaille-t-il dans ce service ?

	n313	n314	n315	n316	n317
Id #	A t-il reçu une formation en matière de détection et de prise en charge de la malnutrition ?<br><br>1 Oui, 0 Non	A quand remonte la dernière formation reçue par vos personnels en matière de détection et de prise en charge de la malnutrition<br><br>NB MOIS	(Nom) Est-il/elle présent aujourd'hui?<br><br>1 Oui<br>0 Non	Si NON Pourquoi?<br><br>1. Congé annuel de repos<br>2. Congé de maladie<br>3. Congé de maternité<br>4. En formation<br>5.Absence autorisée<br>6. Absence non autorisée<br>7 .En retard<br>97. Autre(Préciser)	Qui le remplace dans ses activités ?<br><br>Notez le code correspondant de la colonne 4 du précédent tableau

Q4 : Locaux

n401	Dans quel endroit l'activité de suivi de la croissance est-elle exécutée ?	1. Salle d'attente de la FOSA<br>2. Salle de consultation curative<br>3. Salle de vaccination<br>4. Local séparé du bloc principal<br>5. Couloir<br>6. Local de stockage des intrants thérapeutiques<br>7. Dans la cour<br>96. Il n'y a pas d'activité de suivi de la croissance<br>97. Autre (à préciser)<br>98. Ne sait pas	|__|
n402	Dans quel endroit l'activité de dépistage de la malnutrition est-elle exécutée ?	1. Salle d'attente de la FOSA<br>2. Salle de consultation curative<br>3. Salle de vaccination<br>4. Local séparé du bloc principal<br>5. Couloir<br>6. Local de stockage des intrants thérapeutiques<br>7. Dans la cour<br>96. Il n'y a pas d'activité de dépistage<br>97. Autre (à préciser)<br>98. Ne sait pas	|__|
n403	Dans quel endroit l'activité de SSN est-elle exécutée ?	1. Salle d'attente de la FOSA<br>2. Salle de consultation curative<br>3. Salle de vaccination<br>4. Local séparé du bloc principal<br>5. Couloir<br>6. Local de stockage des intrants thérapeutiques<br>7. Dans la cour<br>96. Il n'y a pas de SSN<br>97. Autre (à préciser)<br>98. Ne sait pas	|__|
n404	Dans quel endroit l'activité de STA est-elle exécutée ?	1. Salle d'attente de la FOSA<br>2. Salle de consultation curative<br>3. Salle de vaccination<br>4. Local séparé du bloc principal<br>5. Couloir<br>6. Local de stockage des intrants thérapeutiques<br>7. Dans la cour<br>96. Il n'y a pas de STA<br>97. Autre (à préciser)<br>98. Ne sait pas	|__|
n405	Où sont stockés les intrants thérapeutiques pour le traitement de la malnutrition aigüe modérée ?	1 Dans la pharmacie<br>2 Dans une pièce à part fermée à clé<br>3 Dans la salle de consultation du SSN<br>7 Autre, préciser.	|__|
n406	Si la pièce de stockage est fermée à clé, qui dispose de la clé ?	1 …<br>2 …	|__|

n407	Où sont stockés les intrants thérapeutiques pour le traitement de la malnutrition aigüe sévère ?	1 Dans la pharmacie<br>2 Dans une pièce à part fermée à clé<br>3 Dans la salle de consultation du STA<br>7 Autre, préciser.	|__|
n408	Si la pièce de stockage est fermée à clé, qui dispose de la clé ?<br>CODE DE L'AGENT DE SANTE	1 …<br>2 …	|__|

Q5 : Système de santé

n501	Lorsque vous avez un enfant malnutri sévère avec complications, le référez-vous vers une FOSA ?	1. Oui<br>0. Non<br>8 Ne sait pas	______
n502	Code de la FOSA		|__|__|
n503	Est-ce que vous recevez un feed-back à propos des enfants que vous avez référés vers d'autres formations sanitaires ?	1. Oui<br>2. Non<br>3. Non applicable	
n504	Connaissez-vous la distance entre votre centre de santé et cette formation sanitaire ?	1. Oui (Nb de kilomètres en aller-simple)<br>98 Ne sait pas	|__|__|
n505	Existe-t-il des centres de santé qui réfèrent vers votre centre de santé en cas de malnutrition aigüe modérée ?	1. Oui<br>0. Non<br>8. Ne sait pas	___________
n506	Quels sont ces CDS ?	Code du CDS	
n507	Connaissez-vous la distance entre votre centre de santé et ceux-ci ?	1. Oui (Nb de kilomètres en aller-simple)<br>98. Ne sait pas	
n508	Existe-t-il descentres de santé qui réfèrent vers votre centre de santé en cas de malnutrition aigüe sévère ?	Nom de la FOSA	
n509	Quels sont ces CDS ?	Code du CDS	
n510	Connaissez-vous la distance entre votre centre de santé et ceux-ci ?	1. Oui (Nb de kilomètres en aller-simple)<br>98 Ne sait pas	|__|__|
n511	Est-ce que vous donnez un feed-back à propos des enfants que vous avez accepté d'autres formations sanitaires ?	1. Oui<br>2. Non<br>3. Non applicable	
n512	Savez-vous combien de sous-collines couvrent les services de prise en charge de la malnutrition ?	1. Oui (Nombre)<br>98 Nesait pas	|__|__|

Q6 : Activités de nutrition au niveau communautaire

n601	Avez-vous connaissances d'activités nutritionnelles à base communautaire dans votre aire de responsabilité ?	1 Oui<br>0 Non	|__|
n602	Si oui, lesquelles, et sous l'encadrement de qui ?	1 Activités de dépistage réalisées par les ASC sous l'encadrement du TPS<br>2 FARNs réalisés par les ASC sous l'encadrement du TPS<br>3 Séances de sensibilisation réalisées par les ASC sous l'encadrement du TPS<br>4 FARNs réalisés sous l'encadrement d'ONGs<br>5 Séances de sensibilisation réalisées sous l'encadrement d'ONGs<br>7 Autre, préciser	|__|
n603	Etes-vous impliqués dans ces activités ?	1 Oui<br>0 Non	|__|
n604	Si oui lesquelles ?	1 Activités de dépistage réalisées par les ASC sous l'encadrement du TPS<br>2 FARNs réalisés par les ASC sous l'encadrement du TPS<br>3 Séances de sensibilisation réalisées par les ASC sous l'encadrement du TPS<br>4 FARNs réalisés sous l'encadrement d'ONGs<br>5 Séances de sensibilisation réalisées sous l'encadrement d'ONGs<br>7 Autre, préciser	|__|

Q7 : Supervision des services de prévention et de prise en charge de la malnutrition

n701	Durant le dernier semestre, avez-vous bénéficié de séances de vérifications /Supervisions formatives et intégrées de la part de l'ECD sur le thème de la malnutrition ?	1. Oui<br>0. Non<br>8. Ne sait pas	|__|
n702	Si oui, de combien ?		|__|
n703	S'il en a eu, qui a vérifié/supervisé vos activités de nutrition ?<br><br>COCHER TOUTES LES REPONSES POSITIVES	1 Le superviseur polyvalent<br>2 Le point focal nutrition du BPS<br>3 Le point focal nutrition du BDS<br>4 Le PAM<br>5 L'UNICEF<br>6 Le PRONIANUT<br>7 Autre, préciser.	|__|
n704	Savez-vous à quand remonte la dernière activité de vérification / supervision effectuée dans votre FOSA ?VERIFIEZ DANS LE REGISTRE DE SUPERVISION	1. Oui (MOIS et ANNEE)<br>8. Ne sait pas	|__|__| /|__|__|

Q8 : Partenaire Techniques et Financiers (PTF) appuyant le volet nutrition

n801	Qui sont les PTF appuyant l'activité de détection de la malnutrition au niveau de votre centre de santé ?<br><br>COCHER LES PTF CITES SELON LA GRILLE SUIVANTE	0 Aucun<br>1 Le PAM<br>2 L'UNICEF<br>3 USAID<br>4 FHI360<br>5 Food for the Hungry (FH)<br>6 GVC<br>7 PASS<br>8 PATHFINDER<br>9 World Vision<br>10 International Med. Corps (IMC)<br>11 Caritas<br>12 Concern<br>13 Croix Rouge<br>14 Catholic Relief Services (CRS)<br>15 GIZ<br>16 MSF<br>17 MSH<br>18 Village Health Works (VHW)<br>97 Autre, préciser.<br>98 Ne sait pas.	|__||__|
n802	Qui sont les PTF appuyant l'activité de suivi de la croissance au niveau de votre centre de santé ?<br><br>COCHER LES PTF SELON LA GRILLE SUIVANTE	0 Aucun<br>1 Le PAM<br>2 L'UNICEF<br>3 USAID<br>4 FHI360<br>5 Food for the Hungry (FH)<br>6 GVC<br>7 PASS<br>8 PATHFINDER<br>9 World Vision<br>10 International Med. Corps (IMC)<br>11 Caritas<br>12 Concern<br>13 Croix Rouge<br>14 Catholic Relief Services (CRS)<br>15 GIZ<br>16 MSF<br>17 MSH<br>18 Village Health Works (VHW)<br>97 Autre, préciser<br>98 Ne sait pas.	|__||__|

n803	Qui sont les PTF appuyant l'activité de prise en charge de la malnutrition aigüe modérée au niveau de votre centre de santé ?<br><br>COCHER LES PTF SELON LA GRILLE SUIVANTE	0 Aucun<br>1 Le PAM<br>2 L'UNICEF<br>3 USAID<br>4 FHI360<br>5 Food for the Hungry (FH)<br>6 GVC<br>7 PASS<br>8 PATHFINDER<br>9 World Vision<br>10 International Med. Corps (IMC)<br>11 Caritas<br>12 Concern<br>13 Croix Rouge<br>14 Catholic Relief Services (CRS)<br>15 GIZ<br>16 MSF<br>17 MSH<br>18 Village Health Works (VHW)<br>97 Autre, préciser.<br>98 Ne sait pas.	|__||__|
n804	Qui sont les PTF appuyant l'activité de prise en charge de la malnutrition aigüe sévère au niveau de votre centre de santé ?<br><br>COCHER LES PTF SELON LA GRILLE SUIVANTE	0 Aucun<br>1 Le PAM<br>2 L'UNICEF<br>3 USAID<br>4 FHI360<br>5 Food for the Hungry (FH)<br>6 GVC<br>7 PASS<br>8 PATHFINDER<br>9 World Vision<br>10 International Med. Corps (IMC)<br>11 Caritas<br>12 Concern<br>13 Croix Rouge<br>14 Catholic Relief Services (CRS)<br>15 GIZ<br>16 MSF<br>17 MSH<br>18 Village Health Works (VHW)<br>97 Autre, préciser.<br>98 Ne sait pas.	|__||__|

n805	Qui sont les PTF appuyant l'activité de détection de la malnutrition au niveau des agents de santé communautaires ?<br><br>COCHER LES PTF SELON LA GRILLE SUIVANTE	0 Aucun<br>1 Le PAM<br>2 L'UNICEF<br>3 USAID<br>4 FHI360<br>5 Food for the Hungry (FH)<br>6 GVC<br>7 PASS<br>8 PATHFINDER<br>9 World Vision<br>10 International Med. Corps (IMC)<br>11 Caritas<br>12 Concern<br>13 Croix Rouge<br>14 Catholic Relief Services (CRS)<br>15 GIZ<br>16 MSF<br>17 MSH<br>18 Village Health Works (VHW)<br>97 Autre, préciser.<br>98 Ne sait pas.	|__||__|
n806	Qui sont les PTF appuyant l'activité de suivi de la croissance au niveau des agents de santé communautaires ?<br>COCHER LES PTF SELON LA GRILLE SUIVANTE	0 Aucun<br>1 Le PAM<br>2 L'UNICEF<br>3 USAID<br>4 FHI360<br>5 Food for the Hungry (FH)<br>6 GVC<br>7 PASS<br>8 PATHFINDER<br>9 World Vision<br>10 International Med. Corps (IMC)<br>11 Caritas<br>12 Concern<br>13 Croix Rouge<br>14 Catholic Relief Services (CRS)<br>15 GIZ<br>16 MSF<br>17 MSH<br>18 Village Health Works (VHW)<br>97 Autre, préciser.<br>98 Ne sait pas.	|__||__|

n807	Qui sont les PTF appuyant l'activité de prise en charge de la malnutrition aigüe modérée au niveau des agents de santé communautaires ?<br>COCHER LES PTF SELON LA GRILLE SUIVANTE	0 Aucun<br>1 Le PAM<br>2 L'UNICEF<br>3 USAID<br>4 FHI360<br>5 Food for the Hungry (FH)<br>6 GVC<br>7 PASS<br>8 PATHFINDER<br>9 World Vision<br>10 International Med. Corps (IMC)<br>11 Caritas<br>12 Concern<br>13 Croix Rouge<br>14 Catholic Relief Services (CRS)<br>15 GIZ<br>16 MSF<br>17 MSH<br>18 Village Health Works (VHW)<br>97 Autre, préciser.<br>98 Ne sait pas.	|__||__|

Q9 : Continuité des services de prise en charge de la malnutrition – gestion des intrants

n901	Votre CdSa-t-il manqué de carnets de santé pour réaliser correctement l'activité de suivi de la croissance durant les 6 derniers mois ?	1 Oui<br>0 Non	|__|
n902	Savez-vous pendant combien de jours ?	1. Oui (Nombre de jours)<br>998. Ne sait pas	|__|__|_|
n903	Votre CdS a-t-elle eu à interrompre les services de prise en charge de la malnutrition durant les 6 derniers mois ?<br><br>VERIFIEZ LES REGISTRES, LES FICHES DE STOCK ET LES DOSSIERS INDIVIDUELS	1 Oui, le SSN >D<br>2 Oui, le STA >F<br>3 Oui, les deux >D<br>0 Non, aucun des deux > FIN DES QUESTIONS	|__|
n904	Savez-vous pendant combien de jours votre CdS a-t-il eu à interrompre les services pour le SSN ?	1. Oui (Nombre de jours)<br>998. Ne sait pas	|_|_|_|
n905	Quelles étaient la(les) raisons de cette interruption de service ?	1 Rupture de stock<br>2 Les personnes qualifiées étaient absentes et n'ont pas pu être remplacées<br>7 Autre, préciser.	|__|
n906	Votre CdS a-t-il manqué de CSB au cours des 6 derniers mois ?	1. Oui<br>0. Non<br>9. Pas de fiches de stock ou fiches de stocks mal remplies	|__|
n907	Savez-vous pendant combien de temps votre CdS a manqué de CSB ?<br>VERIFIER RAPPORT	1. Oui (Nombre de jours)<br>998. Ne sait pas	|__|_|__|
n908	Si votre CdS a manqué de CSB, avez-vous opté pour une solution de rechange pour parer à ce problème ?	1 Oui<br>0 Non	|__|
n909	Si oui, quelle était cette solution ?<br><br>COCHER LA REPONSE QUI EST DONNEE	1 Requête auprès des ONG partenaires et don<br>2 Achat de farine enrichie sur le marché local<br>3 Commande et achat de CSB ou assimilé sur le marché extérieur (Rwanda, Tanzanie ?...)<br>4 Emprunt auprès d'autres CdS<br>5 Utilisation du stock pour femmes enceintes et allaitantes après rupture de celui des enfants<br>6 Malnutris sous protocole SSN mis sous protocole STA<br>7 Autre, préciser.	|__|
n910	Savez-vous pendant combien de jours votre CdS a-t-il eu à interrompre les services pour le STA ?	Nombre de jours.<br>998. Ne sait pas	|__|_|_|
n911	Quelles étaient la(les) raisons de cette interruption de service ?	1 Rupture de stock<br>2 Les personnes qualifiées étaient absentes et n'ont pas pu être remplacées<br>7 Autre, préciser.	|__|
n912	Votre CdS a-t-il manqué de Plumpy Nut au cours des 6 derniers mois ?	1. Oui<br>0. Non<br>9. Pas de fiches de stock ou fiches de stocks mal remplies	|__|

n913	Savez-vous pendant combien de temps votre CdS a manqué de Plumpy Nut?<br>VERIFIER RAPPORT	1. Oui (Nombre de jours)<br>998. Ne sait pas	|__|_|__|
n914	Si votre CdS a manqué de PlumpyNut, avez-vous opté pour une solution de rechange pour parer à ce problème ?	1 Oui<br>0 Non	|__|
n915	Si oui, quelle était cette solution ?<br><br>COCHER LA REPONSE QUI EST DONNEE	1 Requête auprès des ONG partenaires et don<br>2 Achat de PlumpyNut ou assimilé sur le marché local<br>3 Commande et achat de PlumpyNut ou assimilé sur le marché extérieur (Rwanda, Tanzanie ?...)<br>4 Emprunt auprès d'autres CdS<br>5 Utilisation du stock pour femmes enceintes et allaitantes après rupture de celui des enfants<br>6 Malnutris sous protocole STA mis sous protocole SSN<br>7 Autre, préciser.	|__|

Outils de vérification

RÉPUBLIQUE DU BURUNDI
MINISTÈRE DE LA SANTÉ PUBLIQUE

PROGRAMME NATIONAL INTEGRE D'ALIMENTATION ET DE NUTRITION (PRONIANUT)

RAPPORT MENSUEL DU STOCK NUTRITIONNEL

Mois/Année : ………………………… Province Sanitaire de……………………….. District sanitaire de…………………………
Nom de l'Hôpital/ Centre de Santé de ……………………………

	Lait F-100 (cartons)	Lait F-75 (cartons)	Plumpy nut (cartons)	ReSoMal (cartons)	Amoxy	Vit. A 200 UI	Vit. A 100 UI	Acide folique (5 mg)	Fer folate	Albend.	Mebend.
Entrée											
Sortie											
Etat de stock											
Entrée											
Sortie											
Etat de stock											
Entrée											
Sortie											
Etat de stock											

Module (5) Connaissances du personnel nutrition

Questionnaire V

A ADRESSER AUX TROIS **AGENTS DE SANTE LES PLUS IMPLIQUES** DANS LES ACTIVITES DE PREVENTION ET DE PRISE EN CHARGE DE LA **MALNUTRITION**.

REFEREZ-VOUS AU MANUEL DE L'ENQUETEUR POUR LE CONSENTEMENT ECLAIRE.

Identification

**Id001**	Date d'enquête	Jour/mois/année	|__|__|/|__|__|/|__|__|
**Id002**	Code de l'enquêteur		|__|__|
**id003**	**Code de la province**	**Voir codes provinces**	**|__|__|**
**id004**	**Code FOSA**	**Voir codes FOSA**	**|__|__||__|__||__|__|**

Vérification du Module (5) par le contrôleur

Nom du contrôleur	
Code du contrôleur	|_||_|
Date	|_||_|/|_||_|/|_||_|

Vérification du Module (5) par le superviseur

Nom du superviseur	
Code du superviseur	|_||_|
Date	|_||_|/|_||_|/|_||_|

Informations générales

v101	Nom et prénom de l'agent de santé		
v102	Code de l'agent de santé	Voir codes dans la grille du personnel (questionnaire G)	|__||__|
v103	Age de l'agent de santé	En années	|__|
v104	Etes-vous né dans cette province ?	1 Oui<br>0 Non<br>9. Refus de réponse	|__|
v105	Statut matrimonial	1 Célibataire<br>2 Marié(e) / Union libre<br>3 Veuf (veuve)<br>4 Divorcé(e) / séparé(e)<br>9. Refus de réponse	|__|
v106	Est-ce que vous vivez avec votre conjoint ?	1 Oui<br>0 Non<br>9. Refus de réponse	|__|
v107	Votre conjoint est-il/elle né(e) dans cette province ?	1 Oui<br>0 Non<br>9. Refus de réponse	|__|
v108	Avez-vous des enfants ?	1 Oui<br>0 Non<br>9. Refus de réponse	|__|
v109	Quel est l'âge de votre plus jeune enfant ?	En années<br>99. Refus de réponse	|__||__|
v110	Qui est votre employeur ?	1 Etat<br>2 Centre de santé<br>3 Organisation Non Gouvernementale (ONG)<br>7 Autre, préciser :<br>9. Refus de réponse	|__|
v111	Quel type de contrat avez- vous signé avec votre employeur ?	1 Permanent (public) et donnant droit à une pension (personnel sous statut)<br>2 Personnel sous contrat (Etat)<br>3 Personnel sous contrat (Centre de santé)<br>4 Permanent (Privé) et donnant droit à une pension<br>5 Contrat de courte durée (< 6 mois)<br>6 Contrat à durée déterminée (> 6 mois)<br>7 Informel (pas de contrat)<br>8 Bénévole<br>97 autre, préciser :	|__|
v112	Quel est votre diplôme le plus élevé ?	1 Niveau A3<br>2 Niveau A2<br>3 Niveau A1<br>4 Niveau Licence<br>5 Médecin généraliste<br>7 Autre, préciser :	|__|
v113	Depuis combien de temps travaillez-vous pour ce centre de santé ?	En années	|__||__|

v114	Quel est votre poste en tant que prestataire tel que défini par le Ministère de la Santé Publique et de la Lutte contre le Sida	01 Titulaire du centre de santé<br>02 Titulaire Adjoint du CDS<br>03 Chef d'équipe des services préventifs<br>04 Infirmier dans le service<br>05 Chef du service laboratoire<br>06 Technicien de laboratoire<br>07 Chef du service de promotion de la santé<br>08 Technicien de Promotion de la Santé (TPS)<br>09 Gestionnaire financier<br>10 Secrétaire<br>11 Médecin<br>12 Gestionnaire de la pharmacie<br>97 Autres	|__||__|
v115	Avez-vous été amené à changer de poste / fonction depuis votre arrivée au CdS ?	1. Oui<br>0. Non	|__|
	**<u>Formation continue</u>**		
v116	Après la fin de vos études professionnelles, avez-vous suivi une des formations FBP suivantes ?<br><br>LISEZ CHAQUE OPTION A HAUTE VOIX, POUR CHACUNE DES OPTIONS, COCHER SI L'AGENT DE SANTE REPOND OUI	1 Formation surle manuel des procédures FBP<br>2 Formation sur la grille d'évaluation de la qualité au niveau des CDS<br>3 Formation sur la base de données FBP<br>7 Autre formation en relation avec le FBP, préciser :	|__| |__| |__| |__|
v117	Après la fin de vos études professionnelles, avez-vous suivi une des formations du PRONIANUT suivantes ?<br><br>LISEZ CHAQUE OPTION A HAUTE VOIX, POUR CHACUNE DES OPTIONS, COCHER SI L'AGENT DE SANTE REPOND OUI	1Suivi nutritionnel et de croissance<br>2Dépistage et PEC de la malnutrition aigue<br>3 Formation sur les outils de collecte de données<br>4 Education Nutritionnelle et fonctionnement des FARN<br>5 PEC de malnutrition sévère avec complication<br>6 Gestion des intrants nutritionnels<br>7 Autre formation en relation avec la prévention et la prise en charge de la malnutrition, préciser :	|__| |__| |__| |__| |__| |__| |__|

v118	Après la fin de vos études professionnelles, avez-vous suivi une des formations du DPSHA suivantes ?<br><br>LISEZ CHAQUE OPTION A HAUTE VOIX, POUR CHACUNE DES OPTIONS, COCHER SI L'AGENT DE SANTE REPOND OUI	1 Formation sur la communication en santé communautaire<br>2Formation sur les aspects de santé communautaire3 Formation sur la base de données nutrition<br>4 Gestion des intrants nutritionnels<br>7 Autre formation en relation avec la promotion de la santé, préciser :	|__| |__| |__| |__| |__|
v119	Y a-t-il des besoins en formation que vous estimez nécessaires pour votre travail actuel ?	1 Oui<br>0 Non(→ R)	|__|
v120	Si oui, de quelle(s) formation(s) supplémentaire(s) auriez-vous besoin pour votre travail actuel ?<br><br>NE PAS LIRE LES OPTIONS (DEJA LUES JUSTE AVANT), ET COCHER LES REPONSES QUE L'AGENT ENONCE	1 Formation surle manuel des procédures FBP<br>2 Formation sur la grille d'évaluation de la qualité au niveau des CDS<br>3 Formation sur la base de données FBP<br>4 Suivi nutritionnel et de croissance<br>5 Dépistage et PEC de la malnutrition aigue<br>6 Formation sur les outils de collecte de données<br>07Education Nutritionnelle et fonctionnement des FARN<br>8 PEC de malnutrition sévère avec complication<br>9 Gestion des intrants nutritionnels<br><br>10 Formation sur la communication en santé communautaire<br>11 Formation sur les aspects de santé communautaire<br>12 Formation sur la base de données nutrition<br>133Gestion des intrants nutritionnels<br>97 Autre formation en relation avec le FBP, préciser<br>997 Autre formation en relation avec la prévention et la prise en charge de la malnutrition, préciser :9997Autre formation en relation avec la promotion de la santé, préciser :	|__| |__| |__| |__| |__| |__| |__| |__| |__| |__| |__| |__| |__| |__| |__| |__| |__|
	**<u>Nombre d'heures et fonctions</u>**		
v121	Combien d'heures au total avez-vous travaillé dans ce centre de santé durant les 7 derniers jours ?	Nombre d'heures [0-168]	|__||__||__|
v122	Est-ce que l'activité de promotion de la croissance fait partie de vos responsabilités ?	1 Oui<br>0 Non	|__|

v123	Avez-vous travaillé sur cette activité durant les 7 derniers jours ?	1 Oui<br>0 Non (-> U)	|__|
v124	Si oui, combien d'heures au total avez-vous travaillé sur cette activité durant les 7 derniers jours ?	Nombre d'heures [0-168]	|__||__||__|
v125	Est-ce que l'activité de prise en charge de la malnutrition aigüe modérée fait partie de vos responsabilités?	1 Oui<br>0 Non	|__|
v126	Avez-vous travaillé sur cette activité durant les 7 derniers jours ?	1 Oui<br>0 Non (-> X)	|__|
v127	Si oui, combien d'heures au total avez-vous travaillé sur cette activité durant les 7 derniers jours ?	Nombre d'heures [0-168]	|__||__||__|
v128	Est-ce que l'activité de prise en charge de la malnutrition aigüe sévère fait partie de vos responsabilités?	1 Oui<br>0 Non	|__|
v129	Avez-vous travaillé sur cette activité durant les 7 derniers jours ?	1 Oui<br>0 Non (-> AA)	|__|
v130	Si oui, combien d'heures au total avez-vous travaillé sur cette activité durant les 7 derniers jours ?	Nombre d'heures [0-168]	|__||__||__|
v131	Avez-vous d'autres responsabilités ?	1 Oui<br>0 Non	|__|
	**Salaire et compensations**		
v132	Pourriez-vous me donner approximativement le montant de votre salaire mensuel net (en FBU)	01 Moins de 50,000<br>02 Entre 50,000 et 100,000<br>03 Entre 100,000 et 200,000<br>04 Entre 200,000 et 300,000<br>05 Entre 300,000 et 400,000<br>06 Entre 400,000 et 500,000<br>07 Entre 500,000 et 600,000<br>08 Entre 600,000 et 700,000<br>09 Entre 700,000 et 1,000,000<br>10 Plus de 1,000,000<br>99 Pas de salaire	|__||__|
v133	Pendant les 12 derniers mois, avez-vous toujours reçu la totalité de votre salaire dans les délais prévus par le calendrier des paiements ?	1 Oui toujours<br>2 Non pas toujours<br>0 Jamais	|__|
v134	Avez-vous reçu la totalité de votre salaire au dernier paiement mensuel ?	1 Oui<br>0 Non	|__|
	**Supervision**		
v135	Durant le dernier semestre, avez-vous reçu des séances de vérifications /Supervisions formatives et intégréesde la part de l'ECD sur le thème de la malnutrition ?	1 Oui<br>0 Non (Passer à la section suivante)	|__|

v136	S'il y en a eu, qui a vérifié/supervisé vos activités de nutrition ?<br><br>COCHER TOUTES LES REPONSES POSITIVES	1 Le superviseur polyvalent<br>2 Le point focal nutrition du BPS<br>3 Le point focal nutrition du BDS<br>4 Le PAM<br>5 L'UNICEF<br>6 Le PRONIANUT<br>7 Autre, préciser.	|__|

LA SUITE EST A REMPLIR SUR PAPIER.

Vignette 1

Prise en charge de l'enfant 1	
HEURE AU DEBUT DE LA SIMULATION	__ __ : __ __
**Scenario:** Une maman vient vous consulter parce qu'elle trouve que Léonard, son petit enfant, ne va pas bien.	

Q1 : Quelles questions poseriez-vous à la mère?

ICI POUR CHAQUE LIGNE, L'ENQUETEUR QUI JOUE LE ROLE DE L'OBSERVATEUR COCHE « OUI » (1) LORSQUE L'AGENT DE SANTE POSE LA QUESTION. DANS LE MEME TEMPS, L'ENQUETEUR QUI JOUE LE ROLE DE LA MERE LIT L'INFORMATION SITUEE DANS LA COLONNE « INFORMATION » DE LA MEME LIGNE, AFIN DE REPONDRE A LA QUESTION DE L'AGENT. UNE FOIS LA CONSULTATION TERMINEE, L'ENQUETEUR QUI JOUE LE ROLE DE L'OBSERVATEUR COCHE « NON » (0) SI LA QUESTION N'A PAS ETE POSEE.

	Questions	Oui	Non	Information
v1101	Age de l'enfant (mois) ?	1	0	13
v1102	Sexe de l'enfant ?	1	0	M
v1103	Présence de diarrhée ?	1	0	Non, comme d'habitude
	v1103_1 Durée de la diarrhée	1	0	0
	v1103_2 Sang dans les selles ?	1	0	Non
	v1103_3 Douleurs/crampes abdominales ?	1	0	Non
v1104	Présence de toux ?	1	0	Oui
	v1104_1 Toux productive ou sèche ?	1	0	Parfois sèche, parfois productive
	v1104_2 Durée de la toux	1	0	3 jours
	v1104_3 Douleurs de gorge?	1	0	Non
	v1104_4 Difficultés à respirer ?	1	0	Non, il respire même plus vite qu'avant
v1105	Appétit ?	1	0	Il mange, mais pas autant que d'habitude
	v1105_1 L'allaitez-vous encore ?	1	0	Oui
	v1105_2 Combien de fois depuis ce matin/hier ?	1	0	Pas ce matin/2 fois
	v1105_3 Capable de boire/d'avaler ?	1	0	Il s'endort au sein
	v1105_4 Vomissements ?	1	0	Oui, parfois
	v1105_5 Quel autre aliment reçoit-il d'habitude ?	1	0	Comme les enfants de son âge
	v1105_6 A quel âge (mois) avez-vous commencé les aliments ?	1	0	5
	v1105_7 Qu'a-t-il mangé depuis hier ?	1	0	Un peu de riz deux fois
	v1105_8 Alimentation changée récemment ?	1	0	Il mange depuis 3 jours
v1106	Présence de fièvre ?	1	0	Est parfois très chaud la nuit
	v1106_1 Périodicité ?	1	0	Est parfois très chaud la nuit
	v1106_2 Sudation/frissons ?	1	0	Parfois
	v1106_3 Présence de convulsions ?	1	0	Non
	v1106_4 Altération de la conscience ?	1	0	Semble endormi souvent
v1107	Quelles maladies a-t-il déjà eu auparavant ?	1	0	Rien de spécial
v1108	A reçu des médicaments récemment?	1	0	paracétamol
v1109	Combien de médicaments ?	1	0	1 paracétamol il y a 2 jours
v1110	Porte-t-il la main à l'oreille ?	1	0	Non

Q2: Quel examen médical de l'enfant feriez-vous?

ICI POUR CHAQUE LIGNE, L'ENQUETEUR QUI JOUE LE ROLE DE L'OBSERVATEUR COCHE « OUI » (1) LORSQUE L'AGENT DE SANTE REALISE L'EXAMEN. DANS LE MEME TEMPS, L'ENQUETEUR QUI JOUE LE ROLE DE LA MERE LIT L'INFORMATION SITUEE DANS LA COLONNE « INFORMATION » DE LA MEME LIGNE, POUR DONNER LE RESULTAT DE L'EXAMEN A L'AGENT. UNE FOIS LA CONSULTATION TERMINEE, L'ENQUETEUR QUI JOUE LE ROLE DE L'OBSERVATEUR COCHE « NON » (0) SI L'EXAMEN N'A PAS ETE REALISE.

	Examens	Oui	Non	Information
v1201	Etat général de l'enfant ?	1	0	L'enfant est éveillé mais léthargique
	v1201_1 Température ?	1	0	38,6 C°
	v1201_2 Pouls ?	1	0	110 par minutes
	v1201_3 Rythme respiratoire ?	1	0	52 par minute
	v1201_4 Tirage sous-costal	1	0	Pas de tirage
v1202	Déshydratation ?	1	0	
	v1202_1 Yeux enfoncés récemment ?	1	0	Non
	v1202_2 Pli cutané ?	1	0	Non
v1203	Signes d'anémie ?	1	0	Conjonctives+lit de l'ongle pâles
v1204	Raideur de la nuque ?	1	0	Pas de raideur
v1205	Fontanelle bombée	1	0	Non
v1206	Examiner la gorge et les oreilles	1	0	La langue est pâle, oreilles pas de problèmes
v1207	Examiner rate/foie ?	1	0	Pas d'hépatomégalie ou de splénomégalie
v1208	Malnutrition ?	1	0	Enfant mince
	v1208_1 Poids ?	1	0	Poids=7,1 kilos
	v1208_2 Forme de la courbe de croissance ?	1	0	Même poids que 3 mois avant
	v1208_3 Taille ?	1	0	Taille=69,8 cm
	v1208_4 Circonférence brachiale ?	1	0	MUAC=120 mm
	v1208_5 Œdèmes aux pieds ?	1	0	Les pieds ne sont pas gonflés
	v1208_6 Z-score calculé (notez le score)	1	0	Z score : > -3 - < -2<br>Z Score calcule par AS :
v1209	Signes de rougeole ?	1	0	Pas d'éruption cutanée
v1210	Etat vaccinal ?	1	0	N'a pas encore reçu vaccin rougeole
v1211	Examens de laboratoire ? Lesquels ?	1	0	
	v1211_1Malaria test (GE ou Paracheck)	1	0	positif
	v1211_2 Hémoglobine	1	0	9.1g/dl
	v1211_3 Urine dipstick (ECBU)	1	0	négatif
	v1211_4____________________________	1	0	En attente
	v1211_5____________________________	1	0	En attente

Q3 : A quel(s) diagnostic(s) préliminaires pensez-vous ?

ICI POUR CHAQUE LIGNE, L'ENQUETEUR QUI JOUE LE ROLE DE L'OBSERVATEUR COCHE « OUI » (1) LORSQUE L'AGENT DE SANTE POSE LE DIAGNOSTIC, PUIS INDIQUE LES EXPLICATIONS DE L'AGENT DANS LA COLONNE « EXPLICATIONS DE L'AGENT ». UNE FOIS LA CONSULTATION TERMINEE, IL COCHE « NON » (0) POUR TOUS LES DIAGNOSTICS QUI N'ONT PAS ETE POSES.

	Diagnostics	Oui	Non	Explications de l'agent de santé
v1301	Malaria sévère (malaria + anémie)	1	0	
v1302	Malaria	1	0	
v1303	Anémie	1	0	
v1304	Méningite	1	0	
v1305	Malnutrition compliquée (pneumonie)	1	0	
v1306	Pneumonie	1	0	
v1307	Malnutrition modérée	1	0	
v1308	Malnutrition sévère	1	0	
v1309	Déshydratation	1	0	
v1310	Parasitose intestinale	1	0	
v1311	Diarrhée	1	0	
v1312	Manque d'appétit	1	0	
v1313	Retard de croissance	1	0	
v1399	Ne sait pas	1	0	

Q4: Quels traitements ou actions allez-vous entreprendre ?

ICI POUR CHAQUE LIGNE, L'ENQUETEUR QUI JOUE LE ROLE DE L'OBSERVATEUR COCHE « OUI » (1) LORSQUE L'AGENT DE SANTE ENTREPREND LE TRAITEMENT OU L'ACTION, PUIS INDIQUE LES EXPLICATIONS DE L'AGENT DANS LA COLONNE « EXPLICATIONS DE L'AGENT ». UNE FOIS LA CONSULTATION TERMINEE, IL COCHE « NON » (0) POUR TOUS LES TRAITEMENTS OU ACTIONS QUI N'ONT PAS ETE ENTREPRIS.

	Traitements ou actions	Oui	Non	Explications de l'agent de santé
v1401	Traitement anti-malarique ? Lequel ?	1	0	
v1402	Traitement antibiotique ? Lequel ? Pour quelle indication ?	1	0	
v1403	Traitement antipyrétique ? Lequel ?	1	0	
v1404	Prescription fer+acide folique	1	0	
v1405	Prescription de SRO ?	1	0	
v1406	Prescription d'antiparasitaire ?	1	0	
v1407	Envoyer l'enfant au SSN ?	1	0	
v1408	Envoyer l'enfant au STA ?	1	0	
v1409	Référer l'enfant au SST ?	1	0	
v1410	Renvoyer l'enfant à la maison et donner une date de visite de suivi ?	1	0	
v1411	Garder l'enfant en observation dans la FOSA ?	1	0	
v1412	Compléter les vaccinations ?	1	0	
v1413	Donner des conseils diététiques ? Lesquels ?	1	0	
v1414	Donner des conseils sur l'hygiène ? Lesquels ?	1	0	
v1415	Expliquer à la maman l'utilisation des médicaments ?	1	0	
v1416	Expliquer à la maman les signes de danger ?	1	0	
v1417	Expliquer à la maman l'urgence d'aller au SST et ce qui va s'y passer ?	1	0	
v1418	Discuter avec la maman les actions possibles et lui demander son avis ?	1	0	
v1499	Ne sait pas	1	0	

**Heure à la fin de la simulation**	**__ __ : __ __**

Q5. Commentaires de l'enquêteur

v1501	Durée de la consultation ?	minutes	|__|__|
v1502	Diriez-vous de cette consultation qu'elle était (en termes thérapeutiques)	1.De très bonne qualité<br>2. De bonne qualité<br>3. De qualité acceptable<br>4. De qualité faible<br>5. De très faible qualité	|__|
v1503	Diriez-vous de cette consultation qu'elle était (en termes de conseils)	1.De très bonne qualité<br>2. De bonne qualité<br>3. De qualité acceptable<br>4. De qualité faible<br>5. De très faible qualité	|__|
v1504	Quelle était l'attitude de l'agent de santé par rapport au parent et à son enfant ? (écoute, respect, communication, etc.)	1.De très bonne qualité<br>2. De bonne qualité<br>3. De qualité acceptable<br>4. De qualité faible<br>5. De très faible qualité	|__|
v1505	Quels aspects de la consultation seraient améliorables?	--<br>--<br>--<br>--	

Vignette 2

Prise en charge de l'enfant 2	
HEURE AU DEBUT DE LA SIMULATION	__ __ : __ __
**Scenario:** Une maman vient vous consulter parce qu'elle trouve que son petit Manassé n'est pas aussi fort que l'enfant de la voisine pourtant né la même semaine que le sien	

Q1 : Quelles questions poseriez-vous à la mère?

ICI POUR CHAQUE LIGNE, L'ENQUETEUR QUI JOUE LE ROLE DE L'OBSERVATEUR COCHE « OUI » (1) LORSQUE L'AGENT DE SANTE POSE LA QUESTION. DANS LE MEME TEMPS, L'ENQUETEUR QUI JOUE LE ROLE DE LA MERE LIT L'INFORMATION SITUEE DANS LA COLONNE « INFORMATION » DE LA MEME LIGNE, AFIN DE REPONDRE A LA QUESTION DE L'AGENT. UNE FOIS LA CONSULTATION TERMINEE, L'ENQUETEUR QUI JOUE LE ROLE DE L'OBSERVATEUR COCHE « NON » (0) SI LA QUESTION N'A PAS ETE POSEE.

	Questions	Oui	Non	Information
v2101	Age de l'enfant (mois) ?	1	0	9
v2102	Sexe de l'enfant ?	1	0	M
v2103	Présence de diarrhée ?	1	0	Parfois, a fait une grosse diarrhée il y a 2 semaines
	v2103_1 Durée de la diarrhée	1	0	Pas actuellement
	v2103_2 Sang dans les selles ?	1	0	Non
	v2103_3 Douleurs/crampes abdominales ?	1	0	Parfois
v2104	Présence de toux ?	1	0	Parfois
	v2104_1 Toux productive ou sèche ?	1	0	Cela dépend
	v2104_2 Durée de la toux	1	0	Pas actuellement
	v2104_3 Douleurs de gorge?	1	0	Non
	v2104_4 Difficultés à respirer ?	1	0	Non
v2105	Appétit ?	1	0	Comme tous les enfants de son âge
	v2105_1 L'allaitez-vous encore ?	1	0	Oui
	v2105_2 Combien de fois depuis ce matin/hier ?	1	0	1 fois/2 fois
	v2105_3 Capable de boire/d'avaler ?	1	0	oui
	v2105_4 Vomissements ?	1	0	Oui, parfois
	v2105_5 Quel autre aliment reçoit-il d'habitude ?	1	0	Comme tous les enfants de son âge
	v2105_6 A quel âge (mois) avez-vous commencé les aliments ?	1	0	5
	v2105_7 Qu'a-t-il mangé depuis hier ?	1	0	1 bouillie de manioc le matin 1 bouillie de maïs l'après-midi
	Alimentation changée récemment ?	1	0	Non
v2106	Présence de fièvre ?	1	0	Se plaint de la chaleur
	v2106_1 Périodicité ?	1	0	Ne sait pas
	v2106_2 Sudation/frissons ?	1	0	Parfois
	v2106_3 Présence de convulsions ?	1	0	Non
	v2106_4 Altération de la conscience ?	1	0	Un peu calme depuis sa diarrhée
v2107	Quelles maladies a-t-il déjà eu auparavant ?	1	0	Rougeole à 5 mois, bronchite à 7 mois
v2108	A reçu des médicaments récemment?	1	0	Non
v2109	Combien de médicaments ?	1	0	Non
v2110	Porte-t-il la main à l'oreille ?	1	0	Non

Q2: Quel examen médical de l'enfant feriez-vous?

ICI POUR CHAQUE LIGNE, L'ENQUETEUR QUI JOUE LE ROLE DE L'OBSERVATEUR COCHE « OUI » (1) LORSQUE L'AGENT DE SANTE REALISE L'EXAMEN. DANS LE MEME TEMPS, L'ENQUETEUR QUI JOUE LE ROLE DE LA MERE LIT L'INFORMATION SITUEE DANS LA COLONNE « INFORMATION » DE LA MEME LIGNE, POUR DONNER LE RESULTAT DE L'EXAMEN A L'AGENT. UNE FOIS LA CONSULTATION TERMINEE, L'ENQUETEUR QUI JOUE LE ROLE DE L'OBSERVATEUR COCHE « NON » (0) SI L'EXAMEN N'A PAS ETE REALISE.

	Examens	Oui	Non	Information
v2201	Etat général de l'enfant ?	1	0	L'enfant est éveillé
	v2201_1 Température ?	1	0	37,6 C°
	v2201_2 Pouls ?	1	0	120 par minutes
	v2201_3 Rythme respiratoire ?	1	0	35 par minute
	v2201_4 Tirage sous-costal	1	0	Pas de tirage
v2202	Déshydratation ?	1	0	
	v2202_1 Yeux enfoncés récemment ?	1	0	Non
	v2202_2 Pli cutané ?	1	0	Non
v2203	Signes d'anémie ?	1	0	Conjonctives + lit de l'ongle pâles
v2204	Raideur de la nuque ?	1	0	Pas de raideur
v2205	Fontanelle bombée	1	0	Non
v2206	Examiner la gorge et les oreilles	1	0	La langue est pâle, oreilles pas de problèmes
v2207	Examiner rate/foie ?	1	0	Pas d'hépatomégalie ou de splénomégalie
v2208	Malnutrition ?	1	0	Enfant mince
	v2208_1 Poids ?	1	0	Poids=6,9 kilos
	v2208_2 Forme de la courbe de croissance ?	1	0	Pas disponible
	v2208_3 Taille ?	1	0	Taille=65,0 cm
	v2208_4 Circonférence brachiale ?	1	0	MUAC=112 mm
	v2208_5 Œdèmes aux pieds ?	1	0	Petit godet au niveau dorsal de chaque pied
	v2208_6 Z-score calculé (notez le score)	1	0	Z score : >-1 - <median<br>Z Score calcule par AS :
v2209	Signes de rougeole ?	1	0	Pas d'éruption cutanée
v2210	Etat vaccinal ?	1	0	A jour
v2211	Examens de laboratoire ? Lesquels ?	1	0	
	V2211_1 Malaria test (GE ou Paracheck)	1	0	négatif
	V2211_2 Hémoglobine	1	0	7.3g/dl
	V2211_3 Urine dipstick (ECBU)	1	0	négatif
	V2211_4____________________________	1	0	En attente
	V2211_5____________________________	1	0	En attente

Q3 : A quel(s) diagnostic(s) préliminaires pensez-vous ?

ICI POUR CHAQUE LIGNE, L'ENQUETEUR QUI JOUE LE ROLE DE L'OBSERVATEUR COCHE « OUI » (1) LORSQUE L'AGENT DE SANTE POSE LE DIAGNOSTIC, PUIS INDIQUE LES EXPLICATIONS DE L'AGENT DANS LA COLONNE « EXPLICATIONS DE L'AGENT ». UNE FOIS LA CONSULTATION TERMINEE, IL COCHE « NON » (0) POUR TOUS LES DIAGNOSTICS QUI N'ONT PAS ETE POSES.

	Diagnostics	Oui	Non	Explications de l'agent de santé
v2301	Malaria sévère (malaria + anémie)	1	0	
v2302	Malaria	1	0	
v2303	Anémie	1	0	
v2304	Méningite	1	0	
v2305	Malnutrition compliquée (pneumonie)	1	0	
v2306	Pneumonie	1	0	
v2307	Malnutrition modérée	1	0	
v2308	Malnutrition sévère	1	0	
v2309	Déshydratation	1	0	
v2310	Parasitose intestinale	1	0	
v2311	Diarrhée	1	0	
v2312	Manque d'appétit	1	0	
v2313	Retard de croissance	1	0	
v2399	Ne sait pas	1	0	

Q4 : Quels traitements ou actions allez-vous entreprendre ?

ICI POUR CHAQUE LIGNE, L'ENQUETEUR QUI JOUE LE ROLE DE L'OBSERVATEUR COCHE « OUI » (1) LORSQUE L'AGENT DE SANTE ENTREPREND LE TRAITEMENT OU L'ACTION, PUIS INDIQUE LES EXPLICATIONS DE L'AGENT DANS LA COLONNE « EXPLICATIONS DE L'AGENT ». UNE FOIS LA CONSULTATION TERMINEE, IL COCHE « NON » (0) POUR TOUS LES TRAITEMENTS OU ACTIONS QUI N'ONT PAS ETE ENTREPRIS.

	Traitements ou actions	Oui	Non	Explications de l'agent de santé
v2401	Traitement anti-malarique ? Lequel ?	1	0	
v2402	Traitement antibiotique ? Lequel ? Pour quelle indication ?	1	0	
v2403	Traitement antipyrétique ? Lequel ?	1	0	
v2404	Prescription fer+acide folique	1	0	
v2405	Prescription de SRO ?	1	0	
v2406	Prescription d'antiparasitaire ?	1	0	
v2407	Envoyer l'enfant au SSN ?	1	0	
v2408	Envoyer l'enfant au STA ?	1	0	
v2409	Référer l'enfant au SST ?	1	0	
v2410	Renvoyer l'enfant à la maison et donner une date de visite de suivi ?	1	0	
v2411	Garder l'enfant en observation dans la FOSA ?	1	0	
v2412	Compléter les vaccinations ?	1	0	
v2413	Donner des conseils diététiques ? Lesquels ?	1	0	-<br>-<br>-<br>-
v2414	Donner des conseils sur l'hygiène ? Lesquels ?	1	0	-<br>-<br>-
v2415	Expliquer à la maman l'utilisation des médicaments ?	1	0	
v2416	Expliquer à la maman les signes de danger ?	1	0	
v2417	Expliquer à la maman l'urgence d'aller au SST et ce qui va s'y passer ?	1	0	
v2418	Discuter avec la maman les actions possibles et lui demander son avis ?	1	0	
v2499	Ne sait pas	1	0	

**Heure à la fin de la simulation**	__ __ : __ __

Q5. Commentaires de l'enquêteur

v2501	Durée de la consultation ?	minutes	|__|__|
v2502	Diriez-vous de cette consultation qu'elle était (en termes thérapeutiques)	1.De très bonne qualité<br>2. De bonne qualité<br>3. De qualité acceptable<br>4. De qualité faible<br>5. De très faible qualité	|__|
v2503	Diriez-vous de cette consultation qu'elle était (en termes de conseils)	1.De très bonne qualité<br>2. De bonne qualité<br>3. De qualité acceptable<br>4. De qualité faible<br>5. De très faible qualité	|__|
v2504	Quelle était l'attitude de l'agent de santé par rapport au parent et à son enfant ? (écoute, respect, communication, etc.)	1.De très bonne qualité<br>2. De bonne qualité<br>3. De qualité acceptable<br>4. De qualité faible<br>5. De très faible qualité	|__|
v2505	Quels aspects de la consultation seraient améliorables?	--<br>--<br>--<br>--	

Vignette 3

Prise en charge de l'enfant 3	
**HEURE AU DEBUT DE LA SIMULATION**	__ __ : __ __
Scenario: Une maman emmène sa petite Mélanie à la FOSA pour recevoir la troisième dose des vaccins polio et DTC+Hib+HepB.	

Q1 : Quelles questions poseriez-vous à la mère?

ICI POUR CHAQUE LIGNE, L'ENQUETEUR QUI JOUE LE ROLE DE L'OBSERVATEUR COCHE « OUI » (1) LORSQUE L'AGENT DE SANTE POSE LA QUESTION. DANS LE MEME TEMPS, L'ENQUETEUR QUI JOUE LE ROLE DE LA MERE LIT L'INFORMATION SITUEE DANS LA COLONNE « INFORMATION » DE LA MEME LIGNE, AFIN DE REPONDRE A LA QUESTION DE L'AGENT. UNE FOIS LA CONSULTATION TERMINEE, L'ENQUETEUR QUI JOUE LE ROLE DE L'OBSERVATEUR COCHE « NON » (0) SI LA QUESTION N'A PAS ETE POSEE.

	Questions	Oui	Non	Information
v3101	Age de l'enfant (mois) ?	1	0	4,5
v3102	Sexe de l'enfant ?	1	0	F
v3103	Présence de diarrhée ?	1	0	Parfois
	v3103_1 Durée de la diarrhée	1	0	Pas actuellement
	v3103_2 Sang dans les selles ?	1	0	Non
	v3103_3 Douleurs/crampes abdominales ?	1	0	Parfois
v3104	Présence de toux ?	1	0	Non
	v3104_1Toux productive ou sèche ?	1	0	-
	v3104_2 Durée de la toux	1	0	-
	v3104_3 Douleurs de gorge?	1	0	-
	v3104_4 Difficultés à respirer ?	1	0	Non
v3105	Appétit ?	1	0	Pas de problème
	v3105_1 L'allaitez-vous encore ?	1	0	Évidemment !
	v3105_2 Combien de fois depuis ce matin/hier ?	1	0	Beaucoup de fois (à la demande)
	v3105_3Capable de boire/d'avaler ?	1	0	
	v3105_4 Vomissements ?	1	0	Régurgite parfois
	v3105_5 Quel autre aliment reçoit-il d'habitude ?	1	0	Seulement le sein
	v3105_6 A quel âge (mois) avez-vous commencé les aliments ?	1	0	-
	v3105_7 Qu'a-t-il mangé depuis hier ?	1	0	Seulement le sein
	v3105_8 Alimentation changée récemment ?			Non
v3106	Présence de fièvre ?	1	0	Parfois
	v3106_1 Périodicité ?	1	0	Ne sait pas
	v3106_2 Sudation/frissons ?	1	0	Non
	v3106_3 Présence de convulsions ?	1	0	Non
	v3106_4 Altération de la conscience ?	1	0	Non
v3107	Quelles maladies a-t-il déjà eu auparavant ?	1	0	Aucune
v3108	A reçu des médicaments récemment?	1	0	Non
v3109	Combien de médicaments ?	1	0	Non
v3110	Porte-t-il la main à l'oreille ?	1	0	Non

Q2: Quel examen médical de l'enfant feriez-vous?

ICI POUR CHAQUE LIGNE, L'ENQUETEUR QUI JOUE LE ROLE DE L'OBSERVATEUR COCHE « OUI » (1) LORSQUE L'AGENT DE SANTE REALISE L'EXAMEN. DANS LE MEME TEMPS, L'ENQUETEUR QUI JOUE LE ROLE DE LA MERE LIT L'INFORMATION SITUEE DANS LA COLONNE « INFORMATION » DE LA MEME LIGNE, POUR DONNER LE RESULTAT DE L'EXAMEN A L'AGENT. UNE FOIS LA CONSULTATION TERMINEE, L'ENQUETEUR QUI JOUE LE ROLE DE L'OBSERVATEUR COCHE « NON » (0) SI L'EXAMEN N'A PAS ETE REALISE.

	Examens	Oui	Non	Information
v3201	Etat général de l'enfant ?	1	0	L'enfant est éveillé
v3202	Température ?	1	0	37,6 C°
	v3202_1 Pouls ?	1	0	110 par minutes
	v3202_2 Rythme respiratoire ?	1	0	33 par minute
	v3202_3 Tirage sous-costal	1	0	Pas de tirage
v3203	Déshydratation ?	1	0	
	v3203_1 Yeux enfoncés récemment ?	1	0	Non
	v3203_2 Pli cutané ?	1	0	Non
v3204	Signes d'anémie ?	1	0	Conjonctives + lit de l'ongle pâles
v3205	Raideur de la nuque ?	1	0	Pas de raideur
v3206	Fontanelle bombée	1	0	Non
v3207	Examiner la gorge et les oreilles	1	0	Pas de problèmes
v3208	Examiner rate/foie ?	1	0	Pas d'hépatomégalie ou de splénomégalie
v3209	Malnutrition ?	1	0	Enfant potelé
	v3209_1 Poids ?	1	0	Poids=4,8 kilos
	v3209_2 Forme de la courbe de croissance ?	1	0	A perdu 300 g par rapport au mois passé
	v3209_3 Taille ?	1	0	Taille=57,8 cm
	v3209_4 Circonférence brachiale ?	1	0	MUAC=114 mm
	v3209_5 Œdèmes aux pieds ?	1	0	Pieds semble gonflés (pas de godet)
	v3209_6 Z-score calculé (notez le score)	1	0	Z score : > -2 - <-1<br>Z Score calcule par AS :
v3210	Signes de rougeole ?	1	0	Pas d'éruption cutanée
v3211	Etat vaccinal ?	1	0	A jour
v3212	Examens de laboratoire ? Lesquels ?			
	V3212_1Malaria test (GE ou Paracheck)	1	0	Négatif
	V3212_2 Hémoglobine	1	0	10.1g/dl
	V3212_3 Urine dipstick (ECBU)	1	0	Négatif
	V3212_4______________________	1	0	En attente
	V3212_5______________________	1	0	En attente

Q3 : A quel(s) diagnostic(s) préliminaires pensez-vous ?

ICI POUR CHAQUE LIGNE, L'ENQUETEUR QUI JOUE LE ROLE DE L'OBSERVATEUR COCHE « OUI » (1) LORSQUE L'AGENT DE SANTE POSE LE DIAGNOSTIC, PUIS INDIQUE LES EXPLICATIONS DE L'AGENT DANS LA COLONNE « EXPLICATIONS DE L'AGENT ». UNE FOIS LA CONSULTATION TERMINEE, IL COCHE « NON » (0) POUR TOUS LES DIAGNOSTICS QUI N'ONT PAS ETE POSES.

	Diagnostics	Oui	Non	Explications de l'agent de santé
v3301	Malaria sévère (malaria + anémie)	1	0	
v3302	Malaria	1	0	
v3303	Anémie	1	0	
v3304	Méningite	1	0	
v3305	Malnutrition compliquée (pneumonie)	1	0	
v3306	Pneumonie	1	0	
v3307	Malnutrition modérée	1	0	
v3308	Malnutrition sévère	1	0	
v3309	Déshydratation	1	0	
v3310	Parasitose intestinale	1	0	
v3311	Diarrhée	1	0	
v3312	Manque d'appétit	1	0	
v3313	Retard de croissance	1	0	
v3399	Ne sait pas	1	0	

Q4 : Quels traitements ou actions allez-vous entreprendre ?

ICI POUR CHAQUE LIGNE, L'ENQUETEUR QUI JOUE LE ROLE DE L'OBSERVATEUR COCHE « OUI » (1) LORSQUE L'AGENT DE SANTE ENTREPREND LE TRAITEMENT OU L'ACTION, PUIS INDIQUE LES EXPLICATIONS DE L'AGENT DANS LA COLONNE « EXPLICATIONS DE L'AGENT ». UNE FOIS LA CONSULTATION TERMINEE, IL COCHE « NON » (0) POUR TOUS LES TRAITEMENTS OU ACTIONS QUI N'ONT PAS ETE ENTREPRIS.

	Traitements ou actions	Oui	Non	Explications de l'agent de santé
v3401	Traitement anti-malarique ? Lequel ?	1	0	
v3402	Traitement antibiotique ? Lequel ? Pour quelle indication ?	1	0	
v3403	Traitement antipyrétique ? Lequel ?	1	0	
v3404	Prescription fer+acide folique	1	0	
v3405	Prescription de SRO ?	1	0	
v3406	Prescription d'antiparasitaire ?	1	0	
v3407	Envoyer l'enfant au SSN ?	1	0	
v3408	Envoyer l'enfant au STA ?	1	0	
v3409	Référer l'enfant au SST ?	1	0	
v3410	Renvoyer l'enfant à la maison et donner une date de visite de suivi ?	1	0	
v3411	Garder l'enfant en observation dans la FOSA ?	1	0	
v3412	Compléter les vaccinations ?	1	0	
v3413	Donner des conseils diététiques ? Lesquels ?	1	0	-<br>-<br>-<br>-
v3414	Donner des conseils sur l'hygiène ? Lesquels ?	1	0	-<br>-<br>-
v3415	Expliquer à la maman l'utilisation des médicaments ?	1	0	
v3416	Expliquer à la maman les signes de danger ?	1	0	
v3417	Expliquer à la maman l'urgence d'aller au SST et ce qui va s'y passer ?	1	0	
v3418	Discuter avec la maman les actions possibles et lui demander son avis ?	1	0	
v3499	Ne sait pas	1	0	

**Heure à la fin de la simulation**	__ __ : __ __

Q5. Commentaires de l'enquêteur

v3501	Durée de la consultation ?	minutes	|__|__|
v3502	Diriez-vous de cette consultation qu'elle était (en termes thérapeutiques)	1.De très bonne qualité<br>2. De bonne qualité<br>3. De qualité acceptable<br>4. De qualité faible<br>5. De très faible qualité	|__|
v3503	Diriez-vous de cette consultation qu'elle était (en termes de conseils)	1.De très bonne qualité<br>2. De bonne qualité<br>3. De qualité acceptable<br>4. De qualité faible<br>5. De très faible qualité	|__|
v3504	Quelle était l'attitude de l'agent de santé par rapport au parent et à son enfant ? (écoute, respect, communication, etc.)	1.De très bonne qualité<br>2. De bonne qualité<br>3. De qualité acceptable<br>4. De qualité faible<br>5. De très faible qualité	|__|
v3505	Quels aspects de la consultation seraient améliorables?	--<br>--<br>--	

Demandes de consentement éclairé, par type de questionnaire

Note : ces textes seront traduits en Kirundi.

Questionnaire G, adressé aux gestionnaires des centres de santé :

Bonjour, Monsieur/Madame,

Je m'appelle ____ , je travaille pour le Ministère de la Santé Publique et de la Lutte contre le Sida. Le Ministère nous a demandé de mener une enquête portant sur les services de prévention et de prise en charge de la malnutrition dans les formations sanitaires au Burundi. L'idée est d'étudier le niveau de l'offre de soins au niveau des formations sanitaires, en vue de faire des propositions pour une amélioration, mais aussi d'évaluer si le financement basé sur la performance des activités de prévention et de prise en charge de la malnutrition est une bonne idée pour améliorer la qualité de ce type de services.

Pour faire cette évaluation, 90 centres de santé avec SSN et STA ont été sélectionnés aléatoirement : votre centre en fait partie. Le FBP Nutrition sera introduit à l'automne 2014 dans la moitié de ces centres (groupe de traitement) ; les autres centres constitueront le groupe témoin ; ces derniers recevront une compensation financière équivalente à la moyenne reçue dans les centres du groupe de traitement recevant le FBP Nutrition. On réalise une enquête maintenant, avant que le FBP Nutrition soit introduit, afin d'évaluer quelle est la situation de base, quelle est la qualité des activités liées à la malnutrition aujourd'hui. On repassera dans un an puis dans deux ans afin d'évaluer quelle est l'évolution de la qualité des services dans les groupes de traitement et les groupes témoins, et de statuer sur l'efficacité du FBP Nutrition. Les résultats de cette évaluation au niveau du Burundi seront communiqués fin 2014 pour la présente enquête, puis fin 2015 et fin 2016 pour les enquêtes d'évaluation du FBP Nutrition. En outre, les informations obtenues lors de cette enquête permettront de faire une évaluation individualisée de votre centre de santé (d'ici l'automne 2014), et de vous proposer des solutions pour améliorer la qualité de vos services en termes de gestion et de nutrition.

Afin d'évaluer la qualité des activités liées à la malnutrition, nous souhaitons, dans chacun des 90 centres de santé, nous entretenir avec le personnel, leurs responsables et quelques enfants-patients et leurs accompagnants. Nous souhaitons également observer des consultations et consulter les registres SSN et STA.

Votre centre de santé fait partie des 90 centres sélectionnés pour cette étude d'impact sur le FBP Nutrition. La présente étape consiste à faire un entretien avec vous comme responsable de ce centre de santé, sur les aspects organisationnels généraux du centre de santé.

Vous êtes libre d'accepter ou de refuser de participer à l'étude. Aussi, si vous acceptez de participer, vous êtes libre de refuser certaines questions ou d'arrêter l'entretien à tout moment. De plus, vous être libre de modifier ou ajuster vos réponses à tout moment. Si vous acceptez de prendre part à cet exercice, la confidentialité de vos propos est garantie. Le nom du centre et le vôtre, ainsi que toutes les données personnelles n'apparaîtront sur aucun document. Ils seront remplacés par des codes dont seul le responsable de l'étude a la clé. Vos réponses à cette enquête ne pourront en aucun cas être utilisées pour remettre en cause votre emploi dans la fonction publique.

Si vous avez de questions sur cette enquête vous pouvez toujours contacter Docteur Novat Twungubumwe.

Acceptez-vous de répondre à mes questions ?
1. Je marque mon accord pour participer à l'enquête /___/
2. Je suis désolé de ne pas pouvoir participer à l'enquête. /___/ *Arrêt de l'interview*

/___/ *Je déclare être âgé d'au moins 18 ans et avoir reçu les informations nécessaires pour cette étude*

Signature ou empreintes digitales: /____________________/

Nom et signature du chercheur : /____/

Questionnaire N, adressé aux responsables des services de nutrition :

Bonjour, Madame /Monsieur,

Je m'appelle ______________________________ , je travaille pour le Ministère de la Santé Publique et de la Lutte contre le Sida. Le Ministère nous a demandé de mener une enquête portant sur les services de prévention et de prise en charge de la malnutrition dans les formations sanitaires au Burundi. L'idée est d'étudier le niveau de l'offre de soins au niveau des formations sanitaires, en vue de faire des propositions pour une amélioration, mais aussi d'évaluer si le financement basé sur la performance des activités de prévention et de prise en charge de la malnutrition est une bonne idée pour améliorer la qualité de ce type de services.

Pour faire cette évaluation, 90 centres de santé avec SSN et STA ont été sélectionnés aléatoirement : votre centre en fait partie. Le FBP Nutrition sera introduit à l'automne 2014 dans la moitié de ces centres (groupe de traitement) ; les autres centres constitueront le groupe témoin ; ces derniers recevront une compensation financière équivalente à la moyenne reçue dans les centres du groupe de traitement recevant le FBP Nutrition. On réalise une enquête maintenant, avant que le FBP Nutrition soit introduit, afin d'évaluer quelle est la situation de base, quelle est la qualité des activités liées à la malnutrition aujourd'hui. On repassera dans un an puis dans deux ans afin d'évaluer quelle est l'évolution de la qualité des services dans les groupes de traitement et les groupes témoins, et de statuer sur l'efficacité du FBP Nutrition. Les résultats de cette évaluation au niveau du Burundi seront communiqués fin 2014 pour la présente enquête, puis fin 2015 et fin 2016 pour les enquêtes d'évaluation du FBP Nutrition. En outre, les informations obtenues lors de cette enquête permettront de faire une évaluation individualisée de votre centre de santé (d'ici l'automne 2014), et de vous proposer des solutions pour améliorer la qualité de vos services en termes de gestion et de nutrition.

Afin d'évaluer la qualité des activités liées à la malnutrition, nous souhaitons, dans chacun des 90 centres de santé, nous entretenir avec le personnel de ce centre de santé et leurs responsables. Nous souhaitons également observer des consultations et consulter les registres SSN et STA.

Votre centre de santé fait partie des 90 centres sélectionnés pour cette étude d'impact sur le FBP Nutrition. La présente étape consiste à faire un entretien avec vous comme responsable des services nutritionnels de ce centre de santé, sur les aspects organisationnels de ces services.

Vous êtes libre d'accepter ou de refuser de participer à l'étude. Aussi, si vous acceptez de participer, vous êtes libre de refuser certaines questions ou d'arrêter l'entretien à tout moment. De plus, vous être libre de modifier ou ajuster vos réponses à tout moment. Si vous acceptez de prendre part à cet exercice, la confidentialité de vos propos est garantie. Le nom du centre et le vôtre, ainsi que toutes les données personnelles n'apparaîtront sur aucun document. Ils seront remplacés par des codes dont seul le responsable de l'étude a la clé. Vos réponses à cette enquête ne pourront en aucun cas être utilisées pour remettre en cause votre emploi dans la fonction publique.

Si vous avez de questions sur cette enquête vous pouvez toujours contacter Docteur Novat Twungubumwe .<u>Acceptez-vous de répondre à mes questions ?</u>

1. Je marque mon accord pour participer à l'enquête /___/
2. Je suis désolé de ne pas pouvoir participer à l'enquête. /___/ Arrêt de l'interview

/___/ Je déclare être âgé d'au moins 18 ans et avoir reçu les informations nécessaires pour cette étude

Signature ou empreintes digitales: /___________________/

Nom et signature du chercheur : /____/

Grilles d'observation C et P, adressées aux trois agents de santé les plus impliqués dans la nutrition :

Bonjour, Madame /Monsieur,

Je m'appelle ______________________________ , je travaille pour le Ministère de la Santé Publique et de la Lutte contre le Sida. Le Ministère nous a demandé de mener une enquête portant sur les services de prévention et de prise en charge de la malnutrition dans les formations sanitaires au Burundi. L'idée est d'étudier le niveau de l'offre de soins au niveau des formations sanitaires, en vue de faire des propositions pour une amélioration, mais aussi d'évaluer si le financement basé sur la performance des activités de prévention et de prise en charge de la malnutrition est une bonne idée pour améliorer la qualité de ce type de services.

Pour faire cette évaluation, 90 centres de santé avec SSN et STA ont été sélectionnés aléatoirement : votre centre en fait partie. Le FBP Nutrition sera introduit à l'automne 2014 dans la moitié de ces centres (groupe de traitement) ; les autres centres constitueront le groupe témoin ; ces derniers recevront une compensation financière équivalente à la moyenne reçue dans les centres du groupe de traitement recevant le FBP Nutrition. On réalise une enquête maintenant, avant que le FBP Nutrition soit introduit, afin d'évaluer quelle est la situation de base, quelle est la qualité des activités liées à la malnutrition aujourd'hui. On repassera dans un an puis dans deux ans afin d'évaluer quelle est l'évolution de la qualité des services dans les groupes de traitement et les groupes témoins, et de statuer sur l'efficacité du FBP Nutrition. Les résultats de cette évaluation au niveau du Burundi seront communiqués fin 2014 pour la présente enquête, puis fin 2015 et fin 2016 pour les enquêtes d'évaluation du FBP Nutrition. En outre, les informations obtenues lors de cette enquête permettront de faire une évaluation individualisée de votre centre de santé (d'ici l'automne 2014), et de vous proposer des solutions pour améliorer la qualité de vos services en termes de gestion et de nutrition.

Afin d'évaluer la qualité des activités liées à la malnutrition, nous souhaitons, dans chacun des 90 centres de santé, nous entretenir avec le personnel, leurs responsables et quelques enfants-patients et leurs accompagnants. Nous souhaitons également observer des consultations et consulter les registres SSN et STA.

Votre centre de santé fait partie des 90 centres sélectionnés pour cette étude d'impact sur le FBP Nutrition. La présente étape consiste à observer un total de dix consultations (préventives et curatives) des trois agents de santé les plus impliqués dans les services nutritionnels de ce centre de santé. La grille permettra d'évaluer vos connaissances mais surtout votre pratique en consultation.

Vous êtes libre d'accepter ou de refuser de participer à l'étude. Aussi, si vous acceptez de participer, vous êtes libre de demander à arrêter l'observation à tout moment. Si vous refusez de prendre part à cet exercice, cela n'aura aucun impact négatif sur vous. Si vous acceptez de prendre part à cet exercice vous êtes libre de modifier ou ajuster vos réponses à tout moment et la confidentialité des informations collectées est garantie. Le nom du centre et le vôtre, ainsi que les données personnelles n'apparaîtront sur aucun document. Ils seront remplacés par des codes dont seul le responsable de l'étude a la clé. Vos réponses à cette enquête ne pourront en aucun cas être utilisées pour remettre en cause votre emploi dans la fonction publique.

Si vous avez de questions sur cette enquête vous pouvez toujours contacter Docteur Novat Twungubumwe .<u>Acceptez-vous de prendre part à l'enquête et d'être observé en consultation ?</u>

1.Je marque mon accord pour participer à l'enquête et être observé en consultation /___/
2.Je suis désolé ne pas pouvoir participer à l'enquête et être observé en consultation /___/ Arrêt de l'observation

/___/ Je déclare être âgé d'au moins 18 ans et avoir reçu les informations nécessaires pour cette étude

Signature ou empreintes digitales: /______________/

Nom et signature du chercheur : /____/

Grilles d'observation C et P, impliquant dix patients (enfants) et leurs accompagnants:

Bonjour, Madame /Monsieur,

Je m'appelle ____________________________________ je travaille pour le Ministère de la Santé Publique et de la Lutte contre le Sida. Le Ministère nous a demandé de mener une enquête portant sur les services de prévention et de prise en charge de la malnutrition dans les formations sanitaires au Burundi. L'idée est d'étudier le niveau de l'offre de soins au niveau des formations sanitaires, en vue de faire des propositions pour une amélioration, mais aussi d'évaluer si le financement basé sur la performance des activités de prévention et de prise en charge de la malnutrition est une bonne idée pour améliorer la qualité de ce type de services.

Pour faire cette évaluation, 90 centres de santé ont été sélectionnés aléatoirement : votre centre en fait partie. On réalise une enquête maintenant, afin d'évaluer quelle est la situation de base, quelle est la qualité des activités liées à la malnutrition aujourd'hui.

Afin d'évaluer la qualité des activités liées à la malnutrition, nous souhaitons, nous entretenir avec quelques enfants-patients et leurs accompagnants. On aimerait par la présente étape observer votre consultation.

En tant qu'accompagnant de patient sélectionné pour l'observation, vous êtes libre d'accepter ou de refuser de participer à l'étude. Aussi, si vous acceptez de participer, vous serez libre de demander à arrêter l'observation à tout moment. De plus, vous être libre de modifier ou ajuster vos réponses à tout moment. Votre acceptation ou refus de participer n'aura aucun effet négatif sur la qualité des soins que vous allez recevoir. Aucune information obtenue dans cette enquête ne sera rattachée à votre personne ou au centre de santé. Le nom du centre, le vôtre et celui de l'enfant que vous accompagnez, ainsi que toutes les données personnelles n'apparaîtront sur aucun document. Ils seront remplacés par des codes dont seul le responsable de l'étude a la clé. Les informations obtenues lors de cette enquête ne pourront en aucun cas être utilisées pour remettre en cause votre accès, à vous et vos proches, aux soins de santé ici.

Si vous avez de questions sur cette enquête vous pouvez toujours contacter Docteur Novat Twungubumwe.

Acceptez-vous de prendre part à l'enquête et d'être observé en consultation ?

1.Je marque mon accord pour participer à l'enquête et être observé en consultation /___/

2.Je suis désolé ne pas pouvoir participer à l'enquête et être observé en consultation /___/ Arrêt de l'observation

/___/ Je déclare être âgé d'au moins 18 ans et avoir reçu les informations nécessaires pour cette étude

Signature ou empreintes digitales: /______________/

Nom et signature du chercheur : /____/

Questionnaire V, adressé aux trois agents de santé les plus impliqués dans la nutrition :

Bonjour, Madame /Monsieur,

Je m'appelle ____ , je travaille pour le Ministère de la Santé Publique et de la Lutte contre le Sida. Le Ministère nous a demandé de mener une enquête portant sur les services de prévention et de prise en charge de la malnutrition dans les formations sanitaires au Burundi. L'idée est d'étudier le niveau de l'offre de soins au niveau des formations sanitaires, en vue de faire des propositions pour une amélioration, mais aussi d'évaluer si le financement basé sur la performance des activités de prévention et de prise en charge de la malnutrition est une bonne idée pour améliorer la qualité de ce type de services.

Pour faire cette évaluation, 90 centres de santé avec SSN et STA ont été sélectionnés aléatoirement : votre centre en fait partie. Le FBP Nutrition sera introduit à l'automne 2014 dans la moitié de ces centres (groupe de traitement) ; les autres centres constitueront le groupe témoin ; ces derniers recevront une compensation financière équivalente à la moyenne reçue dans les centres du groupe de traitement recevant le FBP Nutrition. On réalise une enquête maintenant, avant que le FBP Nutrition soit introduit, afin d'évaluer quelle est la situation de base, quelle est la qualité des activités liées à la malnutrition aujourd'hui. On repassera dans un an puis dans deux ans afin d'évaluer quelle est l'évolution de la qualité des services dans les groupes de traitement et les groupes témoins, et de statuer sur l'efficacité du FBP Nutrition. Les résultats de cette évaluation au niveau du Burundi seront communiqués fin 2014 pour la présente enquête, puis fin 2015 et fin 2016 pour les enquêtes d'évaluation du FBP Nutrition. En outre, les informations obtenues lors de cette enquête permettront de faire une évaluation individualisée de votre centre de santé (d'ici l'automne 2014), et de vous proposer des solutions pour améliorer la qualité de vos services en termes de gestion et de nutrition.

Afin d'évaluer la qualité des activités liées à la malnutrition, nous souhaitons, dans chacun des 90 centres de santé, nous entretenir avec le personnel, leurs responsables et quelques enfants-patients et leurs accompagnants. Nous souhaitons également observer des consultations et consulter les registres SSN et STA.

Votre centre de santé fait partie des 90 centres sélectionnés pour cette étude d'impact sur le FBP Nutrition. La présente étape consiste à faire un entretien avec les trois agents de santé les plus impliqués dans les services nutritionnels de ce centre de santé. Les questions portent sur votre formation, votre implication dans les services nutritionnels, ainsi que sur vos connaissances.

Vous êtes libre d'accepter ou de refuser de participer à l'étude. Aussi, si vous acceptez de participer, vous êtes libre de refuser certaines questions ou d'arrêter l'entretien à tout moment. De plus, vous être libre de modifier ou ajuster vos réponses à tout moment. Si vous refusez de prendre part à cet exercice, cela n'aura aucun impact négatif sur vous. Si vous acceptez de prendre part à cet exercice, la confidentialité de vos propos est garantie. Le nom du centre et le vôtre, ainsi que les données personnelles n'apparaîtront sur aucun document. Ils seront remplacés par des codes dont seul le responsable de l'étude a la clé. Vos réponses à cette enquête ne pourront en aucun cas être utilisées pour remettre en cause votre emploi dans la fonction publique.

Si vous avez de questions sur cette enquête vous pouvez toujours contacter Docteur Novat Twungubumwe.

Acceptez-vous de répondre à mes questions ?
1. Je marque mon accord pour participer à l'enquête /___/
2. Je suis désolé de ne pas pouvoir participer à l'enquête. /___/ *Arrêt de l'interview*

/___/ Je déclare être âgé d'au moins 18 ans et avoir reçu les informations nécessaires pour cette étude

Signature ou empreintes digitales: /_______________/

Nom et signature du chercheur : /____/

Questionnaire S, adressé à dix accompagnants d'enfants, à la sortie :

Bonjour, Madame /Monsieur,

Je m'appelle ____ je travaille pour le Ministère de la Santé Publique et de la Lutte contre le Sida. Le Ministère nous a demandé de mener une enquête portant sur les services de prévention et de prise en charge de la malnutrition dans les formations sanitaires au Burundi. L'idée est d'étudier le niveau de l'offre de soins au niveau des formations sanitaires, en vue de faire des propositions pour une amélioration, mais aussi d'évaluer si le financement basé sur la performance des activités de prévention et de prise en charge de la malnutrition est une bonne idée pour améliorer la qualité de ce type de services.

Pour faire cette évaluation, 90 centres de santé avec SSN et STA ont été sélectionnés aléatoirement : votre centre en fait partie. Le FBP Nutrition sera introduit à l'automne 2014 dans la moitié de ces centres (groupe de traitement) ; les autres centres constitueront le groupe témoin ; ces derniers recevront une compensation financière équivalente à la moyenne reçue dans les centres du groupe de traitement recevant le FBP Nutrition. On réalise une enquête maintenant, avant que le FBP Nutrition soit introduit, afin d'évaluer quelle est la situation de base, quelle est la qualité des activités liées à la malnutrition aujourd'hui. On repassera dans un an puis dans deux ans afin d'évaluer quelle est l'évolution de la qualité des services dans les groupes de traitement et les groupes témoins, et de statuer sur l'efficacité du FBP Nutrition. Les résultats de cette évaluation au niveau du Burundi seront communiqués fin 2014 pour la présente enquête, puis fin 2015 et fin 2016 pour les enquêtes d'évaluation du FBP Nutrition. En outre, les informations obtenues lors de cette enquête permettront de faire une évaluation individualisée de votre centre de santé (d'ici l'automne 2014), et de vous proposer des solutions pour améliorer la qualité de vos services en termes de gestion et de nutrition.

Afin d'évaluer la qualité des activités liées à la malnutrition, nous souhaitons, dans chacun des 90 centres de santé, nous entretenir avec le personnel, leurs responsables et quelques enfants-patients et leurs accompagnants. Nous souhaitons également observer des consultations et consulter les registres SSN et STA.

Ce centre de santé fait partie des 90 centres sélectionnés pour cette étude d'impact sur le FBP Nutrition. La présente étape consiste à faire un entretien avec une dizaine d'accompagnants d'enfants sortant d'une consultation, comme vous. Nous aimerions avoir votre appréciation sur les différents services qui vous sont offerts dans ce centre de santé de façon à nous permettre de rechercher les voies et moyens d'améliorer la qualité des soins. Nous souhaiterions également prendre les mesures anthropométriques de l'enfant que vous accompagnez ; en outre, si nous voyons que l'enfant souffre de malnutrition, nous vous en informerons et vous suggérerons les mesures à prendre immédiatement .

Vous êtes libre d'accepter ou de refuser de participer à l'étude. Aussi, si vous acceptez de participer, vous serez libre de refuser certaines questions ou d'arrêter l'entretien à tout moment. De plus, vous être libre de modifier ou ajuster vos réponses à tout moment. Votre acceptation ou refus de participer à cet entretien n'aura aucun effet négatif sur vous, sur votre accès aux services de santé ou sur la qualité des soins que vous recevez ici, ni sur ce centre de santé. La confidentialité de vos propos est garantie. Aucune information obtenue dans cette enquête ne sera rattachée à votre personne ou au centre de santé. Le nom du centre, le vôtre et celui de l'enfant que vous accompagnez, ainsi que les données personnelles n'apparaîtront sur aucun document. Ils seront remplacés par des codes dont seul le responsable de l'étude a la clé. Vos réponses à cette enquête ne pourront en aucun cas être utilisées pour remettre en cause votre accès, à vous et vos proches, aux soins de santé ici.

Si vous avez de questions sur cette enquête vous pouvez toujours contacter Docteur Novat Twungubumwe.

<u>Acceptez-vous de répondre à mes questions ?</u>
1. Je marque mon accord pour participer à l'enquête /___/
2. Je suis désolé de ne pas pouvoir participer à l'enquête. /___/ *Arrêt de l'interview*

/___/ *Je déclare être âgé d'au moins 18 ans et avoir reçu les informations nécessaires pour cette étude*

Signature ou empreintes digitales: /_____________/

Nom et signature du chercheur : /____/
