# Supplementary material for: Impact of the extension of a performance-based financing scheme to nutrition services in Burundi on malnutrition prevention and management among children below five: A cluster-randomized control trial
Source: PLoS One. 2020 Sep 18;15(9):e0239036. doi: 10.1371/journal.pone.0239036 (PMC7500612; doi:10.1371/journal.pone.0239036)
Supplement: S7 File — Rapport des enquêtes de référence au niveau des centres de santé et des ménages ». Source: Authors. (PDF) [file pone.0239036.s009.pdf]

Institut de Médecine Tropicale
d'Anvers

Banque Mondiale

République du Burundi
Ministère de la Santé et de Lutte
contre le SIDA

FBP Nutrition au Burundi

Rapport des enquêtes de référence
au niveau des centres de santé et des ménages

C. Korachais
K. de Polnay
S. Nkurunziza
M. Nimpagaritse
P. Kolsteren
B. Meessen

Octobre 2015

Ce rapport présente les résultats des enquêtes de référence relatives à l'étude d'impact du financement basé sur la performance (FBP) appliqué à la nutrition au Burundi. Ces enquêtes ont été réalisées sur la période septembre 2014 à janvier 2015. Le FBP appliqué à la nutrition a pour objectif d'améliorer la prévention et la prise en charge de la malnutrition dans le système de santé burundais et a été mis en place dans le groupe d'intervention à partir de janvier 2015. L'évaluation d'impact finale est prévue pour fin 2016.

Ce rapport a été validé par la Comité de Pilotage du Ministère de la Santé Publique et de la Lutte contre le SIDA le 9 décembre 2015.

Remerciements

Cette étude d'impact du financement basé sur la performance appliqué à la nutrition au Burundi est financée par la Banque Mondiale, à travers le *Health Results Innovation Trust Fund (HRITF)*. Les auteurs de ce rapport tiennent à remercier les membres de la Banque Mondiale et du Ministère de la Santé Publique et de la Lutte contre le SIDA impliqués dans ce projet, et particulièrement ceux de la Cellule Technique chargée du FBP et ceux du PRONIANUT, pour leur collaboration, notamment dans l'élaboration du design de l'étude. Nous souhaitons également exprimer notre gratitude envers l'INSP et l'ISTEEBU qui ont mis en place et mené à bien les enquêtes respectivement auprès des centres de santé et des ménages. En outre, les membres de notre équipe Ulises Huerta, Elodie Macouillard, Léonard Ntakarutimana, Désiré Munezero et Jacqueline Manisabwe méritent tout autant d'être salués pour leur aide remarquable durant la préparation et la mise en place des enquêtes. Enfin, nous souhaiterions remercier les membres du Comité Technique et du Comité de Pilotage pour leur suivi et leurs commentaires contructifs.

Comment citer ce rapport

Korachais, C., K. de Polnay, S. Nkurunziza, M. Nimpagaritse, P. Kolsteren et B. Meessen. 2015. FBP Nutrition au Burundi : Rapport des enquêtes de référence au niveau des centres de santé et des ménages. Institut de Médecine Tropicale d'Anvers, Belgique.

Table des matières

Résumé exécutif 4
Contexte du projet 4
Méthodologie 6
Résultats de l'enquête de référence 9
Validité de l'étude 12
Recommandations 14
Acronymes et abréviations 18
Index des tableaux 20
Index des figures 22
1. Vue d'ensemble du projet d'étude 23
1.1 Contexte 23
Objectifs du Millénaire pour le Développement (OMDs) au Burundi : où en sommes-nous et quels sont les principaux défis ? 23
Histoire du FBP au Burundi 23
Malnutrition: un des principaux problèmes de santé publique au Burundi 24
1.2 Intervention évaluée 25
Description de l'intervention 25
Mise en œuvre 27
1.3 Objectifs de l'étude 28
Théorie du changement 28
Questions et objectifs de l'évaluation d'impact 30
Perspectives politiques 31
Structure du rapport 31
2. Méthodologie 32
2.1 Design de l'étude 32
2.2 Randomisation et taille d'échantillon 32
Au niveau de la communauté 32
Au niveau des centres de santé 33
2.3 Variables pour l'analyse des données 33
Au niveau de la communauté 33
Au niveau des centres de santé 33
2.4 Instruments de collecte et d'assurance qualité 34
2.4.1 Enquête au niveau des centres de santé 34
2.4.2 Enquête au niveau de la communauté 44
2.5 Stockage, gestion et politique d'accès aux données 48
3. Représentativité de l'échantillon et validité externe de l'étude 49
3.1 Représentativité géographique 49
Représentativité nationale 49
Représentativité du secteur santé 49
3.2 Comparaison entre les enquêtes de référence et les statistiques nationales existantes 50
Enquête au niveau des centres de santé 50
Enquête au niveau des ménages 51
4. Résultats de l'enquête de référence 54
4.1 Enquête au niveau des centres de santé 54

Aspects organisationnels des centres de santé 54
Evaluation des dossiers médicaux individuels issus des services liés à la malnutrition 60
Qualité des services de santé 68
Connaissances et compétences des agents de santé 83
4.2 Enquête au niveau des ménages 109
Caractéristiques des ménages 109
Caractéristiques des enfants et de leurs responsables 111
Synthèse 119
5. Validité interne de l'étude 120
5.1 Puissance de calcul ex-post 120
Puissance de calcul ex-post au niveau des centres de santé 120
Puissance de calcul ex-post au niveau des ménages 120
5.2 Obstacles rencontrés et implications pour la validité de l'étude 121
Enquête au niveau des centres de santé 121
Enquête auprès des ménages 124
5.3 Equilibre de l'échantillon: résultats des tests de différence 124
Enquête au niveau des centres de santé 124
Enquête auprès des ménages 125
6. Discussion 126
6.1 Enquête sur au niveau des centres de santé 126
Performance des services de nutrition pour les de MAM (SSN) 126
Performance des services de nutrition pour les cas de MAS (STA) 126
Aspects organisationnels des services de nutrition (SSN et STA) 127
Fourniture des soins de santé des enfants - Performance et connaissances des agents de santé 127
Fourniture des soins de santé pour les enfants – Appréciation par les patients 135
6.2 Enquête auprès des ménages 136
7. Recommandations 138
7.1 A l'égard de l'équipe d'évaluation 138
Améliorer les questionnaires 138
Préparer les enquêtes de suivi 138
Documenter le processus de mise en œuvre de l'intervention 138
Plaider pour un bon remplissage et archivage des fiches individuelles de suivi et des registres 139
Organiser des formations personnalisées sur demande des CDS 139
7.2 A l'égard des parties prenantes dans la mise en œuvre de l'intervention 140
Disponibilité des intrants 140
Formations des agents de santé 140
Achever la mise en œuvre de l'intervention FBP Nutrition 140
7.3 A l'égard du MSPLS de manière plus générale 141
Amélioration de la performance des agents de santé 141
Amélioration du fonctionnement quotidien des services de pédiatrie et de nutrition 143
7.4 A l'égard des commanditaires de l'étude - MSPLS et Banque Mondiale 143
Préparation des enquêtes de suivi 143
Recommandations majeures 143
8. Références bibliographiques 145
9. Annexe. Comparaison des principales statistiques parmi les groupes de contrôle et d'intervention 148

Résumé exécutif

Contexte du projet

Santé et financement de la santé au Burundi

Le Burundi est un petit pays enclavé se trouvant à l'Est de l'Afrique Centrale. Avec un Produit National Brut par habitant estimé en 2014 à 770 dollars internationaux (Banque Mondiale, 2015), le Burundi est parmi les pays les plus pauvres du monde. La population burundaise est d'environ 10.6 millions avec 421 habitants par km carré. Toutefois il importe de signaler que la majorité de cette même population vit en milieu rural. Depuis l'indépendance, le Burundi a connu de continuels soubresauts de conflits et d'instabilité, et depuis 1993 jusqu'à récemment, le pays était en état de guerre civile. Par conséquent, l'état de santé de la population est parmi les plus mauvais d'Afrique. Entre 2006 et 2011, le taux de mortalité des enfants de moins de 5 ans était estimé à 96 décès pour 1000 naissances, tandis que le taux de mortalité maternelle était de 500 décès pour 100 000 naissances (EDS, 2010).

Financement de la santé. En 2006, face à des besoins en santé croissants, le gouvernement a mis en place l'exemption des paiements directs pour les enfants de moins de 5 ans, les soins liés à la grossesse et les accouchements. Cette décision s'est heurtée à de nombreux défis, notamment de financement. Parallèlement, le Burundi expérimentait dans trois provinces une nouvelle stratégie de financement de la santé, le financement basé sur la performance (FBP), dont l'objectif était d'améliorer le système de santé. Après ces expériences pilotes positives, le FBP s'est étendu à l'échelle du pays en avril 2010. Une décision importante prise par le gouvernement fut celle de fusionner le FBP et la gratuité des soins.

Malnutrition. Au Burundi, la malnutrition est une barrière évidente à la réalisation des objectifs du millénaire pour le développement liés à la santé. En effet, 58% des enfants de moins de 5 ans souffrent de malnutrition chronique (EDS, 2010). En 2010, le Ministère chargé de la Santé du Burundi a développé un protocole proposant un plan de traitement et de suivi de la malnutrition aigüe pour les enfants de moins de cinq ans. Aujourd'hui, seulement un tiers des centres de santé et la moitié des hôpitaux offrent des services de prise en charge de la malnutrition aigüe (UNICEF, communication personnelle, 2013).

Introduction de la nutrition dans le FBP. Avec le soutien de la Banque Mondiale, le gouvernement Burundais renforce ce système, en introduisant des indicateurs liés à la nutrition dans le FBP existant : c'est une opportunité à la fois pour améliorer le statut nutritionnel des enfants et pour réaliser une analyse rigoureuse des forces et des faiblesses du FBP. En effet, l'intégration des services de nutrition dans le FBP est un défi dans la mesure où actuellement, la mise en œuvre des services de nutrition est fortement dépendante d'organisations externes comme l'UNICEF ou le Programme Alimentaire Mondial (PAM) qui fournissent les intrants nutritionnels. De plus, les services de nutrition sont négligés de par un manque de connaissances et de savoir-faire du personnel de santé et un manque de supervision des activités de nutrition. Aussi, l'introduction du FBP devrait permettre de réduire ces obstacles.

Description de l'intervention FBP Nutrition

L'objectif de l'introduction des indicateurs liés à la nutrition dans le plan du FBP existant est de contribuer à l'intégration des services nutritionnels dans la stratégie nationale de santé. L'intervention FBP Nutrition consiste à introduire des incitations à trois niveaux, à la fois sur le volet préventif et curatif, tous se focalisant sur les enfants de moins de 5 ans (cf. Tableau 1). Une grille qualitative spécifique à la nutrition est prise en compte dans le calcul des bonus. Le système de vérification est à peu près le même que pour les autres indicateurs FBP au Burundi.

Tableau 1. Indicateurs conçus dans le cadre de l'intervention du FBP Nutrition

Niveau Communautaire	Les agents de santé communautaires reçoivent des bonus pour<br>(1) le dépistage et la référence vers les centres de santé nutritionnels pour les cas de malnutrition aigüe,<br>(2) l'organisation de séances de sensibilisation pour la promotion de régimes alimentaires sains et de bons comportements nutritionnels, et<br>(3) l'organisation de cours de cuisine.
Niveau Centre de Santé	Les bonus seront donnés pour<br>(1) le dépistage de malnutrition aigüe chez les enfants et la bonne prise en charge des cas de malnutrition aigüe (selon le type de centre de santé*), et<br>(2) pour la promotion et le suivi de la croissance.
Niveau Hospitalier	Les bonus seront donnés pour<br>(1) la bonne prise en charge des cas sévères de malnutrition aigüe avec complications médicales, et<br>(2) le nombre de journées d'hospitalisation liées à la malnutrition aigüe.

Source: Note Technique (MSPLS 2013). Note:*En effet, il existe plusieurs sortes de centres de santé : la plupart des centres au Burundi ne fournissent pas de services pour les cas de malnutrition aigüe, certains fournissent des services de prise en charge des cas de malnutrition aigüe modérée, certains pour les cas de malnutrition aigüe sévère, et d'autres pour les cas de malnutrition aigüe modérée et sévère.

Cette intervention a démarré en Janvier 2015 aux niveaux des hôpitaux et des centres de santé. Tous les hôpitaux du Burundi disposant de services de stabilisation des cas de malnutrition aigüe sévère avec complications (SST) sont concernés. Au niveau centre de santé, seuls ceux du groupe d'intervention sont concernés. Parmi les 90 centres de santé nutritionnels[1] sélectionnés pour l'étude, la moitié, soit 45, sont dans le groupe d'intervention ; l'autre moitié reçoit des compensations financières équivalentes sous forme de dotations. Par ailleurs, les centres de santé ne disposant pas de services de prise en charge de la malnutrition aigüe mais référant les cas de malnutrition aigüe vers les 45 centres du groupe d'intervention sont également éligibles à un financement lié à leur performance dans leurs activités de dépistage et référence de la malnutrition ainsi que de suivi de la croissance ; les centres de santé

[1] Un centre de santé est dit « nutritionnel » s'il délivre un paquet complet de services de prise en charge de la malnutrition aigüe, c'est-à-dire un service de supplémentation nutritionnelle (SSN) et un service de thérapeutique ambulatoire (STA), selon le protocole de 2010. Cependant, le protocole de 2014, publié après l'enquête de référence dans les centres de santé, recommande que les services SSN doivent désormais être mis en œuvre au niveau de la communauté, et pas dans les centres de santé.

référant vers les 45 centres du groupe de contrôle reçoivent quant à eux une compensation financière équivalente.

Le niveau communautaire du FBP Nutrition devait démarrer au cours de l'été 2015, mais certains retards sont attendus compte tenu de l'instabilité politique actuelle. Au préalable, les agents de santé communautaires seront regroupés de façon formelle en Groupements d'Agent de Santé Communautaire (GASC), puis formés aux différents indicateurs du FBP Nutrition. Seuls les GASC référant vers les 45 centres de santé nutritionnels du groupe d'intervention seront éligibles ; les GASC référant vers les autres centres ne recevront pas de compensation financière pour des raisons de contraintes budgétaires.

Figure 1. Mise en œuvre du FBP Nutrition et de la compensation

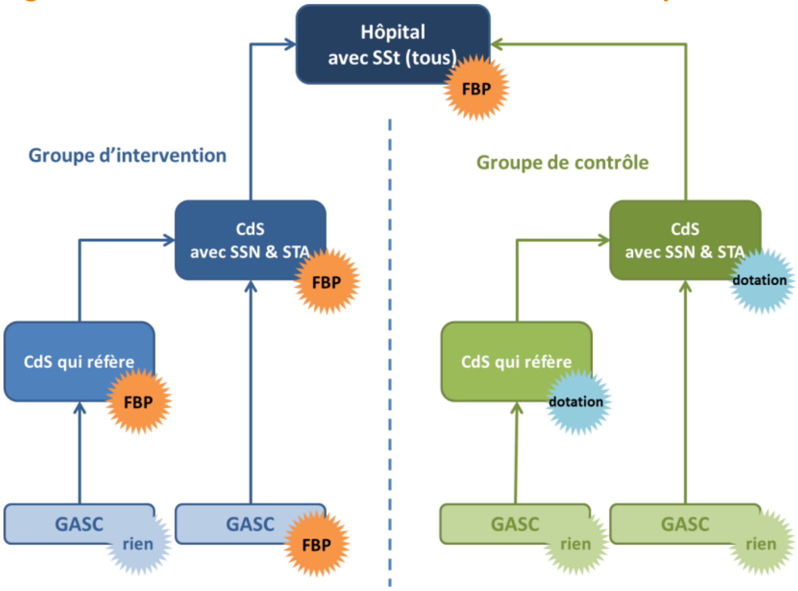

Objectifs de cette évaluation d'impact

L'objectif principal de l'évaluation d'impact est d'évaluer l'impact de l'introduction des indicateurs nutrition dans le système FBP, sur les taux de malnutrition aigüe et chronique, mesurés au niveau de la communauté, ainsi que sur la performance des services de prévention et prise en charge de la malnutrition, à travers principalement les taux de guérison de la malnutrition aigüe, mesurés au niveau des centres de santé nutritionnels.

Méthodologie

Design de l'étude et échantillonnage

Design de l'étude. Cette étude suit un design d'essais randomisés contrôlés par grappes (ou Cluster-RCT) avec le centre de santé comme premier niveau d'échantillonnage et les sous-collines (unité administrative au Burundi) comme second niveau.

Sélection des centres de santé et allocation de l'intervention. Les 90 centres de santé nutritionnels de l'étude ont été sélectionnés aléatoirement parmi les 193 centres de ce type existant au Burundi fin 2013. Ces 90 centres ont été appariés selon différents critères, puis un atelier public a eu lieu en

décembre 214, où les centres de santé étaient invités, paire par paire, à tirer au sort le statut qui sera le leur tout au long de l'étude : « intervention » ou « contrôle ».

Echantillonnage au niveau de la communauté. La taille de l'échantillon est calculée sur base de la plus petite différence dans le résultat principal qui peut être considéré comme significative dans le groupe d'intervention comparé au groupe de contrôle - ici, on considère une réduction du taux de malnutrition aigüe de 25% (compte tenu d'un taux de malnutrition aigüe parmi ces enfants de 10% selon les EDS 2010, on considère une réduction de 2.5 points de pourcentage). Selon la formule de Hayes et Bennett, 1999, pour une erreur de type alpha de 5%, une erreur de type beta de 20% et une corrélation intra-grappes de 0.25, le nombre total d'enfants de 6-23 mois et leurs ménages à enquêter pour chaque enquête (de référence et de suivi) est de 6,480, soit 72 enfants et leurs ménages par centre de santé inclus dans l'étude.

Echantillonnage au niveau des centres de santé. Afin de mesurer la performance des services nutritionnels des centres de santé (et notamment de calculer les taux de guérison), 12 fiches individuelles de suivi sont retranscrites par type de service (SSN ou STA) et par centre de santé. Ce nombre a été calculé sur base de la plus petite différence dans le résultat principal qui peut être considéré comme significative dans le groupe d'intervention comparé au groupe de contrôle - ici, on considère une hausse du taux de guérison de 80% à 90%, avec une erreur de type alpha de 5%, une erreur de type beta de 20%, et une corrélation intra-grappes de 0.15 (selon la formule de Hayes et Bennett, 1999).

Instruments de collecte au niveau des centres de santé

Différents instruments de collecte ont été administrés au niveau des centres de santé.

Fiches individuelles de suivi. Dans chaque centre de santé, a été effectuée une retranscription anonymisée de 12 fiches individuelles de suivi sélectionnées de façon aléatoire parmi les fiches de tous les enfants de moins de cinq ans ayant participé à un programme de service de supplémentation nutritionnelle (SSN) au cours des six derniers mois (en date de septembre 2014). L'objectif principal est d'évaluer les indicateurs de performance des SSN au Burundi ; un objectif secondaire est d'évaluer la qualité de la documentation relative aux patients. Les données collectées permettent de calculer les taux de guérison, la durée moyenne des traitements, et autres indicateurs de performance du SSN, afin de pouvoir répondre à la question principale de l'étude. Les données anthropométriques à l'admission et à la sortie du programme sont collectées afin d'évaluer si les critères d'admission et de sortie (pour guérison ou référence) sont respectés. Dans le cas où les fiches individuelles étaient absentes ou mal remplies, l'enquêteur devait chercher l'information dans les registres du service. La même procédure a été suivie pour la retranscription de 12 fiches individuelles d'enfants de moins de cinq ans ayant participé à un programme de service thérapeutique ambulatoire (STA).

Aspects organisationnels. Afin de collecter des informations sur les aspects organisationnels du centre de santé et des services de nutrition, deux questionnaires indépendants ont été administrés auprès du titulaire du centre de santé et du responsable du service de nutrition. L'objectif est d'utiliser ces données comme facteurs confondants dans les analyses statistiques. Les informations collectées

incluent des informations générales sur le centre de santé (organisation et infrastructure, informations sanitaires, gestion du centre, ressources humaines, partenaires techniques et financiers) et des informations générales sur les activités préventives et curatives des services de nutrition (organisation de ces services, ressources humaines, locaux utilisés pour le stockage des intrants, organisation du système concernant les références, les agents de santé communautaires et les supervisions, partenaires techniques et financiers, gestion des intrants thérapeutiques et nutritionnels) .

Qualité des services de santé. La qualité des services de santé est évaluée à partir de deux instruments : les observations de consultations et les entretiens à la sortie. Dans chaque centre de santé, **six observations de consultations curatives** d'enfants âgés de 6-59 mois ont été effectuées. L'objectif est d'évaluer la qualité des consultations, et plus spécifiquement en relation avec le respect du manuel de prise en charge intégrée des maladies de l'enfant (PCIME, WHO, 2014) en place au Burundi, et relativement à l'évaluation nutritionnelle de l'enfant. Les enquêteurs observant ces consultations étaient formés et, au cours des consultations, ont rempli manuellement des grilles d'observations contenant des listes de questions pour l'anamnèse et de points d'observation pour les examens physiques, les diagnostics, et les traitements. A la fin de la consultation, des questions ouvertes permettaient à l'enquêteur d'évaluer la qualité de la communication et les conseils fournis par l'agent de santé à l'accompagnant de l'enfant. Suite à cette consultation, il était demandé aux enfants et leurs accompagnants de répondre à un **entretien à la sortie**, administré par un autre enquêteur. Le premier objectif de ce questionnaire était d'obtenir des informations de la part des accompagnants sur leur opinion sur le contenu et la qualité de la consultation, et d'évaluer leur compréhension de l'information qui leur était donnée. Le second objectif était de documenter les mesures anthropométriques des enfants prises par les agents du centre de santé, puis de répéter ces mesures par les enquêteurs eux-mêmes.

Connaissances et savoir-faire des agents de santé. Les connaissances et compétences des agents de santé sont évaluées à partir de **trois vignettes cliniques** (consultations factices), proposées aux deux agents de santé ayant été observés en consultation durant la journée de travail. L'introduction à la vignette était très brève, par exemple « une maman vient vous consulter parce qu'elle trouve que Léonard, son petit enfant, ne va pas bien ». Ensuite, l'agent de santé était observé de la même manière qu'en consultation, avec une grille d'observation contenant des listes de questions d'anamnèse, d'examens physiques, de diagnostics et de traitements. Pour chaque question ou examen réalisé, l'enquêteur donnait la réponse, par exemple, si l'agent demandait si l'enfant avait la toux, l'enquêteur donnait la réponse donnée dans la vignette. Si bien qu'à la fin, il était possible pour l'agent de santé de déterminer le diagnostic du patient ainsi que de proposer un traitement.

Instruments de collecte au niveau de la communauté

Un seul questionnaire a été utilisé au niveau de l'enquête ménage. Il est composé de différentes sections, portant sur différentes thématiques et administrées à différents membres.

Caractéristiques générales des ménages. L'objectif de cette section est d'évaluer le statut socio-économique des ménages. Le questionnaire est basé sur les parties relatives des EDS et PMS (EDS, 2010; MSPLS, 2014) et est administré au chef de ménage ou à son conjoint.

Sécurité alimentaire des ménages. Les questions utilisées pour évaluer la sécurité alimentaire sont basées sur les questions génériques HFIAS d'échelle de l'accès déterminant l'insécurité alimentaire des ménages pour la mesure de l'accès alimentaire des ménages (cf. Coates, Swindale, & Bilinsky, 2007 ; projet FANTA). Cela comporte neuf questions de survenance qui présentent un niveau croissant de gravité de l'insécurité alimentaire (accès) et neuf questions de « fréquence-de-survenance » qui sont posées ensuite pour déterminer combien de fois est survenue cette situation.

Soins et alimentation apportés à l'enfant. Les questionnaires sont une combinaison de ceux du manuel de l'OMS pour évaluer les pratiques d'alimentation du nourrisson et du jeune enfant (WHO, 2010) et de ceux conçus par l'unité de Santé et Nutrition de l'Enfant de l'IMT d'Anvers.

Etat nutritionnel de l'enfant. Les mesures anthropométriques collectées à travers ce questionnaire permettent d'évaluer l'état nutritionnel de l'enfant. Cela permet d'évaluer les taux de prévalence de malnutrition aigüe et chronique parme les enfants de 6-23 mois dans la communauté. Couplées avec les données de l'enquête finale, ces données permettront de dire si le FBP Nutrition a un impact sur les taux de malnutrition dans la communauté.

Résultats de l'enquête de référence

Enquête au niveau des centres de santé

L'enquête s'est déroulée du 21 septembre au 7 octobre 2014.

Performance des services SSN et STA. Au total, 971 dossiers individuels de suivi issus des services SSN et 963 des services STA ont été retranscrits. Le premier constat est une mauvaise documentation : mauvais archivage des fiches individuelles de suivi, remplissage des fiches non systématique en ce qui concerne les données anthropométriques à l'admission et à la sortie, le traitement, le résultat du programme, etc. Ceci rend difficile l'évaluation de la performance des services SSN et STA. Néanmoins, les indicateurs de performance calculés pour les deux programmes STA et SSN ont atteint la plupart des recommandations nationales, notamment en termes de taux de guérison, mais pas en termes de gain de poids moyen quotidien et de durée de séjour dans le programme. Cela étant, 10% des cas reportés comme guéris dans le service SSN avaient encore un z-score de rapport poids pour taille inférieur à -2 (indiquant en fait une non-guérison).

Qualité du diagnostic. Parmi les dossiers individuels issus du SSN, environ 20% ont reçu un mauvais diagnostic : les patients étaient dans un service de prise en charge de la malnutrition aigüe modérée (MAM ; service SSN) alors que, selon les données anthropométriques renseignées sur la fiche, ils auraient dû recevoir un diagnostic de malnutrition aigüe sévère (MAS) avec une référence au service STA. En outre, parmi les 514 évaluations des enfants observés en consultation et à la sortie le jour de l'enquête, 51 étaient des cas de MAM (selon les données anthropométriques collectées par les enquêteurs à la sortie), mais seulement 12 de ces enfants avaient effectivement reçu un diagnostic de MAM par les agents de santé (AS) lors des consultations. Dans le service STA, il y avait également de mauvais diagnostics : la moyenne du z-score du rapport poids pour taille était au-dessus de -3, ce qui suggère qu'en moyenne les enfants entrant dans le service ne souffraient pas de MAS (mais de MAM) ; cela étant, le manque de documentation concernant les périmètres brachiaux (PB) et les œdèmes dans

les fiches individuelles fait qu'il est difficile d'évaluer la qualité du diagnostic à l'admission dans ce service. Par ailleurs, parmi les 514 évaluations de consultations et entretiens à la sortie, 27 enfants souffraient de MAS (selon les données anthropométriques collectées à la sortie par les enquêteurs), mais seulement deux de ces enfants ont effectivement reçu ce diagnostic par l'AS lors des consultations. Un problème fondamental qui pourrait expliquer en partie l'erreur des diagnostics régulièrement observée de malnutrition aiguë est le fait que seulement 14% des formations sanitaires (FOSAs) avait tout l'équipement nécessaire pour les mesures anthropométriques à la fois disponible et fonctionnel.

Qualité du traitement. Il s'agit là de points malheureusement mal renseignés dans les fiches individuelles de suivi. Néanmoins, à partir des questions adressées aux 90 responsables de services de nutrition, on note que 89% des FOSAs ont connu une rupture de stock de compléments alimentaires dans les trois mois précédant l'enquête et 100% des FOSAs ont connu une rupture de stock de médicaments nécessaires pour les services SSN et STA dans la même période de temps. Il est apparu que les taux de consommation mensuels pour les médicaments avaient été calculés et rapportés dans la plupart des FOSAs ; par contre il y avait très peu de données similaires en ce qui concerne les compléments alimentaires (CSB, huile, sucre et PlumpyNut). Cela pourrait suggérer que le manque de compléments alimentaires est un problème avec la gestion des stocks au niveau de la FOSA, alors que le manque de médicaments semble être au-delà et pourrait signifier un problème à un niveau plus central.

Qualité des consultations – anamnèse et examens physiques généraux. Globalement, cette enquête a observé de faibles niveaux de performance des AS par rapport au respect des protocoles internationaux et nationaux tels que la PCIME et le protocole national de PEC de la malnutrition. Par exemple, à partir des observations des consultations (N=514), moins de 1% des enfants ont été interrogés sur les trois signes de danger et moins de 1% se sont vus poser des questions sur les cinq symptômes principaux de la PCIME. En outre, dans les cas où les AS ont posé des questions à propos de l'un des cinq symptômes principaux, ils ont ensuite systématiquement omis de poser d'autres questions exploratoires ou d'effectuer des examens physiques pertinents à l'égard de ces symptômes et des signes après avoir reçu une réponse positive. Par ailleurs, seulement 13,5% des enfants ont été interrogés sur leur statut de vaccination et aucun des enfants observés lors des consultations n'a passé les examens physiques de base dans la liste fixe du questionnaire. Ces résultats médiocres à l'égard de la pédiatrie générale ont également été observés avec les séries de trois vignettes cliniques proposées aux 145 AS (env. 1.6 AS par FOSA), ce qui est d'autant plus surprenant car ils disposaient alors de plus de temps pour examiner ces cas fictifs (par rapport à de réelles consultations) et qu'il leur avait été demandé de donner des réponses en fonction des besoins cliniques plutôt qu'en fonction des manques de ressources qu'ils avaient (souvent) dans leur centre.

Qualité des consultations – anamnèse et examens physiques relatifs à la nutrition. Aucun des enfants n'a été interrogé sur toutes les questions de nutrition spécifiques (ajustement pour l'âge), et seulement 2,5% des enfants ont passé tous les examens physiques liés à la nutrition. Le poids est la mesure la plus souvent prise (52% des cas), la taille et le périmètre brachial (PB) sont les prochaines mesures les plus fréquemment prises (32% et 30% respectivement). Peu d'enfants ont été évalués pour la présence d'œdème (12,5%), un facteur qui peut faire basculer le diagnostic d'un enfant ayant un z-score de poids

pour taille ou un PB qualifiés de normaux ou de MAM, à un diagnostic de MAS. Ces résultats médiocres à l'égard de la nutrition ont également été observés lors de l'administration des trois vignettes cliniques.

Qualité des mesures anthropométriques. Les mesures de taille enregistrées par les AS sont significativement inférieures à celles des enquêteurs - en utilisant le même équipement (de la FOSA) ; ceci pourrait conduire à des calculs de z-scores plus élevés et ainsi à un sous-diagnostic de la malnutrition aiguë. Au contraire, les PBs pris par les AS étaient significativement inférieurs à ceux pris par les enquêteurs ce qui pourrait conduire à un sur-diagnostic de la malnutrition aiguë, mais cela n'a pas été observé dans les résultats. Un autre facteur qui pourrait avoir conduit au sous-diagnostic de la malnutrition aiguë vu dans cette enquête, était la différence statistiquement significative observée dans les poids pris par les enquêteurs avec les balances de la FOSA par rapport aux balances de l'enquête : les balances de l'enquête ont toujours montré un poids inférieur. Cette différence est le résultat d'un problème relatif à l'équipement plutôt que d'erreur humaine (avec le même équipement, les mesures de poids étaient similaires).

Communication. Un des aspects les plus négligés de la consultation (observé également dans les vignettes cliniques) était la communication entre les AS et les accompagnants : on a calculé des pourcentages très faibles d'AS qui ont donné des conseils de santé ou de nutrition aux accompagnants ou qui ont expliqué correctement le diagnostic ou le traitement pour leur enfant.

Formation et supervision des AS. Parmi les raisons qui pourraient expliquer cette mauvaise performance observée chez les AS, on constate de faibles niveaux initiaux en formation clinique (87% des FOSAs ne disposaient d'aucun AS avec des qualifications de niveau universitaire), le manque de formation professionnelle continue (moins de 50% des AS observés en consultations avaient reçu une formation sur la nutrition dans les six mois précédents et moins de 10% pour les sujets de santé communautaire), le manque de supervision (moins de 40% des AS observés en consultations avaient reçu une supervision d'une autorité externe à la FOSA sur le thème de la malnutrition dans les six derniers mois). La majorité des AS (90%) observés en consultation avaient reçu leurs salaires au complet et à temps, donc ce ne serait probablement pas une raison pour une certaine démotivation et ainsi une telle mauvaise performance.

Satisfaction des patients. Les entretiens à la sortie avec les accompagnants des enfants ont confirmé une mauvaise performance des AS en termes de communication. Cela étant, très peu d'accompagnants (2%) ont signalé qu'absolument rien ne les avait satisfaits à propos de leur visite ce jour-là. Il est intéressant de noter, bien que la majorité d'entre eux ait déclaré que rien ne les avait déçus, que 81% d'entre eux ont effectivement eu des suggestions d'amélioration - ce qui pourrait être une façon plus souple d'exprimer leurs insatisfaction avec les services sans le dire directement.

Enquête au niveau de la communauté

Les données ont été collectées auprès de 6,199 ménages et enfants de 6-23 mois entre le 8 décembre 2014 et le 13 janvier 2015.

Sécurité alimentaire des ménages. La méthodologie utilisée et suivant les questions génériques HFIAS d'échelle de l'accès déterminant l'insécurité alimentaire des ménages pour la mesure de l'accès

alimentaire des ménages a permis d'observer que 68% des ménages enquêtés subissaient une grave insécurité alimentaire, 19% une insécurité alimentaire modérée, et 13% une insécurité alimentaire faible ou nulle. Ces chiffres sont à la fois impressionnants et inquiétants, et mettent certainement en évidence un domaine qui nécessite de l'attention.

Soins et alimentation apportés à l'enfant. Plus de la moitié des enfants enquêtés avaient été malades durant les deux semaines précédant l'enquête (59%), et 81% d'entre eux ont consulté les services de santé, ce qui semble assez élevé si on note que 50% des répondants reportaient une distance au centre de santé supérieure à une heure. La majorité des indicateurs relatifs à l'allaitement étaient satisfaisants au regard des recommandations nationales d'alimentation du jeune enfant (ANJE). Cependant, les pratiques d'alimentation complémentaire n'étaient en moyenne pas satisfaisantes : seuls 50% des enfants avaient une diversité alimentaire suffisante, à savoir minimum quatre groupes alimentaires, et 44% avaient une fréquence de repas suffisante ; au total, seuls un quart des enfants recevaient une alimentation complémentaire acceptable en terme de fréquence et de diversité (indicateurs adaptés selon leur âge).

Etat nutritionnel de l'enfant. Les données collectées en décembre 2014 et janvier 2015 ont permis d'observer un taux de malnutrition aigüe parmi les enfants de 6-23 mois de 6.0%, et un taux de malnutrition chronique de 53.1% à cette période. Les analyses bivariées et multivariées ont confirmé un certain nombre de relations connues entre les taux de malnutrition et les déterminants socio-économiques, à savoir que les taux élevés de malnutrition aiguë sont observés dans les ménages avec des scores socio-économiques inférieurs (ici, les deux quintiles les plus bas), qui souffrent d'insécurité alimentaire et dont les mères ont un faible niveau d'éducation. En outre, la relation bien connue entre les taux élevés de malnutrition aiguë chez les enfants qui avaient été récemment malades (dans les deux dernières semaines) a également été observée, ce qui souligne le cercle vicieux de la maladie et de la malnutrition. Les résultats plus exceptionnels de cette enquête comprennent la prévalence de la malnutrition aiguë significativement supérieure chez les garçons par rapport aux filles. Enfin, il n'y avait pas de différence significative dans les taux de malnutrition aiguë entre les groupes d'âge, mais cela pourrait être expliqué par le fait que les enfants de la tranche d'âge des 6-23 mois sont les plus à risque, et ainsi au sein de cette tranche d'âge le risque est susceptible d'être similaire quel que soit l'âge.

Validité de l'étude

Validité externe

Au niveau des centres de santé. De par la sélection des 90 centres de santé de l'étude, l'échantillon couvre l'ensemble du territoire burundais, excepté la province de Bujumbura mairie. Les données collectées ont pu être comparées à des données provenant d'autres enquêtes d'envergure nationale. Ainsi, les caractéristiques générales des centres de santé ici observées sont relativement similaires avec celles observées lors de l'enquête FOSA 2013 réalisée par l'INSP (réf. MSPLS et al. 2014). Aussi, les données relatives aux indices de performance des services SSN et STA ont été validées après comparaison avec les statistiques collectées par le PRONIANUT pour l'année 2012.

Au niveau de la communauté. Le taux de malnutrition chronique observé parmi les enfants de 6-23 mois est relativement similaire à celui observé lors de l'enquête démographie et santé 2010 (EDS 2010). Par contre, le taux de malnutrition aigüe a chuté entre l'enquête EDS de 2010 (10% pour cette tranche d'âge) et l'enquête ici présentée (6%). Il se peut qu'en quatre années la situation se soit améliorée, mais on doit également garder à l'esprit que la malnutrition aigüe est un évènement de court terme, et que des variations relativement importantes peuvent être observées selon la saison. Or, l'enquête EDS 2010 couvrait une des périodes de soudure (octobre-novembre), tandis que l'enquête ici présentée était réalisée en décembre et début janvier, ce qui correspond à une période de récolte, où la nourriture est plus disponible. Enfin, les statistiques relatives à la sécurité alimentaire ont pu être comparées avec les données collectées dans les provinces de Cankuzo et Ruyigi en octobre 2012 dans le cadre d'une évaluation de programme de prévention de la malnutrition (réf. Projet FANTA), et se sont avérées relativement similaires.

Validité interne

Puissance de calcul ex-post au niveau des centres de santé. La taille de l'échantillon de fiches individuelles de suivi à collecter avait été calculée sur la plus petite différence de taux de guérison qui pouvait être considérée comme significative d'un point de vue de santé publique ; on avait alors fait l'hypothèse que l'intervention FBP Nutrition pourrait permettre une augmentation du taux de guérison de la malnutrition aigüe de 80% à 90%, en considérant une erreur de type alpha de 5%, un pouvoir statistique de 80% et un ICC de 0.15. Selon les données collectées, les taux de guérison de malnutrition aigüe modérée et sévère sont respectivement de 80% (N=628) et 85% (N=665), ce qui est proche de notre hypothèse de départ, mais avec un nombre inférieur d'observations (étant donné la faible qualité de documentation). Par ailleurs, étant donné le niveau de corrélation intra-centre de santé plus important que prévu dans le service SSN, la puissance de calcul pour ce service tombe de 80% à 60%. Concernant le service STA, le niveau de corrélation intra centre de santé est plus faible, et la puissance de calcul demeure à 80% si on suppose que l'intervention permettra une augmentation du taux de guérison de MAS de 85% à 95%. Notons tout de même qu'il s'agit ici de calculs hypothétiques, et que la puissance de calcul finale dépendra fortement de la qualité de documentation des fiches individuelles de suivi en 2016, avant l'enquête finale.

Puissance de calcul ex-post au niveau de la communauté. La taille de l'échantillon d'enfants à enquêter avait été calculée sur base du taux de malnutrition aigüe dans ce groupe d'âge de 6-23 mois au Burundi. L'hypothèse de départ était de 10%, et était basée sur les données de l'enquête démographie et de santé 2010 (EDS 2010). Les données collectées ici observent en fait un taux de malnutrition aigüe de 6%, mais une corrélation intra-grappes bien moindre à ce qui avait été supposé lors du calcul initial. En prenant ces données en compte, la puissance de calcul d'une réduction du taux de malnutrition aigüe de 6% à 4.5% est réduite à 60% ; elle demeure à 80% si on observe une réduction du taux de malnutrition aigüe plus importante, à savoir de 6% à 4%.

Comparabilité des deux groupes. Aucune différence significative n'a été observée entre les deux groupes (contrôle et intervention) en ce qui concerne les variables d'intérêt primaire pour cette recherche, qu'il s'agisse des données au niveau des centres de santé ou au niveau des ménages. De plus, la plupart des variables calculées ont été testées pour la différence entre les groupes de traitement et

de contrôle, et la majorité des différences s'avèrent non significatives, ce qui suggère fortement que les deux groupes sont similaires, et donc comparables.

Recommandations

A l'égard de l'équipe d'évaluation

Améliorer les questionnaires. Les enquêtes de références présentées ici ont bien répondu à nos attentes et sont de bonne qualité pour la plupart des variables. Cela étant, ont été identifiés quelques éléments pouvant être modifiés pour améliorer encore la qualité et la complétude des données. Aussi, les questionnaires, ainsi que les programmes d'entrée des données et les manuels des enquêteurs seront révisés.

Préparer les enquêtes de suivi. Ensuite, l'équipe devra à nouveau préparer bien en amont les enquêtes de suivi, et considérer environ quatre mois de travail préparatoire avant le travail sur le terrain, en collaboration avec les instituts mettant en œuvre les enquêtes, l'INSP et l'ISTEEBU, ainsi qu'avec la Banque Mondiale, qui contractualise et finance ces derniers.

Assurer un suivi rapproché durant la période d'intervention. Il est important d'effectuer un suivi-évaluation rapproché de la mise en œuvre de l'intervention, durant toute la période d'exposition, pour permettre d'expliquer par quels mécanismes le FBP Nutrition a un impact, mais aussi pour pourvoir expliquer les raisons d'un éventuel échec. Il s'agit notamment d'observer des éléments tels que la problématique de l'approvisionnement en intrants diététiques pour le SSN, qui n'est plus assuré par le PAM et pour lequel aucune alternative n'est encore mise en place ; la formation au nouveau protocole de prise en charge de la malnutrition aigüe (MSPLS, OMS, UNICEF, 2014) qui n'est pas encore totalement réalisée pour le STA et pas démarrée pour le SSN ; les retards dans la mise en œuvre de l'intervention, par exemple, au moment de l'écriture de ce rapport, les contrats secondaires entre les CDS nutritionnels et les CDS non nutritionnels qui réfèrent vers les premiers les cas de MA dépistés, permettant avec le volet communautaire de compléter la chaîne des agents concernés par la MA, ne sont pas encore établis. Enfin, l'équipe d'évaluation doit également faire attention aux éléments externes pouvant altérer l'efficacité de l'intervention.

Plaider pour un bon remplissage et archivage des fiches individuelles de suivi et des registres. La mauvaise qualité du remplissage et de l'archivage des fiches individuelles de suivi et des registres des services SSN et STA affecte la puissance de calcul ex-post de l'évaluation d'impact au niveau des CDS. Aussi, afin d'optimiser le pouvoir statistique de cette évaluation, l'équipe se doit de plaider pour un bon remplissage et archivage de ces fiches et registres.

Organiser des formations personnalisées sur demande des CDS. Partant du manque de connaissances des AS révélé par cette étude, l'équipe propose deux formations pour améliorer la qualité des services. Les titulaires et gestionnaires des CDS sont en train d'exprimer, à travers des bons de commande et selon un processus d'enchère de Vickrey, leur demande et leur volonté à payer pour ces formations. Une fois tous les bons de commande collectés, l'équipe d'évaluation identifiera les CDS qui ont manifesté le plus d'intérêt, et organiser les formations.

A l'égard des parties prenantes dans la mise en œuvre de l'intervention

Il est attendu que l'introduction du FBP Nutrition au Burundi permette d'améliorer la performance des AS en matière de prévention et de prise en charge de la malnutrition, à travers les centres de santé du pays. Un certain nombre de problèmes sont néanmoins à résoudre pour garantir son efficacité.

Disponibilité des intrants. A ce jour, on note que les intrants diététiques dans le SSN ne sont pas disponibles dans la plupart des provinces, ce qui rend *de facto* les services SSN non effectifs. Pour pallier ce problème, il est urgent que des fournisseurs de farine, chez qui les CDS pourront se fournir, soient identifiés. La disponibilité des intrants concerne également les intrants diététiques dans le STA et les intrants liés au traitement systématique : on a noté des ruptures de stock en Plumpy Nut dans 55% des CDS ainsi que des kits de produits de traitement systématique incomplets dans 76% des CDS. Or il est urgent et important de garantir la possibilité continue pour les CDS de s'approvisionner en intrants pour que l'intervention FBP Nutrition soit efficace.

Formations des AS. Comme dit plus haut, les formations au nouveau protocole de prise en charge de la malnutrition aigüe (MSPLS, OMS, UNICEF, 2014) sont seulement en cours de réalisation sur le STA, et n'ont pas encore démarré sur le SSN. Il est urgent que ces formations soient réalisées, sans quoi est compromise la réussite de l'intervention FBP Nutrition – compte tenu par ailleurs de la faible qualité des services de santé en matière de prévention et prise en charge de la malnutrition relevée dans ce rapport. Des formations des AS au suivi et à la promotion de la croissance, à la prise et interprétation de mesures anthropométriques, ainsi qu'aux conseils d'hygiène et diététiques nécessaires à un bon état nutritionnel seraient également utiles.

Achever la mise en œuvre de l'intervention FBP Nutrition. Certains retards ont été observés concernant la mise en œuvre même de l'intervention, notamment sur le volet communautaire de l'intervention et sur les contrats secondaires entre les CDS nutritionnels et les CDS non nutritionnels qui réfèrent vers les premiers les cas de MA dépistés. La mise en œuvre effective de ces éléments doit être suivie de près et corrigée le cas échéant. Par ailleurs, le volet qualitatif de l'intervention, quoique évalué depuis juillet 2015 pour le deuxième trimestre, n'est pas encore pris en compte dans le calcul des subsides versés aux CDS du groupe d'intervention, en raison de malus trop élevés pour l'ensemble des CDS : si la raison invoquée est compréhensible, il est néanmoins important de communiquer aux CDS du groupe d'intervention leurs scores qualité ainsi que de les inciter à mieux faire les prochains mois en leur assurant que la qualité sera cette fois prise en compte pour le calcul des subsides.

A l'égard du MSPLS de manière plus générale

Amélioration de la performance des agents de santé

L'introduction du FBP pour la nutrition au Burundi devrait permettre d'améliorer la performance des AS à travers les différents CDS. Un certain nombre d'autres recommandations ont été identifiées à partir de ce rapport.

Effectuer des recherches qualitatives sur les attitudes et les opinions des AS. Des entretiens approfondis et des groupes de discussion pourraient être utilisés pour élucider les défis et les besoins des AS à l'égard de leur travail quotidien dans les centres de santé.

Augmenter la fréquence et la qualité de la formation des AS. Pour la formation continue, il semble primordial de fournir aux AS une formation personnalisée à leurs besoins, ou au moins aux besoins identifiés pour le CDS dans son ensemble; plutôt que des formations génériques sur la malnutrition ou sur la PCIME.

Disponibilité et mise en œuvre des protocoles nationaux. Une recommandation forte serait de fournir aux FOSAs des copies de tous les protocoles pertinents (notamment avec le nouveau protocole national pour la prise en charge de la malnutrition de 2014 à mettre en œuvre cette année) et, le cas échéant, de mettre en place un système de sécurité ou un système de prêt en bibliothèque pour s'assurer que les AS aient toujours accès à ceux-ci lors des consultations. Ceci devrait être complété par une supervision et un audit de l'utilisation de ces protocoles (voir la recommandation suivante).

Supervision améliorée. Une autre recommandation forte serait d'améliorer la formation des superviseurs (à différents niveaux) pour faire en sorte que leurs visites de supervision soient efficaces en termes de transfert des connaissances théoriques. Un point important est de veiller à ce que les visites de supervision prévues soient réalistes en termes de temps et de contexte géographique ; concrètement, les superviseurs devraient prévoir suffisamment de temps dans chaque centre et avoir déjà une connaissance de base des besoins des AS.

Création de stages et de centres d'excellence. Un certain nombre d'AS ont signalé des lacunes dans leurs connaissances dans des domaines spécifiques, par exemple dans la prise en charge des enfants avec le VIH. En plus de formations et de supervisions améliorées sur ces sujets, il est suggéré de mettre en place un système où les AS iraient faire un «stage» dans une FOSA, identifiée comme ayant un grand nombre de ces cas spécifiques ou ayant un personnel expérimenté dans de tels soins, afin qu'ils puissent apprendre de manière pratique. Ces centres pourraient recevoir un soutien externe pour devenir, par exemples, le centre d'excellence pour la gestion des enfants séropositifs dans la santé primaire ou celui pour la gestion des enfants MAS avec la tuberculose.

Amélioration du fonctionnement quotidien des services de pédiatrie et de nutrition

Les efforts pour améliorer le fonctionnement quotidien des services pédiatriques et de la malnutrition pourraient avoir également un impact positif sur la performance des AS (cela doit être vérifié par des recherches qualitatives sur les défis et les besoins des AS). Les recommandations seraient:

Amélioration de la disponibilité et de la fonctionnalité de l'équipement. Les CDS manquent de nombreuses pièces essentielles de matériel ou de ressources pour évaluer l'état nutritionnel des enfants. Les fonds des CDS, provenant non seulement des allocations FBP mais également d'autres sources, devraient être en priorité alloués à l'amélioration de la qualité de l'équipement et à l'assurance de sa sécurité et de son entretien dans les centres.

Amélioration de l'organisation et de la gestion des données des patients dans les services de la malnutrition. Les résultats de cette enquête ont montré un clair besoin d'amélioration dans la gestion des données patient et dans l'organisation des services de la malnutrition. Il pourrait y avoir un certain nombre de solutions simples à cela, notamment en améliorant le système de stockage des fiches du patient. Aussi, plutôt que d'avoir un seul membre du personnel affecté à cette tâche, il pourrait y avoir

une petite équipe qui pourrait maintenir la continuité de la gestion de la documentation, même avec des changements de personnel ou des absences. Une autre option serait de garder l'original des carnets de santé / fiches ou des copies dans les CDS plutôt que de les donner aux accompagnants.

A l'égard des commanditaires de l'étude (MSPLS et Banque Mondiale)

Préparation des enquêtes de suivi. Afin d'optimiser la qualité des enquêtes de suivi et d'éviter des retards dans les activités de formation mais surtout de collecte des données, il est ici rappelé de bien inscrire ces enquêtes dans l'agenda des enquêtes à réaliser pour le MSPLS et la Banque Mondiale, ainsi que d'effectuer à temps les contractualisations avec les instituts pressentis pour réaliser les enquêtes (INSP et ISTEEBU). Ceci devra faire l'objet d'une collaboration rapprochée avec l'équipe d'évaluation.

Recommandations majeures. Pour que, à la fin de ce projet d'évaluation, l'intervention FBP Nutrition ait un impact positif et significatif sur les taux de guérison de MA et sur la prévalence de MA dans la communauté, un certain nombre d'obstacles sont à lever rapidement. Il s'agit principalement (1) du problème de la disponibilité des intrants nutritionnels dans le SSN, et (2) de l'amélioration de la performance des AS par, au minimum, des formations au nouveau protocole de prévention et prise en charge de la malnutrition ainsi qu'au suivi et promotion de la croissance. Concernant le premier problème, la possibilité pour les CDS d'acheter des intrants nutritionnels pour les SSN est prévue d'être effective dès novembre 2015 (ce qui reste à confirmer). Concernant le second problème, la formation des AS au nouveau protocole de prévention et de prise en charge de la malnutrition aigüe est seulement en cours de réalisation sur le STA, et n'est pas encore démarrée sur le SSN. Ces deux problèmes, tant qu'ils n'auront pas été réglés, rendront l'intervention FBP Nutrition inefficace. Par ailleurs, si, comme cela est prévu, les enquêtes de suivi ont lieu en juillet 2016, soit 18 mois après le démarrage officiel de l'intervention, de par sa forme, la collecte de données relatives au taux de guérison de MAM et de MAS concerne la période des six mois précédents. Cela signifie que, si tant est que les intrants deviennent effectivement disponibles à partir de novembre 2015 et que les formations au nouveau protocole se terminent avant fin 2015, la durée d'exposition à l'intervention effective n'aura pas été de 18 mois mais de 1 à 6 mois selon les observations. Ceci présage d'un résultat nul ou quasi nul lors de l'évaluation d'impact de l'intervention au niveau des CDS. Cette inquiétude demeure au niveau communautaire tant que les problèmes ne sont pas résolus au niveau des CDS et étant donné que le volet communautaire de l'intervention n'a été mis en place qu'en octobre 2015. Compte tenu de ces risques, il est proposé aux commanditaires de l'étude d'envisager de prolonger la durée d'exposition à l'intervention – ce qui semble possible d'un point de vue budgétaire du côté de la mise en œuvre étant donné que le taux de consommation actuellement du budget dédié à l'intervention reste autour de 10%. Parallèlement, il est suggéré de continuer à soutenir les parties prenantes de la mise en œuvre de l'intervention, à assurer un suivi et évaluation de la mise en œuvre effective ainsi que des défis à relever.

Acronymes et abréviations

ACT Combinaisons Thérapeutiques à base d'Artémisinine (*Artemisinin-based Combination Therapy*)
ANJE Alimentation du nouveau-né et du jeune enfant
AS Agent de santé
ASC Agent de santé communautaire
BF Allaitement (*Breastfeeding*)
BM Banque Mondiale
CDS Centre de santé
CSB Mélange maïs-soja (*Corn Soya Blend*)
CTA Combinaisons Thérapeutiques à base d'Artémisinine
CT-FBP Cellule Technique pour le FBP
DPSHA Direction de la Promotion de la Sante Hygiène et Assainissement
EDS Enquête Démographie et Santé
FANTA Food and Nutrition Technical Assistance
FARN Foyers d'Apprentissage et de Réhabilitation Nutritionnelle
FBP Financement Basé sur la Performance
FOSA Formation sanitaire
GASC Groupement d'agents de santé communautaire
HAZ z-score du rapport taille pour âge *(Height-for-Age Z-score)*
HFIAS Echelle de l'accès déterminant l'insécurité alimentaire des ménages *(Household Food Insecurity Access Scale)*
ICC Coefficient de correlation intra-classe *(Intraclass Correlation Coefficient)*
IMT Institut de Médecine Tropicale d'Anvers
INSP Institut National de Santé Publique
ISTEEBU Institut de Statistiques et d'Etudes Economiques du Burundi
IV Intraveineuse
IYCF Alimentation du nouveau-né et du jeune enfant *(Infant and Young Child Feeding)*
LQAS Assurance de qualité de lots *(Lot Quality Assurance Sampling)*
MA Malnutrition aigüe
MAM Malnutrition aigüe modérée
MAS Malnutrition aigüe sévère
MSPLS Ministère de la Santé Publique et de la Lutte contre le SIDA
MUAC Périmètre brachial *(Mid-Upper Arm Circumference)*
ODK Open Data Kit
OMD Objectif du Millénaire pour le Développement
OMS Organisation Mondiale de la Santé
ONG Organisation Non-Gouvernementale
ORS Solution de réhydratation orale *(Oral Rehydration Solution)*
PAM Programme Alimentaire Mondial
PB Périmètre brachial
PCIME Prise en charge intégrée des maladies de l'enfant
PEC Prise en charge
PMA Paquet minimum d'activités
PNUD Programme des Nations Unies pour le Développement
PRONIANUT Programme de Nutrition (au MSPLS)
P/T Poids pour taille

PTF	Partenaire technique et financier
SD	Ecart-type *(Standard Deviation)*
SRO	Solution de réhydratation orale
SSN	Service de Supplémentation Nutritionnelle (service de prise en charge de la MAM)
SST	Service de Stabilisation (service de prise en charge de la MAS avec complications)
STA	Service de Thérapeutique Ambulatoire (service de prise en charge de la MAS sans complications)
T/A	Taille pour âge
UNICEF	Fonds des Nations Unies pour l'Enfance *(United Nations Children's Fund)*
URTI	Upper Respiratory Tract Infection (Infection Respiratoire Supérieure Aigue)
WHZ	Z-score du rapport poids pour taille *(Weight-for-Height Z-score)*

Index des tableaux

Tableau 1. Indicateurs conçus dans le cadre de l'intervention du FBP Nutrition 5
Tableau 2. Indicateurs conçus dans le cadre de l'intervention du FBP Nutrition 26
Tableau 3. Chemins à considérer dans les groupes d'intervention et de contrôle 30
Tableau 4. Variables de résultats à collecter et analyser 33
Tableau 5. Variables de résultats à analyser au niveau de la structure de soins 34
Tableau 6. Résumé des vignettes cliniques 43
Tableau 7. Catégories de sécurité alimentaire, selon HFIAS 46
Tableau 8. Principales caractéristiques des centres de santé et équipement anthropométrique selon l'enquête FOSA 2013 et l'enquête FBP Nutrition de référence auprès des centres de santé (2014) 50
Tableau 9. Performance des services de nutrition selon le PRONIANUT et l'enquête FBP Nutrition de référence auprès des centres de santé (2014) 51
Tableau 10. Comparaisons de mesures anthropométriques entre les EDS 2010 et l'enquête FBP Nutrition de référence auprès des ménages 52
Tableau 11. Statistiques de sécurité alimentaire dans les provinces de Cankuzo et Ruyigi, de l'étude d'évaluation de Parket et al. (2012) à l'enquête FBP Nutrition de référence au niveau des ménages (2014-15) 53
Tableau 12. Taux de couverture pour chacun des outils de collecte de l'enquête CDS 54
Tableau 13. Informations manquantes sur les fiches individuelles de suivi ou registres SSN 61
Tableau 14. Mesures anthropométriques à l'entrée et à la sortie et les raisons de sortie du service SSN (là où l'information est disponible) 62
Tableau 15. Performance du service SSN par rapport aux directives nationales 63
Tableau 16. Traitement systématique et diététique au service SSN 63
Tableau 17. Informations manquantes sur les fiches individuelles de suivi ou les registres STA 65
Tableau 18. Mesures anthropométriques à l'entrée et à la sortie et les raisons de la sortie du service STA, lorsque l'information est disponible 66
Tableau 19. Performance du STA comparée aux directives nationales 67
Tableau 20. Traitements systématique et diététique en STA 67
Tableau 21. Nombre de cas interrogés sur les signes de danger, les principaux symptômes et les questions autonomes 69
Tableau 22. Nombre de cas avec examens et signes vitaux effectués 70
Tableau 23. Questions et examens effectués sur les cas pour lesquels est déclarée la présence de diarrhée 71
Tableau 24. Questions posées aux cas ayant déclaré la présence de la toux 72
Tableau 25. Questions et examens effectués sur les cas déclarés avec présence de fièvre 73
Tableau 26. Questions et examens relatifs à la nutrition exécutés 75
Tableau 27. Fréquence et catégorie de conseils de nutrition donnés aux patients 76
Tableau 28. Conseils de nutrition donnés aux accompagnants d'enfants qui ont été diagnostiqués comme des cas de MAM par l'agent de santé 77
Tableau 29. Catégories de conseils nutritionnels donnés aux accompagnants au cours de la consultation 79
Tableau 30. Principales catégories d'aspects qui ont le plus satisfait les accompagnants en ce qui concerne la visite actuelle au centre de santé 79
Tableau 31. Suggestions d'améliorations possibles des services 81
Tableau 32. Nombre d'agents de santé qui ont interrogé sur les signes de danger, les principaux symptômes et des questions autonomes - Vignette no. 1 83
Tableau 33. Fréquence et type d'examens physiques que les agents de santé ont indiqué vouloir effectuer - Vignette no.1 84

Tableau 34. Questions complémentaires relatives à la toux 85
Tableau 35. Questions spécifiques à la nutrition - Vignette no.1 86
Tableau 36. Examens et tâches spécifiques de nutrition - Vignette no.1 87
Tableau 37. Diagnostics effectués par les AS - Vignette no.1. 89
Tableau 38. Conseils et explications donnés à l'accompagant - Vignette no.1 90
Tableau 39. Catégories de médicaments prescrits par les AS - Vignette no.1 91
Tableau 40. Réponses à la question en rapport avec les antibiotiques - Vignette no.1 91
Tableau 41. Réponses aux questions en rapport avec les antibiotiques parmi les AS qui ont signalé le diagnostic de pneumonie - Vignette no.1 91
Tableau 42. Plan de traitement - Vignette no.1 92
Tableau 43. Nombre d'AS qui ont cherché les signes de danger, les principaux symptoms et posé les questions autonomes 93
Tableau 44. Fréquence et type d'examens physiques que les AS effectueraient – Vignette no.2 94
Tableau 45. Questions et examens physiques effectués après déclaration de la presence de diarrhée – Vignette no.2 95
Tableau 46. Questions spécifiques à la nutrition - Vignette no.2 96
Tableau 47. Examens et tâches spécifiques à la nutrition - Vignette no.2 96
Tableau 48. Diagnostics effectués par les AS - Vignette no.2 97
Tableau 49. Conseils et explications données à l'accompagnant - Vignette no.2 98
Tableau 50. Catégories de médicaments prescrits par les AS - Vignette no.2 99
Tableau 51. Réponses sur la question en rapport avec les antibiotiques - Vignette no.2 99
Tableau 52. Indication donnée sur la prescription des antibiotiques* - Vignette no.2 99
Tableau 53. Plans de prise en charge - Vignette no.2 100
Tableau 54. Nombre d'AS qui ont cherché les signes de danger, les principaux symptômes et posé les questions autonomes-vignette no.3 101
Tableau 55. Fréquence et type d'examen physique que les AS ont signalé qu'ils effectueraient - Vignette no.3 102
Tableau 56. Questions spécifiques de nutrition - Vignette no.3 102
Tableau 57. Examens et tâches spécifiques de nutrition – Vignette no.3 103
Tableau 58. Diagnostics et fréquence avec laquelle ils étaient donnés par les AS-vignette no.3 104
Tableau 59. Conseils et explications donnés à l'accompagnant 105
Tableau 60. Catégories de médicaments prescrits par les AS - Vignette no.3 106
Tableau 61. Type d'anti-malaria prescript par les AS 106
Tableau 62. Destination des references/plan de prise en charge 106
Tableau 63. Formation professionnelle continue des AS (PRONIANUT) 107
Tableau 64. Formation professionnelle continue des AS (DPSHA) 107
Tableau 65. Domaines nécessitant de formation complémentaire selon les AS 107
Tableau 66. Visites de supervision reçues par les AS au cours des six derniers mois 108
Tableau 67. Caractéristiques générales et socio-économiques des ménages 109
Tableau 68. Conditions de sécurité alimentaire 110
Tableau 69. Prévalence des différentes catégories de sécurité alimentaire 111
Tableau 70. Caractéristiques générales des responsables des enfants 111
Tableau 71. Caractéristiques générales de l'enfant 111
Tableau 72. Etat de santé de l'enfant 112
Tableau 73. Indicateurs relatifs à l'allaitement 113
Tableau 74. Indicateurs relatifs à l'alimentation de complément 114
Tableau 75. Mesures anthropométriques 116

Tableau 76. Relation entre l'âge et le taux de malnutrition aigüe – analyse univariée utilisant les estimations logistiques 117
Tableau 77. Relation entre le genre et le taux de malnutrition aigüe – analyse univariée utilisant les estimations logistiques 117
Tableau 78. Relation entre état de santé et taux de malnutrition aigüe –analyse univariée utilisant les estimations logistiques 117
Tableau 79. Relation entre état de santé et taux de malnutrition aigüe sévère –analyse univariée utilisant les estimations logistiques 118
Tableau 80. Relation entre les quintiles socio-économiques et le taux de malnutrition aigüe – analyse univariée utilisant les estimations logistiques 118
Tableau 81. Relation entre le niveau d'éducation des responsables des enfants et le taux de malnutrition aigüe – analyse univariée utilisant les estimations logistiques 118
Tableau 82. Relation entre le score de sécurité alimentaire des ménages et le taux de malnutrition aigüe - analyse univariée utilisant les estimations logistiques 119
Tableau 83. Comparabilité des centres de santé des groupes de contrôle et d'intervention en terme de gestion (questionnaire G) 148
Tableau 84. Comparabilité des services de supplementation nutritionnelle (SSN) des groups de contrôle et d'intervention 149
Tableau 85. Comparabilité des services de thérapeutique ambulatoire (STA) dans les groups de contrôle et d'intervention 152
Tableau 86. Comparabilité des centres de santé des groups de contrôle et d'intervention en termes de qualité des consultations (quest. C) 155
Tableau 87. Comparabilité des centres de santé des groups de contrôle et d'intervention – entretiens à la sortie 157
Tableau 88. Comparabilité des ménages des groups de contrôle et d'intervention – caractéristiques générales et socio-économiques 159
Tableau 89. Comparabilité des ménages des groups de contrôle et d'intervention en terme de sécurité alimentaire 161
Tableau 90. Comparabilité des enfants dans les groups de contrôle et d'intervention – caractéristiques générales et de santé 162
Tableau 91. Comparabilité des enfants dans les groups de contrôle et d'intervention – mesures anthropométriques 163

Index des figures

Figure 1. Mise en œuvre du FBP Nutrition et de la compensation 6
Figure 2. Mise en œuvre du FBP Nutrition et de la compensation 28
Figure 3: Chaine des résultats liés à l'introduction de la Nutrition dans le FBP 29
Figure 4. Structure des questionnaires de l'enquête au niveau des centres de santé 35
Figure 5. Carte du Burundi avec les 90 centres de santé de l'étude 49
Figure 6. Age d'introduction d'aliments de compléments (parmi les enfants âgés de 12-23 mois) 115

1. Vue d'ensemble du projet d'étude

1.1 Contexte

Objectifs du Millénaire pour le Développement (OMDs) au Burundi : où en sommes-nous et quels sont les principaux défis ?

Le Burundi est un petit pays enclavé se trouvant à l'Est de l'Afrique Centrale. Avec un Produit National Brut par habitant estimé en 2014 à 770 dollars internationaux (Banque Mondiale 2015), le Burundi est parmi les pays les plus pauvres du monde. La population burundaise est d'environ 10.6 millions avec 421 habitants par km carré. Toutefois il importe de signaler que la majorité de cette même population vit en milieu rural. Depuis l'indépendance, le Burundi a connu de continuels soubresauts de conflits et d'instabilité, et depuis 1993 jusqu'à récemment, le pays était en état de guerre civile. Par conséquent, l'état de santé de la population est parmi les plus mauvais d'Afrique. Entre 2006 et 2011, le taux de mortalité des enfants de moins de 5 ans était estimé à 96 décès pour 1000 naissances, tandis que le taux de mortalité maternelle était de 500 décès pour 100 000 naissances (EDS 2010).

Selon le rapport du PNUD de 2010 sur les OMD au Burundi, la probabilité d'atteindre les objectifs liés à la santé est plutôt limitée. Même si l'on constate de nombreux efforts dans le domaine de la vaccination, de la lutte contre les maladies graves ainsi que dans l'allocation des ressources à la santé, de nombreux défis demeurent, comme notamment la faible capacité du système sanitaire en termes d'infrastructures et du personnel, un taux de natalité élevé, l'insécurité alimentaire et sanitaire, etc. L'insécurité alimentaire est aussi la conséquence d'une forte pression démographique et d'un manque d'investissement dans le domaine de l'agriculture (UNDP 2010). Au Burundi, environ 28% des ménages souffrent d'insécurité alimentaire, et les taux de malnutrition chronique sont parmi les plus élevés au monde avec 58% des enfants de moins de 5 ans affectés par un retard de croissance (EDS 2010; WFP 2008).

Histoire du FBP au Burundi

Comme tout pays très pauvre, le Burundi a des contraintes budgétaires serrées eu égards des importants besoins de santé de sa population. Alors que les paiements directs demeurent la source de financement de la santé la plus importante, le gouvernement du Burundi a fait des efforts considérables ces dernières années : la décision présidentielle de supprimer les frais à charge de l'usager pour les enfants de moins de cinq ans et les accouchements au niveau des centres de santé était une étape clé de cet engagement. Cette décision était cohérente avec l'agenda des OMDs, adopté au Burundi, et a bénéficié d'un soutien politique fort. Cependant, comme pour d'autres initiatives similaires en Afrique sub-Saharienne (Meessen et al. 2011), cette décision n'était pas parfaitement planifiée et la suppression des frais à charge de l'usager a souffert de cette préparation limitée (Nimpagaritse et Bertone 2011), avec des défauts dans la conception et la mise en œuvre de l'initiative ainsi qu'un manque de système de suivi et évaluation robuste. L'augmentation soudaine et massive de l'utilisation des services de santé a également été source de frustrations et démotivations parmi le personnel de santé.

Parallèlement, dès 2006, le Burundi expérimentait dans trois provinces une nouvelle stratégie de financement de la santé, le financement basé sur la performance (FBP). Cette stratégie s'est développée petit à petit et a rapidement gagné en popularité pour finalement être mise en œuvre à l'échelle nationale en avril 2010. Le Burundi est ainsi devenu le deuxième pays d'Afrique à avoir mis en œuvre le FBP dans le secteur santé au niveau national. Dans le même temps, on a pu observer des améliorations importantes au niveau des mécanismes de financement de la santé, en termes de cohérence, gouvernance et vision. La décision la plus cruciale a probablement été celle de fusionner la stratégie de gratuité avec le programme de FBP (Basenya et al. 2011).

Toutes les formations sanitaires (FOSA) publiques et presque toutes les FOSA privées à but non lucratif sont couvertes par le programme national de FBP. Ce programme est gouverné par la Cellule Technique FBP (CT-FBP), un groupe technique basé au Ministère de la Santé Publique et de la Lutte contre le VIH/SIDA (MSPLS). Le système de FBP burundais reflète les principales priorités sanitaires du pays, à savoir le VIH/SIDA, la tuberculose, la santé maternelle et de l'enfant.

Le système de FBP repose sur des arrangements contractuels impliquant différentes parties. Les FOSA rapportent mensuellement leurs activités liées aux indicateurs FBP. Ces rapports sont ensuite vérifiés et validés au niveau provincial par le comité provincial de vérification et de validation (CPVV), puis envoyés à la CT-FBP, qui lance alors les transferts de subventions FBP aux FOSA. Dans les conditions normales de disponibilité des ressources chez tous les acteurs impliqués, le processus prend entre deux et trois mois (MSPLS 2011) ; cela peu prendre un peu plus de temps si l'un ou l'autre a des difficultés de trésorerie. Le programme national de FBP est financé à travers différentes sources, le gouvernement et la Banque Mondiale (HSDSP) étant les deux plus grosses sources de financement.

Malnutrition: un des principaux problèmes de santé publique au Burundi

Au Burundi, la malnutrition est une barrière évidente à la réalisation des OMDs liés à la santé. En effet, 58% des enfants de moins de 5 ans souffrent de malnutrition chronique et 6% de malnutrition aigüe (EDS, 2010). Les jeunes enfants sont particulièrement affectés : environ 10% des enfants de 6-18 mois souffraient de malnutrition aigüe en 2010, et 2.6% de malnutrition aigüe sévère. Selon la tendance internationale, en 2010, le Ministère chargé de la Santé du Burundi a développé un protocole proposant un plan de traitement et de suivi de la malnutrition aigüe pour les enfants de moins de cinq ans, intégré au système de santé (c'est-à-dire aux centres de santé et hôpitaux). Aujourd'hui, seulement un tiers des centres de santé et la moitié des hôpitaux offrent des services de prise en charge de la malnutrition aigüe (UNICEF, communication personnelle, 2013).

De plus, la mise en œuvre des services de nutrition à tous les niveaux est très dépendante des intrants provenant des partenaires techniques et financiers, principalement l'UNICEF et le Programme Alimentaire Mondial (PAM). Un autre problème observé par rapport aux intrants est le manque de gestion des stocks (par exemple, les fiches de stock sont habituellement mal remplies, il n'y a pas de suivi de la distribution des intrants, etc. (Ntakarutimana & Nimpagaritse 2013). Le résultat est que les FOSA souffrent de régulièrement de pénuries d'intrants thérapeutiques er nutritionnels. D'ailleurs, pour des raisons de contraintes budgétaires, le PAM a, en 2014, arrêté d'approvisionner en suppléments nutritionnels la majeure partie du pays, rendant ainsi les services de prise en charge de la malnutrition

aigüe modérée (MAM) ineffectifs. Un autre problème observé est le manque de connaissances et de compétences parmi les professionnels de la santé impliqués dans les activités de malnutrition (Ntakarutimana & Nimpagaritse 2013). En effet, même si des formations spécifiques ont été réalisées en 2010, on constate toujours, parmi les professionnels de santé et les agents de santé communautaires, un manque de connaissances relatives à la prévention et la prise en charge de la malnutrition (en partie du fait de rotations importantes du personnel) ainsi qu'une motivation réduite. Le manque de supervision des activités liées à la nutrition pourraient jouer un rôle.

1.2 Intervention évaluée

Description de l'intervention

Avec le soutien de la Banque Mondiale, le gouvernement Burundais renforce ce système, en introduisant des indicateurs liés à la nutrition dans le FBP existant : c'est une opportunité à la fois pour améliorer le statut nutritionnel des enfants et pour réaliser une analyse rigoureuse des forces et des faiblesses du FBP. En effet, l'intégration des services de nutrition dans le FBP est un défi dans la mesure où actuellement, la mise en œuvre des services de nutrition est fortement dépendante d'organisations externes comme l'UNICEF ou le Programme Alimentaire Mondial (PAM) qui fournissent les intrants nutritionnels. De plus, on constate que les services de nutrition sont négligés de par un manque de connaissances et de savoir-faire du personnel de santé et un manque de supervision des activités de nutrition. Aussi, l'introduction du FBP devrait permettre de réduire ces obstacles.

Afin de s'attaquer au défi de la malnutrition et de fournir des soins appropriés aux enfants malnutris, le MSPLS, avec le soutien de la Banque Mondiale, a décidé d'introduire des indicateurs liés à la nutrition dans le FBP existant. L'objectif est de renforcer la stratégie nationale d'intégration des services nutritionnels et de la rendre effective dans toutes les régions du Burundi. L'intervention FBP Nutrition consiste à introduire des incitations à trois niveaux (hôpital, centre de santé et niveau communautaire), à la fois sur le volet préventif et curatif, tous se focalisant sur les enfants de moins de 5 ans (cf. Tableau 2).

Tableau 2. Indicateurs conçus dans le cadre de l'intervention du FBP Nutrition

Niveau Communautaire	Les agents de santé communautaires reçoivent des bonus pour (1) le dépistage et la référence vers les centres de santé nutritionnels pour les cas de malnutrition aigüe, (2) l'organisation de séances de sensibilisation pour la promotion de régimes alimentaires sains et de bons comportements nutritionnels, et (3) l'organisation de cours de cuisine.
Niveau Centre de Santé	Les bonus seront donnés pour (1) le dépistage de malnutrition aigüe chez les enfants et la bonne prise en charge des cas de malnutrition aigüe (selon le type de centre de santé*), et (2) pour la promotion et le suivi de la croissance.
Niveau Hospitalier	Les bonus seront donnés pour (1) la bonne prise en charge des cas sévères de malnutrition aigüe avec complications médicales, et (2) le nombre de journées d'hospitalisation liées à la malnutrition aigüe.

Source: Note Technique (MSPLS 2013). Note:*En effet, il existe plusieurs sortes de centres de santé : la plupart des centres au Burundi ne fournissent pas de services pour les cas de malnutrition aigüe, certains fournissent des services de prise en charge des cas de malnutrition aigüe modérée, certains pour les cas de malnutrition aigüe sévère, et d'autres pour les cas de malnutrition aigüe modérée et sévère.

Une grille qualitative spécifique à la nutrition est prise en compte dans le calcul des bonus[2]. Ainsi, la qualité des activités de nutrition est évaluée chaque trimestre, de la même manière que pour les autres indicateurs de qualité. Un bonus ou un malus qualité est appliqué aux subventions reçues par les FOSA, selon leur score de qualité : si le score est supérieur à 80%, alors la FOSA reçoit un bonus équivalent au score multiplié par 25% des subventions FBP nutrition ; au contraire, si le score de qualité est inférieur à 60%, la FOSA est sujette à un malus allant de 10 à 25% du montant de subventions FBP Nutrition.

De plus, l'efficacité et la qualité des services de nutrition au niveau de la communauté sont évalués par des associations locales (ASLO). Cette évaluation est réalisée tous les six mois, en même temps que l'évaluation communautaire mise en œuvre pour le FBP général.

Le système de vérification est à peu près le même que pour les autres indicateurs FBP au Burundi. Les centres de santé rapportent mensuellement la quantité de services encouragés par le FBP effectivement réalisés. Ces rapports sont vérifiés puis validés au niveau provincial par les Comités Provinciaux de Vérification et de Validation (CPVV), et envoyés à la CT-FBP (i.e. niveau central), qui effectue les transferts de subventions vers les centres de santé. Avec l'introduction des indicateurs nutrition dans le FBP existant, la vérification et la validation des indicateurs nutrition au niveau du centre de santé sont réalisés par les CPVV, de la même manière que pour le FBP existant. Au niveau de la communauté, la pré-vérification de la quantité des services nutritionnels réalisés par les ASC est vérifiée par les centres

[2] En fait, cette grille spécifique a été mise en place en juillet 2015 et a été utilisée rétrospectivement pour évaluer la qualité des activités nutrition subventionnées à partir du mois d'avril 2015. Pour les mois précédents (janvier à mars 2015), c'est la grille qualité générale pré-existante qui a été utilisée.

de santé qui les supervisent, à l'aide des registres et rapports qui leurs sont rapportés. Le processus de vérification et validation est ensuite finalisé par le CPVV.

Il est prévu que le groupe de contrôle reçoive des compensations financières sous forme de dotations, équivalentes aux subventions FBP Nutrition reçues dans le groupe d'intervention (selon une moyenne pondérée prenant en compte le volume des activités et du personnel).

Toutes les parties prenantes (hôpitaux, centres de santé du groupe d'intervention, CPVV, bureaux sanitaires provinciaux et de districts, etc.) ont reçu une formation relative à cette nouvelle intervention, durant un atelier qui a pris place à Bujumbura en décembre 2014.

Mise en œuvre

L'intervention a démarré en Janvier 2015 aux niveaux des hôpitaux et des centres de santé. Tous les hôpitaux du Burundi disposant de services de stabilisation des cas de malnutrition aigüe sévère avec complications (SST) sont concernés. Au niveau centre de santé, seuls ceux du groupe d'intervention sont concernés. Parmi les 90 centres de santé nutritionnels[3] sélectionnés pour l'étude, la moitié, soit 45, sont dans le groupe d'intervention et reçoivent des financements basés sur la performance dans les activités liées à la malnutrition[4] ; il est prévu que l'autre moitié (le groupe de contrôle) reçoive des compensations financières équivalentes sous forme de dotations[5]. Par ailleurs, les centres de santé ne disposant pas de services de prise en charge de la malnutrition aigüe mais référant les cas de malnutrition aigüe vers les 45 centres du groupe d'intervention sont également éligibles à un financement lié à leur performance dans leurs activités de dépistage et référence de la malnutrition ainsi que de suivi de la croissance[6] ; il est prévu que les centres de santé référant vers les 45 centres du groupe de contrôle reçoivent également une compensation financière équivalente.

Le niveau communautaire du FBP Nutrition devait démarrer au cours de l'été 2015, mais cela a pris un certain retard compte tenu de l'instabilité politique, et il est maintenant prévu que cela démarre en octobre 2015[7]. Au préalable, les agents de santé communautaires seront regroupés de façon formelle en Groupement d'Agents de Santé Communautaire (GASC), puis formés aux différents indicateurs du FBP Nutrition. Seuls les GASC référant vers les 45 centres de santé nutritionnels du groupe d'intervention seront éligibles ; les GASC référant vers les autres centres ne recevront pas de compensation financière pour des raisons de contraintes budgétaires.

---

[3] Un centre de santé est dit « nutritionnel » s'il délivre un paquet complet de services de prise en charge de la malnutrition aigüe, c'est-à-dire un service de supplémentation nutritionnelle (SSN) et un service de thérapeutique ambulatoire (STA), selon le protocole de 2010. Cependant, le protocole de 2014, publié après l'enquête de référence dans les centres de santé, recommande que les services SSN soient désormais mis en œuvre au niveau de la communauté, et pas dans les centres de santé.

[4] Le montant versé aux FOSA tient compte de la qualité des activités nutrition à partir du deuxième trimestre 2015.

[5] A l'heure où nous écrivons ce rapport, aucun virement vers le groupe de contrôle n'a encore été fait. La formule de calcul a été arrêtée courant septembre 2015, et les ordres de virement pour les compensations des CDS du groupe de contrôle devraient être signés sous peu.

[6] Il est prévu que ces CDS dits "secondaires" établissent un contrat FBP Nutrition avec les CDS nutritionnels de l'étude (dits aussi CDS "primaires"); ces derniers effectuent alors les paiements des subsides FBP Nutrition auprès de ces CDS secondaires. Il nous est à l'heure actuelle difficile d'évaluer si ce processus est activé.

[7] Le processus a démarré et les modèles d'outils à signer ont déjà été envoyés.

Figure 2. Mise en œuvre du FBP Nutrition et de la compensation

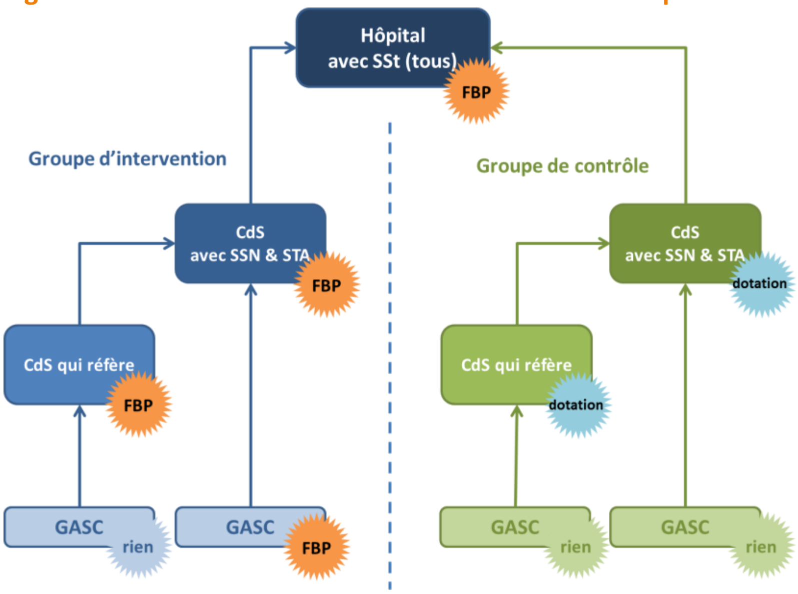

Source: auteurs.

1.3 Objectifs de l'étude

Théorie du changement

L'introduction d'indicateurs de nutrition dans le FBP devrait avoir un impact sur la performance des structures de santé de différentes manières. Nous avons identifié six chemins par lesquels le FBP a traditionnellement un impact sur la performance du système de santé, et nous pensons que chacun de ces chemins jouera un rôle dans la performance du FBP Nutrition en particulier :

1) **Chemin des revenus** : l'injection de ressources financières (conditionnée par la performance) aura un impact positif sur les services de nutrition, dans la mesure où cela permet au responsable de la structure de soins de recruter plus de personnel, de mieux équiper la structure, etc.

2) **Chemin des incitations** : si les tarifs du FBP Nutrition sont suffisamment élevés, les agents de santé seront motivés à accroître leur performance afin d'augmenter leur environnement de travail et leur salaire. Au niveau de la structure, l'effet sur les autres services reste incertain. Ils peuvent être négatifs pour les uns (i.e. si les agents en charge de la nutrition avaient l'habitude de s'occuper d'autres services et s'en détournent maintenant) et positifs pour d'autres (il y a des possibilités d'économies d'échelle, par exemple les activités de promotion et de suivi de la croissance peuvent être réalisées en même temps que les séances de vaccinations des enfants).

3) **Chemin de l'information** : à travers le contrat de FBP Nutrition (et les séances de formation), le personnel aura une meilleure compréhension sur ce qu'est la performance en matière de nutrition. De plus, les feedbacks provenant du système FBP les guideront et indiqueront leurs marges d'amélioration dans ces services. On suppose un effet positif sur les services de nutrition.

4) **Chemin de la supervision** : la vérification s'étendra aux services de nutrition, notamment sur requête des centres de santé ; aussi, la supervision devrait s'accompagner de conseils aux agents de santé sur les bonnes pratiques et de reconnaissance de leurs efforts.

5) **Chemin de la culture d'entreprise** : le FBP implique un changement d'un système passif à un système où l'initiative, la créativité et les réalisations d'objectifs sont valorisés. On suppose que cette voie est déjà active étant donné que le FBP est une politique nationale depuis trois ans.

6) **Chemin du système de santé** : on s'attend à certains effets sur le système de santé. Certains de ces effets proviendront des partenaires impliqués dans l'évaluation d'impact (par exemple, le MSPLS demandant à l'UNICEF et au PAM de fournir plus d'intrants nutritionnels). Une partie viendra des structures de santé (par exemple, pression du responsable d'une FOSA auprès du PRONIANUT pour qu'il devienne un fournisseur fiable et rapide). On attend également que les agents de santé communautaires réfèreront les enfants mal nourris aux centres de santé et que ces centres réfèreront à leur tour les enfants en situation de malnutrition sévère aux hôpitaux de référence.

Figure 3: Chaine des résultats liés à l'introduction de la Nutrition dans le FBP

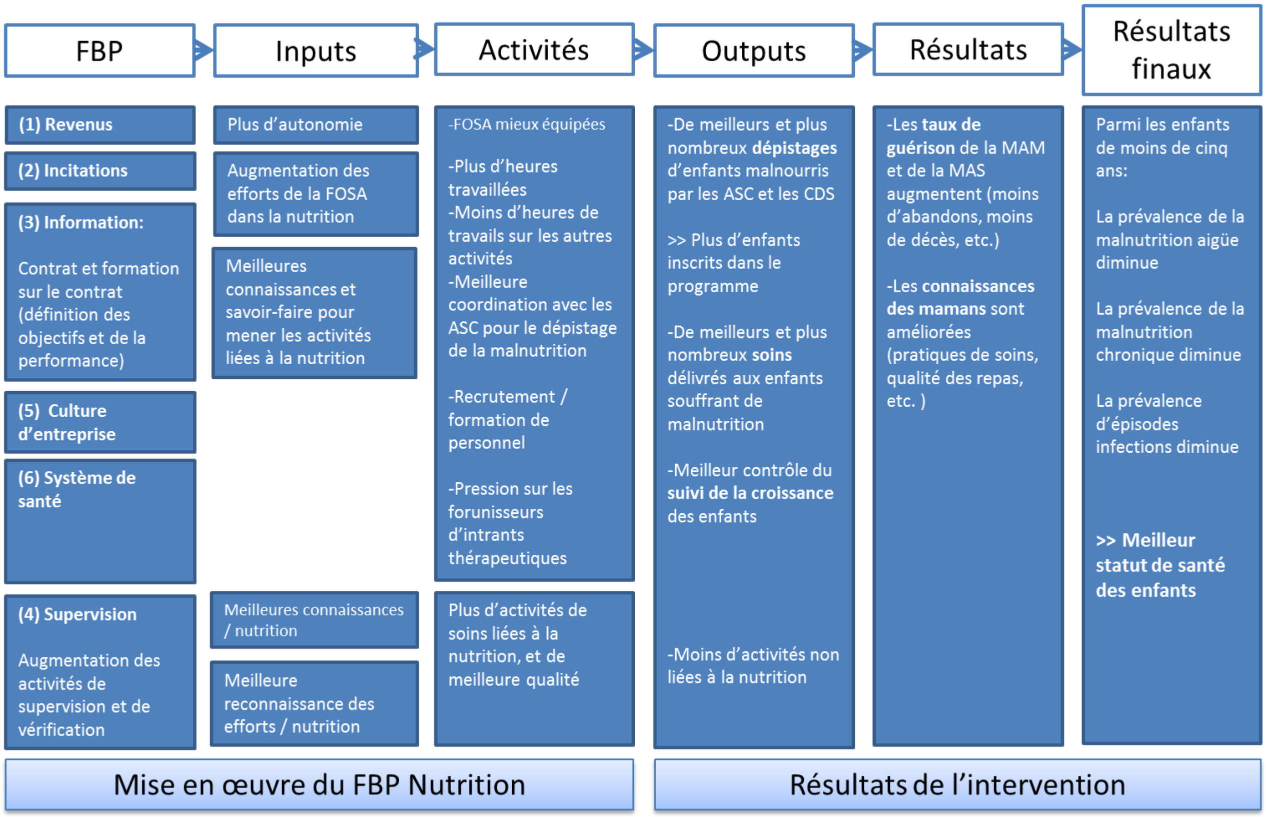

Source: Auteurs.

Tableau 3. Chemins à considérer dans les groupes d'intervention et de contrôle

	Groupe d'intervention	Groupe de contrôle
(1) Revenus	✓✓✓	✓✓✓[a]
(2) Incitations	✓✓✓	
(3) Information	✓✓✓	✓[b]
(4) Supervision	✓✓✓	✓[c]
(5) Culture d'entreprise	✓[d]	
(6) Système de santé	✓✓✓	✓[e]

Notes: (a) Les structures de santé du groupe de contrôle recevront une compensation financière additionnelle correspondant à la moyenne pondérée des subventions reçues par le groupe d'intervention. (b) Comme les centres de santé du groupe de contrôle auront été mis au courant de leurs performances en termes de nutrition (lors d'un atelier avec les 90 centres nutritionnels de l'étude), ils pourront ressentir un besoin de formation également, s'ils anticipent en effet une généralisation du FBP Nutrition. (c) Dans un district où il y a des structures appartenant au groupe d'intervention et de contrôle, il est possible que l'équipe de superviseurs du district transfère quelques bonnes pratiques observées dans un centre de santé du groupe d'intervention à un centre de santé du groupe de contrôle. (d) Etant donné que le FBP est déjà en place depuis 2010, on estime que la culture d'entreprise l'est également, au niveau du centre de santé dans son ensemble ; le FBP Nutrition peut cependant agir sur la culture d'entreprise des services spécifiques à la nutrition. (e) Certains effets sur le système de santé pourront affecter les centres de santé du groupe de contrôle.

Questions et objectifs de l'évaluation d'impact

L'objectif principal de l'évaluation d'impact est d'évaluer l'impact de l'introduction des indicateurs nutrition dans le système FBP, sur (i) les taux de malnutrition aigüe et chronique, mesurés au niveau de la communauté, ainsi que (ii) sur la performance des services de prévention et prise en charge de la malnutrition, à travers principalement les taux de guérison de la malnutrition aigüe, mesurés au niveau des centres de santé nutritionnels.

L'impact de l'intervention n'est pas garanti : le FBP est déjà en place au Burundi, la malnutrition est un problème multisectoriel, et on dispose à ce jour de très peu d'expérience opérationnelle sur les façons dont le système FBP devrait être monté pour s'occuper effectivement de la malnutrition. Par conséquent, la recherche incorpore également des études secondaires (*work packages*) qui permettront d'identifier les causes possibles des succès ou échecs du programme de FBP Nutrition.

Cette étude suit un design d'essais randomisés contrôlés par grappes (ou Cluster-RCT). Les principales questions sont aux niveaux de 1) la communauté, et 2) du centre de santé :

(1) Est-ce que l'introduction des critères axés sur les activités préventives et curatives de lutte contre la malnutrition dans le système FBP actuel se traduit par une réduction des taux de malnutrition aigüe et chronique dans la communauté ? En une meilleure équité des résultats liés à la nutrition ? Est-ce que l'intervention génère des externalités sur d'autres résultats de santé ?

(2) Est-ce que l'introduction des critères axés sur les activités préventives et curatives de lutte contre la malnutrition dans le système FBP actuel se traduit par de meilleurs résultats au niveau des centres de santé, par exemple en une meilleure gestion des cas de malnutrition (meilleurs taux de guérison, périodes de traitement plus courtes, etc.) ?

Perspectives politiques

La Banque Mondiale a recruté une équipe de l'Institut de Médecine Tropicale d'Anvers (IMT) pour soutenir le gouvernement et l'équipe de la BM dans la mise en œuvre de cette évaluation d'impact. Le principal objectif de cette étude est d'aider le MSPLS à (i) évaluer l'introduction graduelle des services de nutrition dans le programme FBP et à (ii) mettre à l'échelle cette expérience.

Cette évaluation d'impact permettra d'aider le dialogue politique national, mais elle fournira également des informations clé pour d'autres pays disposant déjà d'un système FBP et confrontés à des problèmes de malnutrition. Si les résultats de l'évaluation d'impact s'avéraient positifs, cela pourrait entrainer d'énormes implications dans la lutte mondiale contre la malnutrition.

Structure du rapport

La deuxième section de ce rapport décrit la méthodologie utilisée dans l'étude, détaillant ainsi la conceptualisation, la randomisation et l'échantillonnage, ainsi que les variables à utiliser pour l'analyse, les instruments de collecte de donnée et le stockage des données. La troisième section explique dans quelle mesure les données collectées sont représentatives du pays, et si celles-ci sont comparables, i.e. plus ou moins similaires à d'autres enquêtes réalisées au Burundi. La quatrième section décrit les résultats issus des enquêtes de référence, au niveau des FOSA puis de la communauté. La cinquième section explore la validité interne de l'étude, en révisant les puissances de calcul, discutant les menaces et s'assurant que les deux groupes de contrôle et d'intervention sont similaires et comparables. La sixième section discute les résultats et la septième section propose quelques recommandations liées au fonctionnement du système de santé au Burundi.

2. Méthodologie

2.1 Design de l'étude

Cette étude suit un design d'essais randomisés contrôlés par grappes (ou Cluster-RCT) avec le centre de santé[8] comme premier niveau d'échantillonnage et les sous-collines (unité administrative au Burundi) comme second niveau. Les 90 centres de santé sélectionnés ont été alloués aléatoirement soit à un groupe de contrôle (45) soit à un groupe d'intervention (45). Autour de chacun des centres de santé, six sous-collines sont sélectionnées de façon aléatoire ; au sein de chaque sous-colline, 12 enfants de 6-23 mois et leurs ménages sont enquêtés.

2.2 Randomisation et taille d'échantillon

Au niveau de la communauté

La taille de l'échantillon a été calculée sur base de la plus petite différence dans le résultat principal qui peut être considéré comme significative dans le groupe d'intervention comparé au groupe de contrôle - ici, on considère une réduction du taux de malnutrition aigüe parmi les enfants de 6-23 mois[9] de 25%. Si on fait l'hypothèse que l'intervention résultera en une réduction du taux de malnutrition aigüe parmi ces enfants de 10% à 7.5% (selon l'enquête EDS 2010), et en considérant que 65 enfants âgés de 6-23 mois seront enquêtés dans l'aire de responsabilité de chaque centre de santé, 90 centres de santé sélectionnés aléatoirement sont nécessaires pour une erreur de type alpha de 5%, une erreur de type beta de 20% et une corrélation intra-grappes de 0.25[10]. Le nombre d'enfants à enquêter par centre de santé est augmenté à 72 afin de permettre que certaines données soient manquantes ou incomplètes. Ainsi le nombre total d'enfants de 6-23 mois et leurs ménages à enquêter pour chaque vague d'enquête est de 6,480 (référence de 2014 et suivi de 2016).

La sélection des centres de santé invités à participer à l'étude a été réalisée par sélection aléatoire simple (sur base d'un algorithme aléatoire sous STATA), parmi les 193 centres de santé éligibles, i.e. les centres de santé fournissant les services de prise en charge de la malnutrition aigüe modérée et de la malnutrition aigüe sévère sans complications (appelés respectivement les services SSN et STA ; liste obtenue auprès du PRONIANUT et de l'UNICEF en 2013). Les 90 centres de santé sélectionnés ont ensuite été appariés selon des paramètres essentiels d'organisation et de fonctionnement en relation

---

[8] Cette recherche au niveau de la FOSA se concentre sur les centres de santé (et non les hôpitaux), car le niveau de soins primaires est un point d'engorgement connu en ce qui concerne le diagnostic et le traitement de la malnutrition aigüe. Une évaluation séparée pourrait être réalisée sur les programmes de SST (i.e. au niveau de l'hôpital) au Burundi, mais étant donné que ces unités sont peu nombreuses, et que les patients de ces unités sont également peu nombreux, les résultats n'auraient pas une puissance de calcul suffisante en comparaison des possibilités de tailles d'échantillonnage à travers des enquêtes dans les centres de santé.

[9] On se concentre sur ce groupe d'âge car c'est là qu'on suppose que l'impact sera le plus important. C'est aussi le groupe d'âge pour lesquels les mères sont les plus motives pour respecter le programme.

[10] Calculé selon la formule de Hayes & Bennett (1999) : c (nombre de grappes) = $1+ (z_{\alpha/2} + z_{\beta})^2[\pi_0 (1-\pi_0)/n + \pi_1(1-\pi_1)/n+ k_m^2 (\pi_0^2+\pi_1^2)]/(\pi_0-\pi_1)^2$, avec n=nombre d'enfants par grappe, et k_m le coefficient de variation entre grappes au sein des paires en l'absence d'intervention. K_m est fixé à 0.25, ce qui est plutôt conservateur comme estimation en l'absence d'information détaillée sur le Burundi (les données EDS ne sont pas examinées au niveau du centre de santé).

avec les résultats (activité SSN, volume d'activité, population dans l'aire de santé, taux de guérison parmi les enfants malnutris) tels que mesurés durant l'enquête de référence. Au sein de chacune de ces 45 paires, un centre a été alloué au groupe d'intervention tandis que l'autre l'était au groupe de contrôle ; l'allocation aléatoire a été effectuée sous forme de loterie organisée durant un atelier en décembre 2014.

Un système de sélection aléatoire simple devrait être utilisé pour sélectionner les enfants à enquêter dans les aires de responsabilité des 90 centres de santé. Cependant, une telle approche est difficile à mettre en œuvre, car cela peut être très demandeur en termes de ressources. L'alternative choisie a été de sélectionner des grappes d'individus, i.e. des enfants vivant proches les uns des autres. Six grappes de 12 enfants ont été sélectionnées aléatoirement, dans l'aire de responsabilité de chaque centre de santé. Cela a pu être réalisé sans étendre la taille de l'échantillon étant donné le coefficient de correlation intra-classe (ou ICC) élevé considéré dans le calcul.

Au niveau des centres de santé

Afin de mesurer la performance des services nutritionnels des centres de santé (et notamment de calculer les taux de guérison), 12 fiches individuelles de suivi sont sélectionnées aléatoirement et retranscrites par type de service (SSN ou STA) et par centre de santé. Ce nombre a été calculé sur base de la plus petite différence dans le résultat principal qui peut être considéré comme significative dans le groupe d'intervention comparé au groupe de contrôle - ici, on considère une hausse du taux de guérison de 80% à 90%, avec une erreur de type alpha de 5%, une erreur de type beta de 20%, et une corrélation intra-grappes de 0.15 (selon la formule de Hayes et Bennett, 1999 ; Kaiser et al. 2006).

2.3 Variables pour l'analyse des données

Au niveau de la communauté

L'étude se focalise sur la prévalence de la malnutrition aigüe parmi la population des enfants âgés de 6 à 23 mois comme résultat final. D'autres variables sont considérées. Les indicateurs de résultats à collecter et à analyser sont décrits dans le tableau suivant:

Tableau 4. Variables de résultats à collecter et analyser

Principal résultat	- Prévalence de la malnutrition aigüe (z-score du poids pour taille<-2)<br>- Z-scores du poids-pour-taille et de taille-pour-âge, et périmètre brachial<br>- Prévalence du retard de croissance (z-score de taille-pour-âge<-2)
Résultats secondaires	- Prévalence des épisodes infectieux (diarrhée, infection des voies respiratoires supérieures, fièvre)

Source: Auteurs.

Au niveau des centres de santé

Au niveau du centre de santé, nous souhaitons comprendre si l'introduction de critères nutrition dans le programme de FBP existant apportera de meilleurs résultats au niveau du centre de santé, i.e. une meilleure prise en charge des cas de malnutrition (taux de guérison, durée de périodes de traitement, etc.). Les indicateurs de résultats à collecter et à analyser au niveau de la structure sont indiqués dans le tableau suivant :

Tableau 5. Variables de résultats à analyser au niveau de la structure de soins

Résultat principal	- Taux de guérison de la MAM et de la MAS
Résultats secondaires	- Durée de traitement des cas de MAM et de MAS<br>- Taux d'abandons<br>- Taux de rechute<br>- Pourcentage de cas suivis régulièrement et référés au centre de santé

Source: Auteurs.

2.4 Instruments de collecte et d'assurance qualité

2.4.1 Enquête au niveau des centres de santé

L'organisation de l'enquête de référence au niveau des centres de santé a été confiée à l'Institut National de Santé Publique (INSP). Au total, 24 enquêteurs, deux superviseurs et un gestionnaire ont été mobilisés pour la formation et l'enquête sur le terrain, sur une période couvrant au total quatre semaines en septembre et octobre 2014 (cf. rapport d'enquête, INSP, 2014).

Quatre domaines sont à évaluer à partir de l'enquête au niveau des centres de santé :

(A) L'évaluation des dossiers médicaux individuels issus des services de nutrition

(B) Les aspects organisationnels liés à la fourniture des soins de santé

(C) La qualité des services de santé

(D)Les connaissances et compétences des agents de santé

Ceci était évalué à travers un ou plusieurs questionnaires (suivant le schéma qui suit), dont la méthodologie est traitée ci-dessous.

Figure 4. Structure des questionnaires de l'enquête au niveau des centres de santé

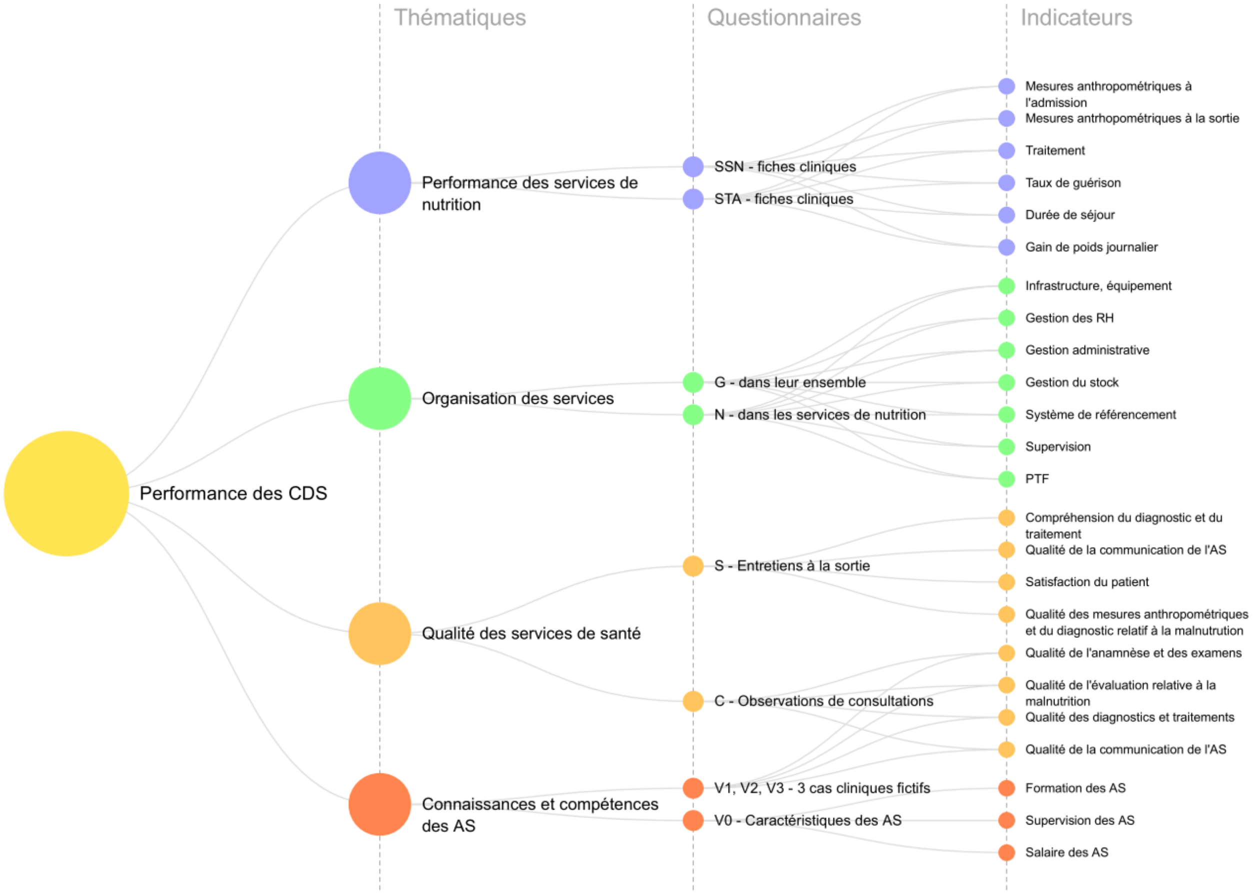

(A) Evaluation des dossiers médicaux individuels issus des services de nutrition

<u>Questionnaire SSN – Retranscription des fiches individuelles de suivi SSN</u>

Objectif du questionnaire

L'objectif principal de cet instrument est d'évaluer les indicateurs de performance des SSN au Burundi ; un objectif secondaire est d'évaluer la qualité de la documentation relative aux patients.

Les données collectées permettent de calculer les taux de guérison, la durée moyenne des traitements, et autres indicateurs de performance du SSN, afin de pouvoir répondre à la question principale de l'étude. Les données anthropométriques à l'admission et à la sortie du programme sont collectées afin d'évaluer si les critères d'admission et de sortie (pour guérison ou référence) sont respectés.

Contenu et mise en œuvre

Dans chaque centre de santé, a été effectuée une retranscription anonymisée de 12 fiches individuelles sélectionnées de façon aléatoire parmi les fiches de tous les enfants de moins de cinq ans ayant

participé à un programme de service de supplémentation nutritionnelle (SSN) au cours des six derniers mois[11].

Le questionnaire SSN était à compléter sur un smartphone à travers un formulaire électronique (développé avec le software Open Data Kit, ODK), qui suivait la structure de la fiche individuelle de suivi à retranscrire. L'agent de santé (AS) en charge de ce service était informé des objectifs et du contenu de ce questionnaire et fournissait les fiches individuelles. L'enquêteur devait ensuite sélectionner aléatoirement 12 fiches parmi les cas éligibles (c'est-à-dire des enfants de moins de cinq ans et ayant quitté le service durant la période de six mois spécifiée).

Dans de nombreux cas, il n'y avait pas de fiches individuelles au centre de santé, ou alors elles étaient mal remplies. Dans ce cas, les enquêteurs devaient chercher l'information à partir des registres du service SSN. Néanmoins, généralement, les registres ne fournissent pas l'information sur les traitements systématiques ou nutritionnels administrés aux patients ; mais normalement les informations sur les mesures anthropométriques à l'admission et à la sortie ainsi que sur le résultat du programme y sont reportées.

Les enquêteurs ont reçu une formation durant quatre jours sur les objectifs de ce questionnaire et sur comment remplir correctement le formulaire sur le smartphone. La formation a inclus des cours interactifs et des exercices pratiques avec de vrais fiches individuelles de suivi et registres. Ils ont tous été évalués à partir d'un examen écrit et pratique durant le cinquième jour de la formation. Afin d'assurer la qualité des données collectées, un LQAS a été réalisé par deux superviseurs durant l'enquête.

Questionnaire STA - Retranscription des fiches individuelles de suivi STA

Objectif du questionnaire

L'objectif principal de cet instrument est d'évaluer les indicateurs de performance des STA au Burundi ; un objectif secondaire est d'évaluer la qualité de la documentation relative aux patients.

Les données collectées permettent de calculer les taux de guérison, la durée moyenne des traitements, et autres indicateurs de performance du STA, afin de pouvoir répondre à la question principale de l'étude. Les données anthropométriques à l'admission et à la sortie du programme sont collectées afin d'évaluer si les critères d'admission et de sortie (pour guérison ou référence) sont respectés.

Contenu et mise en œuvre

Dans chaque centre de santé, a été effectuée une retranscription anonymisée de 12 fiches individuelles sélectionnées de suivi de façon aléatoire parmi les fiches de tous les enfants de moins de cinq ans ayant participé à un programme de service thérapeutique ambulatoire (STA) au cours des six derniers mois[12].

---

[11] Cependant les services SSN n'étaient pas fonctionnels en 2014 à cause d'une rupture d'approvisionnement des intrants nutritionnels (sauf dans les provinces de Ruyigi et Rutana) ; la période considérée pour les fiches individuelles a alors glissé vers Mars-Septembre 2013, plutôt que Février-Août 2014.

Le questionnaire STA se complétait à travers un formulaire électronique, développé sur smartphone (en utilisant le software Open Data Kit, ODK), qui suivait la structure de la fiche individuelle à retranscrire. L'AS en charge de ce service était informé des objectifs et du contenu de ce questionnaire et fournissait les fiches individuelles. L'enquêteur devait ensuite sélectionner aléatoirement 12 fiches parmi les cas éligibles (c'est-à-dire des enfants de moins de cinq ans et ayant quitté le service durant la période de six mois spécifiée).

Dans de nombreux cas, il n'y avait pas de fiches individuelles au centre de santé, ou alors elles étaient mal remplies. Dans ce cas, les enquêteurs devaient chercher l'information à partir des registres du service STA. Néanmoins, généralement, les registres ne fournissent pas l'information sur les traitements systématiques ou nutritionnels administrés aux patients ; mais normalement les informations sur les mesures anthropométriques à l'admission et à la sortie ainsi que sur le résultat du programme y sont reportées.

Les enquêteurs chargés de retranscrire les fiches STA étaient les mêmes que ceux chargés de retranscrire les fiches SSN. De la même manière que pour la retranscription des fiches SSN, ils ont reçu une formation sur les objectifs de ce questionnaire et sur comment remplir correctement le formulaire sur le smartphone. La formation a inclus des cours interactifs et des exercices pratiques avec de vrais fiches individuelles et registres. Ils ont tous été évalués à partir d'un examen écrit et pratique durant le cinquième jour de la formation. Afin d'assurer la qualité des données collectées, un LQAS a été réalisé par deux superviseurs durant l'enquête.

(B) Aspects organisationnels des services de santé

Questionnaire G – Aspects organisationnels du centre de santé

Objectif du questionnaire

L'objectif principal de ce questionnaire est de collecter des informations sur les aspects organisationnels du centre de santé. Ces variables plus contextuelles seront notamment utilisées pour capturer les facteurs confondants dans les analyses statistiques.

Contenu et mise en œuvre

Ce questionnaire est administré au titulaire ou au titulaire adjoint de chacun des 90 centres de santé. Ceux-ci sont informés au préalable de l'enquête et des appels sont faits pour s'assurer de la présence d'un de ces deux responsables le jour de l'enquête. Dans deux cas cependant, ils étaient tous deux absents et le questionnaire a été administré à un autre membre du personnel.

Il s'agit d'un questionnaire avec cinq sections. La première section concerne des informations relatives à l'organisation et l'infrastructure générales du centre de santé (propriétaire, source d'eau, équipement). La deuxième section étudie le système d'information sanitaire (aire de responsabilité, relations avec les

---

[12] Afin d'être cohérent avec le questionnaire SSN, la période considérée pour les fiches individuelles STA est la même, à savoir Mars-Septembre 2013, plutôt que Février-Août 2014 (sauf dans les provinces de Ruyigi et Rutana où le SSN fonctionnait).

autres centres de santé et les agents de santé communautaires, fourniture du paquet minimum d'activités). La troisième section est liée à la gestion du centre de santé. Ces trois sections ont été administrées à l'aide des smartphones et du software ODK.

Les deux autres sections ont été administrées sur papier. L'une concerne les ressources humaines, nécessitant d'écrire une liste de tout le personnel avec des informations sur leurs contrats et leur éducation. La dernière section note les partenaires techniques et financiers (PTF) avec lesquels le centre de santé collabore.

Les enquêteurs administrant ce questionnaire ont reçu une formation sur quatre jours sur ses objectifs et sur comment remplir correctement le formulaire sur le smartphone et papier. Ils ont tous été évalués à partir d'un examen écrit et pratique durant le cinquième jour de la formation.

Questionnaire N – Aspects organisationnel des services liés à la malnutrition

Objectif du questionnaire

L'objectif principal de ce questionnaire est de collecter des informations sur les aspects organisationnels des services de prévention et de prise en charge de la malnutrition. Cela a pour but de comprendre l'environnement dans lequel les activités de détection de la malnutrition, promotion de la croissance et prise en charge de la malnutrition sont réalisées. Ces variables seront également utilisées pour capturer les facteurs confondants dans les analyses statistiques.

Contenu et mise en œuvre

Ce questionnaire est administré au responsable du service de nutrition de chacun des 90 centres de santé. Ceux-ci sont informés au préalable de l'enquête et des appels sont faits pour s'assurer de sa présence le jour de l'enquête. Pour chaque question, excepté le module relatif au personnel, il y a une observation par centre de santé.

Ce questionnaire comporte huit sections. La première est liée aux services de suivi et promotion de la croissance et de prévention et prise en charge de la malnutrition. La deuxième section liste le personnel travaillant dans ces services, note leurs responsabilités ainsi que leur formation. La troisième composante est liée aux locaux utilisés pour ces activités ainsi que pour les intrants nutritionnels (CSB, huile, sucre et Plumpy Nut). Les quatrième, cinquième et sixième sections collectent des informations sur le système d'information sanitaire lié à la malnutrition, les références, les ASC et les supervisions. La septième section collecte des informations sur les PTF impliqués dans chacune des activités liées à la malnutrition. La huitième section est liée à la gestion des stocks des intrants thérapeutiques et nutritionnels.

Les enquêteurs administrant ce questionnaire ont reçu une formation sur quatre jours sur ses objectifs et sur comment remplir correctement le formulaire sur le smartphone et papier. Ils ont tous été évalués à partir d'un examen écrit et pratique durant le cinquième jour de la formation.

(C) Qualité des services de santé

Questionnaire C – Observation de consultations

Objectif du questionnaire

Le principal objectif du questionnaire C est d'observer six consultations curatives d'enfants de 6-59 mois, dans les centres de santé. L'objectif est d'évaluer la qualité des consultations, et plus spécifiquement en relation avec le respect du manuel de prise en charge intégrée des maladies de l'enfants (PCIME, WHO, 2014) en place au Burundi, et relativement à l'évaluation nutritionnelle de l'enfant.

Contenu

Il s'agit d'une grille d'observation, où l'enquêteur (observateur) doit cocher les questions posées, examens réalisés, etc., par l'agent de santé effectuant la consultation, et indiquer les réponses et résultats des examens le cas échéant.

La liste de questions relatives à l'anamnèse est basée sur la PCIME et les directives nationales relatives à la malnutrition (MSPLS, 2010). Elles couvrent les sujets basiques qui devraient être discutés lors d'une consultation pédiatrique.

L'anamnèse nutritionnelle est essentielle dans les consultations pédiatriques mais généralement négligée par les AS dans la plupart des centres de santé primaires des pays à revenus faibles (Oladele et al. 2012; Valadez et al. 1996). Les questions ont été inclues ici afin d'observer si c'était le cas dans les centres de santé au Burundi, mais aussi pour obtenir une référence et observer si l'introduction du FBP pour la nutrition aura un impact sur la fréquence de présence de ces questions en consultation curative lors de l'enquête finale. En effet, c'est lors de ces consultations curatives, c'est-à-dire pour des problèmes de santé a priori non liés, que la détection de malnutrition peut être réalisée, à temps, afin que le traitement et la prévention soient possibles.

Un processus similaire a été utilisé pour les examens physiques ; la liste d'examens proposés dans la grille d'observations concerne des examens basiques (non spécialisés et non compliqués) et suit les recommandations de la PCIME. De plus, des paramètres spécifiques à la nutrition ont été ajoutés pour les même raisons qu'indiqué ci-dessus pour les questions de nutrition. Il s'agit des examens suivants : poids, taille, PB, présence d'œdème, calcul du z-score poids-pour-taille, test d'appétit, et évaluation de la courbe de croissance. Il y avait également une section pour les tests de laboratoire, où l'enquêteur pouvait écrire librement quels tests avaient été proposés par l'AS (dans le cas où ces tests n'étaient pas dans la liste).

Les sections suivantes concernent le diagnostic puis le traitement. Les diagnostics les plus communs et importants (en termes de sévérité et de traçabilité) ont été inclus dans la liste d'options, avec une option additionnelle 'autres' au cas où le diagnostic de l'AS n'était pas présent dans cette liste. Les traitements ont été listés sur la même base, les principaux traitements pertinents pour les diagnostics ayant été inclus. A la toute fin de la grille d'observation, des questions ouvertes étaient proposées pour évaluer la qualité de la communication et les conseils donnés par l'AS à l'accompagnant de l'enfant.

Mise en œuvre

Dans chaque centre de santé, six observations de consultations curatives d'enfants âgés de 6-59 mois ont été effectuées[13].

L'intégralité de ce questionnaire est remplie sur papier. Les enquêteurs ont au préalable reçu une formation de quatre jours, pour comprendre les objectifs du questionnaire et comment le remplir correctement. Le principal message était de comprendre que leur simple présence en consultation allait amener un biais dans le comportement de l'AS (connu comme l'effet de Hawthorne), et qu'il fallait essayer de minimiser ce biais le plus possible. La moitié des enquêteurs du questionnaire C avaient une expérience dans la prise de mesures anthropométriques durant une enquête et tous avaient une expérience pour évaluer la nutrition de par leur expérience passée dans le secteur médical. Le groupe était principalement composé d'infirmiers. La formation contenait des cours interactifs, des exercices de groupes, des tutoriels vidéo et des jeux de rôle. Un examen écrit et un examen pratique durant le cinquième jour de la formation ont permis d'évaluer leur compréhension et savoir-faire.

Sur le terrain, les enquêteurs disposaient d'un texte pour expliquer les objectifs du questionnaire à l'AS observé, et pour souligner qu'il ne s'agissait pas d'un test et que leurs observations étaient de nature impartiale. Les accompagnants des enfants ont également reçu des explications s'agissant des objectifs du questionnaire, au début de chaque consultation. Le consentement écrit devait être obtenu auprès de l'AS et de l'accompagnant avant chaque observation.

Pour la partie relative à l'anamnèse, les enquêteurs ont reçu comme instruction de cocher lorsqu'une question de la liste était posée, puis d'indiquer la réponse de l'accompagnant. Pour l'examen physique, ils devaient noter si un examen en particulier était réalisé, puis noter le résultat de l'examen : si celui-ci n'était pas dit assez fort lors de la consultation par l'AS effectuant l'examen, l'enquêteur devait retrouver le résultat en faisant attention de ne pas déranger le cours de la consultation. Par exemple si l'AS avait pris le pouls de l'enfant sans toutefois le dire tout haut, l'enquêteur pouvait attendre la fin de la consultation pour demander à l'AS quel était le pouls.

Concernant les mesures anthropométriques, les enquêteurs devaient noter si l'AS avait pris les mesures spécifiques lui-même, et/ou s'il avait fait référence à / discuté ce type de mesures avec l'accompagnant durant la consultation. Si les enquêteurs notaient que l'AS avait mesuré ou fait référence à l'une de ces mesures, sans donner la valeur de cette mesure, ils devaient alors attendre la fin de la consultation pour demander à l'accompagnant de voir le carnet de santé de l'enfant où se trouvaient les mesures. La même méthodologie a été utilisée lorsque des tests de laboratoire ont été prescrits.

Dans le cas où l'AS n'explique pas le diagnostic à l'accompagnant, l'enquêteur devait demander à l'AS quel était le diagnostic et le plan de traitement, mais seulement quand l'accompagnant et l'enfant avaient quitté la consultation, afin de ne pas influencer les décisions ou actions prises. Cela pouvait parfois impliquer d'attendre que l'accompagnant soit revenu du laboratoire avec les résultats des tests.

[13] Le plan était d'observer trois consultations curatives effectuées par deux agents de santé ; dans le cas où un seul agent de santé était affecté aux consultations curatives pédiatriques le jour de l'enquête, celui-là seul était observé, pour six consultations au total.

Chaque accompagnant recevait un numéro, de façon à ce que les enquêteurs puissent les identifier correctement durant leur journée passée au centre de santé.

Questionnaire S – Entretien à la sortie

Objectif du questionnaire

Suite à cette consultation observée, il était demandé aux enfants et leurs accompagnants de répondre à un entretien à la sortie, administré par un autre enquêteur. Le premier objectif de ce questionnaire était d'obtenir des informations de la part des accompagnants sur leur opinion sur le contenu et la qualité de la consultation, et d'évaluer leur compréhension de l'information qui leur était donnée. Le second objectif était de documenter les mesures anthropométriques des enfants prises par les agents du centre de santé, puis de répéter ces mesures par les enquêteurs eux-mêmes.

Contenu

La première partie du questionnaire S était une suite de questions permettant d'établir le statut socio-économique ainsi que le niveau d'éducation de l'accompagnant de l'enfant; il lui était également demandé quelle était son lien avec l'enfant qu'il accompagnait. La deuxième partie permettait de voir si l'accompagnant avait compris le diagnostic de l'enfant ainsi que le plan prévu pour le soigner. Il y avait également une question pour savoir s'il avait reçu des conseils diététiques, et quels étaient-ils, le cas échéant. Les questions suivantes évaluaient leur niveau de satisfaction avec la consultation et quelles étaient leurs suggestions pour améliorer les services. Finalement, des mesures anthropométriques des enfants étaient prises, avec le matériel du centre de santé et le matériel de l'enquête.

Mise en œuvre

Le questionnaire était rempli sur smartphone à l'aide du logiciel ODK pour une partie (caractéristiques générales et mesures anthropométriques), et sur papier pour les questions relatives à l'appréciation de la consultation. L'enquêteur qui réalisait les observations de consultations (questionnaire C) donnait à l'accompagnant une carte avec un numéro d'identifiant à la fin de la consultation. Les accompagnants et leurs enfants devaient ensuite attendre d'être appelés par l'enquêteur qui administrait le questionnaire S (mais seulement après qu'ils aient été au laboratoire si des tests avaient été prescrits) et à la pharmacie (si des médicaments avaient été prescrits). L'enquêteur devait s'assurer avant de commencer l'entretien que le séjour au centre de santé de l'accompagnant et de l'enfant était terminé (ce qui inclut de retourner voir l'AS s'ils veulent discuter plus amplement les résultats et le traitement).

Pour les mesures anthropométriques, l'enquêteur devait d'abord regarder, dans le carnet de santé de l'enfant ou un autre document approprié, le poids, la taille, le PB, le WHZ ainsi que la courbe de croissance s'ils étaient documentés, et noter ces résultats s'ils l'étaient. Ces mesures étaient ensuite répétées avec l'équipement du centre de santé, mais aussi avec l'équipement de l'enquête (balance mère-enfant SECA 876, toise UNICEF et mètre ruban SECA 212). Les enquêteurs devaient aussi évaluer la présence d'œdèmes.

Les enquêteurs chargés de ce questionnaire ont suivi une formation de quatre jours pour bien s'entretenir avec les accompagnants, remplir le questionnaire à la fois sur le smartphone et sur le papier, et sur la prise des mesures anthropométriques. Le cinquième jour était consacré à l'examen écrit

et pratique. Un tiers des enquêteurs de ce questionnaire avaient eu une expérience dans le passé pour la prise des mesures anthropométriques durant une enquête.

Dans les cas où les mesures anthropométriques prises par l'enquêteur indiquaient un problème de malnutrition chez l'enfant, l'enquêteur devait raccompagner l'enfant vers l'AS qui l'avait eu en consultation pour discuter du résultat. Ce processus était facilité par un message automatique apparaissant sur le smartphone de l'enquêteur une fois que toutes les mesures étaient saisies. La responsabilité ultime pour prescrire un traitement revenait à l'AS.

Un consentement écrit était obtenu auprès des accompagnants des enfants avant de commencer l'entretien relatif au questionnaire S.

(D) Connaissances et compétences des agents de santé

Questionnaire V – Vignettes cliniques

Objectif du questionnaire

L'objectif principal des vignettes est d'évaluer les connaissances et compétences des agents de santé responsables des consultations et services pédiatriques dans les centres de santé.

Contenu

Les connaissances et compétences des agents de santé sont évaluées à partir de trois vignettes cliniques (consultations factices), proposées aux deux agents de santé ayant été observés en consultation durant la journée de travail.

Chaque vignette correspond à une mise en situation clinique. Un motif de consultation de 'cas imaginaire' est proposé, et l'agent de santé peut poser toutes les questions nécessaires, faire des examens physiques et proposer de faire des examens complémentaires pour arriver à un diagnostic et proposer une prise en charge. L'enquêteur, qui joue le role de l'accompagnant de l'enfant, donne les réponses à toute requêtede l'AS (questions et examens), dont le but est d'arriver à un diagnostic et une prise en charge corrects du cas. Le tableau ci-après propose une synthèse des signes positifs, diagnostics et plans de prise en charge attendus pour chacun des trois cas.

Tableau 6. Résumé des vignettes cliniques

Vignette	Signes	Diagnostic	Traitement
**n°1**	▪ garcon de 13 mois<br>▪ tachypnéique<br>▪ tempéraure: 38.6°c<br>▪ pâleur<br>▪ même poids que trois mois avant<br>▪ z-score à -3<br>▪ hémoglobine <9,3g/dl	**MAS compliquée** avec pneumonie, anémie et retard de croissance	▪ antibiothérapie (amoxycilline) pour la pneumonie mais aussi comme traitement systématique<br>▪ antipyrétique (paracétamol) car présence de fièvre<br>▪ acide folique pour anémie et comme traitement systématique<br>▪ albendazole en traitement systématique<br>▪ référer l'enfant au SST<br>▪ compléter la vaccination<br>▪ donner des conseils diététiques et sur l'hygiène<br>▪ expliquer les signes de danger
**n°2**	▪ garcon de 7 mois<br>▪ hémoglobine <9.3g/dl<br>▪ z-score entre >-3 et <-2 MAIS<br>▪ PB =114mm (<115mm)<br>▪ Presence d'œdèmes bilatéraux	**MAS non compliquée** avec anémie et retard de croissance	▪ antibiothérapie (amoxycilline) pour traitement systématique<br>▪ antipyrétique (paracétamol) si fièvre<br>▪ acide folique comme traitement systématique<br>▪ envoyer l'enfant au STA<br>▪ donner des conseils dietétiques et sur l'hygiène
**n°3**	▪ fille de 18 mois<br>▪ temperature: 37,8°c<br>▪ test rapide de paludisme (RDT): positif, sans signes de sévérité<br>▪ z-score entre <-2 et >-3<br>▪ pas d'œdèmes ou d'autres complications médicales<br>▪ même poids que 3 mois avant	**MAM** avec paludisme non compliqué et retard de croissance	▪ ASAQ en traitement oral; pas d'indication pour IV<br>▪ antipyrétique (paracétamol)<br>▪ envoyer l'enfant au SSN<br>▪ donner des conseils diététiques et sur l'hygiène

L'introduction à la vignette était très brève, par exemple « une maman vient vous consulter parce qu'elle trouve que Léonard, son petit enfant, ne va pas bien ». Ensuite, l'agent de santé était observé de la même manière qu'en consultation, avec une grille d'observation contenant des listes de questions d'anamnèse, d'examens physiques, de diagnostics et de traitements, basés sur les recommandations de la PCIME et le protocole national de prévention et de prise en charge de la malnutrition aigüe.

Pour chaque question ou examen réalisé, l'enquêteur donnait la réponse, par exemple, si l'agent demandait si l'enfant avait la toux, l'enquêteur donnait la réponse donnée dans la vignette. Si bien qu'à la fin, il était possible pour l'agent de santé de déterminer le diagnostic du patient ainsi que de proposer un traitement.

Mise en œuvre

Le questionnaire V était rempli pour la plupart sur papier. Il était administré en fin de journée aux agents observés en consultation auparavant (idéalement deux, parfois un seul lorsqu'il n'y en avait qu'un qui faisait ce travail ce jour-là). Les enquêteurs chargés de l'administration des vignettes sont les mêmes que ceux qui sont chargés des observations de consultations (questionnaire C). La formation,sur quatre jours, leur a permis de comprendre les objectifs des vignettes et de savoir comment le remplir correctement. La formation contenait des cours interactifs, des exercices de groupes, des tutoriels vidéo et des jeux de rôle. Les enquêteurs ont dû passer un examen écrit et un examen pratique durant le cinquième jour de la formation.

Avant d'administrer le questionnaire, les enquêteurs devaient, à l'aide d'un texte, expliquer les objectifs du questionnaire aux AS, clarifiant que les résultats ne seraient pas redirigés directement à leur employeur. Le consentement éclairé de chaque AS enquêté pour V devait aussi être obtenu.

Les enquêteurs ont reçu l'instruction d'administrer le questionnaire V après la fin des consultations, quand les AS avaient plus de temps et n'étaient plus dérangés par leurs responsabilités médicales quotidiennes. Les enquêteurs conseillaient aux AS de poser les questions d'anamnèse directement à l'enquêteur lui-même, comme s'il était l'accompagnant réel de l'enfant fictif. De même, les AS pouvaient proposer les examens physiques et tests de laboratoire qu'ils voudraient réaliser avant de donner le diagnostic et le plan de prise en charge. Les enquêteurs ne devaient souffler en aucun cas des idées aux AS, y compris ce que l'AS devrait expliquer (ou pas) à l'accompagnant fictif. Il était dit aux AS qu'ils pouvaient prescrire librement les tests de laboratoire, sans tenir compte de l'équipement disponible ou pas dans leur CDS.

2.4.2 Enquête au niveau de la communauté

L'organisation de l'enquête de référence au niveau de la communauté a été confiée à l'Institut de Statistiques et d'Etudes Economiques du Burundi (ISTEEBU). Au total, 72 enquêteurs, six superviseurs de terrain et trois gestionnaires ont été mobilisés pour la formation et l'enquête sur le terrain. L'enquête sur le terrain s'est déroulée du 8 décembre 2014 au 11 janvier 2015 (cf. rapport d'enquête, ISTEEBU, 2015).

Objectifs

Le principal objectif des questionnaires était d'évaluer le taux de malnutrition aigüe dans la communauté parmi les enfants de 6-23 mois. Les objectifs secondaires sont d'évaluer si ces taux ont une relation avec le statut socio-économique du ménage, le statut de santé de l'enfant, la sécurité alimentaire.

Contenu

SECTION 2 : Caractéristiques générales et socio-économiques

L'objectif de cette section est d'évaluer le statut socio-économique des ménages. Le questionnaire est basé sur les parties relatives des EDS et PMS (EDS 2010; MSPLS 2014) et est administré au chef de ménage ou à son conjoint.

SECTION 3 : Sécurité alimentaire

Les questions utilisées pour évaluer la sécurité alimentaire sont basées sur les questions génériques HFIAS d'échelle de l'accès déterminant l'insécurité alimentaire des ménages pour la mesure de l'accès alimentaire des ménages (cf. Coates, Swindale, & Bilinsky, 2007 ; projet FANTA). Cela comporte neuf questions de survenance qui présentent un niveau croissant de gravité de l'insécurité alimentaire (accès) et neuf questions de « fréquence-de-survenance » qui sont posées ensuite pour déterminer combien de fois est survenue cette situation.

Le Tableau 7 ci-dessous synthétise les principales catégories de sécurité alimentaire.

Tableau 7. Catégories de sécurité alimentaire, selon HFIAS

	Fréquence (durant les quatre dernières semaines)		
Questions<br><br>Durant les quatre dernières semaines,…	Une ou deux fois (rarement)	Trois à dix fois (de temps en temps)	Dix fois ou plus (souvent)
1. étiez-vous préoccupé que votre ménage n'avait pas assez de nourriture?			
2. est-ce que vous-même ou un membre de votre ménage n'a pas pu manger les types de nourriture que vous préférez à cause d'un manque de ressources?			
3. est-ce que vous-même ou un membre de votre ménage a mangé une variété limitée d'aliments parce que les ressources n'étaient pas suffisantes?			
4. est-ce que vous-même ou un membre de votre ménage a mangé une nourriture que vous ne souhaitiez pas manger à cause du manque de ressources pour obtenir d'autres types de nourriture?			
5. est-ce que vous-même ou un membre de votre ménage a mangé un repas plus petit que vous n'auriez souhaité parce qu'il n'y avait pas assez à manger?			
6. est-ce que vous-même ou un membre de votre ménage a mangé moins de repas par jour parce qu'il n'y avait pas assez de nourriture?			
7. est-il arrivé que le ménage soit sans nourriture du tout parce qu'il n'y avait pas de ressources pour en acheter?			
8. est-ce que vous-même ou un membre de votre ménage est allé au lit en ayant faim parce qu'il n'y avait pas assez de nourriture?			
9. est-ce que vous-même ou un membre de votre ménage a passé toute une journée sans manger parce qu'il n'y avait pas assez de nourriture?			

	Sécurité alimentaire		Insécurité alimentaire modérée
	Insécurité alimentaire légère		Insécurité alimentaire sévère

Source: Coates, Swindale, & Bilinsky, 2007 (FANTA)

SECTIONS 4, 5, 6 : Etat de santé, soins et alimentation apportés à l'enfant

Ces sections de questionnaire sont une combinaison de ceux du manuel de l'OMS pour évaluer les pratiques d'alimentation du nourrisson et du jeune enfant (WHO 2010) et de ceux conçus par l'unité de Santé et Nutrition de l'Enfant de l'IMT d'Anvers.

Les questions ont pour objectif d'évaluer, de façons subjective (opinion de la mère) puis objective (e.g. par le nombre de fois que l'enfant a visité le centre de santé durant une certaine période), le statut de santé et le statut nutritionnel de l'enfant. Les pratiques d'alimentation des mères sont évaluées, de même que leur opinion au regard des meilleures pratiques d'alimentation du nourrisson et du jeune enfant (WHO 2010).

SECTION 7 : Etat nutritionnel de l'enfant

Les mesures anthropométriques collectées à travers ce questionnaire permettent d'évaluer l'état nutritionnel de l'enfant. Cela permet d'évaluer les taux de prévalence de malnutrition aigüe et chronique parme les enfants de 6-23 mois dans la communauté. Couplées avec les données de l'enquête finale, ces données permettront de dire si le FBP Nutrition a un impact sur les taux de malnutrition dans la communauté.

Les indicateurs de résultat collectés sont les suivants :

- Prévalence de malnutrition aigüe (définie avec un z-score de poids pour taille inférieur à -2 ou un périmètre brachial inférieur à 125mm) ;
- Z-score du rapport poids pour taille, z-score du rapport taille pour âge et périmètre brachial
- Présence d'œdèmes
- Prévalence de malnutrition chronique (définie avec un z-score du rapport taille pour âge inférieur à -2)
- Périmètre brachial de la mère

Mise en œuvre

Les six superviseurs ont reçu une formation de six jours et les 72 enquêteurs de cinq jours. Cette formation couvrait les objectifs, le contenu et la mise en œuvre du questionnaire, et comprenait des cours ainsi que des sessions de groupe, des jeux de rôle et des sessions de démonstration pratique. Un examen écrit et pratique (y compris des mesures anthropométriques) a eu lieu le dernier jour de la formation. Un pré-test pilote avec tous les superviseurs et enquêteurs faisait également partie de leur évaluation.

Sur le terrain, les enquêteurs travaillaient en paires, avec un superviseur pour six paires d'enquêteurs. Les sections 1, 2, 3 étaient administrées au chef de ménage ; s'il n'était pas présent, à la personne qui avait le plus de responsabilité dans le ménage après le chef de ménage. Les sections 4, 5 et 6 ont été administrées à la personne qui s'occupait principalement de l'enfant identifié (habituellement, la mère. La section 7 implique généralement cette personne pour aider pour les mesures anthropométriques de l'enfant, et également la mesure sur elle-même du PB. Comme dit plus haut, les enquêteurs ont reçu une formation complète pour la prise de mesures anthropométriques (un certain nombre d'entre eux avaient déjà une expérience pour la prise de telles mesures, que ce soit à travers des enquêtes ou à travers des expériences cliniques). Aussi, un exercice de standardisation a été mis en œuvre durant la formation.

L'intégralité du questionnaire a été remplie sur smartphone à l'aide du software ODK. Le consentement de chacun des membres du ménage participant à l'enquête était obtenu au préalable.

2.5 Stockage, gestion et politique d'accès aux données

Le stockage et la gestion des données sont la responsabilité de l'ISTEEBU et l'INSP. L'accès est limité à l'ISTEEBU, l'INSP et l'IMT durant la période de l'analyse des données de l'enquête de référence. Les données pourront être accessibles par des parties tierces sur demande (et dossier d'application) ; les droits d'accès seront gérés par la Banque Mondiale et le gouvernement du Burundi.

3. Représentativité de l'échantillon et validité externe de l'étude

3.1 Représentativité géographique

Représentativité nationale

De par la sélection des 90 centres de santé de l'étude, l'échantillon couvre l'ensemble du territoire burundais : toutes les provinces, exceptée la province de Bujumbura mairie, disposent d'au minimum un centre de santé dans l'étude, comme le montre la carte ci-dessous. En conséquence, l'enquête auprès des ménages, dont les unités primaires d'échantillonnage sont les 90 centres de santé, est représentative du Burundi rural.

Figure 5. Carte du Burundi avec les 90 centres de santé de l'étude

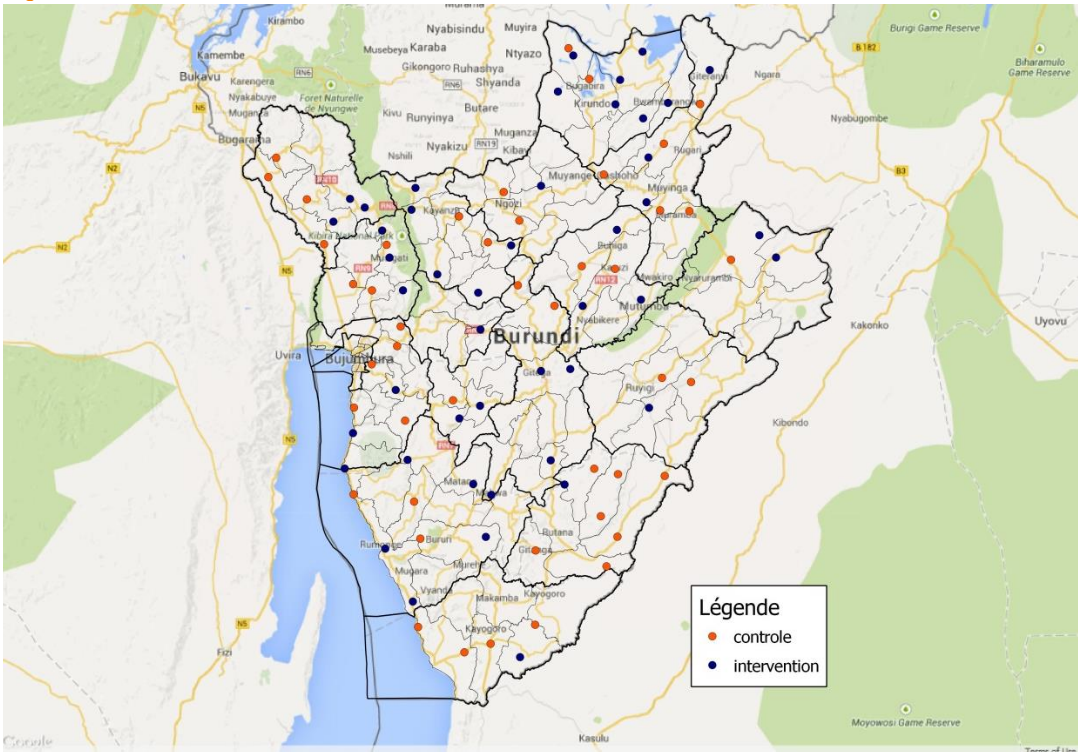

Source: Auteurs, avec Google map et Quantum GIS.

Représentativité du secteur santé

L'intégralité du secteur de la santé n'est pas représentée puisque les hôpitaux et les agents de santé communautaires ne font pas partie de l'étude. De plus, seuls les centres de santé avec FBP[14] sont impliqués dans cette étude, et seuls ceux qui disposent des services SSN et STA : en 2014, 193 centres de santé étaient éligibles, sur les 641 qui composaient le Burundi (UNICEF et PRONIANUT, communication personnelle 2013)

[14] En effet, certains CDS n'ont pas de contrat FBP direct avec le MSPLS: il s'agit de tous les CDS privés (non confessionnels), ainsi que de certains CDS publics et confessionnels ne suivant pas les normes en termes d'aire de responsabilité (ceux-ci signent en général un contrat secondaire avec des CDS sous contrat FBP direct).

3.2 Comparaison entre les enquêtes de référence et les statistiques nationales existantes

Enquête au niveau des centres de santé

Les données collectées ont pu être comparées à des données provenant d'autres enquêtes d'envergure nationales. Ainsi, les caractéristiques générales des centres de santé ici observées sont relativement similaires avec celles observées lors de l'enquête FOSA 2013 réalisée par l'INSP (réf. MSPLS et al. 2014). Les proportions de centres de santé publics par rapport à privés ainsi que l'aire de responsabilité moyenne sont à peu près les mêmes, selon ce que suggèrent les chiffres dans le tableau ci-dessous.

Tableau 8. Principales caractéristiques des centres de santé et équipement anthropométrique selon l'enquête FOSA 2013 et l'enquête FBP Nutrition de référence auprès des centres de santé (2014)

	FOSA (2013)	Enquête FBP Nutrition de Référence auprès des centres de santé (2014)
**Caractéristiques générales**		
**Statut du centre de santé**		
**Public: N (%)**	99 (80%)	75 (83%)
**Privé: N (%)**	25 (20%)	15 (17%)
**Population dans l'aire de responsabilité: moyenne (médiane)**	17,249 (14,938)	18,653 (med: 16,753)
**Equipement anthropométrique**		
**Présence d'une balance adulte et d'une balance pédiatrique Salter: % (n/N)**	57%	62% (46/74)
**Présence d'une toise (%)**	35%	68% (50/74)
**Présence de la grille de z-scores poids-pour-taille 2006**	40%	65% (48/74)
**No. observations**	124	90

Note: Les statistiques FOSA (2013) sont calculées pour les centre de santé sous FBP seulement (c'est-à-dire que (les centres n'ayant pas de contrat FBP et les hôpitaux ont été exclus afin d'avoir une population similaire). Source: Calculs des auteurs (à partir de l'enquête FOSA 2013 et de l'enquête FBP Nutrition de référence auprès des centres de santé).

Les données relatives aux indices de performance des services SSN et STA ont également été comparées avec les statistiques collectées par le PRONIANUT et le SNIS pour l'année 2012. Là aussi, ces chiffres sont relativement similaires à ceux obtenus durant l'enquête FBP Nutrition de référence au niveau des centres de santé fin 2014 (Tableau 9).

Tableau 9. Performance des services de nutrition selon le PRONIANUT et l'enquête FBP Nutrition de référence auprès des centres de santé (2014)

	PRONIANUT 2012	Enquête FBP Nutrition de référence auprès des centres de santé (2014)
**SSN**		
**Taux de guérison**	85.7%	79.9%
**Taux de référence**	3.0%	11.3%
**Taux de décès**	0.2%	0.8%
**Taux d'abandon**	4.7%	4.6%
**Taux de non répondant**	6.3%	3.2%
**N**	52,793	628
**STA**		
**Taux de guérison**	78.3%	85.4%
**Taux de référence**	4.3%	2.0%
**Taux de décès**	0.8%	1.4%
**Taux d'abandon**	11.1%	5.1%
**Taux de non répondant**	4.6%	5.1%
**N**	20,345	665

Source: PRONIANUT, et calculs des auteurs sur l'enquête de référence.

Enquête au niveau des ménages

Les mesures anthropométriques obtenues lors de l'enquête FBP Nutrition de référence au niveau des ménages ont pu être comparées à celles obtenues dans les EDS 2010, collectées d'août 2010 à janvier 2011. Selon les données présentées dans le Tableau 10, les chiffres de malnutrition chroniques sont similaires et suggèrent qu'ils n'ont pas beaucoup changé entre 2010 et 2014 : ils sont passés de 54% en 2010 à 53% en 2014 pour la tranche d'âge qui nous concerne, à savoir les 6-23 mois. Par contre, le taux de malnutrition aigüe a chuté entre l'enquête EDS de 2010 (10% pour cette tranche d'âge) et l'enquête ici présentée (6%). Cependant, il se peut qu'en quatre années la situation se soit améliorée, mais on doit également garder en tête que la malnutrition aigüe est un évènement de court-terme, et que des variations relativement importantes peuvent être observées selon la saison. Or, l'enquête EDS 2010 couvrait une des périodes de soudure (octobre-novembre), tandis que l'enquête FBP Nutrition de référence ici présentée était réalisée en décembre et début janvier, ce qui correspond à une période de récolte, où la nourriture est plus disponible. Cela peut expliquer ce taux de malnutrition aigüe réduit tel que vu avec l'enquête de fin 2014.

Une enquête SMART a été réalisée en février et mars 2014, fournissant également des chiffres sur la malnutrition pour les enfants de moins de cinq ans (WFP 2014). Cependant, le rapport ne fournit pas systématiquement les chiffres pour les groupes d'âge spécifiques considérés dans l'enquête FBP Nutrition, i.e. mes 6-23 mois. La malnutrition chronique est reportée et concerne 47.5% des enfants de cet âge, ce qui est 5.5 points de pourcentage en-dessous de nos estimations. Il est également reporté que 8.1% des enfants de 6-17 mois souffrent de malnutrition aigüe, ce qui est 1.6 points de

pourcentages en plus que dans notre enquête de référence. Cela étant, c'est considéré comme de faibles déviations.

La sécurité alimentaire a également pu faire l'objet de vérification. Les statistiques relatives à la sécurité alimentaire ont pu être comparées avec les données collectées dans les provinces de Cankuzo et Ruyigi en octobre 2012 dans le cadre d'une évaluation de programme de prévention de la malnutrition (réf. Projet FANTA, Parker et al. 2014). On observe dans le Tableau 11 ci-dessous que les chiffres sont relativement similaires. Par exemple, le score de sécurité alimentaire était de 11.7 sur 27 dans ces deux provinces en octobre 2012 (enquête FANTA), et est passé à 10.8 en moyenne en décembre 2014 et janvier 2015 selon les données collectées durant l'enquête FBP Nutrition de référence au niveau des ménages.

Tableau 10. Comparaisons de mesures anthropométriques entre les EDS 2010 et l'enquête FBP Nutrition de référence auprès des ménages

**EDS 2010**							
	Malnutrition chronique sévère (HAZ<-3)	Malnutrition chronique (HAZ<-2)	HAZ Score	Malnutrition aigüe sévère (WHZ<-3)	Malnutrition aigüe (WHZ<-2)	Surpoids (WHZ>+2)	WHZ score
Age 6-8m	12.9%	32.7%	-1.5	2.8%	10.2%	4.4%	-0.3
n	188	188	188	188	188	188	188
Age 9-11m	15.9%	45.4%	-1.6	3.8%	11.1%	3.0%	-0.6
n	185	185	185	185	185	185	185
Age 12-17m	25.7%	58.7%	-2.2	2.2%	10.7%	2.8%	-0.6
n	381	381	381	381	381	381	381
Age 18-23m	32.0%	63.4%	-2.4	2.2%	8.5%	2.0%	-0.4
n	380	380	380	380	380	380	380
**Enquête FBP Nutrition de Référence auprès des ménages(2014)**							
	Malnutrition chronique sévère (HAZ<-3)	Malnutrition chronique (HAZ<-2)	HAZ Score	Malnutrition aigüe sévère (WHZ<-3)	Malnutrition aigüe (WHZ<-2)	Surpoids (WHZ>+2)	WHZ score
Age 6-8m	9.7%	33.7%	-1.6	1.1%	5.7%	3.4%	-0.2
n	1166	1166	1166	1166	1166	1166	1166
Age 9-11m	15.2%	44.3%	-1.9	1.2%	6.2%	1.5%	-0.4
n	1015	1015	1015	1015	1015	1015	1015
Age 12-17m	22.5%	56.2%	-2.2	1.3%	6.9%	0.9%	-0.4
n	2076	2076	2076	2076	2076	2076	2076
Age 18-23m	29.2%	66.0%	-2.5	1.0%	5.1%	0.7%	-0.3
n	1942	1942	1942	1942	1942	1942	1941

Source: EDS 2010 et calculs des auteurs sur l'enquête de référence.

Tableau 11. Statistiques de sécurité alimentaire dans les provinces de Cankuzo et Ruyigi, de l'étude d'évaluation de Parket et al. (2012) à l'enquête FBP Nutrition de référence au niveau des ménages (2014-15)

	**Parker et al** Oct. 2012	**PBF Nutrition référence** *Dec 2014-Jan 2015*
**Score (entre 0 et 27)**	11.7	10.8
**Sécurité alimentaire**	10.2 %	14.7%
**Insécurité alimentaire légère**	6.9%	7.8%
**Insécurité alimentaire modérée**	33.4%	18.9%
**Insécurité alimentaire sévère**	49.5%	58.6%
**N**	2,614	408

Source: Parker, Leroy, Olney, Harris, & Ruel, 2014(FANTA) et calculs des auteurs sur l'enquête de référence.

4. Résultats de l'enquête de référence

4.1 Enquête au niveau des centres de santé

L'enquête s'est déroulée du 21 septembre au 7 octobre 2014, et a permis la collecte de données auprès des 90 centres de santé de l'étude, comme prévu. Le tableau suivant donne, pour chaque type de questionnaire, le nombre de questionnaires collectés au total, le taux de couverture de l'échantillon ainsi que l'explication de l'écart.

Tableau 12. Taux de couverture pour chacun des outils de collecte de l'enquête CDS

Questionnaire	Nombre attendu	Nombre collecté	Taux de couverture	Explication
Questionnaire G	90	90	100%	
Questionnaire N	90	90	100%	
Questionnaire SSN	90 x 12 = 1080	971	90%	Pas suffisamment de dossiers individuels dans 14 CDS
Questionnaire STA	90 x 12 = 1080	963	89%	Pas suffisamment de dossiers individuels dans 17 CDS
Questionnaire C	90 x 6 = 540	515	95%	Pas suffisamment de consultations dans 10 CDS
Questionnaire S	90 x 6 = 540	512	95%	Pas suffisamment de consultations dans 10 CDS + 3 accompagnants perdus
Questionnaire V	90 x 2 = 180	145	81%	Un seul AS dans 35 CDS

Source: Rapport d'enquête, INSP 2014.

Aspects organisationnels des centres de santé

Questionnaire G– Aspects organisationnels du centre de santé

Comme indiqué dans la section 2, ce questionnaire est prévu pour être administré au titulaire de chacun des 90 centres de santé de l'étude. En cas d'empêchement de ce dernier il est administré au titulaire adjoint. Il a été effectivement administré au titulaire dans 57 centres de santé (63%), au titulaire adjoint dans 31 cas (34%) et à un autre personnel du centre dans 2 cas (2%).

Là où c'était possible, les questions devaient être recoupées par les enquêteurs (vérification de la documentation fournie, demande d'autres sources de données, etc.). Malgré la formation et les efforts de supervision, les rapports hebdomadaires des superviseurs étaient révélatrices d'un manque de vérification systématique par les enquêteurs. Nous réservons la prudence à certaines des réponses et statistiques en raison de cet état de fait.

En moyenne, selon les répondants, la population de l'aire de responsabilité d'un centre de santé est de 18,653 (médiane: 16,754). Au sein de cette population, le nombre d'enfants de moins de cinq ans est en moyenne de 3,840 (médiane: 3,255). Un centre de santé couvre en moyenne 22 sous-collines (médiane: 20).

Organisation et infrastructure

Globalement, 83% des centres de santé sont du secteur public, tandis que 17% relèvent du secteur privé confessionnel.

En termes de gestion générale et d'outils de communication, 86% des centres de santé ont déclaré avoir un téléphone, un accès à une imprimante pour 73%, et un ordinateur fonctionnel pour 50%. Parmi ces derniers, 76% ont déclaré avoir une imprimante fonctionnelle et un accès à internet pour 11%.

Gestion et ressources humaines

Quatre-vingt-huit centres de santé (98%) ont déclaré qu'ils ont un comité de santé (appelé « COSA») et 85 (94%) ont déclaré avoir un comité de gestion (également appelé « COGE »). D'après les normes de santé 2011-2015 du Burundi, le COGE est élu au sein du COSA en vue d'une cogestion du CDS. Ainsi, si on ne considère que ces résultats, il ressort qu'il y a certains CDS où la cogestion n'est pas garantie. Par ailleurs, du moment que la question était posée au responsable de la structure sans vérification par l'enquêteur, il est difficile de conclure que ces organes de bonne gouvernance sont fonctionnels dans ces structures. Bien plus, il y a lieu de se demander s'il n'y a pas eu confusion sur la définition même de COGE, entre le COGE pour la cogestion et le COGE habituellement en place dans les FOSA dans le cadre du FBP. Ce dernier est constitué uniquement par le personnel du CDS et s'intéresse chaque mois à la planification des recettes et dépenses et au partage d'une éventuelle enveloppe dégagée pour les primes de performance. Cette question devra être clarifiée pour éviter la confusion et inciter à la vérification par les enquêteurs durant l'enquête de suivi.

En moyenne, un centre de santé emploie 15,5 personnes (médiane: 15) et s'appuie également sur 23,7 agents de santé communautaire (médiane: 18,5). Parmi les employés, 60% sont des hommes; 57% sont dits être employés par l'Etat, 41% par le centre de santé, et 4% par une ONG. En moyenne, 58% du personnel a un contrat avec l'état (44% sous-statut, 14% sous-contrat), et 39% ont un contrat à durée déterminée avec le secteur privé.

Parmi tous les employés des 90 centres de santé recensés, 28% ont un diplôme de niveau A3 (deux années de formation paramédicale), 21% le niveau A2 (quatre années de formation paramédicale), et seulement 1% le niveau d'un diplôme A1 (diplôme universitaire d'infirmière) ou plus. Au total, 78 (87%) des centres de santé ne disposent pas de prestataires avec le niveau A1 et cinq (6%) ne disposent pas d'un personnel avec le niveau A1 ou A2. Parmi ces cinq centres de santé, l'aire de responsabilité couvre une population allant de 8 à 28 milliers de personnes.

Selon les normes sanitaires du Burundi, un centre de santé devrait avoir au moins trois infirmiers avec un diplôme A2 (ou plus), trois infirmiers avec un diplôme A3, un technicien de laboratoire, un technicien de promotion de la santé ainsi qu'un gestionnaire financier. Seulement 20 (22%) centres de santé respectent ces normes. Plus précisément:

-58 (64%) centres de santé ont trois ou plus d'infirmières avec un diplôme A2 (ou plus) ;
-69 (77%) centres de santé ont trois ou plus d'infirmières avec un diplôme A3 ;
-50 (56%) ont un technicien de laboratoire ;

-46 (51%) ont un technicien de promotion de la santé ;
-72 (80%) ont un gestionnaire financier.

L'organigramme du centre de santé a été signalé affiché et mis à jour dans 39 centres de santé (43%), les registres de présence ont été signalés disponibles et mis à jour dans 77 centres de santé (86%) et les fiches descriptives des postes dans 52 centres de santé (58 %). Au total, seulement 27 centres de santé ont déclaré avoir tous les trois documents disponibles et mis à jour. Les registres de formations entreprises étaient présents dans huit (9%) centres de santé. Les documents de statuts du personnel étaient disponibles et mis à jour dans 52 (58%) centres de santé.

Soixante-dix-neuf centres de santé (88%) ont déclaré qu'ils évaluent régulièrement la performance de leur personnel. En outre, la moitié des répondants ont déclaré que leur personnel suit régulièrement des bilans de santé. Un seul centre de santé sur 90 a déclaré avoir une procédure pour le suivi des accidents de travail.

Tous les centres de santé ont déclaré qu'ils procèdent régulièrement à un inventaire des stocks de leurs médicaments.

Questionnaire N - Aspects organisationnels des services liés à la malnutrition

Comme pour le questionnaire G, quand c'est possible, l'information fournie par le répondant (membre du personnel en charge des services de nutrition et qui y travaille régulièrement) était censée être recoupée par les enquêteurs. Mais tout comme avec le questionnaire G, il semble que cela n'a pas été fait systématiquement. Cela devrait nous rendre prudents sur la validité des réponses et l'interprétation de ces statistiques. Les questions sur les traitements et sur les équipements spécifiques sont cependant susceptibles d'être plus fiables d'autant plus que les enquêteurs devaient vérifier leur disponibilité eux-mêmes et rapporter les données figurant dans les registres spécifiques.

Description des services

Programme de suivi et promotion de la croissance

Parmi les 90 centres de santé, 74 (82%) ont déclaré qu'ils offraient des services de suivi de la croissance. Ils ont dit qu'ils réalisaient cette activité en mesurant le poids (100%), la hauteur (85%) et le PB (69%), et en prodiguant des conseils, soit individuellement (66%) ou en groupe (88%).

La moitié des répondants (51%) ont déclaré qu'ils n'avaient jamais tracé de courbe de croissance. En effet, bien que 49% d'entre eux ont indiqué qu'ils tracent systématiquement les courbes de croissance, cela n'a pas été confirmé par les informations recueillies dans les interviews à la sortie (S) et les observations des consultations (C) : il y avait seulement une courbe de croissance observée tracée dans un carnet de santé sur 514 enfants enquêtés lors des entretiens à la sortie (questionnaire S) ; de même, le traçage de la courbe ou la référence à la courbe de croissance de l'enfant ont été observés dans seulement 37 consultations (sur un total de 513 ; questionnaire C).

Enfin, plus de la moitié des répondants (54%) ont déclaré qu'ils appliquaient le protocole de suivi de la croissance. Etant donné qu'actuellement il n'y a pas de lignes directrices nationales de promotion et

suivi de la croissance pour le Burundi, nous supposons que les répondants font référence aux directives de la PCIME (OMS et UNICEF, 2001, page 49). L'efficacité de l'application de ce protocole peut être à nouveau mise en doute à la lumière des données des questionnaires S et C, comme mentionné ci-dessus.

Détection de la malnutrition

Au total, 79% des répondants au questionnaire N ont rapporté qu'ils détectent la malnutrition lors des consultations curatives (comme nous le verrons plus loin avec les questionnaires S et C, la détection de la malnutrition dans les consultations curatives est en fait constamment négligée). Ils ont également signalé que la détection de la malnutrition se fait lors des consultations préventives (72%), des consultations post-natales (23%), et des consultations nourrissons sains (40%). Dans trois centres de santé, il a été rapporté qu'il n'y avait pas de détection du tout. Cent pour cent des répondants ont déclaré qu'ils prodiguent des conseils sur la nutrition (76% sur une base individuelle, 80% dans un groupe), mais nous verrons à partir des questionnaires S et C que seulement un quart des patients ont reçu des conseils de nutrition.

Parmi les 74 centres de santé qui ont répondu à ces questions, dix d'entre eux (14%) ont tout l'équipement et les outils nécessaires pour le dépistage des enfants (de la malnutrition) non seulement disponible, mais fonctionnel. Seuls deux centres de santé ont déclaré avoir du matériel d'éducation pour la santé en matière de nutrition et du materiel de démonstration culinaire.

Prise en charge de la MAM – service de supplémentation nutritionnelle (SSN)

Parmi les 90 centres de santé enquêtés, 54 (60%) ont déclaré qu'ils avaient un service SSN, ce qui signifie que 36 ont indiqué le contraire. Pourtant, ces centres de santé ont été sélectionnés pour faire partie de l'étude parce qu'ils avaient ce service (ensemble avec le service STA). Cette information avait été fournie par l'UNICEF en 2013, et plus tard vérifiée par l'un des auteurs au début de 2014[15]. Ces réponses négatives sont probablement dues au fait que les aliments de supplémentation ne sont plus fournis par le PAM depuis 2014, ce qui fait que les centres considèrent qu'ils ne peuvent plus réaliser ces services.

Les documents nécessaires pour ce service SSN sont les registres, cartes individuelles des patients, les fiches de statistiques hebdomadaires/mensuels sur le rendement, fiches de bilan de stocks et des bordereaux de livraison. Ils étaient tous disponibles et mis à jour dans seulement quatre centres de santé (7%). Les aliments de supplémentation (traitement diététique) étaient disponibles dans 10 (19%) des centres de santé, mais les consommations moyennes mensuelles (CMM) n'ont été trouvées calculées dans aucun des centres. Au cours des trois derniers mois, quarante-huit (89%) des centres de santé ont connu une rupture de stock en aliments de supplémentation.

Les indicateurs de performance du service pour le PRONIANUT (cf. protocole, MSPLS 2010) sont calculés systématiquement dans 22 (41%) des centres de santé; ils ne sont jamais calculés également dans 22 (41%) des centres de santé.

---

[15] Le PAM a cessé de fournir les intrants thérapeutiques alimentaires dans les centres de santé en 2014 (sauf dans les provinces de Rutana et Ruyigi).

Onze (20%) des centres de santé ont indiqué qu'ils font des visites de sensibilisation au niveau communautaire pour la récupération des abandons du service SSN.

Prise en charge de la MAS – Service de thérapeutique ambulatoire (STA)

Parmi les 90 centres de santé, 89 (99%) ont déclaré qu'ils ont un service de réhabilitation (STA). La plupart d'entre eux (90%) ouvrent le service une fois par semaine.

Les documents nécessaires pour ce service sont les registres, cartes individuelles des patients, des fiches de statistiques hebdomadaires/mensuelles sur le rendement, les fiches de bilan de stocks et les bordereaux de livraison. Ils étaient tous disponibles et mis à jour dans huit centres de santé (9%). Les intrants diététiques étaient disponibles dans 67 (75%) des centres de santé; les consommations moyennes mensuelles étaient calculées dans 48% de ces cas. Au total, 47 (55%) des centres de santé ont connu une rupture de stock en intrants diététiques, au cours des trois derniers mois.

Les indicateurs de performance du service pour le PRONIANUT (cf. protocole, MSPLS 2010) étaient calculés systématiquement dans 42 (47%) des centres de santé; ils n'étaient jamais calculés dans 24 (27%) des centres de santé.

Quatorze (16%) des centres de santé ont indiqué qu'ils font des visites de sensibilisation au niveau communautaire pour la recuperation des abandons du service de STA.

Infrastructure de stockage

Les aliments de supplémentation pour le service SSN, à savoir le CSB, l'huile et le sucre, sont stockés pour 88% des centres de santé dans un endroit qui est verrouillé et pour 6% dans la pharmacie.

Les aliments thérapeutiques pour le service STA, soit le Plumpy Nut®, sont stockés pour 74% des centres de santé dans un endroit qui est verrouillé et pour 19% dans la pharmacie.

Références

Références vers l'hôpital

Quatre-vingt-neuf (99%) des centres de santé ont indiqué qu'ils réfèrent les cas de malnutrition aiguë avec des complications vers un service spécialisé (SST) dans un hôpital. Parmi ces 89 centres, 75% ont déclaré qu'ils reçoivent une rétroinformation sur les enfants référés. Ces services SST sont en moyenne à 21 kilomètres (médiane: 20 km) du centre de santé.

Références venant d'autres centres de santé

Au total, 56 (62%) des centres de santé ont signalé qu'ils reçoivent des enfants détectés et dépistés comme MAM d'autres centres de santé qui ne disposent pas de services de malnutrition et 53 (59%) des centres de santé reçoivent des cas de MAS d'autres centres. Cependant, à partir des fiches individuelles individuelles, il est évident que, pour 89% de l'échantillon pour le SSN et 79% pour le STA, il n'a pas été clairement indiqué si le patient avait été dépisté dans le centre de santé lui-même ou référé d'un autre centre de santé.

Supervision des services de nutrition

Seulement 40 (44%) des centres de santé ont indiqué que leurs services de nutrition ont été visités par l'équipe de supervision du district les six derniers mois. Parmi eux, 22 (55%) avaient été supervisés par le point focal de la nutrition du district et 23 (58%) par le superviseur polyvalent. En moyenne, ils avaient 1,9 visites de supervision par centre.

Partenaires techniques et financiers

Globalement, 33% des centres de santé ont signalé qu'ils n'avaient pas de partenaires pour les activités de dépistage de la malnutrition au niveau des centres de santé, tandis que 26% ont déclaré une ou plusieurs ONG qui les appuie(nt). En ce qui concerne le traitement de la malnutrition aiguë, 18 (20%) des centres de santé ont signalé qu'ils n'avaient pas de partenaires pour appuyer ces activités, tandis que 53 (59%) des centres de santé ont déclaré qu'ils ont été appuyés par le PAM et/ou l'UNICEF, et 14 (16%) par une ou plusieurs ONG.

Interruption des services de nutrition

Les données de la section du questionnaire sur les interruptions de service ne sont pas disponibles en raison d'un filtre mal placé dans la conception du questionnaire sur le smartphone. On a demandé aux répondants si leurs services de nutrition avaient été interrompus au cours des six derniers mois, ce qui était la raison de l'interruption, et quelles ont été les stratégies mises en place pour y faire face. Malheureusement, en raison de l'erreur de filtre, seuls 36 ont répondu à ces questions.

Traitement systématique

Le traitement systématique réfère à la médication fournie aux enfants souffrant de malnutrition aiguë. Selon les lignes directrices nationales de 2010 pour le traitement de la malnutrition (en usage pendant la période d'enquête), pour les cas de MAS dans le STA, il consiste à l'amoxicilline, l'artésunate-amiodiaquine (ASAQ ; si un test pour le paludisme était positif), l'al/mébendazole, l'acide folique (s'il y a des signes d'anémie à l'admission – il est le plus souvent trouvé dans les centres de santé en combinaison avec le fer) et la vaccination antirougeoleuse. Le traitement des cas de MAM dans les services SSN implique la vitamine A, l'al/mébendazole, l'acide folique/fer et la vaccination contre la rougeole (MSPLS, 2010). Les enquêteurs, en collaboration avec l'enquêté, procédaient à la vérification de la disponibilité et de la quantité dans la pharmacie et dans les registres et fiches spécifiques.

Parmi les 90 centres de santé, 75 (83%) avaient l'amoxicilline, 77 (86%) l'ASAQ, 86 (96%) l'al/mébendazole, 43 (48%) l'acide folique, 64 (71%) le fer/acide folique, et 71 (79%) le vaccin antirougeoleux. Dans l'ensemble, 22 (24%) avaient tout le traitement systématique requis pour les services de la malnutrition le jour de l'enquête.

Dans tous les 90 centres de santé il a été signalé qu'ils étaient en rupture de stock ou en avaient connu pour un ou plusieurs médicaments systématiques au cours des trois derniers mois. Afin d'éviter ces ruptures de stock, un outil de gestion, le calcul de la consommation moyenne mensuelle (CMM), est censé être utilisé dans tous les centres de santé. Il semble qu'elle a été calculée dans 81% des centres de santé pour l'amoxicilline, 91% pour l'ASAQ, 77% et 91% pour l'al/mébendazole, 77% pour l'acide folique, de 81% pour le fer/acide folique et 46% pour les vaccins contre la rougeole.

Evaluation des dossiers médicaux individuels issus des services liés à la malnutrition

Fiches individuelles de suivi des services de supplémentation nutritionnelle (SSN)

Dans l'ensemble, les enquêteurs ont transcrit les informations relatives à 971 cas cliniques de MAM. Toutefois, comme indiqué dans la section 2, dans de nombreux cas, il n'y avait pas de dossiers individuels disponibles dans le centre de santé, et les enquêteurs ont ensuite été amenés à transcrire les informations des registres spécifiques. Les registres ne fournissaient pas d'information sur le traitement reçu ou le nombre de visites, mais le plus souvent ont fourni des informations sur les mesures anthropométriques à l'admission et à la sortie ainsi que la situation du patient à la sortie (guéri, référé, etc.). Malheureusement, dans de nombreux cas, les informations relatives à la taille et au poids ainsi que le résultat du programme n'ont pas été trouvées (Tableau 13).

D'après les rapports hebdomadaires des superviseurs de l'enquête, nous pouvons voir que toutes les fiches individuelles SSN manquaient dans 16 centres de santé (sur 85) et parmi ces 16, cinq centres de santé utilisaient des carnets non standards (souvent des petits cahiers achetés sur le marché) plutôt que les fiches individuelles standards du SSN. Dans huit centres de santé (sur les 85), il manquait également les registres SSN.

Mesures anthropométriques à l'admission

En moyenne, le z-score du rapport P/T à l'entrée dans le programme SSN était -2,06, soit en dessous de -2 et au dessus de -3, ce qui signifie que, en moyenne, les cas admis dans le service de supplémentation nutritionnelle souffraient en effet de MAM (Tableau 14). Cependant, dans 153 cas (17%), le z-score du rapport P/T était inférieur à -3, ce qui signifie que l'enfant était en fait un cas de MAS et ne devait pas être dans le service SSN. Cent vingt-sept cas (14%) étaient au-dessus de -2 et avec un PB normal et sans œdème (mais il n'y avait aucune information sur la présence d'oedème dans 84 cas) ce qui signifie que l'enfant était nourri normalement. En outre, certains de ces cas ont été signalés comme ayant un œdème à l'admission (3,8% ou 15 cas au total, parmi les 394 fiches pour lesquelles les informations sur la présence d'oedème a été rapportée), ce qui devait conduire à les référer directement au service STA.

Mesures anthropométriques à la sortie et raisons de sortie

Parmi les 400 fiches individuelles de suivi pour lesquelles des informations sur l'anthropométrie à la sortie pouvait être retrouvée, le z-score P/T à la sortie était en moyenne de -1,33, soit supérieur à -2, ce qui suggère que, en moyenne, les enfants ne souffraient plus de MAM à la sortie du programme. Selon les 628 fiches individuelles pour lesquelles nous disposons d'informations sur la raison de sortie, 79,9% des enfants étaient guéris; 11,3% ont été transférés à un niveau supérieur (10,5% au service STA, 0,8% au service SST), 4,6% étaient des rechutes et 0,8% sont décédés. Ces chiffres suggèrent une performance relativement bonne du service par rapport aux lignes directrices nationales (Tableau 15).

Toutefois, il convient encore de noter que 49 cas qui ont été rapportés comme guéris avaient un z-score P/T inférieur à -2 (huit d'entre eux ayant un PB normal). Aucun des 502 cas guéris n'avait un oedème à la sortie du programme.

En outre, le gain pondéral journalier en grammes par poids initial était en moyenne de 1,74 g / kg / jour (Tableau 15), ce qui est en dessous des directives de protocole national. En outre, la durée du

traitement était en moyenne de 75 jours (médiane: 70), qui est au-dessus des recommandations des directives nationales (devrait être inférieure à 60 jours).

Traitement systématique et supplémentation nutritionnelle

Le Tableau 16 donne les statistiques concernant le traitement systématique donné aux patients (tel que rapporté dans les fiches). Selon les données, pour la plupart des cas (64,7%), il n'a pas été rapporté de traitement systématique reçu; seulement 19,3% ont reçu l'al/mébendazole, 9,8% la vitamine A, et 8,4% le fer/acide folique. Seulement 6, 1% ont reçu les trois médicaments.

En ce qui concerne la supplémentation nutritionnelle, nous avons observé que, en moyenne, pour un tiers des visites, les enfants n'ont reçu aucun supplément alimentaire. En raison de la mauvaise qualité de la documentation, il était souvent difficile pour les enquêteurs d'enregistrer avec précision le nombre de visites; donc l'analyse a été effectuée sur tous les cas qui ont eu plus de deux visites afin d'éviter d'utiliser des cas qui avaient seulement une visite d'entrée et de sortie notifiées.

Le manque apparent de supplémentation nutritionnelle par visite pourrait être un problème de documentation des patients plutôt que des enfants ne recevant pas réellement le traitement systématique et/ou suppléments alimentaires. Même pour les cas où, par exemple, il y avait une croix dans la case correspondant à un supplément, les quantités n'ont pas été enregistrées (même si cela est demandé sur la fiche même); ainsi, nous étions incapables de calculer non seulement les quantités reçues par l'enfant, mais aussi la valeur nutritionnelle de la supplémentation pour chaque enfant.

Tableau 13. Informations manquantes sur les fiches individuelles de suivi ou registres SSN

Information manquante sur:	Nombre de fiches	Moyenne	Déviation standard
Poids à l'admission	971	1.1%	0.00
Taille à l'admission	971	3.6%	0.01
PB à l'admission	971	17.6%	0.03
oedèmes à l'admission	971	59.4%	0.05
raison de sortie du programme	971	35.3%	0.04
Poids à la sortie	971	9.8%	0.02
Taille à la sortie	971	57.8%	0.04
PB à la sortie	971	36.8%	0.04
Oedèmes à la sortie	971	67.5%	0.05

Source: calculs des auteurs à partir de l'enquête de base FBP Nutrition

Tableau 14. Mesures anthropométriques à l'entrée et à la sortie et les raisons de sortie du service SSN (là où l'information est disponible)

	Nombre de fiches	Moyenne	Déviation standard
**Age (mois)**	951	24.76	0.69
**Sexe (1=masculin, 2=féminin)**	942	1.52	0.01
**z-score P/T à l'admission**	903	-2.06	0.07
**z-score P/A à l'admission**	894	-3.39	0.10
**PB (mm) à l'admission**	800	119.33	0.61
**oedèmes à l'admission**			
Absents	394	96.2%	0.01
+	394	1.8%	0.01
++	394	1.0%	0.01
+++	394	1.0%	0.01
**z-score P/T à la sortie**	400	-1.33	0.10
**z-score P/A à la sortie**	391	-3.07	0.20
**Raison de sortie**			
Transféré au SST(hôpital)	628	0.8%	0.00
transféré au STA	628	10.5%	0.03
guéri	628	79.9%	0.03
Abandon	628	4.6%	0.01
Décédé	628	0.8%	0.00
Non répondant	628	3.2%	0.01
**Gain pondéral en grammes par poids initial en kg et par jour de traitement, depuis l'entrée jusqu'à la sortie (uniquement pour ceux rapports comme guéris)**	437	1.74	0.22
**La durée du traitement en jours, depuis l'entrée jusqu'à la sortie (uniquement pour ceux rapportés comme guéris)**	477	74.64	5.75

Source: calculs des auteurs à partir de l'enquête de base FBP Nutrition

Tableau 15. Performance du service SSN par rapport aux directives nationales

	Enquête	Directives nationales	Atteint?
**Taux de guérison**	79.9% (N=628)	>75%	✓
**Taux de tranfert**	11.3% (N=628)	<10%	✓
**Taux de décès**	0.8% (N=628)	<3%	✓
**Taux d'abandon**	4.6% (N=628)	<15%	✓
**Gain pondéral en grammes par poids initial en kg et par jour de traitement, depuis l'entrée jusqu'à la sortie**	1.74 (N=437)	>2-3	✗
**La durée du traitement en jours, depuis l'entrée jusqu'à la sortie (uniquement pour ceux rapportés comme guéris)**	74.64 (N=477) (med: 70)	<60	✗

Source: calculs des auteurs à partir de l'enquête de base FBP Nutrition et MSPLS (2010)

Tableau 16. Traitement systématique et diétetique au service SSN

	Nombre de fiches	Moyenne	Déviation standard
**Traitement systématique:**			
Aucun	971	64.7%	0.04
al/mebendazole	971	19.3%	0.03
vitamine A	971	9.8%	0.02
Fer/Acide folique	971	8.4%	0.02
**Traitement diétetique:**			
Nombre de visites sans aliments de supplementation sur le nombre total de visites (si nombre de visites >2)	677	29.0%	0.04

Source: calculs des auteurs à partir de l'enquête de base FBP Nutrition

Fiches individuelles de suivi des services de thérapeutique ambulatoire (STA)

Dans l'ensemble, les enquêteurs ont retranscrit les informations relatives à 963 cas cliniques de MAS. Cependant, comme pour le SSN, dans de nombreux cas, il n'y avait pas de fiches individuelles de suivi au centre de santé, et les enquêteurs ont été amenés à retranscrire les informations contenues dans des registres spécifiques. Malheureusement, dans de nombreux cas, il y avait beaucoup d'informations qui ne pouvaient pas être trouvées: tel est le cas par exemple du test d'appétit à l'entrée dans 41% des cas, de la présence d'oedèmes à l'entrée et à la sortie dans respectivement 34,4% et 47,7% des cas, du traitement systématique dans 29,5% des cas, du résultat du patient dans 30,9% des cas et de la taille à la sortie dans 55,2% des cas (Tableau 17).

Des rapports de rendement hebdomadaires faits par les superviseurs de l'enquête, nous pouvons voir que toutes les fiches individuelles STA manquaient dans neuf centres de santé (sur 85); parmi ces neuf, quatre centres de santé utilisaient des carnets non standards plutôt que les fiches individuelles standards STA. Dans six centres de santé (sur les 85), il manquait également les registres de STA.

Mesures anthropométriques à l'admission

En moyenne, le z-score P/T à l'entrée est -2.45, soit au-dessus de -3, (Tableau 18), ce qui signifie qu'en moyenne, les cas admis dans le service de STA ne disposaient pas d'un z-score P/T correspondant à la MAS, mais cela ne donne pas l'image suffisante car dans les cas où l'enfant a aussi des oedèmes, alors le diagnostic de MAS est correct et il est approprié d'être dans le service STA. Aussi, d'après les données disponibles, dans 111 cas (12%), le z-score P/T à l'entrée était au-dessus de -3, il n'y avait pas d'œdèmes signalés et le PB était au-dessus de 115, ce qui signifie que l'enfant n'était pas sévèrement malnutri. Le z-score T/A moyen est inférieur à -3, ce qui suggère que, en moyenne, les enfants souffraient de retard de croissance sévère. Parmi les 632 fiches pour lesquelles nous disposons d'informations sur les oedèmes, 47,3% ont été trouvés avec oedèmes, de légère (15,7%) à sévère (10,8%). Parmi les 568 fiches pour lesquelles nous disposons d'informations sur le test d'appétit, le résultat était bon dans 83,1% des cas et moyen dans 16,5%.

Mesures anthropométriques à la sortie et raisons de sortie

Parmi les 408 fiches pour lesquelles des informations sur l'anthropométrie à la sortie pouvaient être récupérées, le z-score P/A était en moyenne de -1,26, soit supérieur à -2, ce qui suggère que, en moyenne, les enfants étaient guéris à la sortie du programme. Selon les 665 fiches individuelles pour lesquelles nous avons l'information, 85,4% des enfants étaient rapportés comme guéris, 2,0% ont été transférés au service SST (hôpital), 5,1% étaient des abandons et 1,4% décédés. Ces chiffres suggèrent une performance relativement bonne du service par rapport aux directives nationales (Tableau 19).

Il convient toutefois de noter que, parmi les 568 cas signalés comme guéris, le z-score P/T était inférieur à -3 dans 21 cas et les oedèmes ont été signalés dans 8 cas: au total, 29 (5%) des cas rapportés comme guéris ne sont pas effectivement guéris.

En outre, le gain pondéral journalier en grammes par poids initial est en moyenne de 3,92 g/kg/jour (Tableau 19). Ce chiffre est inférieur à celui des directives du protocole national. Enfin, la durée du traitement est de 59 jours en moyenne (médiane: 50), qui est au-dessus de celui recommandé par les directrices nationales (devrait être inférieure à 30 jours).

Traitement systématique et supplémentation nutritionnelle

Le Tableau 20 donne les statistiques sur le traitement systématique rapporté. Selon les 679 fiches pour lesquelles nous avions des informations sur le traitement systématique, 47,6% ont reçu de l'amoxicilline et 64,7% de l'al / mébendazole. Dans l'ensemble, 44,3% ont reçu les deux médicaments et 16,9% ont reçu également de l'acide folique et de la vitamine A.

En ce qui concerne les aliments thérapeutiques de supplémentation, nous avons observé que, en moyenne, pour un cinquième des visites, les enfants ne recevaient aucune nourriture thérapeutique. En raison de la mauvaise qualité de la documentation, il est souvent difficile pour les enquêteurs d'enregistrer avec précision le nombre de visites, donc l'analyse a été effectuée uniquement sur les cas qui ont eu plus de deux visites afin d'éviter d'utiliser des cas qui avaient seulement comme visites documentées, l' entrée et la sortie.

Comme pour le SSN, le manque apparent d'aliments thérapeutiques de supplémentation par visite pourrait être un problème dû à la documentation des patients plutôt que des enfants ne recevant pas réellement le traitement systématique et/ou aliments thérapeutiques. Même dans les cas où, par exemple, il y avait une croix dans la case correspondant à un aliment thérapeutique, les quantités n'ont pas été enregistrées (même si cela est demandé sur la fiche même); ainsi, nous étions incapables de calculer non seulement les quantités reçues par l'enfant, mais aussi la valeur nutritionnelle de la supplémentation recue par chaque enfant.

Tableau 17. Informations manquantes sur les fiches individuelles de suivi ou les registres STA

information manquante sur:	Nombre de fiches	Moyenne	Déviation standard
si le cas était référé	963	79.2%	0.04
si le cas avait été déjà traité de MAS	963	60.5%	0.05
Poids à l'entrée	963	0.7%	0.00
Taille à l'entrée	963	3.9%	0.02
PB à l'entrée	963	13.1%	0.03
Oedèmes	963	34.4%	0.04
Test d'appétit	963	41.0%	0.05
Traitement systématique	963	29.5%	0.04
vaccin anti-rougeoleux	963	72.7%	0.04
traitement anti-paludique	963	75.1%	0.04
raison de sortie du programme	963	30.9%	0.04
poids à la sortie	963	6.1%	0.02
taille à la sortie	963	55.2%	0.04
PB à la sortie	963	31.5%	0.04
oedèmes à la sortie	963	47.7%	0.04

Source: calculs des auteurs à partir de l'enquête de base FBP Nutritiobn

Tableau 18. Mesures anthropométriques à l'entrée et à la sortie et les raisons de la sortie du service STA, lorsque l'information est disponible

	Nombre de fiches	Moyenne	Déviation standard
**Age (mois)**	949	27.05	0.77
**Sexe (1=masculin, 2=féminin)**	918	1.45	0.02
**z-score P/T à l'admission**	869	-2.45	0.08
**z-score P/A à l'admission**	875	-3.65	0.10
**PB (mm) à l'admission**	837	115.40	0.75
**Oedèmes à l'admission**			
Absents	632	52.7%	0.03
+	632	15.7%	0.02
++	632	20.9%	0.03
+++	632	10.8%	0.02
**Test d'appétit**			
Bon	568	83.1%	0.04
Moyen	568	16.5%	0.04
refusé	568	0.4%	0.00
**z-score P/T à la sortie**	408	-1.26	0.12
**z-score P/A à la sortie**	413	-3.18	0.16
**Raison de sortie**			
Transfert au SST(Hôpital)	665	2.0%	0.01
Guéri	665	85.4%	0.02
Abandon	665	5.1%	0.01
Décès	665	1.4%	0.00
Non répondant	665	5.1%	0.01
**Gain pondéral en grammes par poids initial en kg et par jour de traitement, depuis l'entrée jusqu'à la sortie**	536	3.92	0.33
**La durée du traitement en jours, depuis l'entrée jusqu'à la sortie (uniquement pour ceux rapportés comme guéris)**	556	58.89	3.19

Source: calculs des auteurs à partir de l'enquête de base FBP Nutrition

Tableau 19. Performance du STA comparée aux directives nationales

	Enquête	Directives nationales	Atteint?
**Taux de guérison**	85.4% (N=665)	>75%	✓
**Taux de transfert**	2.0% (N=665)		
**Taux de décès**	1.4% (N=665)	<5%	✓
**Taux d'abandon**	5.1% (N=665)	<13%	✓
**Taux de non réponse**	5.1% (N=665)	<7%	✓
**Gain pondéral en grammes par poids initial en kg et par jour de traitement, depuis l'entrée jusqu'à la sortie**	3.92 (N=536)	>4-6	✗
**La durée du traitement en jours, depuis l'entrée jusqu'à la sortie (uniquement pour ceux rapportés comme guéris)**	58.89 (N=556) (med:50)	<30	✗

Source: calculs des auteurs à partie de l'enquête de base FBP Nutrition et MSPLS(2010)

Tableau 20. Traitements systématique et diététique en STA

	Nombre de fiches	moyenne	Déviation standard
**Traitement systématique**			
Amoxicilline	679	47.6%	0.05
vitamine A	679	37.8%	0.05
Acide folique	679	32.5%	0.05
al/mebendazole	679	64.7%	0.05
amoxicilline et al/mebendazole	679	44.3%	0.05
**Traitement diétetique**			
Nombre de visites sans aliments thérapeutiques sur le nombre total de visites (si nombre de visites >2)	850	18.1%	0.03

Source: calculs des auteurs à partir de l'enquête de base FBP Nutrition

Synthèse

Au total, 971 dossiers médicaux individuels issus des services SSN et 963 des services STA ont été retranscrits.

Le premier constat est une mauvaise documentation : mauvais archivage des dossiers, remplissage des fiches individuelles de suivi non systématiques en ce qui concerne les données anthropométriques à l'admission et à la sortie, le traitement, le résultat du programme, etc. Ceci rend difficile l'évaluation de la performance des services SSN et STA.

Néanmoins, les indicateurs de performance calculés pour les deux programmes STA et SSN ont atteint la plupart des recommandations nationales, notamment en termes de taux de guérison, mais pas en termes de gain de poids moyen quotidien et de durée de séjour dans le programme. Cela étant, 10% des

cas reportés comme guéris dans le service SSN avaient encore un z-score de rapport poids pour taille inférieur à -2 (indiquant en fait une non-guérison).

Parmi les fiches individuelles de suivi SSN, environ 20% ont reçu un mauvais diagnostic : les patients étaient dans un service de prise en charge de la malnutrition aigüe modérée (MAM ; service SSN) alors que, selon les données anthropométriques renseignées sur la fiche, ils auraient dû recevoir un diagnostic de malnutrition aigüe sévère (MAS) avec une référence au service STA.

Dans le service STA, il y avait également de mauvais diagnostics : la moyenne du z-score du rapport poids pour taille était au-dessus de -3, ce qui suggère qu'en moyenne les enfants entrant dans le service ne souffraient pas de MAS (mais de MAM) ; cela étant, le manque de documentation concernant les périmètres brachiaux (PB) et les œdèmes dans les dossiers médicaux individuels fait qu'il est difficile d'évaluer la qualité du diagnostic à l'admission dans ce service.

Qualité des services de santé

Observations directes de consultations pédiatriques (Questionnaire C)

En général

L'analyse du questionnaire C se concentre principalement sur l'interrogatoire et l'examen observés et dans une moindre mesure sur les tests de laboratoire demandés, les diagnostics et les traitements. Comme nous ne pouvons pas être sûrs que les diagnostics sont corrects, il est difficile de commenter les traitements administrés et une exploration approfondie de ces décisions cliniques est au-delà de la portée de ce rapport. En tant que tel, notre principal point d'attention s'est borné aux directives internationales (PCIME, manuel pédiatrique de l'OMS) et nationales (directives nationales sur la malnutrition 2010) dans les étapes initiales de la consultation.

Un des premiers aspects de l'approche PCIME est de vérifier trois signes de danger pour l'enfant : l'enfant est-il capable d'avaler en toute sécurité (boisson ou allaitement), a-t-il eu ou a-t-il des vomissements, a-t-il eu des convulsions (OMS 2014) ? Au total, deux cas (0,3%) ont été interrogés sur ces trois questions par les prestataires. Trois cent vingt cas (62%) n'ont été interrogés sur aucun de ces trois signes de danger.

L'autre priorité de la PCIME est d'accorder une attention particulière aux «principaux symptômes» (WHO 2014: 2-5). Cela passe par une série de questions sur la présence de diarrhée, de toux, de difficultés respiratoires, de fièvre et de problèmes d'oreille. Dans cette enquête, au total deux cas (0,3%) ont été interrogés sur toutes les cinq questions, et 59 cas (12%) n'ont été interrogés sur aucun de ces symptômes. Un autre aspect de la PCIME est de vérifier le statut vaccinal de l'enfant : au total, 70 cas (14%) ont été interrogés pour vérifier si leurs vaccinations étaient à jour.

D'un point de vue plus global, dans notre grille d'observation des consultations, sur un total de 31 questions dans la section de l'interrogatoire, il y avait 23 questions autonomes (c'est-à-dire des questions qui ne se rapportent pas à la réponse de la question précédente ; une question non-autonome est par exemple celle sur la durée de la diarrhée – elle ne sera posée que si une présence de la diarrhée

a été rapportée). Aucun patient n'a été interrogé sur toutes les 23 questions. Trois cas (0,6%) ont été interrogés sur 13 questions ; ce fut le nombre maximum de questions posées. Quatre cas (0,8%) n'ont été interrogés sur aucune de ces 23 questions. Le Tableau 21 résume les principaux résultats de la section du questionnaire de l'interrogatoire.

Tableau 21. Nombre de cas interrogés sur les signes de danger, les principaux symptômes et les questions autonomes

Questions relatives à…	cas interrogés n (%) N=514
Aux signes de danger - toutes les trois posées	**2 (0.3)**
capable de boire/avaler	24 (5)
vomissements	164 (32)
présence de convulsions	39 (8)
Aux cinq principaux symptômes - toutes les cinq posées	**2 (0.3)**
diarrhée	267 (52)
toux	261 (51)
difficultés respiratoires	31 (6)
fièvre	302 (59)
problèmes d'oreilles	59 (12)
Au statut vaccinal	**70 (14)**
Toutes les questions autonomes du questionnaire C (23)	*0 (0)*
Nombre maximum posé: 13	*3 (0.6)*
Aucune des questions posées	*4 (0.8)*

Il y avait 19 examens physiques figurant dans le questionnaire. Aucun des cas observés n'a subi tous les examens. Le nombre maximal d'examens effectués a été de 14 (un seul cas) ; pour 88 cas (17%) aucun des examens physiques énumérés dans le questionnaire n'a été effectué sur eux.

Si nous retirons de la liste des examens les aspects qui sont plus spécifiques à la malnutrition (poids, taille, interprétation de la courbe de croissance, PB, oedèmes, test de l'appétit et le calcul des z-scores P/T), nous restons encore avec aucun cas chez qui tous les examens restants (au total de 12) ont été effectués ; le nombre maximum d'examens réalisés était de huit et n'a été observé que pour un cas.

En outre, aucun des cas n'avait tous les trois principaux signes vitaux (température, pouls et le rythme respiratoire) mesurés. Vingt-six cas (5%) ont eu deux des signes vitaux mesurés, 283 cas (55%) avaient un seul mesuré et 205 cas (40%) n'avaient aucun des signes vitaux enregistrés. Le Tableau 22 présente les résultats sur les examens physiques.

Tableau 22. Nombre de cas avec examens et signes vitaux effectués

Examen physique	cas observés n (%) N=512
Tous les examens du questionnaire C (19)	0 (0)
Nombre maximum réalisé: 14	1 (0.2)
Aucun examen effectué	88 (17)
**Signes vitaux - tous les trois ont été mesurés**	0 (0)
température	300 (58)
pouls	2 (0.4)
rythme respiratoire	33 (6)
pas de signes vitaux pris	205 (40)

Principaux symptômes PCIME: diarrhée

Deux cent soixante-sept cas (52%) ont été interrogés pour savoir si la diarrhée était présente. Parmi ceux-ci, il y avait 139 réponses positives. Après la question demandant la présence de la diarrhée, il y a six autres questions –présentes dans la grille d'observation des consultations– qui explorent les signes et les symptômes connexes importants : la durée de la diarrhée, la présence des vomissements, la présence d'une diarrhée sanglante (un des signes de danger selon la PCIME), la présence de crampes abdominales, la présence de la fièvre ou un changement récent dans le régime alimentaire. Parmi ces 139 cas de diarrhée, aucun n'a été interrogé sur six ou même cinq de ces questions complémentaires. Pour quatre cas (3%), aucune de ces questions complémentaires n'a été posée.

Passant à l'examen physique, il y a quatre examens-clé pour évaluer la déshydratation (qui peut entraîner de la diarrhée): la fréquence du pouls, les signes de déshydratation (WHO 2013a), un pli cutané s'effaçant lentement et les yeux enfoncés récemment. Parmi les 139 cas de diarrhée, seulement cinq cas (4%) ont eu tous ces examens effectués sur eux ; pour 51 cas (37%) aucun de ces examens n'a été pratiqué. Le Tableau 23 résume les questions posées et les examens effectués sur les cas de déclaration de la diarrhée.

Pour 10% des cas de diarrhée (comme indiqué par le personnel soignant) il a été posé un diagnostic de «diarrhée», 8% ont été qualifiés de «gastro-entérites» et 32% d'«infection parasitaire». Fait intéressant, sur les 125 cas rapportés comme diagnostiqués d'infection parasitaire (y compris ceux pour qui il n'est pas signalé de diarrhée), seulement 59% avaient réalisé un test des selles pour rechercher les parasites.

Sur les 139 cas de diarrhée, il y avait seulement trois cas signalés comme ayant des signes de déshydratation, mais étant donné que seulement quelques enfants avaient bénéficié d'un examen physique pour rechercher des signes de déshydratation, ce chiffre est susceptible de ne pas être fiable. De ces trois cas de déshydratation, un seul a eu une prescription de solution de réhydratation orale (SRO). Un total de 54 cas (39%) des 139 rapportés comme ayant de la diarrhée ont reçu une prescription d'antibiotique. Un antibiotique contre la diarrhée peut être approprié si il y a des selles sanglantes, mais il y avait seulement deux cas qui ont déclaré des selles sanglantes et de ceux-là un seul a effectivement reçu une prescription d'antibiotique.

Tableau 23. Questions et examens effectués sur les cas pour lesquels est déclarée la présence de diarrhée

Questions et examens complémentaires	cas observés n (%) N = 139
**Questions explorant l'histoire de la diarrhée (6)**	
cas interrogés sur toutes les six questions	0 (0)
cas sans aucune question complémentaire posée	4 (3)
durée de la diarrhée	97 (70)
sang dans les selles	29 (21)
crampes abdominales	33 (24)
vomissements	58 (42)
alimentation changée récemment	3 (2)
fièvre	89 (64)
**Examens exploratoires de la déshydratation (4)**	
cas avec tous les 4 examens	5 (4)
cas sans aucun examen	51 (37)
pouls	0 (0)
signe de déshydratation	24 (17)
pli cutané s'effaçant lentement	16 (12)
yeux enfoncés récemment	7 (5)

Principaux symptômes PCIME: toux et difficultés respiratoires

Pour 261 cas (51%), il a été demandé s'il y avait présence de toux ; 153 cas (64%) ont répondu positivement. Comme pour l'exemple de la diarrhée, la présence d'une toux devrait susciter un certain nombre de questions exploratoires pour compléter l'histoire de la toux: présence de fièvre, durée de la toux, si la toux est sèche ou productive, et s'il y a des difficultés respiratoires associées (un signe de danger selon la PCIME). Deux cas (1%) des 153 cas ont été interrogés sur toutes les quatre questions exploratoires de la toux, et pour 27 cas (18%) il n'a été posé aucune de ces questions. Le Tableau 24 donne plus de détails sur le nombre de cas pour lesquels des questions au sujet de la toux ont été posées.

Tableau 24. Questions posées aux cas ayant déclaré la présence de la toux

Questions cliniques	cas interrogés (%) n = 153
**Questions exploratoires de l'histoire de la toux (4)**	
cas interrogés sur toutes les 4 questions	2 (1)
cas sans aucune question complémentaire posée	27 (18)
fièvre	97 (64)
durée de la toux	69 (45)
toux sèche ou productive	23 (15)
difficultés respiratoires associées	14 (9)

Au total, il y a eu 21 cas où l'accompagnant a rapporté à l'AS des difficultés respiratoires chez leur enfant. Parmi ceux-ci, 12 cas (57%) ont eu leur température prise, mais seulement cinq cas (24%) ont eu leur fréquence respiratoire mesurée et trois cas (14%) ont eu leurs poumons auscultés. Trente-trois cas sur un total de 512 cas (6%) ont eu leur fréquence respiratoire mesurée. Sur les 19 cas (parmi ces 33) pour lesquels on a un chiffre réel enregistré pour la fréquence respiratoire, considérant l'âge de l'enfant, sept étaient normaux et 12 étaient anormaux.

Pour vingt cas, l'AS a donné le diagnostic de pneumonie. Selon la définition de l'OMS de la pneumonie chez un enfant, il doit y avoir la présence d'une augmentation du rythme respiratoire et les signes thoraciques anormaux (WHO 2013a). Aucun des 20 cas n'avait ces signes rapportés tous les deux à la fois. Six de ces cas avaient rapporté une augmentation du rythme respiratoire et quatre cas avaient des signes thoraciques anormaux. Un regard plus en profondeur sur les données de chacun de ces cas suggère que dans la plupart des cas, soit la seule présence de toux, soit la présence de toux associée à de la fièvre, ont suffi comme symptômes pour mener à ce diagnostic. Dix-neuf des 20 cas signalés comme ayant une pneumonie ont eu une prescription d'antibiotiques. Dans 11 des 20 cas (55%), il a été prescrit de l'amoxicilline, et dans 7 (35%) du co-trimoxazole.

Principaux symptômes PCIME: fièvre

Dans 302 cas (59%), l'AS a demandé la présence de fièvre. Parmi ces 302 cas, il y avait 191 réponses positives. En tenant compte de ces 191 cas et de ceux qui ont été trouvés ayant de la fièvre à l'examen physique (température axillaire> 37,5 ° C), il y avait un total de 239 cas d'enfants ayant de la fièvre selon la réponse de l'accompagnant et/ou l'examen physique de fièvre. Ces 239 cas de fièvre, plutôt que 191 cas seulement provenant des réponses de l'accompagnant, ont été utilisés dans les calculs qui suivent. En effet, même si un accompagnant ne rapporte initialement pas de fièvre, une fièvre trouvée lors de l'examen physique doit inciter l'AS à revenir en arrière et à poser d'autres questions et à effectuer des examens physiques supplémentaires ou des tests de laboratoire.

Ainsi, quand il y a présence de la fièvre, il y a quatre questions complémentaires explorant les signes et les symptômes connexes qui sont importantes: la périodicité de la fièvre (par exemple, plus fréquente la nuit, le jour, etc.), la présence de raideur de la nuque, présence de convulsions (un des signes de danger

selon PCIME) et l’altération de la conscience. Trois cas (1%) parmi les 239 cas ont été interrogés sur toutes les quatre questions ; pour 152 cas (64%), aucune de ces questions n’a été posée.

Parmi les questions figurant dans ce questionnaire, il y a six questions qui aideraient à trouver la source de la fièvre (voir Tableau 25). Sur les 239 cas de fièvre, aucun d'entre eux n’a été interrogé sur l'ensemble de ces six questions. Pour 13% des cas, aucune de ces questions n’a été posée.

Il y a sept examens physiques différents dans ce questionnaire qui sont importants dans le contexte de la fièvre et/ou susceptibles d'aider à trouver la source de la fièvre (voir Tableau 25). Aucun des 239 cas de fièvre n’a reçu l’ensemble de ces sept examens physiques ; chez 132 cas (55%), aucun de ces examens n’a été pratiqué. Le Tableau 25 présente les résultats de l'exploration de la fièvre à travers l'interrogatoire et l'examen.

Tableau 25. Questions et examens effectués sur les cas déclarés avec présence de fièvre

Questions et examens complémentaires	Cas interrogés n (%) N = 239
**Questions exploratoires de l’histoire de la fièvre (4)**	
cas interrogés sur toutes les 4 questions	3 (1)
cas sans aucune question posée	152 (64)
périodicité de la fièvre	68 (28)
raideur	21 (9)
convulsions	28 (12)
altération de l’état général	6 (3)
**Questions sur la source de la fièvre (6)**	
cas interrogés sur toutes les six questions	0 (0)
cas sans aucune question posée	30 (13)
diarrhée	141 (59)
sang dans les selles	8 (4)
toux	143 (60)
difficultés respiratoires	17 (7)
vomissements	94 (39)
préoccupation/plaintes de douleur à l’oreille	35 (15)
**Examens physiques relatifs à la présence de fièvre (7)**	
cas avec tous les 7 examens pratiqués	0 (0)
cas sans aucun examen pratiqué	132 (55)
pouls	1 (0.4)
rythme respiratoire	21 (9)
auscultation pulmonaire	6 (3)
raideur de la nuque	4 (2)
examen de l’oreille, du nez et de la gorge	57 (24)
examen du foie et de la rate	22 (9)
examen de la peau	36 (15)

Sur le point des examens de laboratoire, parmi les 239 cas de fièvre, 159 (65%) ont subi un test de paludisme (test microscopique ou rapide). Quatre-vingt-trois cas (35%) des 276 cas sans fièvre (ni déclarée, ni apparue à l'examen) ont également eu un test de paludisme effectué.

Il y a beaucoup de différentes causes de fièvre, et explorer les diagnostics corrects et le traitement de ceux-ci est au-delà de la portée de ce rapport. Cependant, si nous regardons seulement le paludisme, il y avait 93 cas (39%) des 239 cas de fièvre ayant reçu un diagnostic de paludisme (sept graves et 86 non compliqués). Parmi ces 93 cas, 83 (90%) avaient subi un test de paludisme.

Principaux symptômes PCIME: problèmes d'oreilles

Dans 59 cas (12%), l'AS a demandé si l'enfant avait des problèmes d'oreille (c'est-à-dire, il était demandé si l'enfant avait des douleurs dans ou autour de l'oreille ou si l'enfant portait la main àl'oreille). Sur ces 59 cas qui ont été interrogés, 54 cas ont donné une réponse ; la réponse a été positive pour 12 d'entre eux (22%). Dans neuf de ces 12 cas avec problème d'oreille, il a aussi été démandé s'il l'enfant avait de la fièvre; aussi, neuf cas ont eu leur température mesurée. Parmi les 12 cas ayant déclaré un problème d'oreille, 7 (59%) ont eu les oreilles et la gorge examinées. Sept cas (59%) ont reçu un diagnostic d'otite moyenne aiguë ; et il n'y a pas de cas chez qui ce diagnostic a été posé alors que les oreilles et la gorge n'avaient pas été examinés.

Nutrition

Sept questions figurant dans la grille d'observation des consultations portent spécifiquement sur la nutrition : les sept sont pertinentes pour les enfants de moins de deux ans, tandis que cinq sont pertinentes pour les enfants de plus de deux ans (les questions sur l'allaitement sont retirées ; voir Tableau 26).

Parmi les 250 enfants de moins de deux ans, aucun n'a été questionné sur toutes les sept questions de nutrition. Dans 106 cas (43%), aucune de ces questions n'avait été posée.

Parmi les 200 enfants âgés de plus de deux ans, aucun n'a été questionné sur toutes les cinq questions ; dans 93 cas (47%), aucune des questions relatives à la nutrition n'a été posée.

Dans la grille d'observation, six aspects de l'examen physique sont spécifiquement liés à l'état nutritionnel de l'enfant: poids, taille, périmètre brachial, oedèmes, dessiner / discuter de la courbe de croissance, et calcul du rapport P/T. Au total, 13 cas (3%) ont vu tous ces six examens réalisés dans le cadre de leurs soins ; 211 cas (41%) n'ont reçu aucun de ces examens.

Au total, 122 cas (24%) ont reçu des conseils de nutrition. Les catégories des conseils les plus fréquemment rapportées sont présentées dans le Tableau 27.

Tableau 26. Questions et examens relatifs à la nutrition exécutés

Questions et examens relatifs à la nutrition	Cas interrogés n (%)
**Questions de nutrition**	N=250
cas de moins de 2 ans interrogés sur les 7 questions	0 (0)
cas de moins de 2 ans sans aucune question posée	106 (42)
	N=200
cas de plus de 2 ans interrogés sur les 5 questions	0 (0)
cas de plus de 2 ans sans aucune des cinq questions posées	93 (47)
	N=250
encore allaité (< 2 ans uniquement)	76 (30)
épisodes d'allaitement depuis ce matin/hier	15 (6)
	N=514
appétit	226 (44)
autres aliments complémentaires habituellement donnés	74 (15)
âge d'introduction des aliments complémentaires	16 (3)
Qu'est ce qu'il a mangé depuis hier	17 (4)
alimentation changée récemment	6 (1)
**Examens de Nutrition / tâches**	N=513
tous les six examens / tâches	13 (3)
aucun des examens / tâches	211 (41)
poids	268 (52)
taille	165 (32)
PB	153 (30)
oedèmes	64 (13)
courbe de croissance	37 (7)
rapport P/T	95 (19)

Tableau 27. Fréquence et catégorie de conseils de nutrition donnés aux patients

Catégories de conseils de nutrition	Nb de patients ayant reçu ces conseils n (%) N = 122*
L'apport de denrées alimentaires spécifiques	56 (46)
Les trois catégories d'aliments (rapportés comme les glucides, les protéines et les graisses)	13 (11)
Une surveillance active de l'alimentation	1 (1)
Les pratiques d'hygiène en ce qui concerne la nourriture / alimentation	6 (5)
Fréquence d'alimentation («trois fois par jour»)	3 (3)
Augmenter la fréquence de l'allaitement maternel (enfants de 2 ans et moins)	5 (4)

* les fréquences sont calculées par rapport au nombre de patients ayant reçu des conseils de nutrition (N=122).

Malnutrition

Pour une appréciation complète de la précision du diagnostic et l'orientation des cas de malnutrition aiguë, les résultats du questionnaire C ont été confrontés aux mesures anthropométriques et à l'évaluation des œdèmes faite par les enquêteurs avec le questionnaire S, en utilisant les équipements propres à l'enquête (utilisés comme mesures de référence).

Malnutrition Aigüe Modérée (MAM)

Selon les résultats du questionnaire S, il y avait 51 cas (10%) de MAM dans l'échantillon. Cependant, seuls 12 enfants (3%) au total ont reçu un diagnostic de MAM par l'agent de santé lors des observations de consultations (questionnaire C). Sur ces 12 cas, huit ont été orientés vers le service approprié pour les enfants de MAM, c'est-à-dire dans un service de supplémentation nutritionnelle (SSN) ; il s'agit d'un programme ambulatoire pour la prise en charge des enfants souffrant de MAM.

Si on considère la totalité de l'échantillon (i.e. pas seulement les cas diagnostiqués MAM), au total 11 enfants (et pas seulement huit) ont été orientés vers le SSN lors des consultations. En utilisant les données anthropométriques collectées à la sortie (questionnaire S), il apparaît que sur ces 11 cas, seulement deux étaient des cas réels de MAM ; quatre souffraient en fait de malnutrition aiguë sévère (MAS), et cinq étaient normalement nourris.

Parmi les 12 enfants qui ont été diagnostiqués comme souffrant de MAM, six ont reçu des conseils de nutrition. Le Tableau 28 présente les types de conseils de nutrition donnés aux six enfants.

Tableau 28. Conseils de nutrition donnés aux accompagnants d'enfants qui ont été diagnostiqués comme des cas de MAM par l'agent de santé

Conseil nutrition	Nb de patients MAM ayant reçu ce conseil N=12*
S'assurer que l'enfant a touts les «trois groupes d'aliments» (non spécifié ce que ces groupes étaient)	2
Donnez une alimentation équilibrée, incluant les haricots, les petits poissons, œufs, lait, bouillie, fruits, noix	1
Poursuivre le programme SSN	1
Donner à l'enfant les légumes verts et superviser tous les repas, si possible	1
Donnez plus de fruits et de l'huile de palme	1

Note : * Au total, 12 cas ont été diagnostiqués MAM par les AS.

Malnutrition Aigüe Sévère (MAS)

Selon les données anthropométriques et d'œdème collectées à la sortie (questionnaire S), il y avait 27 cas (6%) de MAS. Selon les diagnostics des AS collectés lors des observations de consultations (questionnaire C), il y avait un cas de MAS non compliqué et un cas de MAS compliquée. Pour le cas de MAS compliquée, l'agent de santé a expliqué son diagnostic de la façon suivante : « en raison de l'anorexie et des vomissements après tous les repas ». Cet enfant a été envoyé à des services d'hospitalisation pour les cas de MAS compliquées, i.e. le service de stabilisation (SST). Le cas de MAS simple a été orienté vers le service de prise en charge ambulatoire pour les cas MAS, i.e. le service thérapeutique ambulatoire (STA).

Au total, cinq cas ont été référés au STA. De ces cinq, deux étaient des cas réels de MAS (en utilisant les données du questionnaire S) et trois étaient normalement nourris ; y compris l'enfant que l'agent de santé avait diagnostiqué comme un cas simple de MAS dans le questionnaire C. Au total, un enfant a été référé au SST et ce fut le cas précité de MAS compliquée, correctement diagnostiqué par l'agent de santé lors de la consultation.

Les deux enfants diagnostiqués avec MAS ont reçu des conseils diététiques.

Entretiens à la sortie (Questionnaire S)

Compréhension du diagnostic

Les enquêteurs ont posé aux accompagnants la question 'qu'est-ce qui ne va pas avec votre enfant?' après la consultation avec l'agent de santé. Sur 516 accompagnants interrogés, 341 (66%) ont donné une réponse qui correspond soit à un diagnostic soit à un ensemble de symptômes ; 171 accompagnants (34%) ont en revanche déclaré ne pas savoir ce qui n'allait pas avec leur enfant après la consultation. Parmi les 341 accompagnants qui ont déclaré que l'agent de santé leur avait dit ce qui n'allait pas avec leur enfant, 215 (63%) ont donné un diagnostic réel alors que 126 (37%) ont raconté les signes et symptômes dont leur enfant souffrait.

Parmi les 171 accompagnants qui n'ont pas déclaré de diagnostic ou d'ensemble de symptômes, 101 (59%) ont répondu qu'ils ne connaissaient pas le diagnostic parce que l'agent de santé ne les en avait pas informés. Trente-cinq accompagnants (20%) ont répondu qu'ils ne savaient pas, sans autre explication, et 23 (13%) ont déclaré que l'agent de santé avait prescrit des médicaments seulement, mais ne les avait pas informés du diagnostic.

Aussi, sur les 341 cas qui ont déclaré que l'agent de santé leur avait dit ce qui n'allait pas avec leur enfant, deux rapporté le diagnostic de «malnutrition» (sans distinction si elle était aiguë ou chronique, modérée ou sévère). Ce chiffre est à comparer avec les 14 cas de malnutrition aiguë (12 MAM et 2 MAS) diagnostiqués par les AS lors des consultations observées (questionnaire C), et les 78 cas réels de malnutrition aiguë (51 MAM et 27 MAS) selon les mesures anthropométriques et l'évaluation des œdèmes faites par les enquêteurs à la sortie (questionnaire S).

Compréhension du traitement

Cinq cent douze accompagnants (99%) ont donné une réponse à la question concernant le traitement prévu pour leur enfant : 324 (76%) ont déclaré que l'AS avait prescrit des médicaments, mais n'avait pas donné d'autres conseils ou d'autre explication sur l'état ou le traitement de l'enfant. Cinquante-deux (10%) ont indiqué que l'agent de santé n'a pas donné des conseils, mais avait prescrit des médicaments et a dit à l'accompagnant de revenir pour un suivi s'il n y a pas d'amélioration malgré le traitement.

Conseils nutritionnels

Cinq cent onze accompagnants (99%) ont donné une réponse à la question quant à savoir si on leur avait donné des conseils sur la nutrition ou non lors de la consultation. Parmi ces 511 accompagnants, 393 cas (76%) ont indiqué qu'ils n'avaient pas reçu de conseils de nutrition. Le Tableau 29 présente les principales catégories de conseils donnés (l'accompagnant peut avoir reçu une ou plusieurs catégories de conseils).

Tableau 29. Catégories de conseils nutritionnels donnés aux accompagnants au cours de la consultation

Conseil nutrition	Nombre d'accompagnants ayant reçu les conseils n (%) N = 118*
L'apport de denrées alimentaires spécifique	75 (64)
Trois catégories d'aliments (rapportées comme les glucides, les protéines et les graisses)	13 (11)
Une surveillance active de l'alimentation	1 (1)
Les pratiques d'hygiène en ce qui concerne la nourriture / alimentation	11 (10)
Fréquence d'alimentation («trois fois par jour»)	5 (4)
Augmenter la fréquence de l'allaitement maternel (enfants de 2 ans et moins)	2 (2)

Note : *Au total, 118 accompagnants ont dit avoir reçu des conseils de nutrition ; les pourcentages entre parenthèses sont donnés en rapport de ces 118 individus.

Niveaux de satisfaction de l'accompagnant

Il y avait une grande variété de commentaires sur les aspects qui avaient été le plus satisfaisants pour les accompagnants en ce qui concerne leur visite au centre de santé ce jour-là, mais ils peuvent être regroupés en trois catégories principales : l'organisation, le contenu médical et les aspects de la communication. Les aspects organisationnels comprennent l'organisation du processus d'enregistrement et d'attente, l'état des bâtiments et d'autres centres, les références vers d'autres centres de santé, etc. Les aspects du contenu médical comprennent la façon dont l'enfant a été examiné et l'interrogatoire de l'accompagnant, les tests de laboratoire et les médicaments prescrits, les conseils donnés, les mesures anthropométriques prises, etc. Les aspects de communication comprennent l'accueil des accompagnants, l'écoute active, etc. Les accompagnants peuvent avoir reçu une ou plusieurs catégories de conseils. Le Tableau 30 présente ces résultats.

Tableau 30. Principales catégories d'aspects qui ont le plus satisfait les accompagnants en ce qui concerne la visite actuelle au centre de santé

Catégories	Nombre d'accompagnants n (%) N = 512
aspects organisationnels	44 (9)
aspects du contenu médical	296 (58)
aspects de communication	233 (46)

En analysant les réponses plus en détail, l'aspect le plus fréquemment cité comme élément de satisfaction était la manière dont ils avaient été accueillis par le personnel du centre, avec 207 accompagnants (40%) ayant déclaré cela. Cent seize accompagnants (23%) ont déclaré que c'était spécifiquement la façon dont leur enfant a été examiné et consulté qui était la plus satisfaisante pour

eux. Quatre-vingt-deux accompagnants (18%) ont déclaré la prescription de médicaments qu'ils ont reçue comme étant l'aspect le plus satisfaisant, et 74 accompagnants (14%) ont signalé la demande de tests de laboratoire pour leur enfant comme le plus satisfaisant.

Onze cas (2%) ont déclaré qu'il n'y avait rien de leur visite qui les avaient satisfait ce jour-là ou même qu'ils étaient insatisfaits. Quarante-neuf accompagnants (10%) ont déclaré qu'ils étaient satisfaits par la visite du jour, mais que cela avait été une exception par rapport à leurs visites habituelles. De même, 28 accompagnants (5%) ont déclaré qu'ils étaient satisfaits du fait que leurs enfants aient été mesurés et pesés et ont déclaré que ce n'était pas une pratique normale.

Sur la question concernant ce qui avait été l'aspect le moins satisfaisant de la visite pour les accompagnants, 442 accompagnants (86%) ont déclaré qu'il n'y avait rien sur quoi ils n'étaient pas satisfaits. L'aspect le moins satisfaisant le plus fréquemment rapporté au cours de la visite, était le temps d'attente passé dans le centre de santé (pour la consultation, examens de laboratoire et les médicaments), avec 32 accompaganants (6%) ayant déclaré cela.

Suggestions pour améliorer les services

Sur les 514 accompagnants qui ont répondu à la question de ce qui pourrait être fait pour améliorer les services dans le centre de santé, 416 d'entre eux (81%) avaient une suggestion, mais celles-ci étaient très variées. Le Tableau 31 montre les réponses les plus fréquemment rapportées.

Tableau 31. Suggestions d'améliorations possibles des services

Suggestion	Nombre d'accompagnants n (%) N = 514
Améliorer la disponibilité des médicaments dans la pharmacie	84 (16)
Aucune amélioration nécessaire	82 (16)
S'assurer que tous les services sont exécutés à temps	60 (13)
Améliorer l'attitude du personnel en ce qui concerne le respect du patient et l'accueil	55 (11)
Donner la priorité aux patients eu égard seulement à leurs besoins médicaux	30 (6)
S'assurer que les patients sont accueillis comme le jour de l'enquête et non pas comme les jours précédents	23 (4)
Embaucher un médecin et/ou une infirmière spécialisée en pédiatrie	22 (4)
Améliorer la disponibilité des suppléments pour les services de malnutrition	17 (4)
Prendre les mesures anthropométriques de chaque enfant à chaque visite	10 (2)
Veiller à ce que tous les cas soient traités de manière égale et sans népotisme	8 (2)
Je ne sais pas	15 (3)
Aucune suggestion	97 (18)

Mesures anthropométriques

Des t-tests ont été utilisés pour comparer les mesures anthropométriques prises par l'AS et par les enquêteurs à la fois en utilisant les équipements des centres de santé et aussi ceux de l'enquête. Il n'y avait pas de différence significative observée entre les poids pris par l'AS et ceux pris par les enquêteurs utilisant les balances des centres de santé (p=0,52, N=404). Par contre, les tailles prises par les AS étaient significativement inférieures à celles prises par les enquêteurs en utilisant la toise du centre de santé (p=0,02, N=269). Les mesures du PB prises par l'AS étaient également significativement inférieures à celles prises par les enquêteurs avec le même mètre ruban (p=0,00, N=224). Enfin, les poids mesurés par les enquêteurs en utilisant les balances du centre de santé étaient constamment plus élevés (malgré le calibrage des balances avant chaque mesure) que ceux pris également par l'enquêteur, mais en utilisant les balances de l'enquête (balance mère/enfant SECA 876) (p=0,00, N=497).

Synthèse

Globalement, cette enquête a observé de faibles niveaux de performance des AS par rapport au respect des protocoles internationaux et nationaux telles que la PCIME et le protocole national de PEC de la malnutrition. Par exemple, à partir des observations des consultations (N=514), moins de 1% des enfants ont été interrogés sur les trois signes de danger et moins de 1% ont se sont vus poser des questions sur les cinq symptômes principaux de la PCIME. En outre, dans les cas où les AS ont posé des questions à propos de l'un des cinq symptômes principaux, ils ont ensuite systématiquement omis de poser d'autres questions exploratoires ou d'effectuer des examens physiques pertinents à l'égard de ces symptômes et des signes si ils ont reçu une réponse positive. Par ailleurs, seulement 13,5% des enfants ont été

interrogés sur leur statut de vaccination et aucun des enfants observés lors des consultations n'a passé les examens physiques de base dans la liste fixe du questionnaire.

Concernant les aspects liés à la malnutrition, aucun des enfants n'a été interrogé sur toutes les questions de nutrition spécifiques (ajustement pour l'âge), et seulement 2,5% des enfants ont passé tous les examens physiques liés à la nutrition. Le poids est la mesure la plus souvent prise (52% des cas), la taille et le périmètre brachial (PB) sont les prochaines mesures les plus fréquemment prises (32% et 30% respectivement). Peu d'enfants ont été évalués pour la présence d'œdème (12,5%), un facteur qui peut faire basculer le diagnostic d'un enfant ayant un z-score de poids pour taille ou un PB qualifiés de normaux ou de MAM, à un diagnostic de MAS.

Parmi les 514 évaluations des enfants observés en consultation et à la sortie le jour de l'enquête, 51 étaient des cas de MAM (selon les données anthropométriques collectées par les enquêteurs à la sortie), mais seulement 12 de ces enfants avaient effectivement reçu un diagnostic de MAM par les agents de santé (AS) lors des consultations. De même, 27 enfants souffraient de MAS (selon les données anthropométriques collectées à la sortie par les enquêteurs), mais seulement deux de ces enfants ont effectivement reçu ce diagnostic par l'AS lors des consultations. Un problème fondamental qui pourrait expliquer en partie l'erreur des diagnostics régulièrement observés de malnutrition aiguë est le fait que seulement 14% des formations sanitaires (FOSAs) avait tout l'équipement nécessaire pour les mesures anthropométriques la fois disponible et fonctionnel. En outre, les mesures de taille enregistrées par les AS sont significativement inférieures à celles des enquêteurs - en utilisant le même équipement (de la FOSA) ; ceci pourrait conduire à des calculs de z-scores plus élevés et ainsi à un sous-diagnostic de la malnutrition aiguë. Au contraire, les PBs pris par les AS étaient significativement inférieurs à ceux pris par les enquêteurs ce qui pourrait conduire à un sur-diagnostic de la malnutrition aiguë, mais cela n'a pas été observé dans les résultats. Un autre facteur qui pourrait avoir conduit au sous-diagnostic de la malnutrition aiguë vu dans cette enquête, était la différence statistiquement significative observée dans les poids pris par les enquêteurs avec les balances de la FOSA par rapport aux balances de l'enquête : les balances de l'enquête ont toujours montré un poids inférieur. Cette différence est le résultat d'un problème relatif à l'équipement plutôt que d'erreur humaine (avec le même équipement, les mesures de poids étaient similaires).

Un des aspects les plus négligés de la consultation était la communication entre les AS et les accompagnants : on a calculé des pourcentages très faibles d'AS qui ont donné des conseils de santé ou de nutrition aux accompagnants ou qui ont expliqué correctement le diagnostic ou le traitement pour leur enfant. Les entretiens à la sortie avec les accompagnants des enfants ont confirmé cette mauvaise performance en termes de communication. Cela étant, très peu d'accompagnants (2%) ont signalé qu'absolument rien ne les avait satisfaits à propos de leur visite ce jour-là. Il est intéressant de noter, bien que la majorité d'entre eux ait déclaré que rien ne les avait insatisfaits, que 81% d'entre eux ont effectivement eu des suggestions d'amélioration - ce qui pourrait être une façon plus «confortable» d'exprimer leurs insatisfaction avec les services sans le dire directement.

Connaissances et compétences des agents de santé

Vignettes cliniques (Questionnaire V)

1. Vignette n° 1 - cas de MAS compliquée avec une pneumonie, anémie et un retard de croissance

1.1 Interrogatoire et examen

Comme mentionné dans la section précédente, l'un des premiers aspects de l'approche PCIME est d'évaluer les signes de danger de l'enfant. Parmi les 145 AS ayant passé la vignette no.1, trois (2%) ont posé toutes les trois questions relatives aux signes de danger. En revanche 77 agents de santé (53%) n'ont cherché aucun des trois signes de danger.

L'autre priorité de la PCIME implique des questions à la recherche des «principaux symptômes» (WHO 2014: 2-5). Au total, trois agents de santé (2%) ont posé toutes les cinq questions relatives aux « principaux symptômes » ; cinq agents de santé (4%) n'ont cherché aucun de ces symptômes. La plupart des agents de santé ont posé deux à trois de ces questions.

D'un point de vue plus global, dans la grille d'observation des vignettes, sur un total de 28 questions relatives à l'anamnèse, il y a 19 questions autonomes (c'est à dire des questions qui ne se rapportent pas à la réponse de la question précédente, par exemple la durée de la diarrhée ne serait pas posée si la présence de la diarrhée n'avait pas déjà été confirmée). Aucun des agents de santé n'a posé toutes les 19 questions. Deux agents de santé (1%) n'ont posé aucune des 19 questions. La plupart des agents de santé ont posé entre deux et six questions. Le Tableau 32 présente les principaux résultats de la section interrogatoire de la première Vignette.

Tableau 32. Nombre d'agents de santé qui ont interrogé sur les signes de danger, les principaux symptômes et des questions autonomes - Vignette no. 1

**Questions relatives à…**	**Nb d'AS<br>n (%)<br>N=145**
Aux signes de danger - toutes les trois posées	**3 (2)**
capable de boire/avaler	9 (6)
vomissements	56 (39)
présence de convulsions	20 (14)
Aux cinq principaux symptômes - toutes les cinq posées	**3 (2)**
diarrhée	81 (56)
toux	112 (77)
difficultés respiratoires	26 (18)
fièvre	123 (85)
problèmes d'oreilles	19 (13)
Toutes les questions autonomes (19)	0 (0)
Nombre maximum posé: 13	1 (1)
Aucune des questions posées	2 (1)

Il y a 19 examens physiques sur la grille d'observation de la vignette. Aucun des agents de santé n'a déclaré qu'il effectuerait tous ces examens. Six agents de santé (5%) n'ont signalé effectuer aucun des examens physiques figurant dans la grille. La plupart des agents de santé ont déclaré qu'ils effectueraient entre un et cinq des examens listés.

Si nous retirons de la grille des examens les aspects qui sont spécifiques à la nutrition (poids, taille, interpretation de la courbe de croissance, PB, oedèmes, test de l'appétit et calcul du rapport P/T), encore aucun des agents de santé n'a déclaré qu'il effectuerait tous les examens restants (13) ; le nombre maximal d'examens déclarés à effectuer était de huit et seulement deux agents de santé (2%) ont déclaré qu'ils allaient les effectuer.

En outre, seulement 14 agents de santé (10%) ont indiqué qu'ils mesureraient tous les trois signes vitaux majeurs. Vingt-cinq agents de santé (18%) ont indiqué qu'ils mesureraient deux des signes vitaux, 80 agents de santé (56%) ont indiqué qu'ils en mesureraient un et 24 agents de santé (17%) n'ont pas mentionné de mesure de signes vitaux dans le cadre de leur examen. Le Tableau 33 résume cette situation.

Tableau 33. Fréquence et type d'examens physiques que les agents de santé ont indiqué vouloir effectuer - Vignette no.1

**Examen physique**	**Nb d'AS<br>n (%)<br>N=145**
Tous les examens de la vignette (19)	0 (0)
Nombre maximum réalisé: 12	4 (3)
Aucun examen effectué	6 (5)
**Signes vitaux - tous les trois ont été mesurés**	
température	116 (80)
pouls	16 (11)
rythme respiratoire	44 (31)
pas de signes vitaux pris	24 (17)

1.1.1 Pneumonie

Cent douze (77%) agents de santé ont interrogé sur la présence de toux. La réponse pour cette vignette clinique était oui. Cette réponse positive devrait susciter un certain nombre de questions exploratoires pour compléter l'histoire de la toux. Des 112 AS qui ont interrogé sur la toux, seulement cinq (5%) ont posé les quatre questions associées. Cinq (5%) de ces AS n'ont posé aucune des questions associées. Le Tableau 34donne plus de détails concernant les questions sur l'histoire de la toux.

Tableau 34. Questions complémentaires relatives à la toux

Questions cliniques	Nb d'AS (parmi ceux qui ont demandé si présence de toux) n (%) N = 112
**Questions exploratoires de l'histoire de la toux (4)**	
cas interrogés sur toutes les 4 questions	5 (4)
cas sans aucune question complémentaire posée	5 (4)
fièvre	99 (89)
durée de la toux	62 (55)
toux sèche ou productive	23 (21)
difficultés respiratoires associées	23 (21)

Sur les 145 AS au total, 26 (18%) ont interrogé sur les difficultés respiratoires (qu'ils aient ou non demandé à propos de la toux). Ceci est un signe de danger clé et devrait susciter d'autres questions et des examens physiques spécifiques lorsque la réponse est positive (comme c'est le cas dans cette vignette-ci). Par exemple, il est important de demander si l'enfant est capable de boire/avaler et s'il y a eu une alteration dans la conscience de l'enfant. Parmi les 26 AS, un seul a posé des questions sur la capacité de déglutition, et un autre a posé des questions sur le niveau de conscience ; aucun d'entre eux n'a posé les deux questions ensemble. Les examens physiques à effectuer quand des difficultés respiratoires sont rapportées sont de mesurer la fréquence respiratoire, le pouls, la température, et le tirage sous-costal. Aucun des 26 AS qui ont interrogé sur les difficultés respiratoires n'a déclaré qu'ils effectueraient tous les quatre examens. La plupart ont indiqué qu'ils en effectueraient un.

Les résultats des examens reportés à l'AS étaient que l'enfant avait une fièvre, avait un pouls normal mais était tachypnéique et avait un tirage sous-costal modéré. Ceci, combiné avec les réponses données aux questions de l'interrogatoire, devrait inciter l'AS à donner un diagnostic de pneumonie. Cinquante-neuf AS (41%) ont donné un diagnostic de pneumonie. Quarante-quatre AS (31%) ont donné un diagnostic d'une infection des voies respiratoires supérieures (IVRS), et l'un des AS qui a donné un diagnostic de pneumonie a également donné un diagnostic d'infection des voies respiratoires supérieures. Deux AS ont donné un diagnostic de tuberculose pulmonaire, trois AS ont dit « pneumopathie » comme diagnostic, deux ont rapporté « grippe », un AS a déclaré une « infection respiratoire » et quatre AS ont signalé une « infection non identifiée » (voir le Tableau 37).

1.1.2. Anémie

Sur les 145 AS, 61 (42%) ont dit qu'ils allaient chercher des signes d'anémie dans le cadre de l'examen physique. La réponse qu'ils ont ensuite obtenue était que les conjonctives, les paumes et les lits des ongles étaient pâles. Parmi ces 61 AS, 21 (35%), ont alors dit qu'ils allaient demander un test d'hémoglobine; le résultat donné était 9,1g/dl (l'anémie est définie par l'OMS comme un taux d'hémoglobine inférieur à 9,3g/dl ; WHO 2013). En outre, parmi les 84 AS qui n'ont pas déclaré vouloir chercher des signes d'anémie, deux ont déclaré demander un test d'hémoglobine.

Au total 34 AS (24%) ont donné un diagnostic correct d'anémie (voir le Tableau 37). Parmi ces 34, tous avaient indiqué qu'ils chercheraient des signes d'anémie dans le cadre de l'examen clinique, mais seulement 16 ont déclaré qu'ils demanderaient un test d'hémoglobine.

La réponse de la vignette relative aux difficultés respiratoires, combinée avec la fréquence respiratoire accrue à l'examen, devrait également susciter un examen physique pour rechercher des signes d'anémie. Sur les 26 AS qui ont interrogé sur les difficultés respiratoires (et en tant que telle ont reçu une réponse positive), 16 d'entre eux (62%) ont effectué un examen à la recherche des signes d'anémie. Sur les 44 AS qui ont dit qu'ils mesureraient la fréquence respiratoire (et en tant que tels ont reçu un résultat d'augmentation du rythme), 28 d'entre eux (64%) ont cherché des signes d'anémie.

1.1.3. Malnutrition

Il y avait sept questions de la section de l'interrogatoire liées spécifiquement à la nutrition. Aucun des 145 AS n'a posé toutes les sept questions. Le maximum posé a été de cinq questions, cela a été fait par un AS (1%). Trente-six AS (25%) n'ont posé aucune de ces questions. Le Tableau 35 présente les questions qui ont été posées.

Tableau 35. Questions spécifiques à la nutrition - Vignette no.1

Questions de nutrition	Nb d'AS<br>n (%)<br>N=145
Toutes les sept questions posées	0 (0)
Aucune des sept questions posée	36 (25)
encore allaité*	35 (24)
épisodes d'allaitement depuis ce matin/hier soir	5 (4)
appétit	96 (66)
autres aliments complémentaires habituellement donnés	15 (11)
âge d'introduction des aliments complémentaires	4 (3)
qu'est ce qu'il a mangé depuis hier (dernières 24h)	8 (6)
alimentation changée récemment	4 (3)

* Dans cette vignette l'enfant était âgé de moins de deux ans, donc c'était une question appropriée pour l'âge

Dans la liste des examens physiques, il y avait six examens ou tâches liées à la nutrition : mesurer le poids, la taille, le PB; évaluer les œdèmes; calculer le rapport P/T ; et analyser la courbe de croissance. Deux AS (2%) ont déclaré qu'ils allaient mener toutes ces tâches et 46 AS (32%) n'ont mentionné aucune de ces tâches. Le Tableau 36 montre plus en détail le nombre d'AS qui ont effectué quelle tâche relative à la nutrition.

Tableau 36. Examens et tâches spécifiques de nutrition - Vignette no.1

Examens / tâches de nutrition	Nb d'AS n (%) N=145
tous les six examens / tâches	2 (2)
aucun des examens / tâches	46 (32)
mesure du poids	89 (62)
mesure de la taille	57 (40)
mesure du PB	57 (40)
recherche d'oedèmes	44 (31)
calcul du rapport P/T	42 (29)
analyse de la courbe de croissance	10 (7)

Quand les AS mentionnaient qu'ils voudraient effectuer ces tâches, ils recevaient de l'administrateur de la vignette des mesures qui correspondaient à un rapport P/T avec Z-score exactement de -3 (en utilisant les valeurs de référence poids-pour-taille 2006 de l'OMS) et un PB de 115mm. Selon le protocole national burundais de traitement de malnutrition (MSPLS 2010), la MAS est diagnostiquée chez les enfants avec un P/T inférieur à -3 et un PB de moins de 115mm. L'enfant dans ce cas n'avait pas d'œdème, donc techniquement l'enfant pouvait être classé comme MAM, mais le but de l'exercice pour l'AS était de réaliser que ces mesures anthropométriques combinées avec le diagnostic de pneumonie devaient inciter un diagnostic de MAS compliquéé, qui menait à un traitement très différent de celui de la MAM. Seuls 10 AS (7%) ont signalé le diagnostic correct de MAS compliquée (voir le Tableau 37).

Une analyse plus approfondie des réponses de ces 10 AS (ayant correctement diagnostiqué la MAS compliquée) a révélé que sept d'entre eux ont aussi donné un diagnostic de pneumonie. Parmi les trois autres, un a donné un diagnostic supplémentaire d'une infection des voies respiratoires supérieures qui ne serait pas inclus comme une complication conduisant au diagnostic supplémentaire de MAS compliquée (selon le protocole du Burundi) et aussi aurait été un diagnostic incorrect en soi à la lumière des résultats de l'interrogatoire et de l'examen. Un autre a donné un diagnostic supplémentaire d'anémie, mais n'a pas demandé un taux d'hémoglobine qui aurait montré certes l'anémie, mais une anémie non sévère; en effet, ce taux d'hémoglobine (9,1g/dl) ne conduit pas au diagnostic de MAS compliquée ; cela suggère qu'il se fiait sur les seules observations cliniques qui ne sont pas fiables pour évaluer la gravité de l'anémie. Le dernier AS n'a pas donné d'autres diagnostics en dehors de MAS compliqué.

Vingt-sept AS (19%) ont donné un diagnostic de MAM. Parmi ces 27 AS, 12 ont également signalé un diagnostic de pneumonie qui aurait du changer le diagnostic de MAM en MAS compliquée. Quatorze AS (10%) ont déclaré un diagnostic de MAS non compliquée; six d'entre eux ont également signalé un diagnostic de pneumonie, qui aurait à nouveau du changer le diagnostic en MAS compliquée.

Seuls 10 AS (7%) ont mentionné qu'ils voudraient analyser/ remplir/ discuter de la courbe de croissance. Ceux-ci ont reçu l'information que le poids était stagnant depuis la dernière visite d'il y a trois mois. Malgré cela, aucun des 145 AS au total n'a rapporté un diagnostic correct de retard de croissance.

1.2 Examens de Laboratoire

Cent seize AS (80%) ont déclaré qu'ils allaient mesurer la température du patient. Ceux-ci ont reçu la réponse que la température était de 38,6°C. Parmi ces 116 AS, 105 (91%), ont alors demandé un test de paludisme, pour lequel ils ont été informés qu'il était négatif. Il y avait au total 124 tests de paludisme demandés par les 145 AS, 19 d'entre eux (15%) ont été demandés par les AS qui n'avaient pas chercher au préalable à mesurer la température du patient et 17 (14%) ont été demandés par les AS qui n'avaient pas demandé à l'accompagnant si l'enfant avait de la fièvre. Parmi les 116 AS qui voulaient mesurer la température, cinq (5%) ont déclaré qu'ils allaient effectuer un examen des urines. Il n'y avait pas de tests d'urine demandés par les AS qui n'avaient pas déclaré qu'ils mesureraient la température. Voir la section « anémie » ci-dessous en rapport avec les tests d'hémoglobine

1.3 Diagnostics

Le Tableau 37 présente la fréquence des diagnostics déclarés par les AS pour la vignette no. 1. Les diagnostics en gras sont les diagnostics corrects pour cette vignette. Les AS pouvaient donner autant de diagnostics qu'ils estimaient appropriés. Il y avait aussi une option de « autre » avec un espace pour le texte libre ainsi l'enquêteur pouvait signaler les diagnostics qui ne sont pas dans la liste pré-établie.

Tableau 37. Diagnostics effectués par les AS - Vignette no.1.

Diagnostic	Nb d'AS qui ont rapporté le diagnostic n (%) N = 145	Diagnostic	Nb d'AS qui ont rapporté le diagnostic n (%) N = 145
Malaria sévère	2 (2)	**Retard de croissance**	0 (0)
Malaria non compliquée	3 (2)	otite moyenne aigüe	0 (0)
**Anémie**	**34 (24)**	gastro-entérite	0 (0)
Méningite	0 (0)	infection urinaire	0 (0)
**MAS compliquée**	**10 (7)**	IVRS	44 (31)
**Pneumonie**	**59 (41)**	(autre) TB pulmonaire	2 (2)
MAM	27 (19)	(autre) pneumopathie	3 (2)
MAS non compliqué	14 (10)	(autre) grippe	2 (2)
Déshydratation	0 (0)	(autre) infection respiratoire	2 (2)
parasitose intestinale	4 (3)	(autre) infection non identifiée	4 (3)
Diarrhée	0 (0)	(autre) candidose orale	2 (2)
Anorexie	0 (0)	je ne sais pas	2 (2)

MAS: Malnutrition Aigüe Sévère. MAM: Malnutrition Aigüe Modérée. IVRS: Infections des Voies Respiratoires Supérieures.

1.4 Traitement

1.4.1 Pneumonie

L'OMS et les directives PCIME recommandent de traiter la pneumonie avec un antibiotique et plus particulièrement l'amoxicilline en première ligne. Cent dix-sept AS (81%) ont prescrit un antibiotique. Cependant, il n'y a que 59 AS qui ont donné le bon diagnostic de la pneumonie ce qui signifie que les 58 restant ont prescrit un antibiotique à tort car il n'y avait pas d'autre indication de le faire dans cette vignette. Les antibiotiques font partie du traitement systématique pour les cas de MAS ; ici il s'agissait d'un cas de MAM qui devait cependant être considérée comme MAS (compliquée), en raison de la présence d'une pneumonie : c'est donc la pneumonie qui devait conduire l'AS à prescrire les antibiotiques et non la présence de malnutrition aigüe. Sur les 59 AS qui ont diagnostiqué une pneumonie, la majorité – 53 AS (90%) – ont prescrit un antibiotique (voir le Tableau 39). Soixante et un pour cent des AS qui ont diagnostiqué une pneumonie ont prescrit correctement l'amoxicilline. Les autres antibiotiques les plus fréquemment cités après l'amoxicilline étaient des choix incorrects ; certains sont présents dans les précédentes directives et certains sont en effet corrects pour le

traitement de première intention d'une pneumonie sévère, mais non d'une pneumonie (voir le Tableau 40). La majorité des AS (86%) ont également prescrit un antipyrétique, ce qui était approprié puisque l'enfant avait de la fièvre (voir le Tableau 39).

1.4.2. Anémie

Vingt-huit AS (20%), sur un total de 145, ont prescript le fer/acide folique (voir le Tableau 39). Parmi ces 28 AS, 19 (68%) avaient également donné le diagnostic correct d'anémie.

1.4.3. Malnutrition

La réaction correcte face à ce cas de MAS compliquée est de référer le patient est le service de stabilisation (SST), qui est l'unité de traitement des cas de MAS compliquées en milieu hospitalier. En effet, cet enfant est tachypnéique et montre d'autres signes de détresse respiratoire, combinés avec un rapport P/T et un PB juste à la limite de la MAS, ce qui signifie qu'il doit être traité comme un cas de MAS compliquée et a besoin de stabilisation et de surveillance étroite. Seuls huit AS (6%) ont déclaré ce plan de gestion correct et un seul de ces huit a indiqué qu'il expliquerait aussi à la mère qu'elle devait aller au SST et ce qui s'y passerait. Vingt-huit AS (20%) ont donné les conseils de nutrition à l'accompagnant (voir le Tableau 38).

1.4.4. Communication/conseils

L'aspect communication/conseil de la consultation a été systématiquement négligé avec plus de trois quarts des AS qui n'ont donné aucun des conseils ou explications proposés dans le questionnaire (voir le tableau 34).

Tableau 38. Conseils et explications donnés à l'accompagant - Vignette no.1

**Conseils ou explications**	**Nb d'AS n (%) N=145**
**conseil de nutrition**	28 (20)
conseils sur l'hygiène individuelle	12 (9)
**explications sur l'utilisation des médicaments**	35 (24)
**explications sur les signes de danger**	18 (13)
**explication sur la référence au SST et ce qui s'y passera**	4 (3)
**discuter avec la maman du plan de traitement et demander son avis**	14 (10)

SST: Service de Stabilisation

Les Tableau 39 et Tableau 42 montrent plus de détails sur les options de traitement signalés. Comme indiqué ci-dessus, les traitements en gras sont les traitements corrects en fonction de la PCIME, l'OMS et/ou les directives nationales burundaises en cas de malnutrition.

Tableau 39. Catégories de médicaments prescrits par les AS - Vignette no.1

Médicaments ou traitements	Nb d'AS n (%) N=145
anti-malaria	5 (4)
**antibiotiques**	**117 (81)**
**antipyrétiques**	**124 (86)**
**fer/acide folique**	**28 (20)**
SRO	5 (4)
anti-parasitaires	23 (16)
pas de traitement	3 (2)

SRO: Sérum de Réhydratation Orale

Tableau 40. Réponses à la question en rapport avec les antibiotiques - Vignette no.1

Réponses	Nombre d'AS n (%) N = 145
**Amoxicilline**	**72 (65)**
Cotrimoxazole	23 (21)
Ampicilline	2 (2)
Ampicilline et Gentamicine	4 (4)
Chroramphénicol	1 (1)
Erythromycine	1 (1)
Cloxacilline	1 (1)
**antibiotique non spécifié dans la prescription**	7 (5)
pas d'antibiotique prescrit	34 (24)

Tableau 41. Réponses aux questions en rapport avec les antibiotiques parmi les AS qui ont signalé le diagnostic de pneumonie - Vignette no.1

Réponses	Nb d'AS n (%) N=59
**Amoxicilline**	**36 (61)**
Cotrimoxazole	6 (10)
Ampicilline	1 (2)
Ampicilline et Gentamycine	3 (5)
Chloramphénicol	1 (2)
Erythromycine	1 (2)
Cloxacilline	1 (2)
**Antibiotique non spécifié dans la prescription**	3 (5)
Pas d'antibiotique prescrit	6 (10)

Tableau 42. Plan de traitement - Vignette no.1

Plan de traitement	Nb d'AS<br>n (%)<br>N=145
Référer à un SSN (pour les cas de MAM)	24 (17)
Référer à un STA (pour les cas de MAS non compliquée)	21 (15)
**Référer à un SST (pour les cas de MAS compliquée)**	**8 (6)**
Renvoyer l'enfant à la maison et donner une date de visite de suivi au centre de santé	14 (10)
**Mettre à jour/completer les vaccinations de l'enfant**	**4 (3)**
garder l'enfant en observation au centre de santé	2 (2)

SSN: Service de Supplémentation Nutritionnelle, STA: Service Thérapeutique Ambulatoire, SST: Service de Stabilisation

2. Vignette n° 2 - Un cas de MAS non compliquée avec anémie et un retard de croissance

2.1 Interrogatoire et Examen médical

En ce qui concerne les signes de danger, deux AS (2%) ont posé les trois questions relatives. Quatre-vingt-six AS (59%) n'ont cherché aucun des trois signes de danger.

Pour les « principaux symptômes », aucun des AS n'a posé toutes les cinq de ces questions et 42 AS (29%) n'ont cherché aucun de ces symptômes. La plupart des AS ont posé entre deux et trois de ces questions.

Sur les 19 questions autonomes, aucun des AS n'a posé toutes les 19 questions. Cinq AS (4%) n'ont posé aucune de ces 19 questions. La plupart des AS ont posé entre deux et sept questions. Le Tableau 43 présente les principaux résultats de la section interrogatoire du questionnaire pour la Vignette no.2.

Tableau 43. Nombre d’AS qui ont cherché les signes de danger, les principaux symptoms et posé les questions autonomes

Questions relatives à…	Nb d’AS<br>n (%)<br>N=145
Aux signes de danger - toutes les trois posées	2 (2)
capable de boire/avaler	25 (17)
vomissements	45 (31)
présence de convulsions	8 (6)
Aux cinq principaux symptômes - toutes les cinq posées	0 (0)
diarrhée	82 (57)
toux	76 (53)
difficultés respiratoires	7 (5)
fièvre	80 (55)
problèmes d’oreilles	9 (6)
Toutes les questions autonomes (19)	0 (0)
Nombre maximum posé: 11	3 (2)
Aucune des questions posées	5 (4)

En ce qui concerne l'examen physique, aucun des AS n’a déclaré qu'il effectuerait tous les examens (19) inclus dans le questionnaire. Sept AS (5%) n’ont aucun des examens physiques figurant dans le questionnaire. La plupart des AS ont déclaré qu'ils effectueraient entre une et huit des examens énumérés.

En retirant de la grille d’observation les aspects qui sont les plus liés à la nutrition/malnutrition, toujours aucun des AS n’a déclaré qu'il effectuerait tous les examens restants (13) ; le nombre maximal d'examens (non liés à la nutrition) déclarés à effectuer a été de 11 ; un seul AS a atteint ce score.

En outre, seulement 10 AS (7%) ont déclaré qu'ils mesureraient les trois signes vitaux majeurs. Onze AS (8%) ont déclaré qu'ils mesureraient deux des signes vitaux, 52 AS (36%) ont indiqué qu'ils mesureraient un et 72 AS (50%) n’ont pas mentionné de mesure des signes vitaux du tout dans le cadre de leur examen. Le Tableau 44 donne plus de détails sur les examens physiques.

Tableau 44. Fréquence et type d'examens physiques que les AS effectueraient – Vignette no.2

Examen physique	Nb d'AS<br>n (%)<br>N=145
Tous les examens de la vignette (19)	0 (0)
Nombre maximum réalisé: 15	1 (1)
Aucun examen effectué	7 (5)
**Signes vitaux - tous les trois ont été mesurés**	10 (7)
température	69 (48)
pouls	14 (10)
rythme respiratoire	21 (15)
pas de signes vitaux pris	72 (50)

2.1.1. Diarrhée/Gastro-entérite

Au total, 82 AS (57%) ont déclaré qu'ils demandaient la présence de diarrhée. La réponse était positive. Normalement, suite à cette constatation, un certain nombre de questions doivent alors être posées afin de retracer l'histoire de la diarrhée. Sur les 82 AS qui ont demandé sur la présence de diarrhée suite, aucun d'entre eux n'a ensuite posé l'ensemble des six questions complémentaires. La plupart des AS ont posé entre une et trois questions, et quatre AS (5%) n'ont posé aucune de ces questions.

De même, il existe quatre principaux examens physiques pour la recherche d'une déshydratation. Parmi les 82 AS qui ont interrogé sur la présence de diarrhée (ont reçu une réponse positive), cinq d'entre eux (6%) ont indiqué qu'ils effectueraient les quatre examens complémentaires requis, tandis que 47 AS (58%) n'ont mentionné aucun de ces examens. Le Tableau 45 donne plus d'informations sur les questions et examens associés à la diarrhée.

Tableau 45. Questions et examens physiques effectués après déclaration de la presence de diarrhée – Vignette no.2

Questions et examens complémentaires	AS ayant demandé la présence de diarrhée n (%) N = 82
**Questions explorant l'histoire de la diarrhée (6)**	
cas interrogés sur toutes les six questions	0 (0)
cas sans aucune question complémentaire posée	4 (5)
durée de la diarrhée	40 (49)
sang dans les selles	14 (17)
crampes abdominales	12 (15)
vomissements	39 (48)
alimentation changée récemment	3 (4)
fièvre	63 (77)
**Examens exploratoires de la déshydratation (4)**	
cas avec tous les 4 examens	5 (6)
cas sans aucun examen	47 (58)
pouls	10 (12)
signe de déshydratation	18 (22)
pli cutané s'effaçant lentement	28 (34)
yeux enfoncés récemment	24 (30)

2.1.2. Anémie

Sur les 145 AS au total, 51 (35%) ont dit qu'ils allaient chercher des signes d'anémie. La réponse qu'ils ont ensuite obtenu était que les conjonctives, les paumes et les lits des ongles étaient pâles, suggérant une anémie. Parmi ces 51 AS, 22 (43%) ont alors dit qu'ils demanderaient un test d'hémoglobine ; le résultat donné était 8,3g/dl. L'anémie est définie par l'OMS comme un taux d'hémoglobine inférieur à 9,3g/dl (WHO 2013a). En outre, parmi les 94 AS qui n'ont pas déclaré qu'ils allaient chercher des signes d'anémie, deux ont déclaré qu'ils allaient demander un test d'hémoglobine.

Vingt-huit AS (20%) ont posé un diagnostic correct d'anémie (voir Tableau 48). Sur ces 28 AS, 25 (90%) avaient indiqué qu'ils chercheraient des signes d'anémie dans le cadre de l'examen clinique, mais seulement 11 (40%) ont déclaré qu'ils allaient demander un test d'hémoglobine.

2.1.3. Malnutrition

Un des 145 AS a posé toutes les sept questions de nutrition, 20 AS (14%) n'ont posé aucune de ces questions. La plupart des AS ont posé entre une et trois de ces questions. Le Tableau 46 montre plus en détail les questions qui ont été posées.

Tableau 46. Questions spécifiques à la nutrition - Vignette no.2

Questions de nutrition	Nb d'AS n (%) N=145
Toutes les sept questions posées	1 (1)
Aucune des sept questions posée	20 (14)
encore allaité*	77 (53)
épisodes d'allaitement depuis ce matin/hier soir	25 (17)
appétit	86 (60)
autres aliments complémentaires habituellement donnés	43 (30)
âge d'introduction des aliments complémentaires	23 (16)
qu'est ce qu'il a mangé depuis hier (dernières 24h)	46 (32)
alimentation changée récemment	7 (5)

* Dans cette vignette l'enfant était âgé de moins de deux ans, donc c'était une question appropriée pour l'âge

Quatre AS (3%) ont déclaré qu'ils allaient procéder à tous les six examens physiques et tâches liées à nutrition, tandis que 19 AS (13%) n'ont mentionné aucune de ces tâches. Le Tableau 47 montre plus en détail les comportements des AS par rapport aux examens et tâches spécifiques à la nutrition.

Tableau 47. Examens et tâches spécifiques à la nutrition - Vignette no.2

Examens / tâches de nutrition	Nb d'AS n (%) N=145
tous les six examens / tâches	4 (3)
aucun des examens / tâches	19 (13)
mesure du poids	116 (80)
mesure de la taille	86 (60)
mesure du PB	73 (51)
recherche d'oedèmes	48 (33)
calcul du rapport P/T	52 (36)
analyse de la courbe de croissance	12 (9)

Si les AS ont mentionné qu'ils effectueraient ces tâches, ils ont reçu des mesures qui correspondaient à un rapport P/T entre -3 et -2 (MAM), un PB de 114mm, qu'il y avait des œdèmes bilatéraux au niveau des pieds seulement et que la courbe de croissance n'était pas disponible (c'est-à-dire que l'accompagnant n'avait pas apporté son carnet de santé). Le diagnostic devait donc être une MAS non compliquée: par le PB et /ou le z-score de MAM, combiné à la présence d'œdèmes. Il n'y avait pas de complications médicales actuelles qui justifiaient un diagnostic de MAS compliquée. Vingt AS (14%) ont donné un diagnostic correct de MAS non compliquée. Sur ces 20 AS pourtant, seulement neuf (45%) avaient mentionné qu'ils mesureraient le PB, évalueraient les œdèmes et calculeraient le rapport P/T.

2.2 Examens de Laboratoire

Soixante-neuf AS (48%) ont déclaré qu'ils allaient mesurer la température du patient : la réponse était que la température était 37,6°C. Parmi ces 69 AS, 39 (57%), ont ensuite demandé un test de paludisme (pour lequel ils ont été informés qu'il était négatif). Il y avait 53 tests de paludisme demandés parmi tous les 145 AS, 14 d'entre eux (26%) ont été demandés par les AS qui n'avaient pas déclaré qu'ils allaient d'abord mesurer la température du patient et 12 (23%) par les AS qui n'avaient pas demandé si l'enfant avait de la fièvre.

Parmi les 69 AS qui auraient mesuré la température, trois AS (4.5%) ont déclaré qu'ils effectueraient un examen d'urines. Voir la section « anémie » ci-dessus concernant les tests d'hémoglobine.

2.3 Diagnostics

Le Tableau 48 montre la fréquence des diagnostics déclarés par les AS pour la Vignette n°2. Les diagnostics en gras sont les diagnostics corrects pour cette vignette.

Tableau 48. Diagnostics effectués par les AS - Vignette no.2

Diagnostic	Nb d'AS qui ont rapporté le diagnostic n (%) N = 145	Diagnostic	Nb d'AS qui ont rapporté le diagnostic n (%) N = 145
Malaria sévère	1 (1)	Retard de croissance	4 (3)
Malaria non compliquée	0 (0)	Otite moyenne aigüe	0 (0)
**Anémie**	**28 (21)**	**Gastro-entérite**	**11 (8)**
Méningite	0 (0)	Infection urinaire	0 (0)
MAS compliquée	28 (20)	IVRS	7 (5)
Pneumonie	3 (2)	(autre) toux	1 (1)
MAM	36 (25)	(Autre) froid	1 (1)
**MAS non compliquée**	**20 (14)**	(Autre) intoxication alimentaire	1 (1)
Déshydratation	1 (1)	(Autre) enfant non malade	5 (4)
Infection parasitaire	18 (13)	(Autre) angine	1 (1)
**Diarrhée**	**12 (9)**	Ne sait pas	13 (9)
Anorexie	0 (0)		

MAS: Malnutrition Aigüe Sévère. MAM: Malnutrition Aigüe Modérée. IVRS: Infection des Voies Respiratoires Supérieures

2.4 Traitement

2.4.1. Diarrhée/Gastro-entérite

Le patient dans cette vignette ne présente pas de signes de déshydratation et les seules pertes sont de la diarrhée, il n'y a pas de vomissements. Ainsi, le traitement correct, considérant la MAS, serait soit

l'administration d'eau après chaque selle, ou le ReSoMal qui est une solution de réhydratation spécialement conçue pour les enfants souffrant de malnutrition sévère (moins de sodium, plus de potassium que dans le SRO) (MSPLS 2010; WHO 2013b). Malgré cela, 40 AS (28%) ont incorrectement prescrit du SRO (voir le Tableau 50).

2.4.2. Anémie

Trente-et-un (22%) AS sur le total de 145 ont prescrit du fer/acide folique. Parmi ces 31 AS, 14 (45%) avaient également donné un diagnostic correct d'anémie.

2.4.3 Malnutrition

Ce cas présentait une « MAS non compliquée ». La destination correcte de référence était donc le STA. Quarante AS (28%) ont fait ce choix. Vingt et un AS (15%) ont déclaré qu'ils référeraient l'enfant au SSN (service de prise en charge pour les cas de MAM) et 20 AS ont déclaré qu'ils référeraient l'enfant au SST (service d'hospitalisation pour MAS compliquée) (voir le Tableau 53).

2.4.4. Prescription d'antibiotiques

Quarante-neuf AS (34%) ont prescrit un ou plusieurs antibiotiques (voir le Tableau 50). L'antibiotique le plus couramment prescrit était l'amoxicilline avec 27 AS l'ayant prescrit. La majorité des AS (22) ne donnent pas une indication de la raison pour laquelle ils ont prescrit un antibiotique. Six AS ont déclaré que l'indication était le traitement systématique de MAS (voir le Tableau 51).

2.4.5. Communication/conseils

De même que pour la vignette n°1, l'aspect de communication et conseils lors de la consultation a été largement négligé, à l'exception des conseils diététiques qui ont été donnés plus fréquemment que dans la vignette n°1 : 60 AS (42%) ont mentionné qu'ils donneraient ce type de conseils à l'accompagnant. Les AS pouvaient mentionner plus d'une catégorie de conseils (Tableau 49).

Tableau 49. Conseils et explications données à l'accompagnant - Vignette no.2

conseils/explications	Nombre d'AS (%) n = 145
conseils nutrition	60 (42)
conseils sur l'hygiène individuelle	20 (14)
explication de l'utilisation des médicaments	28 (20)
explications des signes de danger	7 (5)
explications en rapport avec la reference au SST et ce qui va s'y passer	2 (2)
Discuter avec la maman du plan de traitement et lui demander son avis	13 (9)

SST: Service de Stabilisation

Les Tableau 50 à Tableau 53 présentent les options de traitement signalés. Les traitements en gras sont les traitements corrects en fonction de la PCIME, l'OMS et/ou des directives nationales burundaises en cas de malnutrition.

Tableau 50. Catégories de médicaments prescrits par les AS - Vignette no.2

Médicaments/traitement	Nombre d'AS (%) n = 145
Anti-malaria	3 (2)
**Antibiotiques***	49 (34)
Antipyrétiques	28 (20)
**Fer/Acide Folique**	31 (22)
SRO	40 (28)
Anti-parasitaires	50 (35)
Pas de traitement	6 (4)

SRO: Sérum de Réhydratation Orale; * correct uniquement si il s'agit d'amoxicilline pour le traitement systématique de la MAS.

Tableau 51. Réponses sur la question en rapport avec les antibiotiques - Vignette no.2

Réponse	Nombre d'AS qui ont prescrit un antibiotique (%) n = 49
**Amoxicilline**	27 (55)
Cotrimoxazole	13 (27)
Ampicilline	1 (2)
Pénicilline et Amoxicilline	1 (2)
Chloramphénicol	2 (4)
Erythromycine	1 (2)
Non spécifié	1 (2)

Tableau 52. Indication donnée sur la prescription des antibiotiques* - Vignette no.2

Indication	Nombre d'AS qui ont prescrit un antibiotique (%) n = 49
Bronchite	2 (4)
Diarrhée	6 (12)
Toux	5 (10)
enfant très faible	1 (2)
'Infection Respiratoire Aigüe	1 (2)
Infection parasitaire	1 (2)
**Traitement systématique des enfants malnutris sévères**	**6 (12)**
Pas un antibiotique(paracétamol)	1 (2)
pas d'indication donnée	22 (45)

***** Plus d'une indication peut avoir été donnée par l'AS

Tableau 53. Plans de prise en charge - Vignette no.2

Plan de traitement	Nombre d'AS (%) n = 45
Référer à un SSN (pour les cas de MAM)	21 (15)
**Référer à un STA (pour les cas de MAS non compliquée)**	**40 (28)**
Référer à un SST (pour les cas de MAS compliquée)	20 (14)
Renvoyer l'enfant à la maison avec une date de visite de suivi au centre de santé	5 (4)
**Mettre à jour/completer les vaccinations de l'enfant**	**5 (4)**
garder l'enfant en observation au centre de santé	2 (2)

SSN: Service de Supplémentation Nutritionnelle, STA: Service Thérapeutique Ambulatoire, SST: Service de Stabilisation

3. Vignette n°3 - Un cas de MAM avec paludisme non compliqué et un retard de croissance

3.1 Interrogatoire et examen médical

En ce qui concerne les signes de danger, cinq AS (4%) ont posé les trois questions relatives. Soixante-quatre AS (44%) n'ont cherché aucun des trois signes de danger.

Pour les « principaux symptômes », aucun des AS n'a posé les cinq de ces questions, et 13 AS (9%) n'ont posé aucune de ces questions relatives aux principaux symptômes. La plupart des AS ont posé entre une et trois de ces questions.

Sur les 19 questions autonomes, aucun des AS n'a posé toutes les 19 questions. Onze AS (8%) n'ont posé aucune de ces questions. La plupart des AS ont posé entre trois et quatre questions. Le Tableau 54 résume les principaux résultats de la section de l'interrogatoire du questionnaire pour la vignette no.3.

Tableau 54. Nombre d’AS qui ont cherché les signes de danger, les principaux symptômes et posé les questions autonomes-vignette no.3

Questions relatives à…	Nb d’AS<br>n (%)<br>N=145
Aux signes de danger - toutes les trois posées	5 (4)
capable de boire/avaler	17 (12)
vomissements	63 (44)
présence de convulsions	28 (20)
Aux cinq principaux symptômes - toutes les cinq posées	0 (0)
diarrhée	82 (57)
toux	99 (69)
difficultés respiratoires	11 (8)
fièvre	84 (58)
problèmes d’oreilles	31 (22)
	(N = 144)
Toutes les questions autonomes (19)	0 (0)
Nombre maximum posé: 9	2 (2)
Aucune des questions posées	11 (8)

En ce qui concerne l'examen physique, aucun des AS n’a déclaré qu'il effectuerait tous les examens (19) inclus dans le questionnaire. Sept AS (5%) n’ont signalé aucun des examens physiques figurant dans le questionnaire. La plupart des AS ont déclaré qu'ils effectueraient entre un et six des examens énumérés.

En enlevant les aspects qui sont liés à la nutrition / malnutrition, encore aucun des AS n’a déclaré qu'il effectuerait tous les examens/tâches restants (13), le nombre maximal d'examens déclarés à effectuer était neuf (seulement deux AS).

Onze AS (8%) ont indiqué qu'ils mesureraient les trois signes vitaux majeurs. Sept AS (5%) ont déclaré qu'ils allaient mesurer deux des signes vitaux, 95 AS (66%) ont indiqué qu'ils mesureraient un et 32 AS (22%) n’ont pas mentionné de mesure de signes vitaux du tout dans le cadre de leur examen. Le Tableau 55 donne plus de détails sur les examens physiques réalisés.

Tableau 55. Fréquence et type d'examen physique que les AS ont signalé qu'ils effectueraient - Vignette no.3

Examen physique	Nb d'AS n (%) N=145
Tous les examens de la vignette (19)	0 (0)
Nombre maximum réalisé: 14	1 (1)
Aucun examen effectué	7 (5)
**Signes vitaux - tous les trois ont été mesurés**	11 (9)
température	112 (77)
pouls	12 (9)
rythme respiratoire	18 (13)
pas de signes vitaux pris	32 (22)

3.1.1. Malnutrition

Aucun des 145 AS n'a posé les sept questions de nutrition, 55 AS (38%) n'ont posé aucune de ces questions. La plupart des AS ont posé entre une et deux de ces questions. Le Tableau 56 montre plus en détail les questions qui ont été posées.

Tableau 56. Questions spécifiques de nutrition - Vignette no.3

Questions de nutrition	Nb d'AS n (%) N=145
Toutes les sept questions posées	0 (0)
Aucune des sept questions posée	55 (38)
encore allaité*	44 (31)
épisodes d'allaitement depuis ce matin/hier soir	2 (1)
appétit	68 (47)
autres aliments complémentaires habituellement donnés	10 (7)
âge d'introduction des aliments complémentaires	1 (1)
qu'est ce qu'il a mangé depuis hier (dernières 24h)	5 (4)
alimentation changée récemment	10 (7)

* Dans cette vignette l'enfant était âgé de moins de deux ans, donc c'était une question appropriée pour l'âge

Un seul AS a rapporté qu'il effectuerait les six examens physiques et tâches liées à la nutrition ; 52 AS (36%) n'ont mentionné aucun de ces examens ou tâches. Le Tableau 57 présente plus en détail le nombre d'AS qui effectueraient quelles tâches de nutrition.

Tableau 57. Examens et tâches spécifiques de nutrition – Vignette no.3

Examens / tâches de nutrition	Nb d'AS n (%) N=145
tous les six examens / tâches	1 (1)
aucun des examens / tâches	52 (36)
mesure du poids	87 (60)
mesure de la taille	60 (42)
mesure du PB	53 (37)
recherche d'oedèmes	39 (27)
calcul du rapport P/T	44 (31)
analyse de la courbe de croissance	6 (4)

Si les AS ont mentionné qu'ils effectueraient ces tâches, ils ont reçu des mesures qui correspondaient à un P/T entre -3 et -2, un PB de 120 mm, qu'il n'y avait pas d'œdèmes bilatéraux et que la courbe de croissance montrait le même poids que trois mois auparavant.

3.2 Examens de Laboratoire

Cent douze AS (77%) ont indiqué qu'ils mesureraient la température du patient : ils ont été informés que la température était de 37,8°C. Parmi ces 112 AS, 109 (98%) ont ensuite demandé un test du paludisme (pour lequel ils ont été informés qu'il était positif). Il y avait 142 tests de paludisme demandés parmi tous les 145 AS, 33 d'entre eux (23%) ont été demandés par les AS qui n'avaient pas déclaré qu'ils allaient d'abord mesurer la température du patient et 59 (42%) par les AS qui n'avaient pas demandé si l'enfant avait de la fièvre. Parmi les 112 AS qui mesureraient la température, quatre AS (4%) ont déclaré qu'ils allaient effectuer un examen d'urine.

Soixante-sept AS (46%) ont déclaré qu'ils allaient chercher des signes d'anémie. La réponse qui leur a été donnée était que les conjonctives, les paumes et les lits des ongles étaient pâles. Parmi ces 67 AS, 30 (45%) ont ensuite demandé un test d'hémoglobine ; la réponse qui leur était alors donnée est 9,5g/dl. Il n'y a pas eu de test d'hémoglobine demandé par les AS qui n'avaient pas déclaré qu'ils chercheraient des signes d'anémie.

3.3 Diagnostics

Le Tableau 56 montre la fréquence des diagnostics déclarés par les AS pour vignette no.3. Les diagnostics en gras sont les diagnostics corrects pour cette vignette.

Tableau 58. Diagnostics et fréquence avec laquelle ils étaient donnés par les AS-vignette no.3

Diagnostic	Nb d'AS qui ont rapporté le diagnostic n (%) N = 145	Diagnostic	Nb d'AS qui ont rapporté le diagnostic n (%) N = 145
Malaria sévère	31 (22)	Anorexie	0 (0)
**Malaria non compliquée**	**110 (76)**	**Retard de croissance**	**1 (1)**
Anémie	29 (20)	otite moyenne aigüe	0 (0)
Méningite	0 (0)	gastro-entérite	1 (1)
MAS compliquée	8 (6)	infection urinaire	0 (0)
Pneumonie	0 (0)	IVRS	0 (0)
**MAM**	**38 (26)**	(autre) pb digestifs	1 (1)
MAS non compliquée	5 (4)	(autre) suspicion de fièvre typhoïde	1 (1)
Déshydratation	0 (0)	(autre) tachypnée	1 (1)
Infection parasitaire	6 (4)	(autre) probable syndrome infectieux	1 (1)
Diarrhée	1 (1)	Ne sait pas	2 (2)

MAS Malnutrition Aigüe Sévère.Malnutrition Aigüe Modérée. IVRS: Infection des voies Respiratoires Supérieurs

3.3.1. Malaria non compliquée

Au total, 110 AS (76%) ont donné le bon diagnostic da paludisme non compliqué et chacun de ces AS avaient fait un test de paludisme. Trente-et-un autre AS (22%) ont donné le diagnostic de paludisme grave même si il n'y avait pas de signes de gravité dans cette vignette clinique.

3.3.2. Malnutrition

Trente-huit AS (26%) ont donné le diagnostic correct de MAM (voir le Tableau 58). Cinq ont signalé un diagnostic de MAS non compliquée et huit de MAS compliquée. Un seul AS a donné un diagnostic correct de retard de croissance, en dépit des six AS ayant dit qu'ils allaient analyser la courbe de croissance.

3.4 Traitement

3.4.1 Malaria non compliquée

Cent trente-six AS (94%) ont prescrit un médicament antipaludique (voir Tableau 60). Parmi eux, 103 AS (78%) ont déclaré qu'ils prescriraient une combinaison à base d'artémisinine ; avec 95 AS précisant l'Artésunate-Amiodiaquine. Dix AS (7%) ont déclaré qu'ils prescriraient la quinine orale (ce qui est une thérapie de deuxième ligne si la combinaison à base d'artémisinine n'est pas disponible). Douze AS ont indiqué qu'ils donneraient un traitement intra-veineux (IV) : un ayant prescrit l'artésunate et 11 la quinine. Cent dix-huit (81%) AS ont prescrit un antipyrétique (voir le Tableau 60). Vingt-trois (18%) de ces 118 AS ont prescrit un antipyrétique sans demander la présence de la fièvre et/ou dire qu'ils mesureraient la température de l'enfant.

3.4.2. Malnutrition

Il s’agissait ici d’un cas de MAM, et le plan de traitement correct consistait à référer au SSN. Au total, 26 AS (18%) ont déclaré qu'ils référeraient l'enfant au SSN, 24 (93%) de ces 26 AS ayant également donné un diagnostic de MAM. Douze AS ont déclaré qu'ils référeraient l'enfant au STA, neuf de ces AS ayant pourtant donné à l'enfant un diagnostic de MAM et les trois autres ayant donné un diagnostic de MAS non compliquée. Onze AS ont envoyé l'enfant au SST, quatre d'entre eux ayant donné un diagnostic de MAM, six de MAS compliquée et un de MAS non compliquée (voir le Tableau 62).

Quarante-sept AS (33%) ont déclaré qu'ils donneraient des conseils de nutrition à l'accompagnant (voir le Tableau 59).

3.4.3. Communication/conseils

Le Tableau 59 montre que, comme dans les vignettes précédentes, l'aspect communication de la consultation a été négligé avec moins de la moitié des AS ayant donné des conseils ou posé des questions ou discuté avec l’accompagnant sur le plan de gestion de la maladie.

Tableau 59. Conseils et explications donnés à l’accompagnant

**Conseils ou explications**	**Nombre d’AS n (%) N = 145**
conseils de nutrition	47 (33)
conseisl sur l’hygiène individuelle	22 (15)
explications sur l’utilisation des médicaments	52 (36)
explications des signes de danger	15 (11)
explications en rapport avec la référence au SST et ce qui s’y passera	2 (2)
discuter avec la maman du plan de prise en charge et demander son avis	20 (14)

SST: Service de Stabilisation

3.4.3. Prescription de fer/acide folique

Trente-sept AS (26%) ont prescrit du fer/acide folique (voir le Tableau 60). Vingt de ces 37 (53%), ont donné l'anémie comme raison de leur prescription. Quinze (41%) de ces 37 avaient demandé un taux d'hémoglobine (la réponse donnée était alors 9,5g/dl) et 31 (85%) avaient dit qu'ils allaient chercher des signes d'anémie. Un des 37 AS qui a prescrit du fer/acide folique a déclaré qu'il était lié au traitement systématique de malnutrition.

Les Tableau 60 à Tableau 62 montrent plus de détails sur les options de traitement proposées. Les traitements en gras sont les traitements corrects en fonction de la PCIME, de l'OMS et/ou des directives nationales burundaises en cas de malnutrition.

Tableau 60. Catégories de médicaments prescrits par les AS - Vignette no.3

Médicaments/traitement	Nombre d'AS<br>n (%)<br>N = 145
**Anti-malaria**	**136 (94)**
Antibiotiques	8 (6)
**Antipyrétiques**	**118 (81)**
Fer/Acide Folique	37 (26)
SRO	9 (7)
Anti-parasitaires	21 (15)
Pas de traitement	0 (0)

SRO: Sérum de Réhydratation Orale

Tableau 61. Type d'anti-malaria prescript par les AS

Médicaments/Traitement	Nombre d'AS<br>n (%)<br>N = 145
**ACT**	**8 (6)**
**Artésunate-Amodiaquine**	**95 (71.5)**
Artésunate en IV	1 (1)
Quinine en IV	11 (9)
Quinine PO*	10 (8)
Non spécifié	11 (8)

IV: intra-veineuse; PO: per os; *correct si ACT non disponibles

Tableau 62. Destination des references/plan de prise en charge

Plan de prise en charge	Nombre d'AS<br>n (%)<br>N = 145
**Référer au SSN (pour les cas de MAM)**	**26 (18)**
Référer au STA (pour les cas de MAS non compliquée)	12 (9)
Référer au SST (pour les cas de MAS compliquée)	11 (8)
Renvoyer l'enfant à la maison avec une date de visite de suivi au centre de santé	6 (4)
**Mettre à jour/compléter les vaccinations de l'enfant**	**2 (2)**
Garder l'enfant en observation au centre de santé	2 (2)

SSN: Service de Supplémentation Nutritionnelle, STA: Service Thérapeutique Ambulatoire, SST: Service de Stabilisation

4. Formation professionnelle continue, supervision et rémuneration des AS

Comme annoncé dans la section méthodologique, dans le cadre des vignettes, nous avons également collecté des informations sur l'AS évalué. Les Tableau 63 et Tableau 64 ci-dessous présentent les formations que les AS ont reçu après avoir terminé leurs études officielles (i.e. formation professionnelle continue) de la part du Programme de Nutrition (PRONIANUT) ou de la Direction de la promotion de la

Santé Hygiène et Assainissement (DPSHA) du ministère de la Santé. Ces formations continues sont pertinentes pour le développement de leurs connaissances et compétences cliniques.

Le Tableau 65 présente quant à lui les principaux domaines de formation complémentaire nécessaire, selon les AS eux-mêmes pour améliorer leurs connaissances et leurs compétences cliniques.

Tableau 63. Formation professionnelle continue des AS (PRONIANUT)

Type de formation reçue (PRONIANUT)	Nombre d'AS<br>n (%)<br>N = 145
Suivi et promotion de la croissance	21 (15)
Dépistage et prise en charge de la malnutrition aiguë	60 (42)
Conseils nutritionnels et fonctionnement des FARN	16 (11)
Prise en charge de la MAS compliquée	33 (23)

FARN: 'Foyers d'Apprentissage et de Réhabilitation Nutritionnelle' .

Tableau 64. Formation professionnelle continue des AS (DPSHA)

Type de formation reçue (DPSHA)	Nombre d'AS<br>n (%)<br>N = 145
Compétences de communication en santé communautaire	6 (4)
Santé communautaire (y compris la PCIME)	2 (2)

Tableau 65. Domaines nécessitant de formation complémentaire selon les AS

Type de formation indispensable	Nombre d'AS<br>n (%)<br>N = 145
Dépistage et prise en charge de la malnutrition aiguë	98 (68)
Gestion des MAS compliquées	74 (51)
Suivi et promotion de la croissance	52 (36)
Conseils nutritionnels et fonctionnement des FARN	47 (33)
Compétences de communication en santé communautaire	34 (24)
Santé communautaire (y compris la PCIME)	33 (23)
Planification familiale et santé reproductive	11 (8)
Gestion des enfants vivant avec le VIH	10 (7)
Prise en Charge Intégrée des maladies de l'enfant (PCIME)	8 (6)
Soins obstétricaux, prénataux et post-nataux	8 (6)
Prévention et prise en charge de la transmission du VIH de la mère à l'enfant	3 (2)
Gestion des enfants tuberculeux	3 (2)
Gestion des enfants atteints de paludisme	2 (2)

En ce qui concerne la supervision des activités des AS, 57 AS (40%) ont déclaré qu'ils avaient reçu une ou plusieurs visites de supervision par une autorité en dehors du centre de santé sur le thème de la

malnutrition aucours des six derniers mois. Le Tableau 66 résume de qui étaient ces visites de supervision.

Tableau 66. Visites de supervision reçues par les AS au cours des six derniers mois

Autorité ayant effectué la visite de supervision	Nombre d'AS n (%) N = 145
Point focal nutrition du district	27 (19)
Superviseur du district	29 (20)
Médecin chef de district	3 (2)
Superviseur du BPS	3 (2)
Personnel du PAM	2 (2)
Superviseur du PEV	1 (1)

En ce qui concerne le salaire, 122 AS (93%) ont indiqué que dans les 12 derniers mois, ils avaient toujours reçu leur salaire à temps. De même, 129 AS (98%) ont déclaré qu'ils avaient reçu la totalité du montant de leur salaire pour le mois dernier.

4.2 Enquête au niveau des ménages

Les données ont été collectées auprès de 6,199 ménages et enfants de 6-23 mois entre le 8 décembre 2014 et le 13 janvier 2015 (ISTEEBU 2015a, 2015b).

Caractéristiques des ménages

Caractéristiques générales et socio-économiques

Le Tableau 67 présente les caractéristiques socio-économiques clé issues de l'enquête.

Tableau 67. Caractéristiques générales et socio-économiques des ménages

	Obs.	Moyenne	Ecart-type
Taille du ménage (nombre de personnes)	6199	5.64	0.04
Enfants de moins de cinq ans par ménage	6199	1.66	0.01
Chef de ménage sans activité	5756	1%	0.00
Activité du chef de ménage: agriculture, élevage, pêche	5756	82%	0.01
Chef de ménage ne sait ni lire ni écrire	6080	29%	0.01
Chef de ménage sait lire et écrire	6080	69%	0.01
Chef de ménage n'a pas été scolarisé	6015	67%	0.02
Chef de ménage a terminé l'école primaire	6015	28%	0.01
Chef de ménage a terminé l'école secondaire et/ou l'enseignement supérieur	6015	4%	0.01
Ménage possède des terres cultivables	6195	90%	0.01
Ménage possède un véhicule	6199	2%	0.00
Ménage possède une bicyclette	6199	24%	0.01
Ménage possède de l'électricité	6199	5%	0.01
Ménage possède un savon	6194	65%	0.01
Source d'eau: robinet	6199	3%	0.01
Source d'eau: fontaine	6199	40%	0.02
Source d'eau: puits ou source naturelle protégés	6199	37%	0.02
Distance par rapport à la source d'approvisionnement en eau: moins de 30 minutes	6190	78%	0.02
Distance par rapport à la source d'approvisionnement en eau: plus de 30 minutes mais moins d'une heure	6190	18%	0.01
Traitement de l'eau avant de la boire	6199	5%	0.00
Eau suffisante pour tous les besoins du ménage	6197	80%	0.01
Eau insuffisante pour les besoins du ménages	6197	17%	0.01
Combustible: bois de chauffe	6199	92%	0.01
Toilette privée pour le ménage	6198	95%	0.01
Toilette partagée avec autres ménages	5919	16%	0.01

Sécurité alimentaire

Les conditions liées à l'insécurité alimentaire des ménages

Les résultats de l'enquête de référence montrent qu'il y a eu des réponses positives pour les neuf questions de survenance des conditions d'insécurité alimentaire. La fréquence de survenue des conditions d'insécurité alimentaire est relativement élevée, car seules deux conditions reviennent dans moins de 50% des ménages, ces deux conditions étant celles témoignant d'une insécurité alimentaire grave. Plus de 70% des répondants ont expérimenté les cinq premières conditions d'insécurité alimentaire. Le Tableau 68 présente ces résultats.

Tableau 68. Conditions de sécurité alimentaire

Conditions	Obs.	Proportion	Ecart-type
Fréquence de ménages:			
1. ayant été préoccupé par le manque de nourriture	6196	79%	0.01
2. dont un membre n'a pas pu manger les aliments préférés par manque de ressources	6195	86%	0.01
3. dont un membre a mangé une variété plus limitée d'aliments par manque de ressources	6198	85%	0.01
4. dont un membre a mangé des aliments qu'il n'aime pas manger par manque de ressources	6194	84%	0.01
5. dont un membre a mangé un repas plus petit par manque de ressources	6195	70%	0.02
6. dont un membre a mangé moins de repas par jour par manque de ressources	6196	62%	0.02
7. ayant manqué totalement de nourriture par manque de ressources	6196	61%	0.02
8. dont un membre est allé au lit en ayant faim par manque de ressources	6197	43%	0.02
9. dont un membre a passé la journée sans manger par manque de ressources	6197	17%	0.01

La prévalence de l'insécurité alimentaire des ménages

Considérant ces réponses et la méthode d'échelle de l'accès déterminant l'insécurité alimentaire des ménages pour la mesure de l'accès alimentaire des ménages (cf. section 2, et plus particulièrement le Tableau 7, page 46), cette enquête révèle que 68% des répondants expérimentent une insécurité alimentaire grave, tandis que 19% des répondants expérimentent une insécurité alimentaire modérée (Tableau 69).

Tableau 69. Prévalence des différentes catégories de sécurité alimentaire

Catégories d'insécurité alimentaire	Obs.	Proportion	Ecart-type
Sécurité alimentaire	6189	8%	0.01
Insécurité alimentaire légère	6189	5%	0.00
Insécurité alimentaire modérée	6189	19%	0.01
Insécurité alimentaire grave	6189	68%	0.02

Caractéristiques des enfants et de leurs responsables

Caractéristiques générales

Les Tableau 70 et Tableau 71 présentent les principales caractéristiques des enfants et de leurs responsables.

Tableau 70. Caractéristiques générales des responsables des enfants

	Obs.	Moy.	Ecart-type
Le responsable de l'enfant est sa mère	6199	99%	0.00
Age de la mère	6092	28.36	0.12
Le responsable de l'enfant est le chef de ménage	6197	4%	0.00
**Scolarisation du responsable de l'enfant**			
Aucune	6199	73%	0.01
Niveau primaire	6199	23%	0.01
Niveau secondaire ou supérieur	6199	4%	0.01
Capable de lire et d'écrire	6199	51%	0.02
**Activité du responsable de l'enfant**			
Aucune	6135	2%	0.00
Agriculture	6135	95%	0.01

Tableau 71. Caractéristiques générales de l'enfant

	Obs.	Freq.	Ecart-type
**Age**			
6-11 mois	6199	35%	0.01
12-17 mois	6199	33%	0.01
18-23 mois	6199	31%	0.01
**Sexe**			
masculin	6199	49%	0.01

Etat de santé, statut nutritionnel et allaitement de l'enfant

Etat de santé de l'enfant

Plus de la moitié des enfants enquêtés (59%) ont été malades dans les deux semaines précédant l'entretien. La plainte la plus communément rencontrée étant la fièvre (55%) et la diarrhée et l'infection respiration étant survenues chacune chez un tiers des enfants. La majorité de ces enfants (81%) ont été amenés en consultation au centre de santé ; et 100% des répondants ont affirmé que leur enfant été connu au centre de santé (l'enfant avait déjà visité le centre de santé ou un autre un bon nombre de fois). Ce pourcentage important d'enfants ayant été amenés en consultation est impressionnant dans la mesure où 50% des répondants doivent faire plus d'une heure pour arriver à la formation sanitaire. Très peu d'enfants (0.6%) ont été rapportés comme étant suivis dans un service de supplémentation nutritionnelle (SSN) ou un service thérapeutique ambulatoire (STA). La majorité des enfants (79%) avaient un carnet de santé (Tableau 72).

Tableau 72. Etat de santé de l'enfant

	Obs.	Freq.	Ecart-type
**A été malade les deux dernières semaines**	6199	59%	0.01
A eu de la diarrhée	3668	35%	0.01
A eu de la fièvre	3668	55%	0.01
A eu une infection respiratoire	3668	33%	0.02
A consulté le centre de santé	3668	81%	0.01
**Est connu au centre de santé**	6199	100%	0.00
**Temps pour arriver au centre de santé: moins de 30 minutes**	5996	22%	0.02
**Temps pour arriver au centre de santé: plus d'une heure**	5996	50%	0.02
**Est suivi au SSN**	6199	0.6%	0.00
**Est suivi au STA**	6199	0.6%	0.00
**Possède un carnet de santé**	6199	79%	0.01

Opinions des responsables des enfants sur leur état nutritionnel

Au total, 36% (2,259) des mères ont rapporté ne pas être satisfaites de l'état nutritionnel de leur enfant. Parmi ces dernières, 88% de leurs enfants ne souffraient pas de malnutrition aigüe mais 65% plutôt de malnutrition chronique. Plus spécifiquement, 826 des 2259 mères (36%) ont exprimé le souhait que leur enfant ait une plus grande taille, ce qui est conforme à la prévalence relativement élevée du retard de croissance observé chez leurs enfants.

Au contraire, 63% (3,914) des mères ont rapporté être satisfaites de l'état nutritionnel de leur enfant. Parmi elles, 97% de leurs enfants étaient correctement nutris mais paradoxalement, 46% d'entre eux souffraient d'un retard de croissance.

Pratiques relatives à l'allaitement

La majorité des indicateurs relatifs à l'allaitement étaient satisfaisants par rapport aux recommandations nationales sur les pratiques de l'Alimentation du Nourrisson et du Jeune Enfant (ANJE, WHO 2010; cf. Tableau 73). Ceci peut être expliqué en partie, par l'importance culturelle que revêt l'allaitement au Burundi.

Les pratiques relatives à l'alimentation de complément

Approximativement, pour la moitié des enfants, toutes catégories d'âge confondues, la personne responsable a rapporté que l'enfant recevait le nombre minimum de groupes d'aliments recommandés (quatre) ; plus l'enfant est âgé, plus cet état de fait est marqué. De même, un peu moins de la moitié des enfants reçoivent globalement le nombre minimum de repas recommandés par jour et par rapport à leur âge, avec une meilleure fréquence chez les enfants les plus âgés quoique le chiffre reste en-deçà de ce qui serait considéré comme satisfaisant. Sans surprise, seul un quart des enfants a un score satisfaisant à l'indicateur combinant le minimum de fréquence des repas et le minimum de diversité alimentaire. La majorité des enfants ont reçu du fer ou de la vitamine A dans les derniers six mois. Le Tableau 74 donne plus de détails sur les pratiques de l'alimentation de complément.

Tableau 73. Indicateurs relatifs à l'allaitement

Indicateurs	Recommandations nationales	Freq.	Obs.
Enfants ayant été allaités		99.9%	6197
Allaitement initié dans l'heure après l'accouchement	85%	86.1%	6090
Allaitement initié dans les 24 heures suivants l'allaitement		98.0%	6090
Allaitement exclusive jusqu'à l'âge de six mois	80%	81.7%	6074
Les enfants qui continuent d'être allaité ou ont été allaité jusqu'à l'âge de 12mois (pour les enfants de 12-15mois)		97.7%	1364
% des enfants qui continuent d'être allaité jusqu'à l'âge de 20 mois (pour les enfants de 20-23 mois)	80%	85.0%	1238
Moyenne d'âge de sevrage (parmi les enfants déjà sevrés)		14.7 mois	427

Tableau 74. Indicateurs relatifs à l'alimentation de complément

Indicateurs	Freq.	Obs.
**Nombre minimum recommandé de groupes d'aliments (i.e. diversité alimentaire minimale) - min. 4 groupes**	49.3%	5542
6-11 mois	42.1%	1769
12-17 mois	50.4%	1944
18-23 mois	55.1%	1829
**Fréquence minimale de repas par jour**	43.8%	6164
6-11 mois	32.8%	2176
12-17 mois	47.6%	2067
18-23 mois	52.1%	1921
**Minimum alimentaire acceptable (i.e. fréquence et diversité satisfaisante)**	25.0%	6144
6-11 mois	17.7%	2173
12-17 mois	27.6%	2060
18-23 mois	30.5%	1911
**Alimentation mixte (allaitement plus alimentation de complément) (durant les 24 heures précédentes)**	83.1%	6165
6-11 mois	81.3%	2176
12-17 mois	88.9%	2067
18-23 mois	79.1%	1922
**Consommation d'aliments sucrés**	22.8%	5575
6-11 mois	27.6%	1774
12-17 mois	20.6%	1954
18-23 mois	20.8%	1847
**Supplémentation en Vitamine A ou fer dans les six derniers mois**	78.7%	6192
6-11 mois	79.1%	2178
12-17 mois	80.5%	2074
18-23 mois	76.5%	1940

Figure 6. Age d'introduction d'aliments de compléments (parmi les enfants âgés de 12-23 mois)

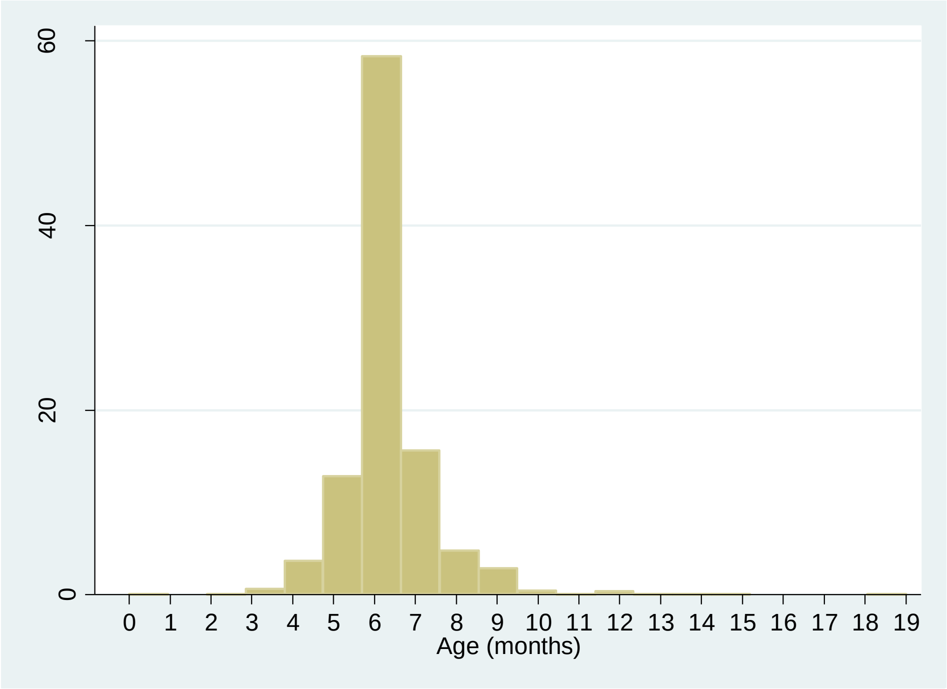

Mesures anthropométriques

Le Tableau 75 présente les principales variables anthropométriques mesurées dans l'enquête de référence auprès des ménages.

Relation entre l'âge et le sexe, et l'état nutritionnel

En faisant une analyse univariée à l'aide d'estimations logistiques, aucune différence statistiquement significative n'a été observée ($p>0.10$) dans les prévalences de la malnutrition aigüe entre les différentes catégories d'âge (Tableau 76). Par contre, les garçons ont une probabilité significativement plus élevée de souffrir de malnutrition aiguë que les filles (Tableau 77).

Tableau 75. Mesures anthropométriques

	Obs.	Moy.	Ecart-type
PB (mm)	6199	139.92	0.28
PB < 115mm	6199	2.0%	0.00
Œdèmes	6199	1.8%	0.00
z-score P/T pour enfants de 6-11mois	2181	-0.25	0.03
z-score P/T pour enfants de 12-17mois	2076	-0.45	0.03
z-score P/T pour enfants de 18-23mois	1941	-0.33	0.03
Malnutrition aigüe (tous les enfants)	6199	6.0%	0.00
Enfants de 6-11 mois avec malnutrition aigüe	2181	5.9%	0.01
Enfants de 12-17 mois avec malnutrition aigüe	2076	6.9%	0.01
Enfants de 18-23 mois avec malnutrition aigüe	1942	5.1%	0.00
Malnutrition aigüe sévère (tous les enfants)	6199	1.1%	0.00
Enfants de 6-11 mois avec MAS	2181	1.1%	0.00
Enfants de 12-17 mois avec MAS	2076	1.3%	0.00
Enfants de 18-23 mois avec MAS	1942	1.0%	0.00
z-score T/A pour enfants de 6-11 mois	2181	-1.71	0.04
z-score T/A pour enfants de 12-17 mois	2076	-2.18	0.03
z-score T/A pour enfants de 18-23 mois	1942	-2.46	0.04
Retard de croissance (tous les enfants)	6199	53.1%	0.00
Enfants de 6-11 mois avec retard de croissance	2181	38.7%	0.01
Enfants de 12-17 mois avec retard de croissance	2076	56.2%	0.01
Enfants de 18-23 mois avec retard de croissance	1942	66.0%	0.01
Retard de croissance sévère (tous les enfants)	6199	21.0%	0.00
Enfants de 6-11 mois avec retard de croissance sévère	2181	12.2%	0.01
Enfants de 12-17 mois avec retard de croissance sévère	2076	22.5%	0.01
Enfants de 18-23 mois avec retard de croissance sévère	1942	29.2%	0.01
Pas de courbe de croissance dans le carnet de santé	4917	90%	0.02
Courbe ascendante	4917	10%	0.02
Courbe stationnaire	4917	0%	0.00
Courbe descendante	4917	0%	0.00

Tableau 76. Relation entre l'âge et le taux de malnutrition aigüe – analyse univariée utilisant les estimations logistiques

	Taux de MA	Régression logistique			
**Catégorie d'âge**		Coef.	Ecart-type	t	p
**6-11mois**	5.9%				
**12-17mois**	6.9%	0.17	0.15	1.14	0.26
**18-23mois**	5.1%	-0.16	0.12	-1.29	0.20

Note : MA signifie Malnutrition Aigüe. Les estimations tiennent compte l'effet de grappe.

Tableau 77. Relation entre le genre et le taux de malnutrition aigüe – analyse univariée utilisant les estimations logistiques

	Taux de MA	Régression logistique			
**Genre**		Coef.	Ecart-type	t	p
**Fille**	5.0%				
**Garçon**	7.1%	0.37	0.12	2.99	0.00

Note : MA signifie Malnutrition Aigüe. Les estimations tiennent compte l'effet de grappe.

Relation entre état de santé et statut nutritionnel

Une autre analyse univariée avec des estimations logistiques a observé une différence statistiquement significative dans les taux de malnutrition aigüe entre les enfants qui ont été malades durant les deux semaines précédentes et ceux qui ne l'ont pas été. Les enfants qui ont été malades étaient les plus exposés (7.1% versus 4.4%; Tableau 78). Par contre, il n'existe pas de différence statistiquement significative dans les taux de malnutrition aigüe sévère entre les enfants ayant été malades et ne l'ayant pas été (1.3% versus 0.9%; Tableau 79).

Tableau 78. Relation entre état de santé et taux de malnutrition aigüe –analyse univariée utilisant les estimations logistiques

	Taux de MA	Régression logistique			
**Episode de maladie les deux semaines précédentes**		Coef.	Ecart-type	t	p
**Non**	4.4%				
**Oui**	7.1%	0.51	0.11	4.51	0.00

Note: Les estimations tiennent compte de l'effet de grappe

Tableau 79. Relation entre état de santé et taux de malnutrition aigüe sévère –analyse univariée utilisant les estimations logistiques

	Taux de MAS	Régression logistique			
**Episode de maladie les deux semaines précédentes**		Coef.	Ecart-type	t	p
**Non**	0.9%				
**Oui**	1.3%	0.33	0.23	1.39	0.17

Note: Les estimations tiennent compte de l'effet de grappe

Relation entre les variables socio-économiques du ménage et le statut nutritionnel de l'enfant

Cette enquête de référence confirme des corrélations entre les variables socio-économiques et le taux de malnutrition aigüe (Tableau 80-Tableau 82). Premièrement, les enfants appartenant aux ménages des quintiles socio-économiques les plus favorisés sont significativement moins exposés à souffrir de la malnutrition aigüe comparés à ceux appartenant aux ménages des quintiles socio-économiques les plus défavorisés. Deuxièmement, les responsables des enfants ayant atteint un niveau d'éducation secondaire ou supérieure étaient moins à risque d'avoir des enfants souffrant de la malnutrition aigüe que ceux n'ayant pas été éduqués. Troisièmement, les ménages qui ont expérimenté une insécurité alimentaire grave sont statistiquement plus exposés à avoir des enfants souffrant de la malnutrition aigüe que les ménages ne connaissant pas d'insécurité alimentaire.

Tableau 80. Relation entre les quintiles socio-économiques et le taux de malnutrition aigüe – analyse univariée utilisant les estimations logistiques

	Taux de MA	Régression logistique			
**Quintile**		Coef.	Ecart-type	t	p
**1**	8.7%				
**2**	7.1%	-0.21	0.14	-1.46	0.15
**3**	5.7%	-0.45	0.15	-3.07	0.00
**4**	4.4%	-0.73	0.15	-4.92	0.00
**5**	4.2%	-0.78	0.17	-4.47	0.00

Note: Les estimations tiennent compte de l'effet de grappe

Tableau 81. Relation entre le niveau d'éducation des responsables des enfants et le taux de malnutrition aigüe – analyse univariée utilisant les estimations logistiques

	Taux de MA	Régression logistique			
**Niveau d'éducation**		Coef.	Ecart-type	t	p
**0 (aucune)**	6.4%				
**1 (primaire)**	5.5%	-0.16	0.13	-1.29	0.20
**2 (secondaire or supérieur)**	2.2%	-1.10	0.47	-2.33	0.02

Note: Les estimations tiennent compte de l'effet de grappe

Tableau 82. Relation entre le score de sécurité alimentaire des ménages et le taux de malnutrition aigüe - analyse univariée utilisant les estimations logistiques

	Taux de MA	Régression logistique			
**Score de sécurité alimentaire**		Coef.	Ecart-type	t	p
**1**	3.2%				
**2**	3.7%	0.17	0.43	0.38	0.70
**3**	4.4%	0.35	0.31	1.11	0.27
**4**	7.0%	0.83	0.27	3.06	0.00

Note: Les estimations tiennent compte de l'effet de grappe

Synthèse

Les données ont été collectées auprès de 6,199 ménages et enfants de 6-23 mois entre le 8 décembre 2014 et le 13 janvier 2015.

La méthodologie utilisée et suivant les questions génériques HFIAS d'échelle de l'accès déterminant l'insécurité alimentaire des ménages pour la mesure de l'accès alimentaire des ménages a permis d'observer que 68% des ménages enquêtés subissaient une grave insécurité alimentaire, 19% une insécurité alimentaire modérée, et 13% une insécurité alimentaire faible ou nulle. Ces chiffres sont à la fois impressionnants et inquiétants et mettent certainement en évidence un domaine qui nécessite de l'attention.

Plus de la moitié des enfants enquêtés avaient été malades durant les deux semaines précédant l'enquête (59%), et 81% d'entre eux ont consulté les services de santé, ce qui semble assez élevé si on note que 50% des répondants reportaient une distance au centre de santé supérieure à une heure. La majorité des indicateurs relatifs à l'allaitement étaient satisfaisants au regard des recommandations nationales d'alimentation du jeune enfant (ANJE). Cependant, les pratiques d'alimentation complémentaire n'étaient en moyenne pas satisfaisantes : seuls 50% des enfants avaient une diversité alimentaire suffisante, à savoir minimum quatre groupes alimentaires, et 44% avaient une fréquence de repas suffisante ; au total, seuls un quart des enfants recevaient une alimentation complémentaire acceptable en terme de fréquence et de diversité (indicateurs adaptés selon leur âge).

Les données collectées en décembre 2014 et janvier 2015 ont permis d'observer un taux de malnutrition aigüe parmi les enfants de 6-23 mois de 6.0%, et un taux de malnutrition chronique de 53.1% à cette période. Les analyses bivariées et multivariées ont confirmé un certain nombre de relations connues entre les taux de malnutrition et les déterminants socio-économiques, à savoir que les taux élevés de malnutrition aiguë sont observés dans les ménages avec des scores socio-économiques inférieurs (ici, les deux quintiles les plus bas), qui souffrent d'insécurité alimentaire et dont les mères ont un faible niveau d'éducation. En outre, la relation bien connue entre les taux élevés de malnutrition aiguë chez les enfants qui avaient été récemment malades (dans les deux dernières semaines) a également été observée, ce qui souligne le cercle vicieux de la maladie et de la malnutrition. Les résultats plus exceptionnels de cette enquête comprennent la prévalence de la malnutrition aiguë significativement supérieure chez les garçons par rapport aux filles. Enfin, il n'y avait pas de différence significative dans les taux de malnutrition aiguë entre les groupes d'âge, mais cela pourrait être expliqué par le fait que la

tranche d'âge des 6-23 mois sont les plus à risque, et ainsi au sein de cette tranche d'âge le risque est susceptible d'être similaire quel que soit l'âge.

5. Validité interne de l'étude

5.1 Puissance de calcul ex-post

Puissance de calcul ex-post au niveau des centres de santé

La taille de l'échantillon des fiches individuelles de suivi à collecter avait été calculée sur la plus petite différence de taux de guérison qui pouvait être considérée comme significative d'un point de vue de santé publique ; on avait alors fait l'hypothèse que l'intervention FBP Nutrition pourrait permettre une augmentation du taux de guérison de la malnutrition aigüe de 80% à 90%, en considérant une erreur de type alpha de 5%, un pouvoir statistique de 80% et un coefficient de correlation intra-classe (ou ICC) de 0.15 (Kaiser et al. 2006). C'est ainsi que le nombre de fiches individuelles de suivi à collecter par service nutritionnel (SSN et STA) a été établi à 12.

Selon les données collectées, les taux de guérison de malnutrition aigüe modérée et sévère sont respectivement de 80% (N=628) et 85% (N=665). Ces chiffres sont proches de notre hypothèse de départ, mais avec un nombre inférieur d'observations. Comme c'est dit dans la section sur les résultats, plusieurs dossiers médicaux manquaient ou étaient pauvrement documentés, la conséquence étant que nous étions dans l'incapacité d'avoir le résultat du programme, i.e. de savoir si le cas de malnutrition aigüe avait été guéri ou pas. De plus, il a été constaté que la qualité de la documentation ainsi que la performance de services sont fortement liées au niveau même des centres de santé. Ainsi pour les SSN, l'ICC équivaut à 0.41 (basé sur les observations au niveau de 26 centres de santé pour lesquels on a eu des informations provenant de 12 dossiers médicaux), ce qui fait chuter la puissance de notre échantillon à 60% pour une augmentation du taux de guérison passant de 80% à 95%. Espérons que les dossiers médicaux seront mieux tenus et mieux remplis pour la dernière enquête.

En ce qui concerne les STA, l'ICC équivaut à 0.06. Il a été calculé sur base des observations de 32 centres de santé pour lesquels on a obtenu des informations pour 12 dossiers individuels. On espère avoir la même puissance de 80% et la même α-erreur de 5% en supposant que l'intervention aboutira en une augmentation du taux de guérison de la malnutrition aigüe sévère passant de 85% à 95%. Cela dépendra de la qualité de la tenue des dossiers médicaux ainsi que de leur remplissage avant la dernière enquête.

Puissance de calcul ex-post au niveau des ménages

La taille de l'échantillon d'enfants à enquêter avait été calculée sur base du taux de malnutrition aigüe dans ce groupe d'âge de 6-23 mois au Burundi. L'hypothèse de départ était de 10%, et était basée sur les données de l'enquête démographie et santé 2010 (EDS 2010). Les données collectées ici observent en fait un taux de malnutrition aigüe de 6%, mais une corrélation intra-grappes bien moindre à ce qui avait été supposé lors du calcul initial (ICC=0.007, au lieu de 0.25). En prenant ces données en compte, la puissance de calcul d'une réduction du taux de malnutrition aigüe de 6% à 4.5% est réduite à 60% ; elle

demeure à 80% si on observe une réduction du taux de malnutrition aigüe plus importante, à savoir de 6% à 4%.

5.2 Obstacles rencontrés et implications pour la validité de l'étude

Enquête au niveau des centres de santé

Questionnaire SSN

Le questionnaire SSN a été confronté à la mauvaise tenue des dossiers médicaux ; cela va des dossiers manquants à la mauvaise qualité de la documentation. Il n'y avait pas de dossiers médicaux SSN dans 16 centres de santé, et dans d'autres centres le nombre de dossiers médicauxs était plutôt faible. Pour faire face à ce problème, les enquêteurs devaient rechercher l'information dans le registre y relatif. Mais ces registres souffrent aussi d'une relative mauvaise tenue et gestion. L'équipe d'enquête n'a pu retrouver les données pour 12 cas de malnutrition aigüe modérée que dans 26 centres de santé parmi les 90 ; dans les autres centres, le nombre de cas était inférieur à ce quota qu'on s'était fixés.

De plus, pour certaines questions, il aurait été intéressant de connaitre la source de l'information pour savoir si elle provenait du dossier individuel du patient ou du registre ; nous n'avions malheureusement pas anticipé ce problème et le design du questionnaire ne permettait pas de collecter cette information aisément. Il aurait également été intéressant de connaître la qualité de la documentation pour chaque élément, pour savoir si c'était correctement rempli (on a eu par exemple une croix à la place de la quantité d'aliments ou le traitement reçu non rempli, etc.). Il s'agit là de points qui pourront être pris en compte dans le design des questionnaires de la prochaine enquête.

Les ruptures de stock des aliments de supplémentation et des médicaments en provenance du niveau central ou du PAM constituent un problème additionnel, qui ne dépend pas directement des agents de santé, mais qui les a probablement influencés dans l'information rapportée dans les dossiers ou les registres. Néanmoins, un bon travail d'archivage aurait documenté quand ces ruptures de stocks ont lieu.

Questionnaire STA

Ce questionnaire a aussi souffert de la mauvaise tenue des dossiers médicaux. La mauvaise tenue allait des dossiers manquants à la pauvreté de la documentation provenant des dossiers existants. Il n'y avait pas de dossiers médicaux STA dans 9 centres de santé, et dans bien d'autres, le nombre de dossiers disponibles était réduit. Pour faire face à ce problème, il était également demandé aux enquêteurs de rechercher l'information dans le registre y relatif. Malheureusement, la mauvaise tenue des dossiers et la mauvaise qualité de la documentation demeure également problématique à ce niveau. Finalement, il nous a été possible de retrouver le bon nombre de 12 cas de malnutrition aigüe sévère dans seulement 32 centres de santé sur les 90.

Comme pour le questionnaire SSN, il aurait été intéressant de connaitre à l'analyse, la source de l'information pour savoir si elle provient du dossier individuel du patient ou du registre (un défaut dans notre questionnaire). Il aurait également été intéressant de connaître la qualité de la documentation pour chaque élément pour savoir si c'était correctement rempli.

Questionnaire G

Il y a eu un certain nombre de centres de santé où le titulaire était absent ou la personne responsable du service nutritionnel était absente. Ceci revient à signaler que les données ont parfois été difficilement collectées.

Ce questionnaire était sujet à biais potentiel : certains répondants des questionnaires pourraient avoir donné des réponses pas toujours factuelles dans le but de montrer que leurs services fonctionnaient bien, possiblement par peur de réprimandes. Pour éviter cette situation, et dans la mesure du possible, les questions devaient être contre-vérifiées par les enquêteurs. Malheureusement, en dépit des efforts de formation et de supervision, les rapports hebdomadaires des superviseurs révèlent un manque au niveau de la vérification systématique par les enquêteurs. C'est pour cette raison que nous préférons faire preuve d'une certaine prudence vis-à-vis de certaines réponses et statistiques.

Pour l'enquête finale, nous changerons, autant que possible, les questions réservées au titulaire du centre de santé en questions posées aux enquêteurs eux-mêmes : les questions devraient être reformulées pour permettre à l'enquêteur de vérifier l'information au lieu de se contenter de poser uniquement la question. De plus, le contenu du questionnaire devrait être légèrement modifié pour permettre de développer une fiche de score qui donnerait une image plus claire de la performance de la gestion du centre de santé (Edward et al. , 2011 par exemple).

Questionnaire N

De façon générale, comme pour le questionnaire G, il était supposé que l'information fournie par le répondant soit vérifiée par les enquêteurs. Mais comme pour le questionnaire G, cela n'a pas été systématiquement fait et certains répondants pour le questionnaire N pourraient également ne pas avoir été 100% factuels dans leurs réponses pour la même raison mentionnée plus haut. Ceci devrait nous rendre prudents sur l'interprétation des statistiques. Les questions sur les traitements, les équipements spécifiques et les documents sont susceptibles d'être plus fiables étant donné que les enquêteurs ont réellement vérifié leur existence et rapportaient cela dans un registre y relatif.

Pour l'enquête finale, nous recommandons, autant que possible, de changer les questions posées au responsable du service nutritionnel en des questions posées aux enquêteurs eux-mêmes : les questions devraient être reformulées pour permettre à l'enquêteur de vérifier l'information au lieu de se contenter de poser uniquement la question.

Dans la même optique, la question sur le protocole concernant le suivi de la croissance devrait être changée en « quel protocole utilisez-vous pour cette activité ? » (PCIME, burundais, autre, aucun). Le contenu du questionnaire devrait être aussi légèrement revu pour essayer de le rendre moins lourd : les questions sur les places aussi bien que la grille du personnel et les questions relatives au niveau communautaire et au matériel donné ou pas devraient être enlevées. Ceci pour la simple raison qu'elles ne fournissent pas d'information fiable et ou valide. En plus, certains filtres pour la saisie des données ont besoin d'être intégrés.

Questionnaire C

On recense deux principales menaces à la validité des données du questionnaire C. La première est le changement de comportement de l'AS pouvant survenir avec la présence de l'enquêteur dans la salle de consultation (effet Hawthorne). Malgré le fait que les enquêteurs aient informé les AS qu'ils n'étaient pas en train d'être officiellement évalués, il peut toujours y avoir une tendance, de la part de ces derniers, à vouloir être plus consciencieux que d'habitude, dans leur façon de prendre l'histoire du patient ainsi que son examen physique. Ainsi, il n'a pas été possible d'avoir une perception réelle des services de santé quotidiennement fournis aux enfants dans ces centres.

Nous avons eu la certitude que l'effet Hawthorne a joué à travers les entretiens à la sortie avec les responsables des enfants (questionnaire S) : 10% des accompagnants ont déclaré avoir été satisfaits par la visite de ce jour mais que cela était une exception comparativement à leur visite ordinaire. De la même façon, 5% ont dit qu'ils se réjouissaient que leur enfant ait été mesuré, pesé et ont affirmé que cette pratique n'était pas tout à fait ordinaire. Le fait aussi qu'il y ait eu 16 accompagnants d'enfants (3%) qui ont tout simplement affirmé se réjouir que leur enfant ait été mesuré pourrait insinuer que cette pratique devrait être une habitude à chaque visite au centre de santé et non quelque chose d'inhabituel/occasionnelle. Néanmoins, ce risque était prévu, et cet effet Hawthorne, qui nous fait sur-estimer la qualité des consultations pédiatriques, ne fait que renforcer notre préoccupation sur la mauvaise prise en charge intégrée des enfants.

La deuxième menace à la validité était qu'il s'agissait d'observations, c'est-à-dire une que la collecte des données était potentiellement contrainte par une baisse épisodique dans l'attention de l'enquêteur ou des difficultés d'observation ou d'écoute. Cela signifie que l'enquêteur a pu manquer certaines questions posées, un conseil donné ou un examen physique réalisé, du fait que les consultations se font très souvent rapidement et que l'enquêteur avait reçu comme instruction de n'interférer en aucun cas dans la consultation. Ce problème peut se poser pour certains gestes plus que pour d'autres. Par exemple l'AS aurait pu rechercher une hépatomégalie, et s'il le faisait discrètement et sans en aviser l'enquêteur, alors il se pouvait que ce dernier n'ai pas vu et reporté cet examen. A contrario, l'examen des oreilles est lui plus évident.

Ce risque avait été identifié dès la formation. Nous avons essayé d'atténuer cette menace par une formation soutenue des enquêteurs en faisant recours à des vidéos montrant ce qu'est une consultation réelle et aussi par des jeux de rôle où nous avons essayé de simuler des consultations telles qu'on les rencontre réellement sur le terrain. Nous avons aussi donné comme instructions aux enquêteurs la possibilité de demander à l'AS en fin de consultation certaines informations ratées au cours de la consultation, comme le diagnostic ou le traitement (une fois que l'enfant et son responsable sont partis). Ils pouvaient alors demander quelles décisions l'AS avait pris (si du moins il ne l'avait pas clairement expliqué à l'accompagnant). Il leur a aussi été demandé de regarder le carnet de santé de l'enfant ou tout autre document qui leur est remis durant la visite au centre de santé, pour voir si les mesures anthropométriques avaient été prises (dans le cas où l'AS n'en avait pas discuté avec la personne responsable de l'enfant). Ceci dit, le fait que l'AS ne discutait pas avec le responsable de l'enfant des aspects concernant la croissance de celui-ci constituait une information en soi.

Questionnaire S

Les risques pour la validité des données du questionnaire S proviennent surtout des biais de rappel de la part des accompagnants des enfants, ou encore du fait qu'ils n'aient pas compris que les enquêteurs étaient indépendants du centre de santé : il est en effet possible que les accompagnants aient pu ne pas se sentir à l'aise pour répondre, craignant des retombées négatives sur les soins accordés à leur enfant dans le futur. Quelques preuves de cela proviennent du fait que 86% des personnes responsables de l'enfant ont rapporté que tout était satisfaisant lors de leur visite, mais 81% avaient des suggestions en vue de l'amélioration de la qualité des services.

L'autre problème soulevé était le manque potentiel d'équipements de mesure au centre de santé comme les balances, le kit de mesure et les bandes MUAC. Cela signifie qu'il n'était pas toujours possible de faire une comparaison entre les mesures prises par l'enquêteur avec son équipement et celles prises avec l'équipement du centre de santé.

Questionnaire V

Le questionnaire V était relativement direct et précis, et nous soupçonnons peu de menaces sur la validité des données. Les enquêteurs avaient reçu comme instruction d'informer les AS, à propos des tests de laboratoire prescrits, qu'ils disent ce qu'ils aimeraient faire dans une situation idéale et selon les besoins cliniques rencontrés, sans se baser sur ce qui était disponible dans leur centre de santé. Cependant, c'est toujours probable qu'ils n'aient pas bien compris cela et par la force de l'habitude, qu'ils aient tout simplement demandé les tests auxquels ils savaient qu'ils avaient habituellement accès. Il en est de même pour l'équipement nécessaire pour les examens physiques (exemple : otoscope). Une formation claire des enquêteurs à ce sujet devrait avoir permis de diminuer ce risque.

Enquête auprès des ménages

Les menaces sur la validité des données ménages sont les mêmes que pour toute grande enquête reposant sur les réponses de la personne interviewée réalisée à son domicile. Les répondants pourraient vouloir donner bonne impression de leur statut socio-économique et éducationnel et ainsi ne pas être 100% factuels dans leurs réponses. Pour la sécurité alimentaire, l'état de santé de l'enfant et l'alimentation de complément, il pourrait y avoir eu des biais de rappel de la part des responsables de l'enfant, surtout pour les questions de sécurité alimentaire qui sont relatives aux quatre dernières semaines et celles sur l'état de santé de l'enfant aux deux dernières semaines.

Les mesures anthropométriques doivent respecter plusieurs règles (ex. les balances doivent être posées sur une surface plane). Les enquêteurs avaient reçu une formation soutenue pour essayer d'éviter ce genre de pièges ou d'erreurs. De plus, un exercice d'assurance qualité de lots (LQAS) a été réalisé afin de contrôler la qualité des mesures prises ; celles-ci ont été jugées satisfaisantes.

5.3 Equilibre de l'échantillon: résultats des tests de différence

Enquête au niveau des centres de santé

La plupart des variables calculées à partir de tous les questionnaires ont été testées pour la différence entre le groupe contrôle et le groupe intervention (cf. tableaux en annexe, à partir de la page 148). Pour

la majorité d'entre elles, la différence est non significative. Cela confirme que les deux groupes sont comparables ; ce qui permet d'être confiant sur la qualité future de notre étude d'impact.

Au niveau de quelques variables, les deux groupes étaient différents. Dans la plupart des cas, ces variables sont des facteurs confondants ou des variables de peu d'intérêt. Cependant, certaines différences apparaissent au niveau des variables d'intérêt.

Premièrement, le WHZ à l'admission du service SSN, calculé à partir des fiches individuelles de suivi et registres, est trouvé être significativement différente pour le groupe intervention (-2.23) et le groupe contrôle (-1.90), avec un t-test équivalant à -2.30. Toutefois, la médiane est similaire (-2.31 dans le groupe d'intervention contre -2.24 dans le groupe contrôle) et la différence semble être due à quelques valeurs aberrantes. De plus, notre principale variable d'intérêt dans ce questionnaire, c'est-à-dire, le « taux de guérison » est, lui, similaire dans les deux groupes.

Deuxièmement, le WHZ à la sortie du STA, calculé à partir des dossiers médicaux et des registres est significativement différent entre le groupe d'intervention (-1.60) et le groupe de contrôle (-0.97) avec un t-test à -2.77. Les médianes sont proches mais restent différentes (-1.36 dans le groupe d'intervention contre -1.06 dans le groupe de contrôle). Cependant, le nombre d'observations est fortement réduit, et notre principale variable d'intérêt dans ce questionnaire, le « taux de guérison » est, lui, similaire dans les deux groupes.

Troisièmement, dans les entretiens à la sortie, il y a des différences significatives pour certaines variables comme: le « diagnostic rapporté par l'accompagnant de l'enfant est le vrai diagnostic » ; « des conseils nutritionnels étaient également rapportés par les responsables des enfants ». Ces conseils incluent ceux concernant la diversification des groupes d'aliments. Une autre différence significative a été trouvée dans les WHZ tels que mesurés par les AS : -0.88 dans le groupe d'intervention contre -0.41 dans le groupe de contrôle (t-test : -2.40). Cependant, nous n'avons pas trouvé de différence entre les mêmes WHZ tels que mesurés par les enquêteurs, ce qui suggère que la différence est le résultat de mesures incorrectes faites par les AS (cette différence reste faible cependant). Enfin, la malnutrition sévère se retrouve plus dans le groupe de contrôle (20%) que dans le groupe d'intervention (15%) selon les mesures faites par les enquêteurs avec un t-test de -2.48.

Enquête auprès des ménages

Les variables de tous les questionnaires ont été testés pour la différence entre le groupe de contrôle et le groupe d'intervention (cf. tableaux en annexe, à partir de la page 159). Pour la majorité d'elles, la différence est non significative, suggérant que les deux groupes sont comparables. Seules les catégories de sécurité alimentaire montrent une différence significative : dans le groupe d'intervention, 64% des ménages souffrent d'une grave insécurité alimentaire tandis qu'ils sont 72% dans le groupe de contrôle (t-test : -2.08). Comme c'est une variable confondante, cela ne devrait pas affecter les résultats de l'évaluation de l'impact.

6. Discussion

Dans cette section, nous revenons sur les principaux résultats de l'enquête et explorons certaines explications possibles de la faible performance documentée dans la prise en charge de la malnutrition par les centres de santé.

6.1 Enquête sur au niveau des centres de santé

Performance des services de nutrition pour les de MAM (SSN)

A cause de la faible disponibilité et de la mauvaise qualité de la documentation dans les centres de santé, il y a un certain nombre d'aspects concernant la performance de ce service qui sont difficiles à évaluer. Cependant, certains problèmes nécessitent une attention particulière. Alors que pour la plupart des indicateurs de performance du service SSN, les recommandations du protocole national étaient atteintes (excepté pour le gain de poids moyen par jour et la durée de séjour dans le service), on constate toujours des diagnostics incorrects de MAM. En effet, à l'admission dans le service, 17% des enfants avaient un WHZ<-3, et souffraient donc de MAS, de même que 3.8% avaient des œdèmes (faisant d'eux également des cas de MAS). Ces enfants courent de plus grands risques et ils auraient dû être admis dans le STA plutôt que dans le SSN. De plus, parmi les 502 cas rapportés comme guéris à la sortie du programme SSN, 49 cas (10%) avaient encore un WHZ à la sortie <-2 : ces enfants sortaient donc tout en étant toujours à plus grand risque de morbidité. Des évidences de diagnostics incorrects de malnutrition aigüe ont également été retrouvés dans les résultats des questionnaires C et V.

En ce qui concerne le traitement systématique, 64.7% des enfants admis en SSN n'ont pas reçu le traitement prévu par le protocole national ; il faut cependant rappeler qu'ils auraient pu l'avoir déjà reçu au niveau du STA (s'ils étaient passés par là). Néanmoins, encore une fois, la faible qualité de la documentation ne permettait pas de savoir avec précision quels cas provenaient du STA et quels cas avaient été directement admis dans le SSN (sans référence).

Une certaine tolérance vis-à-vis des AS pourrait être de mise : on peut en effet comprendre qu'ils soient démotivés par rapport aux ruptures de stock en aliments de supplémentation ; cela ne devrait néanmoins pas totalement excuser le mauvais archivage des dossiers ou les soins médiocres.

Performance des services de nutrition pour les cas de MAS (STA)

La moyenne de WHZ à l'admission dans le STA était au-dessus de -3 (elle était égale à -2.45), signifiant qu'il y avait potentiellement eu, un mauvais diagnostic pour ces enfants. Mais cela ne traduit pas l'image complète de la situation. Il se pourrait qu'il y ait eu beaucoup d'enfants avec un WHZ compris entre -3 et -2 mais avec un MUAC qui était inférieur à 115mm et/ou des œdèmes et/ou une complication médicale sévère qui auraient fait d'eux des cas de MAS et donc des candidats appropriés pour le STA. Mais en raison de la non disponibilité et de la faible qualité des données cliniques concernant les patients, il nous est impossible d'apprécier de façon objective ces décisions cliniques.

Les indicateurs de la performance pour le STA répondent également à la plupart des recommandations nationales (à l'exception du gain de poids moyen par jour et la durée de séjour dans le service). Cela dit,

parmi les 568 cas rapportés guéris, 29 cas (5%) avaient un WHZ à la sortie encore <-3, et souffraient donc encore de MAS.

Aspects organisationnels des services de nutrition (SSN et STA)

Même si nous avons des doutes sur certaines données du questionnaire N (ces dernières n'ayant pas été toutes contre-vérifiées par les enquêteurs), ont toutefois pu être observés certains aspects intéressants qui pourraient être associés à la faible motivation des AS (et donc à la performance) ainsi qu'à l'échec de pouvoir atteindre la moyenne quotidienne du gain de poids et la durée recommandée de séjour dans les SSN et STA. Le questionnaire N a en effet révélé un taux élevé de rupture de stock, non seulement en aliments de suppléments pour le SSN et le STA (plus de 89% des centres de santé ont connu une rupture de stock dans les trois derniers mois), mais aussi dans la documentation requise (par exemple, fiches individuelles de suivi vierges à compléter) avec moins de 10% des SSN et STA ayant tous les documents nécessaires pour réaliser une bonne documentation. Plus surprenant et inquiétant encore, 100% des centres de santé ont connu une rupture de stock en médicaments nécessaires pour le traitement systématique dans les trois derniers mois. Il faut noter que concernant les aliments de supplémentation, moins de la moitié des STA et aucun des SSN ne calculait les quantités mensuelles requises obtenues par calcul des consommations mensuelles (CMM). D'autres part, plus de 75% des SSN et STA avaient leurs CMM calculées pour les médicaments nécessaires dans le traitement systématique (excepté pour le vaccin contre la rougeole). Cela pourrait suggérer que le manque des aliments de supplémentation est un problème de gestion des stocks au niveau des centres de santé alors que le manque en médicaments est un problème au-delà des capacités des centres de santé et est à un niveau supérieur.

Un problème fondamental qui pourrait être lié au constat observé de diagnostics incorrects de malnutrition aigüe est le fait que, selon le questionnaire N, seulement 14% des centres de santé disposaient de la totalité de l'équipement nécessaire pour la prise des mesures anthropométriques (dans un état fonctionnel). Cela n'explique néanmoins pas totalement les diagnostics incorrects, si on repense à la faible performance des AS à mesurer la taille et le PB, ainsi que dans leur manque d'évaluation des œdèmes (cf. questionnaires S, C et V).

Fourniture des soins de santé des enfants - Performance et connaissances des agents de santé

Anamnèse

Dans cette étude, la performance des AS a été évaluée sur base de leur acceptation et adhérence aux protocoles ; ici, il s'agissait du protocole en rapport avec la PCIME, le livre de poche de pédiatrie de l'OMS et le protocole national burundais pour la prise en charge de la malnutrition. En général, cette étude a observé de façon constante la faible performance des AS en charge de la consultation pédiatrique. Pourtant, les recommandations de la PCIME ont été développées à destination des centres de santé primaires des pays à faible revenu, qui sont connus comme n'ayant pas de médecins, et pour lesquels ce sont le plus souvent des infirmiers ou agents de santé communautaires, etc., qui évaluent et prennent en charge les problèmes des enfants. Le premier objectif de l'approche PCIME est d'identifier, à travers les « signes de danger », les enfants sérieusement malades ayant besoin d'être référés au niveau secondaire ou tertiaire. Du questionnaire C, il ressort que les AS ont posé les trois questions

relatives aux « signes de danger » dans seulement 0.3% des consultations observées (au total 2 cas sur 514) ; pour 62% des enfants observés en consultations, aucun des trois signes de danger n'a été investigué. Cette négligence généralisée de la quête des signes de danger, directement liés à la mortalité, est une défaillance majeure de la première ligne du système de santé du Burundi.

Une autre priorité dans la PCIME est de s'inquiéter à propos des « cinq principaux symptômes». La logique sous-jacente est de simplifier et standardiser les consultations pédiatriques pour garantir que les systèmes vitaux sont examinés pour protéger le malade. L'enquête révèle que tous ces symptômes ont été considérés par l'AS pour seulement 0.3% des enfants observés en consultation.

Parmi ces cinq symptômes, les AS s'intéressaient surtout à la diarrhée, la toux et la fièvre ; cela pour plus de 50% des enfants vus en consultation. Ils ont cependant généralement oublié de poser des questions d'exploration ou d'effectuer des examens physiques complémentaires en cas de réponse positive. Par exemple seulement 3.5% des enfants qui avaient de la diarrhée ont reçu tous les examens physiques nécessaires à la recherche de déshydratation. Les AS ont demandé aux accompagnants si leur enfant avait des difficultés de respiration pour seulement 9% des enfants qui avaient la toux ; et parmi les 21 cas chez qui on a observé une difficulté à respirer, la fréquence respiratoire a été mesurée chez moins d'un quart. On pourrait simplement argumenter qu'un enfant tachypnéique est facilement identifié par un clinicien avéré ; mais l'importance de prendre la fréquence respiratoire pour décider de davantage de soins à suivre ne peut pas être sous-estimée. La fièvre est un signe très commun chez les enfants avec beaucoup de causes, et pourtant aucun enfant présentant de la fièvre ne s'est vu poser les six questions complémentaires initiales pour essayer de trouver la source de cette fièvre. On a demandé à seulement 12% de ces enfants fébriles la présence de convulsions, qui n'est pas seulement un signe de danger mais aussi un facteur très important par rapport à la prise en charge immédiate d'un enfant malade. A nouveau, ces chiffres sont extrêmement inquiétants et dénotent une très faible qualité dans la consultation pédiatrique de première ligne dans le Burundi rural.

L'une des dernières priorités de la PCIME est de profiter de la consultation pour vérifier le statut vaccinal ; dans cette enquête, les AS ne sont enquéris de cela que pour 13.5% des enfants. Les services de vaccination sont généralement bien gérés et constitutent une intervention de santé publique simple mais très efficace pour réduire la mortalité infanto-juvénile. Il semble qu'au Burundi, son suivi n'est pas optimisé.

Examens

Le principal pilier pour assurer des soins de qualité - l'histoire clinique (ou anamnèse) – a été, dans cette étude, systématiquement négligée par les AS. Cette négligence s'est perpétuée au niveau des examens physiques : aucun enfant n'a reçu tous les examens de la PCIME tels que listés dans le questionnaire C. Il ne s'agissait pourtant pas d'examens complexes exigeant une grande expertise ou un équipement spécialisé. Un otoscope est essentiel et devrait être un élément des équipements des services de base (nous reconnaissons néanmoins que cet outil manque souvent dans ce type de contexte). Même si on focalise notre analyse sur les examens les plus basiques, qui sont la mesure des trois signes vitaux, les observations des consultations réalisées dans cette enquête ont révélé qu'aucun enfant n'a eu tous ces signes mesurés, et que 40% des enfants n'ont eu aucun des signes vitaux mesurés. Non seulement c'est

un élément essentiel des soins, mais c'est également vital pour transmettre des informations sur un enfant, soit à un autre membre du personnel dans le même centre, ou lors d'une référence pour assurer une bonne continuité des soins.

La prescription d'examens de laboratoire a été un peu plus difficile à évaluer objectivement comme nous ne savions pas quels genres d'examens pouvaient être faits dans chacun des centres. Cela étant, le raisonnement menant à la prescription de tests de laboratoire pouvait être évalué à travers les vignettes (voir ci-dessous).

Diagnostic et traitement

Cette évaluation sévère de la qualité des soins au niveau des consultations pédiatriques au Burundi repose essentiellement sur la façon dont l'histoire de la maladie a été prise et comment l'examen physique a été conduit. En effet, cette enquête ne nous a pas permis d'évaluer avec précision les diagnostics (spécifiquement dans le cas de diagnostics multiples) ; cela est difficile sans examiner le patient nous-mêmes, malgré les observations réalisées par les enquêteurs. Cependant, si nous examinons les diagnostics les plus fréquents (ainsi que ceux documentés dans la PCIME), nous voyons, par exemple, que parmi les 20 enfants dont le diagnostic retenu était la pneumonie, aucun d'entre eux n'avait tous les symptômes et signes physiques nécessaires pour conduire à ce diagnostic (selon les recommandations de l'OMS). Les diagnostics pour les enfants souffrant de diarrhée étaient variés et incohérents : 32% des cas de diarrhée, par exemple, ont reçu comme diagnostic « infection parasitaire » mais seulement la moitié d'entre eux s'étaient vus prescrire un examen de selles pour la recherche de parasites ; or, il est pratiquement impossible de faire un tel diagnostic sans examen de selles mis à part pour les parasites qui peuvent être vues à l'œil nu (ex : ascaris), et la diarrhée n'est pas le symptôme le plus courant (Brent et al. 2014).

Déterminer l'exactitude des traitement**s** était difficile pour les mêmes raisons que pour le diagnostic (cf. ci-dessus ; surtout du fait qu'un traitement donné pour un diagnostic donné pourrait être correct ; mais si l'AS l'avait donné pour un autre diagnostic qu'ils ont donné à l'enfant, cela serait totalement une indication incorrecte). De plus, nous n'avons pas pu nous rendre compte de tous les médicaments qui étaient disponibles dans les centres de santé le jour de la visite, et si les AS prescrivaient les médicaments sur la base de ce qu'ils savaient être disponibles en pharmacie. Les réponses des responsables des enfants dans le questionnaire S et les données dans le questionnaire N, ont en fait souligné qu'il y avait un manque de médicaments dans beaucoup de centres de santé. Mais les AS auraient toujours pu prescrire les médicaments qu'ils jugent meilleurs et dire à l'accompagnant de l'enfant d'aller dans une pharmacie privée. Si nous regardons quelques-uns des traitements, parmi les 20 enfants diagnostiqués comme souffrant de pneumonie, 19 ont reçu un traitement antibiotique et cela était pour la plupart correct et conforme aux recommandations de l'OMS, que ce soit pour la première ou la deuxième ligne. Comme l'évaluation des enfants souffrant de diarrhée et de déshydratation est médiocre, ce n'est pas possible de dire combien d'enfants souffraient de déshydratation. Il est toujours intéressant de noter que le SRO a été prescrit à seulement 42% des cas de diarrhée, alors que c'est le médicament de base pour la diarrhée dans la communauté. De plus, 39% des cas de diarrhée ont reçu des antibiotiques ce qui est rarement indiqué pour les diarrhées.

Statut nutritionnel

Notre étude d'impact se concentre sur la problématique de la lutte cotnre la malnutrition ; notre appréciation de la PCIME a dès lors été particulièrement attentive à cet aspect. Evaluer le statut nutritionnel d'un enfant est d'ailleurs également un pilier essentiel de l'approche PCIME.

Questions et examens spécifiques à la nutrition

Aucun des enfants de notre échantillon n'a eu toutes les questions spécifiques à la nutrition et seulement 2.5% des enfants ont bénéficié de tous les examens physiques relatifs à la nutrition. Le poids a été la mesure la plus fréquente : 52% des enfants ont été pesés, mais cela signifie qu'il reste un enfant sur deux qui ne l'est pas. La taille et le MUAC sont les mesures qui viennent en deuxième position avec 32% et 30% respectivement. Cette enquête montre de façon inquiétante, que les œdèmes ont été recherchés pour très peu d'enfants (12.5%). Il s'agit pourtant là d'un facteur qui peut complètement changer le diagnostic d'un enfant : un z-score de rapport poids pour taille ou un MUAC normaux ou MAM peuvent faire passer à un diagnostic de MAS si il y a présence d'œdème, ce qui est un diagnostic plus sérieux et exigeant un traitement prompt et complet ; surtout que les enfants avec MAS avec œdème ont des grands risques de mortalité (Manary et Brewster 2000; Singh 2014). La performance la plus médiocre a été observée au niveau de l'analyse et de la discussion par les AS de la courbe de croissance. Cependant nous ne pouvons pas dire si cela est le résultat d'une négligence de cette tâche par l'AS, ou bien de l'oubli du carnet médical de l'enfant (où la courbe est documentée) par l'accompagnant de l'enfant.

Une preuve supplémentaire de cette faible performance en ce qui concerne l'évaluation du statut nutritionnel de l'enfant, vient des réponses des accompagnants des enfants dans le questionnaire S où, malgré qu'ils ne fussent pas nombreux, 5% ont rapporté qu'ils étaient contents que leur enfant ait été mesuré ce jour (comme si cela n'arrivait pas d'habitude). Cela pouvait signifier que l'effet Hawthorne a amené les AS à mener des activités qu'ils n'auraient pas fait en l'absence de nos enquêteurs.

Mesures anthropométriques

Au-delà de ces médiocres performances observées dans la pose de questions et la prise des mesures pour évaluer le statut nutritionnel, nous avons ensuite observé des résultats tout aussi préoccupants en rapport avec l'exactitude de ces mesures et l'exactitude des diagnostics donnés. Nos résultats montrent que les AS ont enregistré des tailles significativement inférieures que les enquêteurs (avec pourtant le même équipement); cela conduit à un calcul de WHZ plus élevé et proche de la médiane, et ainsi à un sous-diagnostic de la malnutrition aigüe (voir aussi plus bas). Au contraire, les PB mesurés par les AS étaient significativement inférieurs à ceux pris par les enquêteurs ce qui pourrait conduire là à un sur-diagnostic de la malnutrition aigüe, ce qui n'a pas été observé dans les résultats ; peut-être que les AS mettent l'accent plus sur les WHZ que sur les PB comme moyen de diagnostiquer de la malnutrition.

Un facteur supplémentaire qui expliquerait le sous-diagnostic de la malnutrition aigüe est la différence significative observée entre les poids pris par les enquêteurs utilisant les balances des centres de santé et les balances de l'enquête. Ces dernières montraient constamment un poids inférieur. Il s'agit là d'un problème de matériel et non d'une erreur humaine. Les auteurs de ce rapport ont noté que dans la majorité des centres de santé qu'ils ont eux-mêmes visités, les balances étaient manuelles, souvent en

mauvais état et difficiles à calibrer avec précision. Il se pourrait que nous soyons face à une situation où les AS font de leur mieux dans la prise du poids des enfants avec l'équipement qu'ils ont. Ils ont par contre besoin d'améliorer leur expérience dans la prise de la taille et du PB (cf. ci-dessus).

Evaluation et prise en charge de la MAM

Comme mentionné plus haut, cette inexactitude des mesures pourrait être un facteur explicant le sous-diagnostic de malnutrition aigüe observé durant cette enquête. En effet, selon les mesures des enquêteurs, il y avait 51 cas de MAM observés durant les consultations, mais seuls 12 ont reçu un diagnostic de MAM (parmi eux, huit ont été référé au service approprié, i.e. le SSN). Cela signifie que 39 cas ont été ratés et n'ont pas été enregistrés dans le système comme MAM, et 43 enfants MAM n'ont pas été référés pour un traitement correct. Il est possible que le fait que les 12 enfants diagnostiqués MAM par les AS n'ont pas tous été référés au SSN parce que la plupart des centres de santé ne disposent plus de services SSN et que le Programme Alimentaire Mondial soutient les activités des SSN dans seulement deux provinces depuis 2014 (communication personnelle, PRONIANUT et PAM 2014). Pendant l'enquête, l'un des auteurs de ce rapport a observé une situation interpelante : un enfant a été identifié comme un cas de MAM lors de la mesure par les enquêteurs ; quand l'AS en a été informé, il a dit que même si l'enfant est diagnostiqué comme MAM, il n'y avait aucun SSN où il pouvait être référé. Cette anecdote illustre le manque de motivation des AS pour activement chercher ces cas dans cette configuration où il n'y a aucun service où ils peuvent être référés. On pourrait toutefois répondre que ces enfants devraient être documentés dans le système (au cas où les services de prise en charge de la MAM redevenaient disponibles) ; que les AS proposent un suivi rapproché de l'enfant ; que les AS donnent au minimum quelques conseils de nutrition. Cela est d'autant plus pertinent que seulement la moitié des 12 enfants diagnostiqués MAM ont reçu des conseils de nutrition ; alors que pourtant l'évidence scientifique montre qu'à eux seuls, les conseils (c'est-à-dire sans supplément nutritionnel), peuvent avoir un impact sur la guérison de la MAM, tant que la participation des responsables des enfants dans les sessions de conseils est assurée (Nikiema et al 2014).

Evaluation et prise en charge de la MAS

Comme souligné précédemment, le diagnostic de MAS est d'une importance capitale, compte tenu du risque associé accru de mortalité. Il y a eu 27 cas de MAS diagnostiqués par nos enquêteurs ; seuls deux enfants avaient reçu ce même diagnostic de la part de l'AS, lors des consultations observées. Ces deux enfants ont été référés aux services appropriés (i.e. STA) ; mais 25 cas n'ont pas reçu le traitement correct relatif à ce sérieux problème. Contrairement aux services SSN (pour enfants souffrant de la MAM), la plupart des centres de santé de l'enquête (89 sur 90) ont rapporté qu'ils assurent les services de thérapeutiques ambulatoires pour les enfants souffrant de la MAS (STA) et qu'ils pouvaient aussi les référer en hospitalisation pour les cas de MAS avec complications (SST). Il y a donc moins de contraintes que pour les cas de MAM, et nous sommes ici vraiment face à des situations où la vie des enfants est en jeu.

Connaissances des AS

Pour essayer d'explorer davantage les connaissances des AS et atténuer certaines des limites de l'observation directe des consultations, notre recherche a aussi eu recours à la technique des vignettes. Trois vignettes cliniques ont été présentées aux AS observés en consultations (questionnaire V). Comme

décrit dans la méthodologie, les AS étaient avisés qu'ils devaient rapporter un maximum : les questions qu'ils pourraient poser à la personne accompagnant l'enfant, les examens physiques qu'ils pourraient effectuer, les examens de laboratoire qu'ils pourraient demander et enfin les diagnostics posés et les traitements prescrits. Dans cet exercice fictionnel, ils devaient partir du besoin clinique plutôt que ce que ce qu'ils croyaient être disponible dans leurs centres de santé (soit un contexte idéal plutôt que celui conforme à leur réalité). Dans le cadre de cet exercice, ils n'avaient pas non plus les mêmes pressions en ce qui concerne le temps comme dans les consultations habituelles.

Compte tenu de tous ces facteurs, nous nous attendions à ce que les AS obtiennent de meilleurs résultats dans les vignettes cliniques que dans les observations directes de la consultation. Cependant, comme rapporté dans la section 4, nous avons de nouveau observé une performance très faible – fort similaire, dans presque tous les aspects de la consultation, à ce qui avait été observé en pratique réelle. Seuls quelques aspects étaient de meilleure qualité[16].

Par exemple, quand on considère le nombre d'AS qui se sont enquis des signes de danger, leur pourcentage varie entre 1.5-3% à travers les vignettes ; cela concernait 0.3% des consultations observées dans le questionnaire C. La proportion d'AS qui n'ont demandé à propos d'aucun de ces signes de danger varie entre 44 et 59% selon les vignettes ; c'est à comparer avec 62% des consultations dans le questionnaire C. En ce qui concerne les cinq principaux symptômes, on constate à nouveau une faible performance dans les vignettes, analogue à celle documentée en situation réelle, avec un pourcentage compris entre 0 et 2% des AS (selon les vignettes) qui demandaient à propos des cinq principaux symptômes (cela avait été demandé dans 0.3% des consultations dans le questionnaire C). Une image semblable a été vue en ce qui concerne les examens physiques. Aucun des AS, quelle que soit la vignette, n'a dit qu'il souhaitait effectuer tous les examens physiques listés ; la même proportion a été observée dans le questionnaire C. Il y avait une légère amélioration dans les vignettes par rapport aux consultations réelles en ce qui concerne les signes vitaux, avec des proportions de 7 à 10% des AS (selon les vignettes) rapportant qu'ils auraient mesuré les trois signes vitaux ; dans le questionnaire C, on n'a observé cela dans aucune des consultations (0%). Cependant la proportion de ceux qui n'ont mesuré aucun de ces signes était relativement similaire avec 17 à 49.5% des AS concernés selon les vignettes, et 40% des consultations réelles concernées.

Dans quelques aspects, les AS ont mieux fait dans les vignettes que dans les observations directes, par exemple, dans l'exercice fictif de la vignette no. 1, 77% des AS se sont inquiétés de la présence d'une toux (comparativement à 51% des consultations dans le questionnaire C) ; et 18.5% d'entre eux se sont inquiétés de la difficulté dans la respiration (comparativement à 9% des consultations avérées avec toux dans le questionnaire C). Mais ce qui restait faible, que ce soit lors de l'administration de la vignette ou lors des consultations réelles avec tous, était ce qui devait suivre, c'est-à-dire poser des questions d'exploration à propos de ces symptômes pour les caractériser davantage et ensuite analyser leur sévérité.

[16] Notons que les comparaisons présentées dans ce rapport ne prennent pas en compte les mêmes unités d'observations: pour les vignettes (questionnaire V), l'unité dobservation est l'AS; tandis que pour le questionnaire C, c'est la consultation. Des comparaisons rigoureuses font actuellement l'objet d'un travail approfondi qui viendra ultérieurement.

Les AS ont été instruits, par les enquêteurs administrant les vignettes, de demander des examens de laboratoire en fonction des besoins cliniques et non de leur disponibilité. Nous nous attendions donc de voir de meilleures performances avec les vignettes à ce niveau que dans les observations directes, mais cela n'a pas été le cas. Cela pourrait aussi être lié à la faible performance dans la prise de l'histoire de la maladie et de la conduite des examens physiques. Par exemple, si l'AS ne disait pas qu'il voulait chercher des signes d'anémie, alors il ne lui était pas communiqué que l'enfant présentait des signes y relatifs, et donc il ne chercherait pas à mener des examens de laboratoires relatifs. Cependant, même parmi les AS auxquels il a été transmis les signes cliniques d'anémie, seulement 35 à 43% d'entre eux selon les vignettes (no. 1 ou 2) ont dit qu'ils effectueraient un test d'hémoglobine. Ils ont légèrement fait mieux en ce qui concerne la demande d'un test de malaria quand l'enfant avait de la fièvre (à l'exception de la vignette no. 2) avec la majorité des AS (plus de 90%) demandant le test en cas d'hyperthermie. Par contre, très peu (entre 3.5% et 4.5% selon les vignettes) ont pensé à demander un test de bandelette urinaire pour un enfant qui a de la fièvre, ce qui suggère une réflexion limitée par rapport aux diagnostics autres que la malaria lorsqu'il y a présence de fièvre.

Les vignettes ont confirmé les faiblesses dans la pose du diagnostic. Les réponses aux questions, et résultats de l'examen physique et des tests de laboratoire ont été conçus pour identifier clairement un diagnostic communément observé dans les soins primaires. Malgré cela, chaque diagnostic correct a été rapporté par moins de 50% des AS (excepté pour le paludisme non compliqué avec 76% des AS qui ont rapporté ce diagnostic correct dans la vignette no.3 ; il est cependant à noter que 25% ont donné le diagnostic du paludisme grave alors qu'il n'y avait pas d'indication pour ce diagnostic dans cette vignette).

Les décisions de traitement des AS dans les vignettes ont montré à la fois une bonne qualité de performance dans certains cas mais une faible performance dans d'autres. Par exemple, pour la vignette no. 1, 90% des 59 AS qui ont donné un diagnostic de pneumonie ont prescrit un antibiotique ; le choix de l'antibiotique, dans ce cas, était raisonnable. Si on considère les données sous un autre angle, on constate que 117 AS ont prescrit un antibiotique dans le cadre de cette vignette alors que seuls 59 d'entre eux avaient donné le bon diagnostic de pneumonie, ce qui signifie que les 58 autres, ayant prescrit l'antibiotique sans avoir diagnostiqué la pneumonie, ont fait cette prescription à tort puisque rien n'indiquait de le faire ainsi. Il ressort donc, qu'il y a un abus de prescription des antibiotiques.

Si on s'intèresse plus particulièrement au dépistage et à la prise en charge de la malnutrition, on retrouve ce même problème de performance, avec toujours ce peu de différence entre les vignettes et ce qui a été mesuré dans les observations directes de consultations réelles. Par exemple, entre 0% et 0.5% des AS selon les vignettes ont posé toutes les questions relatives à la nutrition, ce qui était semblable au 0% de consultations observées dans le questionnaire C. De la même façon, pour l'examen physique relatif à la nutrition, 0.5 à 3% des AS (selon les vignettes) ont effectué tout ce qui était requis ; cela avait été effectué dans 2.5% des consultations observées dans le questionnaire C. La distribution des mesures anthropométriques réalisées dans le cadre des vignettes est également similaire à celle observée en consultation réelle : le poids est la mesure la plus souvent prise et seuls quelques AS ont recherché des œdèmes ; le calcul du WHZ a également été négligé ; mais la tâche la plus négligée était le remplissage de la courbe de croissance ce qui pourrait indiquer que le suivi de la croissance n'est pas

une priorité pour la majorité des AS. En effet, les données du questionnaire N révèlent que moins de la moitié des répondants disent qu'ils dessinent de façon systématique une courbe de croissance des patients. Cinquante-quatre pourcent des répondants de questionnaire N disent qu'ils utilisent un protocole de suivi de la croissance, mais il n'existe actuellement aucun protocole national pour le suivi de la croissance. Il se pourrait qu'ils utilisent la section des recommandations de la PCIME (ce serait alors une faute dans la conception du questionnaire de ne pas capter cela ; à prendre en compte pour la prochaine enquête).

Très peu d'AS ont rapporté un diagnostic correct de la MAS compliquée ou non compliquée pour les vignettes no. 1 et no. 2 (7% et 14% respectivement) ; ils ont fait un peu mieux dans le diagnostic de la MAM dans la vignette no. 3 (26%), mais en général, ces chiffres sur l'identification de la malnutrition sont très préoccupants. Cela l'est d'autant plus que les enfants souffrants de la MAS chez qui on a raté le diagnostic encourent des risques de conséquences sérieuses pour leur santé, sur le court et le long terme. En phase avec ce sombre tableau, il y a eu une tout aussi mauvaise performance sur la prise de décision en matière de référence (à apprécier à la lumière du fait que le diagnostic était si souvent incorrect).

Moins de 50% des AS ont dit qu'ils donneraient des conseils de nutrition aux accompagnants des enfants (19.5 à 41.5% selon les vignettes) ; à comparer avec les chiffres suivants : 24% des consultations observées ont reçu des conseils de nutrition dans le questionnaire C et exactement le même chiffre (24%) des responsables des enfants ont rapporté qu'ils avaient reçu des conseils de nutrition dans le questionnaire S à la sortie de la consultation. Il n'est pas facile de juger avec précision la qualité des conseils de nutrition donnés aux responsables des enfants comme il n'y avait pas de méthodes qualitatives extensives dans nos vignettes. Cependant, nous pouvons voir que la majorité des conseils se contentaient de citer aux responsables des enfants quelques produits alimentaires spécifiques à donner aux enfants, ou de faire référence aux « trois groupes d'aliments » ce qui est en soi une notion dépassée puisqu'elle a été remplacée par celle des « sept groupes » d'aliments (WHO 2010). Or les recherches internationales ont montré que cette manière non personnalisée de donner des conseils a rarement été efficace pour améliorer le statut nutritionnel des enfants, surtout celui de ceux qui souffrent de malnutrition (WHO 2010a; Ashworth et Ferguson 2009).

D'autres conseils ont aussi été négligés par les AS. Par exemple, seulement 19.5 à 36% des AS selon les vignettes ont rapporté qu'ils expliqueraient aux responsables des enfants comment prendre les médicaments, seulement 5 à 12.5% ont rapporté qu'ils discuteraient les signes de danger (et de quand il faudrait ramener l'enfant au centre de santé), et seulement 9 à 14% des AS ont rapporté qu'ils discuteraient du plan de traitement et de suivi de l'enfant avec son responsable pour voir s'ils avaient compris ce qui était nécessaire de leur côté et s'ils avaient des questions.

Pourquoi une si faible performance?

Cette étude de *référence* a documenté de façon consistante la faible performance des AS dans la prise en charge des enfants malnutris au niveau des centres de santé du Burundi, mais aussi de façon plus générale, dans les consultations curatives pédiatriques. L'étude permet de fournir plusieurs pistes pouvant expliquer cette faible performance.

Premièrement, si on regarde la formation initiale de ces AS, il ressort du questionnaire G que seulement 1% des employés des centres de santé listés avaient une formation paramédicale de niveau universitaire; d'ailleurs, la majorité des centres de santé (87%) ne disposent pas de prestataires avec le niveau A1 (diplôme universitaire d'infirmière). Enfin, seulement 20 (22%) centres de santé respectent les normes sanitaires du Burundi concernant le minimum de ressources humaines requis.

Deuxièment, selon notre questionnaire complémentaire aux vignettes V, moins de la moitié des AS interrogés ont rapporté avoir reçu une formation de la part du PRONIANUT, dans le domaine de la nutrition et de l'évaluation de la croissance, depuis la fin de leur formation initiale. De même, moins de 10% des AS ont rapporté avoir reçu une formation sur la santé communautaire (incluant les recommandations de la PCIME) et la communication relative à la santé communautaire. Quand on leur demande, il y a 13 domaines différents dans lesquels les AS identifient des besoins de formation. Les domaines les souvent cités sont le dépistage et la prise en charge de la malnutrition aigüe (67.5% des AS), la prise en charge de la malnutrition aigüe sévère avec complication (51%), le suivi et la promotion de la croissance (36%), les conseils nutritionnels à donner au responsables des enfants (32.5%) et la santé communautaire (inclue la PCIME) (23.5%). Ces derniers résultats sont rassurants en ce sens qu'ils montrent que les AS sont conscients de leurs lacunes.

Un autre facteur pouvant expliquer cette faible performance est la situation au niveau de la supervision. Certaines études ont montré que la formation aurait un impact relativement limité sur la performance des AS, mais qu'en revanche une formation de bonne qualité couplée à des audits et suivis, pouvait avoir un impact très important (Rowe et al. 2005; Grimshaw et al. 2004). Or, moins de 40% des AS interrogés sur les vignettes V ont rapporté avoir reçu une supervision dans le domaine de la malnutrition de la part d'un responsable externe durant les six derniers mois. Ce chiffre a été confirmé par les réponses rendues dans le cadre du questionnaire N.

les usagers ont aussi leur point de vue sur la performance (voir aussi point suivant). Il ressort des réponses des responsables des enfants dans le questionnaire S, que selon eux, il existe des problèmes liés aux conditions physiques du centre de santé lui-même, ses équipements, les fournitures médicales (y compris les médicaments) et le nombre d'AS disponibles au centre de santé. La réalité de ces contraintes et le fait qu'elles soient perçues par les usagers peut constituer un environnement de travail moins favorable qui met le personnel sous pression dans son travail quotidien, aboutissant à des pertes de moral et de motivation (Rowe et al 2005).

Ceci dit, on doit par contre écarter une cause souvent évoquée dans la littérature sur la mauvaise performance des ressources humaines: les incohérences ou les retards de salaires (Rowe et al 2005). En effet, plus de 90% des AS interrogés après les vignettes ont rapporté avoir toujours reçu leur salaire dans leur intégralité durant les 12 derniers mois.

Fourniture des soins de santé pour les enfants – Appréciation par les patients

Une dimension importante de la qualité des soins est la compréhension par le patient (ou ici le responsable de l'enfant) des informations transmises par l'AS vers le patient. Des entretiens effectués à la sortie des consultations (questionnaire S), il a été observé que 66% des responsables des enfants ont

répondu à la question leur demander de rapporter ce qu'ils avaient compris sur le problème de santé de leur enfant. Cette situation est inquiétante, d'autant plus, que parmi eux, seulement 63% étaient capables de rapporter un diagnostic ; les autres 37% étaient juste capables de rapporter des signes et symptômes. Deux explications possibles : soit le diagnostic leur avait été communiqué mais ils l'avaient oublié ou pas compris, soit le diagnostic ne leur avait pas été communiqué et il répondait à l'enquêteur conformément à la symptomatologie de l'enfant qui avait motivé la consultation au centre de santé.

Parmi les 34% de responsables d'enfants ayant rapporté ne pas avoir compris ce qui n'allait pas bien chez leur enfant, la raison principalement donnée (59% des réponses) était que l'AS ne le leur avait pas expliqué. Une confirmation de la mauvaise performance des AS à communiquer le diagnostic se révèle dans le fait que seulement 2 accompagnants d'enfants ont rapporté que leur enfant avait reçu comme diagnostic la malnutrition (sans spécification s'il s'agissait de MAM ou MAS), alors que 14 enfants avaient reçu ce diagnostic en consultation (questionnaire C).

La faiblesse de la communication entre les AS et les patients se retrouve dans le fait que 76% des accompagnants d'enfants ont déclaré que l'AS avait prescrit des médicaments, mais qu'il n'avait pas donné d'autres conseils ou d'autre explication sur l'état ou le traitement de l'enfant (questionnaire S). Ceci correspond au chiffre de 74.5% de consultations pour lesquelles le traitement médical n'avait pas été expliqué (questionnaire C)

En ce qui concerne ce qui a le plus satisfait les responsables des enfants lors de leur visite au centre de santé, il est intéressant de noter que quoique la majorité ait rapporté l'aspect médical (la manière dont l'enfant a été examiné, les questions posées, etc.) comme étant l'aspect le plus satisfaisant (58% des accompagnants), la communication vient en second (46%). Ce résultat, couplé au le fait que le premier élément qui a le plus plu aux accompagnants d'enfants a été la façon dont ils ont été accueillis au centre de santé (40%), montre l'importance de la communication entre les AS et les responsables des enfants. Ces résultats restant en dessous de 50% ; ce qui suggère qu'il y a moyen de faire mieux dans le domaine de la communication.

Très peu d'accompagnants d'enfants ont rapporté que rien ne les avait satisfait lors de leur visite au centre de santé (2%), et, de la même façon, 86% ont rapporté que rien n'avait été insatisfaisant lors de leur visite au centre de santé. Toutefois, comme mentionné dans la section 5 sur la validité de l'étude, avec une enquête à la sortie, on ne pas exclure que les répondants se montrent plus positifs qu'ils ne le pensent vraiment, par égard pour le personnel, par peur de l'impact négatif que cela pourrait avoir sur les soins pourvus à leur enfant. On notera ainsi que, bien que la majorité des accompagnants ait déclaré que tout a été satisfaisant, 81% d'entre eux avaient quand même des suggestions d'améliorations à faire. Ceci est en effet une façon plus confortable d'exprimer ses pensées en rapport avec ce qui ne va pas bien dans le centre de santé, sans avoir à le dire directement.

6.2 Enquête auprès des ménages

Les résultats de l'enquête auprès des ménages sont plus circonscrits. Ils ont confirmé les associations déjà connues entre la malnutrition et les déterminants socio-économiques. Ainsi, les taux élevés de malnutrition aigüe sont rencontrés parmi : (1) les ménages avec un score socio-économique bas (les

deux quintiles les plus bas dans l'enquête) ; (2) ceux qui connaissent une insécurité alimentaire ; et (3) ceux dont les mères ont un niveau d'éducation bas. De telles associations ont été documentées ailleurs dans le monde, aussi bien dans des études anciennes que récentes (Van de Poel et al. 2008; Nagahori et al. 2015; Kismul et al. 2015).

Cette étude a également mis en exergue une association entre le statut de malnutrition aigüe et le fait que l'enfant ait été récemment malade (les deux semaines précédentes). Cette association, qui a également été démontrée dans d'autres pays, met en évidence le cercle vicieux de la malnutrition et de la maladie, au sujet duquel il existe un débat quant au sens de la causalité (Bhutta et Salam 2012).

Certains résultats étaient plus inattendus. Notre étude a révélé une prévalence de la malnutrition aigüe plus élevée chez les garçons que chez les filles. L'absence de différence statistique des taux de malnutrition aigüe entre les différentes catégories d'âge était aussi une surprise. Toutefois, ceci pourrait être expliqué par le fait que la catégorie d'âge la plus exposée aux risques est celle des 6-23 mois comparée aux autres catégories d'âge ; les risques sont potentiellement moins différenciés entre les sous-catégories d'âge à l'intérieur de ce groupe des 6-23 mois qu'on étudie ici (par rapport à une comparaison entre ce groupe d'âge et les plus de 23 mois).Enfin, le résultat relatif à l'insécurité alimentaire grave dans 68% des ménages est à la fois surprenant et alarmant, et ne fait que confirmer que la malnutrition est un défi majeur pour le Burundi.

7. Recommandations

7.1 A l'égard de l'équipe d'évaluation

Améliorer les questionnaires

Les enquêtes de références présentées ici ont bien répondu à nos attentes et sont de bonne qualité pour la plupart des variables. Cela étant, quelques éléments peuvent être modifiés pour améliorer encore la qualité et la complétude des données. Par exemple, il a été noté que certaines réponses aux questionnaires administrés aux titulaires des CDS et aux responsables de service nutrition n'étaient pas toujours contre-vérifiées par les enquêteurs ; une solution serait de formuler différemment les questions, de les adresser directement aux enquêteurs pour qu'ils vérifient directement plutôt qu'ils posent la question. Cela devra également faire l'objet de davantage d'attention durant la formation. De même, les formulaires pour retranscrire les fiches individuelles de suivi de SSN et de STA devraient permettre de connaître la source de l'information (fiche individuelle ou registre) et de collecter plusieurs éléments relatifs à la qualité de remplissage des fiches individuelles. Aussi, tous les filtres dans les programmes de saisie des données devront être vérifiés avant le démarrage des formations des enquêteurs. De même, les manuels des enquêteurs devront être actualisés pour prendre ces changements en compte.

Préparer les enquêtes de suivi

Une fois les questionnaires révisés et finalisés, l'équipe devra à nouveau préparer bien en amont les enquêtes de suivi, et considérer environ quatre mois de travail préparatoire avant le travail sur le terrain, en collaboration avec les instituts mettant en œuvre les enquêtes, l'INSP et l'ISTEEBU, ainsi qu'avec la Banque Mondiale, qui contractualise et finance ces derniers. Ceci doit permettre de bien planifier les activités préparatoires, à savoir le recrutement des enquêteurs et superviseurs, la formation des enquêteurs et superviseurs, le test pilote, l'évaluation des enquêteurs, l'organisation logistique du travail de terrain (plan de déploiement, équipement nécessaire, budget, etc.). La formation devra à nouveau inclure un exercice de standardisation pour optimiser la qualité des données anthropo-métriques. Dans la même optique, un plan d'assurance qualité des données avec LQAS devra à nouveau être pensé et mis en œuvre.

Documenter le processus de mise en œuvre de l'intervention

Il est important d'effectuer un suivi évaluation rapproché de la mise en œuvre de l'intervention, durant toute la période d'exposition, pour permettre d'expliquer par quels mécanismes le FBP Nutrition a un impact, mais aussi pour pourvoir expliquer les raisons d'un éventuel échec. Particulièrement, à ce jour, on note que les intrants diététiques dans le SSN ne sont pas disponibles dans la plupart des provinces, ce qui rend *de facto* les services SSN non effectifs. Pour pallier ce problème, des fournisseurs de farine, chez qui les CDS avec SSN pourront se fournir, doivent être identifiés. A ce jour, neuf mois après le début de l'intervention, cela n'est toujours pas effectif, ce qui rend le FBP Nutrition inefficace en ce qui concerne la prise en charge de la MAM dans le SSN.

De même, les formations au nouveau protocole de prise en charge de la malnutrition aigüe (MSPLS, OMS, UNICEF, 2014) sont seulement en cours : elles ont été réalisées pour le SST, sont en cours de réalisation pour le STA, et n'ont pas démarré pour le SSN, qui est, faute d'intrants, jugé inexistant. Encore une fois, ces éléments risquent de perturber la réussite de l'intervention FBP Nutrition – compte tenu par ailleurs de la faible qualité des services de santé en matière de prévention et prise en charge de la malnutrition relevée dans ce rapport.

Par ailleurs, compte tenu de la situation politico-sécuritaire traversée courant 2015, certains retards ont été observés concernant la mise en œuvre même de l'intervention. Notamment, le volet communautaire de l'intervention, censé démarrer en juillet 2015 n'a finalement été mis en œuvre qu'en octobre 2015. De même, dans le groupe d'intervention, les contrats secondaires entre les CDS nutritionnels (dits « primaires ») et les CDS non nutritionnels qui réfèrent vers les premiers les cas de MA dépistés, permettant, avec le volet communautaire, de compléter la chaîne des agents concernés par la MA, ne sont pas encore établis au moment de l'écriture de ce rapport. La mise en œuvre effective de ces éléments doit être suivie et évaluée en continu.

Enfin, l'équipe d'évaluation doit également faire attention aux éléments externes pouvant altérer l'efficacité de l'intervention. Il s'agit notamment des implications des différents partenaires techniques et financiers dans la lutte contre la malnutrition ; par exemple du PAM en ce qui concerne l'arrêt de la fourniture des intrants pour le SSN. Il s'agit également de prendre en compte les implications des différents engagements des bailleurs de fonds dans le budget de la santé mais aussi dans le budget du pays en général ; la situation économique du pays ; etc.

Plaider pour un bon remplissage et archivage des fiches individuelles de suivi et des registres

Dans ce rapport, il a été discuté à plusieurs reprises la mauvaise qualité du remplissage et de l'archivage des fiches individuelles de suivi et des registres des services SSN et STA ; et on a vu que ceci affectait la puissance de calcul ex-post de l'évaluation d'impact au niveau des CDS. Aussi, afin d'optimiser le pouvoir statistique de cette évaluation, l'équipe se doit de plaider pour un bon remplissage et archivage de ces fiches et registres.

Organiser des formations personnalisées sur demande des CDS

Partant du manque de connaissances des AS révélé par cette étude de référence, l'équipe propose deux formations pour améliorer la qualité des services, notamment relatifs à la prévention et la prise en charge de la malnutrition aigüe mais aussi à la promotion de la croissance. Ces formations ont été proposées lors de l'atelier d'assignation aléatoire du 1er décembre 2014, où les titulaires ont exprimé, sans nécessité d'engagement, leur volonté à payer pour ces formations. La demande étant non négligeable, le processus a été formalisé à partir de mai 2015 par des bons de commande, où les titulaires et gestionnaires de CDS ont formellement exprimé leur demande pour ces formations, ainsi que leur volonté à payer pour celles-ci, selon un processus d'enchère de Vickrey. Une fois tous les bons de commande collectés, l'équipe d'évaluation devra identifier les CDS qui ont manifesté le plus d'intérêt, et organiser les formations.

7.2 A l'égard des parties prenantes dans la mise en œuvre de l'intervention

Il est supposé que l'introduction du FBP Nutrition au Burundi va permettre d'améliorer la performance des AS en matière de prévention et de prise en charge de la malnutrition, à travers les centres de santé du pays. Un certain nombre de problèmes sont néanmoins à résoudre pour garantir son efficacité.

Disponibilité des intrants

A ce jour, on note que les intrants diététiques dans le SSN ne sont pas disponibles dans la plupart des provinces, ce qui rend *de facto* les services SSN non effectifs. En effet, depuis 2014, le PAM n'approvisionne plus 15 provinces sur 17 ; en conséquence, une grande partie des SSN ont fermé et la plupart sont non fonctionnels. Pour pallier ce problème, le PRONIANUT s'est engagé à identifier des fournisseurs de farine, chez qui les CDS avec SSN pourront se fournir officiellement. Il est important et urgent que cela soit rendu effectif, car, sans cela, le FBP Nutrition ne pourra pas être efficace en ce qui concerne la prise en charge de la MAM dans le SSN.

La disponibilité des intrants concerne également les intrants diététiques dans le STA et les intrants liés au traitement systématique, à savoir : les antibiotiques, les antipaludéens, les antiparasitaires, le vaccin anti-rougeoleux, l'acide folique et la vitamine A. On a noté des ruptures de stock en intrants diététiques (Plumpy Nut) dans 55% des CDS ainsi que des kits de produits de traitement systématique incomplets dans 76% des cas. Or il est à nouveau urgent et important de garantir la possibilité continue pour les CDS de s'approvisionner en intrants pour que l'intervention FBP Nutrition soit efficace.

Formations des agents de santé

Comme dit plus haut, les formations au nouveau protocole de prise en charge de la malnutrition aigüe (MSPLS, OMS, UNICEF, 2014) sont seulement en cours : elles ont été réalisées pour le SST, sont en cours de réalisation pour le STA, et n'ont pas démarré pour le SSN, qui est, faute d'intrants, jugé inexistant. Il est urgent que ces formations soient réalisées, sans quoi la réussite de l'intervention FBP Nutrition est compromise – compte tenu par ailleurs de la faible qualité des services de santé en matière de prévention et prise en charge de la malnutrition relevée dans ce rapport.

Il est par ailleurs estimé comme essentiel pour la bonne réussite de l'intervention que les AS soient également formés au suivi et à la promotion de la croissance, à la prise et interprétation de mesures anthropométriques, ainsi qu'aux conseils d'hygiène et diététiques nécessaires à un bon état nutritionnel.

Achever la mise en œuvre de l'intervention FBP Nutrition

Certains retards ont été observés conernant la mise en œuvre même de l'intervention. Notamment, le volet communautaire de l'intervention, censé démarrer en juillet 2015 n'a finalement été mis en œuvre qu'en octobre 2015. De même, dans le groupe d'intervention, les contrats secondaires entre les CDS nutritionnels (dits « primaires ») et les CDS non nutritionnels qui réfèrent vers les premiers les cas de MA dépistés, permettant, avec le volet communautaire, de compléter la chaîne des agents concernés par la MA, sont seulement en train d'être mis en place. La mise en œuvre effective de ces éléments doit être suivie et corrigée le cas échéant.

Par ailleurs, le volet qualitatif de l'intervention, quoique évalué depuis juillet 2015 pour le deuxième trimestre[17], n'est pas encore pris en compte dans le calcul des subsides versés aux CDS du groupe d'intervention, en raison de malus trop élevés pour l'ensemble des CDS. Si la raison invoquée est compréhensible, il est néanmoins important de communiquer aux CDS du groupe d'intervention leurs scores qualité ainsi que de les inciter à mieux faire les prochains mois en leur assurant que la qualité sera cette fois prise en compte pour le calcul des subsides.

7.3 A l'égard du MSPLS de manière plus générale

D'une manière générale, un certain nombre de problèmes à résoudre et recommandations sont à envisager, tant pour la réussite de l'intervention que pour la qualité du système de santé en général.

Amélioration de la performance des agents de santé

1. Mener des recherches qualitatives pour documenter les attitudes et opinions des AS

Des entretiens individuels approfondis et des discussions en groupes pourraient être utilisés pour comprendre les défis et besoins des AS au regard de leurs activités quotidiennes dans les centres de santé, et cela dans l'optique d'essayer de trouver les causes profondes de la faible performance observée au cours de l'enquête.

2. Davantage de formations, plus adaptées et de meilleure qualité

Non seulement il a été constaté que plusieurs AS n'avaient pas été formés récemment dans le domaine de la nutrition (et aussi dans les soins pédiatriques de base) ; mais les AS eux-même ont également identifié des domaines pour lesquels ils souhaiteraient davantage de formation. Le défi serait ici, si possible, de permettre aux AS de participer à des formations personnalisées par rapport à leurs besoins, ou au moins par rapport aux besoins du centre de santé; plutôt que des formations génériques sur la malnutrition ou la PCIME. Par exemple si les résultats des vignettes montrent que les AS d'un certain centre de santé ont une performance particulièrement faible sur les anamnèses et examens physiques, alors une formation ciblée sur l'évaluation clinique de base pourrait être de plus grande valeur qu'une formation plus spécialisée dans la prise en charge des cas de malnutrition aigüe sévère avec complications par exemple.

3. Disponibilité et application des protocoles

Une récente revue (Rowe et al 2005) a mis en évidence qu'un problème important dans l'application des protocoles peut tout simplement être la non disponibilité de ceux-ci dans leur forme physique au niveau des centres de santé. Cette situation est apparue comme fréquente au Burundi ; aussi, une recommandation forte serait de fournir à tous les centres de santé des copies des protocoles nécessaires (surtout qu'un nouveau protocole national pour la prise en charge de la malnutrition est disponible depuis octobre 2014), et, si nécessaire, de mettre en place dans chaque CDS un système sécurisé (même simplement une armoire fermée à clé) ou une petite bibliothèque pour s'assurer que les AS peuvent avoir à portée de mains ces protocoles durant leurs consultations. Ceci constituerait un

[17] La qualité du premier trimestre n'a en effet pas été évaluée.

premier pas ; qui nécessiterait d'être complété par une supervision pour s'assurer de l'usage de ces protocoles.

4. Améliorer l'efficacité de la supervision

Les résultats des questionnaires V et N montrent qu'il y a un manque de supervision des AS de la part des autorités sanitaires. Comme mentionné ci-dessus, de récentes études ont montré que la supervision et l'audit peuvent avoir un impact important sur l'amélioration de la performance des AS (Rowe et al 2005; Grimshaw et al.2004) ; plus que la formation seule. Une forte recommandation serait alors d'améliorer la formation des superviseurs, à différents niveaux, pour s'assurer que leurs visites de supervision sont efficaces et productives sur le côté théorique, mais aussi pour que, côté logistique, leurs visites programmées sont réalistes en termes de temps et de contexte géographique. Concrètement, il s'agit pour les superviseurs de prévoir suffisamment de temps dans chaque centre et d'avoir déjà une connaissance de base sur les besoins des AS. Cela semble simple mais c'est souvent négligé (Rowe et al. 2005). Ainsi, plutôt que de se contenter de passer en revue les registres, les superviseurs pourraient prendre le temps de discuter avec les AS des gestes posés sur les patients. Pour cela, un renforcement des compétences des superviseurs sur la prise en charge (anamnèse, examens, diagnostic, traitement et conseils) est potentiellement également nécessaire.

5. Créer des Centres d'Excellence et Organisation des stages

Un certain nombre d'AS ont signalé des lacunes dans leurs connaissances dans des domaines spécifiques, par exemple dans la prise en charge des enfants avec le VIH. En plus de formations et de supervisions améliorées sur ces sujets, il est suggéré de mettre en place un système où les AS iraient faire un «stage» dans une FOSA, identifiée comme ayant un grand nombre de ces cas spécifiques ou ayant un personnel expérimenté dans de tels soins, afin qu'ils puissent apprendre de manière pratique. Ces centres pourraient recevoir un soutien externe pour devenir, par exemples, le centre d'excellence pour la gestion des enfants séropositifs dans la santé primaire ou celui pour la gestion des enfants MAS avec la tuberculose, ou celui d'autres éléments spécialisés de soins.

En particulier, cela serait utile pour les AS travaillant dans des centres de santé qui n'ont pas beaucoup de cas de malnutrition aigüe, d'aller dans des centres de santé qui connaissent une plus grande incidence de malnutrition aigüe, et donc plus spécialisés, afin d'apprendre davantage sur le dépistage et la prise en charge de la malnutrition aigüe. Aussi, il serait bénéfique pour les AS de faire des stages sur une base régulière dans des services de stabilisation (SST) afin de devenir plus familiers avec la prise en charge des complications à l'hôpital mais aussi avec l'identification des complications au niveau du CDS. Cela pourrait aussi aider dans la communication avec les responsables des enfants, d'autant plus qu'ils pourraient leur expliquer, avant de les référer vers le SST, quels soins ils vont recevoir comparativement à ceux dispensés par le centre. Il se peut que des AS aient fait des rotations dans ces services, mais si cette pratique n'est pas régulière, elle peut être oubliée et les AS peuvent perdre confiance dans leurs compétences professionnelles. Cela est d'autant plus important au regard de l'existence d'un nouveau protocole national, depuis 2014, qui a introduit des changements dans la prise en charge hospitalière (critères d'admission et de décharge, utilisation de ReSoMal, etc.).

On pourrait également envisager que des CDS « excellant » dans les consultations curatives en général soient identifiés comme tels, par les superviseurs formés, au sein de chacun des districts, permettant aux AS de chacun des autres CDS du district de se former à moindre coût.

Amélioration du fonctionnement quotidien des services de pédiatrie et de nutrition

Les efforts pour améliorer le fonctionnement quotidien des services pédiatriques et de la malnutrition semblent essentiels pour garantir l'efficacité de l'intervention FBP Nutrition. Par ailleurs, ils pourraient avoir également un impact positif sur la performance des AS (cela doit être vérifié par des recherches qualitatives sur les défis et les besoins des AS). Les recommandations seraient les suivantes.

1. Disponibilité et fonctionnalité des équipements

De par les résultats du questionnaire N, il paraît évident que les CDS manquent de nombreuses pièces essentielles de matériel ou de ressources pour évaluer l'état nutritionnel des enfants. Des arguments supplémentaires viennent du questionnaire S qui a montré des différences significatives dans les poids pris par les enquêteurs avec les balances des CDS comparativement à ceux pris avec des balances neuves utilisées pour l'enquête. Les fonds des CDS, provenant non seulement des allocations FBP mais également d'autres sources, devraient être en priorité alloués à l'amélioration de la qualité de l'équipement et à l'assurance de sa sécurité et de son entretien dans les centres.

2. Organisation et gestion des données patients dans les services de prise en charge de la malnutrition

Les résultats des questionnaires SSN, STA et N ont montré un clair besoin d'amélioration dans la gestion des données patient et dans l'organisation des services de la malnutrition. Il pourrait y avoir un certain nombre de solutions simples à cela, notamment améliorer le système de stockage des fiches du patient. Aussi, plutôt que d'avoir un seul membre du personnel affecté à cette tâche, il pourrait y avoir une petite équipe qui pourrait maintenir la continuité de la gestion de la documentation, même avec des changements de personnel ou des absences. Une autre option serait de garder les originaux des carnets de santé – ou bien des copies – dans les CDS plutôt que de les laisser aux accompagnants des enfants ; mais cela constituerait un changement fondamental dans le fonctionnement des services de santé primaire. Les centres de santé dépendent également des approvisionnements en fiches vierges, souvent réalisés par le niveau central, ce qui signifie qu'il y aurait besoin de l'appui du district sanitaire pour cela.

7.4 A l'égard des commanditaires de l'étude - MSPLS et Banque Mondiale

Préparation des enquêtes de suivi

Afin d'optimiser la qualité des enquêtes de suivi et d'éviter des retards dans les activités de formation mais surtout de collecte des données, il est ici rappelé de bien inscrire ces enquêtes dans l'agenda des enquêtes à réaliser pour le MSPLS et la Banque Mondiale, ainsi que d'effectuer à temps les contractualisations avec les instituts pressentis pour réaliser les enquêtes (INSP et ISTEEBU). Ceci devra faire l'objet d'une collaboration rapprochée avec l'équipe d'évaluation.

Recommandations majeures

Les résultats de ce rapport indiquent clairement les défis que le système de santé burundais doit relever. Les défis demeurent importants si on se limite au cadre de cette étude. En effet, pour que, à la

fin de ce projet d'évaluation, l'intervention FBP Nutrition ait un impact positif et significatif sur les taux de guérison de MA et sur la prévalence de MA dans la communauté, un certain nombre d'obstacles sont à lever rapidement. Il s'agit principalement du problème de la disponibilité des intrants nutritionnels du SSN, et de l'amélioration de la performance des AS par, au minimum, des formations à la prévention et à la prise en charge de la malnutrition ainsi qu'au suivi et promotion de la croissance (pour une liste plus complète des défis à relever, cf. ci-dessus les recommandations à l'égard des parties prenantes dans la mise en œuvre de l'intervention ainsi qu'à l'égard du MSPLS plus généralement).

Le problème de disponibilité des intrants n'est à ce jour pas encore résolu : des fournisseurs de farine doivent encore être identifiés avant de devenir les fournisseurs officiels de farine pour les SSN. La possibilité pour les CDS, d'acheter des intrants nutritionnels pour les SSN, est prévue d'être effective dès novembre 2015 (ce qui reste à confirmer). La formation des AS au nouveau protocole de prévention et de prise en charge de la malnutrition aigüe a été réalisée courant 2015 pour le SST, est en cours de réalisation pour le STA, et n'est pas encore démarrée pour le SSN – jugé inexistant tant qu'il n'y aura pas accès à des intrants nutritionnels. Ces éléments, tant qu'ils n'auront pas été résolus, rendront l'intervention FBP Nutrition inefficace.

Par ailleurs, si, comme cela est prévu, les enquêtes de suivi ont lieu en juillet 2016, soit 18 mois après le démarrage officiel de l'intervention, la collecte de données relatives au taux de guérison de MAM et de MAS concerne la période des six mois précédents. En effet, les fiches individuelles de suivi SSN et STA sélectionnées pour la retranscription concernent cette période, soit de janvier à juin 2016. Aussi, cela signifie que, si tant est que les intrants deviennent effectivement disponibles à partir de novembre 2015 et que les formations au nouveau protocole se terminent avant fin 2015, la durée d'exposition n'aura pas été de 18 mois mais de 1 à 6 mois selon les observations. Par exemple, pour les fiches SSN datées de janvier 2015, la durée d'exposition à un SSN effectif aura été d'à peine 2 mois (sans mentionner que la formation des AS au SSN n'aura probablement pas encore eu lieu à ce moment). Ceci présage de peu ou pas d'efficacité de l'intervention au niveau des CDS. Au niveau communautaire, ces inquiétudes demeurent tant que les problèmes ne sont pas résolus au niveau des CDS mais aussi étant donné que le volet communautaire de l'intervention n'a été mis en place qu'en octobre 2015.

Compte tenu de ces risques importants, il est proposé aux commanditaires de l'étude d'envisager de prolonger la durée d'exposition à l'intervention. D'un point de vue budgétaire du côté de la mise en oeuvre, cela semble possible étant donné que, jusque-là, le taux de consommation du budget dédié à l'intervention reste autour de 10%. Parallèlement, il est suggéré de continuer à soutenir les parties prenantes de la mise en œuvre de l'intervention, à assurer un suivi et évaluation de la mise en œuvre effective ainsi que des défis à relever.

8. Références bibliographiques

Ashworth, A, and E Ferguson. 2009. “Dietary Counselling in the Management of Moderate Malnourishment in Children.” *Food and Nutrition Bulletin* (supplement) 30 (3): S405–33.

Basenya, O, M Nimpagaritse, F Busogoro, JNdayishimiye, C Nkunzimana, G Ntahimpereye, MBossuyt, J Ndereye, and L Ntakarutimana. 2011. “Le Financement Basé Sur La Performance Comme Stratégie Pour Améliorer La Mise En Oeuvre de La Gratuité Des Soins: Premières Leçons de L’expérience Du Burundi.” WP 5. *PBF CoP Working Paper*.

Bhutta, Z, and C Salam. 2012. “Global Nutrition Epidemiology and Trends.” *Ann Nutr Metab*, 61 (Suppl 1): 19–27.

Brent, A, R Davidson, and A Seale. 2014. *Oxford Handbook of Tropical Medicine*. Fourth Edition. OUP.

Coates, J, A Swindale, and P Bilinsky. 2007. “Household Food Insecurity Access Scale (HFIAS) for Measurement of Food Access: Indicator Guide.” Washington, DC: Food and Nutrition: Version 3.

EDS. 2010. “Enquête Démographique et de Santé Burundi 2010.”

Edward, A, B Kumar, F Kakar, AS Salehi, G Burnham, and others 2011. “Configuring Balanced Scorecards for Measuring Health System Performance: Evidence from 5 Years’ Evaluation in Afghanistan.” *PLoS Med* 8 (7).

Grimshaw, JM, RE Thomas, and others. 2004. “Effectiveness and Efficiency of Guideline Dissemination and Implementation Strategies.” *Health Technol Assess* 8 (6).

Hayes, R J, and S Bennett. 1999. “Simple Sample Size Calculation for Cluster-Randomized Trials.” *International Journal of Epidemiology* 28 (2): 319–26.

INSP. 2014. “Rapport de La Collecte Des Données Sur Terrain - Enquête de Base Auprès Des Centres de Santé Dans Le Cadre de L’ « Evaluation D'impact de L'introduction de La Nutrition Dans Le Programme PBF Au Burundi ».” Bujumbura, Burundi.

ISTEEBU. 2015a. “Rapport de Terrain de L’enquête PBF Du 07 Décembre 2014 Au 10 Janvier 2015.” Bujumbura, Burundi.

———. 2015b. “Enquête FBP Nutrition - Rapport de Gestion Des Données.” Bujumbura, Burundi.

Kaiser, R, BA Woodruff, O Bilukha, PB Spiegel, and P Salama. 2006. “Using Design Effects From Previous Cluster Surveys to Guide Sample Size Calculation in Emergency Settings.” *Disasters* 30 (2): 199–211.

Kismul, H., and others 2015. “The Social Context of Severe Child Malnutrition: A Qualitative Household Case Study from a Rural Area of the Democratic Republic of Congo.” *Int J Equity Health* 14 (1).

Manary, MJ, and DR Brewster. 2000. “Intensive Nursing of Children with Kwashiorkor in Malawi.” Acta Paediatr 89: 203±7.

Meessen, B, D Hercot, M Noirhomme, V Ridde, A Tibouti, C Kirunga Tashobya, and L Gilson. 2011. “Removing User Fees in the Health Sector: A Review of Policy Processes in Six Sub-Saharan African Countries.” *Health Policy and Planning* 26 Suppl 2 (suppl_2): ii16–29.

MSPLS. 2010. “Protocole National de Prise En Charge Intégrée de La Malnutrition Aigüe Globale.”

———. 2011. “Rapport de Mise En Œuvre Du FBP et de La Gratuité Des Soins Pour L’année 2011.”

———. 2013. “Note Technique Relative À L’intégration de La Nutrition Dans La Stratégie Nationale de Financement Basé Sur La Performance.”

———. 2014. “Enquete Ménages Pour Le Suivi et L’évaluation de L'impact de L'appui Au Système de Remboursement Du Paquet Minimum Des Services de Santé - Édition 2012,”.

———, OMS, UNICEF. 2014. “ Protocole National de Prise En Charge Intégrée de La Malnutrition Aigüe”.

Nagahori, C., and others. 2015. “Factors Associated with Nutritional Status in Children Aged 5-24 Months in the Republic of Cameroon.” *Nurs Health Sci*. 17 (2): 229–35.

Nikiema, L., and others. 2014. “Treating Moderate Acute Malnutrition in First-Line Health Services: An Effectiveness Cluster-Randomized Trial in Burkina Faso.” *Am J Clin Nutr*. 100 (1): 241–49.

Nimpagaritse, M, and MP Bertone. 2011. “The Sudden Removal of User Fees: The Perspective of a Frontline Manager in Burundi.” *Health Policy and Planning* 26 Suppl 2 (suppl_2): ii63–71.

Ntakarutimana, L, and M Nimpagaritse. 2013. “Etat Des Lieux de La Prise Charge de La Malnutrition Dans Les Formations Sanitaires Au Burundi : Résultats D’une Enquête Effectuée Dans 24 FOSA Du 20 Au 28 Mai 2013.”

Oladele, EA, L Ormond, O Adeyemi, D Patrick, F Okoh, OB Oresanya, and JJ. Valadez. 2012. “Tracking the Quality of Care for Sick Children Using Lot Quality Assurance Sampling: Targeting Improvements of Health Services in Jigawa, Nigeria.” *PloS One* 7 (9).

Parker, M, JL Leroy, D Olney, J Harris, and MT. Ruel. 2014. “Strengthening and Evaluating the Preventing Malnutrition in Children under 2 Approach (PM2A) in Burundi: Baseline Report,” no. May: 1–131.

Rowe, AK, and others. 2005. “How Can We Achieve and Maintain High-Quality Performance of Health Workers in Low-Resource Settings?” *Lancet* 366: 1026–35.

Singh, K et others. 2014. “Management of Children with Severe Acute Malnutrition: Experience of Nutrition Rehabilitation Centers in Uttar Pradesh, India.” *Indian Paediatrics* 51: 21–25.

UNDP. 2010. “Rapport Burundi 2010 - Objectifs Du Millénaire Pour Le Développement.”

Valadez, JJ, LD Brown, WV Vargas, and D Morley. 1996. “Using Lot Quality Assurance Sampling to Assess Measurements for Growth Monitoring in a Developing Country’s Primary Health Care System.” *Int J Epidemiol* 25 (2): 381–87.

Van de Poel, E., and others. 2008. “Socioeconomic Inequality in Malnutrition in Developing Countries.” *Bulletin of the World Health Organization* 86: 282–91.

WFP. 2008. “Comprehensive Food Security and Vulnerability Analysis, Burundi.”

WFP. 2014. “Analyse Globale de La Sécurité Alimentaire, de La Nutrition et de La Vulnérabilité Au Burundi.”

WHO. 2010a. “Indicators for Assessing Infant and Young Child Feeding Practices. Part 3: Country Profiles.” Geneva.

———. 2010b. “Indicators for Assessing Infant and Young Child Feeding Practices - Part 2: Measurement.”

———. 2013a. “Pocket Book of Hospital Care for Children: Guidelines for the Management of Common Childhood Illnesses. Second Edition.”

———. 2013b. “Updates on the Management of Severe Acute Malnutrition in Infants and Children.”

———. 2014. “Integrated Management of Childhood Illness: Chart Booklet.”

World Bank. 2015. "World Development Indicators." http://data.worldbank.org/data-catalog/world-development-indicators.

9. Annexe. Comparaison des principales statistiques parmi les groupes de contrôle et d'intervention

Tableau 83. Comparabilité des centres de santé des groupes de contrôle et d'intervention en terme de gestion (questionnaire G)

	All groups			Treatment group			Control group			Treatment - Control	
	Obs	Mean	SD	Obs	Mean	SD	Obs	Mean	SD	diff	tstat
**Propriétaire = gouvernement**	90	83.3%	0.04	45	80.0%	0.06	45	86.7%	0.05	-6.7%	-0.84
**Propriétaire = mission religieuse**	90	16.7%	0.04	45	20.0%	0.06	45	13.3%	0.05	6.7%	0.84
**Le CDS dispose d'un téléphone**	90	85.6%	0.04	45	88.9%	0.05	45	82.2%	0.06	6.7%	0.89
**NB d'ASC**	86	23.7	1.76	42	21.9	1.85	44	25.5	2.95	-3.6	-1.03
**Le CDS dispose d'un comité de gestion**	90	94.4%	0.02	45	91.1%	0.04	45	97.8%	0.02	-6.7%	-1.38
**Le CDS dispose d'un comité de santé**	90	97.8%	0.02	45	97.8%	0.02	45	97.8%	0.02	0.0%	0.00
**L'organigramme de la structure est présent et à jour dans le CDS**	90	43.3%	0.05	45	48.9%	0.08	45	37.8%	0.07	11.1%	1.06
**Le registre des présences est présent et à jour dans le CDS**	90	85.6%	0.04	45	86.7%	0.05	45	84.4%	0.05	2.2%	0.30
**Le registre de garde est présent et à jour dans le CDS**	90	57.8%	0.05	45	53.3%	0.08	45	62.2%	0.07	-8.9%	-0.85
**Nb de staff dans le CDS**	90	15.5	0.63	45	14.2	0.60	45	16.8	1.08	-2.6	-2.11
**% hommes dans le staff du CDS**	90	60%	0.02	45	60%	0.02	45	60%	0.02	0%	-0.08
**Nb de PTF**	90	3.14	0.28	45	2.80	0.37	45	3.49	0.43	-0.69	-1.22

Tableau 84. Comparabilité des services de supplementation nutritionnelle (SSN) des groups de contrôle et d'intervention

SSN service (a)	All groups			Treatment group			Control group			Treatment - Control	
**Information manquante sur**	Obs	Mean	SD	Obs	Mean	SD	Obs	Mean	SD	diff	tstat
**D'où le cas était référé**	971	83.1%	0.04	487	80.5%	0.06	484	85.7%	0.05	-5.3%	-0.73
**Si le cas avait déjà été traité pour MAM**	971	67.7%	0.05	487	66.3%	0.07	484	69.0%	0.06	-2.7%	-0.30
**Poids à l'admission**	971	1.1%	0.00	487	1.6%	0.01	484	0.6%	0.00	1.0%	1.27
**Taille à l'admission**	971	3.6%	0.01	487	5.3%	0.02	484	1.9%	0.01	3.5%	1.49
**PB à l'admission**	971	17.6%	0.03	487	15.8%	0.05	484	19.4%	0.05	-3.6%	-0.53
**Oedeme à l'admission**	971	59.4%	0.05	487	59.1%	0.07	484	59.7%	0.07	-0.6%	-0.06
**Raison de sortie**	971	35.3%	0.04	487	34.3%	0.06	484	36.4%	0.06	-2.1%	-0.24
**Poids à la sortie**	971	9.8%	0.02	487	10.7%	0.03	484	8.9%	0.04	1.8%	0.38
**Taille à la sortie**	971	57.8%	0.04	487	55.9%	0.06	484	59.7%	0.05	-3.9%	-0.47
**PB à la sortie**	971	36.8%	0.04	487	36.6%	0.06	484	37.0%	0.06	-0.4%	-0.05
**Oedeme à la sortie**	971	67.5%	0.05	487	66.9%	0.07	484	68.0%	0.06	-1.0%	-0.11

SSN service (b)	All groups			Treatment group			Control group			T-C	
	Obs	Mean	SD	Obs	Mean	SD	Obs	Mean	SD	diff	tstat
**age en mois**	951	24.76	0.69	478	24.76	0.99	473	24.77	0.98	-0.01	0.00
**sexe (1=male, 2=female)**	942	1.52	0.01	473	1.50	0.02	469	1.54	0.02	-0.04	-1.35
**WHZ à l'entrée (quand poids et taille renseignés)**	903	-2.06	0.07	442	-2.23	0.09	461	-1.90	0.11	-0.32	-2.30
**HAZ à l'entrée (quand taille et age renseignés)**	894	-3.39	0.10	440	-3.49	0.12	454	-3.29	0.15	-0.19	-1.02
**PB à l'entrée en mm (quand renseigné)**	800	119.33	0.61	410	119.03	0.91	390	119.65	0.82	-0.62	-0.51
**Oedeme à l'entrée : aucun (quand renseigné)**	394	96.2%	0.01	199	97.5%	0.01	195	94.9%	0.02	2.6%	0.94
**Oedeme à l'entrée : + (quand renseigné)**	394	1.8%	0.01	199	1.5%	0.01	195	2.1%	0.01	-0.5%	-0.31
**Oedeme à l'entrée : ++ (quand renseigné)**	394	1.0%	0.01	199	1.0%	0.01	195	1.0%	0.01	0.0%	-0.02
**Oedeme à l'entrée : +++ (quand renseigné)**	394	1.0%	0.01	199	0.0%	0.00	195	2.1%	0.02	-2.1%	-1.25
**WHZ à la sortie (quand poids et taille renseignés)**	400	-1.33	0.10	210	-1.39	0.14	190	-1.27	0.12	-0.12	-0.61
**HAZ à la sortie (quand taille et age renseignés)**	391	-3.07	0.20	205	-3.05	0.31	186	-3.08	0.26	0.03	0.07
**Raison sortie: transfert SSt**	628	0.8%	0.00	320	0.3%	0.00	308	1.3%	0.01	-1.0%	-1.18
**Raison sortie: transfert STA**	628	10.5%	0.03	320	6.3%	0.02	308	14.9%	0.05	-8.7%	-1.50
**Raison sortie: guérison**	628	79.9%	0.03	320	83.8%	0.03	308	76.0%	0.06	7.8%	1.17
**Raison sortie: abandon**	628	4.6%	0.01	320	4.4%	0.01	308	4.9%	0.03	-0.5%	-0.17
**Raison sortie: décès**	628	0.8%	0.00	320	0.9%	0.01	308	0.6%	0.00	0.3%	0.36
**Raison sortie: non répondant**	628	3.2%	0.01	320	4.1%	0.02	308	2.3%	0.01	1.8%	0.80

SSN service (c)	All groups			Treatment group			Control group			T-C	
	Obs	Mean	SD	Obs	Mean	SD	Obs	Mean	SD	diff	tstat
**Aucun traitement reçu**	971	64.7%	0.04	487	64.3%	0.06	484	65.1%	0.06	-0.8%	-0.09
**Traitement d'al/me bendazole reçu**	971	19.3%	0.03	487	19.9%	0.05	484	18.6%	0.05	1.3%	0.20
**Traitement de vitamine A reçu**	971	9.8%	0.02	487	6.6%	0.03	484	13.0%	0.04	-6.4%	-1.31
**Traitement d'acide folique reçu**	971	8.4%	0.02	487	4.1%	0.02	484	12.8%	0.04	-8.7%	-1.91
**Nb de visites sans nourriture % Nb de visites**	971	33.5%	0.04	487	39.8%	0.06	484	27.2%	0.05	12.6%	1.58
**gain de poids entre l'entrée et la sortie en grammes/poids inital en kg/jour de traitement (pour les déclarés guéris seulement)**	437	1.74	0.22	227	2.06	0.30	210	1.39	0.32	0.67	1.54
**durée du traitement en jours entre l'entrée et la sortie (pour les déclarés guéris seulement)**	477	74.64	5.75	252	78.08	8.67	225	70.79	7.41	7.29	0.64

Tableau 85. Comparabilité des services de thérapeutique ambulatoire (STA) dans les groups de contrôle et d'intervention

STA service (a)	All groups			Treatment group			Control group			T-C	
**Information manquante sur**	Obs	Mean	SD	Obs	Mean	SD	Obs	Mean	SD	diff	tstat
**D'où le cas était référé**	963	79.2%	0.04	485	78.4%	0.06	478	80.1%	0.06	-1.8%	-0.22
**le cas avait déjà été traité pour SAM**	963	60.5%	0.05	485	61.0%	0.07	478	60.0%	0.07	1.0%	0.10
**Poids à l'admission**	963	0.7%	0.00	485	0.8%	0.00	478	0.6%	0.00	0.2%	0.37
**Taille à l'admission**	963	3.9%	0.02	485	6.4%	0.03	478	1.5%	0.01	4.9%	1.59
**PB à l'admission**	963	13.1%	0.03	485	13.6%	0.04	478	12.6%	0.04	1.1%	0.19
**Oedeme à l'admission**	963	34.4%	0.04	485	30.3%	0.05	478	38.5%	0.06	-8.2%	-1.06
**Test d'appétit réalisé**	963	41.0%	0.05	485	44.3%	0.07	478	37.7%	0.07	6.7%	0.68
**Traitement systématique reçu**	963	29.5%	0.04	485	29.5%	0.06	478	29.5%	0.07	0.0%	0.00
**Vaccin rougeole reçu**	963	72.7%	0.04	485	78.8%	0.06	478	66.5%	0.07	12.2%	1.43
**Traitement palu reçu**	963	75.1%	0.04	485	81.2%	0.06	478	68.8%	0.06	12.4%	1.48
**Raison de sortie**	963	30.9%	0.04	485	35.5%	0.06	478	26.4%	0.05	9.1%	1.12
**WHZ à la sortie (si poids et taille renseignés)**	963	6.1%	0.02	485	4.5%	0.01	478	7.7%	0.03	-3.2%	-0.99
**HAZ à la sortie (si âge et taille renseignés)**	963	55.2%	0.04	485	57.5%	0.05	478	52.9%	0.06	4.6%	0.59
**PB à la sortie**	963	31.5%	0.04	485	33.2%	0.06	478	29.7%	0.05	3.5%	0.45
**Odeme à la sortie**	963	47.7%	0.04	485	43.9%	0.06	478	51.5%	0.07	-7.5%	-0.85

STA service (b)	All groups			Treatment group			Control group			T-C	
	Obs	Mean	SD	Obs	Mean	SD	Obs	Mean	SD	diff	tstat
**age en mois**	949	27.05	0.77	478	27.12	1.08	471	26.98	1.12	0.14	0.09
**sexe (1=male, 2=female)**	918	1.45	0.02	457	1.42	0.02	461	1.48	0.02	-0.06	-1.78
**Weight for length/height z-score à l'entrée (quand poids et taille renseignés)**	869	-2.45	0.08	421	-2.52	0.13	448	-2.37	0.11	-0.15	-0.93
**Length/height for age z-score à l'entrée (quand taille et age renseignés)**	875	-3.65	0.10	423	-3.69	0.14	452	-3.61	0.15	-0.07	-0.34
**PB à l'entrée en mm (quand renseigné)**	837	115.40	0.75	419	115.89	1.10	418	114.90	1.04	0.99	0.65
**Oedeme à l'entrée : aucun (quand renseigné)**	632	52.7%	0.03	338	53.0%	0.05	294	52.4%	0.05	0.6%	0.09
**Oedeme à l'entrée : + (quand renseigné)**	632	15.7%	0.02	338	13.3%	0.03	294	18.4%	0.04	-5.1%	-1.13
**Oedeme à l'entrée : ++ (quand renseigné)**	632	20.9%	0.03	338	22.8%	0.03	294	18.7%	0.04	4.1%	0.81
**Oedeme à l'entrée : +++ (quand renseigné)**	632	10.8%	0.02	338	10.9%	0.03	294	10.5%	0.03	0.4%	0.10
**Test d'appétit à l'entrée: bon**	568	83.1%	0.04	270	78.9%	0.06	298	86.9%	0.04	-8.0%	-1.09
**Test d'appétit à l'entrée: moyen**	568	16.5%	0.04	270	20.4%	0.06	298	13.1%	0.04	7.3%	0.99
**Test d'appétit à l'entrée: refus**	568	0.4%	0.00	270	0.7%	0.01	298	0.0%	0.00	0.7%	1.45
**Weight for length/height z-score à la sortie (quand poids et taille renseignés)**	408	-1.26	0.12	187	-1.60	0.18	221	-0.97	0.13	-0.63	-2.77
**Length/height for age z-score à la sortie (quand taille et age renseignés)**	413	-3.18	0.16	192	-3.07	0.27	221	-3.28	0.20	0.21	0.62
**Raison sortie: transfert SSt**	665	2.0%	0.01	313	1.9%	0.01	352	2.0%	0.01	-0.1%	-0.05
**Raison sortie: guérison**	665	85.4%	0.02	313	83.7%	0.03	352	86.9%	0.02	-3.2%	-0.87
**Raison sortie: abandon**	665	5.1%	0.01	313	4.2%	0.01	352	6.0%	0.02	-1.8%	-0.76
**Raison sortie: décès**	665	1.4%	0.00	313	1.6%	0.01	352	1.1%	0.01	0.5%	0.51
**Raison sortie: non répondant**	665	5.1%	0.01	313	7.0%	0.02	352	3.4%	0.01	3.6%	1.39

STA service (c)	All groups			Treatment group			Control group			Treatment - Control	
	Obs	Mean	SD	Obs	Mean	SD	Obs	Mean	SD	diff	tstat
**Traitement d'amoxycilline reçu**	679	47.6%	0.05	342	47.7%	0.07	337	47.5%	0.08	0.2%	0.02
**Traitement de vitamine A reçu**	679	37.8%	0.05	342	41.5%	0.07	337	34.1%	0.07	7.4%	0.73
**Traitement d'acide folique reçu**	679	32.5%	0.05	342	33.9%	0.07	337	31.2%	0.07	2.8%	0.30
**Traitement d'al/me bendazole reçu**	679	64.7%	0.05	342	71.1%	0.06	337	58.2%	0.07	12.9%	1.39
**Traitement d'amoxycilline ET d'al/mebendazole reçu**	679	44.3%	0.05	342	45.0%	0.07	337	43.6%	0.07	1.4%	0.14
**Nb de visites sans Plumpy Nut % Nb de visites**	960	21.3%	0.03	484	16.6%	0.04	476	26.0%	0.05	-9.5%	-1.54
**Nb de visites sans Plumpy Nut % (Nb de visites-1)**	894	23.2%	0.03	453	18.1%	0.04	441	28.4%	0.06	-10.3%	-1.53
**gain de poids entre l'entrée et la sortie en grammes/poids inital en kg/jour de traitement (pour les déclarés guéris seulement)**	536	3.92	0.33	247	3.68	0.47	289	4.13	0.48	-0.45	-0.68
**durée du traitement en jours entre l'entrée et la sortie (pour les déclarés guéris seulement)**	556	58.89	3.19	252	61.19	3.97	304	56.97	4.87	4.22	0.68

Tableau 86. Comparabilité des centres de santé des groups de contrôle et d'intervention en termes de qualité des consultations (quest. C)

Questionnaire C (a)	All groups			Treatment group			Control group			Treatment - Control	
	Obs	Mean	SD	Obs	Mean	SD	Obs	Mean	SD	diff	tstat
**Aucun signe danger demandé (sur 3 signes de danger selon la PCIME)**	514	62.3%	0.03	254	61.4%	0.04	260	63.1%	0.04	-1.7%	-0.27
**1 signe danger demandé (sur 3)**	514	31.7%	0.03	254	31.9%	0.04	260	31.5%	0.04	0.4%	0.07
**2 signes danger demandés (sur 3)**	514	5.6%	0.01	254	5.9%	0.02	260	5.4%	0.02	0.5%	0.18
**3 signes danger demandés (sur 3)**	514	0.4%	0.00	254	0.8%	0.01	260	0.0%	0.00	0.8%	1.43
**Aucun des symptoms principaux demandé (sur 5 selon la PCIME)**	513	11.5%	0.02	254	13.0%	0.03	259	10.0%	0.02	3.0%	0.79
**1 symptôme principal demandé (sur 5)**	513	31.6%	0.03	254	29.5%	0.04	259	33.6%	0.03	-4.1%	-0.80
**2 symptômes principaux (sur 5)**	513	30.2%	0.02	254	28.7%	0.03	259	31.7%	0.03	-2.9%	-0.65
**3 symptômes principaux (sur 5)**	513	20.5%	0.02	254	21.7%	0.03	259	19.3%	0.03	2.3%	0.54
**4 symptômes principaux (sur 5)**	513	5.8%	0.02	254	7.1%	0.02	259	4.6%	0.02	2.5%	0.81
**5 symptômes principaux (sur 5)**	513	0.4%	0.00	254	0.0%	0.00	259	0.8%	0.01	-0.8%	-1.01
**0-4 questions posées (sur 31)**	509	33.0%	0.03	250	34.8%	0.05	259	31.3%	0.04	3.5%	0.55
**5-9 questions posées (sur 31)**	509	51.9%	0.03	250	47.2%	0.04	259	56.4%	0.04	-9.2%	-1.66
**10-14 questions posées (sur 31)**	509	13.9%	0.02	250	16.0%	0.04	259	12.0%	0.03	4.0%	0.87
**15-19 questions posées (sur 31)**	509	1.2%	0.01	250	2.0%	0.01	259	0.4%	0.00	1.6%	1.49
**0-4 examens réalisés (sur 19)**	512	73.8%	0.04	254	68.5%	0.06	258	79.1%	0.05	-10.6%	-1.34
**5-9 examens réalisés (sur 19)**	512	23.2%	0.04	254	26.0%	0.05	258	20.5%	0.05	5.4%	0.74
**10-14 examens réalisés (sur 19))**	512	2.9%	0.01	254	5.5%	0.03	258	0.4%	0.00	5.1%	1.94
**Aucun signe vital mesuré (sur 3)**	514	39.9%	0.04	254	34.6%	0.05	260	45.0%	0.05	-10.4%	-1.39
**1 signe vital mesuré (sur 3)**	514	55.1%	0.04	254	59.1%	0.05	260	51.2%	0.05	7.9%	1.13
**2 signes vitaux mesurés (sur 3)**	514	5.1%	0.01	254	6.3%	0.02	260	3.8%	0.02	2.5%	0.88
**Nb de questions les plus pertinentes (total=23)**	510	5.14	0.21	251	5.16	0.34	259	5.12	0.25	0.03	0.07

Questionnaire C (b)	All groups			Treatment group			Control group			T-C	
	Obs	Mean	SD	Obs	Mean	SD	Obs	Mean	SD	diff	tstat
**Aucune question de nutrition (pour les moins de 2 ans – sur 7)**	262	47%	0.04	125	50%	0.05	137	45%	0.06	5%	0.63
**1 question nutrition (sur 7)**	262	38%	0.04	125	35%	0.05	137	41%	0.05	-6%	-0.81
**2 questions nutrition (sur 7)**	262	10%	0.02	125	8%	0.03	137	12%	0.03	-4%	-1.11
**3 questions nutrition (sur 7)**	262	4%	0.01	125	6%	0.02	137	2%	0.01	3%	1.32
**4 questions nutrition (sur 7)**	262	1%	0.01	125	2%	0.01	137	0%	0.00	2%	1.43
**Aucune question de nutrition (pour les plus de 2 ans – sur 5)**	312	52%	0.04	164	53%	0.06	148	51%	0.06	2%	0.22
**1 question nutrition (sur 5)**	312	34%	0.03	164	30%	0.04	148	39%	0.05	-9%	-1.28
**2 questions nutrition (sur 5)**	312	11%	0.02	164	13%	0.03	148	9%	0.02	3%	0.86
**3 questions nutrition (sur 5)**	312	3%	0.01	164	4%	0.02	148	1%	0.01	4%	1.92
**Aucun examen nutrition (sur 6)**	513	41%	0.04	254	39%	0.06	259	43%	0.06	-4%	-0.52
**1 exam nutrition (sur 6)**	513	19%	0.03	254	21%	0.04	259	16%	0.03	5%	0.92
**2 exam nutrition (sur 6)**	513	13%	0.02	254	9%	0.03	259	17%	0.04	-8%	-1.82
**3 exam nutrition (sur 6)**	513	13%	0.02	254	15%	0.04	259	10%	0.03	5%	1.07
**4 exam nutrition (sur 6)**	513	7%	0.02	254	7%	0.03	259	6%	0.03	2%	0.42
**5 exam nutrition (sur 6)**	513	5%	0.02	254	5%	0.02	259	6%	0.03	-1%	-0.18
**6 exam nutrition (sur 6)**	513	3%	0.01	254	3%	0.02	259	2%	0.02	1%	0.46
**Referred to STA or SST**	515	1%	0.01	254	2%	0.01	261	0%	0.00	2%	1.48
**Oedeme (o/n)**	515	1%	0.00	254	1%	0.01	261	0%	0.00	1%	0.84
**MAM**	28	75%	0.09	14	71%	0.16	14	79%	0.10	-7%	-0.38
**MAS**	28	25%	0.09	14	29%	0.16	14	21%	0.10	7%	0.38
**MAS avec PB**	14	14%	0.09	7	14%	0.15	7	14%	0.12	0%	0.00
**MAM avec PB**	14	86%	0.09	7	86%	0.15	7	86%	0.12	0%	0.00

Tableau 87. Comparabilité des centres de santé des groups de contrôle et d'intervention – entretiens à la sortie

Exit interviews (a)	All groups			Treatment group			Control group			Treatment - Control	
	Obs	Mean	SD	Obs	Mean	SD	Obs	Mean	SD	diff	tstat
**Il y a eu un diagnostic oui/non**	514	66%	0.04	254	69%	0.05	260	64%	0.05	5%	0.69
**Le diagnostic est un vrai diagnostic (et pas un symptome)**	341	63%	0.04	175	51%	0.06	166	75%	0.05	-23%	-3.00
**Un diagnostic reporté est la malnutrition**	341	1%	0.00	175	0%	0.00	166	1%	0.01	-1%	-1.47
**Le traitement rapporté est "médicaments seulement"**	512	76%	0.02	253	80%	0.03	259	72%	0.03	8%	1.92
**Le traitement rapporté est "médicaments+suivi"**	512	10%	0.02	253	9%	0.02	259	11%	0.02	-2%	-0.66
**Le traitement rapporté comporte des éléments d'alimentation**	514	7%	0.01	254	4%	0.02	260	10%	0.02	-6%	-2.17
**Le traitement rapporté comporte deux éléments d'alimentation (améliorer+allaitement)**	315	1%	0.00	166	0%	0.00	149	1%	0.01	-1%	-1.41
**Des conseils alimentaires ont été rapportés oui/non**	512	23%	0.03	253	31%	0.04	259	16%	0.03	15%	2.87
**Les conseils alimentaires contiennent des éléments sur les différents groupes d'aliments à fournir**	512	15%	0.02	253	19%	0.03	259	10%	0.03	9%	2.03
**Les conseils alimentaires consistent à augmenter la portion alimentaire seulement**	512	0%	0.00	253	0%	0.00	259	0%	0.00	0%	1.01
**Les conseils alimentaires contiennent des éléments d'hygiène**	512	2%	0.01	253	3%	0.01	259	2%	0.01	1%	0.91

**Exit interviews (b)**	**All groups**			**Treatment group**			**Control group**			**T-C**	
	Obs	Mean	SD	Obs	Mean	SD	Obs	Mean	SD	diff	tstat
**Age de l'enfant en mois**	450	25.47	0.69	216	25.67	1.07	234	25.29	0.90	0.38	0.28
**Sexe de l'enfant (1 male - 2 female)**	512	1.51	0.02	253	1.46	0.02	259	1.56	0.03	-0.10	-2.37
**WHZ avec mesures CDS**	266	-0.67	0.10	142	-0.88	0.09	124	-0.41	0.17	-0.47	-2.40
**HAZ avec mesures CDS**	231	-1.85	0.13	118	-1.79	0.12	113	-1.91	0.24	0.12	0.44
**PB mesuré par CDS**	225	138.67	0.97	126	139.60	1.33	99	137.48	1.35	2.12	1.13
**WHZ avec mesures enqueteur**	510	-0.84	0.06	252	-0.87	0.08	258	-0.81	0.08	-0.06	-0.49
**HAZ avec mesures enqueteur**	446	-1.67	0.09	214	-1.53	0.13	232	-1.79	0.13	0.26	1.42
**PB mesure par enqueteur**	511	144.14	0.66	253	144.57	0.95	258	143.71	0.92	0.86	0.66
**Présence d'oedème (1/0) par enquêteur**	511	2%	0.01	253	1%	0.01	258	4%	0.02	-3%	-1.70
**Présence de MA (avec mesures CDS)**	315	17%	0.02	170	15%	0.03	145	20%	0.03	-5%	-1.04
**Présence MA (avec mesures enquêteur)**	511	15%	0.02	253	11%	0.02	258	19%	0.03	-8%	-2.48
**Présence MAM (avec mesures enquêteur)**	510	11%	0.02	252	8%	0.02	258	14%	0.02	-6%	-2.16
**Présence MAS (avec mesures enquêteur)**	510	4%	0.01	252	3%	0.01	258	5%	0.01	-1%	-0.86

Tableau 88. Comparabilité des ménages des groups de contrôle et d'intervention – caractéristiques générales et socio-économiques

HH socio economic var (a)	All groups			Treatment group			Control group			T-C	
	Obs	Mean	SD	Obs	Mean	SD	Obs	Mean	SD	diff	tstat
**Taille du ménage**	6199	5.64	0.04	3099	5.62	0.07	3100	5.66	0.05	-0.04	-0.45
**nb d'enfants de moins de 5a**	6199	1.66	0.01	3099	1.66	0.02	3100	1.67	0.02	-0.01	-0.27
**nb de membres de + de 65a**	6199	0.04	0.00	3099	0.04	0.00	3100	0.04	0.00	0.00	-0.70
**chef de ménage est un homme**	6199	93%	0.01	3099	92%	0.01	3100	93%	0.01	-1%	-0.77
**Âge du chef de ménage**	5422	34.83	0.18	2691	34.73	0.29	2731	34.92	0.23	-0.20	-0.53
**Le chef de ménage est marié**	6199	95%	0.00	3099	95%	0.01	3100	95%	0.01	0%	0.08
**Le chef de ménage n'a pas d'activité**	5756	1%	0.00	2860	2%	0.00	2896	1%	0.00	0%	0.35
**Le chef de ménage travaille dans l'agriculture ou la pêche**	5756	82%	0.01	2860	81%	0.02	2896	83%	0.02	-2%	-0.83
**Le chef de ménage travaille dans le commerce ou les services**	5756	6%	0.01	2860	6%	0.01	2896	5%	0.01	0%	0.40
**Le chef de ménage travaille dans l'éducation ou la santé**	5756	2%	0.00	2860	2%	0.00	2896	2%	0.00	0%	0.11
**Le chef de ménage travaille dans l'administration**	5756	1%	0.00	2860	1%	0.00	2896	1%	0.00	0%	-0.64
**Le chef de ménage sait lire et écrire**	6080	69%	0.01	3026	68%	0.02	3054	70%	0.02	-2%	-0.77
**Le chef de ménage n'a pas d'education**	6015	67%	0.02	3017	66%	0.02	2998	68%	0.02	-2%	-0.60
**Le chef de ménage a une education primaire**	6015	28%	0.01	3017	30%	0.02	2998	27%	0.02	2%	0.88
**Le chef de ménage a une education secondaire ou tertiaire**	6015	4%	0.01	3017	4%	0.01	2998	5%	0.01	0%	-0.49

**Variables socio-economiques du ménage (b)**	**All groups**			**Treatment group**			**Control group**			**T-C**	
	Obs	Mean	SD	Obs	Mean	SD	Obs	Mean	SD	diff	tstat
**A un terrainpour l'agriculture**	6195	90%	0.01	3097	91%	0.02	3098	89%	0.02	2%	0.74
**A un véhicule motorisé**	6199	2%	0.00	3099	2%	0.01	3100	2%	0.00	0%	-0.36
**A un vélo**	6199	24%	0.01	3099	22%	0.02	3100	25%	0.02	-3%	-1.19
**A l'electricité**	6199	5%	0.01	3099	5%	0.01	3100	5%	0.01	0%	-0.08
**A un savon**	6194	65%	0.01	3095	66%	0.02	3099	63%	0.02	3%	1.10
**Source d'eau: fontaine**	6199	40%	0.02	3099	37%	0.03	3100	43%	0.03	-6%	-1.34
**Source d'eau: puits ou source protégé**	6199	37%	0.02	3099	41%	0.03	3100	33%	0.03	9%	2.04
**Source d'eau: puits ou source non protégé**	6199	11%	0.01	3099	12%	0.02	3100	11%	0.01	1%	0.63
**Distance à l'eau: moins de 30 min**	6190	78%	0.02	3095	78%	0.03	3095	77%	0.02	2%	0.51
**Distance à l'eau: + de 30 min et - de 60 min**	6190	18%	0.01	3095	17%	0.02	3095	19%	0.01	-2%	-0.87
**Distance à l'eau: + de 60 min**	6190	5%	0.01	3095	5%	0.01	3095	4%	0.01	0%	0.24
**Traite l'eau avant de boire**	6199	5%	0.00	3099	5%	0.01	3100	4%	0.00	1%	1.37
**Assez d'eau pour toutes les utilisations**	6197	80%	0.01	3099	81%	0.02	3098	79%	0.02	2%	0.68
**Pas assez d'eau**	6197	17%	0.01	3099	16%	0.02	3098	17%	0.02	-1%	-0.41
**Cuisine au charbon**	6199	6%	0.01	3099	5%	0.02	3100	7%	0.01	-1%	-0.49
**Cuisine au feu de bois**	6199	92%	0.01	3099	93%	0.02	3100	91%	0.01	2%	0.73
**Toilettes: latrines**	6198	95%	0.01	3099	95%	0.01	3099	96%	0.01	-2%	-1.48
**Toilettes: latrines partagées avec d'autres (parmi les utilisateurs de latrines)**	5919	16%	0.01	2935	16%	0.02	2984	17%	0.02	0%	-0.17

Tableau 89. Comparabilité des ménages des groups de contrôle et d'intervention en terme de sécurité alimentaire

	All groups			Treatment group			Control group			Treatment - Control	
	Obs	Mean	SD	Obs	Mean	SD	Obs	Mean	SD	diff	tstat
% de ménages en sécurité alimentaire	6189	8%	0.01	3093	10%	0.01	3096	7%	0.01	3%	2.17
% de ménages en insécurité alimentaire légère	6189	5%	0.00	3093	5%	0.01	3096	4%	0.01	1%	1.39
% de ménages en insécurité alimentaire modérée	6189	19%	0.01	3093	21%	0.02	3096	17%	0.01	3%	1.45
% de ménages en insécurité alimentaire grave	6189	68%	0.02	3093	64%	0.03	3096	72%	0.02	-8%	-2.08
% de ménages ayant été préoccupé par le manque de nourriture	6196	79%	0.01	3098	76%	0.02	3098	81%	0.02	-5%	-1.98
% de ménages dont un membre n'a pas pu manger les aliments préférés par manque de ress	6195	86%	0.01	3097	84%	0.01	3098	87%	0.01	-3%	-1.70
% de ménages dont un membre a mangé une variété plus limitée d'aliments par manque de ressources	6198	85%	0.01	3098	83%	0.02	3100	87%	0.01	-4%	-1.71
% de ménages dont un membre a mangé des aliments qu'il n'aimenpas manger par manque de ressources	6194	84%	0.01	3095	82%	0.02	3099	87%	0.01	-5%	-2.20
% de ménages dont un membre a mangé un repas plus petit par manque de ressources	6195	70%	0.02	3097	68%	0.02	3098	72%	0.02	-4%	-1.39
% de ménages dont un membre a mangé moins d'un repas par jour par manque de ressources	6196	62%	0.02	3096	59%	0.03	3100	65%	0.02	-6%	-1.59
% de ménages ayant manqué totalement de nourriture par manque de ressources	6196	61%	0.02	3097	57%	0.03	3099	64%	0.02	-7%	-1.80
% de ménages dont u nmembre est allé au lit en ayant faim par manque de ressources	6197	43%	0.02	3098	42%	0.03	3099	45%	0.02	-3%	-0.84
% de ménages dont un membre a passé la journée sans manger par manque de ressources	6197	17%	0.01	3097	17%	0.02	3100	18%	0.01	-1%	-0.26
Nombre de mois pendant lesquels le ménage a manqué de nourriture	5635	3.97	0.07	2762	3.91	0.10	2873	4.03	0.09	-0.12	-0.93

Tableau 90. Comparabilité des enfants dans les groups de contrôle et d'intervention – caractéristiques générales et de santé

	All groups			Treatment group			Control group			T-C	
	Obs	Mean	SD	Obs	Mean	SD	Obs	Mean	SD	diff	tstat
**Age: 6-11 m**	6199	35%	0.01	3099	36%	0.01	3100	34%	0.01	2%	1.50
**Age: 12-17 m**	6199	33%	0.01	3099	33%	0.01	3100	34%	0.01	-1%	-0.46
**Age: 18-23 m**	6199	31%	0.01	3099	31%	0.01	3100	32%	0.01	-1%	-1.09
**Sexe: masculin**	6199	49%	0.01	3099	49%	0.01	3100	49%	0.01	0%	-0.25
**A été malade durant les deux dernières semaines**	6199	59%	0.01	3099	57%	0.02	3100	61%	0.01	-3%	-1.63
**Parmi les maades: a eu diarrhée**	3668	35%	0.01	1781	35%	0.02	1887	35%	0.01	1%	0.24
**Parmi les maades: a eu fievre**	3668	55%	0.01	1781	55%	0.02	1887	54%	0.02	1%	0.38
**Parmi les maades: a eu inf. Respiratoire**	3668	33%	0.02	1781	34%	0.02	1887	33%	0.02	1%	0.35
**Parmi les maades: a visité CDS**	3668	81%	0.01	1781	80%	0.01	1887	82%	0.01	-1%	-0.71
**Fréquente un CDS**	6199	100%	0.00	3099	100%	0.00	3100	100%	0.00	0%	-2.25
**Distance au CDS: < 30 minutes**	5996	22%	0.02	2979	22%	0.02	3017	22%	0.02	0%	-0.09
**Distance au CDS: 30-59 minutes**	5996	28%	0.01	2979	28%	0.02	3017	29%	0.02	-1%	-0.53
**Distance au CDS: > une heure**	5996	50%	0.02	2979	51%	0.03	3017	49%	0.02	2%	0.40
**Est dans un SSN**	6199	0.6%	0.00	3099	0.8%	0.00	3100	0.4%	0.00	0.4%	1.68
**Est dans un STA**	6199	0.6%	0.00	3099	0.9%	0.00	3100	0.4%	0.00	0.5%	1.27
**A un carnet de santé**	6199	79%	0.01	3099	79%	0.02	3100	80%	0.01	-1%	-0.37

Tableau 91. Comparabilité des enfants dans les groups de contrôle et d'intervention – mesures anthropométriques

	All groups			Treatment group			Control group			T-C	
	Obs	Mean	SD	Obs	Mean	SD	Obs	Mean	SD	diff	Tstat
**PB (mm)**	6199	139.92	0.28	3099	140.10	0.38	3100	139.75	0.42	0.35	0.63
**PB < 115 mm**	6199	2%	0.00	3099	2%	0.00	3100	2%	0.00	-1%	-1.82
**Oedeme**	6199	2%	0.00	3099	2%	0.00	3100	2%	0.00	-1%	-1.02
**WHZ des enfants de 6-11m**	2181	-0.25	0.03	1118	-0.25	0.04	1063	-0.25	0.05	-0.01	-0.10
**WHZ des enfants de 12-17**	2076	-0.45	0.03	1030	-0.46	0.04	1046	-0.44	0.04	-0.02	-0.33
**WHZ des enfants de 18-23m**	1941	-0.33	0.03	950	-0.33	0.04	991	-0.33	0.04	0.00	0.05
**Enfants de 6-11m avec MA**	2181	6%	0.01	1118	6%	0.01	1063	6%	0.01	-1%	-0.71
**Enfants de 12-17avec MA**	2076	7%	0.01	1030	7%	0.01	1046	7%	0.01	-1%	-0.59
**Enfants de 18-23m avec MA**	1942	5%	0.00	951	5%	0.01	991	5%	0.01	0%	0.34
**Enfants de 6-11m avec MAS**	2181	1%	0.00	1118	1%	0.00	1063	1%	0.00	0%	0.87
**Enfants de 12-17avec MAS**	2076	1%	0.00	1030	2%	0.00	1046	1%	0.00	1%	2.20
**Enfants de 18-23m avec MAS**	1942	1%	0.00	951	1%	0.00	991	1%	0.00	0%	-1.02
**HAZ des enfants de 6-11m**	2181	-1.71	0.04	1118	-1.72	0.05	1063	-1.69	0.05	-0.03	-0.44
**HAZ des enfants de 12-17**	2076	-2.18	0.03	1030	-2.17	0.05	1046	-2.18	0.04	0.01	0.20
**HAZ des enfants de 18-23m**	1942	-2.46	0.04	951	-2.49	0.05	991	-2.44	0.05	-0.04	-0.63
**Enfants de 6-11m avec malnutrition chronique**	2181	39%	0.01	1118	39%	0.02	1063	38%	0.02	1%	0.28
**Enfants de 12-17avec malnutrition chronique**	2076	56%	0.01	1030	57%	0.02	1046	56%	0.02	1%	0.40
**Enfants de 18-23m avec malnutrition chronique**	1942	66%	0.01	951	66%	0.02	991	66%	0.02	0%	0.14
**Enfants de 6-11m avec maln. chronique sévère**	2181	12%	0.01	1118	13%	0.01	1063	11%	0.01	2%	1.53
**Enfants de 12-17avec maln. chronique sévère**	2076	22%	0.01	1030	23%	0.01	1046	22%	0.01	1%	0.61
**Enfants de 18-23m avec maln. chronique sévère**	1942	29%	0.01	951	31%	0.02	991	28%	0.02	3%	1.15
**Nb de poids reportés dans le carnet des 6-11m**	1873	3.4	0.19	948	3.6	0.28	925	3.3	0.25	0.3	0.70
**Nb de poids reportés dans le carnet des 12-17m**	1618	4.1	0.24	802	4.3	0.36	816	3.8	0.32	0.6	1.16
**Nb de poids reportés dans le carnet des 18-23m**	1424	4.8	0.31	694	5.5	0.51	730	4.2	0.33	1.3	2.17
**Pas de courbe tracée dans le carnet**	4917	90%	0.02	2445	87%	0.03	2472	92%	0.02	-5%	-1.36
