# Supplementary material for: Impact of the extension of a performance-based financing scheme to nutrition services in Burundi on malnutrition prevention and management among children below five: A cluster-randomized control trial
Source: PLoS One. 2020 Sep 18;15(9):e0239036. doi: 10.1371/journal.pone.0239036 (PMC7500612; doi:10.1371/journal.pone.0239036)
Supplement: S8 File — Evaluation d’impact au niveau des centres de santé et des ménages ». Source: Authors. (PDF) [file pone.0239036.s010.pdf]

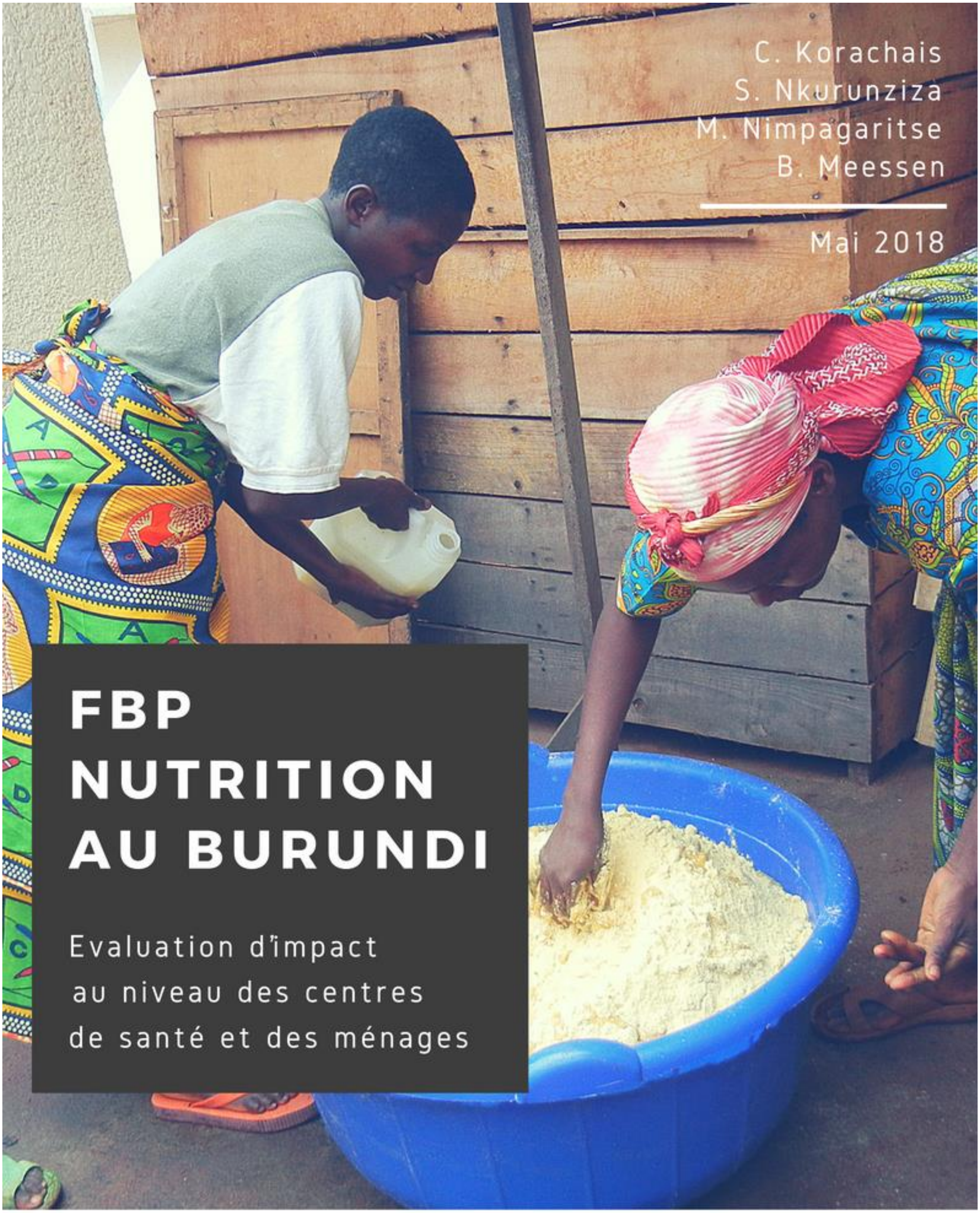

C. Korachais
S. Nkurunziza
M. Nimpagaritse
B. Meessen

Mai 2018

FBP NUTRITION AU BURUNDI

Evaluation d'impact au niveau des centres de santé et des ménages

Ce rapport présente les résultats de l'étude d'impact du financement basé sur la performance (FBP) appliqué à la nutrition au Burundi. Les données utilisées proviennent d'enquêtes réalisées sur la période septembre 2014 à janvier 2015 (baseline) et février à avril 2017 (endline). Le FBP appliqué à la nutrition a pour objectif d'améliorer la prévention et la prise en charge de la malnutrition dans le système de santé burundais et a été mis en place dans le groupe d'intervention de janvier 2015 à décembre 2017.

Remerciements

Cette étude d'impact du financement basé sur la performance appliqué à la nutrition au Burundi est financée par la Banque Mondiale, à travers le *Health Results Innovation Trust Fund (HRITF)*. Les auteurs de ce rapport tiennent à remercier les membres de la Banque Mondiale et du Ministère de la Santé Publique et de la Lutte contre le SIDA impliqués dans ce projet, et particulièrement ceux de la Cellule Technique chargée du FBP et ceux du PRONIANUT, pour leur collaboration. Nous souhaitons également exprimer notre gratitude envers l'INSP et l'ISTEEBU qui ont mis en place et mené à bien les enquêtes respectivement auprès des centres de santé et des ménages. En outre, les membres de notre équipe Kirrily de Polnay, Maxime Rouve, Ulises Huerta, Elodie Macouillard, Léonard Ntakarutimana, Désiré Munezero et Jacqueline Manisabwe méritent tout autant d'être salués pour leur aide remarquable durant la préparation et la mise en place des enquêtes. Enfin, nous souhaiterions remercier les membres du Comité Technique et du Comité de Pilotage pour leur suivi et leurs commentaires contructifs.

Comment citer ce rapport

Korachais, C., S. Nkurunziza, M. Nimpagaritse, et B. Meessen. 2018. FBP Nutrition au Burundi : Evaluation d'impact au niveau des centres de santé et des ménages. Institut de Médecine Tropicale d'Anvers, Belgique.

Table des matières

Acronymes et abréviations 5
Index des tableaux 7
Index des figures 8
1. Vue d'ensemble du projet d'étude 9
1.1 Contexte au début du projet 9
1.2 Intervention évaluée 11
1.3 Objectifs de l'étude 13
2. Méthodologie 17
2.1 Design de l'étude 17
2.2 Randomisation et taille d'échantillon 17
2.3 Variables pour l'analyse des données 19
2.4 Instruments de collecte et d'assurance qualité 19
2.5 Analyse 24
2.6 Stockage, gestion et politique d'accès aux données 24
3. Représentativité de l'échantillon et validité externe de l'étude 25
3.1 Validité externe 25
3.2 Validité interne 25
4. Résultats de l'évaluation d'impact 27
4.1 Données analysées 27
4.2 Mise en œuvre de l'intervention 28
4.3 Impact sur les indicateurs d'input et de processus 38
4.4 Impact sur les indicateurs d'output 57
4.5 Impact sur les indicateurs de résultat 64
5. Discussion 71
5.1 Mise en œuvre de l'intervention 71
5.2 Inputs et processus 72
5.3 Output 77
5.4 Résultat clinique de la prise en charge 79
5.5 Limites de l'étude 80
6. Recommandations 81
6.1 A l'égard du MSPLS pour la mise à l'échelle du FBP Nutrition 81
6.2 A l'égard du MSPLS de manière générale 83
6.3 A l'égard des commanditaires d'autres études futures 85
7. Références bibliographiques 87
8. Annexes 89
8.1 Instruments de collecte – les vignettes 89
8.2 Statistiques descriptives clé générales de l'enquête de 2017 90
8.3 Statistiques descriptives des données de routine du FBP Nutrition (groupe d'intervention) 109
8.4 Tableaux synthétiques sur les résultats de l'intervention (mise en œuvre et impact) et les recommandations politiques 111

Acronymes et abréviations

ACT	Combinaisons Thérapeutiques à base d'Artémisinine (*Artemisinin-based Combination Therapy*)
ANJE	Alimentation du nouveau-né et du jeune enfant
AS	Agent de santé
ASC	Agent de santé communautaire
BF	Allaitement (*Breastfeeding*)
BM	Banque Mondiale
CDS	Centre de santé
CSB	Mélange maïs-soja (*Corn Soya Blend*)
CTA	Combinaisons Thérapeutiques à base d'Artémisinine
CT-FBP	Cellule Technique pour le FBP
DPSHA	Direction de la Promotion de la Sante Hygiène et Assainissement
DODS	Direction de l'Offre et la Demande de Soins
EDS	Enquête Démographie et Santé
FANTA	Food and Nutrition Technical Assistance
FARN	Foyers d'Apprentissage et de Réhabilitation Nutritionnelle
FBP	Financement Basé sur la Performance
FOSA	Formation sanitaire
GASC	Groupement d'agents de santé communautaire
HAZ	z-score du rapport taille pour âge *(Height-for-Age Z-score)*
HFIAS	Echelle de l'accès déterminant l'insécurité alimentaire des ménages *(Household Food Insecurity Access Scale)*
ICC	Coefficient de correlation intra-classe *(Intraclass Correlation Coefficient)*
IMT	Institut de Médecine Tropicale d'Anvers
INSP	Institut National de Santé Publique
ISTEEBU	Institut de Statistiques et d'Etudes Economiques du Burundi
IV	Intraveineuse
IYCF	Alimentation du nouveau-né et du jeune enfant *(Infant and Young Child Feeding)*
LQAS	Assurance de qualité de lots *(Lot Quality Assurance Sampling)*
MA	Malnutrition aigüe
MAM	Malnutrition aigüe modérée
MAS	Malnutrition aigüe sévère
Max	Maximum
Med	Médiane
Moy	Moyenne
Min	Minimum
MSPLS	Ministère de la Santé Publique et de la Lutte contre le SIDA
MUAC	Périmètre brachial *(Mid-Upper Arm Circumference)*
N	Nombre d'observations
N att	Nombre d'observations attendues
ODK	Open Data Kit
OMD	Objectif du Millénaire pour le Développement
OMS	Organisation Mondiale de la Santé
ONG	Organisation Non-Gouvernementale
ORS	Solution de réhydratation orale *(Oral Rehydration Solution)*
PAM	Programme Alimentaire Mondial

PB	Périmètre brachial
PCIME	Prise en charge intégrée des maladies de l'enfant
PEC	Prise en charge
PMA	Paquet minimum d'activités
PNUD	Programme des Nations Unies pour le Développement
PRONIANUT	Programme de Nutrition (au MSPLS)
P/T	Poids pour taille
PTF	Partenaire technique et financier
SD	Ecart-type *(Standard Deviation)*
SRO	Solution de réhydratation orale
SSN	Service de Supplémentation Nutritionnelle (service de prise en charge de la MAM)
SST	Service de Stabilisation (service de prise en charge de la MAS avec complications)
STA	Service de Thérapeutique Ambulatoire (service de prise en charge de la MAS sans complications)
T/A	Taille pour âge
UNICEF	Fonds des Nations Unies pour l'Enfance *(United Nations Children's Fund)*
URTI	Upper Respiratory Tract Infection (Infection Respiratoire Supérieure Aigue)
WHZ	Z-score du rapport poids pour taille *(Weight-for-Height Z-score)*

Index des tableaux

Tableau 1. Indicateurs conçus dans le cadre de l'intervention du FBP Nutrition 12
Tableau 2. Chemins à considérer dans les groupes d'intervention et de contrôle 15
Tableau 3. Variables de résultats à collecter et analyser 19
Tableau 4. Variables de résultats à analyser au niveau de la structure de soins 19
Tableau 5. Taux de couverture pour chacun des outils de collecte de l'enquête CDS 27
Tableau 6. Différence absolue entre le poids des enfants mesuré par les balances des CDS et le poids mesuré par les balances SECA (gold standard), en 2014 et 2017 38
Tableau 7. Différence absolue entre le poids des enfants mesuré par les balances des CDS et le poids mesuré par les balances SECA (gold standard), en 2014 et 2017 39
Tableau 8. Disponibilité et rupture des traitements diététiques, en 2014 et 2017 40
Tableau 9. Liste des questions et examens relatifs à la nutrition 41
Tableau 10. Réalisation des questions et examens relatifs à la nutrition en consultation 42
Tableau 11. Les agents de santé ont-ils donné des conseils nutritionnels et d'hygiène aux patients ? 42
Tableau 12. Qualité des mesures anthropométriques dans les CDS en 2014 et 2017 43
Tableau 13. Réalisation des questions et examens relatifs à la nutrition en consultation dans les vignettes, en 2014 et 2017 44
Tableau 14. Conseils nutritionnels et d'hygiène donnés par les AS aux patients fictifs des vignettes 45
Tableau 15. Taux de succès sur les diagnostics de malnutrition sur les vignettes en 2014 et 2017 45
Tableau 16. Ressources humaines dans les CDS en 2014 et 2017 47
Tableau 17. Formations des ressources humaines dans les CDS en 2014 et 2017 48
Tableau 18. Activités réalisées par les agents de santé communautaire relatives à la nutrition faites sous l'encadrement du CDS 49
Tableau 19. Supervision en 2014 et 2017 49
Tableau 20. Disponibilité et actualisation de la documentation SSN et STA en 2014 et 2017 51
Tableau 21. Remplissage des fiches cliniques SSN, en 2014 et 2017 54
Tableau 22. Remplissage des fiches cliniques STA, en 2014 et 2017 55
Tableau 23. Tableau synthétique – sur le remplissage des fiches SSN et STA 56
Tableau 24. Suivi de la croissance en 2014 et 2017 58
Tableau 25. Dépistages de malnutrition aigue observés en consultations curatives 59
Tableau 26. Les paramètres d'entrée dans les programmes SSN et STA en 2014 et 2017 60
Tableau 27. Nombre moyen de cas enregistrés dans les services SSN et STA sur la période de six mois précédant l'enquête, en 2014 et 2017 61
Tableau 28. Traitements systématique et diététique reçus par les enfants traités dans le SSN 62
Tableau 29. Traitements systématique et diététique reçus par les enfants traités dans le STA 63
Tableau 30. Pourcentage d'inscrits en SSN ou STA dans la communauté 63
Tableau 31. Séances de sensibilisation et culinaires effectuées par les ASC, en 2017 seulement 64
Tableau 32. Performance des SSN en 2014 et 2017 65
Tableau 33. Performance des STA en 2014 et 2017 67
Tableau 34. Pratiques alimentaires des enfants dans la communauté, en 2014 et 2017 68
Tableau 35. Jugement sur l'état de malnutrition de l'enfant 69

Tableau 36. Statistiques descriptives des indicateurs de résultats au niveau de la communauté, par groupe d'intervention et par période 70
Tableau 37. Résumé des vignettes cliniques 89
Tableau 38. Ressources humaines 91
Tableau 39. Mesures et conseils réalisés et protocoles utilisés dans le cadre du suivi et de la promotion de la croissance en 2017 92
Tableau 40. Disponibilité du traitement diététique 92
Tableau 41. Interruption des services de nutrition en 2017 93
Tableau 42. Source des dossiers médicaux individuels du SSN en 2017 94
Tableau 43. Mesures anthropométriques à l'entrée et à la sortie et les raisons de sortie du service SSN 95
Tableau 44. Performance du service SSN par rapport aux directives nationales 96
Tableau 45. Traitement systématique et diétetique au service SSN 96
Tableau 46. Source des dossiers médicaux individuels du STA en 2017 97
Tableau 47. Mesures anthropométriques à l'entrée et à la sortie et les raisons de la sortie du service STA, lorsque l'information est disponible 98
Tableau 48. Performance du STA comparée aux directives nationales (de 2010) 98
Tableau 49. Traitements systématique et diétetique en STA 98
Tableau 50. Nombre de cas interrogés sur les signes de danger, les principaux symptômes et les questions autonomes 100
Tableau 51. Nombre de cas avec examens et signes vitaux effectués 100
Tableau 52. Questions et examens relatifs à la nutrition exécutés 101
Tableau 53. Différences dans les mesures anthropométriques 103
Tableau 54. Conditions de sécurité alimentaire 104
Tableau 55. Prévalence des différentes catégories de sécurité alimentaire 104
Tableau 56. Etat de santé de l'enfant 105
Tableau 57. Indicateurs relatifs à l'allaitement 106
Tableau 58. Indicateurs relatifs à l'alimentation de complément 106
Tableau 59. Mesures anthropométriques 107
Tableau 60. Détail des évaluations qualité dans le groupe d'intervention sur 2015-2016 109
Tableau 61. Indicateurs FBP Nutrition validés par semestre en 2015 et 2016 110
Tableau 62. Mise en œuvre du FBP Nutrition à travers la loupe de la théorie du changement 111
Tableau 63. Résultats sélectionnés: statistiques descriptives dans le groupe d'intervention en 2017 et impact estimé du FBP Nutrition 112
Tableau 64. Recommandations politiques : problèmes identifiés et actions proposées 115

Index des figures

Figure 1. Plan de mise en œuvre du FBP Nutrition et de la compensation par niveau et par groupe 13
Figure 2: Chaine des résultats liés à l'introduction de la Nutrition dans le FBP 15
Figure 3. Structure des questionnaires de l'enquête au niveau des centres de santé 20
Figure 4. Timeline du FBP Nutrition 35
Figure 5. Mise en œuvre du FBP Nutrition et de la compensation, par niveau et groupe de traitement.. 36
Figure 6. Performance des 317 agents de santé enquêtés en 2014 et 2017 46

1. Vue d’ensemble du projet d’étude

1.1 Contexte au début du projet

Objectifs du Millénaire pour le Développement (OMDs) au Burundi : où en sommes-nous et quels sont les principaux défis ?

Le Burundi est un petit pays enclavé se trouvant à l’Est de l’Afrique Centrale. Avec un Produit National Brut par habitant estimé en 2014 à 770 dollars internationaux (Banque Mondiale 2015), le Burundi est parmi les pays les plus pauvres du monde. La population burundaise est d’environ 10.6 millions avec 421 habitants par km carré. Toutefois il importe de signaler que la majorité de cette même population vit en milieu rural. Depuis l’indépendance, le Burundi a connu de continuels soubresauts de conflits et d’instabilité, et depuis 1993 jusqu’à récemment, le pays était en état de guerre civile. Par conséquent, l’état de santé de la population est parmi les plus mauvais d’Afrique. En 2017, le taux de mortalité des enfants de moins de 5 ans était encore estimé à 47 décès pour 1000 naissances, tandis que le taux de mortalité maternelle était de 334 décès pour 100 000 naissances (EDS 2018).

Même si l’on constate de nombreux efforts dans le domaine de la vaccination, de la lutte contre les maladies graves ainsi que dans l’allocation des ressources à la santé, de nombreux défis demeurent, comme notamment la faible capacité du système sanitaire en termes d’infrastructures et du personnel, un taux de natalité élevé, l’insécurité alimentaire et sanitaire, etc. L’insécurité alimentaire est aussi la conséquence d’une forte pression démographique et d’un manque d’investissement dans le domaine de l’agriculture (UNDP 2010). Au Burundi, environ 28% des ménages souffrent d’insécurité alimentaire, et les taux de malnutrition chronique sont parmi les plus élevés au monde avec 56% des enfants de moins de 5 ans affectés par un retard de croissance (EDS 2018; WFP 2008).

Histoire du FBP au Burundi

Comme tout pays très pauvre, le Burundi a des contraintes budgétaires serrées eu égards des importants besoins de santé de sa population. Alors que les paiements directs demeurent la source de financement de la santé la plus importante, le gouvernement du Burundi a fait des efforts considérables ces dernières années : la décision présidentielle de supprimer les frais à charge de l’usager pour les enfants de moins de cinq ans et les accouchements au niveau des centres de santé était une étape clé de cet engagement. Cette décision était cohérente avec l’agenda des OMDs, adopté au Burundi, et a bénéficié d’un soutien politique fort. Cependant, comme pour d’autres initiatives similaires en Afrique sub-Saharienne (Meessen et al. 2011), cette décision n’était pas parfaitement planifiée et la suppression des frais à charge de l’usager a souffert de cette préparation limitée (Nimpagaritse et Bertone 2011), avec des défauts dans la conception et la mise en œuvre de l’initiative ainsi qu’un manque de système de suivi et évaluation robuste. L’augmentation soudaine et massive de l’utilisation des services de santé a également été source de frustrations et démotivations parmi le personnel de santé.

Parallèlement, dès 2006, le Burundi expérimentait dans trois provinces une nouvelle stratégie de financement de la santé, le financement basé sur la performance (FBP). Cette stratégie s’est développée

petit à petit et a rapidement gagné en popularité pour finalement être mise en œuvre à l'échelle nationale en avril 2010. Le Burundi est ainsi devenu le deuxième pays d'Afrique à avoir mis en œuvre le FBP dans le secteur santé au niveau national. Dans le même temps, on a pu observer des améliorations importantes au niveau des mécanismes de financement de la santé, en termes de cohérence, gouvernance et vision. La décision la plus cruciale a probablement été celle de fusionner la stratégie de gratuité avec le programme de FBP (Basenya et al. 2011).

Toutes les formations sanitaires (FOSA) publiques et presque toutes les FOSA privées à but non lucratif sont couvertes par le programme national de FBP. Ce programme est gouverné par la Cellule Technique FBP (CT-FBP), un groupe technique basé au Ministère de la Santé Publique et de la Lutte contre le VIH/SIDA (MSPLS). Le système de FBP burundais reflète les principales priorités sanitaires du pays, à savoir le VIH/SIDA, la tuberculose, la santé maternelle et de l'enfant.

Le système de FBP repose sur des arrangements contractuels impliquant différentes parties. Les FOSA rapportent mensuellement leurs activités liées aux indicateurs FBP. Ces rapports sont ensuite vérifiés et validés au niveau provincial par le comité provincial de vérification et de validation (CPVV), puis envoyés à la CT-FBP, qui lance alors les transferts de subventions FBP aux FOSA. Dans les conditions normales de disponibilité des ressources chez tous les acteurs impliqués, le processus prend entre deux et trois mois (MSPLS 2011) ; cela peu prendre un peu plus de temps si l'un ou l'autre a des difficultés de trésorerie. Le programme national de FBP est financé à travers différentes sources, le gouvernement et la Banque Mondiale (HSDSP) étant les deux plus grosses sources de financement.

Malnutrition: un des principaux problèmes de santé publique au Burundi

Au Burundi, la malnutrition est une barrière évidente à la réalisation des OMDs liés à la santé. En effet, encore aujourd'hui, 56% des enfants de moins de 5 ans souffrent de malnutrition chronique et 5% de malnutrition aigüe (EDS, 2018). Les chiffres de malnutrition chronique sont particulièrement alarmants : ils sont en effet plus de 20 points supérieurs aux moyennes du groupe des pays à revenus faibles (36%) et de la région d'Afrique sub-Saharienne (34%). Le Burundi est en fait un des trois pays dans le monde ayant la pire situation en matière de malnutrition chronique ces dernières années, avec le Timor-Leste et l'Erythrée. Cela est d'autant plus alarmant qu'on ne voit aucune amélioration depuis ces trente dernières années : la prévalence de malnutrition chronique chez les enfants de moins de cinq ans était de 56% en 1987 (UNICEF, WHO, and The World Bank 2017).

Sensible à ce problème et selon la tendance internationale, le Ministère chargé de la Santé du Burundi a développé en 2010 un protocole proposant un plan de traitement et de suivi de la malnutrition aigüe pour les enfants de moins de cinq ans, intégré au système de santé (c'est-à-dire aux centres de santé et hôpitaux). Aujourd'hui néanmoins, seulement un tiers des centres de santé et la moitié des hôpitaux offrent des services de prise en charge de la malnutrition aigüe (UNICEF, communication personnelle, 2013).

De plus, la mise en œuvre des services de nutrition à tous les niveaux est très dépendante des intrants provenant des partenaires techniques et financiers, principalement l'UNICEF et le Programme Alimentaire Mondial (PAM). Un autre problème observé par rapport aux intrants est le manque de

gestion des stocks (par exemple, les fiches de stock sont habituellement mal remplies, il n'y a pas de suivi de la distribution des intrants, etc. (Ntakarutimana & Nimpagaritse 2013). Le résultat est que les FOSA souffrent régulièrement de pénuries d'intrants thérapeutiques er nutritionnels. D'ailleurs, pour des raisons de contraintes budgétaires, le PAM a, en 2014, arrêté d'approvisionner en suppléments nutritionnels la majeure partie du pays, rendant ainsi les services de prise en charge de la malnutrition aigüe modérée (MAM) ineffectifs. Un autre problème observé est le manque de connaissances et de compétences parmi les professionnels de la santé impliqués dans les activités de malnutrition (Ntakarutimana & Nimpagaritse 2013). En effet, même si des formations spécifiques ont été réalisées en 2010, puis en 2014-2015, on constate toujours, parmi les professionnels de santé et les agents de santé communautaires, un manque de connaissances relatives à la prévention et la prise en charge de la malnutrition (en partie du fait de rotations importantes du personnel) ainsi qu'une motivation réduite. Le manque de supervision des activités liées à la nutrition pourraient jouer un rôle.

1.2 Intervention évaluée

Avec le soutien de la Banque Mondiale, le gouvernement Burundais a souhaité renforcer ce système, en introduisant des indicateurs liés à la nutrition dans le FBP existant. Il s'agissait alors d'une opportunité à la fois pour améliorer le statut nutritionnel des enfants et pour réaliser une analyse rigoureuse des forces et des faiblesses du FBP. En effet, l'intégration des services de nutrition dans le FBP est un défi dans la mesure où actuellement, la mise en œuvre des services de nutrition est fortement dépendante d'organisations externes comme l'UNICEF ou le Programme Alimentaire Mondial (PAM) qui fournissent les intrants nutritionnels. De plus, on constate que les services de nutrition sont négligés de par un manque de connaissances et de savoir-faire du personnel de santé et un manque de supervision des activités de nutrition. Aussi, l'introduction du FBP devrait pouvoir permettre de réduire ces obstacles, situés au niveau du système de santé. Il est cependant admis que la malnutrition est le résultat de nombreux facteurs qui ne relèvent pas du système de santé (socio-économiques notamment, cf. Nkurunziza et al. 2017) ; aussi, l'intervention FBP Nutrition à elle seule ne peut pas résoudre le problème de la malnutrition au Burundi, et devrait idéalement faire partie d'un programme global d'interventions dans plusieurs secteurs (cf. SUN initiative, http://scalingupnutrition.org/)

Afin de s'attaquer au défi de la malnutrition et de fournir des soins appropriés aux enfants malnutris, le MSPLS, avec le soutien de la Banque Mondiale, a décidé d'introduire des indicateurs liés à la nutrition dans le FBP existant. L'objectif était de renforcer la stratégie nationale d'intégration des services nutritionnels et de la rendre effective dans toutes les régions du Burundi. L'intervention FBP Nutrition consistait à introduire des incitations à trois niveaux (hôpital, centre de santé et niveau communautaire), à la fois sur le volet préventif et curatif, tous se focalisant sur les enfants de moins de 5 ans (cf. Tableau 1).

Tableau 1. Indicateurs conçus dans le cadre de l'intervention du FBP Nutrition

Niveau	Bonus pour
**Communautaire**	(1) le dépistage et la référence vers les centres de santé nutritionnels pour les cas de malnutrition aigüe,<br>(2) l'organisation de séances de sensibilisation pour la promotion de régimes alimentaires sains et de bons comportements nutritionnels, et<br>(3) l'organisation de cours de cuisine.
**Centre de Santé**	(1) le dépistage de malnutrition aigüe chez les enfants et la bonne prise en charge des cas de malnutrition aigüe (selon le type de centre de santé*), et<br>(2) pour la promotion et le suivi de la croissance.
**ospitalier**	(1) la bonne prise en charge des cas sévères de malnutrition aigüe avec complications médicales, et<br>(2) le nombre de journées d'hospitalisation liées à la malnutrition aigüe.

Source: Note Technique (MSPLS 2013). Note:*En effet, il existe plusieurs sortes de centres de santé : la plupart des centres au Burundi ne fournissent pas de services pour les cas de malnutrition aigüe, certains fournissent des services de prise en charge des cas de malnutrition aigüe modérée, certains pour les cas de malnutrition aigüe sévère, et d'autres pour les cas de malnutrition aigüe modérée et sévère.

Une grille qualitative spécifique à la nutrition est prise en compte dans le calcul des bonus[1]. Ainsi, la qualité des activités de nutrition est évaluée chaque trimestre, de la même manière que pour les autres indicateurs de qualité. Un bonus ou un malus qualité est appliqué aux subventions reçues par les FOSA, selon leur score de qualité : si le score est supérieur à 80%, alors la FOSA reçoit un bonus équivalent au score multiplié par 25% des subventions FBP nutrition ; au contraire, si le score de qualité est inférieur à 60%, la FOSA est sujette à un malus allant de 10 à 25% du montant de subventions FBP Nutrition.

De plus, l'efficacité et la qualité des services de nutrition au niveau de la communauté sont évalués par des associations locales (ASLO). Cette évaluation est réalisée tous les six mois, en même temps que l'évaluation communautaire mise en œuvre pour le FBP général.

Le système de vérification est à peu près le même que pour les autres indicateurs FBP au Burundi. Les centres de santé rapportent mensuellement la quantité de services encouragés par le FBP effectivement réalisés. Ces rapports sont vérifiés puis validés au niveau provincial par les Comités Provinciaux de Vérification et de Validation (CPVV), et envoyés à la CT-FBP (i.e. niveau central), qui effectue les transferts de subventions vers les centres de santé. Avec l'introduction des indicateurs nutrition dans le FBP existant, la vérification et la validation des indicateurs nutrition au niveau du centre de santé sont réalisés par les CPVV, de la même manière que pour le FBP existant. Au niveau de la communauté, la pré-vérification de la quantité des services nutritionnels réalisés par les ASC est effectuée par les centres de santé qui les supervisent, à l'aide des registres et rapports qui leurs sont rapportés. Le processus de vérification et validation est ensuite finalisé par le CPVV.

[1] En fait, cette grille spécifique a été mise en place en juillet 2015 et a été utilisée rétrospectivement pour évaluer la qualité des activités nutrition subventionnées à partir du mois d'avril 2015. Pour les mois précédents (janvier à mars 2015), c'est la grille qualité générale pré-existante qui a été utilisée.

Il était aussi prévu que le groupe de contrôle reçoive des compensations financières sous forme de dotations, équivalentes aux subventions FBP Nutrition reçues dans le groupe d'intervention (selon une moyenne pondérée prenant en compte le volume des activités et du personnel).

Figure 1. Plan de mise en œuvre du FBP Nutrition et de la compensation par niveau et par groupe

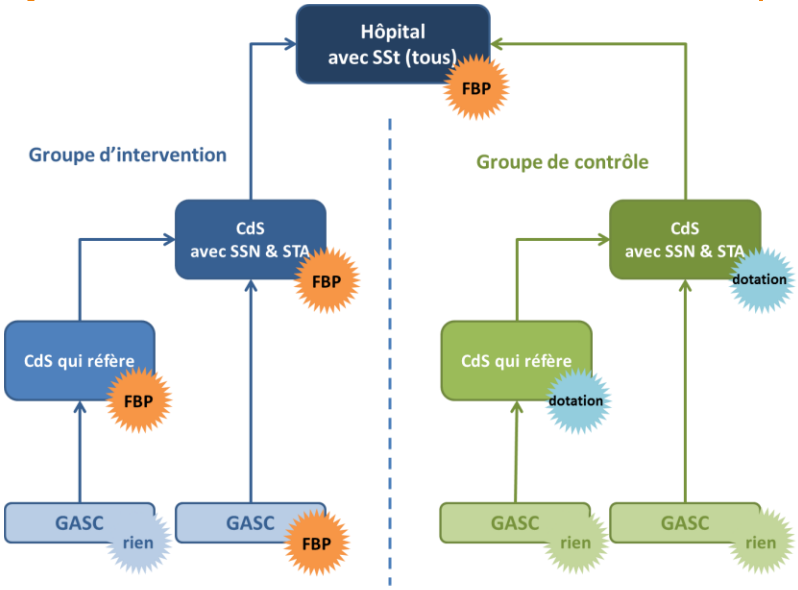

Source: auteurs.

La mise en œuvre effective de l'intervention est décrite dans la section Résultats / Mise en œuvre de l'intervention (page 27).

1.3 Objectifs de l'étude

Théorie du changement

L'introduction d'indicateurs de nutrition dans le FBP devrait avoir un impact sur la performance des structures de santé de différentes manières. Nous avons identifié six chemins par lesquels le FBP a traditionnellement un impact sur la performance du système de santé, et nous pensons que chacun de ces chemins jouera un rôle dans la performance du FBP Nutrition en particulier :

1) **Chemin des revenus** : l'injection de ressources financières (conditionnée par la performance) aura un impact positif sur les services de nutrition, dans la mesure où cela permet au responsable de la structure de soins de recruter plus de personnel, de mieux équiper la structure, etc.

2) **Chemin des incitations** : si les tarifs du FBP Nutrition sont suffisamment élevés, les agents de santé seront motivés à accroître leur performance afin d'augmenter leur environnement de travail et leur salaire. Au niveau de la structure, l'effet sur les autres services reste incertain. Ils peuvent être négatifs pour les uns (i.e. si les agents en charge de la nutrition avaient l'habitude de s'occuper d'autres services et s'en détournent maintenant) et positifs pour d'autres (il y a des possibilités d'économies d'échelle, par exemple les activités de promotion et de suivi de la croissance peuvent être réalisées en même temps que les séances de vaccinations des enfants).

3) **Chemin de l'information** : à travers le contrat de FBP Nutrition (et les séances de formation), le personnel aura une meilleure compréhension sur ce qu'est la performance en matière de nutrition. De plus, les feedbacks provenant du système FBP les guideront et indiqueront leurs marges d'amélioration dans ces services. On suppose un effet positif sur les services de nutrition.

4) **Chemin de la supervision** : la vérification s'étendra aux services de nutrition, notamment sur requête des centres de santé ; aussi, la supervision devrait s'accompagner de conseils aux agents de santé sur les bonnes pratiques et de reconnaissance de leurs efforts.

5) **Chemin de la culture d'entreprise** : le FBP implique un changement d'un système passif à un système où l'initiative, la créativité et les réalisations d'objectifs sont valorisés. On suppose que cette voie est déjà active étant donné que le FBP est une politique nationale depuis trois ans.

6) **Chemin du système de santé** : on s'attend à certains effets sur le système de santé. Certains de ces effets proviendront des partenaires impliqués dans l'évaluation d'impact (par exemple, le MSPLS demandant à l'UNICEF et au PAM de fournir plus d'intrants nutritionnels). Une partie viendra des structures de santé (par exemple, pression du responsable d'une FOSA auprès du PRONIANUT pour qu'il devienne un fournisseur fiable et rapide). On attend également que les agents de santé communautaires réfèreront les enfants mal nourris aux centres de santé et que ces centres réfèreront à leur tour les enfants en situation de malnutrition sévère aux hôpitaux de référence.

Figure 2: Chaine des résultats liés à l'introduction de la Nutrition dans le FBP

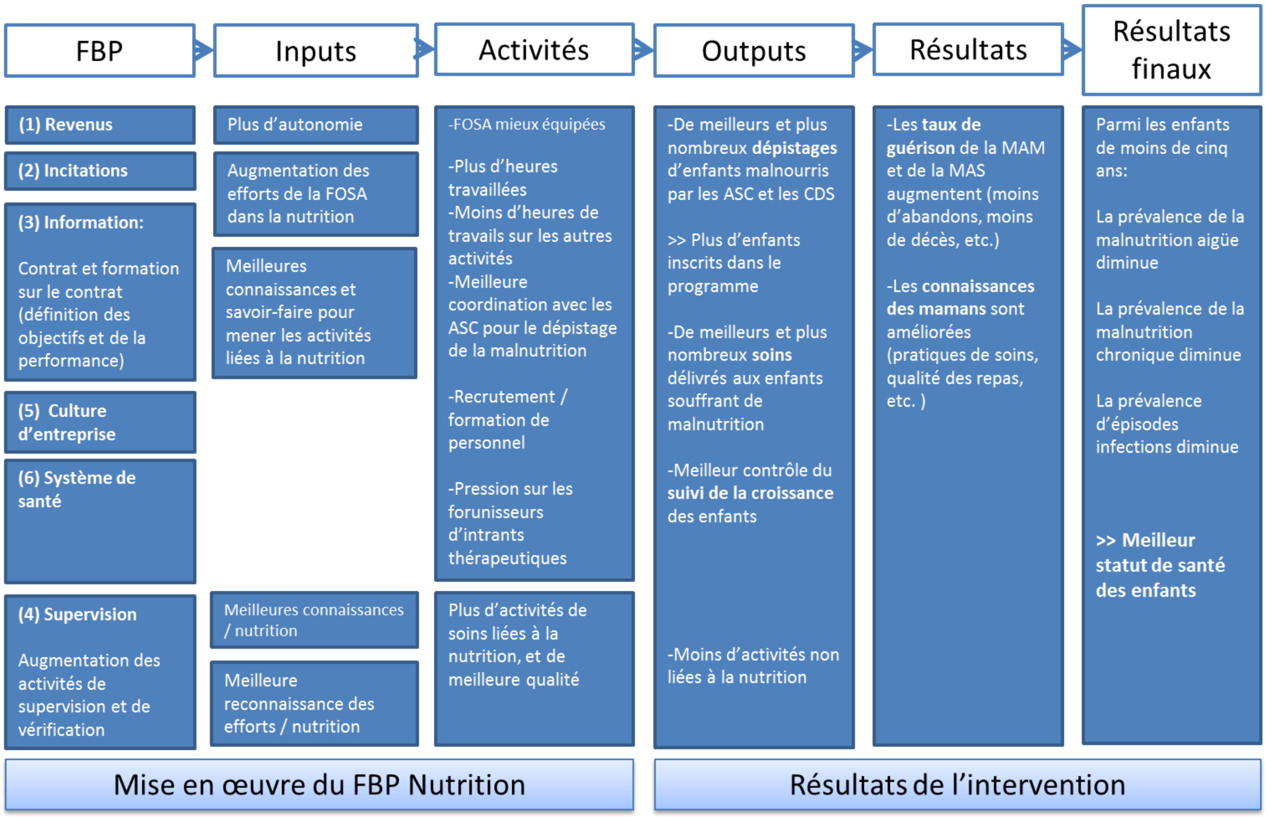

Source: Auteurs.

Tableau 2. Chemins à considérer dans les groupes d'intervention et de contrôle

Chemin :	Groupe d'intervention	Groupe de contrôle
**(1) Revenus**	✓✓✓	✓✓✓[a]
**(2) Incitations**	✓✓✓	
**(3) Information**	✓✓✓	✓[b]
**(4) Supervision**	✓✓✓	✓[c]
**(5) Culture d'entreprise**	✓[d]	
**(6) Système de santé**	✓✓✓	✓[e]

Notes: (a) Les structures de santé du groupe de contrôle recevront une compensation financière additionnelle correspondant à la moyenne pondérée des subventions reçues par le groupe d'intervention. (b) Comme les centres de santé du groupe de contrôle auront été mis au courant de leurs performances en termes de nutrition (lors d'un atelier avec les 90 centres nutritionnels de l'étude), ils pourront ressentir un besoin de formation également, s'ils anticipent en effet une généralisation du FBP Nutrition. (c) Dans un district où il y a des structures appartenant au groupe d'intervention et de contrôle, il est possible que l'équipe de superviseurs du district transfère quelques bonnes pratiques observées dans un centre de santé du groupe d'intervention à un centre de santé du groupe de contrôle. (d) Etant donné que le FBP est déjà en place depuis 2010, on estime que la culture d'entreprise l'est également, au niveau du centre de santé dans son ensemble ; le FBP Nutrition peut cependant agir sur la culture d'entreprise des services spécifiques à la nutrition. (e) Certains effets sur le système de santé pourront affecter les centres de santé du groupe de contrôle.

Questions et objectifs de l'évaluation d'impact

L'objectif principal de l'évaluation d'impact est d'évaluer l'impact de l'introduction des indicateurs nutrition dans le système FBP, sur (i) les taux de malnutrition aigüe et chronique, mesurés au niveau de la communauté, ainsi que (ii) sur la performance des services de prévention et prise en charge de la malnutrition, à travers principalement les taux de guérison de la malnutrition aigüe, mesurés au niveau des centres de santé nutritionnels. La malnutrition étant un problème multisectoriel, l'impact de l'intervention n'est pas garanti. Aussi, la recherche incorpore également des études complémentaires ayant pour objectif d'identifier les causes possibles des succès ou échecs du programme de FBP Nutrition.

Cette étude suit un design d'essais randomisés contrôlés par grappes (ou Cluster-RCT). Les principales questions sont aux niveaux de 1) la communauté, et 2) du centre de santé :

(1) Est-ce que l'introduction des critères axés sur les activités préventives et curatives de lutte contre la malnutrition dans le système FBP actuel se traduit par une réduction des taux de malnutrition aigüe et chronique dans la communauté ? En une meilleure équité des résultats liés à la nutrition ? Est-ce que l'intervention génère des externalités sur d'autres résultats de santé ?
(2) Est-ce que l'introduction des critères axés sur les activités préventives et curatives de lutte contre la malnutrition dans le système FBP actuel se traduit par de meilleurs résultats au niveau des centres de santé, par exemple en une meilleure gestion des cas de malnutrition (meilleurs taux de guérison, périodes de traitement plus courtes, etc.) ?

Perspectives politiques

La Banque Mondiale a recruté une équipe de l'Institut de Médecine Tropicale d'Anvers (IMT) pour soutenir le gouvernement et l'équipe de la BM dans la mise en œuvre de cette évaluation d'impact. Le principal objectif de cette étude est d'aider le MSPLS à (i) évaluer l'introduction graduelle des services de nutrition dans le programme FBP et à (ii) mettre à l'échelle cette expérience. Cette évaluation d'impact doit permettre d'aider le dialogue politique national, mais elle doit également fournir des informations clé pour d'autres pays disposant déjà d'un système FBP et confrontés à des problèmes de malnutrition.

Structure du rapport

La deuxième section de ce rapport décrit la méthodologie utilisée dans l'étude, détaillant ainsi la conceptualisation, la randomisation et l'échantillonnage, ainsi que les variables à utiliser pour l'analyse, les instruments de collecte de donnée et le stockage des données. La troisième section explique dans quelle mesure les données collectées sont représentatives du pays, et si celles-ci sont comparables, i.e. plus ou moins similaires à d'autres enquêtes réalisées au Burundi. La quatrième section décrit les résultats de l'intervention : de la mise en œuvre à l'impact sur les indicateurs de résultat final. La cinquième section discute les résultats et la sixième section propose quelques recommandations liées au fonctionnement du système de santé au Burundi.

2. Méthodologie

2.1 Design de l'étude

Cette étude suit un design d'essais randomisés contrôlés par grappes (ou Cluster-RCT) avec le centre de santé[2] comme premier niveau d'échantillonnage et les sous-collines (unité administrative au Burundi) comme second niveau. Les 90 centres de santé sélectionnés ont été alloués aléatoirement soit à un groupe de contrôle (45) soit à un groupe d'intervention (45). Autour de chacun des centres de santé, six sous-collines sont sélectionnées de façon aléatoire ; au sein de chaque sous-colline, 12 enfants de 6-23 mois et leurs ménages sont enquêtés.

2.2 Randomisation et taille d'échantillon

Au niveau de la communauté

La taille de l'échantillon a été calculée sur base de la plus petite différence dans le résultat principal qui peut être considéré comme significative dans le groupe d'intervention comparé au groupe de contrôle - ici, on a considéré une réduction du taux de malnutrition aigüe parmi les enfants de 6-23 mois[3] de 25%. On a fait l'hypothèse que l'intervention résulterait en une réduction du taux de malnutrition aigüe parmi ces enfants de 10% à 7.5% (selon l'enquête EDS 2010), et en considérant que 65 enfants âgés de 6-23 mois seraient enquêtés dans l'aire de responsabilité de chaque centre de santé, 90 centres de santé sélectionnés aléatoirement étaient nécessaires pour une erreur de type alpha de 5%, une erreur de type beta de 20% et une corrélation intra-grappes de 0.25[4]. Nous avons augmenté le nombre d'enfants à enquêter par centre de santé à 72 afin de permettre que certaines données soient manquantes ou incomplètes. Ainsi le nombre total d'enfants de 6-23 mois et leurs ménages à enquêter pour chaque vague d'enquête était fixé à 6,480 (référence de 2014 et suivi de 2017). Les données collectées lors de la première vague en 2014 ont révélé un taux de malnutrition aigue de 6% (i.e. inférieur à celui des EDS 2010 utilisé pour les calculs ci-dessus ; il s'agissait d'ailleurs probablement d'un effet saisonnier) mais une corrélation intra-grappes bien moindre à ce qui avait été supposé lors du calcul initial (ICC=0.007, au lieu de ICC=0.25) : en prenant ces données en compte, la puissance de calcul d'une réduction du taux de malnutrition aigüe de 25% est réduite à 60% ; elle demeure à 80% si on observe une réduction du taux de malnutrition aigue d'un tiers. Aussi, pour la deuxième vague d'enquêtes, il a été décidé de maintenir le nombre d'enfants à 6,480.

---

[2] Cette recherche au niveau de la FOSA se concentre sur les centres de santé (et non les hôpitaux), car le niveau de soins primaires est un point d'engorgement connu en ce qui concerne le diagnostic et le traitement de la malnutrition aigüe. Une évaluation séparée pourrait être réalisée sur les programmes de SST (i.e. au niveau de l'hôpital) au Burundi, mais étant donné que ces unités sont peu nombreuses, et que les patients de ces unités sont également peu nombreux, les résultats n'auraient pas une puissance de calcul suffisante en comparaison des possibilités de tailles d'échantillonnage à travers des enquêtes dans les centres de santé.

[3] On se concentre sur ce groupe d'âge car c'est là qu'on suppose que l'impact sera le plus important. C'est aussi le groupe d'âge pour lesquels les mères sont les plus motivées pour respecter le programme.

[4] Calculé selon la formule de Hayes & Bennett (1999) : c (nombre de grappes) = $1+ (z_{\alpha/2} + z_{\beta})^2[\pi_0 (1-\pi_0)/n + \pi_1(1-\pi_1)/n+ k_m^2 (\pi_0^2+\pi_1^2)]/(\pi_0-\pi_1)^2$, avec n=nombre d'enfants par grappe, et k_m le coefficient de variation entre grappes au sein des paires en l'absence d'intervention. K_m est fixé à 0.25, ce qui est plutôt conservateur comme estimation en l'absence d'information détaillée sur le Burundi (les données EDS ne sont pas examinées au niveau du centre de santé).

La sélection des centres de santé invités à participer à l'étude a été réalisée par sélection aléatoire simple (sur base d'un algorithme aléatoire sous STATA), parmi les 193 centres de santé éligibles, i.e. les centres de santé fournissant les services de prise en charge de la malnutrition aigüe modérée et de la malnutrition aigüe sévère sans complications (appelés respectivement les services SSN et STA ; liste obtenue auprès du PRONIANUT et de l'UNICEF en 2013). Les 90 centres de santé sélectionnés ont ensuite été appariés selon des paramètres essentiels d'organisation et de fonctionnement en relation avec les résultats (activité SSN, volume d'activité, population dans l'aire de santé, taux de guérison parmi les enfants malnutris) tels que mesurés durant l'enquête de référence. Au sein de chacune de ces 45 paires, un centre a été alloué au groupe d'intervention tandis que l'autre l'était au groupe de contrôle ; l'allocation aléatoire a été effectuée sous forme de loterie organisée durant un atelier en décembre 2014.

Un système de sélection aléatoire simple devrait être utilisé pour sélectionner les enfants à enquêter dans les aires de responsabilité des 90 centres de santé. Cependant, une telle approche est difficile à mettre en œuvre, car cela peut être très demandeur en termes de ressources. L'alternative choisie a été de sélectionner des grappes d'individus, i.e. des enfants vivant proches les uns des autres. Six grappes de 12 enfants ont été sélectionnées aléatoirement, dans l'aire de responsabilité de chaque centre de santé. Cela a pu être réalisé sans étendre la taille de l'échantillon étant donné le coefficient de correlation intra-classe (ou ICC) élevé considéré dans le calcul.

Au niveau des centres de santé

Afin de mesurer la performance des services nutritionnels des centres de santé (et notamment de calculer les taux de guérison), 12 fiches individuelles de suivi sont sélectionnées aléatoirement et retranscrites par type de service (SSN ou STA) et par centre de santé. Ce nombre a été calculé sur base de la plus petite différence dans le résultat principal qui peut être considéré comme significative dans le groupe d'intervention comparé au groupe de contrôle - ici, on considère une hausse du taux de guérison de 80% à 90%, avec une erreur de type alpha de 5%, une erreur de type beta de 20%, et une corrélation intra-grappes de 0.15 (selon la formule de Hayes et Bennett, 1999 ; Kaiser et al. 2006).

Selon les données collectées lors de la première vague d'enquêtes en 2014, les taux de guérison de malnutrition aigüe modérée et sévère sont respectivement de 80% (N=628) et 85% (N=665). Ces chiffres sont proches de notre hypothèse de départ, mais avec un nombre inférieur d'observations. En effet, plusieurs dossiers médicaux manquaient ou étaient pauvrement documentés, la conséquence étant que nous étions dans l'incapacité d'avoir le résultat du programme, i.e. de savoir si le cas de malnutrition aigüe avait été guéri ou pas. De plus, il a été constaté que la qualité de la documentation ainsi que la performance de services sont fortement liées au niveau même des centres de santé. Ainsi pour les SSN, l'ICC équivaut à 0.41 (basé sur les observations au niveau de 26 centres de santé pour lesquels on a eu des informations provenant de 12 dossiers médicaux), ce qui fait chuter la puissance de notre échantillon à 60% pour une augmentation du taux de guérison passant de 80% à 95%. Pour la deuxième vague d'enquêtes, augmenter le nombre de dossiers médicaux SSN à collecter a été envisagé. Cela étant, le nouveau protocole national de prise en charge de la malnutrition aigu publié fin 2014 n'incitait plus les agents de santé à tenir des fiches individuelles de suivi pour le traitement de la MAM (il n'y avait plus de fiche formelle pour ce service) ; de plus, il a été observé une très faible activité de ce service SSN

en 2015 et 2016 dans le groupe de contrôle, ce qui laissait présager de toute façon un fort biais de collecte ; aussi, il a été décidé de maintenir le nombre de fiches à retranscrire au niveau de l'enquête de 2014. En ce qui concerne les STA, l'ICC observé à partir des données de 2014 était de 0.06. Aussi, on a maintenu le nombre de fiches à retranscrire pour ce service également.

2.3 Variables pour l'analyse des données

Au niveau de la communauté

L'étude se focalise sur la prévalence de la malnutrition aigüe parmi la population des enfants âgés de 6 à 23 mois comme résultat final. D'autres variables sont considérées. Les indicateurs de résultats à collecter et à analyser sont décrits dans le tableau suivant:

Tableau 3. Variables de résultats à collecter et analyser

Principal résultat	- Prévalence de la malnutrition aigüe (z-score du poids pour taille<-2)<br>- Z-scores du poids-pour-taille et de taille-pour-âge, et périmètre brachial<br>- Prévalence du retard de croissance (z-score de taille-pour-âge<-2)
Résultats secondaires	- Prévalence des épisodes infectieux (diarrhée, infection des voies respiratoires supérieures, fièvre)

Source: Auteurs.

Au niveau des centres de santé

Au niveau du centre de santé, nous souhaitons comprendre si l'introduction de critères nutrition dans le programme de FBP existant apportera de meilleurs résultats au niveau du centre de santé, i.e. une meilleure prise en charge des cas de malnutrition (taux de guérison, durée de périodes de traitement, etc.). Les indicateurs de résultats à collecter et à analyser au niveau de la structure sont indiqués dans le tableau suivant :

Tableau 4. Variables de résultats à analyser au niveau de la structure de soins

Résultat principal	- Taux de guérison de la MAM et de la MAS
Résultats secondaires	- Durée de traitement des cas de MAM et de MAS<br>- Taux d'abandons<br>- Taux de rechute<br>- Pourcentage de cas suivis régulièrement et référés au centre de santé

Source: Auteurs.

2.4 Instruments de collecte et d'assurance qualité

Les instruments décrits ci-dessous correspondent aux outils utilisés lors des enquêtes de référence en 2014-2015 et pour l'enquête finale ayant eu lieu en 2017. L'ensemble des outils est décrit de façon détaillées dans le rapport de référence ; nous fournissons ici une description générale synthétique ainsi que les éléments ayant connu des changements par rapport à 2014.

2.4.1 Enquêtes au niveau des centres de santé

L'organisation des deux enquêtes (baseline et finale) au niveau des centres de santé a été confiée à l'Institut National de Santé Publique (INSP). Pour chacune des deux enquêtes, 24 enquêteurs, deux

superviseurs et un gestionnaire ont été mobilisés pour la formation et l'enquête sur le terrain, sur une période couvrant au total quatre semaines en septembre et octobre 2014 (cf. rapports d'enquête, INSP).

Quatre domaines sont à évaluer à partir de l'enquête au niveau des centres de santé :

- L'évaluation des dossiers médicaux individuels issus des services de nutrition
- Les aspects organisationnels liés à la fourniture des soins de santé
- La qualité des services de santé
- Les connaissances et compétences des agents de santé

Ceci était évalué à travers un ou plusieurs questionnaires (suivant le schéma qui suit).

Figure 3. Structure des questionnaires de l'enquête au niveau des centres de santé

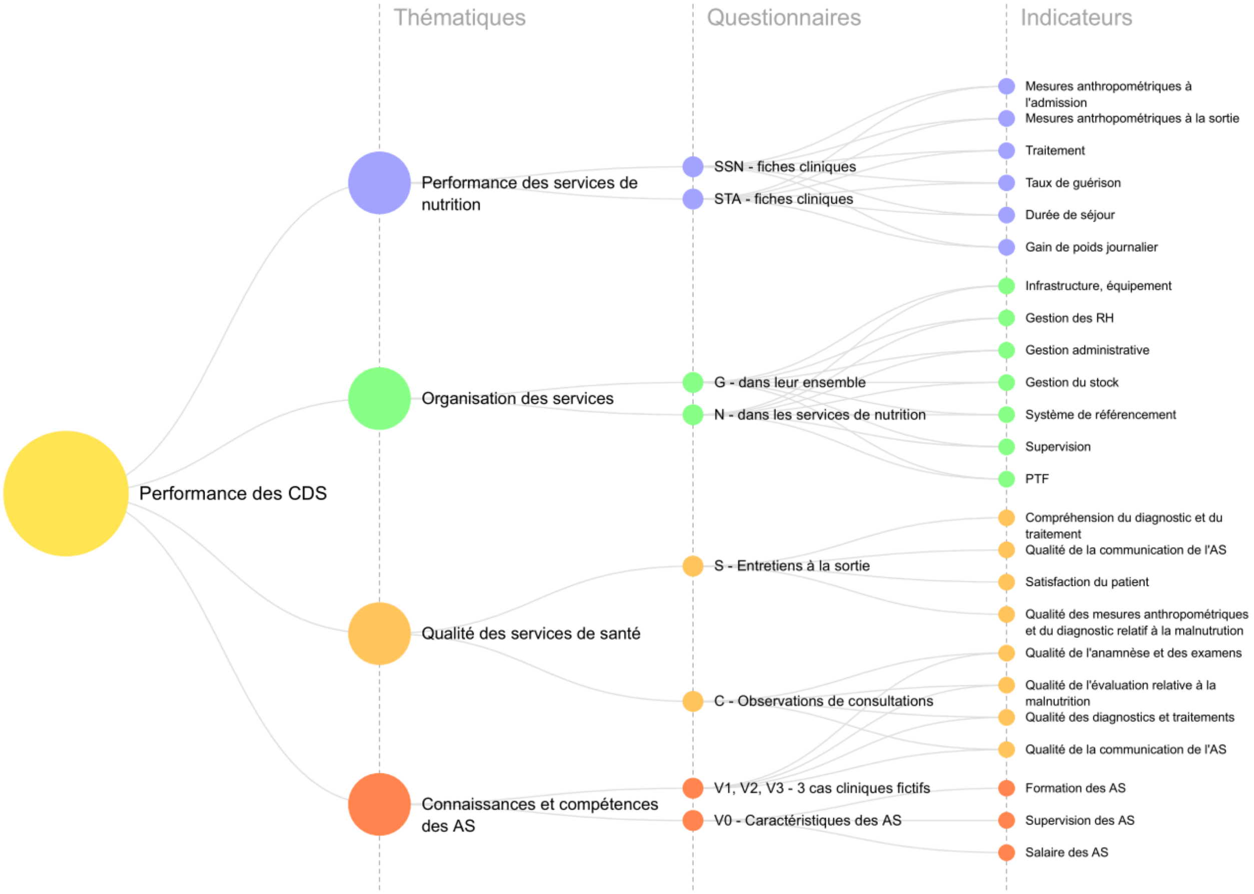

(A) Evaluation des dossiers médicaux individuels issus des services de nutrition

<u>Questionnaire SSN – Retranscription des fiches individuelles de suivi SSN</u>

L'objectif principal de cet instrument est d'évaluer les indicateurs de performance des SSN au Burundi ; un objectif secondaire est d'évaluer la qualité de la documentation relative aux patients. Les données collectées permettent de calculer les taux de guérison, la durée moyenne des traitements, et autres

indicateurs de performance du SSN, afin de pouvoir répondre à la question principale de l'étude. Les données anthropométriques à l'admission et à la sortie du programme sont collectées afin d'évaluer si les critères d'admission et de sortie (pour guérison ou référence) sont respectés.

Lors de l'enquête de baseline, les services SSN n'étaient pas fonctionnels en 2014 à cause d'une rupture d'approvisionnement des intrants nutritionnels (sauf dans les provinces de Ruyigi et Rutana) ; la période considérée pour les fiches individuelles a alors glissé vers Mars-Septembre 2013, plutôt que Février-Août 2014. Egalement, dans de nombreux cas, il n'y avait pas de fiches individuelles au centre de santé, ou alors elles étaient mal remplies. Dans ce cas, les enquêteurs devaient chercher l'information à partir des registres du service SSN. Néanmoins, généralement, les registres ne fournissent pas l'information sur les traitements systématiques ou nutritionnels administrés aux patients ; mais normalement les informations sur les mesures anthropométriques à l'admission et à la sortie ainsi que sur le résultat du programme y sont reportées.

Pour l'enquête finale, afin de connaître la source des données retranscrites et de faciliter la retranscription par les enquêteurs, trois types de questionnaires de retranscription étaient proposés (correspondant à soit la fiche issue du protocole de 2010, soit à des feuilles volantes reprenant les informations de la fiche de 2010, soit le registre). Aussi, de même que pour l'enquête de baseline, les services SSN étaient souvent non fonctionnels à cause de ruptures d'approvisionnement, et les agents enquêteurs ont alors retranscrit les fiches disponibles sur la période de six mois la plus récente.

Questionnaire STA - Retranscription des fiches individuelles de suivi STA

L'objectif principal de cet instrument est d'évaluer les indicateurs de performance des STA au Burundi ; un objectif secondaire est d'évaluer la qualité de la documentation relative aux patients. Les données collectées permettent de calculer les taux de guérison, la durée moyenne des traitements, et autres indicateurs de performance du STA, afin de pouvoir répondre à la question principale de l'étude. Les données anthropométriques à l'admission et à la sortie du programme sont collectées afin d'évaluer si les critères d'admission et de sortie (pour guérison ou référence) sont respectés.

Lors de l'enquête de baseline, dans de nombreux cas, il n'y avait pas de fiches individuelles au centre de santé, ou alors elles étaient mal remplies. Dans ce cas, les enquêteurs devaient chercher l'information à partir des registres du service STA. Néanmoins, généralement, les registres ne fournissent pas l'information sur les traitements systématiques ou nutritionnels administrés aux patients ; mais normalement les informations sur les mesures anthropométriques à l'admission et à la sortie ainsi que sur le résultat du programme y sont reportées.

Pour l'enquête finale, afin de connaître la source des données et de faciliter la retranscription par les enquêteurs, trois types de questionnaires de retranscription étaient proposés (correspondant à soit la fiche issue du protocole de 2010, soit la fiche issue du protocole de 2014, soit le registre).

(B) Aspects organisationnels des services de santé

Questionnaire G – Aspects organisationnels du centre de santé

L'objectif principal de ce questionnaire est de collecter des informations sur les aspects organisationnels du centre de santé. Ces variables plus contextuelles seront notamment utilisées pour capturer les facteurs confondants dans les analyses statistiques.

Pour plus d'efficacité et d'assurance qualité, le questionnaire a été modifié pour l'enquête finale. Ainsi, les sections ont été réduites. De même, la section sur les PTF a été supprimée : elle était mal comprise par les répondants et longue à administrer (plus de 30 minutes) et au final elle ne donnait que peu d'informations exploitables (nombre de PTF). Par contre, la section sur les ressources humaines (toujours sur papier) comprend désormais des informations sur les formations et rôles des employés dans la nutrition.

Par ailleurs, lors de l'enquête de baseline, il a été noté que certaines réponses aux questionnaires administrés aux titulaires des CDS n'étaient pas toujours contre-vérifiées par les enquêteurs ; aussi, les questions ont été formulées différemment, de sorte que les enquêteurs devaient vérifier directement plutôt que de poser la question.

Questionnaire N – Aspects organisationnel des services liés à la malnutrition

L'objectif principal de ce questionnaire est de collecter des informations sur les aspects organisationnels des services de prévention et de prise en charge de la malnutrition. Cela a pour but de comprendre l'environnement dans lequel les activités de détection de la malnutrition, promotion de la croissance et prise en charge de la malnutrition sont réalisées. Ces variables seront également utilisées pour capturer les facteurs confondants dans les analyses statistiques.

Pour plus d'efficacité et d'assurance qualité, le questionnaire a été modifié pour l'enquête finale. Ainsi, les sections ont été réduites ; de même, la section sur le staff a été supprimée et remplacée par la grille complétée dans le questionnaire G. Aucune section n'est désormais à remplir sur papier.

Par ailleurs, lors de l'enquête de baseline, il a été noté que certaines réponses aux questionnaires administrés aux responsables de service nutrition n'étaient pas toujours contre-vérifiées par les enquêteurs ; aussi, les questions ont été formulées différemment, de sorte que les enquêteurs devaient vérifier directement plutôt que de poser la question.

(C) Qualité des services de santé

Questionnaire C – Observation de consultations

Le principal objectif du questionnaire C est d'observer six consultations curatives d'enfants de 6-59 mois, dans les centres de santé. L'objectif est d'évaluer la qualité des consultations, et plus spécifiquement en relation avec le respect du manuel de prise en charge intégrée des maladies de l'enfants (PCIME, WHO, 2014) en place au Burundi, et relativement à l'évaluation nutritionnelle de l'enfant.

Questionnaire S – Entretien à la sortie

Suite à cette consultation observée, il était demandé aux enfants et leurs accompagnants de répondre à un entretien à la sortie, administré par un autre enquêteur. Le premier objectif de ce questionnaire était d'obtenir des informations de la part des accompagnants sur leur opinion sur le contenu et la qualité de la consultation, et d'évaluer leur compréhension de l'information qui leur était donnée. Le second objectif était de documenter les mesures anthropométriques des enfants prises par les agents du centre de santé, puis de répéter ces mesures par les enquêteurs eux-mêmes.

Pour plus d'efficacité, le questionnaire a été modifié pour l'enquête finale. Ainsi, une grande partie des questions évaluant le niveau de satisfaction des mamans avec la consultation et quelles étaient leurs suggestions pour améliorer les services, initialement addressées sur papier, ont été supprimées. Elles ont été remplacées par quelques questions encodées sur ODK, à choix multiple.

Dans les cas où les mesures anthropométriques prises par l'enquêteur indiquaient un problème de malnutrition chez l'enfant, l'enquêteur devait raccompagner l'enfant vers l'AS qui l'avait eu en consultation pour discuter du résultat[5]. Ce processus était facilité par un message automatique apparaissant sur le smartphone de l'enquêteur une fois que toutes les mesures étaient saisies. La responsabilité ultime pour prescrire un traitement revenait à l'AS.

(D) Connaissances et compétences des agents de santé

Questionnaire V – Vignettes cliniques

L'objectif principal des vignettes est d'évaluer les connaissances et compétences des agents de santé responsables des consultations et services pédiatriques dans les centres de santé.

Une description détaillée des vignettes peut être trouvée dans le rapport sur les enquêtes de référence de 2014. Le Tableau 37 en annexe propose une synthèse des trois vignettes cliniques proposées aux agents de santé, en synthétisant les signes, diagnostics et plans de prise en charge attendus pour chacun des trois cas.

2.4.2 Enquête au niveau de la communauté

L'organisation des enquêtes au niveau de la communauté a été confiée à l'Institut de Statistiques et d'Etudes Economiques du Burundi (ISTEEBU). Au total, pour chaque vague, 72 enquêteurs, six superviseurs de terrain et trois gestionnaires ont été mobilisés pour la formation et l'enquête sur le terrain. L'enquête de référence s'est déroulée du 8 décembre 2014 au 11 janvier 2015 (cf. rapport d'enquête, ISTEEBU, 2015) ; l'enquête finale s'est déroulée du 16 mars au 19 avril 2017 (cf. rapport d'enquête, ISTEEBU, 2017) .

[5] Il est possible que cette pratique de référer à nouveau vers les agents de santé les cas de malnutrition détectés lors de l'entretien à la sortie ait pu engendrer un certain biais dans l'activité des agents de santé (ceux-ci se sentant observés et jugés sur les aspects de prévention et prise en charge de la malnutrition peuvent avoir changé de comportement en consultation). Néanmoins, ce biais de comportement devrait être le même dans les groupes de contrôle et d'intervention, n'entrainant donc pas de biais concernant l'estimation de l'impact. Par ailleurs, force est de constater à travers les observations des consultations que les activités des agents de santé n'ont pas été fort influencées en ce qui concerne la prévention et l'identification de la malnutrition (cf. résultats).

Le principal objectif des questionnaires était d'évaluer le taux de malnutrition aigüe dans la communauté parmi les enfants de 6-23 mois. Les objectifs secondaires sont d'évaluer si ces taux ont une relation avec le statut socio-économique du ménage, le statut de santé de l'enfant, la sécurité alimentaire.

Le contenu de chaque section et la mise en œuvre de l'enquête sont décrits de façon détaillée dans le rapport sur les enquêtes de référence de 2014.

2.5 Analyse

L'analyse d'impact est évaluée à travers l'analyse économétrique de modèles en double différence (Lechner 2011). L'estimateur varie selon les caractéristiques des variables : les MCO sont utilisés pour les variables continues, l'estimateur logistique pour les variables binaires.

2.6 Stockage, gestion et politique d'accès aux données

Le stockage et la gestion des données sont la responsabilité de l'ISTEEBU et l'INSP. L'accès est limité à l'ISTEEBU, l'INSP et l'IMT durant la période de l'analyse des données de l'enquête de référence. Les données pourront être accessibles par des parties tierces sur demande (et dossier d'application) ; les droits d'accès seront gérés par la Banque Mondiale et le gouvernement du Burundi.

3. Représentativité de l'échantillon et validité externe de l'étude

Ce qui suit dans cette section est largement issu du résumé exécutif du rapport de baseline – pour plus de détails, se référer au rapport de baseline.

3.1 Validité externe

Au niveau des centres de santé. De par la sélection des 90 centres de santé de l'étude, l'échantillon couvre l'ensemble du territoire burundais, excepté la province de Bujumbura mairie. Les données collectées ont pu être comparées à des données provenant d'autres enquêtes d'envergure nationale. Ainsi, les caractéristiques générales des centres de santé ici observées sont relativement similaires avec celles observées lors de l'enquête FOSA 2013 réalisée par l'INSP (réf. MSPLS et al. 2014). Aussi, les données relatives aux indices de performance des services SSN et STA ont été validées après comparaison avec les statistiques collectées par le PRONIANUT pour l'année 2012.

Au niveau de la communauté. Le taux de malnutrition chronique observé parmi les enfants de 6-23 mois est relativement similaire à celui observé lors de l'enquête démographie et santé 2010 (EDS 2010). Par contre, le taux de malnutrition aigüe a chuté entre l'enquête EDS de 2010 (10% pour cette tranche d'âge) et l'enquête ici présentée (6%). Il se peut qu'en quatre années la situation se soit améliorée, mais on doit également garder à l'esprit que la malnutrition aigüe est un évènement de court terme, et que des variations relativement importantes peuvent être observées selon la saison. Or, l'enquête EDS 2010 couvrait une des périodes de soudure (octobre-novembre), tandis que l'enquête ici présentée était réalisée en décembre et début janvier, ce qui correspond à une période de récolte, où la nourriture est plus disponible. Enfin, les statistiques relatives à la sécurité alimentaire ont pu être comparées avec les données collectées dans les provinces de Cankuzo et Ruyigi en octobre 2012 dans le cadre d'une évaluation de programme de prévention de la malnutrition (réf. Projet FANTA), et se sont avérées relativement similaires.

3.2 Validité interne

Puissance de calcul ex-post au niveau des centres de santé. La taille de l'échantillon de fiches individuelles de suivi à collecter avait été calculée sur la plus petite différence de taux de guérison qui pouvait être considérée comme significative d'un point de vue de santé publique ; on avait alors fait l'hypothèse que l'intervention FBP Nutrition pourrait permettre une augmentation du taux de guérison de la malnutrition aigüe de 80% à 90%, en considérant une erreur de type alpha de 5%, un pouvoir statistique de 80% et un ICC de 0.15. Selon les données collectées, les taux de guérison de malnutrition aigüe modérée et sévère sont respectivement de 80% (N=628) et 85% (N=665), ce qui est proche de notre hypothèse de départ, mais avec un nombre inférieur d'observations (étant donné la faible qualité de documentation). Par ailleurs, étant donné le niveau de corrélation intra-centre de santé plus important que prévu dans le service SSN, la puissance de calcul pour ce service tombe de 80% à 60%. Concernant le service STA, le niveau de corrélation intra centre de santé est plus faible, et la puissance de calcul demeure à 80% si on suppose que l'intervention permettra une augmentation du taux de guérison de MAS de 85% à 95%. Notons tout de même qu'il s'agit ici de calculs hypothétiques, et que la puissance de calcul finale dépendra fortement de la qualité de documentation des fiches individuelles de suivi en 2016, avant l'enquête finale.

Puissance de calcul ex-post au niveau de la communauté. La taille de l'échantillon d'enfants à enquêter avait été calculée sur base du taux de malnutrition aigüe dans ce groupe d'âge de 6-23 mois au Burundi. L'hypothèse de départ était de 10%, et était basée sur les données de l'enquête démographie et de santé 2010 (EDS 2010). Les données collectées ici observent en fait un taux de malnutrition aigüe de 6%, mais une corrélation intra-grappes bien moindre à ce qui avait été supposé lors du calcul initial. En prenant ces données en compte, la puissance de calcul d'une réduction du taux de malnutrition aigüe de 6% à 4.5% est réduite à 60% ; elle demeure à 80% si on observe une réduction du taux de malnutrition aigüe plus importante, à savoir de 6% à 4%.

Comparabilité des deux groupes. Aucune différence significative n'a été observée entre les deux groupes (contrôle et intervention) en ce qui concerne les variables d'intérêt primaire pour cette recherche, qu'il s'agisse des données au niveau des centres de santé ou au niveau des ménages. De plus, la plupart des variables calculées ont été testées pour la différence entre les groupes de traitement et de contrôle, et la majorité des différences s'avèrent non significatives, ce qui suggère fortement que les deux groupes sont similaires, et donc comparables[6].

[6] Sur les 386 variables testées pour vérifier la comparabilité des deux groupes lors de la baseline (enquêtes centres de santé et ménages), on observait une différence significative ($p<0.05$) pour 26 (6.7%) d'entre elles.

4. Résultats de l'évaluation d'impact

4.1 Données analysées

Dans cette section, nous utilisons plusieurs sources de données.

Les données principalement utilisées sont celles issues des enquêtes. L'enquête de référence s'est déroulée du 21 septembre au 7 octobre 2014, et l'enquête finale du 9 au 24 février 2017. Le tableau suivant donne, pour chaque type de questionnaire, le nombre de questionnaires collectés au total, le taux de couverture de l'échantillon ainsi que l'explication de l'écart.

Les données de routine du projet FBP Nutrition permettent à la fois d'évaluer l'effectivité de la mise en œuvre et de croiser ces informations opérationnelles avec les observations issues des enquêtes. Nous avons eu recours aux données mensuelles et par centre de santé de rapport, vérification et validation des activités par indicateur (données sur le volume d'activités validées, cf. Tableau 61 en annexe) ; il s'agit des quatre indicateurs suivants : promotion et suivi de la croissance, dépistage et référence de cas de malnutrition aigue, dépistage et prise en charge jusqu'à guérison de cas de MAM, dépistage et prise en charge jusqu'à guérison de cas de MAS. Nous avons également eu recours aux données trimestrielles par centre de santé sur les évaluations qualitatives (cf. Tableau 60, annexe). Toutes ces données sont disponibles sur toute la période 2015-2016 et pour le groupe d'intervention uniquement.

Tableau 5. Taux de couverture pour chacun des outils de collecte de l'enquête CDS

outil	Nombre attendu	Nombre collecté en 2014	Taux de couverture en 2014	Explication en 2014	Nombre collecté en 2017	Taux de couverture en 2017	Explication en 2017
**G**	90	90	100%		90	100%	
**N**	90	90	100%		90	100%	
**SSN**	90 x 12 = 1080	971	90%	Pas suffisamment de dossiers dans 14 CDS	674	62% (C : 29% ; T : 96%)	Service fermé dans beaucoup de CDS contrôle
**STA**	90 x 12 = 1080	963	89%	Pas suffisamment de dossiers individuels dans 17 CDS	1048	97%	Pas suffisamment de dossiers individuels dans 4 CDS.
**C**	90 x 6 = 540	515	95%	Pas suffisamment de consultations dans 10 CDS	529	98%	Pas assez d'enfants trouvés en consultation dans six CDS
**S**	90 x 6 = 540	512	95%	idem + 3 perdus	529	98%	idem
**V**	90 x 2 = 180	145	81%	Un seul AS dans 35 CDS	169	94%	Un seul AS dans 11 CDS

Source: Rapports d'enquête, INSP.

4.2 Mise en œuvre de l’intervention

Cette sous-section décrit la mise en oeuvre de l’intervention FBP Nutrition dans le groupe d’intervention, ainsi que dans le groupe de contrôle. Le calendrier de cette mise en œuvre est synthétisé par la Figure 4 page 35 ; la Figure 5 page 36 donne une vue d’ensemble de la mise en œuvre et des barrières observées par niveau et par groupe (intervention ou contrôle). Enfin le Tableau 62 en annexe fournit une vision de la mise en œuvre relative à la théorie du changement.

Formations

Formation à l’intervention

En préparation à l’intervention FBP Nutrition, la CTN a organisé et réalisé des formations à l’endroit des titulaires des 45 CDS Intervention (1 jour), des responsables hôpitaux offrant les SSt (1 jour) ainsi que des coordonnateurs des CPVV. Cette formation s’est déroulée du 2 au 5 Décembre 2014 (cf. rapport de l’atelier pour détails).

A l’endroit des CDS ‘primaires’ et des hôpitaux

La formation des titulaires des CDS nutritionnels du groupe d’intervention et responsables des hôpitaux s’est focalisée sur les éléments suivants : (1) les modalités pratiques de mise en œuvre de l’intervention FBP Nutrition, (2) les indicateurs quantité/ qualité au niveau des CDS et (3) les indicateurs quantité au niveau communautaire.

Au cours de la mise en œuvre, une autre séance de formation sur l’intervention a eu lieu en septembre 2015, en préparation au démarrage de la composante communautaire du FBP/ Nutrition.

A l’endroit des vérificateurs

En ce qui est des CPVV, leur formation s’est focalisée sur (1) les modalités pratiques de mise en œuvre de l’intervention FBP Nutrition, (2) les indicateurs quantité au niveau des CDS, hôpitaux et communautaire ainsi que (3) sur le système de vérification au niveau communautaire, CDS et Hôpitaux en même temps que les principaux outils y relatifs.

Concernant la vérification qualitative, les BPS et les ONGs d’accompagnement chargées de cette activité dans le cadre du FBP initial ont été formés sur la grille d'évaluation qualité FBP Nutrition en Juillet 2015.

A l’endroit des superviseurs

Aucune formation n’a été réalisée à l’endroit des superviseurs des niveaux districts et provinces.

A l’endroit des CDS secondaires

Pour les CdS référant pour Malnutrition aux CDS du groupe Intervention, ces derniers devaient répliquer la formation reçue ; en pratique, cela n’a pas été réalisé.

A l’endroit des agents de santé communautaires

Cette formation concernait en premier lieu les 45 responsables des GASC des différents CDS intervention. Ceux-ci ont été formés au cours de la dernière semaine du mois de Septembre 2015, par la

CT-FBP et le PRONIANUT. La durée de la formation était d'une journée mais répliquée dans trois provinces différentes ; à savoir Gitega, Ngozi, Kayanza avec une audience différente à chaque fois suivant la proximité des provinces concernées[7]. Les objectifs de la formation étaient les suivants :

- ✓ Conscientiser les ASC sur le fardeau que cause la malnutrition sur l'état de santé globale des enfants au Burundi ;
- ✓ Former les ASC sur le dépistage de la malnutrition aigüe ;
- ✓ Former les ASC sur les bonnes pratiques alimentaires pour la prévention de la malnutrition dans toutes ses formes ;.
- ✓ Former les ASC sur tout ce qui concerne le projet pilote du FBP-Nutrition à savoir : le contexte général de l'étude d'impact, ses objectifs, les activités contractualisées, les outils de rapportage, le système de vérification, le montant attribué aux activités sous contrat ainsi que les conditions de payement ;
- ✓ Former les ASC quant au rôle qui est le leur dans ce projet ainsi que la manipulation des outils nécessaires pour son bon déroulement.

Formations ayant eu lieu en parallèle

Formation spécifique SSN

Depuis Décembre 2014, un nouveau protocole de prise en charge de la Malnutrition est entré en vigueur. Dans celui-ci, le traitement de la MAM est relégué à l'annexe et est différent de celui proposé dans le protocole de 2010**.** En effet**,** le nouveau traitement de la MAM, tel que décrit dans le nouveau protocole, est à base de Plumpy Sup ; il n'est plus question de CSB, huile et sucre. Selon le PRONIANUT, dès le début de l'intervention, le Plumpy Sup était uniquement à utiliser dans les deux provinces couvertes par le PAM (Rutana et Ruyigi) et par les CDS qui ne participaient pas à l'étude d'impact ; pour les autres, ils pouvaient utiliser le traitement à base de CSB, sucre et huile sur la période de l'intervention.

Ainsi, il n y a pas eu de formation/recyclage sur le SSN depuis que le processus de révision du protocole national de prise en charge de la malnutrition de 2010 était enclenché (dès 2014). Seules les provinces encore appuyées par le PAM (Ruyigi, Rutana) ont bénéficié des formations sur le SSN en 2015. Pour les autres, il a été réalisé dans chaque district un petit briefing de la part du PRONIANUT auprès des CDS sur le SSN lors de la supervision de la livraison des intrants début janvier 2016.

Il avait été relevé par ailleurs des demandes des vérificateurs des CPVV d'être formés sur les indicateurs à vérifier. Cependant, aucune des parties prenantes (CT_FBP/PRONIANUT) n'a pu organiser de formation à l'endroit de ces acteurs. Aussi, il était convenu qu'un ou deux vérificateurs de chaque CPVV devaient participer dans le briefing du PRONIANUT sus-cité une fois le tour du district du chef-lieu de la province.

---

[7] Il est à noter que certains représentants des GASC et des CDS de la province Bururi n'ont pas pu participer à la formation suite à une communication tardive. Néanmoins, ils ont pu bénéficier d'une séance de rattrapage quelques jours plus tard organisée par le bureau de district.

Formation spécifique STA

Des formations/recyclages des prestataires sur le STA ont été réalisées en 2015 par le PRONIANUT , sur le nouveau protocole de 2014 (la procédure étant la même que dans le protocole de 2010 et dans celle motivée dans le FBP Nutrition).

Formation relative à l'achat d'intrants

Concernant le problème des intrants diététiques pour le SSN (cf. section relative ci-dessous), il est à noter qu'aucune formation n'a été réalisée depuis que la possibilité d'achats d'intrants SSN ait été obtenue.

Contrats FBP

Contrats des CDS 'primaires' et des hôpitaux

Les contrats du FBP existant des CDS objet d'étude d'impact FBP Nutrition ont été adaptés en y ajoutant un article spécifique relatif à l'intervention FBP Nutrition. A ce titre, un article spécifique intitulé : « des modalités particulières relatives à la mise en œuvre du FBP Nutrition » a été ajouté au contrat initial. Cet article précise pour le CDS intervention (1) les indicateurs nutrition contractualisés en plus des indicateurs du PMA du FBP, (2) le système de leur évaluation quantitative et qualitative ainsi que (3) le rôle du CDS dans la vérification des prestations quantitatives nutritionnelles réalisées au niveau communautaire par les GASC. L'article renvoie à la fin à la « *note technique relative à l'intégration de la nutrition dans la stratégie nationale de Financement Basé sur la Performance* **»** pour les modalités pratiques de la mise en œuvre de l'intervention FBP et Nutrition. Il est à noter que celle-ci n'était pas annexée au contrat.

En ce qui est des CDS contrôle, l'article spécifie uniquement que le Centre de Santé fait partie des structures de soins de contrôle et qu'il recevra à ce titre une compensation d'un montant moyen similaire à celui du Centre de Santé auquel il est apparié de manière forfaitaire et sans contrepartie. En pratique, le montant perçu par les CDS contrôle correspondait à une moyenne pondérée (par taux de malnutrition et aire de responsabilité) de ce que recevait le groupe de traitement.

Il est à noter que les signatures de ces deux catégories de contrats spécifiques sont intervenues 1 mois après le début de l'intervention ; soit en février 2015 ; avec effet à partir du 01/01/2015.

Contrats des CDS 'secondaires'

En ce qui est des contrats secondaires pour les CDS non nutritionnels qui réfèrent les cas aux CDS intervention, leurs signatures sont intervenues 12 mois après le début de l'intervention avec effet à partir de 01/01/ 2016. Cette partie là de l'intervention a donc été de jure inopérationnelle en 2015. En pratique, un seul CDS intervention a signé de contrats secondaires avec deux autres CDS secondaires durant toute la période de l'intervention. Cependant, les modalités de vérification et de paiement des prestations déclarées par les les CDS secondaires n'étaient pas préalablement établies.

Contrats des GASC

En ce qui est des contrats avec les GASC, ceux-ci ont été signés lors des formations en septembre 2015, juste avant le démarrage officiel de l'intervention, en octobre 2015.

Vérification des prestations sous FBP Nutrition

Les vérifications quantitatives et qualitatives ont été réalisées dans le même cadre que le FBP existant.

Quantitative

Les vérifications des prestations quantitatives du FBP nutrition (pour les CDS intervention) ont commencé en Avril 2015 ciblant du coup les prestations des mois de Janvier, Février et Mars 2015. Puis elles ont été réalisées de manière mensuelle par la suite.

Malgré l'absence d'intrants diététiques pour le SSN au début de l'intervention, l'indicateur « *dépistage et prise en charge de la malnutrition aigue modérée chez les enfants de moins de 5 ans (SSN) »* a tout de même été vérifié dès avril 2015.

S'agissant de l'indicateur « *dépistage et référence des cas de malnutrition aigue (modérée et sévère) chez les enfants de moins de 5 ans »* : cet indicateur, qui est normalement vérifié une fois déclaré par les CDS secondaires (i.e. CDS sans service nutritionnel SSN et/ou STA mais référant vers les CDS du groupe d'intervention), a été vérifié et validé au niveau de certains CDS primaires dès 2015 ; alors même que les contrats secondaires n'ont été possibles qu'au début 2016.

Qualitative

Tout comme pour le FBP existant, les évaluations qualité relatives au FBP Nutrition sont réalisées par les ONGs d'accompagnement dans les provinces où il y en avait et par les BPS dans les provinces sans ONGs.

La formation sur la grille d'évaluation qualité FBP Nutrition a été réalisée auprès de ces agents en Juillet 2015. C'est donc fin juillet que la première évaluation qualitative des prestations FBP Nutrition a eu lieu ; elle concernait les prestations des deux premiers trimestres de 2015. Néanmoins, les résultats de l'évaluation des premier et deuxième trimestres 2015 n'ont pas été pris en compte pour les bonus/malus lors de l'établissement de la facture du mois d'Avril 2015. La raison exprimée était que leurs très bas scores allaient démotiver les CDS au premier contact des subsides nutrition. Les scores des évaluations qualitatives n'ont donc commencé à être utilisés dans le calcul des bonus/malus qu'avec l'évaluation de la qualité technique du troisième trimestre 2015.

Par la suite, les évaluations qualité ont été réalisées et utilisées dans les calculs des subsides tous les trimestres, à l'exception du cas suivant. En effet, il n'y a pas eu d'évaluation qualité au premier trimestre 2016 dans les provinces de Ngozi, Muyinga, Mwaro, Kayanza et Cibitoke ; pour ces CDS, les scores de qualité du 4è trimestre 2015 ont été reconduits pour le premier trimestre 2016 mais sans aucun malus de qualité appliqué pour les CDS qui avaient eu un score de qualité inferieur a 50%. Ceci a été dicté par le retard dans le processus de renouvellement des contrats des ONGs d'accompagnement qui avaient été recrutés avec pour mission principale d'évaluer trimestriellement la qualité technique des Centres de Santé de ces provinces. Pour les autres provinces restantes, l'évaluation du premier trimestre a eu lieu comme d'habitude avec les BPS et les BDS.

Il n'y a pas eu de vérification communautaire spécifique au FBP Nutrition, comme c'est le cas dans le FBP général.

Contre-vérification
Il n'y a pas eu de contre-vérification spécifique au FBP Nutrition.

Le cas des ASC
D'après les présidents des GASC, la vérification a été réalisée dès la fin du mois d'Octobre 2015. Elle se faisait mensuellement par le CPVV qui validait ce qui avait été fait et quel montant allait être payé. Il est à noter que le CDS faisait une prévérification (en confrontant le nombre d'enfants référés et ceux confirmés ainsi qu'en apposant leur signature sur les rapports des séances au niveau de la sous-colline).

Les GASC étaient payés pour chaque cas de malnutrition dépisté dans la communauté et référé au CDS avec une note préétablie. Les mesures anthropométriques étaient reprises au niveau du CDS pour la confirmation. Le bonus relatif au dépistage de la malnutrition n'était payé que pour les cas confirmés au niveau du CDS. Le CPVV chargé du suivi des activités devait à son tour valider le rapport des GASC après confrontation avec les cas confirmés par le CDS.

Concernant les activités de sensibilisation et de démonstration culinaire, leur tenue était conditionnée par la présence d'un infirmier envoyé par le CDS et les rapports y relatifs devaient comporter la signature de ce dernier. Les participants devaient également signer sur le rapport pour confirmer leur participation. L'effectif minimal exigé était de 15 participants.

Pour valider le payement pour ces deux activités, le CPVV devait vérifier que le rapport était fait suivant le canevas prévu à cet effet. Ce double contrôle était fait afin d'assurer la véracité des prestations faites par les GASC aussi bien du point de vue quantitatif que qualitatif. Certains problèmes ont néanmoins été rapportés, notamment le refus des participants de signer après participation aux différentes séances de sensibilisation.

Paiements

Paiements des CDS 'primaires' du groupe d'intervention
Comme pour le FBP existant, le montant correspondant aux prestations devait être viré sur les comptes bancaires de chaque CDS.

Les factures quantité de janvier, février et mars 2015 étaient prêtes à être payées en juillet 2015 pour les CDS intervention. Ainsi, le premier paiement des CDS intervention dans le cadre du FBP/ Nutrition est intervenu la première quinzaine du mois d'Août 2015. A ce moment, il était attendu les résultats de l'évaluation qualitative pour établir les factures du mois d'Avril. Celles-ci ont été payées en fin Novembre 2015 au même titre que les factures de Mai et Juin 2015. Au 31 Décembre 2015, les CDS avaient reçu les paiements des factures allant jusqu'à septembre 2015. Notons qu'au cours de l'année 2015 et dans le cadre du FBP classique, il y a eu des arriérés de paiement aux FOSA de 8,1 milliards suite à l'indisponibilité des ressources au niveau du gouvernement et à l'arrêt de certains projets financés par les PTFs.

En 2016, les paiements ont été réguliers jusqu'en juillet 2016 en rapport avec les factures des mois d'octobre 2015 à Avril 2016. Le reste des factures a été payé aucours de l'intervalle allant de Décembre 2016 à Février 2017.

En plus des premiers paiements trop tardifs, les titulaires se sont plaints par la suite qu'ils ne pouvaient pas facilement repérer les subsides FBP Nutrition sur leurs historiques bancaires. Ce problème de traçabilité était encore rapporté par certains titulaires à mi-2016.

Paiements des CDS 'primaires' du groupe de contrôle

En ce qui est des CDS contrôle, un premier paiement du montant compensatoire relatif aux mois de janvier à Septembre 2015 est intervenu fin Décembre 2015 ; soit un an après le démarrage de l'intervention. Par la suite, le paiement a été fait au rythme de celui du groupe intervention, c'est à dire des paiements réguliers jusqu'en juillet 2016 en rapport avec les montants compensatoires relatifs aux mois d'octobre 2015 à Avril 2016. Le reste des paiements est intervenu au cours de l'intervalle allant de Décembre 2016 à Février 2017.

Comme pour le groupe intervention, les CDS du groupe contrôle ont connu au cours de l'année 2015, des arriérés d'impayés dans le cadre du FBP classique.

Paiements des CDS primaires vers CDS secondaires

Les contrats secondaires pour les CDS non nutritionnels qui réfèrent les cas aux CDS intervention concernaient l'indicateur : « *dépistage et référence des cas de malnutrition aigue (modérée et sévère) chez les enfants de moins de 5 ans* ». Cet indicateur produit par les CDS secondaires (i.e. CDS sans service nutritionnel SSN et/ou STA mais référant vers les CDS du groupe d'intervention), le montant de la facture y relative était payé au CDS primaire. Seulement voilà, il n'y avait pas préalablement de consignes sur le remboursement à faire au CDS secondaire et la part qui devait rester au CDS primaire.

Paiements des GASC

Les paiements des GASC devaient être éxécutés mensuellement à partir d'octobre 2015. Cependant, il a été rapporté que cinq mois après le début du projet la plupart des GASC n'avaient pas encore reçu leur premier payement. Il a été rapporté que les paiements ont accusé de forts retards tout au long du projet. Aussi, lors de prises de contact avec les responsables des GASC en janvier 2017, la plupart ont rapporté ne pas avoir été payés depuis des mois (plus précisément, le dernier paiement datait d'avril 2016 pour une grande partie d'entre eux).

Supervision

D'après les données issues de l'enquête, il ne semble pas que la supervision relative à la malnutrition ait été davantage réalisée dans le groupe d'intervention.

Problématique des intrants du SSN

Depuis le montage de l'intervention FBP Nutrition, il était prévu qu'il démarre début janvier 2015 dans le groupe d'intervention. Cependant, au moment de mettre en œuvre le FBP Nutrition, depuis plusieurs mois déjà, le PAM s' était retiré d'un grand nombre de provinces et ne fournissait le CSB + sucre + huile que dans deux provinces (celles de Rutana et Ruyigi) ; il s'agit des intrants nécessaires au traitement de

la MAM. Aussi, pour pallier cela, l'idée qui avait été présentée aussi lors de la formation des CDS fin 2014 était d'augmenter les tarifs liés à l'indicateur de « détection et prise en charge de la MAM » afin que les CDS puissent se fournir localement en farine + sucre + huile. Cette idée d'achat isolé par chaque CDS n'a pas été soutenue par le PRONIANUT du fait que les titulaires des CDS ne sont pas formés dans la technologie agro-alimentaire et cette procédure ne permettait pas au PRONIANUT de faire le « contrôle qualité ». Pour le PRONIANUT, il leur serait difficile d'évaluer les indicateurs de performance des différents SSN alors qu'ils n'utilisent pas un produit de traitement identique.

Aussi, les CDS avaient été informés que PRONIANUT allait faire la prospection des différents producteurs locaux de farine (CSB) afin de s'approvisionner chez un seul fournisseur, qui devait alors être identifié et agréé, et ceci pour faciliter le contrôle de qualité de la farine. Au 30 Décembre 2014 (veille du démarrage de l'intervention), les démarches d'échanges avec les différents producteurs de farine n'avaient pas encore abouti et un message officiel du PRONIANUT à l'endroit des MCDs les a instruits à interdire aux titulaires des 45 CDS intervention ne pas acheter de farine dans les marchés locaux et attendre de nouvelles informations sur la disponibilité de la farine. Finalement, l'intervention a débuté sans régler cette question des intrants diététiques pour le traitement de la MAM.

Au fur et à mesure de la recherche des producteurs locaux de farine, il s'est avéré impossible de faire un marché de gré à gré (vu que le marché allait dépasser un milliard), et il doit y avoir une passation de marché suivant la réglementation burundaise. Le PRONIANUT s'est mis alors à préparer une proposition de DAO (dossier d'appel d'offres) soumis à l'expert en passation des marchés du PADSS. Il aura fallu 12 mois pour que le processus aboutisse (soit deux tiers de la durée prévue d'exposition à l'intervention). Une maison de production de farine (CEREALIS) a été retenue à la fin du processus. Concernant la livraison, il a été convenu que le fournisseur livre directement aux BDS. Concernant le contrôle qualité, il a été envisagé qu'il soit assuré par le BBN (Bureau Burundais de Normalisation). Ainsi, les CDS devaient estimer leurs besoins et les transmettre aux BDS qui en faisaient la compilation. La synthèse des besoins du district était envoyée au PRONIANUT pour avis et transmission au CEREALIS qui allait à son tour acheminer la quantité demandée par chaque CDS et validée par le PRONIANUT ainsi que la facture y relative. Pour le paiement, les CDS viraient le montant correspondant au compte du district ; ce dernier se chargeait de virer l'argent à son tour sur le compte de l'entreprise CEREALIS par rapport aux commandes du district (noter qu'il y avait un contrat signé préalablement entre CEREALIS et les MCDs concernés).

Ainsi, la livraison en ce qui est de la farine a commencé en janvier 2016 pour les CDS intervention à un rythme trimestriel. Pour l'approvisionnement en huile et en sucre, les CDS pouvaient se fournir localement, auprès du fournisseur de leur choix. Aussi, il est à noter que pour l'indicateur « *dépistage et prise en charge de la malnutrition aigue modérée chez les enfants de moins de 5 ans (SSN) »*, le tarif a été revu en conséquence à la hausse : le tarif unitaire était de 3 000 Fbu ; il est passé à 47000 FBU à partir de janvier 2016 pour prendre en compte le coût de la farine CSB mais aussi le coût de l'huile et du sucre (ces deux derniers achetés localement).

La majorité des CDS du groupe d'intervention ont fait des commandes en farine dès janvier 2016. Par contre, seuls 12 CDS du groupe contrôle avaient introduit leurs commandes en farine à partir du deuxième trimestre 2016.

Par après, le PAM a repris la fourniture d'intrants diététiques (Plumpy Sup, et non CSB huile et sucre comme initalement) aux CDS contrôle des provinces Rutana et Ruyigi.

Fin Mai 2016, il est fait état des farines avariées livrées aux CDS. La fourniture par la maison CEREALIS est alors momentanément suspendue et n'a repris qu'en septembre 2016 (date à confirmer).

Figure 4. Timeline du FBP Nutrition

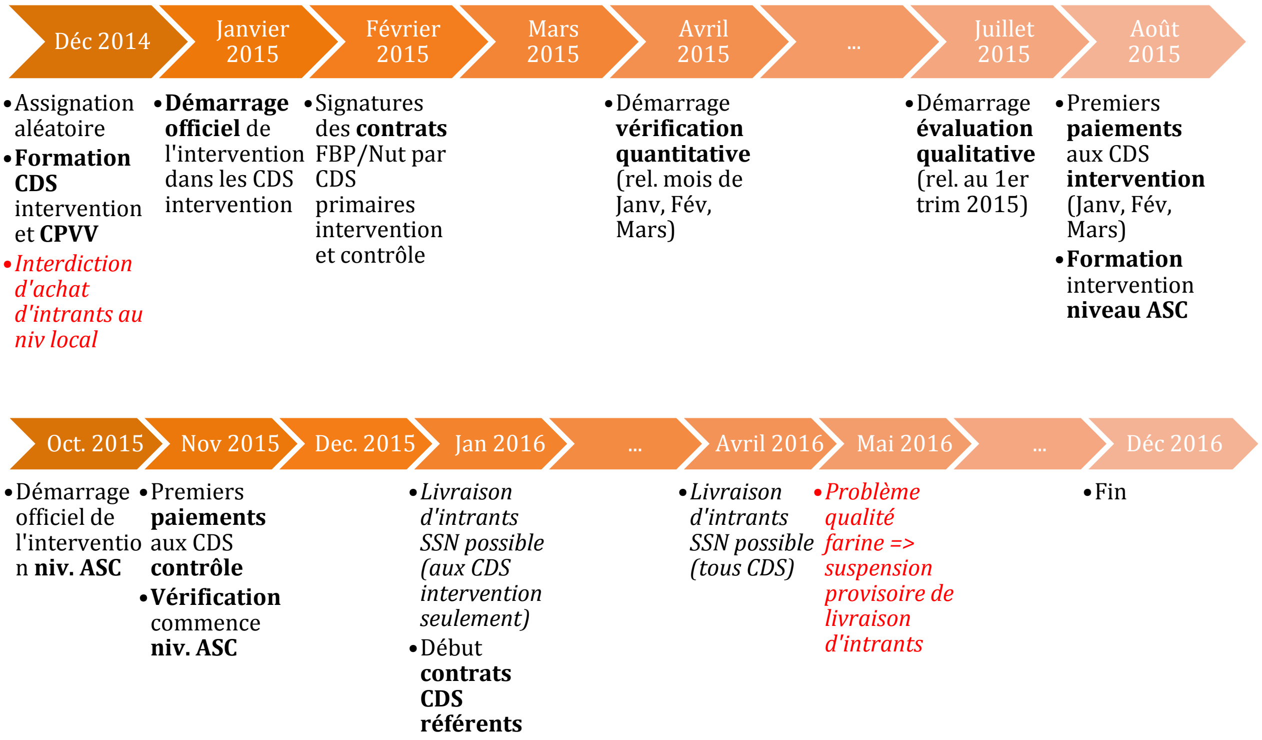

Source : auteurs.

Figure 5. Mise en œuvre du FBP Nutrition et de la compensation, par niveau et groupe de traitement

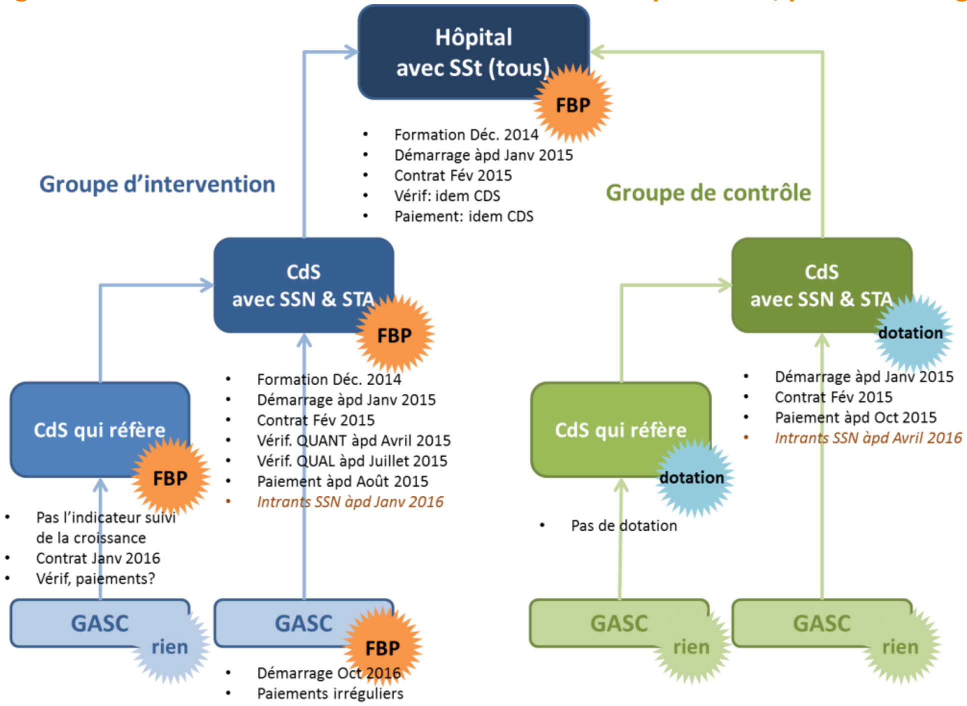

Source : auteurs

Interventions d'autres acteurs dans le système de santé

Des ONGs ont soutenu les FARNs dans leurs zones d'intervention. Il a également été signalé que dans la province de RUMONGE, avec l'appui des PTFs PATHFINDER et UNICEF, le PRONIANUT a réalisé en juillet 2016 une campagne de dépistage au niveau communautaire de la malnutrition chez tous les enfants de 6 à 59 mois. Les enfants dépistés avec malnutrition aigüe étaient envoyés aux SSN ou STA des CDS sous contrat FBP nutrition.

Evaluation qualitative de la mise en œuvre du FBP-Nutrition au niveau communautaire cinq mois après le démarrage de l'intervention

Afin de comprendre les facteurs entravant ou facilitant une mise en œuvre optimale de l'intervention FBP Nutrition au niveau communautaire, nous avons réalisé une enquête qualitative dans le groupe d'intervention auprès de présidents de GASC et des TPS relatifs.

Méthodologie

Dans un premier temps, début 2016, nous avons analysé les données de routine et sélectionné des GASC dans le groupe d'intervention selon (1) qu'ils performaient bien (càd GASC qui réalisaient toutes les activités sous PBF et dont les paiements augmentaient), (2) qu'ils performaient de façon sous–optimale (càd, qui réalisaient toutes les activités mais dont les paiements stagnaient) et (3) qu'ils performaient mal (càd toutes les activités n'étaient pas réalisées). Puis, en mars 2016, soit cinq mois après le démarrage de l'intervention, nous nous sommes rendus sur le terrain et avons effectué des entretiens semi-ouverts avec 42 présidents de GASC et 42 professionnels de santé responsables de l'encadrement des GASCs pour ce projet. Le choix a été porté sur ces derniers étant donné que seule la moitié des CDS ont des TPS dans leurs structures sanitiares.

Résultats

Les entretiens avec les différents informateurs-clés selon qu'ils étaient dans un groupe performant bien, sous-optimalement, ou peu, ont permis de mettre en évidence les facteurs suivants.

Facteurs ayant entravé la mise en œuvre :

- Manque de formation des ASC en matière d'art culinaire ou de dépistage de la malnutrition aigüe ;
- Manque de formation des ASC sur le processus de rapportage ;
- Manque d'outils de dépistage de la malnutrition dans certaines provinces ;
- Manque de matériel pédagogique pour les séances de sensibilisation ;
- Manque de communication avec la communauté entraînant une mauvaise compréhension de la pertinence des activités prévues dans ce projet ;
- Manque de communication avec les autorités locales entraînant un manque de collaboration de leur part (notamment du fait que la rémunération des ASC aurait suscité des jalousies) ;
- Retards considérables de payement des GASC entraînant démotivation et manque de moyens ;
- Personnel insuffisant au niveau du CDS (pas de techniciens de promotion de la santé) ne permettant pas une supervision-formative régulière des activités effectuées par les ASC ;
- La pauvreté et l'insécurité alimentaire des ménages sont également des facteurs entravant la bonne mise en œuvre des séances de démonstration culinaire (car ceux-ci sont supposés apporter les denrées).

Facteurs ayant facilité la mise en œuvre :

- Disponibilité et engagement du personnel de santé au niveau du CDS (les TPS) dans certaines provinces ;
- Soutien technique (formation des ASC) et matériel (denrées alimentaires pour les séances de démonstration culinaire, matériel pédagogique) apporté par certaines ONG dans certaines provinces.

4.3 Impact sur les indicateurs d'input et de processus

Equipement

Equipement disponible et fonctionnel

Pour réaliser les activités liées au suivi de la croissance des enfants, il faut au minimum le matériel suivant : Toise (min 2), Balance Salter (min 2), Pèse-bébé (min 2), Mètre ruban ou bracelet PB (min 2), Grille ou table poids/taille (min 3), IMC pour les enfants de plus de 5 ans (min 3), Culottes (min 4) ou bassines en plastique. Les enquêteurs ont vérifié si ces sept éléments étaient disponibles et fonctionnels dans le CDS. En moyenne, on en a actuellement 3.5 dans le groupe de contrôle et 3.6 dans le groupe d'intervention (Tableau 6). C'est moins que lors de l'enquête de référence (mais lors de l'enquête de référence, les vérifications par les enquêteurs n'étaient pas systématiques – de la même manière pour les deux groupes). L'équipement est jugé complet dans 2 (4%) des CDS du groupe de contrôle et dans 3 (7%) des CDS du groupe d'intervention (là aussi c'est moins qu'en 2014 : 4 CDS soit 11% dans les deux groupes). Cela suggère pas d'impact du FBP Nutrition sur l'équipement ; ceci est confirmé par la régression logistique (Tableau 6).

Tableau 6. Différence absolue entre le poids des enfants mesuré par les balances des CDS et le poids mesuré par les balances SECA (gold standard), en 2014 et 2017

		Groupe de controle			Groupe d'intervention			Impact FBP Nutrition
		Moy	N	N attendu	Moy	N	N attendu	
**Equipement (7 items)**	2014	4.53	36	45	4.26	38	45	n.s.
	2017	3.49	45	45	3.58	45	45	
	Diff	-1.04			-0.69			
**Equipement complet?**	2014	11%	36	45	11%	38	45	n.s.
	2017	4%	45	45	7%	45	45	
	Diff	-7pp			-4pp			

Source des données : questionnaire N ; n.s. signifie que l'impact est non significatif; pp signifie points de pourcentage.

Fiabilité des Pèse bébé

Les différences trouvées dans les prises de mesures du poids, effectuées par les enquêteurs à la sortie des consultations, entre la balance du CDS et la balance de l'enquête (SECA) demeurent existantes, et même augmentent légèrement dans les deux groupes. Cela suggère que les pèse-bébés des CDS ne sont pas plus fiables grace au FBP Nutrition. Ceci est confirmé par l'analyse économétrique de double différence en MCO (Tableau 7).

Tableau 7. Différence absolue entre le poids des enfants mesuré par les balances des CDS et le poids mesuré par les balances SECA (gold standard), en 2014 et 2017

		Groupe de controle			Groupe d'intervention			Impact FBP Nutrition
		Moy	N	N attendu	Moy	N	N attendu	
**Différence absolue poids entre balance CDS et balance SECA (mesures enquêteur)** (en kg)	2014	0.220	258	270	0.140	239	270	n.s.
	2017	0.310	252	270	0.330	260	270	
	Diff	+0.080			+0.190			

Source des données : questionnaire S; n.s. signifie que l'impact est non significatif.

Ces résultats de non impact du FBP Nutrition sur l'équipement sont contre-intuitifs dans la mesure où la grille qualitative du FBP Nutrition contient un indicateur relatif à la disponibilité et à la fonctionnalité des outils pour le dépistage et la prise en charge de la MAM et de la MAS. Les observations de routine suggèrent d'ailleurs une amélioration de ces indicateurs entre 2015 et 2016 (cf. Tableau 60, annexe). Ceci met en doute la qualité des évaluations effectuées pour la grille qualitative et l'efficacité de ces grilles quant à l'influence qu'elles ont sur l'activité des centres de santé.

Disponibilité des traitements diététiques

En 2017, le traitement diététique pour la MAM (CSB+huile+sucre) est disponible dans 5 (11%) des CDS dans les deux groupes. On ne peut malheureusement pas bien le comparer avec 2014 car il manquait alors pas mal de données (liées à un problème de filtres dans le questionnaire). De même, on a observé une rupture dans ce traitement sur la période des trois mois précédant l'enquête de 2017 dans 83 (92%) CDS, également répartis entre les deux groupes. Mais là encore c'est difficile de comparer avec 2014 (Tableau 8).

En ce qui concerne le traitement diététique de la MAS (PlumpyNut®), il est disponible dans 77 (89%) des CDS, également répartis entre les groupes de traitement et de contrôle. L'amélioration en disponibilité est semble-t-il meilleure dans le groupe de contrôle (+20%pts) que dans le groupe d'intervention (+7%pts). On a connu également moins de ruptures de stock dans les trois mois qui ont précédé l'enquête : 49% dans le groupe de contrôle et 38% dans le groupe d'intervention (Tableau 8).

Les régressions logistiques de double différence n'ont en revanche trouvé aucun impact du FBP nutrition sur ces quatre indicateurs (Tableau 8). Ce résultat de non impact du FBP Nutrition sur la disponibilité des intrants semble contre-intuitif dans la mesure où la grille qualitative du FBP Nutrition contient un indicateur relatif à la disponibilité des intrants pour la prise en charge de la MAM et de la MAS. Cela étant, en ce qui concerne la MAM, l'approvisionnement en intrants a été soumis à de forts aléas (cf. section précédente pour une description détaillée), et les scores relatifs à cet indicateur restent faibles tout au long de l'intervention même si on observe quelque amélioration (de 1.2/10 en moyenne dans le groupe d'intervention en 2015, à 4.6/10 en moyenne en 2016, cf. Tableau 60, annexe). En ce qui

concerne la MAS, l'indicateur suggère en effet une certaine stabilité à un niveau moyen : de 6.5/10 en moyenne en 2015 à 6.9/10 en moyenne en 2016, dans le groupe d'intervention (cf. Tableau 60, annexe). Ceci suggère que la grille qualitative n'a pas d'impact sur l'approvisionnement en intrants : soit que cela n'a pas d'influence sur le comportement des agents du centre de santé, soit qu'ils n'ont pas le contrôle de cette activité.

Des analyses économétriques supplémentaires suggèrent par ailleurs qu'il n'y a pas eu de quelconque contamination de l'intervention au sein des districts où il y avait deux types de centres de santé (contrôle et intervention) en terme de disponibilité des intrants diététiques (notamment pour le SSN ; tableaux disponibles sur demande).

Tableau 8. Disponibilité et rupture des traitements diététiques, en 2014 et 2017

		Groupe de controle			Groupe d'intervention			Impact FBP Nutrition
		Moy	N	N attendu	Moy	N	N attendu	
**CSB+huile+sucre disponible le jour de l'enquête?**	2014	26%	27*	45	11%	27*	45	n.s.*
	2017	11%	45	45	11%	45	45	
	Diff	-15pp			=			
**Rupture en CSB/huile/sucre les trois mois précédant l'enquête?**	2014	81%	27*	45	96%	27*	45	n.s.*
	2017	93%	45	45	91%	45	45	
	Diff	+12pp			-5pp			
**PlumpyNut® disponible le jour de l'enquête?**	2014	69%	45	45	82%	44	45	n.s.
	2017	89%	44	45	88%	43	45	
	Diff	+20pp			+7pp			
**Rupture en PlumpyNut® les trois mois précédant l'enquête?**	2014	55%	42	45	55%	44	45	n.s.
	2017	49%	45	45	38%	45	45	
	Diff	-6pp			-17pp			

Source des données : questionnaire N; n.s. signifie que l'impact est non significatif; pp signifie points de pourcentage. *un problème de filtre dans le questionnaire a fait qu'on n'a des données sur le traitement diététique du SSN que pour 27 CDS au lieu de 45 en 2014 : les comparaisons entre 2014 et 2017 et mesures d'impact sont donc à prendre avec précaution.

Ressources humaines

Pratiques des AS en consultation

En consultation

Les observations de consultations permettent de voir si les consultations effectuées par les agents de santé comprennent les anamnèses et examens nécessaires à la prévention et au dépistage de la

malnutrition. Les agents de santé devraient poser 5 à 7 questions relatives à la nutrition (selon l'âge de l'enfant) et réaliser 6 examens physiques relatifs à la nutrition, cf. tableau suivant :

Tableau 9. Liste des questions et examens relatifs à la nutrition

**Questions à poser**	L'enfant a–t-il de l'appétit?
	L'allaitez-vous encore? (pour les moins de 2 ans)
	Combien de fois l'enfant est-il allaité dans la journée ? (pour les moins de 2 ans)
	L'enfant consomme-t-il d'autres aliments liquides ? Lesquels ?
	A quel âge avez-vous commencé les autres aliments ?
	Décrivez-moi ce qu'il mange normalement pendant une journée (24 heures) / qu'a t-il mangé depuis hier ?
	Est-ce que l'alimentation a été changée récemment ?
**Examens à réaliser**	Prise de poids (ou évocation du poids pendant la consultation si le poids a été pris précédemment)
	Prise de la taille (idem)
	Prise du périmètre brachial (idem)
	Recherche d'oedèmes aux pieds
	Dessin de la courbe de croissance
	Calcul du z-score

Dans les deux groupes, le nombre moyen de questions posées reste en deça de une question (sur sept pour les enfants de moins de deux ans et sur cinq pour les enfants de plus de deux ans) et le nombre d'examens effectués reste en deça de deux (sur six). On dénote une très légère amélioration dans le groupe de contrôle et une légère détérioration dans le groupe d'intervention, mais rien n'est attribuable au FBP Nutrition selon les estimations de double différence (coefficient non significatif ; Tableau 10).

Afin de promouvoir une bonne nutrition, il est aussi attendu des agents de santé qu'ils délivrent des conseils en consultation, en termes de nutrition et d'hygiène. En 2017, des conseils nutritionnels ne sont faits que dans 24% des cas dans le groupe de contrôle et 19% des cas dans le groupe d'intervention : pour le groupe d'intervention c'est 11 points de pourcentage de moins qu'en 2014 ; d'après les régressions logistiques, l'impact du FBP Nutrition est de -12 points de pourcentage et est significativement différent de zéro. En ce qui concerne les conseils d'hygiène, ils ne sont donnés que dans 8% et 10% des cas respectivement dans le groupe de contrôle et le groupe d'intervention : c'est là aussi moins bien qu'en 2014, et ce dans les deux groupes (pas d'impact significatif ; Tableau 11)[8].

[8] Noter que la qualité des conseils n'a pas été analysée ici.

Tableau 10. Réalisation des questions et examens relatifs à la nutrition en consultation

		Groupe de controle				Groupe d'intervention				Impact FBP Nutrition
		Moy	Med	N	N att	Moy	Med	N	N att	
**Nb de questions nutrition posées chez les enfants de moins de deux ans** (max=7)	2014	0.72	1	137	-	0.74	1	125	-	n.s.
	2017	0.95	1	128	-	0.74	0	106	-	
	Diff	+0.23				-0.01				
**Nb de questions nutrition posées chez les enfants de plus de deux ans** (max = 5)	2014	0.59	0	148	-	0.68	0	164	-	n.s.
	2017	0.66	1	134	-	0.58	0	159	-	
	Diff	+0.06				-0.10				
**Nb d'examens nutrition effectués** (max = 6)	2014	1.44	1	259	270	1.59	1	254	270	n.s.
	2017	1.60	1	261	270	1.41	1	263	270	
	Diff	+0.16				-0.18				

Source de données : observations des consultations (questionnaire C) ; n.s. signifie que l'impact est non significatif.

Tableau 11. Les agents de santé ont-ils donné des conseils nutritionnels et d'hygiène aux patients ?

		Groupe de controle			Groupe d'intervention			Impact FBP Nutrition
		Moy	N	N att	Moy	N	N att	
**Conseils nutritionnels**	2014	20.8%	260	270	29.9%	254	270	-12.3pp P=0.060
	2017	23.6%	263	270	18.9%	265	270	
	Diff	+3pp			-11pp			
**Conseils d'hygiène**	2014	11.2%	259	270	19.3%	254	270	n.s.
	2017	8.4%	261	270	10.2%	265	270	
	Diff	-3pp			-9pp			

Source de données : observations des consultations (questionnaire C) ; n.s. signifie que l'impact est non significatif; pp signifie points de pourcentage.

Qualité des mesures anthropométriques

Il semble que les prises de mesures anthropométriques n'aient pas été améliorées avec l'intervention (cf. Tableau 12 ci-dessous). Les différences absolues trouvées dans les mesures de poids, taille et PB entre les AS et les enquêteurs demeurent présentes dans les deux groupes, et ont même tendance à augmenter. Cela suggère pas d'impact du FBP Nutrition sur cet indicateur, ce qui est confirmé par des régressions économétriques linéaires en MCO.

Tableau 12. Qualité des mesures anthropométriques dans les CDS en 2014 et 2017

		Groupe de controle			Groupe d'intervention			Impact FBP Nutrition
**Différence absolue de --- mesuré(e) par l'AS avec --- mesuré(e) par l'enquêteur (référence)**		Moyenne	N	N attendu	Moyenne	N	N attendu	
**Le poids** (en kg)	2014	0.40	199	270	0.18	205	270	n.s.
	2017	0.51	187	270	0.29	188	270	
	Diff	+0.11			+0.11			
**La taille** (en cm)	2014	1.80	128	270	0.60	141	270	n.s.
	2017	1.59	113	270	1.62	118	270	
	Diff	-0.20			+1.02			
**Le Périmètre brachial** (en mm)	2014	6.04	98	270	5.24	126	270	n.s.
	2017	7.46	137	270	7.51	120	270	
	Diff	+1.42			+2.27			

Source des données : questionnaire S; n.s. signifie que l'impact est non significatif.

Connaissances des AS

Les vignettes sont des cas fictifs de consultation. Elles ont été administrées aux agents de santé en fin de journée de sorte qu'il n'y avait pas la pression des patients qui attendent et qu'ils avaient tout leur temps. Par ailleurs, les agents de santé peuvent 'réaliser' tous les examens qu'ils souhaitent, sans contrainte de défaillance de l'équipement, et délivrer n'importe quel traitement, sans contrainte de non disponibilité en pharmacie. Les conditions hypothétiques sont donc idéales et permettent d'avoir une idée ajustée du savoir-faire des agents de santé.

Malgré cela, dans les deux groupes, le nombre moyen de questions posées reste en deça de deux questions (sur sept ; c'est mieux qu'en consultation mais cela reste peu) et le nombre d'examens effectués reste en deça de trois (sur six ; idem). La courbe de croissance n'est presque jamais mentionnée. Il ne semble pas que le FBP Nutrition ait eu un quelconque impact sur ces éléments (cf. Tableau 13).

Afin de promouvoir une bonne nutrition, il est aussi attendu des agents de santé qu'ils délivrent des conseils en consultation, en termes de nutrition et d'hygiène. En 2017, des conseils nutritionnels ne sont faits que dans 47% des cas dans le groupe de contrôle et 32% des cas dans le groupe d'intervention : d'après les régressions logistiques, l'impact du FBP Nutrition est de -25 points de pourcentage et est significativement différent de zéro. En ce qui concerne les conseils d'hygiène, ils ne sont donnés que dans 18% et 14% des cas respectivement dans le groupe de contrôle et le groupe d'intervention ; aucun impact n'est identifié[9] (cf. Tableau 14).

[9] Noter que la qualité des conseils n'a pas été analysée ici.

Les trois vignettes étaient les mêmes en 2014 et en 2017 et correspondaient toutes les trois à des cas de malnutrition aigue : la première était un cas de malnutrition aigue sévère compliquée, la deuxième un cas de malnutrition aigue non compliquée, et la troisième un cas de malnutrition aigue modérée. Pour la première, le diagnostic de malnutrition aigue sévère compliquée a été retrouvé par 14% des agents de santé en 2017 ; c'est peu mais c'est mieux qu'en 2014, particulièrement dans le groupe d'intervention. Pour la deuxième, le cas de malnutrition aigue sévère non compliquée a été retrouvé par 23% des agents de santé ; c'est là aussi mieux qu'en 2014, particulièrement dans le groupe d'intervention. Enfin, pour la troisième, le cas de malnutrition aigue modérée a été retrouvé par 38% des agents de santé. Il ne semble pas que le FBP Nutrition ait eu un impact sur ces taux de réussite.

Notons que, sans surprise, il y a une corrélation partielle significative de la performance des agents de santé entre les consultations observées et les vignettes (coefficient de corrélation=0.25 ; $p<0.05$; N=317). Certains agents ont la même performance selon qu'ils soient observés en consultations ou en vignettes (points situés sur la droite, cf. Figure 6 ci-dessous). La plupart des agents cependant obtient de meilleurs scores en vignettes par rapport aux consultations observées, ce qui suggère que des améliorations dans la performance des agents en consultation sont d'ores et déjà possibles, si par exemple les agents disposaient de davantage de temps par patient, de tous les équipements et médicaments nécessaires, etc.

Tableau 13. Réalisation des questions et examens relatifs à la nutrition en consultation dans les vignettes, en 2014 et 2017

		Groupe de controle			Groupe d'intervention			Impact FBP Nutrition
		Moy	N	N att	Moy	N	N att	
**Nb de questions nutrition posées chez les enfants de moins de deux ans** (max=7)	2014	1.45	70	90	1.37	74	90	n.s.
	2017	1.36	85	90	1.19	81	90	
	Diff	-0.09			-0.18			
**Nb d'examens nutrition effectués** (max = 6)	2014	2.09	71	90	2.38	74	90	n.s.
	2017	2.86	85	90	2.61	81	90	
	Diff	+0.76			+0.23			
**Courbe de croissance mentionnée**	2014	6.1%	71	90	6.8%	74	90	n.s.
	2017	3.7%	85	90	3.3%	81	90	
	Diff	-2.4pp			-3.5pp			

Source de données : vignettes (V); n.s. signifie que l'impact est non significatif; pp signifie points de pourcentage. N correspond ici au nombre d'agents de santé ayant répondu à l'enquête.

Tableau 14. Conseils nutritionnels et d'hygiène donnés par les AS aux patients fictifs des vignettes

		Groupe de controle			Groupe d'intervention			Impact FBP Nutrition
		Moy	N	N attendu	Moy	N	N attendu	
**Conseils nutritionnels**	2014	27.7%	71	90	34.2%	74	90	-24.7pp p=0.012
	2017	46.7%	85	90	31.5%	81	90	
	Diff	+19.0pp			-2.8pp			
**Conseils d'hygiène**	2014	9.9%	71	90	14.9%	74	90	n.s.
	2017	18.2%	85	90	13.6%	81	90	
	Diff	+8.4pp			-1.3pp			

Source de données : vignettes (V) ; n.s. signifie que l'impact est non significatif; pp signifie points de pourcentage. N correspond ici au nombre d'agents de santé ayant répondu à l'enquête.

Tableau 15. Taux de succès sur les diagnostics de malnutrition sur les vignettes en 2014 et 2017

		Groupe de controle			Groupe d'intervention			Impact FBP Nutrition
		Moy	N	N attendu	Moy	N	N attendu	
**Vignette 1 MAS compliquée**	2014	7.0%	71	90	6.8%	74	90	n.s.
	2017	12.9%	85	90	16.0%	81	90	
	Diff	+5.9pp			+9.3pp			
**Vignette 2 MAS non compliquée**	2014	15.5%	71	90	12.2%	74	90	n.s.
	2017	20.0%	85	90	25.9%	81	90	
	Diff	+4.5pp			+13.8pp			
**Vignette 3 MAM**	2014	28.2%	71	90	24.3%	74	90	n.s.
	2017	42.4%	85	90	33.3%	81	90	
	Diff	+14.2pp			+9.0pp			

Source de données : vignettes (V) ; n.s. signifie que l'impact est non significatif; pp signifie points de pourcentage. N correspond ici au nombre d'agents de santé ayant répondu à l'enquête.

Figure 6. Performance des 317 agents de santé enquêtés en 2014 et 2017

Scores de nutrition obtenus lors des consultations observées versus lors des vignettes

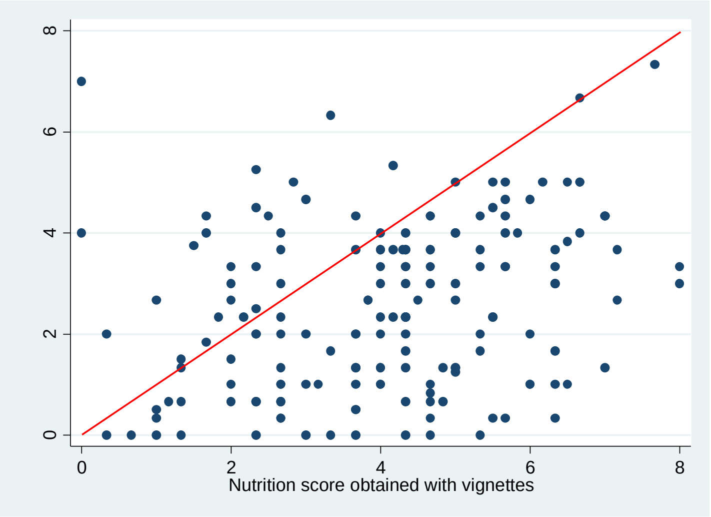

Source de données : vignettes (V) et consultations observées (C) en 2017 ; le score de nutrition est la moyenne par consultation/cas du nombre de questions et examens nutrition effectués ; chaque point représente les scores d'un agent ; la droite représente une situation où la performance par agent serait la même entre consultations observées et vignettes.

Volume et structure des ressources humaines

Le nombre d'employés et la structure du staff en terme de genre sont restés relativement similaires en 2017 par rapport à 2014, et ce dans les deux groupes : il ne semble pas qu'il y ait eu des recrutements de staff grâce aux financements supplémentaires apportés dans les deux groupes. Aussi, selon les normes sanitaires du Burundi, un centre de santé devrait avoir au moins trois infirmiers avec un diplôme A2 (ou plus), trois infirmiers avec un diplôme A3, un technicien de laboratoire, un technicien de promotion de la santé ainsi qu'un gestionnaire financier. On avait vu en 2014 que seulement 22% des centres de santé respectaient ces normes. En 2017, ce chiffre a baissé, plus particulièrement dans le groupe d'intervention où seuls 9% des CDS respectent ces normes (cf. Tableau 16).

Tableau 16. Ressources humaines dans les CDS en 2014 et 2017

		Groupe de controle			Groupe d'intervention			Impact FBP Nutrition
		Moy	N	N attendu	Moy	N	N attendu	
**Nombre d'employés**	2014	16.78	45	45	14.18	45	45	n.s.
	2017	17.36	45	45	14.33	45	45	
	Différence	=			=			
**% hommes**	2014	59.83%	45	45	59.56%	45	45	n.s.
	2017	57.12%	45	45	59.08%	45	45	
	Différence	-2.71pp			-0.48pp			
**Poste TPS existant**	2014	55.56%	45	45	46.67%	45	45	n.s.
	2017	60.00%	45	45	46.67%	45	45	
	Différence	+4.44pp			=			
**Poste gestionnaire existant**	2014	82.22%	45	45	77.78%	45	45	n.s.
	2017	84.44%	45	45	88.89%	45	45	
	Différence	+2.22pp			+11.11pp			
**Normes de RH respectées**	2014	24.44%	45	45	20.00%	45	45	n.s.
	2017	22.22%	45	45	8.89%	45	45	
	Différence	-2.22pp			-11.11pp			

Source : questionnaire G; n.s. signifie que l'impact est non significatif; pp signifie points de pourcentage.

Formation de personnel

En 2017, environ 19% des AS reportent avoir reçu une formation relative au FBP, 53% relative à la nutrition, et 31% relative à la DPSHA ; environ 36% disent ne pas avoir reçu de formation après leur formation initiale. Il ne semble pas que le FBP Nutrition ait eu un impact sur cela (cf. Tableau 17).

Tableau 17. Formations des ressources humaines dans les CDS en 2014 et 2017

		Groupe de controle			Groupe d'intervention			Impact FBP Nutrition
		Moy	N	N attendu	Moy	N	N attendu	
**Formation FBP**	2014	21.1%	71	90	23.0%	74	90	n.s.
	2017	18.8%	85	90	18.5%	81	90	
	Diff	-2.3pp			-4.5pp			
**Formation nutrition**	2014	46.5%	71	90	43.2%	74	90	n.s.
	2017	54.1%	85	90	51.9%	81	90	
	Diff	+7.6pp			+8.6pp			
**Formation DPSHA**	2014	18.3%	71	90	24.3%	74	90	n.s.
	2017	24.7%	85	90	34.6%	81	90	
	Diff	+6.4pp			+10.2pp			
**Formation : aucune**	2014	43.7%	71	90	39.2%	74	90	n.s.
	2017	38.8%	85	90	32.1%	81	90	
	Diff	-4.8pp			-7.1pp			
**Salaire toujours reçu les 12 derniers mois**	2014	90.9%	66	90	93.9%	66	90	n.s.
	2017	98.8%	83	90	94.9%	79	90	
	Diff	+7.9pp			+1.0pp			

Source : questionnaire V; n.s. signifie que l'impact est non significatif; pp signifie points de pourcentage. N correspond ici au nombre d'agents de santé ayant répondu à l'enquête.

Interactions du CDS avec le niveau communautaire

En 2017, environ 78% des CDS encadrent les ASC pour les activités de dépistage, dans les deux groupes (cf. Tableau 18). Les CDS encadrent moins les activités de démonstration culinaire: 18% le font dans le groupe de contrôle, 53% dans le groupe d'intervention. Enfin les CDS encadrent des activités de sensibilisation à hauteur de 76% dans le groupe contrôle et 71% dans le groupe intervention. Le FBP Nutrition ne semble pas avoir influé sur ces activités, excepté pour les démonstrations culinaires où l'impact est de +50.4 points de pourcentage (p=0.000 ; cf. Tableau 18).

Tableau 18. Activités réalisées par les agents de santé communautaire relatives à la nutrition faites sous l'encadrement du CDS

		Groupe de controle			Groupe d'intervention			Impact FBP Nutrition
		Moy	N	N att	Moy	N	N att	
**Dépistage**	2014	88.9%	36	45	87.1%	31	45	n.s.
	2017	77.8%	45	45	77.8%	45	45	
	Diff	-11.1pp			-9.3pp			
**Démonstrations culinaires ou FARN**	2014	52.8%	36	45	29.0%	31	45	+50.4pp p=0.000
	2017	17.8%	45	45	53.3%	45	45	
	Diff	-35.0pp			+24.3pp			
**Sensibilisation**	2014	83.3%	36	45	58.1%	31	45	n.s.
	2017	75.6%	45	45	71.1%	45	45	
	Diff	-7.8pp			+13.0pp			

Source : questionnaire N; n.s. signifie que l'impact est non significatif; pp signifie points de pourcentage.

Supervision

En 2017, environ 65% des AS reportent avoir été supervisé durant le six mois précédant l'enquête. C'est davantage qu'en 2014. En même temps, selon les informations obtenues auprès des responsables nutrition des CDS, les CDS ont reçu un peu moins de supervision de la part de l'équipe cadre district sur le thème de la malnutrition, et ce, dans les deux groupes. Il ne semble pas que l'intervention FBP Nutrition ait eu une quelconque influence sur la supervision (cf. Tableau 19).

Tableau 19. Supervision en 2014 et 2017

		Groupe de controle			Groupe d'intervention			Impact FBP Nutrition
		Moy	N	N att	Moy	N	N att	
**AS supervisé durant les 6 derniers mois** (source V)	2014	32.4%	71	90	45.9%	74	90	n.s.
	2017	65.9%	85	90	65.4%	81	90	
	Diff	+33.5pp			+19.5pp			
**Supervision durant le dernier trimestre de la part de l'ECD sur la malnutrition** (source N)	2014	43.2%	44	45	47.7%	44	45	n.s.
	2017	35.6%	45	45	42.2%	45	45	
	Diff	-7.6pp			-5.5pp			

Note : n.s. signifie que l'impact est non significatif; pp signifie points de pourcentage.

Information sanitaire

Disponibilité de la documentation SSN et STA

Les services de prise en charge de la malnutrition nécessitent un registre pour chaque type de service (SSN et STA) et des fiches individuelles pour chacun des patients. D'après les données issues du questionnaire auprès du responsable du service nutrition (questionnaire N), il semble que ces documents soient davantage disponibles et à jour dans le groupe d'intervention par rapport à la baseline, et par rapport au groupe de contrôle. En effet les régressions logistiques suggèrent un impact positif et significatif sur la bonne tenue de ce type de documentation : l'impact est d'environ +69 points de pourcentage pour la documentation dans le service SSN et de 22 à 23 points de pourcentage pour dans le service STA (cf. Tableau 20).

Les indicateurs de performance du SSN sont systématiquement calculés dans 44% des CDS du groupe d'intervention contre 11% dans le groupe de contrôle en 2017 ; l'impact du FBP Nutrition est de +41 points de pourcentage et est significatif (cf. Tableau 20). Cependant, rappelons qu'un grand nombre de CDS du groupe contrôle ont déclaré ne pas disposer d'un service de prise en charge de la MAM effectif en 2017 ; de même pour environ la moitié des CDS en 2014 (dans les deux groupes) : il est probable que ces CDS n'aient pas calculé les indicateurs de performance de ce service. Aussi, si on ne considère que les CDS qui ont un SSN actif (c'est-à-dire en excluant ces CDS ayant répondu ne pas disposer de service de prise en charge de la MAM effectif), l'impact n'est plus significatif : cela suggère donc que les résultats d'impact sur le calcul des indicateurs de performance sont influencés par l'impact sur le fait de garder ouvert et effectif le service (cf. note de bas de tableau, Tableau 20).

Les indicateurs de performance du STA sont systématiquement calculés dans 76% des CDS dans le groupe d'intervention contre 62% dans le groupe de contrôle en 2017 ; l'impact du FBP Nutrition est de + 25 points de pourcentage sur cet indicateur (cf. Tableau 20).

Il semble ainsi que les CDS du groupe d'intervention aient fait particulièrement attention à la disponibilité de la documentation SSN et STA. Ceci est probablement dû au fait que ces documents sont utilisés dans le système de vérification du FBP Nutrition, et donc nécessaires à la validation et au paiement des subsides relatifs aux indicateurs de prise en charge de MAM et de MAS.

Tableau 20. Disponibilité et actualisation de la documentation SSN et STA en 2014 et 2017

		Groupe de controle			Groupe d'intervention			Impact FBP Nutrition
		Moy	N	N attendu	Moy	N	N attendu	
**SSN registre disponible et à jour**	2014	33.3%	45	45	11.1%	45	45	+68.8pp P=0.000
	2017	17.8%	45	45	71.1%	45	45	
	Diff	-15.6pp			+60.0pp			
**SSN fiches disponibles et à jour**	2014	31.1%	45	45	8.9%	45	45	+68.9pp P=0.000
	2017	8.9%	45	45	68.9%	45	45	
	Diff	-22.2pp			+60.0pp			
**STA registre disponible et à jour**	2014	73.3%	45	45	59.1%	44	45	+21.6pp P=0.019
	2017	71.1%	45	45	82.2%	45	45	
	Diff	-2.2pp			+23.1pp			
**STA fiches disponibles et à jour**	2014	77.8%	45	45	59.1%	44	45	+23.5pp P=0.014
	2017	62.2%	45	45	71.1%	45	45	
	Diff	-15.6pp			+12.0pp			
**indicateurs performance SSN calculés***	2014	44.4%	27	45	37.0%	27	45	+40.9pp P=0.000
	2017	11.1%	45	45	44.4%	45	45	
	Diff	-33.3pp			+7.4pp			
**indicateurs performance STA calculés**	2014	44.4%	45	45	53.3%	44	45	+24.7pp P=0.039
	2017	62.2%	45	45	75.6%	45	45	
	Diff	+17.8pp			+22.2pp			

Source : questionnaire N; n.s. signifie que l'impact est non significatif; pp signifie points de pourcentage. Note : *si on ne considère que les CDS où le SSN est actif, on observe une réduction de 42.3% (n=26) à 33.3% (n=12) dans le groupe de contrôle, et de 38.5% (n=26) à 43.2% (n=44) dans le groupe d'intervention, et l'impact du FBP Nutrition devient non significatif sur cet indicateur.

Remplissage des fiches cliniques SSN

Pour chaque enfant admis dans un programme SSN ou STA, un certain nombre d'indicateurs doivent être reportés au minimum. Il s'agit : des mesures anthropométriques à l'entrée et à la sortie (poids, taille, PB, oedèmes) et de la raison de sortie (guéri, non répondant, décès, transfert, abandon). Selon les fiches retranscrites, le taux de non renseignement du poids à l'entrée est très faible, dans les deux groupes, dans les deux services SSN et STA, en 2014 et 2017. En revanche, les autres éléments sont parfois, voire souvent, non renseignés (cf. Tableau 21).

La taille à l'entrée est non renseignée en 2017 dans 6.4% des fiches du groupes de contrôle contre 0.8% des fiches du groupe d'intervention : on trouve un impact positif du FBP Nutrition dans le bon remplissage de ces fiches concernant cet indicateur (-7.7 points de pourcentage de fiches ne renseignant pas la taille à l'entrée, p=0.023, cf. Tableau 21).

Le PB à l'entrée est non rensigné dans 2.1% des cas dans le groupe d'intervention contre 7.7% dans le groupe de contrôle en 2017 ; on voit donc une amélioration dans les deux groupes car en 2014 le taux de non renseignés était de 15.8% et 19.4% respectivement. En revanche, les oedèmes à l'entrée sont

davantage non renseignés : à hauteur de 66% dans le groupe de contrôle et de 69% dans le groupe d'intervention. La raison de sortie est toujours non renseignée à hauteur d'environ un tiers ; on perçoit en revanche une amélioration dans le groupe de contrôle (cf. Tableau 21).

Le poids à la sortie est relativement bien renseigné : seules 4.5 % des fiches ne le renseignent pas en 2017 ; la taille et le PB à la sortie sont également mieux renseignés qu'en 2014, et ce dans les deux groupes ; les oedèmes à la sortie sont toujours aussi mal renseignés (cf. Tableau 21).On ne trouve pas d'influence du FBP Nutrition sur d'autres renseignements que la taille à l'entrée. Aussi, on remarque que les oedèmes sont les grands oubliés, à l'entrée et à la sortie.

Pour synthétiser, on observe que tous les paramètres d'entrée et de sortie du SSN sont renseignés dans seulement 13.5% des fiches dans le groupe de contrôle et 15.3% des fiches dans le groupe d'intervention, en 2017 ; on observe une légère amélioration dans les deux groupes, et pas d'influence du FBP Nutrition (cf. Tableau 23). Ce résultat est contre-intuitif dans la mesure où la grille qualitative du FBP Nutrition contient des vérifications du respect des critères d'admission, du bon remplissage du registre, et de la bonne prise en charge de 20 cas : ces indicateurs ont d'ailleurs vu une amélioration entre 2015 et 2016, dans le groupe d'intervention (cf. Tableau 60, annexe). L'impact de la grille qualitative et la qualité des évaluations qualitatives sont mis en doute par ces résultats.

Remplissage des fiches cliniques STA

La taille à l'entrée est non renseignée en 2017 dans 19% des fiches du groupes de contrôle contre 6% des fiches du groupe d'intervention : on trouve un impact positif du FBP Nutrition dans le bon remplissage de ces fiches concernant cet indicateur (-18.7 points de pourcentage de fiches ne renseignant pas la taille à l'entrée, p=0.005, cf. Tableau 22).

Le périmètre brachial (PB) à l'entrée est non rensignée dans 10% des cas dans le groupe d'intervention contre 19% dans le groupe de contrôle en 2017 ; on voit donc une amélioration dans les deux groupes car en 2014 le taux de non renseignés était de 14% et 13% respectivement. Il semble que le FBP Nutrition ait eu un impact sur le bon remplissage de cet indicateur (-8.8 points de pourcentage de fiches ne renseignant pas le PB à l'entrée, p=0.090). Les oedèmes à l'entrée sont davantage renseignés qu'en SSN mais restent pour une large part non renseignés : à hauteur de 29% dans le groupe de contrôle et de 15% dans le groupe d'intervention. La raison de sortie est toujours non renseignée à hauteur d'environ un tiers (cf. Tableau 22).

Le poids à la sortie est relativement bien renseigné : seules 2 % des fiches ne le renseignent pas en 2017 ; le PB à la sortie est égalemet mieux renseigné qu'en 2014 : seules 13% des fiches ne renseignent pas le PB à la sortie en 2017. En revanche, la taille est beaucoup moins bien renseignée en 2017 qu'en 2014, et ce dans les deux groupes : environ 89% des fiches ne donnent pas d'information sur la taille de l'enfant à la sortie en 2017 contre 55% en 2014. les oedèmes à la sortie sont également très mal renseignés : 95% des fiches ne donnent pas d'information là-dessus en 2017 contre 48% en 2014 (cf. Tableau 22).On ne trouve pas d'influence du FBP Nutrition sur d'autres renseignements que la taille et le

PB à l'entrée (et sur l'ensemble des paramètres à l'entrée). Aussi, on remarque que les oedèmes sont les grands oubliés, surtout à la sortie[10].

Pour synthétiser, on observe que tous les paramètres d'entrée et de sortie du STA sont renseignés dans seulement 3.5% des fiches dans le groupe de contrôle et 0.6% des fiches dans le groupe d'intervention, en 2017 ; par rapport à 2014, on observe une forte régression dans les deux groupes, due à la nouvelle pratique répandue de ne pas renseigner les oedèmes à la sortie. En revanche, on observe une amélioration dans le renseignement des paramètres à l'entrée : tous les paramètres d'entrée sont renseignés dans 56.3% des CDS du groupe de contrôle et 79.0% dans le groupe d'intervention : on observe ici une influence positive (+18.7%pts, p=0.03) du FBP Nutrition (cf. Tableau 23). Ces résultats sur l'admission font sens dans la mesure où la grille qualitative du FBP Nutrition contient des vérifications du respect des critères d'admission. En revanche, le manque de renseignements à la sortie (notamment des oedèmes) n'est pas cohérent avec l'existence d'indicateurs de bon remplissage du registre et de bonne prise en charge de 20 cas dans la grille qualitative, obtenant pourtant de bons scores en moyenne dans le groupe d'intervention (cf. Tableau 60, annexe). A nouveau, l'impact de la grille qualitative et la qualité des évaluations qualitatives sont mis en doute par ces résultats ; une attention particulière doit être portée sur les oedèmes à la sortie.

[10] Cette chute dans le renseignement des oedèmes, dans les deux groupes, à la sortie est difficilement explicable, le nouveau protocole de prise en charge de la malnutrition les prenant toujours en compte : que ce soit dans le texte, dans les fiches ou les registres, l'état des oedèmes à la sortie est important pour statuer sur l'état de l'enfant et dispose d'une place à part entière dans les registres et fiches spécifiques au STA.

Tableau 21. Remplissage des fiches cliniques SSN, en 2014 et 2017

% de non renseigné pour		Groupe de controle			Groupe d'intervention			Impact FBP Nutrition
		Moy	N	N att	Moy	N	N att	
Le poids à l'entrée	2014	0.6%	484	540	1.6%	487	540	n.s.
	2017	0.6%	156	540	0.0%	518	540	
	Diff	=			-1.6pp			
La taille à l'entrée	2014	1.9%	484	540	5.3%	487	540	-7.7 pp p=0.023
	2017	6.4%	156	540	0.8%	518	540	
	Diff	+4.6pp			-4.6pp			
PB à l'entrée	2014	19.4%	484	540	15.8%	487	540	n.s.
	2017	7.7%	156	540	2.1%	518	540	
	Diff	-11.7pp			-13.7pp			
Œdème à l'entrée	2014	59.7%	484	540	59.1%	487	540	n.s.
	2017	66.0%	156	540	69.3%	518	540	
	Diff	+6.3pp			+10.2pp			
Raison de sortie	2014	36.4%	484	540	34.3%	487	540	n.s.
	2017	28.2%	156	540	36.7%	518	540	
	Diff	-8.2pp			+2.4pp			
Poids à la sortie	2014	8.9%	484	540	10.7%	487	540	n.s.
	2017	4.5%	156	540	4.4%	518	540	
	Diff	-4.4pp			-6.2pp			
Taille à la sortie	2014	59.7%	484	540	55.9%	487	540	n.s.
	2017	33.3%	156	540	23.0%	518	540	
	Diff	-26.4pp			-32.9pp			
PB à la sortie	2014	37.0%	484	540	36.6%	487	540	n.s.
	2017	18.6%	156	540	11.0%	518	540	
	Diff	-18.4pp			-25.5pp			
Œdème à la sortie	2014	68.0%	484	540	66.9%	487	540	n.s.
	2017	81.4%	156	540	78.2%	518	540	
	Diff	+13.4pp			+11.2pp			

Source : fiches SSN; n.s. signifie que l'impact est non significatif; pp signifie points de pourcentage.

Tableau 22. Remplissage des fiches cliniques STA, en 2014 et 2017

% de non renseigné pour		Groupe de controle			Groupe d'intervention			Impact FBP Nutrition
		Moy	N	N att	Moy	N	N att	
**Le poids à l'entrée**	2014	0.6%	478	540	0.8%	485	540	n.s.
	2017	0.2%	522	540	0.6%	524	540	
	Diff	-0.4pp			-0.3%			
**La taille à l'entrée**	2014	1.5%	478	540	6.4%	485	540	-18.7 pp p=0.005
	2017	19.3%	522	540	5.5%	524	540	
	Diff	+17.9pp			-0.9pp			
**PB à l'entrée**	2014	12.6%	478	540	13.6%	485	540	-8.8 pp p=0.090
	2017	19.2%	522	540	9.9%	524	540	
	Diff	+6.6pp			-3.7pp			
**Œdème à l'entrée**	2014	38.5%	478	540	30.3%	485	540	n.s.
	2017	29.1%	522	540	14.9%	524	540	
	Diff	-9.4pp			-15.4pp			
**Raison de sortie**	2014	26.4%	478	540	35.5%	485	540	n.s.
	2017	25.9%	522	540	33.2%	524	540	
	Diff	-0.5pp			-2.3pp			
**Poids à la sortie**	2014	7.7%	478	540	4.5%	485	540	n.s.
	2017	2.1%	522	540	1.9%	524	540	
	Diff	-5.6pp			-2.6pp			
**Taille à la sortie**	2014	52.9%	478	540	57.5%	485	540	n.s.
	2017	89.1%	522	540	88.5%	524	540	
	Diff	+36.2pp			+31.0pp			
**PB à la sortie**	2014	29.7%	478	540	33.2%	485	540	n.s.
	2017	14.2%	522	540	11.5%	524	540	
	Diff	-15.5pp			-21.7pp			
**Œdème à la sortie**	2014	51.5%	478	540	43.9%	485	540	n.s.
	2017	94.8%	522	540	96.6%	524	540	
	Diff	+43.4pp			+52.6pp			

Source : fiches STA; n.s. signifie que l'impact est non significatif; pp signifie points de pourcentage.

Tableau 23. Tableau synthétique – sur le remplissage des fiches SSN et STA

		Groupe de controle			Groupe d'intervention			Impact FBP Nutrition
		Moy	N	N att	Moy	N	N att	
**Paramètres d'entrée en SSN tous renseignés**	2014	37.81%	484	540	36.96%	487	540	n.s.
	2017	33.33%	156	540	29.73%	518	540	
	Diff	-4.48pp			-7.23pp			
**Paramètres de sortie du SSN tous renseignés**	2014	9.09%	484	540	10.88%	487	540	n.s.
	2017	13.46%	156	540	15.64%	518	540	
	Diff	+4.37pp			+4.75pp			
**Paramètres d'entrée et de sortie du SSN tous renseignés**	2014	8.88%	484	540	10.88%	487	540	n.s.
	2017	13.46%	156	540	15.25%	518	540	
	Diff	+4.58pp			+4.37pp			
**Paramètres d'entrée du STA tous renseignés**	2014	55.44%	478	540	60.21%	485	540	+18.71pp P=0.032
	2017	56.32%	522	540	79.01%	524	540	
	Diff	+0.88pp			+18.80pp			
**Paramètres de sortie du STA tous renseignés**	2014	23.85%	478	540	14.23%	485	540	n.s.
	2017	3.45%	522	540	0.57%	524	540	
	Diff	-20.40pp			-13.65pp			
**Paramètres d'entrée et de sortie du STA tous renseignés**	2014	23.43%	478	540	13.81%	485	540	n.s.
	2017	3.45%	522	540	0.57%	524	540	
	Diff	-19.98pp			-13.24pp			

Source : fiches SSN et STA; n.s. signifie que l'impact est non significatif; pp signifie points de pourcentage.

4.4 Impact sur les indicateurs d'output

Au niveau des centres de santé

Suivi de la croissance

Selon les responsables nutrition des CDS, environ 82% des CDS délivraient des séances de suivi de la croissance dans les deux groupes en 2014 ; ce chiffre a fortement diminué et est passé à 51% dans le groupe de contrôle tandis qu'il s'est maintenu à 78% dans le groupe d'intervention[11]. Cela étant, en 2017, la courbe de croissance n'a été évoquée en consultation que dans 21 cas (soit 4% des consultations). C'est un peu moins bien qu'en 2014 et on ne dénote pas d'impact significatif du FBP Nutrition[12] (cf. Tableau 24).

Dans le questionnaire administré aux ménages, on a vérifié la présence de courbes de croissance dessinées sur le carnet de l'enfant. Il semble qu'il y ait eu une diminution de dessins de courbes dans les deux groupes : on est passé à seulement 1.2% d'enfants avec courbe dessinée dans le carnet dans le groupe de contrôle, et 4.6% dans le groupe de traitement, par rapport à 8.1% et 12.7% resp. en 2014 (cf. Tableau 24).

Ce non-impact du FBP Nutrition sur les activités de suivi de la croissance est d'autant plus déroutant que le FBP Nutrition contient un indicateur quantitatif dénommé « nombre de suivis de la croissance », pour lequel la vérification consiste à vérifier l'existence (et la tendance) des courbes. Or les données de routine suggèrent que cette activité a été en forte augmentation sur la période de l'intervention, passant de 0 cas validés en moyenne par mois et par centre de santé lors du premier semestre 2015, à 1,449 cas validés en moyenne par moi et par centre de santé lors du deuxième semestre 2016 (cf. Tableau 61, annexe). Ceci interroge sur la qualité des vérifications relatives à cet indicateur.

Détection de la MA

<u>De meilleurs et plus nombreux dépistages d'enfants malnourris par les CDS ?</u>

En 2017, lors des 529 consultations observées, au total 18 cas ont été diagnostiqués par les AS comme souffrant de malnutrition aigue (soit env. 3.4%). Parmi eux, 5 étaient des faux positifs selon les mesures anthropométriques prises par les enquêteurs (cf. Tableau 25). Par ailleurs, 70 cas (13.7%) souffraient de malnutrition aigue (selon ces mêmes mesures) mais n'ont pas été diagnostiqués comme tels en consultation (12.6% dans le groupe de contrôle et 14.8% dans le groupe d'intervention, cf. Tableau 25[13]). Les régressions logistiques ne montrent pas d'impact significatif du FBP Nutrition ni sur le taux de diagnostic malnutrition ni sur le taux de faux positifs ; par contre, elles montrent un impact significatif

---

[11] On suppose que ce résultat non cohérent avec les résultats des deux autres indicateurs relatifs à la courbe de croissance vient du fait que ces séances sont normalement inscrites dans le registre de surveillance infantile, aussi utilisé et rempli à chaque séance de vaccination.

[12] On devait initialement verifier la presence de courbe de croissances dans les carnets à la sortie des consultations (S) mais un filtre mal placé a fait que cela n'a jamais été vérifié en 2017 ; et ce n'est donc pas possible de réaliser cette double analyse.

[13] Noter que, très logiquement, le taux de faux négatifs par agent de santé est significativement corrélé avec la performance des agents de santé en matière de nutrition, telle que mesurée avec les vignettes en 2014 et 2017 (coef.=-0.12, N=316). En effet, si les agents ne posent pas les questions et n'effectuent pas les examens nécessaires à évaluer le statut nutritionnel des enfants, il est peu probable qu'ils puissent poser un diagnostic quel qu'il soit en matière de nutrition.

(p<5%) sur le taux de faux négatifs : il semblerait que l'intervention ait fait augmenter ce taux de faux négatifs de 12 points de pourcentage[14].

Tableau 24. Suivi de la croissance en 2014 et 2017

		Groupe de controle			Groupe d'intervention			Impact FBP Nutrition
		Moy	N	N att	Moy	N	N att	
**Courbe évoquée en consultation** (source = C)	2014	6.2%	260	270	8.3%	254	270	n.s.
	2017	3.8%	263	270	4.2%	265	270	
	Diff	-2.4pp			-4.1pp			
**Existence de séances de suivi de la croissance** (source = N)	2014	80.0%	45	45	84.4%	45	45	n.s.
	2017	51.1%	45	45	77.8%	45	45	
	Diff	-28.9pp			-6.7pp			
**Courbe présente sur le carnet** (source = données ménages)	2014	8.1%	2472	3240	12.7%	2445	3240	n.s.
	2017	1.2%	2946	3240	4.6%	2941	3240	
	Diff	-6.9pp			-8.2pp			

Note : n.s. signifie que l'impact est non significatif; pp signifie points de pourcentage.

[14] Des analyses économétriques supplémentaires ont été réalisées et suggèrent que le respect des normes nationales en termes de ressources humaines a un impact significatif sur le taux de faux négatifs (le respect réduirait ce taux de 7.2pp, p<0.05, tableaux disponibles sur demande).

Tableau 25. Dépistages de malnutrition aigue observés en consultations curatives

		Groupe de controle			Groupe d'intervention			Impact FBP Nutrition
		Moy	N	N att	Moy	N	N att	
**Diagnostic de MA**	2014	3.46% (n=9)	260	270	1.97% (n=5)	254	270	n.s.
	2017	3.42% (n=9)	263	270	3.40% (n=9)	265	270	
	Diff	-0.04pp			+1.43pp			
**Diagnostic de MA - faux positif**	2014	1.00% (n=2)	205	-	1.33% (n=3)	226	-	n.s.
	2017	0.45% (n=1)	223	-	1.80% (n=4)	260	-	
	Diff	-0.55pp			+0.47pp			
**Diagnostic de MA - faux négatif**	2014	86.54% (n=45)	52	-	92.59% (n=25)	27	-	+12.13pp P=0.027
	2017	80.00% (n=32)	40	-	88.37% (n=38)	43	-	
	Diff	-6.54pp			-4.22pp			

Source de données : observations des consultations (questionnaire C) ; n.s. signifie que l'impact est non significatif ; pp signifie points de pourcentage.

A partir des fiches SSN et STA, on peut vérifier si les cas enregistrés (et retranscrits) ont été bien diagnostiqués selon les informations rapportées sur les fiches cliniques (c'est-à-dire : est-ce que les critères d'entrée dans le service sont respectés ?). Les critères d'entrée dans le SSN sont respectés seulement à hauteur de 44% dans le groupe de traitement contre 31% dans le groupe de contrôle : cela est plutôt stagnant depuis 2014 et il ne semble pas y avoir eu d'influence de l'intervention. Par ailleurs, parmi les cas entrés en SSN, selon les paramètres disponibles (poids, taille, PB, œdème), environ 38.4% auraient du être transférés au STA voire SST pour malnutrition aigue sévère. Ce problème a stagné dans le groupe de contrôle et est légèrement amélioré par rapport à 2014 dans le groupe d'intervention (-8.8%pts, cf. Tableau 26). Les paramètres d'entrée en STA sont corrects dans une grande majorité des cas (90% dans les deux groupes) ; on note une légère détérioration par rapport à 2014 : -5%pts dans les deux groupes (cf. Tableau 26).

Tableau 26. Les paramètres d'entrée dans les programmes SSN et STA en 2014 et 2017

		Groupe de controle			Groupe d'intervention			Impact FBP Nutrition
		Moy	N	N att	Moy	N	N att	
**Paramètres d'entrée dans le SSN corrects**	2014	39.4%	264	540	40.0%	265	540	n.s.
	2017	31.3%	128	540	43.8%	256	540	
	Diff	-8.1pp			+3.8pp			
**Paramètres d'entrée dans le SSN auraient dû amener au STA***	2014	38.9%	211	540	46.6%	219	540	n.s.
	2017	40.5%	74	540	37.7%	212	540	
	Diff	1.7pp			-8.8pp			
**Paramètres d'entrée dans le STA corrects**	2014	95.3%	448	540	94.7%	449	540	n.s.
	2017	90.1%	454	540	89.3%	496	540	
	Diff	-5.2pp			-5.3pp			

Sources : SSN et STA (quand les informations nécessaires pour identifier un cas de malnutrition aigu modéré ou sévère sont disponibles). Notes : n.s. signifie que l'impact est non significatif; pp signifie points de pourcentage ; * Lorsqu'on n'a pas l'information sur l'oedème, on ne peut pas savoir si l'enfant aurait du aller en STA, il s'agit donc d'informations manquantes – c'est pour cela qu'on a davantage d'observations pour la ligne sur les "paramètres d'entrée dans le SSN corrects" que pour la ligne "paramètres d'entrée SSN auraient du amener à STA".

Davantage d'enfants inscrits en SSN et en STA ?

Lors de la retranscription des fiches SSN et STA, les enquêteurs devaient indiquer le nombre de fiches cliniques correspondant (en théorie) au nombre de cas entrés et sortis de ces deux programmes durant la période de six mois précédant l'enquête : ceci permet d'avoir une idée du niveau d'activité dans ces deux services.

Dans le groupe de traitement, le nombre de cas d'enfants enregistrés dans le SSN durant la période de six mois la plus récente est passé de 33 en moyenne en 2014 à 122 en moyenne en 2017 ; alors que dans le groupe de contrôle on observait une diminution (de 62 à 13 cas, cf. Tableau 27). Ceci suggère un impact significatif du FBP Nutrition sur le nombre d'enfants traités pour MAM. Les régressions économétriques de double différence confirment un impact du FBP Nutrition augmentant le nombre de cas enregistrés de 138 en moyenne (p=0.000).

Le nombre de cas d'enfants enregistrés dans le STA durant la période de six mois la plus récente est passé de 25 en moyenne en 2014 à 79 en moyenne en 2017 dans le groupe d'intervention ; alors que dans le groupe de contrôle on observait une stagnation (de 40 à 42 cas, cf. Tableau 27). Ceci suggère un impact significatif du FBP Nutrition sur le nombre d'enfants traités pour MAS. Les régressions économétriques de double différence confirment un impact du FBP Nutrition augmentant le nombre de cas enregistrés de 53 en moyenne (p=0.000). Il ne semble pas que ce résultat soit dû à un relâchement relatif aux critères d'entrée dans les programmes (cf. ci-dessus).

Ces observations sont croisées avec celles issues des données de routine du projet FBP Nutrition, à savoir les volumes d'activité rapportés, vérifiés et validés en ce qui concerne les quatre indicateurs rémunérés dans le FBP Nutrition : nombre de cas de MAM guéris, nombre de cas de MAS guéris, nombre de références de cas de MA, nombre de suivi de la croissance réalisés. Ces données ne sont par définition disponibles que pour le groupe d'intervention.

Ainsi, dans le groupe d'intervention, on observe également une augmentation du nombre de cas traités et guéris (et validés) avec ces données de routine (cf. Tableau 61, annexe). Il est à noter par ailleurs que les données de routine du projet FBP Nutrition, validées, suggèrent un nombre beaucoup plus important de cas guéris par période de six mois dans le groupe d'intervention: au dernier semestre 2016, le nombre total de cas de MAM guéris et validés s'élevait à env. 306 par CDS, ce qui est plus du double de ce qu'on observe lors de l'enquête finale (122 cas de MAM traités en moyenne par semestre et par CDS). Idem pour le nombre de cas de MAS guéris : les données de routine suggèrent 171 cas de MAS guéris par CDS, contre 79 cas de MAS traités par CDS et par semestre pour les données d'enquête (cf. Tableau 61 en annexe, versus Tableau 27).

Tableau 27. Nombre moyen de cas enregistrés dans les services SSN et STA sur la période de six mois précédant l'enquête, en 2014 et 2017

		Groupe de contrôle					Groupe d'intervention					Impact
		**Moy**	**Min**	**Max**	**Mediane**	**N**	**Moy**	**Min**	**max**	**Mediane**	**N**	
**Nb de fiches SSN**	2014	62.0	0	682	29	45	33.4	0	209	20	45	+138.2 P=0.000
	2017	12.7	0	116	0	45	122.2	0	956	66	45	
	diff	-49.3			-29		88.8			+46		
**Nb de fiches STA**	2014	40.1	0	153	33	45	24.9	0	87	17	45	+52.6 P=0.000
	2017	41.5	0	229	28	45	78.9	0	289	62	45	
	diff	1.4			-5		+54.0			+45		

Source : questionnaire SSN et STA ; N attendu correspond au N obtenu dans tous les cas.

Comment expliquer que le nombre de cas inscrits dans les programmes de prises en charge de la malnutrition aigue augmente avec le FBP Nutrition quand le dépistage en consultation ne connaît pas d'amélioration ? Peu de pistes pour le comprendre. La provenance des cas est rarement indiquée sur les fiches SSN (moins de 10% des cas en 2017) ; pour le peu dont on dispose, on voit que la majorité des cas proviennent soit du dépistage dit « spontané » soit des transferts provenant du STA. En ce qui concerne les fiches STA, l'information est davantage disponible, et on voit que la grande majorité des cas proviennent soit du dépistage dit « spontané » soit du dépistage dit « actif » [15].

Prise en charge de la MAM (traitement)

D'après le Tableau 28 page suivante, il semble qu'il y ait eu une amélioration en terme de traitement systématique ou diététique dû au FBP Nutrition. Néanmoins, on doit faire attention à deux points ne permettant pas d'évaluer les effets du FBP Nutrition. En effet, (1) on a très peu de fiches SSN dans le

[15] Un cas dit « spontané » est un cas arrivé de soi au CDS, tandis qu'un dépistage dit « actif » suggère que les agents de santé se sont déplacés vers les communautés pour le dépistage.

groupe de contrôle (et seulement 14 informent sur le traitement systématique), et (2) l'information sur le traitement systématique a été collectée d'une manière différente dans les deux enquêtes ce qui rend les comparaisons non pertinentes.

Prise en charge de la MAS (traitement)

D'après le tableau suivant, on observe une chute du traitement systématique d'al/mebendazole et d'amoxicilline dans les deux groupes (de 44% à 35% dans le groupe de contrôle, et de 45% à 24% dans le groupe d'intervention). Ceci est dû essentiellement à une chute du traitement d'al/mebendazole (problème d'approvisionnement ?). Il ne semble pas y avoir d'effet attribué au FBP Nutrition. Par ailleurs, le nombre de visites sans nourriture a diminué dans les deux groupes, et ce, davantage dans le groupe de contrôle, ce qui suggèrerait un effet négatif du FBP Nutrition sur cet indicateur. Néanmoins, comme pour la prise en charge de la MAM, on doit faire attention au fait que l'information sur le traitement systématique a été collecté d'une manière différente dans les deux enquêtes ce qui rend les comparaisons non pertinentes.

Tableau 28. Traitements systématique et diététique reçus par les enfants traités dans le SSN

		Groupe de contrôle			Groupe d'intervention			Impact du FBP Nutrition
		Moy	N	N att	Moy	N	N att	
**Traitement d'al/me bendazole reçu**	2014	19%	484	540	20%	487	540	n.a.
	2017	86%	14	540	87%	226	540	
	Diff	n.a.			n.a.			
**Traitement de vitamine A reçu**	2014	13%	484	540	7%	487	540	n.a.
	2017	79%	14	540	45%	226	540	
	Diff	n.a.			n.a.			
**Traitement d'acide folique reçu**	2014	13%	484	540	4%	487	540	n.a.
	2017	64%	14	540	35%	226	540	
	Diff	n.a.			n.a.			
**Nb de visites sans nourriture % Nb de visites**	2014	27%	484	540	40%	487	540	n.a.
	2017	13%	141	540	29%	459	540	
	Diff	n.a.			n.a.			

Source : fiches SSN. Note : les comparaisons 2014-2017 et le calcul de la double différence ne sont pas réalisables car la collecte des informations sur le traitement s'est faite de manière différente ; n.a. signifie non applicable (car les comparaisons entre 2014 et 2017 sont non pertinentes, cf. texte).

Tableau 29. Traitements systématique et diététique reçus par les enfants traités dans le STA

		Groupe de contrôle			Groupe d'intervention			Impact du FBP Nutrition
		Moy	N	N att	Moy	N	N att	
Traitement de vitamine A reçu	2014	34%	337	540	42%	342	540	n.a.
	2017	22%	482	540	12%	512	540	
	Diff	n.a.			n.a.			
Traitement d'al/me bendazole & amoxicilline reçu	2014	44%	337	540	45%	342	540	n.a.
	2017	35%	482	540	24%	512	540	
	Diff	n.a.			n.a.			
Nb de visites sans nourriture % Nb de visites	2014	26%	476	540	17%	484	540	n.a.
	2017	13%	449	540	14%	435	540	
	Diff	n.a.			n.a.			

Source : fiches STA. Note : les comparaisons 2014-2017 et le calcul de la double différence ne sont pas réalisables car la collecte des informations sur le traitement s'est faite de manière différente ; n.a. signifie non applicable (car les comparaisons entre 2014 et 2017 sont non pertinentes, cf. texte)

Au niveau de la communauté

Le résultat trouvé au niveau des centres de santé, selon lequel le nombre d'inscrits a augmenté davantage dans le groupe d'intervention, est cohérent avec ce qu'on trouve au niveau des données ménages. En effet, le taux de prise en charge des cas de malnutrition aigue a augmenté, et ce dans les deux groupes : à la baseline, 8.6% des enfants souffrant de malnutrition aigue étaient pris en charge dans un SSN ou un STA (de façon relativement homogène dans les groupes de contrôle et d'intervention) ; à la endline, ce taux est passé à 13.8% dans le groupe de contrôle et à 17.8% dans le groupe d'intervention, ce qui suggère un impact positif du FBP Nutrition sur le taux de prise en charge, néanmoins faible et non significatif (cf. Tableau 30).

Tableau 30. Pourcentage d'inscrits en SSN ou STA dans la communauté, parmi les enfants identifiés comme souffrant de malnutrition aigue par les enquêteurs

		Groupe de contrôle			Groupe d'intervention			Impact FBP Nutrition
		Moyenne	N	N attendu	Moyenne	N	N attendu	
Inscrit en SSN ou en STA	2014	8.81%	193	3240*taux MA	8.38%	179	3240*taux MA	n.s.
	2017	13.78%	283	3240*taux MA	18.79%	282	3240*taux MA	
	Diff	+4.97pp			+10.41pp			

Source : données ménages; n.s. signifie que l'impact est non significatif; pp signifie points de pourcentage.

Nous n'avons pas collecté d'informations au niveau des GASC. Cependant, dans le questionnaire administré aux ménages en 2017, une question était relative à la participation des ménages à des séances de sensibilisation à la nutrition ou de démonstration culinaire. Environ 22% des ménages ont dit avoir participé à une séance de ce type ; 19.4% dans le groupe de contrôle et 23.8% dans le groupe

d'intervention : la différence de 4.3 points de pourcentage entre les deux groupes est significative ce qui suggère qu'il y a eu davantage de familles ciblées à travers les séances organisées dans le groupe d'intervention (cf. Tableau 31).

Tableau 31. Séances de sensibilisation et culinaires effectuées par les ASC, en 2017 seulement

	Groupe de contrôle			Groupe d'intervention			Différence
	Moy	N	N att	Moy	N	N att	
**Participation à une séance de sensibilisation à la nutrition ou de démonstration culinaire**	19.4%	3234	3240	23.8%	3246	3240	4.3pp P=0.000
**Nombre de séances de sensibilisation**	2.44	611	-	2.29	745	-	n.s.
**Nombre de séances de démonstration culinaire**	1.77	603	-	1.87	751	-	n.s.

Source : données ménages; n.s. signifie que la différence entre le groupe de contrôle et le groupe de traitement est non significative; pp signifie points de pourcentage.

4.5 Impact sur les indicateurs de résultat

Au niveau des centres de santé

Au niveau du centre de santé, nous souhaitions comprendre si l'introduction de critères nutrition dans le programme de FBP existant apporterait de meilleurs résultats au niveau du centre de santé, i.e. une meilleure prise en charge des cas de malnutrition (taux de guérison, durée de périodes de traitement, etc.).

Prise en charge de la MAM (taux de guérison, gain de poids et durée traitement chez les guéris)

On trouve un impact significatif sur le taux de guérison : le taux de guérison est passé de 84% à 97% dans le groupe de traitement et de 76% à 78% dans le groupe de contrôle, ce qui donne un effet marginal moyen de 15 points de pourcentage (p=0.007, cf. Tableau 32). Ceci suggère que le FBP Nutrition aurait pour effet d'augmenter le taux de guérison de 15 points de pourcentage. Néanmoins, comme dit plus haut, très peu de fiches ont été collectées côté groupe de contrôle (156 cas, versus 518 cas côté groupe de traitement), ce qui suggère qu'on a un biais de sélection qui fait que le niveau de l'impact est potentiellement sous-estimé : l'effet serait probablement plus important si on avait les FOSA qui n'ont pas rempli du tout de fiches – si on considère que le biais de sélection a en effet gardé les FOSA les plus sérieuses dans le domaine de la nutrition et/ou celles aidées par des ONGs tierces (la majorité de ces fiches viennent de Rutana (60) et Ruyigi (24), provinces aidées par le PAM).

Par ailleurs, il ne semble pas que l'effet soit « falsifié » dans le sens où les paramètres rapportés à la sortie du SSN permettent de vérifier que 75% des cas rapportés comme « guéris » le sont effectivement, et ce dans les deux groupes : il y a une amélioration depuis 2014 mais il y a encore 25% des cas qui sortent « guéris » alors qu'ils ne le sont pas d'après les paramètres anthropométriques rapportés à la sortie (cf. Tableau 32).

La durée de traitement chez les guéris a également été significativement réduite dans le groupe de traitement : on est passé de 78 jours à 44 jours de traitement en moyenne, soit une réduction de 29 jours (p=0.047, cf. Tableau 32). Cela suggère que le service est devenu performant dans le groupe de traitement grâce au FBP Nutrition.

Suivant cette réduction de la durée de traitement, le gain de poids relatif a logiquement augmenté dans le groupe de traitement ; mais la double différence avec avant et avec le groupe de contrôle n'est pas significative.

Enfin, concernant les variables secondaires[16], on observe :

- Pas d'impact significatif sur le taux d'abandon (qui est à 2%).
- Pas d'impact significatif sur le transfert vers SSt ou STA (2%)
- Un impact significatif sur les non répondants de -4.2%pts (p=0.057).

Des analyses économétriques supplémentaires ont été réalisées mais avec le manque de données sur certaines variables (notamment sur la disponibilité d'intrants ou le calcul des indicateurs de performance), les résultats qu'on peut en tirer sont limités (tableaux disponibles sur demande). Ils suggèrent que la complétude et la qualité de l'équipement jouent un rôle certain sur le taux de guérison de MAM ; ils suggèrent par ailleurs qu'il n'y a pas eu de quelconque contamination de l'intervention au sein des districts où il y avait deux types de centres de santé (contrôle et intervention).

Tableau 32. Performance des SSN en 2014 et 2017

		Groupe de contrôle			Groupe d'intervention			Impact
		Moy	N	N att	Moy	N	N att	
**Taux de guérison**	2014	76%	308	540	84%	320	540	+14.7pp P=0.007
	2017	78%	112	540	97%	327	540	
	Diff	+2 pp			+13 pp			
**Gain de poids relatif chez les guéris (g/kg et jour)**	2014	1.39	210	410*	2.06	227	454*	n.s.
	2017	2.12	77	421*	5.50	283	524*	
	Diff	+0.73			+3.44			
**Durée de traitement chez les guéris**	2014	70.79	225	410*	78.08	252	454*	-29.3 P=0.047
	2017	70.26	77	421*	43.96	288	524*	
	Diff	-0.53			-34.12			
**Paramètres de sortie du SSN « guéri » corrects**	2014	65.8%	234	410*	64.6%	268	454*	n.s
	2017	74.7%	87	421*	74.1%	317	524*	
	Diff	+8.9pp			+9.6pp			

Source : fiches SSN; n.s. signifie que l'impact est non significatif; pp signifie points de pourcentage ; * Pour les variables gain de poids, durée et paramètres de sortie correct, le N attendu est estimé à 540 multiplié par le taux de guérison.

[16] Tableau disponible sur demande.

Prise en charge de la MAS (taux de guérison, gain de poids et durée du traitement chez les guéris)

Là, on n'a pas de problème de biais de sélection : environ le même nombre de fiches collectées de chaque côté (n=522 vs 524[17]).

On ne trouve pas d'impact significatif sur le taux de guérison : le taux de guérison est passé de 84% à 92% dans le groupe de traitement et de 87% à 93% dans le groupe de contrôle. Par ailleurs, il semble que les paramètres rapportés à la sortie « guéri » du STA correspondent davantage à des cas guéris, et ce dans les deux groupes. Néanmoins l'amélioration du respect des critères de sortie est plus importante dans le groupe de traitement, et on observe un impact significatif du FBP Nutrition de +19.5 points de pourcentage (p=0.036).

La durée de traitement chez les guéris a également été significativement réduite dans le groupe de traitement : on est passé de 61 jours à 43 jours de traitement en moyenne, soit une réduction de 20 jours (p=0.021). Cela suggère que le service est devenu performant dans le groupe de traitement grâce au FBP Nutrition.

Suivant cette réduction de la durée de traitement, le gain de poids relatif a logiquement augmenté dans le groupe de traitement ; mais la double différence avec avant et avec le groupe de contrôle n'est pas significative.

Enfin, concernant les variables secondaires[18], on observe :

- Pas d'impact significatif sur le taux d'abandons (qui est à 2%).
- Pas d'impact significatif sur le transfert interne (2%)
- Pas d'impact significatif sur les décès (1%)
- Pas d'impact significatif sur les non répondants (2%)

On observe par ailleurs une corrélation significative parmi les centres de santé observés en 2014 et 2017 entre le taux de guérison de la MAM et le taux de guérison de la MAS (coef. corr.=0.44, N=100, $p<0.05$) : cela suggère de bonnes synergies entre les deux services SSN et STA.

Des analyses économétriques supplémentaires ont été réalisées mais avec le manque de données sur certaines variables, les résultats qu'on peut en tirer sont limités (tableaux disponibles sur demande). Ils suggèrent que la complétude de l'équipement ainsi que la disponibilité des intrants nutritionnels jouent un rôle certain sur le taux de guérison de MAS ; ils suggèrent par ailleurs qu'il n'y a pas eu de quelconque contamination de l'intervention au sein des districts où il y avait deux types de centres de santé (contrôle et intervention).

---

[17] Noter que les variables de performance sont calculées sur un plus petit nombre de fiches : les fiches pour lesquelles on n'a pas l'information nécessaire (e.g. guéri ou pas à la sortie) sont exclues ; de plus, les variables gain de poids, durée de traitement et paramètre de sortie corrects sont calculées seulement pour les cas raportés comme guéris. Cela explique les différences de nombre d'observations.

[18] Tableau disponible sur demande.

Tableau 33. Performance des STA en 2014 et 2017

		Groupe de contrôle			Groupe d'intervention			Impact
		Moyenne	N	N att	Moyenne	N	N att	
**Taux de guérison**	2014	87%	352	540	84%	313	540	n.s.
	2017	93%	387	540	92%	350	540	
	Diff	+6 pp			+8 pp			
**Gain de poids relatif chez les guéris (g/kg et jour)**	2014	4.13	289	470*	3.68	247	454*	n.s.
	2017	3.77	315	502*	4.62	278	497*	
	Diff	-0.36			+0.94			
**Durée de traitement chez les guéris**	2014	56.97	304	470*	61.19	252	454*	-19.5 P=0.021
	2017	59.06	317	502*	42.87	282	497*	
	Diff	+2.09			-18.32			
**Paramètres de sortie du STA « guéri » corrects**	2014	46.1%	306	470*	46.6%	262	454*	+19.5pp P=0.036
	2017	93.3%	359	502*	98.8%	322	497*	
	Diff	+47.2pp			+52.2pp			

Source : Fiches STA; n.s. signifie que l'impact est non significatif; pp signifie points de pourcentage; * Pour les variables gain de poids, durée et paramètres de sortie correct, le N attendu est estimé à 540 multiplié par le taux de guérison.

Au niveau de la communauté

Résultat intermédiaire

En toute logique, étant données les pratiques en consultations (cf. Tableau 10 à Tableau 15, pages 42-45), il ne semble pas que le FBP Nutrition ait pu avoir un quelconque impact sur les connaissances et pratiques des mamans envers leurs tout-petits.

En ce qui concerne les pratiques alimentaires, en 2017, sur l'ensemble de l'échantillon (n=5822), environ 51% des enfants reçoivent le bon nombre de groupes alimentaires et 40% ont une fréquence de repas correcte ; le résultat est que seulement 25% des enfants reçoivent une alimentation correcte dans l'ensemble de l'échantillon ; ce qui n'est pas mieux qu'en 2014 (cf. Tableau 34).

En ce qui concerne les connaissances des mamans, on a demandé aux mamans de nous dire quels étaient les signes de malnutrition qu'elles connaissaient. En 2017, sur l'ensemble de l'échantillon, environ 9% des femmes ne connaissaient aucun signe, tandis que 3% pouvaient citer les cinq signes que sont : enfant amaigri, visage vieux, présence d'œdème, enfant apathique, cheveux roux. Tout comme en 2014, la majorité des femmes pouvaient citer deux ou trois de ces signes (tableau disponible sur demande).

On a par ailleurs créé une variable de qualité de jugement de l'état de malnutrition de l'enfant, comparant le jugement de la mère à l'état objectif de l'enfant considérant les mesures anthropométriques des enquêteurs. Il se trouve qu'en 2017 environ 59% des mères jugent correctement l'état nutritionnel de leur enfant ; cela signifie qu'enfiron 41% des femmes se trompent ; ce résultat est quasiment similaire à 2014 et on ne perçoit aucun impact du FBP Nutrition ici (cf. Tableau 35).

On a également demandé aux mamans quels conseils d'allaitement et d'alimentation des bébés de plus de six mois elles donneraient à de jeunes mamans. En 2017, environ 9% des femmes n'avaient cité aucun conseil d'allaitement ; tout comme en 2014, la grande majorité des femmes ont cité entre un et quatre conseils (sur les treize possibles). De même pour les conseils relatifs à l'alimentation du bébé de plus de six mois, environ 5% des femmes n'avaient aucun conseil à donner ; la grande majorité des femmes ont cité entre un et quatre conseils (sur les douze) arrivant à une moyenne de trois conseils par femme (tableau disponible sur demande).

Tableau 34. Pratiques alimentaires des enfants dans la communauté, en 2014 et 2017

		**Groupe de contrôle**			**Groupe d'intervention**			**Impact FBP Nutrition**
		Moyenne	N	N att	Moyenne	N	N att	
**Nb de groupes alimentaires OK**	2014	48.1%	2779	3240	50.5%	2763	3240	n.s.
	2017	50.6%	2882	3240	52.4%	2940	3240	
	Diff	+2.5pp			+2.0pp			
**Fréquence repas OK**	2014	42.9%	3085	3240	44.6%	3079	3240	n.s.
	2017	38.2%	3212	3240	42.4%	3228	3240	
	Diff	-4.8pp			-2.2pp			
**Alimentation OK**	2014	23.7%	3076	3240	26.3%	3068	3240	n.s.
	2017	23.6%	3209	3240	27.2%	3224	3240	
	Diff	-0.0pp			+0.9pp			

Source : données ménages; n.s. signifie que l'impact est non significatif; pp signifie points de pourcentage.

Tableau 35. Jugement sur l'état de malnutrition de l'enfant

		Groupe de contrôle			Groupe d'intervention			Impact FBP Nutrition
		Moyenne	N	N att	Moyenne	N	N att	
**Jugement correct considérant la malnutrition aigue seulement**	2014	65.9%	3097	3240	65.8%	3095	3240	n.s.
	2017	69.4%	3223	3240	70.4%	3236	3240	
	Diff	+3.5pp			+4.6pp			
**Jugement correct, considérant la malnutrition aigue et chronique**	2014	58.2%	3097	3240	58.4%	3095	3240	n.s.
	2017	59.4%	3223	3240	58.5%	3236	3240	
	Diff	+1.2pp			+0.1pp			

Source : données ménages; n.s. signifie que l'impact est non significatif; pp signifie points de pourcentage.

Résultat final

L'étude se focalisait sur la prévalence de la malnutrition aigüe parmi la population des enfants âgés de 6 à 23 mois comme résultat final. Celle-ci a augmenté sur la période : elle est passée de 6.3% à 8.8% dans le groupe de contrôle, et de 5.8% à 8.7% dans le groupe d'intervention. Aussi, l'analyse économétrique n'a pas détecté d'effet dû au FBP Nutrition.

Les analyses statistiques et économétriques réalisées sur les autres indicateurs primaires et secondaires de résultat final n'ont décelé aucun impact du FBP Nutrition (cf. tableau ci-dessous).

Des analyses additionnelles ont été réalisées pour tenter de percevoir un impact différencié selon les tranches d'âge des enfants ou selon leur niveau socio-économique. Mais rien de tout cela n'a été décelé (pas d'impact sur l'équité, tableaux disponibles sur demande).

Tableau 36. Statistiques descriptives des indicateurs de résultats au niveau de la communauté, par groupe d'intervention et par période

		Groupe de contrôle			Groupe d'intervention			Impact FBP Nutrition
		Moy	N	N att	Moy	N	N att	
**Prévalence de la malnutrition aigüe (z-score du poids pour taille<-2)**	2014	6.30%	3100	3240	5.80%	3099	3240	n.s.
	2017	8.80%	3234	3240	8.70%	3246	3240	
	Diff	+3pp			+3pp			
**Prévalence du retard de croissance (z-score de taille-pour-âge<-2**	2014	53.30%	3100	3240	53.60%	3099	3240	n.s.
	2017	49.90%	3234	3240	52.00%	3246	3240	
	Diff	-3pp			-2pp			
**Z-score du poids-pour-taille**	2014	-0.34	3100	3240	-0.34	3098	3240	n.s.
	2017	-0.47	3233	3240	-0.46	3246	3240	
	Diff	-0.13			-0.12			
**Z-score de taille-pour-âge**	2014	-2.10	3100	3240	-2.11	3099	3240	n.s.
	2017	-2.06	3230	3240	-2.08	3244	3240	
	Diff	+0.04			+0.03			
**Périmètre brachial**	2014	139.75	3100	3240	140.1	3099	3240	n.s.
	2017	137.94	3233	3240	138.25	3246	3240	
	Diff	-1.81			-1.85			
**Prévalence des épisodes infectieux durant les deux semaines précédant l'enquête**	2014	60.90%	3100	3240	57.50%	3099	3240	n.s.
	2017	56.50%	3234	3240	53.90%	3246	3240	
	Diff	-4pp			-4pp			

Source : données ménages; n.s. signifie que l'impact est non significatif; pp signifie points de pourcentage.

5. Discussion

Dans cette section, nous revenons sur les principaux résultats de l'étude (cf. aussi Tableau 63 en annexe) et explorons certaines explications possibles des résultats obtenus pour les principales catégories d'indicateurs.

5.1 Mise en œuvre de l'intervention

Toute EI d'une intervention complexe est soumise aux aléas des changements contextuels ou des défis de sa mise en œuvre. Au final, l'intervention réelle peut différer substantiellement de l'intervention telle qu'elle avait été imaginée au départ.

Notre analyse est que l'EI a bien été affectée par de tels facteurs externes et opérationnels.

Nous voulons d'abord souligner les efforts faits par le Ministère de la Santé et ses différentes composantes pour mettre en œuvre le FBP nutrition, en dépit d'un contexte national qui s'est fait fortement dégradé au cours de l'EI. Les activités préparatoires à la mise en place du FBP Nutrition ont bien eu lieu. C'est notamment le cas de la formation des acteurs tels que les CDS, les hôpitaux, les équipes de vérification. La mise en œuvre elle-même a été affectée par différentes contraintes. Il y a eu tout d'abord le retard dans la signature des contrats avec les formations sanitaires (qui ne comportaient pas par ailleurs la note explicative de l'intervention), mais aussi le retard et irrégularités de paiement des subsides aux FOSA, ainsi que le retard et irrégularités dans la disponibilité des intrants (voir plus loin). Il est clair que la baisse de l'appui international au niveau des intrants nutritionnels a affecté l'efficacité du FBP nutrition et la théorie de changement activée par ce dernier : il est difficile de motiver des formations sanitaires à prester des services de santé quand elles ne disposent pas des intrants indispensables.

Il nous faut reconnaitre que le Burundi est un contexte difficile pour mener de telles EI. Avec cette expérience, nous avons malheureusement rencontré les difficultés déjà rencontrées par ailleurs (Kandpal 2016). Il s'agit notamment de l'EI d'un projet pilote mené dans le district du Haut-Katanga en République démocratique du Congo où les problèmes de mise en œuvre ont provoqué une baisse significative des salaires des agents de santé dans les FOSA du FBR, entraînant une perte de motivation. De même comme le note l'évaluation d'impact en Afghanistan, la complexité de l'approche FBR et, en fait, de la formule de paiement elle-même peut empêcher la compréhension du mécanisme incitatif, ce qui empêche l'adoption d'un comportement adéquat en réponse au paiement incitatif de l'agent de santé (Kandpal 2016).

En plus de l'importance de la mise en œuvre, les leçons apprises des EI soulignent également l'importance d'une préparation systémique à une réforme relativement complexe du système de santé. En effet, les premiers résultats du travail qualitatif (non repris dans ce rapport) montrent que les messages et instructions relatifs à l'intervention communiqués lors des formations initiales ont parfois été trop peu partagés par les personnes formées au niveau de leur district ou CDS : «*…nous étions au courant de sa participation à la réunion de lancement mais il n'a jamais restitué et il n'a associé personne dans le remplissage des différents rapports hebdomadaires…*». Pourtant, cette restitution est

jugée utile dans d'autres cas de figure : *«…oui c'est la restitution qui m'a facilité de prendre correctement la relève lors de la mutation du titulaire du service….».*

5.2 Inputs et processus

Equipements et produits de traitement

Equipement

Pour que l'introduction de critères nutrition dans le programme de FBP existant se traduise en de meilleurs résultats au niveau du centre de santé (i.e. une meilleure prise en charge des cas de malnutrition (taux de guérison, durée de périodes de traitement, etc.), il y a nécessité que le centre de santé dispose d'un minimum d'équipements réquis et de bonne qualité, des intrants de traitement diététiques et systématiques.

L'enquête de 2017 montre que le problème fondamental de disponibilité de la totalité de l'équipement nécessaire pour la prise des mesures anthropométriques (dans un état fonctionnel) déjà observé dans les enquêtes de référence, n'a été réglé ni par une action externe (ex. dotation par le programme national et un de ses partenaires) ni par le FBP Nutrition lui-même (qui aurait induit les CDS à résoudre le problème par eux-mêmes). De même, les différences trouvées dans les prises de mesures du poids, effectuées par les enquêteurs à la sortie des consultations, entre la balance du CDS et la balance de l'enquête (SECA) demeurent, et même augmentent légèrement dans les deux groupes ce qui indique que le problème de manque de qualité du matériel subsiste (cf. Tableau 6 et Tableau 7, page 38).

On peut se poser la question du pourquoi les CDS n'ont pas fait la démarche de se procurer d'un équipement aussi important qui constitue la porte d'entrée dans le programme de prise en charge de la malnutrition. Disponibilité sur le marché ? Investissement jugé lourd ? Subsides jugées insuffisantes ?

D'après l'analyse des données de routine sur le score qualité (cf. Tableau 60 en annexe), dans le groupe d'intervention, les scores relatifs à la disponibilité et à la fonctionnalité du matériel sont en fait généralement bons : sur 285 occurrences, 213 ont un score de 20/20 sur cet indicateur pour le suivi de la croissance, 178 pour le SSN et 234 pour le STA. Cela suggère que le signal envoyé aux CDS est que ce déficit d'équipement n'est pas un si gros problème. Si la contrainte de l'équipement est réelle, dans la mesure où l'équipement actuel correspond rarement à ce qui est nécessaire[19], il faudrait peut-être revoir ces indicateurs et veiller à ce que le signal soit non équivoque.

---

[19] Pour réaliser les activités liées au suivi de la croissance des enfants, il faut au minimum le matériel suivant : Toise (min 2), Balance Salter (min 2), Pèse-bébé (min 2), Mètre ruban ou bracelet PB (min 2), Grille ou table poids/taille (min 3), IMC pour les enfants de plus de 5 ans (min 3), Culottes (min 4) ou bassines en plastique.
Les indicateurs qualité relatifs à la disponibilité et fonctionnalité du matériel sont les suivants. Pour le suivi de la croissance : Toise, Balance, Registre de surveillance infantile, Fiches des enfants, Carnets de la mère et de l'enfant vierges ; pour le SSN : Toise, MUAC, Balance, Critères d'admission et de sortie (affichés), Registre SSN, Protocole de PEC de la malnutrition aigue au Burundi, Tables poids pour taille ; pour le STA : Toise, MUAC, Balance, Critères d'admission et de sortie (affichés), Registre STA, Protocole de PEC de la malnutrition aigue au Burundi, Tables poids pour taille.

Intrants nutritionnels

Il n'y a pas de doute par contre que l'indisponibilité des intrants nutritionnels ait été un vrai handicap pour l'efficacité de l'intervention. Les ruptures de stock ont affecté tant les CDS intervention que les CDS contrôle (avec une séquence différente). A nouveau, le FBP Nutrition n'a pas été en mesure à lui seul à renverser cette contrainte externe : nos résultats montrent ainsi qu'il n'en a pas influencé significativement la disponibilité.

En effet, 83 (92%) CDS (dans les deux groupes) ont encore connu les trois mois précédant l'enquête de 2017 une rupture des intrants du traitement de la MAM ; on a en fait assisté à une légère augmentation de rupture de stock dans le groupe de contrôle (+12 points de pourcentage entre 2014 et 2017) et une légère diminution dans le groupe de traitement (-5 points, cf. Tableau 8, page 40).

Informés relativement tôt du désengagement du PAM au niveau des intrants, nous avions espéré que le Ministère de la Santé et les CDS trouveraient une solution par eux-mêmes. Cela a été malheureusement plus compliqué qu'attendu. La possibilité d'achat au niveau local n'a été accordée que 12 mois après le début de l'intervention – vu le processus d'accréditation requis pour le choix d'un fournisseur. Il y a eu ensuite une suspension provisoire due à des problèmes de qualité. On peut supposer que ces contraintes majeures aient affecté la pertinence du FBP nutrition et de plusieurs de ses indicateurs et démotivé les CDS d'intervention.

En ce qui est des CDS contrôle, on pourrait se poser la question si c'était leur choix délibéré de ne pas se fournir ou bien si c'était du au niveau d'information sur la possibilité d'acheter les intrants dont ils disposaient.

Pour la MAS, la disponibilité des intrants s'est améliorée dans les deux groupes (+20%pts dans le groupe de contrôle et +7%pts dans le groupe d'intervention, cf. Tableau 8, page 40). On observe notamment moins de ruptures de stock dans les trois mois qui ont précédé l'enquête. A nouveau, on n'observe cependant pas d'effet significatif de l'intervention.

Notre étude qualitative (non développée dans ce rapport) soulève aussi la présence de faiblesses structurelles dans le circuit d'approvisionnement en intrants et le fait que les agents de santé n'ont d'autre marge de manœuvre que d'avertir le BDS en cas de menace de rupture de stock. Cette situation s'est fait le plus sentir en ce qui est de l'approvisionnement en farine : *« …nous avertissions à temps mais la livraison prenait trop de temps à nous arriver et nous ne faisions qu'attendre… »*. Pourtant, la stratégie d'achat local d'intrants est jugée efficace pour palier ces ruptures de stocks fréquentes (dans la mesure bien évidemment où la question d'irrégularités de paiement est résolue) : *« …la possibilité d'achat local d'intrants serait une mesure efficace si le paiement se fait à temps parce que nous pouvons alors faire notre propre commande à temps en nous basant sur notre consommation moyenne mensuelle… »*. Cette stratégie présente par ailleurs un avantage de prise de conscience sur la bonne gestion : *« …si nous achetons nous-mêmes, chacun s'assure de la bonne gestion car c'est sur le dos des ressources du CDS… »*.

Ressources humaines

L'autre condition nécessaire pour l'efficacité d'un programme nutritionnel est bien sûr la disponibilité d'un personnel compétent. Ces ressources humaines doivent en outre disposer des savoirs, savoir-faires et pratiques indispensables dans la prévention, le dépistage et la prise en charge de la malnutrition. Notre EI a montré qu'aujourd'hui au Burundi, les ressources humaines sont probablement la plus grosse contrainte aujourd'hui pour l'efficacité de toute intervention nutritionelle au niveau du système de santé.

Le tableau était déjà très sombre en 2014. Ce n'était pas l'objectif du FBP nutrition, et on ne peut donc lui reprocher de ne pas l'avoir atteint, mais le FBP n'a pas changé grand-chose à cette réalité. A certains égards, le tableau est encore plus sombre en 2017.

S'agissant du respect des normes sanitaires en termes de nombre et de qualifications, seuls 9% des CDS du groupe intervention respectent ces normes en 2017 (contre 20% en 2014) ; dans le groupe de contrôle on est passé de 24% en 2014 à 22% en 2017 (cf. Tableau 16, page 47).

Comme déjà en 2014, les pratiques en consultation curatives sont loin de permettre une prévention et un dépistage systématiques de la malnutrition à chaque contact avec un enfant (cf. à partir de page 40).

Les résultats de l'EI ne montrent pas d'impact sur les différences absolues trouvées dans les mesures de poids, taille et PB entre les AS et les enquêteurs, ce qui suggère qu'il n'y a pas eu d'amélioration dans la pratique de prise des mesures anthropométriques (cf. Tableau 12 page 43). Les indicateurs qualitatifs du FBP n'ont pas été en mesure de changer cet aspect.

Sur le volet « conseils nutritionnels en consultation », en 2017, ils ne sont faits que dans 24% des cas dans le groupe de contrôle (contre 21% en 2014) et 19% des cas dans le groupe d'intervention (contre 30% en 2014) ; curieusement, on trouve donc un impact négatif significatif du FBP Nutrition de -12.6%pts (p=0.06). S'agissant des conseils d'hygiène, ils ne sont donnés que dans 8% et 10% des cas respectivement dans le groupe de contrôle et le groupe d'intervention : c'est là aussi moins bien qu'en 2014, et ce dans les deux groupes (cf. Tableau 11 page 42).

Ces résultats interpellent clairement le Ministère de la Santé et ses partenaires bien au-delà du FBP. Mais le programme FBP doit aussi se remettre en question, car ce sont aussi les limites de l'approche qui sont révélées. Il n'est pas exclu ainsi que le résultat négatif repris plus haut soit bien le résultat du FBP lui-même.

A nouveau, il est intéressant de comparer les chiffres produits par l'EI et ceux des données de routine sur le score qualité (cf. Tableau 60, annexe). On observe ainsi que sur l'indicateur « Pour chaque session individuelle de suivi et promotion de la croissance, conseils appropriés délivrés[20] », la moyenne est de 23.2/30 et il y a 206 occurrences sur 285 qui ont eu le score maximal. Cela suggère qu'en séance de suivi et promotion de la croissance, des conseils nutritionnels et d'hygiène sont délivrés. Pourquoi alors n'est-ce pas répliqué en consultation curative ? Une hypothèse serait que le personnel, débordé par le volume

[20] Il y a néanmoins lieu de se demander comment cette évaluation est réalisée.

de travail, tiraillé entre les nombreuses tâches et les rémunérations diverses pratiquées par le FBP ait décidé de sacrifier les conseils nutritionnels en consultation curative. Selon les premiers résultats du travail qualitatif, ces conseils nutritionnels sont réputés en effet être des consommateurs énormes de temps. Ils sont donc donnés en groupe surtout aux enfants déjà admis dans les services SSN et STA le jour de la distribution des intrants. On devine que l'on touche ici du doigt le caractère non globalisé des soins individuels dans les CDS au Burundi et probablement dans la majorité des pays africains.

L'EI montre aussi que le FBP Nutrition n'a pas non plus eu d'impact ni sur la formation du personnel existant ou sur la supervision des prestataires (cf. Tableau 17 page 48 et Tableau 19 page 49). A nouveau, ce n'était pas l'objectif assigné au FBP, mais on aurait pu espérer que les CDS du groupe d'intervention prennent leur propre sort en main, pour améliorer leur performance.

Différentes hypothèses peuvent être avancées. La pratique historique veut que les formations soient organisées et dispensées à l'initiative du PRONIANUT. Une certaine passivité est de mise. Ce qui est une erreur car le PRONIANUT programme ses formations en fonction des mises à jour des protocoles.

Le contexte politique très difficile (et notamment la difficulté de circuler dans le pays) peut aussi avoir découragé les CDS les plus ambitieux et entreprenants. La dégradation de la situation économique et les problèmes au niveau des finances publiques ont peut-être aussi suggéré aux CDS que ce n'était pas le bon moment pour investir dans la formation : il était peut-être plus sage de mettre de l'argent de côté pour être capable d'aborder des chocs à venir (ex. nécessité d'acheter les médicaments, payer des salaires). Peut-être aussi que le sens d'initiative des CDS est aujourd'hui surestimé par le programme FBP. Peut-être que des messages plus clairs doivent être formulés sur l'importance d'être proactif, d'investir dans les compétences.

Notre EI pose aussi la question de la juste cible des formations. En effet, au vu des performances des agents observés en consultation et avec les vignettes, et compte tenu que le PRONIANUT effectue chaque année des formations/recyclages des agents de santé, il y a lieu de se poser la question si ce ne sont pas les mêmes personnes qui sont formées, en l'occurrence les titulaires des CDS et responsables des services nutrition au détriment des personnes fréquemment en contact avec les enfants en consultation curative.

Elle pose aussi la question de l'intensité des supervisions. Pourquoi les services nutritionnels sont si peu supervisés ? A nouveau différentes hypothèses peuvent être mises en avant. Peut-être les compétences des équipes cadre district ne sont pas suffisantes, dans ce domaine qui a longtemps été considéré comme relevant des seuls PTFs et ONGs ? Pourtant il existe actuellement un point focal Nutrition au niveau des districts. Son travail est de s'assurer d'une bonne offre de soins nutritionnels au niveau des formations sanaitaires du district. Peut-être le problème est au niveau du caractère censé intégré des supervisions par l'équipe cadre district ?

Nous avons creusé ces questions avec notre travail qualitatif (non développé dans ce rapport). Il semble que la faible intensité des supervisions est due à deux contraintes: (1) l'absence parmi les superviseurs d'une personne qui dispose d'une solide expérience des services nutritionnels et (2) le fait que les rares formations ciblent le point focal ; ainsi les autres superviseurs ne peuvent jamais se hasarder pour une

supervision : « …*quand il y a une formation, ce n'est que le point focal qui y participe des fois. Comment ce seul superviseur peut assurer les supervisions de tous les services nutritionnels en plus de celles des autres services ?...* . »A nouveau, on touche là des questions qui dépasse le seul FBP.

Un résultat positif de notre EI au niveau des supervisions est la bonne collaboration entre le CDS et le niveau communautaire en ce qui concerne l'encadrement des FARN et démonstrations culinaires qui a été infuencé positivement (+50.4 points de pourcentage, p=0.000, cf. Tableau 18 page 49). La question pendante est pourquoi par ailleurs on n'a pas vu d'impact sur l'encadrement du dépistage ? Quel est l'intérêt relatif du CDS à accompagner les ASC dans des activités de FARN/démonstration culinaire plutôt que dans des dépistages systématiques ? Est ce pour une prise en charge en amont en vue de réduire la durée moyenne de séjour ? Est–ce l'influence d'une ONG qui opérait dans les localités et appuyait les FARN ? Nos premiers résultats qualitatifs semblent indiquer le rôle central des ONG pour l'encadrement des FARN ; les informations seraient par ailleurs davantage disponibles dans le groupe d'intervention du fait de la meilleure tenue des registres pour les ASC (et le FBP Nutrition au niveau communautaire).

Information Sanitaire

Pour de nombreux observateurs, un des effets systémiques du FBP est d'améliorer la qualité de l'information sanitaire. Ceci n'est pas secondaire : une documentation correcte et régulière des activités de dépistage et prise en charge de la malnutrition est indispensable pour une bonne lutte contre la malnutrition.

Notre EI confirme cet effet systémique. Elle montre un impact positif et significatif sur la bonne tenue des registres des deux services de prise en charge de la malnutrition, ainsi que sur le calcul systématique des indicateurs de performance du SSN et STA (cf. Tableau 20, page 51).

Toutefois, on ne trouve pas d'influence du FBP Nutrition sur le bon remplissage des fiches cliniques SSN et STA (sauf taille et PB à l'entrée), qui était pourtant rapporté déjà comme très mauvais en 2014 (cf. Tableau 20 à Tableau 22, à partir de page 51. Or ces fiches cliniques sont d'importants outils pour le bon suivi des enfants. On peut se poser la question du pourquoi de cette disparité d'influence sur les registres et les fiches cliniques. Serait-ce du au fait que les registres sont vérifiés mensuellement, ou qu'ils font partie de la grille FBP qualité ? Quelle a été la place des fiches cliniques dans le système de vérification et d'évaluation qualitative ?

Notre travail qualitatif nous a permis d'identifier deux contraintes majeures dans le remplissage des fiches cliniques : d'une part, le nombre d'enfants et les items à compléter, d'autre part, l'insuffisance du personnel préposé à l'accueil le jour de la distribution des intrants. En effet : « …*le jour la distribution tous les enfants de la file active se présentent et généralement il y a un seul prestataire pour la prise des mesures anthropométriques et le rapportage sur les fiches ainsi que la distribution en soi. Si par hasard c'est un prestataire non formé sur les outils, vous comprenez que le remplissage ne sera pas adéquat… ».* À nouveau, nous semblons donc nous cogner contre l'insuffisance du personnel et les faibles qualifications, mais aussi l'insuffisante compréhension par le personnel du caractère central de certains outils pour le juste diagnostic et une bonne prise en charge individuelle.

5.3 Output

Les gros problèmes rencontrés au niveau de l'approvisionnement en intrants durant l'intervention et le les faiblesses structurelles au niveau des ressources humaines ont fortement pesé sur l'efficacité de l'intervention.

Suivi de la croissance

Les résultats de l'EI ne montrent pas d'influence du FBP Nutrition sur les activités de suivi de la croissance que ça soit selon les informations issues du questionnaire N, des consultations curatives ou des ménages (cf. Tableau 24 page 58). Cette activité reposant sur les courbes de croissance apparaissait déjà comme marginale dans le fonctionnement quotidien des CDS au Burundi en 2014. Le FBP nutrition n'a pas renversé la situation et cette activité pourtant primordiale pour la promotion de la bonne croissance et pour le dépistage de cas de malnutrition continue à recevoir trop peu d'attention.

Pourtant, le système de primes du FBP Nutrition contient des indicateurs quantité et qualité sur cette activité[21] ; ceux-ci requièrent le dessin des courbes poids pour âge. Selon les données de routine du FBP Nutrition, au total, environ 200 000 cas de suivi de la croissance ont été validé dans les 45 CDS du groupe d'intervention sur 2015 et 2016. Cela correspond à une intensité de suivi supérieure à celle que nous documentons avec notre enquête. Ceci pose la question de comment ces activités ont été vérifiées et validées par le système FBP. Est-ce que les vérificateurs analysent effectivement les courbes de poids pour âge des cas ? Un examen attentif des données de routine qualité (cf. Tableau 60, annexe) montre en fait que la moyenne de l'indicateur « les courbes de croissance P/A et T/A sont bien tracées » est de 3.4/10 et que 96 cas[22] sur 285 ont eu le score maximal sur toute la période de l'intervention (2015 et 2016). Ceci suggère que le problème est identifié ; toutefois, notre évaluation est encore plus critique. Ceci suggère une plus grande fermeté au niveau de la vérification (voir nos recommandations).

Détection de la malnutrition

À juste titre, le Burundi a fait le choix de la prise en charge intégrée des maladies de l'enfance. Chaque opportunité doit être saisie pour identifier les enfants en danger. Nos résultats sont décevants à ce niveau : nous n'avons pas détecté d'influence du FBP Nutrition sur la détection de la malnutrition lors de la consultation curative : selon notre étude observationnelle, sur 529 patients, seuls 18 ont été diagnostiqués comme souffrant de malnutrition aigue en consultation alors que selon les données anthropométriques, 70 enfants étaient malnutris aigus. Curieusement, on a en fait observé une influence significative du FBP Nutrition sur le taux de faux négatifs (+12%pts, p=0.027, cf. Tableau 25 page 59).

Cette déception – observée sur un petit échantillon – semble être compensée par un résultat positif à partir de l'analyse des fiches cliniques SSN et STA. À ce niveau, nous avons identifié une influence significative du FBP Nutrition sur le nombre d'enfants traités pour (+138 cas en moyenne par mois et par

---

[21] L'indicateur quantité correspond au nombre d'enfants de moins de 2 ans dont le poids et la taille ont été mesurés au cours du mois et dont la courbe de croissance poids / âge suit une bonne trajectoire de croissance ; il doit être vérifié par l'analyse des courbes poids pour âge.

[22] Le nombre d'observations correspond au nombre de CDS multiplié par le nombre de trimestres pour lesquels on a des données sur le score qualité.

CDS sur six mois, p=0.000) et celui d'enfants traités pour MAS (+53 cas sur six mois, p=0.000, cf. Tableau 27 page 61). Ce résultat est confirmé avec les données ménages : à la baseline, environ 8.5% des enfants identifiés comme souffrant de malnutrition aigue par les mesures des enqueteurs étaient pris en charge en SSN ou STA, de façon équivalente dans les deux groupes ; ce taux est passé à 13.8% dans le groupe de contrôle et à 17.8% dans le groupe d'intervention, ce qui suggère que dans le groupe d'intervention les agents de santé identifient et prennent en charge davantage de cas. L'augmentation des cas observée au niveau des centres de santé n'est donc pas le résultat d'un surcodage ou de fraudes de la part des agents de santé, mais bien une réalité. Par contre, le taux faible confirme que le FBP est loin d'avoir réglé le problème (cf. Tableau 30 page 63).

Comment pouvons-nous expliquer ces divergences de résultats entre les différents instruments ? C'est difficile de trancher. La provenance des cas est rarement indiquée sur les fiches SSN (moins de 10% des cas en 2017) ; pour ce dont on dispose, on voit que la majorité des cas proviennent soit du dépistage dit « spontané » soit des transferts provenant du STA. En ce qui concerne les fiches STA, l'information est davantage disponible, et on voit que la grande majorité des cas proviennent soit du dépistage dit « spontané » soit du dépistage dit « actif »[23]. Il y a probablement un lien avec l'ampleur des activités des FARN et démonstrations culinaires sous l'encadrement des CDS (qui est plus importante dans le groupe d'intervention que dans le groupe de contrôle), mais cela ne semble pas suffisant comme explication. On peut supposer que le dépistage est plus systématiquement réalisé lors des consultations préventives, mais pourquoi alors ne pas maintenir cette qualité en consultation curative ? Par manque de temps ? De façon générale, il est clair qu'il y a un gros problème de qualité dans l'activité curative pédiatrique dans les CDS au Burundi. La pression du volume pourrait condamner toute réelle amélioration de la prise en charge.

Les premiers résultats de notre recherche qualitative confirment que le dépistage en consultation curative n'est pas une préoccupation du personnel, la faute aux files d'attente et au nombre insuffisant de prestataires : *« …les canaux de dépistage de la malnutrition sont de deux ordres : en consultation préventive lors des vaccinations ainsi que les références des ASC qui sont aussi contractualisés…».* Peut-être devons-nous acter ce choix opérationnel fait par des prestataires sous pression et peu disponibles. Une meilleure intégration au niveau communautaire serait alors en effet une piste d'action. Les résultats de l'étude qualitative au Rwanda avaient déjà également identifié l'implication des ASC comme la clé du succès du programme, notamment en créant une demande de services au niveau des communautés (Kandpal 2016).

Notre EI montre aussi que les critères d'entrée dans le SSN restent peu respectés, et il ne semble pas y avoir eu d'influence de l'intervention là-dessus. C'est cohérent avec ce que l'on voit dans les données de routine issues des évaluations qualité, cf. Tableau 60, annexe : la moyenne du score relatif au respect des critères d'entrée en SSN est de 5.2/10 sur la période 2015-2016 ; cela étant, on observe une amélioration de cette moyenne, elle est passée de 3.0/10 en 2015 à 7.7/10 en 2016, ce qui suggèrerait que le FBP Nutrition apporte une amélioration en ce sens, même si elle n'est pas détectable avec les

---

[23] Un cas dit « spontané » est un cas arrivé de soi au CDS, tandis qu'un dépistage dit « actif » suggère que les agents de santé se sont déplacés vers les communautés pour le dépistage.

données d'enquête réalisées début 2017. Pour ce qui est des paramètres d'entrée en STA, ils étaient et restent corrects dans une grande majorité des cas, ce qui est conforme au score qualité pratiqué par le FBP nutrition (cf. Tableau 60, annexe).

Prise en charge des cas de MAM et de MAS

D'après l'analyse des données d'enquête, il semble que le FBP Nutrition n'ait pas entrainé d'amélioration en terme de traitement systématique ou diététique (cf. Tableau 28 et Tableau 29 page 62). Dans le STA, on observe une chute du traitement systématique d'amoxicilline et d'al/mebendazole dans les deux groupes, due essentiellement à une chute du traitement d'al/mebendazole (peut-être un autre problème d'approvisionnement ?). Néanmoins, on doit faire attention au fait que l'information sur le traitement systématique a été collectée d'une manière différente dans les deux enquêtes ce qui rend les comparaisons non pertinentes.

Cela étant, d'après les données de routine d'évaluation qualité, on peut juger de la qualité de la prise en charge dans le groupe d'intervention (cf. Tableau 60, annexe). Les scores sont en moyenne plutôt faibles en ce qui concerne la prise en charge dans le SSN (9.3/20), mais ont fort augmenté en passant de 3.9/20 en 2015 à 15.1/20 en 2016 ce qui suggère que le FBP Nutrition a peut-être eu une influence positive sur la prise en charge, même si cela n'est pas détectable au niveau des données d'enquête. Quant au STA, les scores sont relativement élevés et stables (25.0/30).

5.4 Résultat clinique de la prise en charge

Une des variables-clés pour apprécier l'efficacité de l'intervention FBP Nutrition est bien sûr le taux de guérison au niveau des deux services. On a là un des rares résultats positifs de cette EI – mais c'est l'un des plus importants.

Nous avons vu (Tableau 32 page 65) que le taux de guérison de MAM est passé de 84% à 97% dans le groupe d'intervention (versus 76% à 78% dans le groupe de contrôle). Cela donne un impact significatif sur le taux de guérison en SSN de +14.7%pts. Ce taux est potentiellement sous-estimé du fait que très peu de fiches ont été collectées côté groupe de contrôle : on peut en effet penser que dans le groupe de contrôle on a en effet gardé les FOSA les plus sérieuses dans le domaine de la nutrition et/ou celles aidées par des ONGs tierces (la majorité des fiches SSN retranscrites dans le groupe de contrôle viennent de Rutana (60) et Ruyigi (24), provinces aidées par le PAM). Par ailleurs, la durée de traitement chez les guéris a également été significativement réduite dans le groupe de traitement (-29 jours), ce qui satisfait les indicateurs de performance (évalués dans la grille qualitative).

En ce qui en est du STA, nous avons vu que l'intervention n'a pas eu d'impact significatif sur le taux de guérison ; par contre, on trouve un impact significatif du FBP Nutrition sur le respect des critères de sortie (+ 20%pts). Comme avec le SSN, la durée de traitement chez les guéris a également été significativement réduite dans le groupe de traitement (-20 jours ; cf. Tableau 33 page 67).

Element rassurant : les paramètres rapportés à la sortie du SSN et STA permettent de vérifier que les cas rapportés comme « guéris » le sont effectivement, et ce dans les deux groupes ; ce qui soutient l'observation de cette augmentation des taux de guérison.

On observe donc une amélioration générale des critères de performance des deux services SSN et STA. Ceci est potentiellement lié aux évaluations qualité du FBP Nutrition. En effet, le respect des critères de performance compte pour 60 points sur un total maximum de 330 points (ce qui n'est pas négligeable lorsqu'on souhaite atteindre un score supérieur à 80% pour bénéficier d'un bonus qualité, ou au moins supérieur à 60% pour ne pas avoir de malus). Aussi, si on regarde les moyennes annuelles, on observe une amélioration de ces sous-scores de performance dans le SSN (de 12.8/30 à 18.2/30) et dans le STA (de 3.0/30 à 16.4/30 ; cf. Tableau 60, annexe).

Ce résultat pose bien sûr une question centrale sur la théorie de changement de toute l'intervention : comment ces indicateurs de performance se sont-ils améliorés, dans un contexte où on n'a mesuré d'amélioration ni dans l'équipement, ni dans la disponibilité des intrants, ni dans la supervision, ni dans le bon remplissage des fiches cliniques individuelles, ni dans les conseils nutritionnels ? L'hypothèse est que les CDS en savent plus que nous sur comment améliorer la santé des enfants malnutris. Il n'est pas exclu que les FARN y soient pour quelquechose.

Les premières informations collectées de façon qualitative suggèrent que les CDS se sont concentrés beaucoup plus sur les enfants référés par les ASC. Il semble ainsi que dans de nombreux CDS, deux journées par mois sont dédiées au dépistage et à la première prise en charge des dépistés ; ces journées correspondent aux jours où les enfants référés par les ASC doivent se rendre au CDS.

5.5 Limites de l'étude

Cette évaluation d'impact a suivi un design d'essai randomisé avec groupe de contrôle. De par sa conception, les résultats issus de cette recherche sont robustes. Cela étant, les résultats issus des retranscriptions de fiches SSN sont à prendre avec précaution étant donné le biais de sélection : beaucoup plus de fiches ont pu être retranscrites côté intervention par rapport à côté contrôle ; en effet le groupe contrôle a en grande majorité fermé le service en raison du manque d'intrants diététiques ; les seuls qui ont gardé le service ouvert sont ceux qui étaient aidés par d'autres organisations ou pour qui la nutrition est une activité prioritaire. Notre analyse est que ce biais a engendré une sous-estimation de l'effet de l'intervention.

Les données d'enquête auprès des CDS et des ménages sont riches et permettent de creuser différents aspects liés à la situation de la nutrition au Burundi ainsi qu'à l'efficacité de l'intervention. A travers des méthodes d'analyse économétrique de double différence, ces données ont notamment permis de montrer une amélioration de la performance des services de prise en charge de la malnutrition aigue. Cela étant, les raisons de cette amélioration restent obscures : les données dont nous disposons permettent d'affirmer que ce n'est pas grâce à des améliorations dans l'équipement, dans la disponibilité des traitements, dans la supervision, dans la qualité des consultations curatives au regard de la nutrition, ni dans le remplissage des fiches cliniques individuelles. Aussi, des études complémentaires restent à réaliser. Un des experts de cette étude (Manassé Nimpagaritse) étudiera certaines des questions qui demeurent dans les prochains mois, avec des méthodes qualitatives.

6. Recommandations

Cette section décrit les recommandations politiques issues de cette étude. Le tableau 64 en annexe fournit une synthèse de ces recommandations sous forme de plan d'action.

6.1 A l'égard du MSPLS pour la mise à l'échelle du FBP Nutrition

Au niveau de la CT-FBP et du PRONIANUT

Opter pour une approche globale de la malnutrition

Selon nous, les résultats de l'EI indiquent qu'au Burundi, il sera difficile d'obtenir des progrès notables au niveau de la malnutrition des enfants, avec le seul FBP Nutrition, même après révision (voir autres recommandations ci-dessous). Il faut une approche globale. Le FBP peut faire partie de cette approche, notamment pour aider les CDS à faire attention à ce qui importe, mais le FBP seul ne suffira. Il est clair aussi qu'il faut s'attaquer à certains déterminants hors de contrôle des AS. Certains de ces déterminants sont au niveau du système de santé (ex. fourniture en intrants), d'autres (éducation, pauvreté…) vont bien au-delà.

Engager une réflexion critique sur le FBP

Selon nous, les résultats de l'EI posent aussi des questions quant à la théorie de changement du FBP en général. Une priorité est de favoriser une pensée plurielle sur les déterminants de la qualité des soins. Le décalage entre les scores des indicateurs qualité du programme FBP et certaines réalités documentées par cette étude (mauvaise prise en charge intégrée des maladies de l'enfance, faibles connaissances du personnel, faible usage d'outils de dépistage et de traitement…) indique que le problème de qualité des soins au niveau des CDS a peut-être été sous-estimé.

Nous recommandons que le MSPLS travaille sur une stratégie globale sur le défi de la qualité des soins au niveau des CDS. Le FBP doit faire partie de cette stratégie (éventuellement avec des innovations comme une mesure des compétences sur base de vignettes électroniques), mais doit être complété par d'autres stratégies. D'une part, parce que certaines dimensions cruciales de la qualité des soins sont mal capturées par le système FBP, d'autre part, parce que ce dernier n'éveille pas suffisamment les AS sur les gros défis de la qualité des soins. Sans une action profonde sur cette dernière, il est à craindre que les augmentations en volume suscitées par le FBP ne se convertissent pas en gains sanitaires pour les populations.

Au niveau des CDS

Optimiser les ressources humaines

Afin que le FBP Nutrition (et plus généralement le FBP classique) soit efficace, il est indispensable que la contrainte des ressources humaines soit pleinement reconnue et attaquée de front par le MSPLS : elles doivent être en nombre suffisant et compétentes. Comme déjà évoqué dans le rapport de l'étude de référence, il y a un gros défi au Burundi, notamment au niveau de la prise en charge intégrée des maladies de l'enfance. Pour générer un vrai bénéfice pour la population, la formation continue et la

supervision sont à améliorer (cf. recommandations générales à l'égard du MSPLS ci-dessous). Des enjeux existent sans doute aussi au niveau de la formation initiale.

Améliorer la vérification

De nombreux cas d'activités de suivi de la croissance ont été validés (env. 200,000 sur les deux années), alors même que le critère de vérification (courbes de croissance ascendantes) ne sont que très rarement disponibles. Comment ont-été effectuées ces vérifications ? Est-ce que les critères auraient-été revus ? La question reste à creuser avec les CPVV mais il s'agit là d'un point qui doit être traité. Le FBP ne peut induire un effet bénéfique pour les usagers que si ce qui compte est effectivement presté.

Par ailleurs, vérifier ne devrait pas revenir simplement à vérifier pour valider un paiement ; il s'agit aussi de supervision et de conseils pour amélioration : il devrait y avoir des feedbacks de la part des vérificateurs. Qu'en est-il de ces recommandations faites aux CDS ? Est-ce possible de les améliorer ?

Revoir les indicateurs

Il semble qu'il y ait certaines redondances dans les indicateurs. Notamment, l'indicateur quantité « suivi de la croissance » ne devrait pas être complété par un indicateur qualité « existence des courbes de croissance » puisqu'il doit être validé justement à partir de courbes de croissance. De même, parmi les indicateurs de qualité, les indicateurs de prise en charge correcte et les critères d'admission corrects sont redondants.

Par ailleurs, les points attribués dans la grille qualité devraient être revus régulièrement, de manière à améliorer graduellement la qualité. En effet, d'après les données de routine, les scores ont tendance à s'améliorer avec le temps. Aussi, on devrait profiter de cette tendance pour progresser vers la qualité optimale. Dans un premier temps, il s'agirait uniquement de changer le mode d'attribution des points. Par exemple, pour le critère de « disponibilité de l'équipement », au lieu de retirer 5 points pour chaque outil manquant, on pourrait donner la totalité des points seulement quand tout l'équipement est là, et ne donner aucun point dans le cas contraire.

Au niveau des GASC

De la même manière, pour que le FBP communautaire soit efficace, il faudrait , selon la recherche qualitative menée auprès de 42 GASC et 42 professionnels de santé responsables[24] :

- Assurer une formation soutenue des ASC en matière de dépistage de la malnutrition aigüe, de l'art culinaire et du processus de rapportage ;
- Au niveau des CDS, recruter des TPS chargés de la supervision-formation régulière des ASC ;
- Organiser des séances d'explication du projet aux autorités administratives locales ainsi qu'aux leaders communautaires pour une meilleure compréhension des objectifs et une meilleure adhésion au projet, et ainsi réduire le sentiment de jalousie que soulève la rémunération des ASC ;
- Prévoir du matériel pédagogique comme des images illustratives ou des documents de référence pour les ASC en rapport avec les activités de ce projet ;
- Eviter les retards de payement.

[24] Cf. étude de Nkurunziza et al. 2018 (soumise à publication ; disponible sur demande en attendant).

6.2 A l'égard du MSPLS de manière générale

Au vu des résultats de l'étude et dans une perspective de passage à l'échelle, un certain nombre de problèmes à résoudre et recommandations sont à envisager, tant pour la réussite du passage à l'échelle du FBP Nutrition en particulier, que du FBP classique et des éventuelles interventions futures en nutrition.

Améliorer la performance des agents de santé

1. Optimiser les formations des AS

Cette EI a révélé le problème criant de connaissances des agents de santé, notamment en matière de nutrition. Et ce, malgré la réalisation récente à travers le pays de formations faisant suite au nouveau protocole de prise en charge de la malnutrition (datant de fin 2014). Ceci soulève la question de la valeur ajoutée des différents recyclages et formations régulièrement organisées par les divers programmes du MSPLS. Sommes-nous sûrs de leur efficacité ? Il est indispensable de mener une évaluation pour comprendre l'efficacité de ces formations, identifier les barrières à leur efficacité, en vue de nous assurer qu'elles correspondent aux besoins réels, adoptent bien une méthodologie appropriée et touchent les AS susceptibles de générer un bénéfice pour les populations.

2. Assurer la disponibilité et l'application des protocoles

Une des causes de la faible qualité des soins offerts aux enfants du Burundi est la non adhérence aux protocoles de soins en vigueur. Cette non-adhérence peut avoir de nombreuses sources. Selon une récente revue (Rowe et al 2005) une barrière peut être la non disponibilité des protocoles au niveau des centres de santé. Mais il nous faut certainement réfléchir aux autres déterminants de non-suivi et identifier comment le système pourrait s'attaquer à ces déterminants, via des supervisions, les indicateurs qualité du FBP ou même peut-être à terme des solutions via tablettes digitales.

Cependant, même dans les CDS (de l'étude) où ces protocoles étaient disponibles dans leurs formes physiques, ils n'étaient pas pour autant suivis.

Le MSPLS doit rester conscient que l'utilisation systématique des protocoles est lourde pour les centres de santé compte tenu de l'affluence des patients par rapport aux ressources humaines disponibles. Il doit ainsi s'assurer d'approcher le respect des normes en personnels et en aires de responsabilité des centres de santé.

4. Améliorer l'efficacité de la supervision

Les résultats de l'étude remettent en cause également la supervision des centres de santé et plus particulièrement du service nutritionnel. Or, de récentes études ont montré que la supervision et l'audit peuvent avoir un impact important sur l'amélioration de la performance des AS (Rowe et al 2005; Grimshaw et al.2004) ; plus que la formation seule en fait.

Nous recommandons que la formation des superviseurs (à différents niveaux) soit améliorée pour s'assurer que leurs visites de supervision soient efficaces et productives. Concrètement, il s'agit pour les

superviseurs de prévoir suffisamment de temps dans chaque centre et d'avoir déjà une connaissance de base sur les besoins des AS. Cela semble simple mais c'est souvent négligé (Rowe et al. 2005). Ainsi, plutôt que de se contenter de passer en revue les registres, les superviseurs pourraient prendre le temps de discuter avec les AS des gestes posés sur les patients. Pour cela, un renforcement des compétences des superviseurs sur la prise en charge (anamnèse, examens, diagnostic, traitement et conseils) semble également nécessaire.

5. Créer des Centres d'Excellence et organisation de stages

En plus de formations et de supervisions améliorées sur les soins aux enfants, il est suggéré de mettre en place un système où les AS iraient faire un «stage» dans une FOSA, identifiée comme ayant un grand nombre de cas spécifiques et ayant un personnel expérimenté dans de tels soins, afin qu'ils puissent apprendre de manière pratique. Ces centres pourraient recevoir un soutien externe pour devenir, par exemple, le centre d'excellence pour la gestion de la malnutrition dans un contexte de soins de santé primaires

En particulier, cela serait utile pour les AS travaillant dans des centres de santé qui, à tort ou à raison, n'identifient pas beaucoup de cas de malnutrition aigüe. Aller dans des centres de santé qui connaissent une plus grande incidence de malnutrition aigüe, et donc sont plus spécialisés les aideraient à apprendre davantage sur le dépistage et la prise en charge de la malnutrition aigüe. Aussi, il serait bénéfique pour les AS de faire des stages sur une base régulière dans des services de stabilisation (SST) afin de devenir plus familiers avec la prise en charge des complications à l'hôpital, et surtout avec l'identification des complications au niveau du CDS. Cela pourrait aussi aider dans la communication avec les parents, d'autant plus qu'ils pourraient leur expliquer, avant de les référer vers le SST, quels soins ils vont recevoir comparativement à ceux dispensés par le centre. Il se peut que des AS aient fait des rotations dans ces services, mais si cette pratique n'est pas régulière, elle peut être oubliée et les AS peuvent perdre confiance dans leurs compétences professionnelles.

On pourrait également envisager que des CDS « excellant » dans les consultations curatives en général soient identifiés comme tels, par les superviseurs formés, au sein de chacun des districts, permettant aux AS de chacun des autres CDS du district de se former à moindre coût.

Améliorer le fonctionnement quotidien des services de pédiatrie et de nutrition

Au vu des résultats de l'étude, des efforts pour améliorer le fonctionnement quotidien des services pédiatriques et de la malnutrition semblent essentiels pour l'offre des soins appropriés. Par ailleurs, ils pourraient avoir également un impact positif pour la suite sur la performance des AS (cela doit être vérifié par des recherches qualitatives sur les défis et les besoins des AS). Les recommandations seraient les suivantes.

1. Assurer la disponibilité et la fonctionnalité des équipements

De part les résultats, il paraît évident que les CDS sont restés en manque de nombreuses pièces essentielles de matériel ou de ressources pour évaluer l'état nutritionnel des enfants. Il ressort ainsi que les fonds des CDS, provenant non seulement des allocations FBP mais également d'autres sources, n'ont pas permis l'amélioration de la qualité de l'équipement et l'assurance de sa sécurité et de son entretien

dans les centres. S'il s'avère que c'est un problème de disponibilité sur le marché local ou que l'investissement est lourd au délà des capacités des centres de santé, le MSPLS avec l'appui de ses partenaires, doit garantir dans l'immédiat l'équipement initial de base et initier un fonds propre à chaque CDS qui sera destiné à l'entretien et au renouvellement de l'équipement fourni.

2. Mieux organiser et gérer les données patients dans les services de prise en charge de la malnutrition

Les résultats de l'étude ont montré un impact positif dans la tenue registres des services SSN et STA. Cependant, il subsiste de grosses lacunes dans le remplissage des fiches individuelles de suivi. Cela milite en faveur d'une prise en compte globale des outils de collecte des données dans l'évaluation qualitative trimestrielle en vue d'une amélioration de la gestion des données patient et de l'organisation des services de la malnutrition. Ceci dit, l'objectif aux yeux de l'AS doit être clair : il ne s'agit pas de remplir les fiches pour répondre à la requête bureaucratique du FBP, mais bien pour aider la gestion du cas individuel. La révision de la grille incitative doit aider à cette prise de conscience.

Par ailleurs, le montage doit stimuler l'amélioration du système de stockage des fiches du patient. Aussi, plutôt que d'avoir un seul membre du personnel affecté à cette tâche, il pourrait y avoir une petite équipe qui pourrait maintenir la continuité de la gestion de la documentation, même avec des changements de personnel ou des absences.

D'un autre côté, le MSPLS à chaque niveau doit garantir la disponibilité des fiches vierges, et cela dans tous les centres de santé afin de garantir que les CDS récoltent les mêmes informations.

3. Apporter une solution durable à la problématique de disponibilité des intrants

Les résultats de l'étude montrent clairement que la mise en œuvre de l'intervention a été fortement handicapée par la disponibilité des intrants nutritionnels du SSN. Cela a été principalement dû à la lourdeur des procédures de passation des marchés. L'étude a tout de même montré que la prise en charge de la MAM est possible avec des intrants produits localement. Il reste à en améliorer les conditions de production et les modalités de mise sur le marché. Il serait souhaitable que le MSPLS, avec l'appui de son principal partenaire en la matière (PAM), arrive à une législation qui permette la fortification des aliments sur la place avec des mesures d'accompagnement conséquentes.

La disponibilité des intrants concerne également les intrants diététiques dans le STA et les intrants liés au traitement systématique, à savoir : les antibiotiques, les antipaludéens, les antiparasitaires, le vaccin anti-rougeoleux, l'acide folique et la vitamine A. On a noté des ruptures de stock fréquentes en intrants diététiques (Plumpy Nut) ainsi que des kits de produits de traitement systématique très souvent incomplets. Or il est à nouveau urgent et important de garantir la possibilité continue pour les CDS de s'approvisionner en intrants pour une offre de soins complets.

6.3 A l'égard des commanditaires d'autres études futures

1. Préparation du montage de l'étude et de l'enquête de base

La réalisation de l'étude a connu des contraintes notamment en termes de temps du montage au démarrage effectif de l'intervention. Afin d'éviter d'éventuels retards dans toutes les activités, il faut

prévoir suffisamment de temps et une bonne séquence entre le montage de l'intervention, celui de l'étude et le démarrage effectif des activités. Les choses vont toujours moins vite qu'espéré.

2. S'assurer dès le départ d'une bonne coordination des parties prenantes dans l'étude

L'étude a souffert de la mise en place tardive de la structure de coordination des différents intervenants (ref. le comité de pilotage). Par ailleurs, cette structure, une fois mise en place s'est avérée inopérationnelle, ce qui a limité les ajustements éventuels indispensables de l'intervention. Ainsi, il faut une structure solide de coordination qui s'assure régulièrement de la complémentarité des parties prenantes tout au long de l'intervention. Cette structure doit être constituée non pas sur base des personnes , mais sur base des fonctions qu'elles occupent ; ceci pour garantir la continuité du pilotage de l'intervention.

3. Assurer un suivi rapproché durant la période d'intervention

Il est important d'effectuer un suivi évaluation rapproché de la mise en œuvre de l'intervention, durant toute la période d'exposition, pour permettre d'expliquer par quels mécanismes une intervention a un impact, mais aussi pour pourvoir expliquer les raisons d'un éventuel échec. Ceci doit s'assurer que chaque partie prenante joue pleinement sa fonction et qu'elle anticipe sur le contexte du moment propre à chaque situation en vue des ajustements appropriés.

Ce suivi doit également pouvoir déceler les différents éléments externes pouvant interférer avec l'intervention. Il s'agit notamment des implications des différents partenaires techniques et financiers dans le domaine concerné. Il s'agit également de prendre en compte les implications des différents engagements des bailleurs de fonds dans le budget de la santé mais aussi dans le budget du pays en général ; la situation économique du pays ; etc.

4. Viser la triangulation des données

Il est apparu des zones d'ombre pour certaines informations. Ainsi, les entités de mise en œuvre d'une quelconque intervention doivent penser à des mécanismes de collecte des données qui permettront une certaine triangulation de celles-ci. Il s'agirait de s'assurer que les données de routine sont collectées régulièrement, d'inclure les indicateurs cibles dans la contre-vérification même durant l'intervention, etc.

7. Références bibliographiques

Basenya, Olivier, Manassé Nimpagaritse, François Busogoro, Juvénal Ndayishimiye, Canut Nkunzimana, Gérard Ntahimpereye, Michel Bossuyt, Juma Ndereye, and Léonard Ntakarutimana. 2011. "Le Financement Basé Sur La Performance Comme Stratégie Pour Améliorer La Mise En Oeuvre de La Gratuité Des Soins: Premières Leçons de L'expérience Du Burundi." WP 5. *PBF CoP Working Paper*. PBF CoP Working Paper.

DHS. 2010. "Enquête Démographique et de Santé Burundi 2010."

Hayes, R J, and S Bennett. 1999. "Simple Sample Size Calculation for Cluster-Randomized Trials." *International Journal of Epidemiology* 28 (2): 319–26.

INSP. 2014. "Rapport de La Collecte Des Données Sur Terrain - Enquête de Base Auprès Des Centres de Santé Dans Le Cadre de L' « Evaluation D'impact de L'introduction de La Nutrition Dans Le Programme PBF Au Burundi »." Bujumbura, Burundi.

Kaiser, Reinhard, Bradley A. Woodruff, Oleg Bilukha, Paul B. Spiegel, and Peter Salama. 2006. "Using Design Effects From Previous Cluster Surveys to Guide Sample Size Calculation in Emergency Settings." *Disasters* 30 (2): 199–211.

Kandpal, Eashani. 2016. "Completed Impact Evaluations and Emerging Lessons from the Health Results InnovationTrust Fund Learning Portfolio."

Lechner, Michael. 2011. "The Estimation of Causal Effects by Difference-in-Difference Methods." *Foundations and Trends in Econometrics* 4 (3): 165–224. doi:10.1561/0800000014.

Meessen, Bruno, David Hercot, Mathieu Noirhomme, Valéry Ridde, Abdelmajid Tibouti, Christine Kirunga Tashobya, and Lucy Gilson. 2011. "Removing User Fees in the Health Sector: A Review of Policy Processes in Six Sub-Saharan African Countries." *Health Policy and Planning* 26 Suppl 2 (suppl_2): ii16-29. doi:10.1093/heapol/czr062.

Ministère à la Présidence chargé de la Bonne Gouvernance et du Plan [Burundi], Ministère de la Santé Publique et de la Lutte contre le Sida [Burundi] (MSPLS), ICF International, and Institut de Statistiques et d'Etudes Economiques du Burundi (ISTEEBU). 2018. "Troisième Enquête Démographique et de Santé." Bujumbura, Burundi: ISTEEBU, MSPLS, et ICF.

MSPLS. 2010. "Protocole National de Prise En Charge Intégrée de La Malnutrition Aigüe Globale."

———. 2011. "Rapport de Mise En Œuvre Du FBP et de La Gratuité Des Soins Pour L'année 2011."

———. 2013. "Note Technique Relative À L'intégration de La Nutrition Dans La Stratégie Nationale de Financement Basé Sur La Performance."

Nimpagaritse, Manassé, and Maria Paola Bertone. 2011. "The Sudden Removal of User Fees: The Perspective of a Frontline Manager in Burundi." *Health Policy and Planning* 26 Suppl 2 (suppl_2): ii63-71. doi:10.1093/heapol/czr061.

Nkurunziza, Sandra, Sybil Anthierens, Jean Pierre Van Geertruyden, and Catherine Korachais. 2018. "Understanding Factors That Influence the Implementation of the Performance-Based Financing Scheme at Community Level for Nutrition Services in Burundi." *To Be Published*.

Nkurunziza, Sandra, Bruno Meessen, Jean Pierre Van geertruyden, and Catherine Korachais. 2017. "Determinants of Stunting and Severe Stunting among Burundian Children Aged 6-23 Months: Evidence from a National Cross-Sectional Household Survey, 2014." *BMC Pediatrics* 17 (1). BMC Pediatrics: 1–14. doi:10.1186/s12887-017-0929-2.

Ntakarutimana, Leonard, and Manassé Nimpagaritse. 2013. "Etat Des Lieux de La Prise Charge de La Malnutrition Dans Les Formations Sanitaires Au Burundi : Résultats D'une Enquête Effectuée Dans 24 FOSA Du 20 Au 28 Mai 2013."

Rowe, AK, and et al. 2005. "How Can We Achieve and Maintain High-Quality Performance of Health Workers in Low-Resource Settings?" *Lancet* 366: 1026–35.

UNDP. 2010. "Rapport Burundi 2010 - Objectifs Du Millénaire Pour Le Développement."

UNICEF, WHO, and The World Bank. 2017. "Joint Child Malnutrition Estimates." http://www.who.int/nutgrowthdb/estimates2016/en/.

WFP. 2008. "Comprehensive Food Security and Vulnerability Analysis, Burundi."

WHO. 2010. "Indicators for Assessing Infant and Young Child Feeding Practices. Part 3: Country Profiles." Geneva.

———. 2014. "Integrated Management of Childhood Illness: Chart Booklet."

World Bank. 2015. "World Development Indicators."

8. Annexes

8.1 Instruments de collecte – les vignettes

Tableau 37. Résumé des vignettes cliniques

Vignette	Signes	Diagnostic	Traitement
**n°1**	▪ garcon de 13 mois<br>▪ tachypnéique<br>▪ températeure: 38.6°c<br>▪ pâleur<br>▪ même poids que trois mois avant<br>▪ z-score à -3<br>▪ hémoglobine <9,3g/dl	**MAS compliquée** avec pneumonie, anémie et retard de croissance	▪ antibiothérapie (amoxycilline) pour la pneumonie mais aussi comme traitement systématique<br>▪ antipyrétique (paracétamol) car présence de fièvre<br>▪ acide folique pour anémie et comme traitement systématique<br>▪ albendazole en traitement systématique<br>▪ référer l'enfant au SST<br>▪ compléter la vaccination<br>▪ donner des conseils diététiques et sur l'hygiène<br>▪ expliquer les signes de danger
**n°2**	▪ garcon de 7 mois<br>▪ hémoglobine <9.3g/dl<br>▪ z-score entre >-3 et <-2 MAIS<br>▪ PB =114mm (<115mm)<br>▪ Presence d'œdèmes bilatéraux	**MAS non compliquée** avec anémie et retard de croissance	▪ antibiothérapie (amoxycilline) pour traitement systématique<br>▪ antipyrétique (paracétamol) si fièvre<br>▪ acide folique comme traitement systématique<br>▪ envoyer l'enfant au STA<br>▪ donner des conseils dietétiques et sur l'hygiène
**n°3**	▪ fille de 18 mois<br>▪ temperature: 37,8°c<br>▪ test rapide de paludisme (RDT): positif, sans signes de sévérité<br>▪ z-score entre <-2 et >-3<br>▪ pas d'œdèmes ou d'autres complications médicales<br>▪ même poids que 3 mois avant	**MAM** avec paludisme non compliqué et retard de croissance	▪ ASAQ en traitement oral; pas d'indication pour IV<br>▪ antipyrétique (paracétamol)<br>▪ envoyer l'enfant au SSN<br>▪ donner des conseils diététiques et sur l'hygiène

8.2 Statistiques descriptives clé générales de l'enquête de 2017

Enquête au niveau des centres de santé

Les sous-sections suivantes présentent quelques résultats généraux issus de l'enquête finale. Pour les résultats de l'enquête de référence, prière de se référer au rapport de baseline.

Aspects organisationnels des centres de santé

Questionnaire G– Aspects organisationnels du centre de santé

Le questionnaire a été administré dans la plupart des cas au titulaire du CDS comme prévu, et dans env. 20% des cas, il a été administré au titulaire adjoint.

Organisation et infrastructure

Globalement, 83% des centres de santé sont du secteur public, tandis que 17% relèvent du secteur privé confessionnel. En termes de gestion générale et d'outils de communication, 64% des centres de santé ont un téléphone, et un ordinateur fonctionnel pour 88%. Parmi ces derniers, 72% ont une imprimante fonctionnelle et un accès à internet pour 10%.

Gestion et ressources humaines

92% des répondants ont déclaré disposer d'un comité de santé (appelé « COSA») et 73% d'un comité de gestion (également appelé « COGE »). D'après les normes de santé 2011-2015 du Burundi, le COGE est élu au sein du COSA en vue d'une cogestion du CDS. Ainsi, si on ne considère que ces résultats, il ressort qu'il y a certains CDS où la cogestion n'est pas garantie. Cela étant, il s'agit probablement d'erreurs de réponses[25], car les COSA sont une condition sinequanone pour les centres de santé pour signer un contrat FBP ; par ailleurs, le COGE est une composante du COSA.

Par ailleurs, 62% des CDS ont répondu disposer d'un COGE dans le cadre du FBP. Ce dernier est constitué uniquement par le personnel du CDS et s'intéresse chaque mois à la planification des recettes et dépenses et au partage d'une éventuelle enveloppe dégagée pour les primes de performance.

De façon quasi similaire à 2014, en moyenne, un centre de santé emploie 15,8 personnes et s'appuie également sur 22,7 agents de santé communautaire. Parmi les employés, 58% sont des hommes; 55% sont dits être employés par l'Etat, 45% par le centre de santé, et 0.4% par une ONG. En moyenne, 55% du personnel a un contrat avec l'état (40% sous-statut, 15% sous-contrat).

De façon quasi similaire à 2014, parmi tous les employés des 90 centres de santé recensés, 25% ont un diplôme de niveau A3 (deux années de formation paramédicale), 26% le niveau A2 (quatre années de formation paramédicale), et seulement 1% le niveau d'un diplôme A1 (diplôme universitaire d'infirmière) ou plus. Au total, 75 (83%) des centres de santé ne disposent pas de prestataires avec le niveau A1 et un (1%) ne dispose pas d'un personnel avec le niveau A1+ ou A2.

---

[25] Les données suggèrent que les réponses négatives proviennent davantage de répondants non titulaires (et donc n'ayant potentiellement pas tous les aspects administratifs du centre de santé en tête). De plus, dans les faits, il se peut qu'un centre dispose d'un COSA avec un seul membre signataire (e.g. le titulaire) ; le répondant (e.g. le titulaire adjoint), s'il n'a jamais vu une réunion de cet organe, pourrait rapporter qu'il y en a pas.

Selon les normes sanitaires du Burundi, un centre de santé devrait avoir au moins trois infirmiers avec un diplôme A2 (ou plus), trois infirmiers avec un diplôme A3, un technicien de laboratoire, un technicien de promotion de la santé ainsi qu'un gestionnaire financier. Seulement 14(16%) (au lieu de 20 soit 22% en 2014) centres de santé respectent ces normes.

Tableau 38. Ressources humaines

Nb centres de santé ayant	2014	2017
**trois ou plus d'infirmières avec un diplôme A2 (ou plus)**	58 (64%)	71 (79%)
**trois ou plus d'infirmières avec un diplôme A3**	69 (77%)	56 (62%)
**un technicien de laboratoire**	50 (56%)	53 (59%)
**un technicien de promotion de la santé**	46 (51%)	48 (53%)
**un gestionnaire financier**	72 (80%)	78 (87%)
**respecté les normes de RH**	**20 (22%)**	**14 (16%)**

L'organigramme du centre de santé a été signalé affiché et mis à jour dans 47 centres de santé (52%), les registres de présence ont été signalés disponibles et mis à jour dans 80 centres de santé (89%) et les fiches descriptives des postes dans 61 centres de santé (68 %). Au total, tout comme lors de l'enquête de référence, seulement 27 centres de santé ont déclaré avoir tous les trois documents disponibles et mis à jour. Soixante-six centres de santé (73% ; au lieu de 88% en 2014) ont déclaré qu'ils évaluent régulièrement la performance de leur personnel. Soixante-douze (80%) des centres de santé procèdent régulièrement à un inventaire des stocks de leurs médicaments (c'était 100% en 2014, mais c'était seulement déclaré ; ici, en 2017, c'est vérifié).

Questionnaire N - Aspects organisationnels des services liés à la malnutrition

Description des services

Programme de suivi et promotion de la croissance

Parmi les 90 centres de santé, 58 (64%) CDS ont déclaré qu'ils offraient des services de suivi et promotion de la croissance (pour 48 : registre vu, pour 10 : registre non vu). Dans le tableau ci-dessous, les mesures et types de conseils et protocole utilisés dans ce cadre. L'analyse des courbes de croissance est réalisée dans seulement 14 centres de santé (10 dans le groupe de traitement, 4 dans le groupe de contrôle).

Les grilles (tables) poids/taille et la formule de calcul de l'IMC sont toutes deux affichées en salle de consultation dans 17 CDS (19%) ; ne le sont pas du tout dans 51 (57%). Elles sont affichées en salle de vaccination dans 16 (18%) ; pas du tout dans 57 (63%).

Tableau 39. Mesures et conseils réalisés et protocoles utilisés dans le cadre du suivi et de la promotion de la croissance en 2017

**Mesures réalisées**	Poids/taille	95%
	Poids/âge	34%
	Taille/âge	19%
	PB	78%
	Courbe de croissance	24% (n=14)
**Conseils réalisés**	Individualisés	64%
	En groupe	84%
	Aucun	2% (n=1)
**Protocole utilisé**	Protocole national de suivi de la croissance	30 (52%)
	Protocole UNICEF	23 (40%)
	PCIME	2 (3%)
	Pas de protocole	2 (3%)
	Autre : table poids pour taille	1 (2%)
	**Nb d'observations**	**90**

Prise en charge de la MAM – service de supplémentation nutritionnelle (SSN)

Parmi les 90 centres de santé enquêtés, 39 (43%) ont déclaré qu'ils avaient un service SSN (29 du groupe de traitement, 10 du groupe de contrôle) ; et 51 (57%) ont déclaré en avoir eu un unjour, mais qu'il était désormais fermé (16 groupe traitement, 35 contrôle).

Les aliments de supplémentation (traitement diétetique) étaient disponibles dans 10 (11%) des centres de santé (5 dans groupe traitement, 5 dans groupe contrôle), et les consommations moyennes mensuelles (CMM) n'étaient calculées que dans cinq centres (cf. Tableau ci-dessous). Au cours des trois derniers mois, quatre-vingt trois (92%) des centres de santé ont connu une rupture de stock dans un des trois produits (répartis également dans les deux groupes de traitement et de contrôle).

Les indicateurs de performance du service pour le PRONIANUT (cf. protocole, MSPLS 2010) sont calculés systématiquement dans 25 (28%) des centres de santé (20 dans groupe de traitement, 5 dans contrôle); ils ne sont jamais calculés dans 41 (45%) des centres de santé (8 dans traitement, 33 dans contrôle).

Enfin, 17 centres de santé ont indiqué qu'ils font des visites de sensibilisation au niveau communautaire pour la récupération des abandons du service SSN.

Tableau 40. Disponibilité du traitement diététique

Nb (%) de CDS qui ont :	
**Farine + sucre + huile**	10 (11%), dont 5 ont calculé la CMM des trois produits
**Rien**	59 (66%)
**Farine**	29 (32%)

Prise en charge de la MAS – Service de thérapeutique ambulatoire (STA)
Parmi les 90 centres de santé enquêtés, 87 (97%) ont déclaré qu'ils avaient un service STA, et trois (3%) ont déclaré en avoir eu un mais qu'il était désormais fermé.

Le traitement diététique (Plumpy Nut®) était disponible dans 80 centres de santé (également réparti entre les deux groupes), mais les consommations moyennes mensuelles (CMM) n'étaient calculées que dans 45 centres (24 du groupe de traitement, 21 du groupe de contrôle). Dix CDS n'avaient pas de traitement diététique disponible. Au cours des trois derniers mois, 39 (43%) des centres de santé ont connu une rupture de stock (17 du groupe traitement, 22 du groupe contrôle).

Les indicateurs de performance du service pour le PRONIANUT (cf. protocole, MSPLS 2010) sont calculés systématiquement dans 62 (69%) des centres de santé (34 groupe traitement, 28 groupe contrôle); ils ne sont jamais calculés dans 3 (3%) des centres de santé.

Interruption des services de nutrition
On a demandé aux répondants si leurs services de nutrition avaient été interrompus au cours des six derniers mois, ce qui était la raison de l'interruption, et quelles ont été les stratégies mises en place pour y faire face. Vingt quatre CDS (27%) n'ont pas eu à interrompre leurs services SSN et STA ; 48 (53%) ont interrompu le SSN seul, 3 (3%) le STA seul, 15 (17%) les deux services SSN et STA. Le tableau ci-dessous montre leur répartition par groupe de traitement :

Tableau 41. Interruption des services de nutrition en 2017

	Groupe contrôle	Groupe traitement	Total
**Pas d'interruption**	9 (20%)	15 (33%)	24 (27%)
**Interruption du SSN seul**	31 (69%)	17 (38%)	48 (53%)
**Interruption du STA seul**	1 (2%)	2 (4%)	3 (3%)
**Interruption des SSN et STA**	4 (9%)	11 (24%)	15 (17%)
**Total**	45	45	90

Dans les 63 CDS où le SSN a été interrompu au cours des six derniers mois, il l'était pour un à deux mois dans 16 cas ; pour trois à six mois dans 16 cas ; pour plus de six mois dans 31 cas. Les raisons invoquées à ces interruptions étaient en grande majorité une rupture de stock (pour 58 CDS, soit 92% des cas) ; huit cas (13%) ont indiqué une autre raison, mais qui restait liée au manque d'intrants (refus d'acheter, problème de fournisseur, départ des ONG, etc.).

Dans les 18 cas où le STA a été interrompu au cours des six derniers mois, il l'était pour un à deux mois dans 15 cas ; pour trois à six mois dans 2 cas. La raison invoquée dans tous les cas est la rupture de stock d'intrants.

Traitement systématique
Le traitement systématique réfère à la médication fournie aux enfants souffrant de malnutrition aiguë. Selon les lignes directrices nationales de 2010 pour le traitement de la malnutrition (en usage pendant la période d'enquête), pour les cas de MAS dans le STA, il consiste à l'amoxicilline, l'artésunate-

amiodiaquine (ASAQ ; si un test pour le paludisme était positif), l'al/mébendazole, l'acide folique (s'il y a des signes d'anémie à l'admission – il est le plus souvent trouvé dans les centres de santé en combinaison avec le fer) et la vaccination antirougeoleuse. Le traitement des cas de MAM dans les services SSN implique la vitamine A, l'al/mébendazole, l'acide folique/fer et la vaccination contre la rougeole (MSPLS, 2010). Les enquêteurs, en collaboration avec l'enquêté, procédaient à la vérification de la disponibilité et de la quantité dans la pharmacie et dans les registres et fiches spécifiques.

Parmi les 90 centres de santé, 71 (79%) avaient l'amoxicilline, 50 (56%) l'ASAQ, 68 (76%) l'al/mébendazole, 38 (42%) l'acide folique, 47 (52%) le fer/folate, et 42 (47%) le vaccin antirougeoleux. Vingt-quatre (27%) disposaient de tous les traitements (également répartis entre les deux groupes). Dans l'ensemble, ces résultats sont bien moins bons qu'en 2014.

Afin d'éviter les ruptures de stock, un outil de gestion, le calcul de la consommation moyenne mensuelle (CMM), est censé être utilisé dans tous les centres de santé. Il semble qu'elle a été calculée dans 50% des centres de santé pour l'amoxicilline, 40% pour l'ASAQ, 47% pour l'al/mébendazole, 37% pour l'acide folique, de 37% pour le fer/folate et 38% pour les vaccins contre la rougeole. Vingt-cinq (28%) des CDS avaient calculé la CMM pour tout (16 dans groupe traitement, 9 dans groupe contrôle). A nouveau, ce sont de bien moins bons résultats qu'en 2014.

Evaluation des dossiers médicaux individuels issus des services liés à la malnutrition

Fiches individuelles de suivi des services de supplémentation nutritionnelle (SSN)

Dans l'ensemble, les enquêteurs ont transcrit les informations relatives à 602 cas cliniques de MAM seulement (par rapport à 1,080 attendus). Il y a un gros déséquilibre entre ce qui a pu être collecté dans le groupe de contrôle (156 fiches) et dans le groupe de traitement (518) : c'est un résultat en soi. Les CDS du groupe de traitement ont manifestement continué à accueillir des cas de MAM dans leurs services, tandis que ceux du groupe de contrôle ont majoritairement négligé ce service (sauf dans 13 CDS, dont 7 sont dans les provinces de Rutana et Ruyigi aidées par le PAM).

Le tableau ci-dessous indique le format de fiche ou de registre utilisé dans le CDS et utilisé donc pour la retranscription des informations cliniques.

Tableau 42. Source des dossiers médicaux individuels du SSN en 2017

Format de fiche utilisé	groupe controle	groupe traitement	Total
**Fiche du protocole de 2010**	132	470	602
**Pas de fiche, utilisation d'un cahier ou de feuilles volantes reprenant plus ou moins les mêmes informations que la fiche du protocole de 2010**	12	12	24
**Pas de fiche, utilisation d'autres documents**	12	36	48
**Total**	**156**	**518**	**674**

Mesures anthropométriques à l'admission

En moyenne, le z-score du rapport P/T à l'entrée dans le programme SSN était -1,64 dans le groupe de traitement et -1.88 dans le groupe de contrôle, soit au dessus de -2, ce qui suggère que, en moyenne, les cas admis dans le service de supplémentation nutritionnelle ne souffraient pas de MAM. Néanmoins, une autre porte d'entrée est le périmètre bracchial, qui doit être entre 115mm et 125mm pour justifier une entrée en SSN : ici la moyenne est de 120mm dans les deux groupes. On remarque par ailleurs que le rapport taille pour âge à l'entrée est, lorsque les informations sont disponibles pour le calculer, en moyenne inférieur à -3 dans le groupe de traitement et inférieur à -2 dans le groupe de contrôle, ce qui signifie qu'en moyenne, les enfants entrés dans le SSN souffrent respectivement de malnutrition chronique sévère et de malnutrition chronique modérée.

Mesures anthropométriques à la sortie et raisons de sortie

Selon les indicateurs de poids-pour-taille et de périmètre brachial à la sortie, en moyenne les enfants entrés dans le service en sont sortis guéris. D'ailleurs, le taux de guérison est de 97% dans le groupe de traitement et de 78% dans le groupe de contrôle. En outre, parmi les guéris, le gain pondéral journalier en grammes par poids initial était en moyenne de 5,50 g / kg / jour dans le groupe de traitement, ce qui est largement au-dessus des directives du protocole national ; tandis que dans le groupe de contrôle on est en moyenne à 2,12 g. Finalement, la durée du traitement chez les guéris était en moyenne de 44 jours dans le groupe de traitement, ce qui est conforme aux directives, et de 70 jours dans le groupe de contrôle ce qui est au-dessus des recommandations des directives nationales (devrait être inférieure à 60 jours).

Tableau 43. Mesures anthropométriques à l'entrée et à la sortie et les raisons de sortie du service SSN

	Groupe de traitement		Groupe de controle	
	Obs	Mean	Obs	Mean
**WHZ à l'entrée**	503	-1.64	144	-1.88
**HAZ à l'entrée**	512	-3.27	144	-2.71
**PB à l'entrée en mm**	507	120.11	144	119.50
**Oedeme à l'entrée**	159	0.04	53	0.00
**WHZ à la sortie**	388	-0.47	102	-0.64
**HAZ à la sortie**	397	-3.40	103	-2.69
**PB à la sortie**	461	125.14	127	124.96
**Oedeme à la sortie**	113	0.01	29	0.00
**Raison sortie: guérison**	**327**	**97%**	**112**	**78%**
**Raison sortie: autre (rupture de stock)**	327	0%	112	11%
**gain de poids entre l'entrée et la sortie en grammes/poids inital en kg/jour de traitement***	**283**	**5.50**	**77**	**2.12**
**durée du traitement en jours entre l'entrée et la sortie***	**288**	**43.96**	**77**	**70.26**

Note : Les informations ci-dessus correspondent à des moyennes des cas pour lesquels les informations étaient disponibles sur la fiche, ce qui n'est pas toujours le cas. Ces renseignements sont calculés pour les déclarés guéris seulement.

Traitement systématique et supplémentation nutritionnelle

Le Tableau 45 donne les statistiques concernant le traitement systématique donné aux patients (tel que rapporté dans les fiches). En moyenne, les résultats semblent meilleurs dans le groupe de contrôle (mais seulement 14 cas).

En ce qui concerne la supplémentation nutritionnelle, nous avons observé que, en moyenne, pour 29% des visites dans le groupe de traitement et 13% dans le groupe de contrôle (N=14), les enfants n'ont reçu aucun supplément alimentaire.

Tableau 44. Performance du service SSN par rapport aux directives nationales

	Directives nationales	Groupe de traitement		Groupe de contrôle	
		% (N)	Atteint ?	% (N)	Atteint?
**Taux de guérison**	>75%	97% (327)	✓	78% (112)	✓
**Taux de tranfert**	<10%	1% (327)	✓	4% (112)	✓
**Taux de décès**	<3%	1% (327)	✓	4% (112)	✓
**Taux d'abandon**	<15%	1% (327)	✓	4% (112)	✓
**Gain pondéral en grammes par poids initial en kg et par jour de traitement, depuis l'entrée jusqu'à la sortie***	>2-3	5.50 (283)	✓	2.12 (77)	×
**La durée du traitement en jours, depuis l'entrée jusqu'à la sortie***	<60	43.96 (288)	✓	70.26 (77)	×

Note : * Ces renseignements sont calculés pour les déclarés guéris seulement.

Tableau 45. Traitement systématique et diététique au service SSN

	Groupe de traitement		Groupe de controle	
	Obs	Mean	Obs	Mean
**Traitement d'al/me bendazole reçu**	226	87%	14	86%
**Traitement de vitamine A reçu**	226	45%	14	79%
**Traitement d'acide folique reçu**	226	35%	14	64%
**Nb de visites sans nourriture % Nb de visites**	**459**	**29%**	**141**	**13%**

Note : Les informations ci-dessus correspondent à des moyennes des cas pour lesquels les informations étaient disponibles sur la fiche, ce qui n'est pas toujours le cas.

Fiches individuelles de suivi des services de thérapeutique ambulatoire (STA)

Dans l'ensemble, les enquêteurs ont retranscrit les informations relatives à 1046 cas cliniques de MAS, sur 1080 attendues. Là, on n'a pas de problème de biais de sélection : environ le même nombre de cas de chaque côté (522 vs 524).

Tableau 46. Source des dossiers médicaux individuels du STA en 2017

Format de fiche utilisé	groupe controle	groupe traitement	Total
**Fiche du protocole de 2010**	44	59	103
**Fiche du protocole de 2014**	438	453	891
**Pas de fiche de protocole, utilisation d'autres documents**	40	12	52
**Total**	**522**	**524**	**1,046**

Mesures anthropométriques à l'admission

En moyenne, le z-score P/T à l'entrée est -2.4, soit au-dessus de -3, (Tableau 47), ce qui signifie qu'en moyenne, les cas admis dans le service de STA ne disposaient pas d'un z-score P/T correspondant à la MAS, mais cela ne donne pas l'image suffisante car dans les cas où l'enfant a aussi des oedèmes, alors le diagnostic de MAS est correct et il est approprié d'être dans le service STA. Plus de détails sur ces aspects sont disponibles dans la section suivante relative à l'évaluation d'impact.

On remarque par ailleurs que le rapport taille pour âge à l'entrée est, lorsque les informations sont disponibles pour le calculer, en moyenne inférieur à -3 dans les deux groupes, ce qui signifie qu'en moyenne, les enfants entrés dans le STA souffrent de malnutrition chronique sévère.

Mesures anthropométriques à la sortie et raisons de sortie

Selon les indicateurs de poids-pour-taille et de périmètre brachial à la sortie, en moyenne les enfants entrés dans le service en sont sortis guéris. D'ailleurs, le taux de guérison est de 92 à 93% dans les deux groupes.

En outre, parmi les guéris, le gain pondéral journalier en grammes par poids initial était en moyenne de 4,62 g / kg / jour dans le groupe de traitement, ce qui est conforme aux directives du protocole national ; tandis que dans le groupe de contrôle on est en moyenne à 3,77 g. Finalement, la durée du traitement chez les guéris était en moyenne de 43 jours dans le groupe de traitement et de 59 jours dans le groupe de contrôle, ce qui est dans les deux cas au-dessus des recommandations des directives nationales (devrait être inférieure à 30 jours).

Traitement systématique et supplémentation nutritionnelle

Le Tableau 49 donne les statistiques concernant le traitement systématique donné aux patients (tel que rapporté dans les fiches). En moyenne, les résultats semblent meilleurs dans le groupe de contrôle.

En ce qui concerne la supplémentation nutritionnelle, nous avons observé que, en moyenne, pour 13-14% des visites dans les deux groupes, les enfants n'ont reçu aucun supplément alimentaire.

Tableau 47. Mesures anthropométriques à l'entrée et à la sortie et les raisons de la sortie du service STA, lorsque l'information est disponible

	Groupe de traitement		Groupe de controle	
	**Obs**	**Mean**	**Obs**	**Mean**
**WHZ à l'entrée**	484	-2.40	407	-2.46
**HAZ à l'entrée**	486	-3.26	416	-3.39
**PB à l'entrée en mm**	472	115.01	422	113.18
**Oedeme à l'entrée**	446	0.18	370	0.21
**WHZ à la sortie**	59	-0.73	53	-0.98
**HAZ à la sortie**	60	-3.04	57	-3.55
**PB à la sprtie**	464	122.69	448	121.52
**Oedeme à la sortie**	18	0.00	27	0.00

Note : Les informations ci-dessus correspondent à des moyennes des cas pour lesquels les informations étaient disponibles sur la fiche, ce qui n'est pas toujours le cas. Ces renseignements sont calculés pour les déclarés guéris seulement.

Tableau 48. Performance du STA comparée aux directives nationales (de 2010)

	Directives nationales	Groupe de contrôle		Groupe de traitement	
		% (N)	Atteint ?	% (N)	Atteint ?
**Taux de guérison**	>75%	93% (387)	✓	92% (350)	✓
**Taux de tranfert**		1% (387)		3% (350)	
**Taux de décès**	<5%	2% (387)	✓	1% (350)	✓
**Taux d'abandon**	<13%	3% (387)	✓	2% (350)	✓
**Taux de non répondant**	<7%	2% (387)	✓	2% (350)	✓
**Gain pondéral en grammes par poids initial en kg et par jour de traitement, depuis l'entrée jusqu'à la sortie***	>4-6	3.77 (315)	×	4.62 (278)	✓
**La durée du traitement en jours, depuis l'entrée jusqu'à la sortie***	<30	59.06 (317)	×	42.87 (282)	×

Note : * Ces renseignements sont calculés pour les déclarés guéris seulement.

Tableau 49. Traitements systématique et diététique en STA

	Groupe de traitement		Groupe de controle	
	**Obs**	**Mean**	**Obs**	**Mean**
**Traitement d'amoxicilline reçu**	512	62%	482	64%
**Traitement de vitamine A reçu**	512	12%	482	22%
**Traitement d'al/me bendazole reçu**	512	26%	482	37%
**Traitements d'amoxicilline et al/mebendazole reçus**	**512**	**24%**	**482**	**35%**
**Nb de visites sans nourriture % Nb de visites**	435	14%	449	13%

Note : Les informations ci-dessus correspondent à des moyennes des cas pour lesquels les informations étaient disponibles sur la fiche, ce qui n'est pas toujours le cas.

Qualité des services de santé

Observations directes de consultations pédiatriques (Questionnaire C)

En général

L'analyse du questionnaire C se concentre principalement sur l'interrogatoire et l'examen observés et dans une moindre mesure sur les tests de laboratoire demandés, les diagnostics et les traitements. Comme nous ne pouvons pas être sûrs que les diagnostics sont corrects, il est difficile de commenter les traitements administrés et une exploration approfondie de ces décisions cliniques est au-delà de la portée de ce rapport. En tant que tel, notre principal point d'attention s'est borné aux directives internationales (PCIME, manuel pédiatrique de l'OMS) et nationales (directives nationales sur la malnutrition 2010) dans les étapes initiales de la consultation.

D'une manière générale, les résultats étaient très inquiétants en 2014. Ils le sont encore plus aujourd'hui.

Un des premiers aspects de l'approche PCIME est de vérifier trois signes de danger pour l'enfant : l'enfant est-il capable d'avaler en toute sécurité (boisson ou allaitement), a-t-il eu ou a-t-il des vomissements, a-t-il eu des convulsions (OMS 2014) ? Au total, aucun cas n'a été interrogé sur ces trois questions par les prestataires. Au total, 448 (85%) cas n'ont été interrogés sur aucun de ces trois signes de danger.

L'autre priorité de la PCIME est d'accorder une attention particulière aux «principaux symptômes» (WHO 2014: 2-5). Cela passe par une série de questions sur la présence de diarrhée, de toux, de difficultés respiratoires, de fièvre et de problèmes d'oreille. Dans cette enquête, aucun cas n'a été interrogé sur toutes les cinq questions, et 71 (13%) n'ont été interrogés sur aucun de ces symptômes. Un autre aspect de la PCIME est de vérifier le statut vaccinal de l'enfant : au total, 43 cas (8%) seulement ont été interrogés pour vérifier si leurs vaccinations étaient à jour.

D'un point de vue plus global, dans notre grille d'observation des consultations, sur un total de 31 questions dans la section de l'interrogatoire, il y avait 23 questions autonomes (c'est-à-dire des questions qui ne se rapportent pas à la réponse de la question précédente ; une question non-autonome est par exemple celle sur la durée de la diarrhée – elle ne sera posée que si une présence de la diarrhée a été rapportée). Aucun patient n'a été interrogé sur toutes les 23 questions. Un cas (0,2%) a été interrogé sur 13 questions ; ce fut le nombre maximum de questions posées. Six cas (1%) n'ont été interrogés sur aucune de ces 23 questions. Le Tableau 50 résume les principaux résultats de la section du questionnaire de l'interrogatoire.

Tableau 50. Nombre de cas interrogés sur les signes de danger, les principaux symptômes et les questions autonomes

Questions relatives à…	2014<br><br>cas interrogés<br>n (%)<br><br>N=514	2017<br><br>cas interrogés<br>n (%)<br><br>N=528
Aux signes de danger - toutes les trois posées	**2 (0.3%)**	**0 (0%)**
capable de boire/avaler	24 (5%)	29 (5%)
vomissements	164 (32%)	42 (8%)
présence de convulsions	39 (8%)	14 (3%)
Aux cinq principaux symptômes - toutes les cinq posées	**2 (0.3%)**	**0 (0%)**
diarrhée	267 (52%)	239 (45%)
toux	261 (51%)	317 (60%)
difficultés respiratoires	31 (6%)	24 (5%)
fièvre	302 (59%)	319 (60%)
problèmes d'oreilles	59 (12%)	60 (11%)
Au statut vaccinal	**70 (14%)**	**43 (8%)**
Toutes les questions autonomes du questionnaire C (23)	***0 (0%)***	***0 (0%)***
Nombre maximum posé: 13	*3 (0.6%)*	*1 (0.2%)*
Aucune des questions posées	*4 (0.8%)*	*6 (1%)*

Il y avait 19 examens physiques figurant dans le questionnaire. Aucun des cas observés n'a subi tous les examens. Le nombre maximal d'examens effectués a été de 13 (deux cas) ; pour 57 cas (11%) aucun des examens physiques énumérés dans le questionnaire n'a été effectué sur eux.

En outre, huit cas seulement ont vu les trois principaux signes vitaux (température, pouls et le rythme respiratoire) mesurés ; 193 cas (37%) n'avaient aucun des signes vitaux enregistrés. Le Tableau 51 présente les résultats sur les examens physiques.

Tableau 51. Nombre de cas avec examens et signes vitaux effectués

Examen physique	2014<br>cas observés<br>n (%)<br>N=512	2017<br>cas observés<br>n (%)<br>N=528
**Signes vitaux - tous les trois ont été mesurés**	**0 (0%)**	**8 (2%)**
température	300 (58%)	327 (62%)
pouls	2 (0.4%)	11 (2%)
rythme respiratoire	33 (6%)	30 (6%)
pas de signes vitaux pris	205 (40%)	193 (37%)
Tous les examens du questionnaire C (19)	***0 (0%)***	***0 (0%)***
Nombre maximum réalisé: 14	*1 (0.2%)*	*Max: 13 - 2(0.4%)*
Aucun examen effectué	*88 (17%)*	*57 (11%)*

Nutrition

Sept questions figurant dans la grille d'observation des consultations portent spécifiquement sur la nutrition : les sept sont pertinentes pour les enfants de moins de deux ans, tandis que cinq sont pertinentes pour les enfants de plus de deux ans (pour ceux-là, les questions sur l'allaitement sont retirées ; voir Tableau 52). Parmi les 234 enfants de moins de deux ans, aucun n'a été questionné sur toutes les sept questions de nutrition. Dans 116 cas (50%), aucune de ces questions n'avait été posée. Parmi les 293 enfants âgés de plus de deux ans, aucun n'a été questionné sur toutes les cinq questions ; dans 146 cas (50%), aucune des questions relatives à la nutrition n'a été posée.

Dans la grille d'observation, six aspects de l'examen physique sont spécifiquement liés à l'état nutritionnel de l'enfant: poids, taille, périmètre brachial, oedèmes, dessiner / discuter de la courbe de croissance, et calcul du rapport P/T. Aucun cas n'a vu en 2017 tous ces six examens réalisés dans le cadre de leurs soins ; 172 cas (33%) n'ont reçu aucun de ces examens.

A partir des statistiques rapportées dans le tableau suivant, on ne distingue pas une amélioration de comportement vis-à-vis de la nutrition dans le groupe de traitement. A confirmer dans les analyses plus poussées.

Au total, 112 cas (21%) ont reçu des conseils de nutrition. Les catégories des conseils les plus fréquemment rapportées sont les suivantes : apport de denrées spécifiques, combiner trois catégories d'aliments, régime 'équilibré', diversifier.

Tableau 52. Questions et examens relatifs à la nutrition exécutés

	2014	2017		
**Questions et examens relatifs à la nutrition**	Total<br>n (%)	Total<br>n (%)	Traitement<br>n (%)	Contrôle<br>n (%)
**Questions de nutrition**	**N=250**	**N=234**	**N=106**	**N=128**
cas de moins de 2 ans interrogés sur les 7 questions	0 (0%)	0 (0%)	0 (0%)	0 (0%)
cas de moins de 2 ans sans aucune question posée	106 (42%)	116 (50%)	62 (58%)	54 (42%)
	**N=200**	**N=293**	**N=159**	**N=134**
cas de plus de 2 ans interrogés sur les 5 questions	0 (0%)	0 (0%)	0 (0%)	0 (0%)
cas de plus de 2 ans sans aucune des cinq questions posées	93 (47%)	146 (50%)	85 (53%)	61 (46%)
**Examens de Nutrition / tâches**	**N=513**	**N=524**	**N=263**	**N=261**
tous les six examens / tâches	13 (3%)	0 (0%)	0(0%)	0 (0%)
aucun des examens / tâches	211 (41%)	172 (33%)	89 (34%)	83 (32%)
poids	268 (52%)	281 (53%)		
taille	165 (32%)	162 (31%)		
PB	153 (30%)	238 (45%)		
oedèmes	64 (13%)	56 (11%)		
courbe de croissance	37 (7%)	21 (4%)		
rapport P/T	95 (19%)	41 (8%)		

Malnutrition
Pour une appréciation complète de la précision du diagnostic et l'orientation des cas de malnutrition aiguë, les résultats du questionnaire C doivent être confrontés aux mesures anthropométriques et à l'évaluation des œdèmes faite par les enquêteurs avec le questionnaire S, en utilisant les équipements propres à l'enquête (utilisés comme mesures de référence).

Selon les résultats du questionnaire S, il y avait 83 cas (16%) de MA (MAM ou MAS) dans l'échantillon. Cependant, seuls 18 enfants (3%) au total ont reçu un diagnostic de MA par l'agent de santé lors des observations de consultations (questionnaire C ; cas répartis également entre les deux groupes). Sur ces 18 cas, seulement quatre ont été orientés dans le bon service..

Entretiens à la sortie (Questionnaire S)

Compréhension du diagnostic
Les enquêteurs ont posé aux accompagnants la question 'qu'est-ce qui ne va pas avec votre enfant?' après la consultation avec l'agent de santé. Sur 525 accompagnants interrogés, 316 (60%) ont donné une réponse qui correspond soit à un diagnostic soit à un ensemble de symptômes ; 209 (40%) accompagnants ont en revanche déclaré ne pas savoir ce qui n'allait pas avec leur enfant après la consultation. Parmi les 316 accompagnants qui ont déclaré que l'agent de santé leur avait dit ce qui n'allait pas avec leur enfant, 295 (93%) ont donné un diagnostic réel alors que 21 (7%) ont raconté les signes et symptômes dont leur enfant souffrait.

Aussi, sur les 316 cas qui ont déclaré que l'agent de santé leur avait dit ce qui n'allait pas avec leur enfant, aucun n'a rapporté le diagnostic de «malnutrition». Ce chiffre est à comparer avec les 18 cas de malnutrition aiguë diagnostiqués par les AS lors des consultations observées (questionnaire C), et les 83 cas réels de malnutrition aiguë selon les mesures anthropométriques et l'évaluation des œdèmes faites par les enquêteurs à la sortie (questionnaire S).

Compréhension du traitement
Au total, 12% des accompagnants ont déclaré que l'AS avait prescrit des médicaments, mais n'avait pas donné d'autres conseils ou d'autre explication sur l'état ou le traitement de l'enfant. De plus, 27% ont indiqué que l'agent de santé n'a pas donné des conseils, mais avait prescrit des médicaments et a dit à l'accompagnant de revenir pour un suivi s'il n y a pas d'amélioration malgré le traitement. Seulement 13 cas (2.5%) ont rapporté qu'ils avaient reçu le conseil d'améliorer l'alimentation.

Conseils nutritionnels
Au total, 379 cas sur 529 (71%) n'ont reçu aucun conseil nutritionnel. Parmi les 148 ayant reçu des conseils, 107 (72%) ont eu des conseils pour donner des aliments spécifiques ; 42 (28%) ont reçu comme conseil de donner trois catégories d'aliments ; 14 (9%) trois repas par jour ; 19 (13%) ont reçu des conseils d'hygiène.

Mesures anthropométriques

Des t-tests ont été utilisés pour comparer les mesures anthropométriques prises par l'AS et par les enquêteurs à la fois en utilisant les équipements des centres de santé et aussi ceux de l'enquête. Toutes les différences calculées au niveau de l'échantillon total ont été estimées comme significatives.

Tableau 53. Différences dans les mesures anthropométriques
entre enquêteur et agent de santé, et entre matériel d'enquête et du CDS, en 2017

	Différence absolue de poids entre enquêteur et AS (en kg)	Différence absolue de taille entre enquêteur et AS (en cm)	Différenceabsolue de PB entre enquêteur et AS (en mm)	Différence absolue de poids entre balance SECA et balance du CDS (en kg)
moyenne	**0.40**	**1.61**	**7.48**	**0.32**
**n**	375	231	257	512
**se**	0.51	2.32	7.41	0.44
**min**	0.00	0.00	0.00	0.00
**max**	4.00	19.80	48.00	3.70

Enquête au niveau des ménages

Sécurité alimentaire

Les conditions liées à l'insécurité alimentaire des ménages

Les résultats de l'enquête de référence montrent qu'il y a eu des réponses positives pour les neuf questions de survenance des conditions d'insécurité alimentaire. La fréquence de survenue des conditions d'insécurité alimentaire est relativement élevée, car seules deux conditions reviennent dans moins de 50% des ménages, ces deux conditions étant celles témoignant d'une insécurité alimentaire grave. Plus de 70% des répondants ont expérimenté les six premières conditions d'insécurité alimentaire. Le Tableau 54 présente ces résultats.

Tableau 54. Conditions de sécurité alimentaire

	2014		2017	
**Conditions**	Proportion	Obs.	Proportion	Obs.
Fréquence de ménages:				
1. ayant été préoccupé par le manque de nourriture	79%	6196	83%	6474
2. dont un membre n'a pas pu manger les aliments préférés par manque de ressources	86%	6195	89%	6476
3. dont un membre a mangé une variété plus limitée d'aliments par manque de ressources	85%	6198	89%	6477
4. dont un membre a mangé des aliments qu'il n'aime pas manger par manque de ressources	84%	6194	90%	6478
5. dont un membre a mangé un repas plus petit par manque de ressources	70%	6195	77%	6478
6. dont un membre a mangé moins de repas par jour par manque de ressources	62%	6196	72%	6477
7. ayant manqué totalement de nourriture par manque de ressources	61%	6196	42%	6478
8. dont un membre est allé au lit en ayant faim par manque de ressources	43%	6197	50%	6476
9. dont un membre a passé la journée sans manger par manque de ressources	17%	6197	25%	6477

La prévalence de l'insécurité alimentaire des ménages

Considérant ces réponses et la méthode d'échelle de l'accès déterminant l'insécurité alimentaire des ménages pour la mesure de l'accès alimentaire des ménages (cf. section 2), cette enquête révèle que 65% des répondants expérimentent une insécurité alimentaire grave, tandis que 26% des répondants expérimentent une insécurité alimentaire modérée (Tableau 55).

Tableau 55. Prévalence des différentes catégories de sécurité alimentaire

	2014		2017	
**Catégories d'insécurité alimentaire**	Proportion	Obs.	Proportion	Obs.
Sécurité alimentaire	8%	6189	6%	6472
Insécurité alimentaire légère	5%	6189	3%	6472
Insécurité alimentaire modérée	19%	6189	26%	6472
Insécurité alimentaire grave	68%	6189	65%	6472

Etat de santé, statut nutritionnel et allaitement de l'enfant

Etat de santé de l'enfant

Plus de la moitié des enfants enquêtés (55%) ont été malades dans les deux semaines précédant l'entretien. Tout comme en 2014, la plainte la plus communément rencontrée était la fièvre (55%) et la

diarrhée et l'infection respiration étant survenues chacune chez un tiers des enfants. La majorité de ces enfants (81%) ont été amenés en consultation au centre de santé ; et 99% des répondants ont affirmé que leur enfant été connu au centre de santé (l'enfant avait déjà visité le centre de santé ou un autre un bon nombre de fois). Ce pourcentage important d'enfants ayant été amenés en consultation est impressionnant dans la mesure où 39% des répondants doivent faire plus d'une heure pour arriver à la formation sanitaire. Davantage d'enfants qu'en 2014 ont été rapportés comme étant suivis dans un service de supplémentation nutritionnelle (SSN) ou un service thérapeutique ambulatoire (STA) : 5% au lieu de 1%. La majorité des enfants (91%) avaient un carnet de santé (Tableau 56).

Tableau 56. Etat de santé de l'enfant

	2014		2017	
	Freq.	Obs.	Freq.	Obs.
**A été malade les deux dernières semaines**	59%	6199	55%	6480
A eu de la diarrhée	35%	3668	28%	3577
A eu de la fièvre	55%	3668	55%	3577
A eu une infection respiratoire	33%	3668	33%	3577
A consulté le centre de santé	81%	3668	81%	3577
**Est connu au centre de santé**	100%	6199	99%	6480
**Temps pour arriver au centre de santé: moins de 30 minutes**	37%	6180	27%	6423
**Temps pour arriver au centre de santé: plus d'une heure**	48%	6180	39%	6423
**Est suivi au SSN ou au STA**	1%	6199	5%	6480
**Possède un carnet de santé**	79%	6199	91%	6480

Pratiques relatives à l'allaitement

La majorité des indicateurs relatifs à l'allaitement étaient satisfaisants par rapport aux recommandations nationales sur les pratiques de l'Alimentation du Nourrisson et du Jeune Enfant (ANJE, WHO 2010; cf. Tableau 57). Ceci peut être expliqué en partie, par l'importance culturelle que revêt l'allaitement au Burundi.

Les pratiques relatives à l'alimentation de complément

Approximativement, pour la moitié des enfants, toutes catégories d'âge confondues, la personne responsable a rapporté que l'enfant recevait le nombre minimum de groupes d'aliments recommandés (quatre). De même, seuls deux cinquièmes des enfants reçoivent globalement le nombre minimum de repas recommandés par jour et par rapport à leur âge, avec une meilleure fréquence chez les enfants les plus âgés quoique le chiffre reste en-deçà de ce qui serait considéré comme satisfaisant. Sans surprise, tout comme en 2017, seul un quart des enfants a un score satisfaisant à l'indicateur combinant le minimum de fréquence des repas et le minimum de diversité alimentaire. Le Tableau 58 donne plus de détails sur les pratiques de l'alimentation de complément.

Mesures anthropométriques

Le Tableau 59 ci-dessous présente les principales variables anthropométriques mesurées dans les enquêtes auprès des ménages.

Tableau 57. Indicateurs relatifs à l'allaitement

	Recommandations nationales	2014		2017	
**Indicateurs**		Freq.	Obs.	Freq.	Obs.
Enfants ayant été allaités		99.9%	6197	99.7%	6480
Allaitement initié dans l'heure après l'accouchement	85%	86.1%	6090	87.5%	6340
Allaitement initié dans les 24 heures suivants l'allaitement		98.0%	6090	97.9%	6340
Allaitement exclusive jusqu'à l'âge de six mois	80%	81.7%	6074	81.7%	6389
Les enfants qui continuent d'être allaité ou ont été allaité jusqu'à l'âge de 12mois (pour les enfants de 12-15mois)		97.7%	1364	98.9%	1560
% des enfants qui continuent d'être allaité jusqu'à l'âge de 20 mois (pour les enfants de 20-23 mois)	80%	85.0%	1238	85.3%	1285
Moyenne d'âge de sevrage (parmi les enfants déjà sevrés)		14.7 mois	427	14.57	429

Tableau 58. Indicateurs relatifs à l'alimentation de complément

**Indicateurs**	2014		2017	
	Freq.	Obs.	Freq.	Obs.
**Nombre minimum recommandé de groupes d'aliments (i.e. diversité alimentaire minimale) - min. 4 groupes**	49.3%	5542	51.5%	5822
**Fréquence minimale de repas par jour**	43.8%	6164	40.3%	6440
6-11 mois	32.8%	2176	30.7%	2235
12-17 mois	47.6%	2067	43.6%	2244
18-23 mois	52.1%	1921	47.4%	1956
**Minimum alimentaire acceptable (i.e. fréquence et diversité satisfaisante)**	25.0%	6144	25.4%	6433
6-11 mois	17.7%	2173	19.1%	2235
12-17 mois	27.6%	2060	28.9%	2239
18-23 mois	30.5%	1911	28.7%	1954
**Supplémentation en Vitamine A ou fer dans les six derniers mois**	78.7%	6192	48.3%	6459

Tableau 59. Mesures anthropométriques

	2014		2017	
	Moy.	Obs.	Moy.	Obs.
PB (mm)	139.92	6199	138.10	6479
PB < 115mm	2.0%	6199	3.3%	6479
Œdèmes	1.8%	6199	1.3%	6479
z-score P/T pour enfants de 6-11mois	-0.26	2234	-0.43	2240
z-score P/T pour enfants de 12-17mois	-0.44	2113	-0.55	2259
z-score P/T pour enfants de 18-23mois	-0.32	1848	-0.41	1975
**Malnutrition aigüe (tous les enfants)**	**6.0%**	**6199**	**8.7%**	**6480**
Enfants de 6-11 mois avec malnutrition aigüe	6.1%	2234	9.7%	2240
Enfants de 12-17 mois avec malnutrition aigüe	6.9%	2113	10.0%	2260
Enfants de 18-23 mois avec malnutrition aigüe	4.8%	1849	6.2%	1975
**Malnutrition aigüe sévère (tous les enfants)**	**1.2%**	**6199**	**1.8%**	**6480**
Enfants de 6-11 mois avec MAS	1.1%	2234	2.4%	2240
Enfants de 12-17 mois avec MAS	1.5%	2113	1.9%	2260
Enfants de 18-23 mois avec MAS	0.8%	1849	1.2%	1975
z-score T/A pour enfants de 6-11 mois	-1.72	2234	-1.63	2240
z-score T/A pour enfants de 12-17 mois	-2.19	2113	-2.22	2259
z-score T/A pour enfants de 18-23 mois	-2.46	1849	-2.39	1975
**Retard de croissance (tous les enfants)**	**53.1%**	**6199**	**50.9%**	**6480**
Enfants de 6-11 mois avec retard de croissance	39.0%	2234	37.3%	2240
Enfants de 12-17 mois avec retard de croissance	57.1%	2113	54.6%	2260
Enfants de 18-23 mois avec retard de croissance	65.5%	1849	62.2%	1975
**Retard de croissance sévère (tous les enfants)**	**21.0%**	**6199**	**20.7%**	**6480**
Enfants de 6-11 mois avec retard de croissance sévère	12.5%	2234	12.3%	2240
Enfants de 12-17 mois avec retard de croissance sévère	22.8%	2113	23.0%	2260
Enfants de 18-23 mois avec retard de croissance sévère	29.2%	1849	27.7%	1975
Pas de courbe de croissance dans le carnet de santé	89.6%	4917	97.1%	5887
Courbe ascendante	93.7%	510	92.3%	168
Courbe stationnaire	3.9%	510	3.0%	168
Courbe descendante	2.4%	510	4.8%	168

Synthèse

La méthodologie utilisée et suivant les questions génériques HFIAS d'échelle de l'accès déterminant l'insécurité alimentaire des ménages pour la mesure de l'accès alimentaire des ménages a permis d'observer que 65% des ménages enquêtés en 2017 subissaient une grave insécurité alimentaire, 26% une insécurité alimentaire modérée, et 9% une insécurité alimentaire faible ou nulle. Ces chiffres sont

toujours aussi impressionnants et inquiétants qu'en 2014 et mettent certainement en évidence un domaine qui nécessite de l'attention.

Tout comme en 2014, plus de la moitié des enfants enquêtés avaient été malades durant les deux semaines précédant l'enquête (55%), et 81% d'entre eux ont consulté les services de santé, ce qui semble assez élevé si on note que 40% des répondants reportaient une distance au centre de santé supérieure à une heure. La majorité des indicateurs relatifs à l'allaitement étaient satisfaisants au regard des recommandations nationales d'alimentation du jeune enfant (ANJE). Cependant, les pratiques d'alimentation complémentaire n'étaient en moyenne pas satisfaisantes : seule la moitié des enfants avaient une diversité alimentaire suffisante, à savoir minimum quatre groupes alimentaires, et 40% avaient une fréquence de repas suffisante ; au total, seuls un quart des enfants recevaient une alimentation complémentaire acceptable en terme de fréquence et de diversité (indicateurs adaptés selon leur âge).

Enfin, les données collectées en mars-avril 2017 ont permis d'observer un taux de malnutrition aigüe parmi les enfants de 6-23 mois de 8.7%, et un taux de malnutrition chronique de 50.9% à cette période..

8.3 Statistiques descriptives des données de routine du FBP Nutrition (groupe d'intervention)

Tableau 60. Détail des évaluations qualité dans le groupe d'intervention sur 2015-2016

Suivi de la croissance	Barême	Score validé (moyenne sur 2015-2016)	Nb d'occurences avec score maximal (/285)	Score validé en 2015 (moyenne)	Score validé en 2016 (moyenne)
Disponibilité et fonctionnalité des outils/matériel pour le suivi de la croissance	20	18.2	213	18.5	17.9
Les enfants de moins de deux ans dont le rapport poids pour âge est supérieur à -2ET	30	14.8	119	12.1	17.8
Les enfants de moins de deux ans fréquentent les séances de suivi et promotion de la croissance	30	18.9	179	17.1	20.9
Pour chaque session individuelle de suivi et promotion de la croissance, conseils appropriés délivrés	30	23.2	206	22.6	23.9
Les courbes de croissance P/A et T/A sont bien tracées	10	3.4	96	3.1	3.7
**SSN**					
Inventaire trimestriel de l'équipement et matériel pour le service existe et actualisé	10	5.3	152	4.1	6.6
Disponibilité et fonctionnalité des outils/matériel pour le dépistage et la prise en charge	20	14.8	178	11.9	17.9
Respect des critères d'admissions (Prendre 10 fiches individuelles au hasard)	10	5.2	149	3.0	7.7
Registre de PEC correctement remplis	10	4.6	130	2.1	7.2
PEC correcte de 20 cas (Analyse du registre des cas choisi au hasard et fiches individuelles)	20	9.3	106	3.9	15.1
Critères de performances	30	15.4	135	12.8	18.2
Disponibilité des intrants pour la prise en charge et traitement systématique	10	2.8	81	1.2	4.6
**STA**					
Inventaire trimestriel de l'équipement et matériel pour le service existe et actualisé	10	6.4	183	5.7	7.2
Disponibilité et fonctionnalité des outils/matériel pour le dépistage et la prise en charge	20	18.4	234	18.4	18.3
Respect des critères d'admissions (Prendre 10 fiches individuelles au hasard)	10	8.9	255	8.7	9.2
Registre de PEC correctement remplis :Remplissage de toutes les informations requises selon le registre STA	10	8.1	230	7.5	8.7
PEC correcte de 20 cas (Analyse du registre des cas choisis au hasard et fiches individuelles)	30	25.0	170	24.4	25.7
Critères de performances	30	9.5	86	3.0	16.5
Disponibilité des intrants pour la prise en charge et traitement systématique	10	6.7	191	6.5	6.9

Source : données de routine du FBP Nutrition des 45 CDS du groupe d'intervention sur 2015 et 2016 (285 observations en tout).

Tableau 61. Indicateurs FBP Nutrition validés par semestre en 2015 et 2016

		Groupe d'intervention									
	Semestre	N	Moyenne	SD	Min	Max	Median	IQR	Somme pour les 45 CDS sur les 6 mois	Moy CDS sur 6 mois	Median CDS sur 6 mois
**Nb de cas de MAM guéris et validés par mois et par CDS**	2015 S1	266	2.1	7.0	0	61	0	0	571	12.9	0
	2015 S2	270	3.4	10.1	0	56	0	0	923	20.5	0
	2016 S1	270	44.9	49.8	0	424	34	45	12136	269.7	201
	2016 S2	270	51.0	97.6	0	1009	24	65	13767	305.9	144
**Nb de cas de MAS guéris et validés par mois et par CDS**	2015 S1	266	10.2	14.6	0	134	7	9	2712	61.2	42
	2015 S2	270	12.8	14.5	0	118	9	15	3465	77.0	54
	2016 S1	270	26.0	31.5	0	270	18	25	7025	156.1	105
	2016 S2	270	28.6	33.0	0	371	21	26	7709	171.3	126
**Nb de références validées par mois et par CDS**	2015 S1	266	0.9	9.8	0	137	0	0	237	5.3	0
	2015 S2	270	1.9	21.4	0	342	0	0	510	11.3	0
	2016 S1	270	2.6	15.1	0	164	0	0	699	15.5	0
	2016 S2	270	4.4	35.7	0	524	0	0	1182	26.3	0
**Nb de suivis de la croissance validés par mois et par CDS**	2015 S1	266	67.6	136.9	0	715	0	62	17990	405.8	0
	2015 S2	270	141.2	177.5	0	730	64	219	38134	847.4	381
	2016 S1	270	228.9	217.4	0	1040	187	324	61796	1373.2	1119
	2016 S2	270	302.2	276.5	0	1534	242	343	81602	1813.4	1449

Source: données de routine du projet FBP Nutrition. Le N correspond au nombre de CDS multiplié par le nombre de mois pour lesquels on a des données de routine.

8.4 Tableaux synthétiques sur les résultats de l’intervention (mise en œuvre et impact) et les recommandations politiques

Tableau 62. Mise en œuvre du FBP Nutrition à travers la loupe de la théorie du changement

	Route	Observations
**FBP**	**Revenus**	-paiements effectués (avec retards cependant, cf. ci-dessous)
	**Incitations**	-paiements aux CDS primaires : premiers paiements arrivés avec 6 mois de retard ; arriérés de plusieurs mois réguliers dans le FBP d’une manière générale tout le long du projet ; difficulté de repérer les subsides spécifiques au FBP Nutrition pour les CDS primaires<br>-paiements aux CDS secondaires : effectués auprès des CDS primaires, qui devaient transférer auprès des CDS secondaires<br>-paiements aux GASC : retards de plusieurs mois tout du long<br>-premiers scores qualité non pris en compte pour ne pas démotiver les CDS
	**Information**	-CDS primaires : formation et contrats OK<br>-CDS secondaires : pas de formation, contrats avec un retard 12 mois<br>-GASC : formations et contrats avec un retard 9 mois
	**Supervision**	-vérification quantitative : formation OK, mise en œuvre avec retard de 3 mois pour le premier mois à vérifier, puis régulièrement et à temps par la suite<br>-vérification qualitative : formation et mise en œuvre avec retard de 4 mois pour le premier trimestre à évaluer, puis régulièrement et à temps par la suite<br>-pas de contre-vérification spécifique au FBP Nutrition<br>-pas de formations à l’endroit des superviseurs > pas plus de supervision relative à la nutrition

Tableau 63. Résultats sélectionnés: statistiques descriptives dans le groupe d'intervention en 2017 et impact estimé du FBP Nutrition

	Hypothèse	Résultats
Inputs et Activités	**Meilleur équipement ?**	-3.6 éléments/7, impact n.s.<br>-7% des CDS ont équipement complet, impact n.s.<br>-330g de différence entre balance CDS et balance SECA, impact n.s.
	**Activités nutrition de meilleure qualité** ?	-nb moyen de questions nutrition posées :0.7/7 chez les moins de deux ans, 0.6/5 chez les plus de deux ans ; 1.2/7 avec les vignettes, impacts n.s.<br>-nb moyen d'examens nutrition effectués : 1.4/6 en consultation, 2.6/6 avec vignettes, impacts n.s.<br>-conseils nutritionnels donnés dans 18.9% des consultations, impact négatif -12.3pp (p=0.06) ; donnés dans 31.5% des vignettes, impact négatif -24.7pp (p=0.01)<br>-conseils hygiène donnés dans 10.2% des consultations, et dans 13.6% des vignettes, impacts n.s<br>-courbe de croissance mentionnée dans 3.3% des vignettes, impact n.s.<br>-différences moyennes mesures anthropométriques entre AS et enquêteur : 110g pour le poids, 1cm pour la taille, 7.5mm pour le PB, impact n.s.<br>-diagnostics de malnutrition trouvés dans 16% des cas de MAS compliqués, 26% des MAS non compliqués, 33% des MAM (vignettes), impact n.s.
	**Meilleure coordination avec les ASC** ?	- encadrement des ASC pour le dépistage dans 78% des CDS, impact n.s.<br>- encadrement des ASC pour la prise en charge via démonstrations culinaires ou FARN dans 53% des CDS, impact positif +50.4pp (p=0.000)<br>-encadrement des ASC pour la sensibilisation dans 71% des CDS, impact n.s.
	**Recrutement et/ou formation de personnel** ?	-normes RH respectées dans 8.9% des CDS, impact n.s.<br>-formation nutrition reçue chez 52% des AS, impact n.s.<br>-aucune formation reçue chez 32% des AS, impact n.s.
	**Pression sur les fournisseurs d'intrants diététiques ?** (meilleure disponibilité ?)	-traitement MAM dispo dans 11% des CDS, impact n.s.<br>-traitement MAS dispo dans 88% des CDS, impact n.s.<br>-traitement systématique complet dispo dans 29% des CDS, impact n.s.
	**Davantage de supervision rel. nutrition ?**	-supervision sur la malnutrition par l'ECD durant le dernier trimestre dans 42% des CDS, impact n.s.
	**Meilleure tenue de la documentation rel. malnutrition ?**	-registre SSN dispo et à jour dans 71% des CDS, impact +68.8pp (p=0.000)<br>-registre STA dispo et à jour dans 82.2% des CDS, impact +21.6pp (p=0.000)<br>-indicateurs de performance SSN calculés dans 44.4% des CDS, impact +40.9pp (p=0.000)<br>-indicateurs de performance STA calculés dans 75.6% des CDS, impact +24.7pp (p=0.039)<br>-paramètres d'entrée et de sortie du SSN tous renseignés dans 15.3% des fiches, impact n.s.<br>-paramètres d'entrée et de sortie du STA tous renseignés dans 0.6% des fiches, impact n.s.

	Hypothèse	Résultats
**Outputs**	**Meilleurs et plus nombreux dépistages d'enfants malnutris ?**	-malnutrition diagnostiquée dans 3.4% des consultations, impact n.s.<br>-malnutrition non diagnostiquée dans 38 cas de patients souffrant de malnutrition (88% de faux négatifs), impact n.s<br>-paramètres d'entrée dans le SSN corrects dans 44% des cas, impact n.s.<br>-paramètres d'entrée dans le SSN auraient dû amener au STA dans 38% des cas, impact n.s.<br>-paramètres d'entrée dans le STA corrects dans 89% des cas ; impact n.s.<br>-nb moyen de cas de SSN par CDS et par semestre : 122, impact +138 (p=0.000 ; selon enquête), 306 selon données de routine FBP Nutrition<br>-nb moyen de cas de STA par CDS et par semestre : 79, impact +53 (p=0.000 ; selon enquête), 171 selon données de routine FBP Nutrition<br>-5.6% d'enfants inscrits dans un SSN ou STA dans la communauté (enquête ménage), impact n.s.
	**Meilleurs soins délivrés aux enfants souffrant de malnutrition** ?*	-intrants diététiques non reçus dans 29% des visites de patients inscrits en SSN<br>-intrants diététiques non reçus dans 14% des visites de patients inscrits en STA<br>-traitements reçus par les patients inscrits en SSN : al/me bendazole dans 87% des cas, vit. A dans 45% des cas, acide folique dans 35% des cas<br>-traitements reçus par les patients inscrits en STA : vit. A dans 12% des cas, al/me bendazole + amoxicilline dans 24% des cas
	**Meilleur contrôle du suivi de la croissance** ?	-courbe de croissance évoquée dans 4.2% des consultations, impact n.s.<br>-séances de suivi de la croissance reportées comme ayant lieu dans 78% des CDS, impact n.s.<br>-courbe de croissance dessinée sur le carnet de 4.6% des enfants (enquête ménage), impact n.s.<br>-nb moyen de cas de suivi de la croissance validés : 0 durant semestre 1 de 2015, 1449 durant le semestre 2 de 2016 (données de routine FBP Nutrition)
	**Davantage d'activités au niveau de la communauté** ?	-23.8% des ménages ont déclaré avoir participé à une séance de sensibilisation ou de démonstration culinaire, impact +4.3pp (p=0.000)

	Hypothèse	Résultats
Résultats	**Résultats dans le service SSN meilleurs ?** (traitement de la MAM)	-taux de guérison 97%, impact +14.7pp (p=0.007)<br>-gain de poids chez les guéris +3.4g/kg et jour, impact n.s.<br>-durée de traitement chez les guéris ; 44 jours, impact -29 (p=0.047)<br>-paramètres de sortie du SSN « guéri » corrects dans 74% des cas, impact n.s.<br>-taux de non-répondants : 1%, impact -4.2pp (p=0.057)
	**Meilleurs résultats dans le service STA ?** (traitement de la MAS)	-taux de guérison 92%, impact n.s.<br>-gain de poids chez les guéris +4.6/kg et jour, impact n.s.<br>-durée de traitement chez les guéris ; 43 jours, impact -19 (p=0.021)<br>-paramètres de sortie du STA « guéri » corrects dans 99% des cas, impact +19 (p=0.036)<br>-taux de non-répondants : 2%, impact n.s.
	**Connaissances et pratiques des mamans améliorées ?**	-alimentation correcte pour 27% des enfants âgés de 6-23 mois, impact n.s.<br>-jugement correct sur l'état de malnutrition aigüe de son enfant chez 70% des mamans, impact n.s.
Résultats finaux	**Prévalence de malnutrition aigüe diminue** ?	-prévalence de malnutrition aigüe(chez les 6-23 mois) : 8.7%, impact n.s.<br>-z-score poids pour taille moyen (chez les 6-23 mois) : -0.46, impact n.s.<br>-périmètre brachial moyen (chez les 6-23 mois) : 138 mm, impact n.s.
	**Prévalence de malnutrition chronique diminue** ?	-prévalence de retard de croissance (chez les 6-23 mois) : 52%, impact n.s.<br>-z-score taille pour âge moyen (chez les 6-23 mois) : -2.08, impact n.s.
	**Prévalence d'épisodes infectieux diminue** ?	-prévalence d'épisodes infectieux durant les 2 semaines avant l'enquête : 53.9% des enfants, impact n.s.

Note : par souci de synthèse, les résultats donnés sont ceux des CDS du groupe d'intervention uniquement ; * le calcul de l'impact n'était pas possible pour les indicateurs sur les soins délivrés aux enfants inscrits en SSN ou STA.

Tableau 64. Recommandations politiques : problèmes identifiés et actions proposées

Problème	Solution propose	Responsable	Priorité
Retards de paiements (effet incitatif du FBP réduit)	Améliorer la disponibilité des ressources en amont	MSPLS/CT-FBP avec partenaires	Court terme
Equipement rel. nutrition insuffisant	Garantir l'équipement initial de base à tous les CDS	MSPLS/PRONIANUT avec partenaires	Court terme
	Initier un fonds propre à chaque CDS destiné à l'entretien et au renouvellement de l'équipement fourni	MSPLS avec partenaires	Court terme
	Fournir du matériel pédagogique et documents de références aux GASC	MSPLS/DPSHA avec partenaires	Court terme
Ruptures de stock fréquentes	Améliorer les conditions de production et les modalités de mise sur le marché d'aliments fortifiés pour la MAM	MSPLS avec partenaires (notamment PAM)	Court terme
	Légiférer pour permettre la fortification des aliments sur place avec les moyens locaux	MSPLS avec partenaires (notamment PAM)	Court terme
	Améliorer la disponibilité de PlumpyNut®	MSPLS avec partenaires (not. UNICEF)	Moyen terme
	Améliorer la disponibilité des traitements systématiques (vitamine A, amoxicilline, etc.) en faveur des enfants admis en services nutritionnels	MSPLS avec partenaires	Court terme
Coordination insuffisante avec les ASC	Assurer la présence d'un TPS dans chaque CDS	MSPLS	Moyen terme
Documentation sanitaire (nutrition) davantage disponible mais de faible qualité pour ce qui est des fiches cliniques	Réviser les indicateurs de l'évaluation qualitative pour une prise en compte globale des outils de collecte des données, tout en insistant sur le fait que c'est pour aider les AS à mieux gérer les cas individuels	MSPLS/CT-FBP avec partenaires	Court terme, fréquence annuelle
Ressources humaines en trop faible quantité	Assurer que les normes en ressources humaines soient satisfaites dans tous les CDS	MSPLS	Moyen terme

Problème	Solution propose	Responsable	Priorité
Ressources humaines manquant de connaissances	Effectuer des recherches qualitatives sur les attitudes et les opinions des AS pour élucider les défis et les besoins des AS à l'égard de leur travail quotidien dans les CDS	MSPLS	Court terme
	Evaluer l'efficacité des formations et recyclages régulièrement proposés par le MSPLS et partenaires aux CDS	MSPLS	Court terme
	Organiser des formations basées sur les besoins des AS, auprès des AS appropriés (e.g. pas systématiquement les titulaires) au dépistage et à la prise en charge de la malnutrition	PRONIANUT	Moyen terme
	Organiser des formations basées sur les besoins des AS, auprès des AS appropriés (e.g. pas systématiquement les titulaires) à la PCIME	Point Focal PCIME	Moyen terme
	Former les ASC de manière appropriée au dépistage de la malnutrition aigüe, à l'art culinaire et au processus de rapportage	PRONIANUT	Court terme
	Réviser le système FBP pour améliorer la qualité des soins	MSPLS/CT-FBP avec partenaires	Moyen terme
	Améliorer la qualité des supervisions et des vérifications pour assurer une formation en continu (cf. plus bas)	MSPLS/DODS	Court terme
	Créer des centres d'excellence pour des problèmes spécifiques (e.g. malnutrition) et y organiser de stages pour assurer des formations continues des AS ciblées et pratiques	MSPLS/PRONIANUT	Moyen terme
Non adhérence aux protocoles de soins en vigueur	Assurer la disponibilité des protocoles dans les CDS	MSPLS/DODS/CT-FBP	Court terme
	Identifier des solutions pour assurer l'adhérence aux protocoles (e.g. supervision et audit de l'utilisation de ces protocoles, indicateurs FBP, etc.)		Court terme
Efficacité des supervisions médiocre	Evaluer le système de supervision et réviser les documents de références et outils de supervision	MSPLS/DODS	Court terme
	Former les superviseurs à ces nouveaux outils pour faire en sorte que leurs visites de supervision soient efficaces en termes de transfert de connaissances théoriques et planifiées de manière réaliste (e.g. en termes de temps, selon contexte géographique, besoins des AS)	MSPLS/DODS	Moyen terme
	Renforcer les connaissances des superviseurs sur le dépistage et la prise en charge de la malnutrition et sur le suivi de la croissance	MSPLS/PRONIANUT	Moyen terme

Problème	Solution propose	Responsable	Priorité
Efficacité du système de vérifications médiocre	Evaluer le système de vérification du FBP et réviser les documents de références et outils en conséquence	MSPLS/CT-FBP/DODS	Court terme
	Former les vérificateurs à ces outils (pour qu'ils vérifient correctement les indicateurs et qu'ils donnent des feedbacks productifs aux AS)	MSPLS/CT-FBP/DODS	Moyen terme
	Evaluer et améliorer les activités de contre-vérification (pour qu'elles contiennent un maximum d'activités sous FBP contre-vérifiées et pour in fine améliorer l'activité de vérification)	MSPLS/CT-FBP	Court terme, fréquence annuelle
Indicateurs FBP pas toujours appropriés	Réviser la grille des indicateurs FBP (quanti et quali) ; en particulier, retirer les éléments redondants, rendre la grille d'indicateurs plus cohérente, et réviser les pondérations des différents indicateurs)	MSPLS/CT-FBP	Court terme, fréquence annuelle
	Réviser régulièrement les indicateurs (part. qualité) pour inciter à toujours une meilleure qualité de services	MSPLS/CT-FBP	Court terme, fréquence annuelle
Les prévalences de malnutrition dans la communauté restent importantes malgré les meilleures prises en charge de MA	La malnutrition est un problème multifactoriel que le système de santé seul ne peut résoudre : la recommandation ici est de travailler sur l'avancement du plan stratégique multisectoriel de sécurité alimentaire et de nutrition (http://scalingupnutrition.org/fr/pays-sun/burundi/).	Point-focal SUN avec partenaires	Court, Moyen et Long terme
