# Supplementary material for: Impact of the extension of a performance-based financing scheme to nutrition services in Burundi on malnutrition prevention and management among children below five: A cluster-randomized control trial
Source: PLoS One. 2020 Sep 18;15(9):e0239036. doi: 10.1371/journal.pone.0239036 (PMC7500612; doi:10.1371/journal.pone.0239036)
Supplement: Attachment — Submitted filename: Response to Reviewers.docx [file pone.0239036.s012.docx]

Response to reviewers

Ref. : PONE-D-19-30259

Title : Impact of the extension of a performance-based financing scheme to nutrition services in Burundi on malnutrition prevention and management among children below five: a cluster-randomized control trial

Comments received on 7^th^ of April 2020.

Responses written in May-June 2020, sent on the 4^th^ of June 2020.

References to numbers of pages and lines correspond to the unmarked revised version.

|  | Journal Requirements: |  |
| --- | --- | --- |
|  | When submitting your revision, we need you to address these additional requirements. |  |
| 1 | 1. Please ensure that your manuscript meets **PLOS ONE's style requirements**, including those for file naming. The PLOS ONE style templates can be found at <http://www.journals.plos.org/plosone/s/file?id=wjVg/PLOSOne_formatting_sample_main_body.pdf> and <http://www.journals.plos.org/plosone/s/file?id=ba62/PLOSOne_formatting_sample_title_authors_affiliations.pdf> | It was reviewed accordingly. Among other edits, we reviewed the title page; reduced the abstract to meet the 300 words count requirement; edited figures and tables in the manuscript; changed parentheses of citations into brackets; and removed the abbreviations’ section. |
| 2 | 2. In your **Methods section**, please provide additional information about the participant recruitment method and the demographic details of your participants. Please ensure you have provided sufficient details to replicate the analyses such as:  a) the recruitment date range (month and year),  b) a description of any inclusion/exclusion criteria that were applied to participant recruitment,  c) a table of relevant demographic details,  d) a statement as to whether your sample can be considered representative of a larger population,  e) a description of how participants were recruited, and  f) descriptions of where participants were recruited and where the research took place. | (a) Page 7, lines 1-2, this sentence was added in the “participants” subsection: “*Surveyed households, health workers and patients were recruited on the field, at the time of the survey (see the procedures below).”*   For household surveys:   (a) the recruitment date range is, at baseline, in December 2014 and January 2015, and at end-line, in March and April 2017. It is specified page 11 lines 4-6. (b) This sentence was added for the eligibility criteria: “*Eligible households were those living in the area of the study health centres and having a member aged 6-23 months at the time of the survey*.” Page 11 lines 6-7. (c) A table of relevant demographic details is given in the supplementary data (S1). This sentence was added page 11, lines 13-14: *“Demographic details about households and children characteristics at baseline, in 2014, are displayed in S1 Table.”* (d) This sentence was added page 11, lines 14-17: *Chronic malnutrition indicators computed for our sample of children were similar to those found in the nationally representative DHS 2010 survey for same age children, which make us confident that our sample can be considered representative of all households with a toddler living in Burundi at that time.* (e) & (f) Page 11, lines 10-13, the selection of households was detailed: *six eligible households were selected as follows: a random direction from the centre of the sub-hill is selected and following it leads to the first contact with a household, which in turn indicates the next households with children aged 6-23 months*   For Health centres surveys:   (a) This was added page 12 lines 14-15. *Surveyed health workers and patients were recruited at the time of the surveys, as described below.* (c) A table of relevant demographic details does not apply to health centres. (d) This was added page 13, lines 18-22: *The 90 HC selected for the study cover all areas of Burundi and all provinces but Bujumbura city have at least one HC in the sample, and, although this sample is not representative of the whole health sector in Burundi, since neither hospitals nor community health workers were surveyed, we are confident that this sample is representative of all health centres of the rural part of Burundi. Health centre characteristics and nutrition services performance indicators were rather similar to other nationally representative data*. (e) & (f) & (c) The “recruitment” of clinical files was edited to give more details, page 12-13: *These 24 clinical files were randomly selected among all the closed files of all children enrolled acute malnutrition services (12 files for the MAM care program and 12 files for the SAM care program) during a six-month period preceding the survey where both services were procured with malnutrition treatments (March-September 2013 for baseline and April-October 2016 for endline). This constitutes more than a thousand of files for each MAM and SAM service and survey wave.*   Also, this was added page 13 lines 9-13: *For the latter, two health workers are randomly selected among those performing paediatric curative consultations the day of the survey; then six children aged 6-59 months, coming for a consultation the day of the survey, are randomly selected, among the eligible patients in the waiting room ,for being observed in consultation and surveyed afterwards.* |
| 3 | 3. Please correct your reference to "p=0.000" to **"p<0.001"** or as similarly appropriate, as p values cannot equal zero. | Correction made page 23 lines 3 and 4 |
| 4 | 4. Please provide additional details regarding **participant consent**. In the ethics statement in the Methods and online submission information, please ensure that you have specified  (1) whether consent was informed and  (2) what type you obtained (for instance, written or verbal).  If your study included minors, state whether you obtained consent from parents or guardians. If the need for consent was waived by the ethics committee, please include this information. | These two sentences were added page 6 lines 6-8, after the statement on Ethics approvals by ethics committees:  *Informed written consents were obtained for all participants of the study, that is: health workers, household heads, and mothers (or child caregivers) for themselves and their children.* |
| 5 | 5. Thank you for stating the following in the **Competing Interests section**: "CK, SN and MN declare no conflict of interest. BM has contributed to the emergence of PBF as a global health policy, through technical assistance, research and knowledge management. He is the lead facilitator of the PBF Community of Practice. He holds minority shares in Blue Square, a firm developing health system software solutions. "  Please confirm that this does not alter your adherence to all PLOS ONE policies on sharing data and materials, by including the following statement: **"This does not alter our adherence to PLOS ONE policies on sharing data and materials.”** (as detailed online in our guide for authors <http://journals.+-plos.org/plosone/s/competing-interests>).  If there are restrictions on sharing of data and/or materials, please state these. Please note that we cannot proceed with consideration of your article until this information has been declared.  **Please include your updated Competing Interests statement in your cover letter**; we will change the online submission form on your behalf.  Please know it is PLOS ONE policy for corresponding authors to declare, on behalf of all authors, all potential competing interests for the purposes of transparency. PLOS defines a competing interest as anything that interferes with, or could reasonably be perceived as interfering with, the full and objective presentation, peer review, editorial decision-making, or publication of research or non-research articles submitted to one of the journals. Competing interests can be financial or non-financial, professional, or personal. Competing interests can arise in relationship to an organization or another person. Please follow this link to our website for more details on competing interests: <http://journals.plos.org/plosone/s/competing-interests> | This statement applies and we will include it to the competing interests section: *“**This does not alter our adherence to PLOS ONE policies on sharing data and materials”.*  As for restrictions on sharing of data, please see the response to #10, copy-pasted below:  May we change our data statement for this one, which may be more explicit about the process to follow?  *“**Raw data of the impact evaluation were generated at the national institute of public health of Burundi, INSP (*[*https://insp.bi/*](https://insp.bi/)*), for the health facility data, and at the national institute of statistics of Burundi, ISTEEBU (*[*http://www.isteebu.bi/*](http://www.isteebu.bi/)*), for the household data. All data are owned by ISTEEBU and are available from them on request, for researchers who meet the criteria for access to confidential data, by sending an e-mail to the Director General of ISTEEBU:* [*isteebu@isteebu.bi*](mailto:isteebu@isteebu.bi) *or* [*isteebubdi@gmail.com*](mailto:isteebubdi@gmail.com)*.”*  A synthesis of the dataset was added as supporting information:  S1 Dataset. synthesis of datasets used for analysis, with summary statistics (mean, median, sum, variance, standard deviation, number of observations) by survey and treatment group. Source: Authors. |
| 6 | 6. Please **remove your figures from within your manuscript file**, leaving only the individual TIFF/EPS image files, uploaded separately.  These will be automatically included in the reviewers’ PDF. | Figures were removed accordingly. |
| 7 | 7. Please **include captions** **for your Supporting Information files** at the end of your manuscript, and update any in-text citations to match accordingly. Please see our Supporting Information guidelines for more information: <http://journals.plos.org/plosone/s/supporting-information> . | This was reviewed accordingly. |
|  | Reviewers' comments: |  |
|  | Questions |  |
| 8 | 1. Is the manuscript technically sound, and do the data support the conclusions? The manuscript must describe a technically sound piece of scientific research with data that supports the conclusions. Experiments must have been conducted rigorously, with appropriate controls, replication, and sample sizes. The conclusions must be drawn appropriately based on the data presented.  Reviewer #1: Partly Reviewer #2: Yes Reviewer #3: Yes |  |
| 9 | 2. Has the statistical analysis been performed appropriately and rigorously?  Reviewer #1: No Reviewer #2: Yes Reviewer #3: I Don't Know |  |
| 10 | 3. Have the authors made all data underlying the findings in their manuscript fully available?  The [PLOS Data policy](http://www.plosone.org/static/policies.action#sharing) requires authors to make all data underlying the findings described in their manuscript fully available without restriction, with rare exception (please refer to the Data Availability Statement in the manuscript PDF file). The data should be provided as part of the manuscript or its supporting information, or deposited to a public repository. For example, in addition to summary statistics, the data points behind means, medians and variance measures should be available. If there are restrictions on publicly sharing data—e.g. participant privacy or use of data from a third party—those must be specified.  Reviewer #1: No Reviewer #2: Yes Reviewer #3: No | May we change our data statement for this one, which may be more explicit about the process to follow?  Raw data of the impact evaluation were generated at the national institute of public health of Burundi, INSP (<https://insp.bi/>), for the health facility data, and at the national institute of statistics of Burundi, ISTEEBU (<http://www.isteebu.bi/>), for the household data. All data are owned by ISTEEBU and are available from them on request, for researchers who meet the criteria for access to confidential data, by sending an e-mail to the Director General of ISTEEBU: [isteebu@isteebu.bi](mailto:isteebu@isteebu.bi) or [isteebubdi@gmail.com](mailto:isteebubdi@gmail.com).  A synthesis of the dataset was added as supporting information:  S1 Dataset. synthesis of datasets used for analysis, with summary statistics (mean, median, sum, variance, standard deviation, number of observations) by survey and treatment group. Source: Authors. |
| 11 | 4. Is the manuscript presented in an intelligible fashion and written in standard English?  PLOS ONE does not copyedit accepted manuscripts, so the language in submitted articles must be clear, correct, and unambiguous. Any typographical or grammatical errors should be corrected at revision, so please note any specific errors here.  Reviewer #1: Yes Reviewer #2: Yes Reviewer #3: Yes |  |
|  | 5. Review Comments to the Author |  |
|  | Reviewer #1: |  |
| 12 | The study has followed a cluster-randomised controlled trial design, with half of the 90 selected HCs randomly allocated to intervention and the other half constituted the control group. Data were collected at both cluster and individual levels using two cross-sectional surveys. The methods were well documented; however, I have major concerns on the overall trial design, statistical analysis and results presented in this paper. | Thank you for your careful review and comments. We hope that with our amendments your concerns will be vanished. |
| 13 | The CONSORT flow chart suggested that 193 HCs were assessed for eligibility, 100 were randomised, and 90 were selected and enrolled to the study. The authors did not explain why only half of the HCs were randomised, and the discrepancy between 100 and 90 HCs that were randomised. In principle, only eligible clusters should be randomised and included in final analysis. | The number of selected HCs (90) was decided following the sample size calculation (moved to the “randomization” subsection page 7 and edited as follows:  *The sample size was computed on the smallest difference in the main outcome that could be considered of public health significance, i.e. a reduction of  25% in acute malnutrition prevalence (2.5% points in absolute terms) in the communities around the intervention HCs as compared to control HCs. Assuming that the intervention would result in decreasing the prevalence of acute malnutrition in children aged 6-23 months from 10% to 7.5% (DHS survey 2010), and assuming that 65 children aged 6-23 months would be surveyed in the catchment area of each health centre, 90 health centres needed to be randomized to either the intervention or control group, for an α-error of 5% and a β-error of 20%, and taking a conservative intraclass correlation (ICC) of 0.25. This was calculated with the formula of (Hayes & Bennett 1999). The number of children per HC was increased to 72 to allow for missing or incomplete data, amounting to a total of 6,480 children aged 6-23 months over the 90 selected HCs, at each survey round (baseline in 2014 and endline in 2016).*  We were aiming for 90 HC. We initially selected randomly 100 HC, with the idea that some would be removed because they would be too close to each other (that was one exclusion criteria: whenever two selected HC would be too close to each other (that is, less than 5km), one would be removed from the list of study HC). Finally, only two HC were too close (so one was removed for this reason. However, while gathering qualitative information, it appeared that nine HC from the list did not provide MAM or SAM services, so they were excluded at that time. In order to make that clear, we added this note to the CONSORT diagram:  **Information about the existence of both MAM and SAM services was initially obtained from the Ministry of Health; once the randomization was performed, while gathering more information from each HC, we realised than nine of them did not provide either the MAM (1) or the SAM (8) services. Providing both services was an inclusion criterium, so these nine HC were removed from the list.* |
| 14 | The sample size of the household surveys was based on a reduction of 25% in acute malnutrition prevalence between HCs in the two groups. The number of children recruited in each HC was set to 72, from 12 sub-hills per cluster and 6 eligible households per sub-hill. This adds up to a total of 6,480 children aged 6-23 months surveyed at the community level. Was the same target used for baseline survey, which as reported included a total of 6,199 children? | See response to 13 above. The number of children to be recruited around each HC was increased to 72 as a precaution in case of non-completion of all required interviews (missing data). However, what we really needed according to the calculation was 65 children per HC, that is 5850 children in total, which was achieved in both baseline and endline surveys. |
| 15 | For a cluster randomised trial, sample size calculation should be based on both the number of clusters and average cluster size with pre-specified statistical power (>80%) and significance level (normally 5%). Another important parameter to consider is the inflated design effect related to the intra-cluster correlation coefficient (ICC), which is the proportion of the total variance of the outcome that can be explained by the variation between clusters.  The flow chart should also include information on both clusters and individuals, as recommended by the CONSORT 2010 statement with extension to cluster randomised trials. | For the main outcome at community level, the ICC taken initially was 0.25 for the sample size calculation; it appeared from the baseline data that it was only 0.006. This was added to the text on sample size calculation, pages 7 line 18- page 8 line 2:  *(…) and taking a conservative ICC of 0.25. (…) Now, according to the baseline data, the prevalence of acute malnutrition in children aged 6-23 months is 6%, instead of 10%, with an ICC of 0.007. Considering this, the explanatory power for a reduction of 25% (from 6% to 4.5%) was reduced from 80% to 60%, while the explanatory power for a reduction from 6% to 4% remained at 80%.*  For the main outcome at HC level, the ICC taken initially was 0.15 for both MAM and SAM recovery rates. It appeared from our estimates that it was 0.41 for MAM and 0.06 for SAM. We added this in the method section, pages 12-13:  *This sample size was computed on the smallest difference in the main outcome that can be considered of public health significance in intervention health centres, and assuming that the intervention would result in increasing the recovery rate of both MAM and SAM in children from 80% to 90%, for an α-error of 5%, a power of 80% and an ICC of 0.15* [1]    And this as an introductory paragraph to the findings section, page 19  *As explained above, we did not gather the 1,080 clinical files initially planned. Furthermore, in some patient files, the outcome as for the end of the treatment was missing. At baseline, the MAM and SAM outcomes were reported in respectively 628 and 665 clinical files. This affected the explanatory power of our tests. At baseline, the ICC for MAM recovery outcome was assessed to be up to 0.41 (based on observations from 26 HC for which we had the outcome information for 12 clinical files), which reduced the explanatory power to 60% for a recovery rate of increase from 80% to 95%. The ICC for SAM was estimated at 0.06, and the explanatory power remained at 80% with an α-error of 5% assuming that the intervention would result in increasing the recovery rate of SAM from 85% to 95%.*  As for the CONSORT flow chart, we updated it by adding the number of inhabitants covered by HCs (responsibility area population) for both the intervention and the control groups. Adding the number of households surveyed or clinical files transcribed would seem to be inappropriate. |
| 16 | For outcome analysis, the statistical model has included both time points while only the end-line survey data were potentially impacted by the intervention. The beta coefficients estimated the difference-in-difference, which would be hard to quantify the actual effect sizes between intervention and control groups on the defined outcomes. A standard approach is to analyse the end-line survey data only using the mixed models with a random cluster effect, with the assumption that the clusters were similar at baseline by randomisation and matching. | There were some differences between the two groups in the main outcomes, so we thought it was more reasonable to use a difference-in-difference analysis. The effect sizes were transcribed in odds ratios and in marginal impacts which are both common ways to read and interpret findings. |
| 17 | As required by the CONSORT guidelines, baseline characteristics at cluster and individual participant levels should be presented in a table for each group. Continuous variables can be summarised by the mean and standard deviation, and numbers and proportions should be reported for categorical variables. The supplementary table 1 needs to be modified following the guidelines.  The outcome tables are hard to follow, and the numbers are inconsistent. For example, how were the total numbers of observation 1,254 and 1,549 obtained in Table 2? The column titles Mean and N were used for all outcomes regardless of their distributions.  With an interaction term between group and period in the main model, which term was used to estimate the effects of intervention on different outcomes should be clearly stated so that the estimated coefficients, odds ratios, and incidence rate ratios can be interpreted appropriately.  With more than one primary outcome and a large number of variables tested overall (386), any significant differences observed between intervention and control groups should be concluded with caution without controlling for overall Type 1 error rate. | Baseline main characteristics (for primary and secondary outcomes considered in this study) are reported in tables 2 to 5 for both groups. The statistics provided have been changed accordingly: mean, SD and number of observations for continuous variables and proportion, occurrences and number of observations for categorical variables. S1 Table has also been changed accordingly.  The outcome tables display both baseline and endline statistics for both groups on the left side, and provide also the impact statistics on the right side, which we find both comprehensive and illustrative, so we kept that amount of information. We however changed the structure of the findings, displaying first the categorical variables, and second the continuous variables; and the impact measurement (estimator) is now clearly specified. As for the number of observations you mentioned (1,254 and 1,549), these were mistakes which have been fixed.  That’s the interaction term *PBFnut_ht_* that was used to estimate the impact of the PBF-N intervention on the different outcomes as precised in the text (in the ‘statistical analysis’ sub-section), page 16:  *The variable PBFnut_ht_ is an interaction term multiplying group with period: it equals 1 only in the intervention HCs and after the intervention, and 0 otherwise; the beta coefficient provides an estimate of the impact of the PBF-N on the dependent variable.*  The estimator (logit, OLS, Poisson) is now specified clearly in the tables displaying the results, and the models used follow the equation provided in the ‘statistical analysis sub-section.  The large number of variables tested overall you refer to (386) was performed only for the baseline analysis, to compare the two groups in a comprehensive manner. Here, for the purpose of the impact evaluation and of this paper specifically, the focus is on the primary outcomes that were decided upon at the protocol writing stage (that is, acute and chronic malnutrition prevalence among children aged 6-23 months at the community level, and recovery rates of MAM and SAM cases among children aged 6-59 months) – for which one variable shows a significant impact (MAM recov rate, p=0.039). Now, in order to interpret these findings and get a more comprehensive understanding of what happened, we decided to open the analysis to secondary outcome and output indicators (like treatment duration, quality of malnutrition screening, etc.). In total we display findings for 21 indicators in this paper, for which five were showing significant impacts. |
|  | Reviewer #2: | Thank you for your careful review and comments. |
| 18 | 1. Abstract. The word “data” is plural so it should be “data were”. | This was changed accordingly. |
| 19 | 2. p. 9. The last paragraph purports to explain the theory of change of the intervention. It does not do so. Having this explanation is essential especially for understanding why the authors expected health system improvements to have reduced chronic or acute malnutrition prevalence, an expectation that on the face of it does not seem warranted because health systems cannot remove most of the causes of the malnutrition (as the authors note in the Discussion). What the authors have provided here is useless for explaining the theory of change. The authors have basically said to the reader to go find out for yourself by going to the supplementary material, which is irritating and not acceptable. S2-S5 appear to have two files duplicated twice. The protocol is in French; PLoS One is an English language publication. Please revise to explain clearly in the main text of the manuscript the theory of change of the intervention for both recovery from and prevention of malnutrition. | We understand your frustration, so we tried to provide more comprehensive explanations while giving clear links to the most appropriate documents for even more details:  -The paper [2] we refer to in this paragraph is the research protocol in its published form and is written in English.  -We added a reference [3], which provides the revised ToC in the light of the impact evaluation findings at the health centre level.  -Here in the sentence we added page 10, lines 17-20:  *In another paper, recently published, we report on more extensively our findings as for the theory of change* [3]*.Qualitative data suggest that the most influencing mechanisms related to PBF-N were incentives coming with PBF-N as well as information provided in the contract and during the initial training (stating clearly the service performance indicators).*  S2-S5 files (now S3-S6 files) are not duplicated twice, although we understand that at first sight it can seem to be so. There were two separate research protocols for this study, one at the health centre level and one at the community level (for the household surveys). The more comprehensive research protocols are the ones written in their original language, French (so there are two documents in French). There is also an English version, lighter, for each research protocol: that was the version approved by the University of Antwerp Ethics committee (so there are also two documents in English). The four files will remain in the supporting information, however, we won’t cite them anymore in the text, so that readers prefer reading the published papers relating to the ToC ([2] & [3]). |
| 20 | 3. p. 14, top. The authors write “We talk about shortness when LAZ is lower than minus two standard deviations (SD) and stunting when it’s lower than minus three SDs.” WHO (and just about everyone else) defines stunting as less than -2 SD, not -3 SD. Please revise and use the standard definitions to avoid confusion. | Indeed, there was an error here, thank you. This was changed accordingly, see page 14:  *Chronic malnutrition identification is based on length-for-age z-scores (LAZ)and height-for-age z-scores (LAZ and HAZ) respectively for children below and above two years old. Length-for-age and height-for-age deficits indicate past or chronic inadequacies of nutrition and/or chronic or frequent illness (24). We talk about shortness A child below two years old is stunted when their LAZ is lower than minus two standard deviations (SD) and severely stunted when it’s lower than minus three SDs*. |
| 21 | 4. p.15. Where it says “The time varying variable PBFnutht is an an interaction term…” delete “time varying”. This term is not time varying in the common use of this descriptor. | That was changed accordingly. |
| 22 | 5. p. 22. P-values, by definition, cannot be zero. All p-values given as 0.000 should be given as <0.001. | That was changed accordingly. |
| 23 | 6. The manuscript requires careful technical editing and preparation. Overall, the writing is understandable, but many technical problems are throughout the manuscript. Furthermore, the manuscript was not carefully prepared, for example, line numbers in only part of the manuscript. | Careful editing was performed. |
|  | Reviewer #3: |  |
|  | • General comments |  |
| 24 | This is a quality paper on an important nutrition issue using state-of-the art methodology (RCT) with particularly careful pairing of control and intervention HCs. It has often been assumed that health personnel and workers did not have enough incentives to devote more effort to nutrition services and therefore, it was hypothesized that performance-based monetary incentives would boost the nutrition performance and impact. There are some issues, however minor, that need to be addressed. | Thank you for your careful review and comments. |
|  | • Specific comments |  |
| 25 | **1. Abstract** The objectives should be given. The results section is not very clear : 97% - 78% = 19%, not 14.7pp (what is “pp”?). Unless this is not the way the results should read. | The objective was added: *“The objective of this study is to assess the impacts of this intervention, on both child acute malnutrition recovery rates at health centre level and prevalence of chronic and acute malnutrition among children at community level.”*  As for the results, ‘pp’ means percentage points. We changed for Odds Ratios as it is a more common way to read this kind of results (but we keep translating it in terms of percentage points in the findings section) |
| 26 | **2. Introduction :** a. Hunger and food insecurity are among the root causes of malnutrition. Malnutrition encompasses several forms including undernutrition. In addition to poverty and food insecurity, there are factors of child undernutrition which are in the health realm, beyond nutrition care and services, and management of undernutrition, that is, poor access to health care as well as WASH. This could be clearer in the paper.  b. Community-base management of undernutrition (and prevention) is now advocated, although it is clear that health personnel are involved, at community, as well as HC and hospital level. The expected nutrition activities and services at these three levels should be described, perhaps in general and in the Burundi context. | a. Indeed, this was missing. We edited this paragraph page 3, lines 17-23:  *Malnutrition is multifactorial: poverty and food security are central elements in low-income countries, while water, sanitation and hygiene are found to be important factors of chronic malnutrition among children* [4]*. There is also a consensus that the health system has a role to play* [5]*, especially at the health centre level, where generic perinatal care, paediatric prevention and care services are provided: generic health services can prevent and manage generic child health issues that could lead otherwise to malnutrition, while specific to malnutrition interventions aim to prevent, identify and manage malnutrition.*  b. This was added page 4, lines 3-14:  *Community-based management of acute malnutrition (CMAM), although highly relevant* [5]*, was not sufficiently invested in Burundi at the time of the study. CMAM includes 1) community outreach as the basis for early identification and referral of malnourished children, follow up through home visits, sensitisation and mobilisation of the community; 2) management of moderate acute malnutrition (MAM); 3) outpatient treatment for children with uncomplicated severe acute malnutrition (SAM); and, 4) inpatient treatment for children with complicated SAM. In Burundi, active screening was to be performed by CHWs, and systematic screening at health centres through pre-school consultations and curative consultations of children under five; outpatient treatment services for MAM and uncomplicated SAM cases are integrated in the primary health care services, in about one third of health centres. Complicated SAM cases are managed in the hospital, before being transferred back to outpatient treatment and follow-up at the health centre. As for sensitisation at community level, this was mainly performed by NGOs, in only a few provinces.* |
| 27 | **3. Methods** a. How many health centers in total for 200 providing nutrition services?  b. The rationale for the different age range in the health center and the community assessment should be given.  c. Fig. 1: The unit is the health center, please specify  d. Institut national de santé publiQUE  e. Who gets the subsidy and how is it shared in the case of hospitals, HCs and CHWs?  f. What is the MAM treatment supply? It should be explicit as it is often referred to.  g. Shortness and stunting: are these widely accepted cut-offs and terms? Above the age of 24 months, it should be stated that standing height is measured, not length.  h. Please clarify that the duration of the intervention was two years.  i. Is it possible to provide the list of the 19 indicators of quality of nutrition services, perhaps as supplementary data?  j. According to Table 2, CHWs are to screen and refer acute malnutrition cases to HCs. However, is this not accomplished through growth monitoring and promotion activities, which are HC’s activities?  k. The protocol is not needed even in appendix; besides, it is in French. | a. There were 590 HC in total in Burundi, for 193 HC reported as providing both MAM and SAM management services. This was specified page 5, line 20.  b. The primary objective of PBF-N was to improve the performance of MAM and SAM services. These services take in charge children of all ages, mostly from six months to five years old. That’s the reason why we chose this age range. Now, for the outcome at community level, we aimed at having an important explanatory power and we focused on the age group that had the highest prevalence of acute malnutrition (that is children aged 6-23 months). This sentence was added page 14 to make that clear:  *At the community level, for statistical power issues, the focus was made on the age group with the highest prevalence, where we could expect the largest impact, that is, children aged 6-23 months.*  c. This information was added in the text as well as in the figure.  d. This was changed accordingly.  e. This text was added page 8, line 18-page 9, line 2:  *PBF-N subsidies were transferred to the bank accounts of hospitals, HCs and CHW associations. Revenues from the PBF-N were fungible with other revenues raised by the hospitals and HC (the national PBF scheme, user fees, etc.); their use is under the responsibility of the facility staff who based their allocation decisions on a management tool prioritising purchase of key inputs (e.g. medicines); surplus (if any) is distributed to staff as bonuses, again using a specific management tool. As for CHW associations, one part of the funds could be invested into revenue-generating activities while the rest was shared among CHWs.*  f. The MAM treatment supply is a mixture of corn-soy blend, sugar and oil (that were all supplied by the WFP before). This was specified page 15, lines 14-15.  g. Indeed, there was an error in the cut-offs, this was fixed accordingly.  Height is for children above 24 months, yes, but in the text we mostly refer to anthropometrics that were taken on children aged 6-23 months (for the household survey) – however, to make it clearer and more comprehensive, we added this distinction, since we also used anthropometrics at HC level, so for children aged 6-59 months (see page 14).  h. This was made clear pages 6 and 8.  i. These indicators are now added as supplementary data (S2 File).  j. Indeed, CHWs are to screen and refer acute malnutrition cases to HCs. This is an outreach activity, to sensitize and to reach children who do not come to the HC regularly. Moreover, growth monitoring at the HC level was rarely done and the PBF-N intervention did not help in that direction.  k. The research protocol as approved by the ethics committee is requested by the journal for RCTs, so we will keep in the Supporting information the four files (S3-S6 files) related to the research protocol: as stated above -comment #19-, there were two separate research protocols for this study, one at the health centre level and one at the community level; both original research protocols were written in French; but there were also an English version, lighter, of both research protocols, that were approved by the University of Antwerp Ethics committee. |
| 28 | **4. Results** a. On p. 18, there is a distinction of the intervention at hospital, HC, and community level. This is why the specific activities expected at these three levels have to be described in the methods section.  b. Fig. 3 is not very useful.  c. What about the % of defaulters in MAM management, in intervention vs control HCs?  d. It has to be clear that the improved SAM management performance only refers to uncomplicated SAM treatment outside the hospital since control hospitals also get PBF for the management of SAM cases with complications.  e. In Table 2, what is the meaning of **?  f. What about infant and child feeding practices in the community survey? It is referred to it in the discussion (p. 32, line 29) but the results are not shown. | a. Page 8, we edited to make the intervention clearer at all levels and still refer to Table 1 which provides the quantitative indicators, while we now provide also the 19 qualitative indicators as supplementary data.  b. About figure 3, we removed it, as we think indeed it is not useful anymore: the main message is in the text, and this same figure was published in [3].  c. At baseline, according to the clinical files for which an exit reason was reported, the % of defaulters was 4.4% in the intervention group and 4.9% in the control group; at endline, it was 0.1% in the treatment group (4 occurrences / 327 obs) and 3.6% in the control group (4 occurrences / 112 obs). The impact estimates were not significant.  d. We now specified “uncomplicated” whenever it applied (in Findings, Table 2, and Discussion).  e. The ** was removed.  f. There was absolutely no difference in feeding practices between the two groups, no significant impact. We made that clearly by editing the last sentence of the paragraph you refer to:  *PBF-N did not address these issues, mainly because this goes beyond its scope, this is not a mechanism that can address socio-economic determinants such as wealth, education of mothers and number of children*  At both baseline and endline, about one quarter of children had correct child feeding practices (both in terms of frequency and groups diversity). Figures are added in the supplementary table 1. |
| 29 | **5. Discussion and conclusion** a. Training of health personnel (although recent training apparently had no impact) and supervision of CHWs would appear to be major conditions of performance that the PBF strategy did not address. This should be discussed in more depth.  b. In the recommendations, the need for health centre staff training and CHW supervision should be emphasized, along with having personnel specifically for health promotion.  c. Is the fact the control hospitals and HCs also received subsidies not a limitation of the study, although this was intended to clearly identify the impact of the monetary incentives’?  d. The short duration of the intervention (2 years) may not have allowed to show an impact on stunting; this can be regarded as a limitation of the study.  e. P. 31, line 19: reference needed  f. P. 31, line 21: What equipment?  g. P. 32, line 27: also failures in WASH, not only food security  h. P. 32, line 40: “CHW strategy got a boost from PBF”: Please explain | a. We added this page 33:  *At facility level, during paediatric consultations, health workers’ practices towards malnutrition detection were found to be weak, and it appeared that PBF-N had no impact on this: this suggests that health workers capacities were poor.*  There is no indication about whether PBF-N addressed supervision of CHWs.  b. We edited the paragraph page 34 accordingly:  *Better detection will come from improved collaboration with and supervision of CHWs, growth monitoring and capacity to perform integrated management of childhood illnesses, with having personnel specifically in charge of health promotion. Staff technical capacities are a bottleneck, and there is an urgent need for specific training and closer supervision.*  c. The intention of providing revenues (without incentives) to control HCs was indeed to isolate and clearly identify the “incentive” mechanism of PBF. The control HCs had the autonomy to spend these additional revenues to any service (not specifically nutrition related) and, through their contract, they were not encouraged to perform better within growth monitoring and acute malnutrition screening and management activities – although, proactively thinking that PBF-N may be scaled-up in the end, they could have taken action in advance to improve their nutrition services. We think the limitation is minor.  As for the control hospitals, they actually received PBF-N for the management of complicated SAM cases; however, our focus was made on HCs, and even though hospitals may have encouraged control HCs to refer their complicated SAM cases, this does not seem to have played an important role.  d. Indeed. This was added in the limitations:  *At the community level, the short duration of the intervention (two years) may not have allowed to show an impact on chronic malnutrition, although we believe other factors than the health system itself, like food security, water, sanitation and hygiene elements, have a much more important to play.*  e. We added a reference to a systematic review [6].  f. We added the following details page 32: *“e.g. sets of anthropometrics’ equipment -with scales, measuring boards, WHZ tables, etc.- were not fully available and functional in most of the HCs”*  g. Indeed, WASH factors were added. We also illustrated with some figures on WASH in rural Burundi, page 33:  *Improved water source and sanitation are accessible by respectively 82% and 39% of rural inhabitants* [7] *and according to our findings, 65% of them own soap.*  h. This was developed as follows, page 33-34:  *This finding also suggests that the CHW strategy did get a boost from the PBF-N intervention, both at community and HC level, i.e. both through the provision of revenues, conditional on performance, to CHWs, and the PBF-N intervention at the health centre level which encouraged some HCs to work with and integrate CHWs to their malnutrition screening activity.* |

**References used in the responses**

1.	Hayes RJ, Bennett S. Simple sample size calculation for cluster-randomized trials. Int J Epidemiol [Internet]. 1999 Apr [cited 2013 Aug 30];28(2):319–26. Available from: http://www.ncbi.nlm.nih.gov/pubmed/10342698

2.	Nimpagaritse M, Korachais C, Roberfroid D, Kolsteren P, Zine Eddine El Idrissi MD, Meessen B. Measuring and understanding the effects of a performance based financing scheme applied to nutrition services in Burundi—a mixed method impact evaluation design. Int J Equity Health [Internet]. 2016;15(1):93. Available from: http://equityhealthj.biomedcentral.com/articles/10.1186/s12939-016-0382-0

3.	Nimpagaritse M, Korachais C, Meessen B. Effects in spite of tough constraints - A theory of change based investigation of contextual and implementation factors affecting the results of a performance based financing scheme extended to malnutrition in Burundi. PLoS One. 2020;15(1):e0226376.

4.	Cumming O, Cairncross S. Can water, sanitation and hygiene help eliminate stunting? Current evidence and policy implications. Matern Child Nutr [Internet]. 2016;12:91–105. Available from: http://doi.wiley.com/10.1111/mcn.12258

5.	Bhutta ZA, Das JK, Rizvi A, Gaffey MF, Walker N, Horton S, et al. Evidence-based interventions for improvement of maternal and child nutrition: what can be done and at what cost? Lancet [Internet]. 2013;382(9890):452–77. Available from: https://www.sciencedirect.com/science/article/pii/S0140673613609964

6.	Hossain M, Choudhury N, Abdullah KAB, Mondal P, Jackson AA, Walson J, et al. Evidence-based approaches to childhood stunting in low and middle income countries: A systematic review. Arch Dis Child. 2017 Oct 1;102(10):903–9.

7.	MPBGP, MSPLS, ISTEEBU, ICF. Burundi Troisième Enquête Démographique et de Santé 2016-2017 [Internet]. 2017 [cited 2018 Oct 10]. Available from: https://www.dhsprogram.com/publications/publication-FR335-DHS-Final-Reports.cfm
